# Conurbation, Urban, and Rural Living as Determinants of Allergies and Infectious Diseases: Royal College of General Practitioners Research and Surveillance Centre Annual Report 2016-2017

**DOI:** 10.2196/11354

**Published:** 2018-11-26

**Authors:** Simon de Lusignan, Christopher McGee, Rebecca Webb, Mark Joy, Rachel Byford, Ivelina Yonova, Mariya Hriskova, Filipa Matos Ferreira, Alex J Elliot, Gillian Smith, Imran Rafi

**Affiliations:** 1 Department of Clinical and Experimental Medicine University of Surrey Guildford United Kingdom; 2 Royal College of General Practitioners Research and Surveillance Centre London United Kingdom; 3 Real-time Syndromic Surveillance Team, Field Service National Infection Service Public Health England Birmingham United Kingdom; 4 Royal College of General Practitioners Clinical Innovation and Research Centre London United Kingdom

**Keywords:** population surveillance, respiratory tract infections, conjunctivitis, allergic, asthma, urinary tract infections, gastroenteritis, healthcare disparities, socioeconomic factors, social determinants of health, medical records systems, computerized, data collection, records as topic, primary health care, general practice, infectious diseases

## Abstract

**Background:**

Living in a conurbation, urban, or rural environment is an important determinant of health. For example, conurbation and rural living is associated with increased respiratory and allergic conditions, whereas a farm or rural upbringing has been shown to be a protective factor against this.

**Objective:**

The objective of the study was to assess differences in general practice presentations of allergic and infectious disease in those exposed to conurbation or urban living compared with rural environments.

**Methods:**

The population was a nationally representative sample of 175 English general practices covering a population of over 1.6 million patients registered with sentinel network general practices. General practice presentation rates per 100,000 population were reported for allergic rhinitis, asthma, and infectious conditions grouped into upper and lower respiratory tract infections, urinary tract infection, and acute gastroenteritis by the UK Office for National Statistics urban-rural category. We used multivariate logistic regression adjusting for age, sex, ethnicity, deprivation, comorbidities, and smoking status, reporting odds ratios (ORs) with 95% CIs.

**Results:**

For allergic rhinitis, the OR was 1.13 (95% CI 1.04-1.23; *P*=.003) for urban and 1.29 (95% CI 1.19-1.41; *P*<.001) for conurbation compared with rural dwellers. Conurbation living was associated with a lower OR for both asthma (OR 0.70, 95% CI 0.67-0.73; *P*<.001) and lower respiratory tract infections (OR 0.94, 95% CI 0.90-0.98; *P*=.005). Compared with rural dwellers, the OR for upper respiratory tract infection was greater in urban (OR 1.06, 95% CI 1.03-1.08; *P*<.001) but no different in conurbation dwellers (OR 1.00, 95% CI 0.97-1.03; *P*=.93). Acute gastroenteritis followed the same pattern: the OR was 1.13 (95% CI 1.01-1.25; *P*=.03) for urban dwellers and 1.04 (95% CI 0.93-1.17; *P*=.46) for conurbation dwellers. The OR for urinary tract infection was lower for urban dwellers (OR 0.94, 95% CI 0.89-0.99; *P*=.02) but higher in conurbation dwellers (OR 1.06, 95% CI 1.00-1.13; *P*=.04).

**Conclusions:**

Those living in conurbations or urban areas were more likely to consult a general practice for allergic rhinitis and upper respiratory tract infection. Both conurbation and rural living were associated with an increased risk of urinary tract infection. Living in rural areas was associated with an increased risk of asthma and lower respiratory tract infections. The data suggest that living environment may affect rates of consultations for certain conditions. Longitudinal analyses of these data would be useful in providing insights into important determinants.

## Introduction

### Urbanization as a Determinant of Health

There is a wide range of social determinants of health. Conurbation, urban, and rural living are important among these, although their different effects are still unclear [1]. Urbanization is increasing, and the United Nations has predicted that the world urban population will double between 2007 and 2050. Urbanization is an important determinant of health [2], as it may create incubators for infectious disease [3].

Factors associated with urban and rural living contribute to differences in respiratory and allergic conditions. Pollution, climate change, and pollen exposure are all associated with increased respiratory and allergic conditions [4-6]. Farm and rural upbringing have been shown to be protective against allergic rhinitis compared with urban living [7,8]. The same gradient has been reported for asthma in northern Europe [9]. However, asthma has also been shown to have an increasing incidence with higher levels of air pollution, but there is inconsistency between age groups for specific pollutants [10,11]. Pollen levels may also be important in precipitating exacerbations of asthma [12], and there may be a complex interaction between them and with the weather [13]. Much less is known about the impact of conurbation, urban, and rural living on upper respiratory tract infection (URTI), gastroenteritis, or urinary tract infection (UTI).

The UK Royal College of General Practitioners (RCGP) Research and Surveillance Centre (RSC) is one of the oldest sentinel networks and is in its 50th season of reporting infections and respiratory conditions [14,15]. This is a long-standing collaboration with Public Health England [16,17]. The network is recruited to be nationally representative and, at the time of this report, comprised over 1.6 million registered patients. The network’s capabilities include reporting whether patients live in a conurbation, urban, or rural area.

### Objective

We carried out this study to determine whether exposure to living in a conurbation (high-density living), urban (intermediate density, such as a city or town), or rural (least dense, such as the countryside) environment was associated with more presentations to a general practice (GP) of allergic (allergic rhinitis and asthma) or common infectious conditions. This investigation is the theme of the RSC’s annual report on diseases. The annual report also includes the annual weekly rates of GP presentations of all our monitored conditions (Multimedia Appendix 1).

## Methods

### Design, Setting, and Ethical Considerations

We extracted data from 175 volunteer GPs that are members of the RCGP RSC, with a cohort of 1,602,366 patients registered for the first 6 months of the period of April 1, 2016 to March 31, 2017. All data are pseudonymized as close to source as possible. Data were coded with Read version 2 or Clinical Terms version 3 [18]. We only extracted coded data, not free text. Disease surveillance is part of standard health service activity, so no specific ethical approval was needed. No personal identifiers are held on the RCGP RSC secure network at the University of Surrey. We did not process the data of patients who had an opt-out code (2.2% of the RCGP RSC population).

### Data Preparation

We determined a patient’s urban classification by using a UK Office for National Statistics (ONS) lookup tool [19]. We did this on an individual-patient level basis using the ONS’s Lower Super Output Area to estimate population density. Based on this lookup tool, if a patient’s population was classified as mainly rural or largely rural, we classified them as living in a rural population. If a patient lived in an urban with significant rural, urban with city and town, or urban with minor conurbation area, we classified them as living in a city or town (referred to as rural throughout this paper). If a patient lived in an urban with major conurbation area, we classified them as living in a conurbation. These were based on the ONS Lower Super Output Area, which has a mean size in England and Wales of 1640, with population sizes ranging from 820 in South Cambridgeshire to 8250 in Oxford [20].

Our outcome variables, presentation to a GP for allergic and infectious conditions, were a composite of similar conditions grouped together, a method we adopted for the 2016-2017 annual report. To identify our outcomes, we used Read version 2 codes and Clinical Terms version 3 codes to extract the data. These codes are based on *International Classification of Diseases, Tenth Revision* codes. Allergic conditions were allergic rhinitis (including hay fever) and asthma. We divided infections into lower respiratory tract infections (LRTIs), comprising acute bronchitis, pneumonia, and pleurisy; URTIs, including tonsillitis, common cold, sinusitis, conjunctivitis, and otitis media; acute gastroenteritis (AGE); and UTI. We did not include influenza-like illness in this analysis, although data about these illnesses are contained in the annual report (Multimedia Appendix 1), as we plan a separate analysis taking into account vaccine exposure. Similarly, we excluded less-common conditions (eg, measles, mumps, scabies), although their weekly rates of GP presentation are included in the annual report.

In exploring the association between living area and allergic and infectious diseases, we adjusted for age, sex, ethnicity, and socioeconomic status using the Index of Multiple Deprivation (IMD). The IMD is the official measure of relative deprivation for areas in England. It uses 7 domains of deprivation to produce an overall measure (income, employment, education, health, crime, housing and services, and living environment) [21]. We grouped these variables as follows: sex (female was the reference group); age bands (1-4 years, 5-17 years, 18-64 years, and ≥65 years; the reference was 18-64 years); ethnicity (white ethnicity was the reference; we divided the others into Asian, Black, mixed, other, and unclassified ethnicities) using an ontological approach to maximize identification [22]; and deprivation. Using the IMD, we divided deprivation into quintiles (quintile 1, most deprived, was the reference).

From the cohort of 1,602,366 patients registered, we compiled and reported data on conurbation, urban, and rural living by age, sex, ethnicity, and IMD score. We also controlled for comorbid disease. We grouped comorbidities into the following groups: 0 comorbidities (reference), 1 to 2 comorbidities, and 3 or more comorbidities. We included the following as comorbidities: depression; hypertension; chronic obstructive pulmonary disease; rheumatoid arthritis; dementia; stroke or transient ischemic attack (grouped as cerebrovascular disease); acute myocardial infarction, angina, and coronary artery disease (grouped as ischemic heart disease); congestive cardiac failure; peripheral arterial disease; chronic kidney disease; diabetes mellitus; and atrial fibrillation. We also included and controlled for smoking status in our analysis, grouping smokers into active smokers (reference), ex-smokers, nonsmokers, and unknown, based on their latest recorded smoking habit. We used these comorbidities because they are quality and outcomes framework indicators that are used to rate GP performance [23,24]. These conditions are representative of common chronic diseases and likely to be consistently recorded between practices.

### Statistical Analysis

To understand whether rural, urban, or conurbation living was associated with GP presentation for certain allergic or contagious diseases, we carried out a multivariate logistic regression, with rural, urban, or conurbation as the predictor variable and disease as the outcome variable. We report the odds ratio (OR) and 95% CI from the multivariate logistic regression [25] for conurbation, or urban compared with rural (reference). An OR greater than 1 implies greater odds of a patient living in a conurbation or an urban area presenting with the condition, and an OR of less than 1 suggests lower odds of a patient living in a conurbation or an urban area presenting with a condition, adjusting for other variables in the model. We created an aggregated table showing those conditions with significant results highlighted. Given the large number of models, we applied a Benjamini-Hochberg correction [26]. We also report probability (*P* value), which we calculated from the coefficients of the logistic regression.

In addition to the main effect of urban, rural, and conurbation living on GP presentation, we looked at the interaction of age band or sex and urban, rural, and conurbation living on GP presentation (see Multimedia Appendix 2 for detailed results). We also created forest plots for age bands and each of the conditions (Multimedia Appendix 3).

The analysis presented in the annual report (Multimedia Appendix 1) includes the following: (1) a map of the national distribution of RCGP RSC practices; (2) summary tables showing the conditions we monitor (median age, using horizontal box and whisker plots; sex distribution of our monitored conditions; ethnicity distribution, comparing white versus all other ethnicities; median IMD, using a horizontal box and whisker plot; and conurbation, urban, and rural distribution of our monitored conditions); and (3) weekly GP presentation rates of the conditions monitored by the RCGP RSC. Population denominators were based on the population registered in the participating practices in December. The weeks are numbered using the International Organization for Standardization system [27].

## Results

### Population

The RCGP RSC network population consists of 1,602,366 people. Older (>65 years: n=68,378, 25.01%), less deprived (IMD score ≥3: n=274,349, 25.62%), and less ethnically mixed (white: n=204,954, 20.8%; black: n=528, 1.00%) populations live in rural areas. In comparison, younger (25-44 years: n=182,322, 40.7%), ethnically mixed (black: n=44,690, 88.7%; Asian: n=30,827, 67.4%), and more deprived (IMD score <3: n=280,714, 52.81%) populations live in conurbations (see Multimedia Appendix 2, table B.7, and Multimedia Appendix 3, figures C.5-C.7).

### Main Effect

Those living in a conurbation, in comparison with a rural area, had greater odds of presenting to a GP with allergic rhinitis (OR 1.29, 95% CI 1.19-1.41; *P<*.001) but had lower odds of presenting with asthma (OR 0.70, 95% CI 0.67-0.73; *P<*.001) and LRTI (OR 0.94, 95% CI 0.90-0.98; *P=*.005).

**Figure 1:**
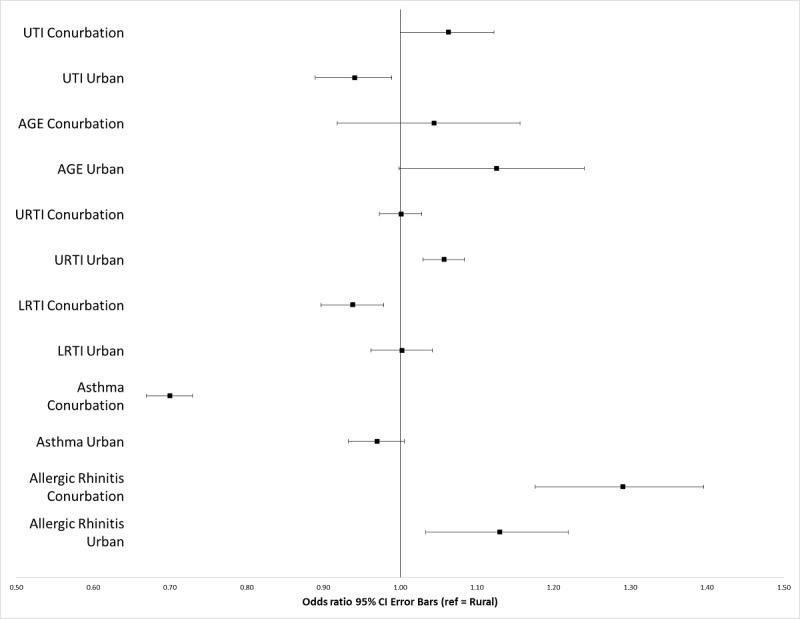
Forest plot showing odds ratios for various allergic and infectious diseases based on living area. AGE: acute gastroenteritis; LRTI: lower respiratory tract infection; ref: reference; URTI: upper respiratory tract infection; UTI: urinary tract infection.

Those living in urban, compared with rural, areas had greater odds of presenting to a GP with allergic rhinitis (OR 1.13, 95% CI 1.04-1.23; *P=*.003), URTI (OR 1.06, 95% CI 1.03-1.08; *P<*.001) and AGE (OR 1.13, 95% CI 1.01-1.25; *P=*.03). On the other hand, urban dwellers were less likely to present to a GP with UTI (OR 0.94, 95% CI 0.89-0.99; *P=*.02; Table 1). Figure 1 displays these main effects in more detail.

### Interaction Effects

We found no interactions between sex and living area, although we did find 4 interactions for age band and living area, using rural and working age (18-64 years) as reference groups. Children aged 0 to 4 years living in urban areas were more likely to present to a GP with asthma than were adults aged 18 to 64 years living in rural areas (OR 1.42, 95% CI 1.20-1.68; *P<*.001).

**Table 1:** Odds ratios (ORs) and 95% CIs of the main effect of conurbation and urban living (rural is the reference) on the 6 conditions of interest.

Conditions of interest	Conurbation		Urban area	
	OR (95% CI)	*P* value^a^	OR (95% CI)	*P* value^a^
Allergic rhinitis	1.29 (1.19-1.41)^b^	<.001^b^	1.13 (1.04-1.23)^b^	.003^b^
Asthma	0.70 (0.67-0.73)^c^	<.001^c^	0.97 (0.93-1.01)	.11
Lower respiratory tract infection	0.94 (0.90-0.98)^c^	.005^c^	1 (0.96-1.04)	.89
Upper respiratory tract infection	1 (0.97-1.03)	.93	1.06 (1.03-1.08)^b^	<.001^b^
Acute gastroenteritis	1.04 (0.93-1.17)	.46	1.13 (1.01-1.25)^b^	.03^b^
Urinary tract infection	1.06 (1.00-1.13)^b^	.04^b^	0.94 (0.89-0.99)^c^	.02^c^

^a^*P* value adjusted using Benjamini-Hochberg correction.

^b^OR>1 and significant adjusted *P* value.

^c^OR<1 and significant adjusted *P* value.

From the results of the logistic regression (Multimedia Appendix 4), we could infer the odds of children in certain areas presenting with asthma. For example, there was a 27% decrease in the odds of 0- to 4-year-olds presenting with asthma if they lived in rural compared with urban areas. Children aged between 5 and 17 years were more likely to consult for URTI than adults aged 18 to 64 years living in rural areas (OR 1.06, 95% CI 1.02-1.11; *P=*.01). On the other hand, children aged 0 to 4 years living in urban areas were less likely to present with AGE than were adults aged 18 to 64 living in rural areas (OR 0.84, 95% CI 0.72-0.98; *P=*.03). Over-65-year-olds living in urban areas were less likely to consult for URTI (OR 0.92, 95% CI 0.88-0.97; *P<*.001) and AGE (OR 0.85, 95% CI 0.72-0.99, *P=*.04) than was our reference group (Table 2). Children aged 0 to 4 years living in a conurbation were more likely to consult for URTI (OR 1.25, 95% CI 1.19-1.31; *P<*.001) than were 18- to 64-year-olds living in rural areas. Children aged 5 to 17 living in conurbations were more likely to consult for asthma (OR 1.14, 95% CI 1.05-1.23; *P=*.001), LRTI (OR 1.32, 95% CI 1.18-1.47; *P<*.001), URTI (OR 1.25, 95% CI 1.20-1.31; *P<*.001), and AGE (OR 1.64, 95% CI 1.36-1.98; *P<*.001) than was our reference group. Additionally, those aged over 65 living in conurbations were more likely to consult for asthma (OR 1.29, 95% CI 1.21-1.39; *P<*.001) and LRTI (OR 1.14, 95% CI 1.08-1.21; *P<*.001) than was our reference group (Table 3).

**Table 2:** Odds ratios (ORs) and 95% CIs of the main effect of urban (rural is the reference) and interaction terms of urban area with age band (18-64 years is the reference) on the 6 conditions of interest.

Conditions of interest	0-4 years		5-17 years		≥65 years	
	OR (95% CI)	*P* value^a^	OR (95% CI)	*P* value^a^	OR (95% CI)	*P* value^a^
Allergic rhinitis	0.81 (0.63-1.04)	.09	0.93 (0.83-1.05)	.23	1.08 (0.94-1.25)	.29
Asthma	1.42 (1.20-1.68)	<.001	1.05 (0.98-1.13)	.18	0.96 (0.91-1.02)	.18
Lower respiratory tract infection	0.99 (0.91-1.08)	.85	1.11 (1.00-1.23)	.06	1.03 (0.99-1.09)	.17
Upper respiratory tract infection	1.03 (0.99-1.08)	.16	1.06 (1.02-1.11)	.01	0.92 (0.88-0.97)	<.001
Acute gastroenteritis	0.84 (0.72-0.98)	.03	1.01 (0.84-1.22)	.92	0.85 (0.72-0.99)	.04
Urinary tract infection	1.100 (0.87-1.38)	.43	1 (0.86-1.17)	.97	0.98 (0.91-1.05)	.58

^a^*P* value adjusted using Benjamini-Hochberg correction.

**Table 3:** Odds ratios (ORs) and 95% CI of the main effect of conurbation (rural is the reference) and interaction terms of conurbation with age band (18-64 years is the reference) on the 6 conditions of interest.

Conditions of interest	0-4 years		5-17 years		≥65 years	
	OR (95% CI)	*P* value^a^	OR (95% CI)	*P* value^a^	OR (95% CI)	*P* value^a^
Allergic rhinitis	0.84 (0.65-1.07)	.16	0.94 (0.83-1.05)	.27	0.99 (0.85-1.17)	.94
Asthma	1.08 (0.90-1.29)	.43	1.14 (1.05-1.23)	.001	1.29 (1.21-1.39)	<.001
Lower respiratory tract infection	0.98 (0.90-1.07)	.68	1.32 (1.18-1.47)	<.001	1.14 (1.08-1.21)	<.001
Upper respiratory tract infection	1.25 (1.19-1.31)	<.001	1.25 (1.20-1.31)	<.001	1.02 0.97-1.08()	.40
Acute gastroenteritis	0.94 (0.80-1.10)	.45	1.64 (1.36-1.98)	<.001	0.93 (0.78-1.12)	.45
Urinary tract infection	1.13 (0.89-1.43)	.31	1.04 (0.89-1.22)	.61	1.02 (0.94-1.10)	.63

^a^*P* value adjusted using Benjamini-Hochberg correction.

## Discussion

### Principal Findings

Patients living in conurbations or urban areas were more likely to consult for allergic rhinitis and URTI, after adjustment for age, sex, ethnicity, socioeconomic status, comorbid disease, and smoking status. The OR of presenting with allergic rhinitis increased with population density. While living in rural areas was associated with an increased risk of asthma and LRTI, both conurbation and rural living were associated with an increased risk of UTI.

Age and living environment interacted when predicting the GP presentation rates of these conditions. Children living in urban areas were more likely to consult for asthma (0-4 years) and URTI (5-17 years) than were 18- to 64-year-old adults living in rural areas (our reference group). Additionally, children living in conurbations were more likely than our reference groups to consult for URTI (0-17 years), LRTI, asthma, and AGE (5-17 years). Over-65-year-olds living in conurbations were also more likely than our reference group to consult for asthma. The risk of AGE was increased in 18- to 64-year-olds living in rural areas in comparison with 0- to 4-year-olds and over-65-year-olds living in urban areas. Rural living for 18- to 64-year-olds was associated with an increased risk of URTI compared with over-65-year-olds living in rural areas.

### Comparison With Prior Work

Conurbation and urban living was associated with increased presentation with allergic rhinitis to a GP. This is consistent with previous research finding that allergic rhinitis is more common in urban areas and conurbations [8,28].

Those living in conurbations had higher odds of consulting for UTI. Conurbation living is arguably very different from rural living. For example, the population density is higher [29], the nightlife is more active [30], and the levels of risky sexual behavior are higher in conurbations [31,32]. As one of the risk factors for UTIs in women is sexual intercourse [33-35], the lifestyle of conurbation living may explain this finding. However, more research is needed to test this.

The results also showed that those living in rural areas were more likely to present with LRTI and asthma. Some studies have found that urban living is associated with increased odds of developing asthma [36], whereas others have found that rural living increases the odds [37]. Clearly more research is needed to identify environmental risk factors for developing asthma. Risk factors for LRTI vary across different studies. For example, risk factors for developing LRTI in children have been found to be pollution, poor ventilation [38], living in urban areas, and parental smoking [39,40]. In older people, difficulty taking medication and poor mobility were risk factors [41]. Based on these risk factors, it is difficult to understand our findings, as pollution and poor ventilation are more likely to be factors found in conurbations. However, looking at preventive factors for developing LRTI may explain the results. For example, research has found that influenza vaccination can be a protective factor against LRTI [41,42]. Furthermore, some studies have found that individuals living in rural areas are less likely to obtain preventive health services such as the influenza vaccine [43-45]. This may possibly explain our findings, although more research is needed.

Infectious diseases are associated with population density [3]; therefore, the increased odds of AGE in adults aged 18 to 64 years living in rural areas does not fit with previous research. A possible explanation may be related to food-borne illness. Risk factors for certain food poisoning–related bacteria include eating restaurant-prepared food, eating undercooked food, drinking raw milk, having contact with farm animals, and travelling abroad [46,47], factors that may be associated with rural living [48,49].

### Implications of the Findings

Living in a conurbation or an urban area leads to an increased risk of allergic rhinitis and URTI in all people, and an increased risk of URTI, LRTI, asthma, and AGE in children. These results are in line with previous research, as densely populated areas have been associated with the rapid spread of infectious diseases such as the severe acute respiratory syndrome virus and avian flu [3]. Future research should therefore focus on aiming to reduce infection spread in high-density populations.

Furthermore, population density and traffic in conurbations may increase the rates of allergic rhinitis and asthma [4-6]. Increasing the number of green spaces may be an important preventive measure [50], as they have been found to prevent higher rates of asthma [51,52] and allergic rhinitis [53,54].

### Strengths and Limitations

We derived the data from a network of general practitioners in which the population in question is large and is representative of the whole of England. This large and representative population allows us to link morbidity to ethnicity, living environment, and socioeconomic status. Patterns found from this dataset can be applied to the whole population.

Further, data quality in the RCGP RSC for infections and allergic conditions is assured through data quality feedback to RSC member practices. More recently, we have introduced financially incentivized training and practice-specific comparative feedback via a dashboard [55], modelled on the principles of audit-based education [56].

The limitations of this study were that not everyone who has infectious or allergic diseases will go to their GP, meaning that actual rates of illness may have been higher in the general population. Furthermore, although we worked hard to ensure accuracy of our data, there were instances where conditions were not recorded accurately. Additionally, the allergic conditions we investigated tend to be chronic conditions, with peaks of exacerbations. We did not control for episode type in our analysis, which may have confounded rates of GP presentation for asthma and allergic rhinitis.

### Conclusions

Overall, we found that different allergic and infectious conditions were associated with rural, urban and conurbation living. A longitudinal study of RCGP RSC data may provide insights, particularly around changes in pollutant emissions or other variations in exposure, on the effect of the environment on allergic and infectious conditions.

## Appendix

Multimedia Appendix 1 RCGP RSC Annual Report 2016-2017.

Multimedia Appendix 2 Data tables.

Multimedia Appendix 3 Additional figures.

Multimedia Appendix 4 Logistic regression output.

## Abbreviations

AGE: acute gastroenteritis
GP: general practice
IMD: Index of Multiple Deprivation
LRTI: lower respiratory tract infection
ONS: Office for National Statistics
OR: odds ratio
RCGP: Royal College of General Practitioners
RSC: Research and Surveillance Centre
URTI: upper respiratory tract infection
UTI: urinary tract infection
