# Supplementary material for: Conurbation, Urban, and Rural Living as Determinants of Allergies and Infectious Diseases: Royal College of General Practitioners Research and Surveillance Centre Annual Report 2016-2017
Source: JMIR Public Health Surveill. 2018 Nov 26;4(4):e11354. doi: 10.2196/11354 (PMC6288591; doi:10.2196/11354)
Supplement: Multimedia Appendix 1 [file publichealth_v4i4e11354_app1.pdf]

REGISTERED
CHARITY

NUMBER 223106

RCGP

Royal College of General Practitioners

Annual Report 2016-2017

Research & Surveillance Centre

Weekly Returns Service Annual Report 2016-2017

Contents

1. Introduction 2

2. What we do – and why 4

Measuring vaccine effectiveness 4

Monitoring disease incidence 4

Collecting samples of viruses and disease-causing organisms 4

Identifying disparities 5

Helping to change clinical practice 5

3. The RSC dataset 6

Our data extraction process and information governance 6

The dataset used for the RSC Annual Report 2016/17 6

Reporting period 6

4. Sample population 7

5. Influenza 9

Swabbing programme 9

Vaccine uptake 10

Vaccine effectiveness 11

6. Disparities 12

Age 12

Gender 12

Ethnicity 12

Deprivation 13

Urban, Rural or Conurbation Living 13

7. Disease incidence 19

8. Episode typing – key part of data quality 21

9. Current Projects 23

European Projects 23

Other Projects 23

10. RCGP Publications 2016/17 24

11. Participating practices 25

12. Contributors 30

13. Appendices 31

1. Introduction

Welcome to the Royal College of General Practitioners (RCGP) Research and Surveillance Centre's (RSC) Annual Report for 2016-17. This is our 51st year of surveillance.

This Annual Report is based on the data that we extract from more than 200 general practices and draws together the principal elements of our work – disease surveillance, virological sampling, and vaccine effectiveness. It covers the period from the 9th May 2016 to the 15th May 2017, which includes the whole of the winter flu season 2016-17.

We would like to thank all the practices who form our network, and of course all their patients, without whose data (which we utilise in an anonymised format) our work would be impossible. Sharing of medical data has become a controversial topic over recent years, but without the support of our member practices and the use of their patients' data we would not be able to carry out our crucial work of monitoring disease rates, assuring the effectiveness of national vaccination programmes, and participating in other research programmes.

Influenza surveillance is the biggest part of our work. We have also been involved in research testing enhanced surveillance for gastroenteritis, within a Wellcome Trust/Department of Health funded project called Integrate[1]. This involved the near real-time detection of many of the causative organisms for this condition. Practice recruitment was carried out from July 2015 until February 2016, when the recruitment target of 60 was successfully met. By January 2017, 56 practices remained in the network due to practices dropping out for various reasons. The successful recruitment and retention of practices within the network enabled surveillance of gastroenteritis to be completed in real time, enabling the next phase of the Integrate project to be completed.

In addition to our surveillance on influenza and gastroenteritis, we produce a dashboard for general practices involved in our network so they can see real-time feedback of the number of microbiological samples they have provided in comparison to the whole RCGP RSC network[2]. We are also involved in benefit-risk monitoring of accelerated development vaccinations[3].

The RCGP RSC has had a couple of years of immense change including a re-design and rebuilding of its data extraction systems and creation of a new data and analytics hub at University of Surrey. This report is based on an extract taken at the end of May 2017. The data presented is an amalgamation of the data from the weekly surveillance reports.

The RCGP RSC works in close collaboration with Public Health England (PHE). The principal source of funding of the RCGP RSC is from PHE as their principal primary care surveillance element. This surveillance contributes to decisions about when flu starts to circulate in the community. The Chief Medical Officer can then state whether there is circulating influenza, which triggers permission being given to general practices to prescribe oral medication for influenza, where they feel appropriate. The

---

[1] http://www.integrateproject.org.uk/

[2] Pathirannehelage S, Kumarapeli P, Byford R, Yonova I, Ferreira F, de Lusignan S. Uptake of a dashboard designed to give realtime feedback to a sentinel network about key data required for influenza vaccine effectiveness studies. 2018. Stud Health Technol Inform 247:161-165. https://www.ncbi.nlm.nih.gov/pubmed/29677943

[3] https://www.imi.europa.eu/projects-results/project-factsheets/advance

RCGP RSC is also looking out for any signs of an influenza pandemic, or any other unexpected rise across a range of diseases.

The innovation we have added to this year's report are new graphics displaying how people who live in different areas tend to present to practices with different conditions (See pages 13, 18 & 20). We hope these graphics will provide insights into patterns of health and disease. The accompanying paper describes this pattern in detail.

2. What we do – and why

For 50 years, the RCGP's Research and Surveillance Centre has used data from general practices across England to monitor disease, measure vaccine effectiveness, and for research. The RCGP RSC is the principal primary care research surveillance system for England; for example, its data makes a major contribution to the announcement by the Chief Medical Officer that there is circulating influenza. This announcement provides the cue to practitioners that they can prescribe oral antiviral treatments for influenza. The robust evidence that we produce enables public health agencies and others to make the best possible decisions in preventing disease and responding to epidemics; alongside this, we contribute to a range of research studies.

Measuring vaccine effectiveness

Vaccination helps protect populations from disease and saves lives every year. Data from RCGP RSC practices is crucial for ensuring that vaccines are as effective as possible:

- The childhood flu vaccination programme was introduced in 2013/14 following successful pilots. This is also known as the live attenuated influenza vaccine (LAIV) programme. Data from RCGP RSC practices provided evidence that the pilot scheme should be rolled out across the country, and is still being used to monitor the ongoing effectiveness of the scheme. (See pgs. 10-11.)
- Data from RCGP RSC practices also helps to monitor the effectiveness of the seasonal flu vaccine for adults. (See pgs. 10-11.)
- Disease does not stop at national borders – international collaboration is key in maximising vaccine effectiveness. We are involved in two projects to put into place European frameworks that will help health professionals, regulatory agencies, public health institutions, vaccine manufacturers, and the general public make more informed decisions on the benefits and risks of vaccines. (See p. 23)

Monitoring disease incidence

We monitor 37 communicable and respiratory diseases on a weekly basis. Our data feeds into PHE's surveillance system and gives an early warning of epidemics or unusual patterns of disease. Our monitoring warns of seasonal events that place pressure on health services, such as the start of the flu season, and allow services to be planned accordingly.

Collecting samples of viruses and disease-causing organisms

RSC practices take samples from patients that are used to increase our understanding of disease and how to combat it:

- Flu virology swabs, taken within 7 days of onset, are used to establish which flu strain is dominant in the current season; and also to infer the strain likely to be dominant the following flu season, so that decisions can be made on how the flu vaccine can be improved in order to target these specific strains. (See p. 9.)

Identifying disparities

The richness of clinical data allows us to identify demographic factors around clinical presentations. For this report, we present the distribution of age, gender, ethnicity, deprivation and rural, urban or conurbation living for each condition, compared to the larger RSC population. The aim of this is to understand any disparities in clinical presentation, concerning key demographic characteristics. This should guide clinical practice in identifying at-risk groups, and provides a strong basis for public health interventions.

Helping to change clinical practice

The RCGP RSC relies on practices agreeing to share their anonymised patient data with us – and, in return, their aggregated data provides evidence for research that is used to improve clinical practice and patient outcomes. The Diabetes Real World Evidence (RWE) Centre, for instance, has explored adherence to and persistence with different classes of anti-diabetes medicines and the thresholds at which different clinicians implement injectable therapy in type 2 diabetes (T2DM)[4,5,6,7,8]. Currently, the work of the RWE centre is focused around demographic disparities in the care of people with T2DM, and targeted interventions around the risk of macrovascular complications.

---

[4] McGovern A, Hinton W, Tippu Z, Whyte M, de Lusignan S. Ethnic disparities in medication persistence in type 2 diabetes: Non-whites have reduced persistence [Abstract]. Diabetes (June 2016). 65 (Supplement 1): A23

[5] McGovern A, Hinton W, Munro NM, Whyte MB, de Lusignan S. Do persistence rates vary between dipeptidylpeptidase-4 inhibitors? [Abstract]. Diabetes (June 2016). 65 (Supplement 1): A576

[6] McGovern A, Hinton W, van Vlymen J, Munro N, Whyte M, de Lusignan S. Real world evidence on the prescribing trends in sodium glucose co-transporter 2 inhibitors in UK primary care [Abstract]. Diabetic Medicine (March 2016). 33 (Supplement 1): P165.

[7] Hinton W, McGovern A, van Vlymen J, Munro N, Whyte M, de Lusignan S. Real world evidence on the prescribing trends of glucagon-like peptide-1 agonists in UK primary care [Abstract]. Diabetic Medicine (March 2016). 33 (Supplement 1): P165.

[8] McGovern A, Hinton W, van Vlymen J, Munro N, Whyte M, de Lusignan S. Real world evidence on the disparities of prescribing of dipeptidyl peptidase-4 inhibitors in UK primary care [Abstract]. Diabetic Medicine (March 2016). 33 (Supplement 1): P183.

3. The RSC dataset

Our data extraction process and information governance

Data are extracted twice weekly from practice systems by Apollo Medical Systems, a well-established data extraction company, on behalf of the RCGP. Patients who have withheld consent for data sharing, for any reason, are excluded from our analyses. These patients are identified through an 'opt-out' code, which the automatic extraction process uses to exclude them.

Data are pseudonymised (this is a process that scrambles any strong identifiers such as name, NHS number, and date of birth) as close to source as possible. The pseudonymised data are held on secure servers at the RCGP RSC data and analytics hub in the Section of Clinical Medicine and Ageing at the University of Surrey. Both Apollo and the University of Surrey are fully compliant with NHS data governance rules.

The dataset used for the RSC Annual Report 2016/17

The dataset used in this report was extracted at the end of May 2017. It includes 1,835,211 patients from 174 practices. This includes all patients who were registered for at least one week during the reporting period, within a practice from the RCGP RSC network for which we received data. There is one exception to this: the dataset used to calculate influenza vaccine coverage. Swabbing results and vaccine effectiveness were defined differently for the specific purposes of the end of influenza season report.

For the week-by-week disease incidence graphs, we include episodes of disease in the numerator if the patient was validly registered with a practice within the RCGP RSC network at the time of the episode. The denominator includes all registered patients for that particular week.

Reporting period

This report covers the period 9th May 2016 to the 7th May 2017 (International Standards Organisation (ISO) Week 19 of 2016 to ISO Week 18 of 2017). It covers this period, rather than a calendar year, in order to ensure that one complete summer season and one complete winter season are included in our analysis.

4. Sample population

This section shows the demographic (age and gender) breakdown of our sample population, and how it compares with that of England as a whole. This report covers the period 9th May 2016 to the 15th May 2017(ISO Week 19 of 2016 to ISO Week 20 of 2017). This includes all patients who were registered for at least one week during the reporting period, within a practice from the RCGP RSC network for which we received data.

Age Sex Breakdown by NHS Region

NHS Region	Gender	<1yr	1-4yrs	5-14yrs	15-24yrs	25-44yrs	45-64yrs	65-74yrs	75-84yrs	85+yrs
South	F	2,321	13,051	32,223	41,014	76,442	75,521	31,655	20,035	12,219
	M	2,350	13,604	34,463	39,468	75,397	75,750	29,841	16,419	6,841
London	F	2,030	10,552	20,870	22,934	80,266	34,851	9,168	5,846	2,967
	M	2,158	10,733	21,696	19,921	74,065	37,123	8,119	4,484	1,738
Midlands And East	F	1,123	6,466	16,531	15,922	35,926	39,950	16,900	10,048	5,893
	M	1,230	6,693	17,389	16,586	35,622	40,125	15,885	8,518	3,326
North	F	2,116	11,629	29,234	44,628	73,383	70,796	29,479	18,425	10,120
	M	2,238	12,273	30,511	41,079	75,796	72,410	28,021	15,261	5,544
National	F	7,590	41,698	98,858	124,498	266,017	221,118	87,202	54,354	31,199
	M	7,976	43,303	104,059	117,054	260,880	225,408	81,866	44,682	17,449

We work very hard to ensure that the demographics of our sample population closely resemble the country as a whole. This correspondence can be seen in the age-sex profile below; the bars show the number of patients within our dataset, broken down by gender and age-band, and the lines show the distribution of the corresponding population of England as a whole. The age-sex profile shows that females aged 40-65 were particularly underrepresented this season. The 0-5 age group tends to be underrepresented in most seasons as general practice registration does not generally take place until a little while after birth. Both males and females aged between 20 and 30 were slightly overrepresented this season.

Age Sex Profile

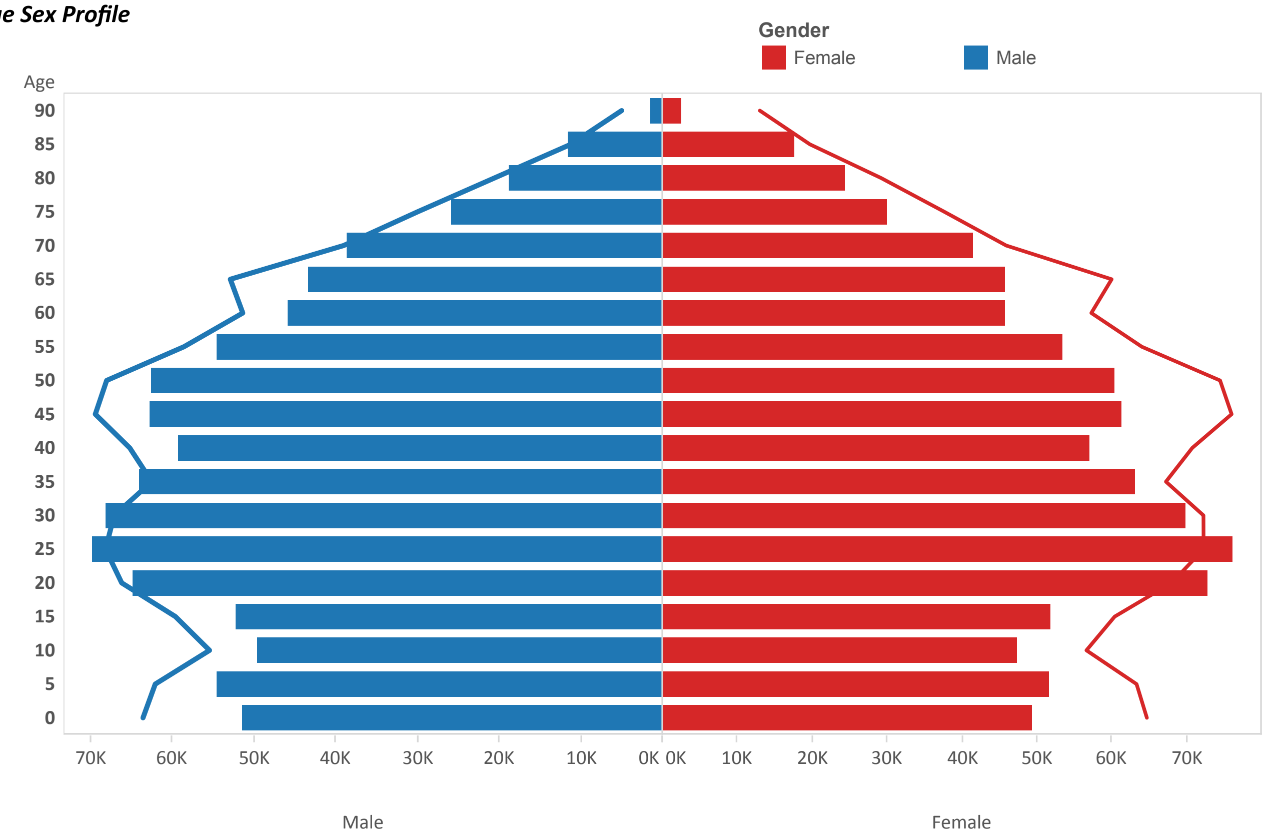

The trends of sum of MalePop, sum of MaleONS, sum of FemalePop and sum of FemaleONS for Age. Color shows details about Gender. The data is filtered on single year of Age, which keeps 91 of 119 members. The view is filtered on Age, which has multiple members selected.

As can be seen from the map below, practices within the Network are spread across England in order to reflect the distribution of the population as a whole. We vary our priorities for recruiting to try to ensure an even spread of practices. We largely recruit new practices through the RCGP Research Ready network.

Distribution of RSC practices among NHS regions in England

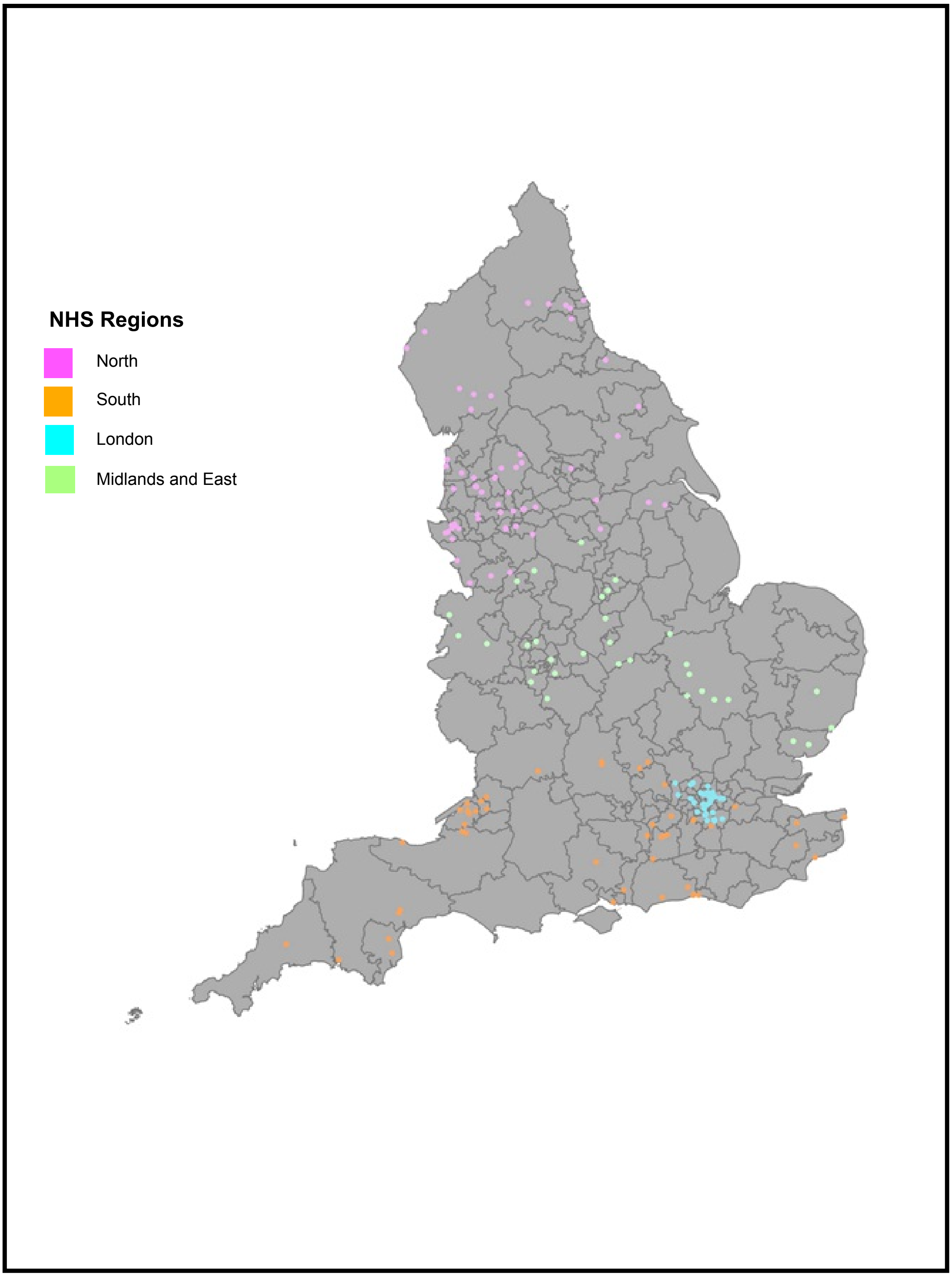

5. Influenza

Work on influenza is a central part of the RCGP RSC's activity. In addition to surveillance of weekly incidence of influenza-like illness, we conduct a programme of virological swabbing throughout the flu season and conduct analysis of vaccine uptake and effectiveness.

Swabbing programme

A total of 2,040 swabs were taken during the season. These are summarised in the table below, which shows positivity rates by age band. The most swabs were taken in the 25-44 and 45-64 age bands. There were fewer cases of flu B recorded this season (2016-17 n = 2; 2015-16 n = 203). On the other hands, the rates of H3 were higher this season (2016-17 n =159; 2015-16 n = 24).

Positivity rate by age band

Age Band	Total Samples	Positive for H3	Positive for flu B	Positive for any virus
**<1**	29 (1.50%)	1 (3.45%)	0 (0%)	6 (20.69%)
**01-4**	135 (7.00%)	4 (2.96%)	0 (0%)	48 (35.56%)
**05-14**	150 (7.78%)	14 (9.33%)	0 (0%)	13 (8.67%)
**15-24**	232 (12.03%)	22 (9.48%)	1 (0.43%)	15(6.47%)
**25-44**	515 (26.70%)	53 (10.29%)	0 (0%)	42 (8.16%)
**45-64**	553 (28.67%)	47 (8.50%)	0 (0%)	7878 (14.10%)
**65-74**	208 (10/78%)	10 (4.81%)	1 (0.48%)	28 (13.46%)
**75-84**	76 (3.94%)	5 (6.58%)	0 (0%)	11 (14.47%)
**85+**	31 (1.61%)	3 (9.68%)	0 (0%)	1 (3.23%)
**Total**	1929 (100%)	159 (8.24%)	2 (0.10%)	242 (12.55%)

The graph below summarises the season's virology coupled with all-age influenza-like illness incidence[9], and allows a comparison between this season and last season's incidence rates.

The number of swabs peaked in Week 51 and again in week 2. The results from the swabbing analysis showed that there were low levels of A(H1N1)[10] and influenza B[11] circulating this season. Influenza H3 was the predominant strain of flu this season. Influenza H3 rates slowly increased from week 41 and peaked at week 3.

[9] Incidence is the number of new cases per 100,000 population registered with RCGP RSC practices.
[10] H1N1 is a subtype of influenza A known to cause pandemics in humans.
[11] Influenza B infections are of one subtype, generally influenza B follows influenza A each winter season.

Influenza Swabbing Surveillance

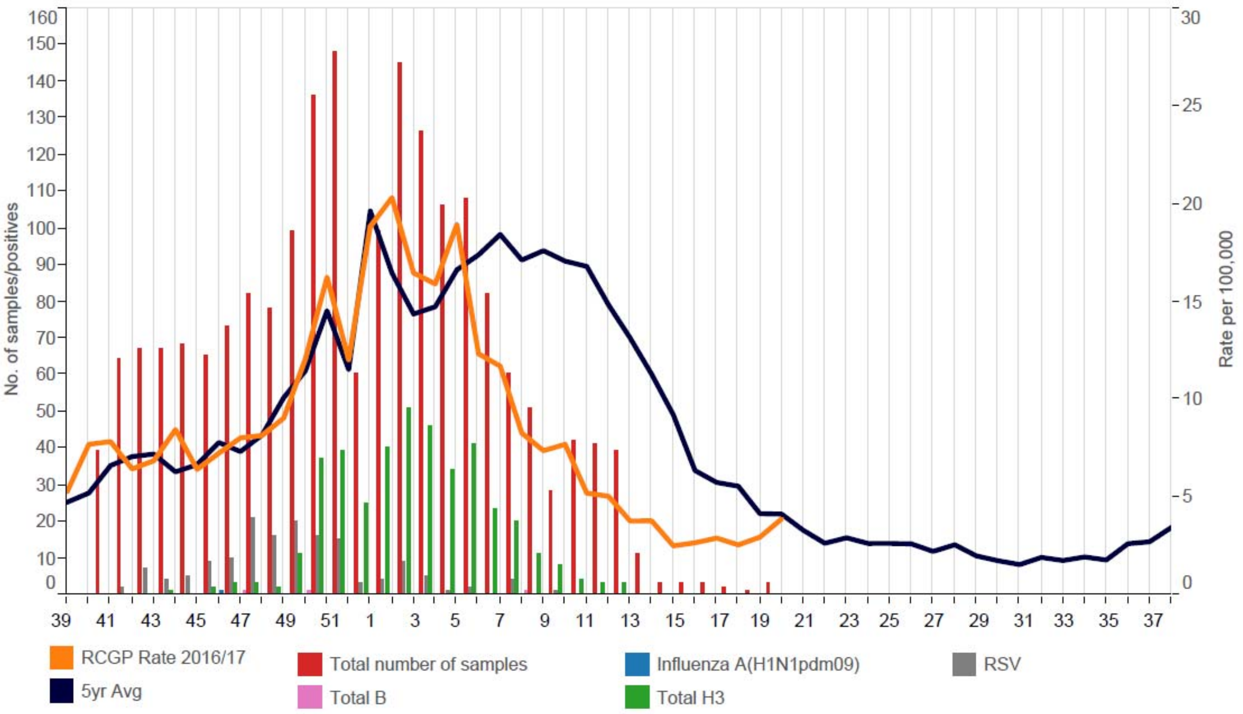

Vaccine uptake

The table below shows influenza vaccine coverage, as observed in our dataset. In addition to vaccine coverage across all patients, we have measured vaccine coverage among the following key populations, who were offered the influenza vaccination in 2016/17:

- Children aged two, three or four eligible for the Live Attenuated Influenza Vaccine (LAIV) at the general practice[12]. Additionally, we are including children in Year 1-2 (aged five to seven) who are eligible for LAIV vaccine at their school[13].
- Those aged 65 and over
- Patients aged under 65 in a clinical risk group (as defined below)

Our results show different vaccine coverage rates than those reported by Public Health England (PHE).[14] In line with PHE's reporting, uptake increases with age and is greatest among the older population.

[12] LAIV is a nasal spray flu vaccine. All LAIV is designed to protect against four different influenza viruses: Influenza A (H1N1), A (H3N2) and two influenza B viruses.
[13] This is a new change in the service specification for 2015/2016, and may not be recorded within the GP data, as the scheme operates in primary schools.
[14] Public Health England (2017) Seasonal influenza vaccine uptake in GP patients: winter season 2016 to 2017 https://assets.publishing.service.gov.uk/government/uploads/system/uploads/attachment_data/file/613452/Seasonal_influenza_vaccine_uptake_in_GP_patients_winter_season_2016_to_2017.pdf

Influenza vaccine coverage

Age Band	Total eligible population	Vaccine coverage
**All patients**	1,699,740	22%
**Children eligible for LAIV (children aged 2-7)**	119,937	36%
**Patients aged under 65 in risk groups**	161,864	42%
**65 and over**	296,802	71%

This analysis is based on a sample of practices for which we were able to extract data at the end of the flu season. Because the extract was taken earlier in the year than that used elsewhere in this Annual Report, our vaccine coverage figures are based on a smaller sample than the analysis in the rest of this report.

The risk factors included in this table are:

- Asplenia or dysfunction of the spleen
- Asthma
- Chronic heart disease (CHD)
- Chronic kidney disease (CKD)
- Chronic liver disease
- Chronic neurological disease (including stroke/transient ischaemic attack, cerebral palsy and multiple sclerosis)
- Chronic respiratory conditions
- Diabetes
- Immuno-suppression

It should also be noted that vaccinations administered in settings other than general practices, such as school or pharmacies, are likely to be inconsistently recorded in the general practice record[15]. Therefore, the vaccine coverage data given here is likely to be a slight underestimate of the true rate.

Vaccine effectiveness

RCGP RSC data was one of the primary data sources for PHE's evaluation of influenza vaccine effectiveness (VE) during the 2016/17 season. The text below is taken from the published evaluations.

'The VE point estimate for inactivated vaccine (IIV) in 18–64-year-olds for influenza A(H3N2) was 36.6% (95% CI: 10.4 to 55.1), however, there was no significant effectiveness against influenza A or specifically A(H3N2) in those aged 65 years and above (aVE: -68.4%; 95% CI: -248.9 to 18.7)…The aVE point estimate for LAIV4 against influenza A(H3N2) in children 2–17 years old was 57% (95% CI: 7.7 to 80), and non-significantly lower for IIV at 24.9% (95% CI: -296.1 to 85.8)… [For influenza B] in adults 18–64 years of age, the aVE point estimate was 52.1% (95% CI: -20 to 80.9), whereas in those 65 years of age and above, the aVE point estimate was not statistically significant with very wide 95% Cis…The aVE point estimate for LAIV4 against influenza B was 78.6% (95% CI: -86 to 97.5).'[16]

[15] de Lusignan S. Flu vaccination by pharmacists leads to suboptimal medical records. BMJ 2017; 359;J5084. https://www.bmj.com/content/359/bmj.j5084

[16] Pebody R, Warburton F, Ellis J, Andrews N, Potts A, Cottrell S, Reynolds A, Gunson R, Thompson C, Galiano M, Robertson C, Gallagher N, Sinnathamby M, Yonova I, Correa A, Moore C, Muhammed S, de Lusignan S,

6. Disparities

We explored disparities by condition, and in comparison to the overall RSC population. The network has been previously shown to be representative of the English population[17]. We examined whether people who presented with a given condition in the previous year had different demographic characteristics (age, gender, ethnicity, deprivation, and rural, urban or conurbation living) than the general population. We present the distribution of these variables, with 95% confidence intervals, for each individual condition in the appendix graphs (B & C). We also include these variables for all conditions in the graphs below.

Age

We obtained the age for each registered patient on 1st May 2016. We found the following patterns:

- The median age for rubella, measles, chickenpox, and strep throat was below that of the population median.
- The median age for herpes zoster, pleurisy, pneumonia and bullous dermatoses was above that of the population median.
- The median age for meningitis/encephalitis, asthma and skin symptoms was similar to that of the population median.
- The median age for influenza-like illness was slightly higher than the population median.

Gender

Gender is recorded by the general practice on each patient's electronic record, when they first register in a practice. We found the following patterns:

- Overall more women presented with conditions than men, reflecting their higher propensity to consult.
- Women presented more often than men with urinary tract infections, herpes simplex and sinusitis.
- Men presented more often than women with viral hepatitis and measles.
- Almost 60% of influenza-like illness consultations were for women.

Ethnicity

Ethnicity is recorded on a proportion of patients. We have developed an algorithm, which incorporates languages spoken and other recorded fields, to generate the most probable ethnicity of a patient[18].

McMenamin J, Zambon M End-of-season influenza vaccine effectiveness in adults and children, Euro Surveill. 2017Nov 22(44):pii=17-00306 https://doi.org/10.2807/1560-7917.ES.2017.22.44.17-00306

[17] Correa A, Hinton W, McGovern A, van Vlymen J, Yonova I, Jones S, de Lusignan S. Royal College of General Practitioners Research and Surveillance Centre (RCGP RSC) sentinel network: a cohort profile. BMJ Open. 2016 Apr 20;6(4):e011092. doi: 10.1136/bmjopen-2016-011092. PubMed PMID: 27098827; PubMed Central PMCID: PMC4838708.

[18] Tippu Z, Liyanage H, Correa A, Burleigh D, McGovern A, Jones S, de Lusignan S. (2016) Ontologies to improve the identification of ethnicity in people with type 2 diabetes [Poster]. Diabetes UK Professional Conference. Glasgow, UK. March 2-4th, 2016.

The ethnic groups used are those in the 2011 census (Asian, Black, Mixed, Other, White). We found the following patterns:

- More non-white patients presented with viral hepatitis, allergic rhinitis and musculoskeletal symptoms compared to the overall population.
- More white patients presented with rubella, bullous dermatoses and infectious mononucleosis compared to the overall population.
- There were slightly more influenza-like illness consultations from non-white people, compared to the overall population.

Deprivation

We determined deprivation using the Index of Multiple Deprivation (IMD)[19], which assigns a score to each Lower Super Output Area (LSOA) in England[20]. When we extract our data, each patient's postcode is converted to LSOA. A lower score represents a less deprived area. We found the following patterns:

- Patients presenting with infectious intestinal disease, scabies and viral hepatitis had a higher median IMD score (more deprived) than the overall population.
- Patients presenting with rubella, infectious mononucleosis and herpes zoster had a lower median IMD score (less deprived) than the overall population.
- Patients presenting with influenza-like illness had a slightly higher median IMD score (more deprived) than the overall population.

Urban, Rural or Conurbation Living

We banded together the Office of National Statistics (ONS) classifications conurbation, city or town into urban living, and rural livingl[21]. These are based on population density. We determined the patient's classification at individual level mapping the first four elements of the post code into conurbation, urban, or rural. These were based on the ONS lower super output area (LSOA), which has a mean size in England and Wales of 1,640, with population sizes ranging from 820 in South Cambridgeshire to 8,250 in Oxford[22].

- Patients living in urban areas were more likely to present with scabies, musculoskeletal symptoms and infectious intestinal diseases.
- Patients living in rural areas were more likely to present with rubella, whooping cough and impetigo.

[19] Department for Communities and Local Government (2015). The English Indices of Deprivation 2015. Available at: https://www.gov.uk/government/statistics/english-indices-of-deprivation-2015

[20] Office of National Statistics (2011). Postcodes (Enumeration) to output areas to lower layer SOA to middle layer SOA to local authority districts E+W lookup. Available at: https://geoportal.statistics.gov.uk/Docs/Lookups/Postcodes_(Enumeration)_(2011)_to_output_areas_(2011)_to_lower_layer_SOA_(2011)_to_middle_layer_SOA_(2011)_to_local_authority_districts_(2011)_E+W_lookup.zip

[21] ONS Guide to applying the Rural Urban Classification to data https://assets.publishing.service.gov.uk/government/uploads/system/uploads/attachment_data/file/539241/Guide_to_applying_the_rural_urban_classification_to_data.pdf

[22] English LSOA Rural/Urban Classification, 2011 https://borders.ukdataservice.ac.uk/easy_download_data.html?data=England_lsoa_ru_classn_2011

Disparity Graphs for all Conditions

Median Age

Disease Name	Sample Size
Rubella	4
Measles	16
Chickenpox	4,486
Strep throat / peritonsillar abscess	1,237
Acute Otitis Media	15,732
Impetigo	4,337
Common Cold	59,382
Infectious Mononucleosis	341
Mumps	111
Upper Respiratory Tract Infections	111,647
Laryngitis / Tracheitis	2,915
Intestinal Infections	6,976
Scabies	1,103
Conjunctivitis	18,803
Allergic Rhinitis	9,813
Non-infective Enteritis / Colitis	6,360
Herpes Simplex	3,516
Respiratory disorders	175,371
Meningitis / Encephalitis	109
Asthma	10,406
Skin symptoms	28,751
Pertussis	172
Influenza-like illness	4,954
Viral Hepatitis	217
Sinusitis	13,930
Skin / subcutaneous infections	37,740
Musculoskeletal symptoms	3,667
Respiratory / chest symptoms	14,780
Bronchitis	46,718
Peripheral Neuropathy	6,157
Lower Respiratory Tract Infections	48,052
Urinary Tract Infections	17,996
Herpes Zoster	4,921
Pleurisy	678
Pneumonia / Pneumonitis	1,034
Bullous Dermatoses	114

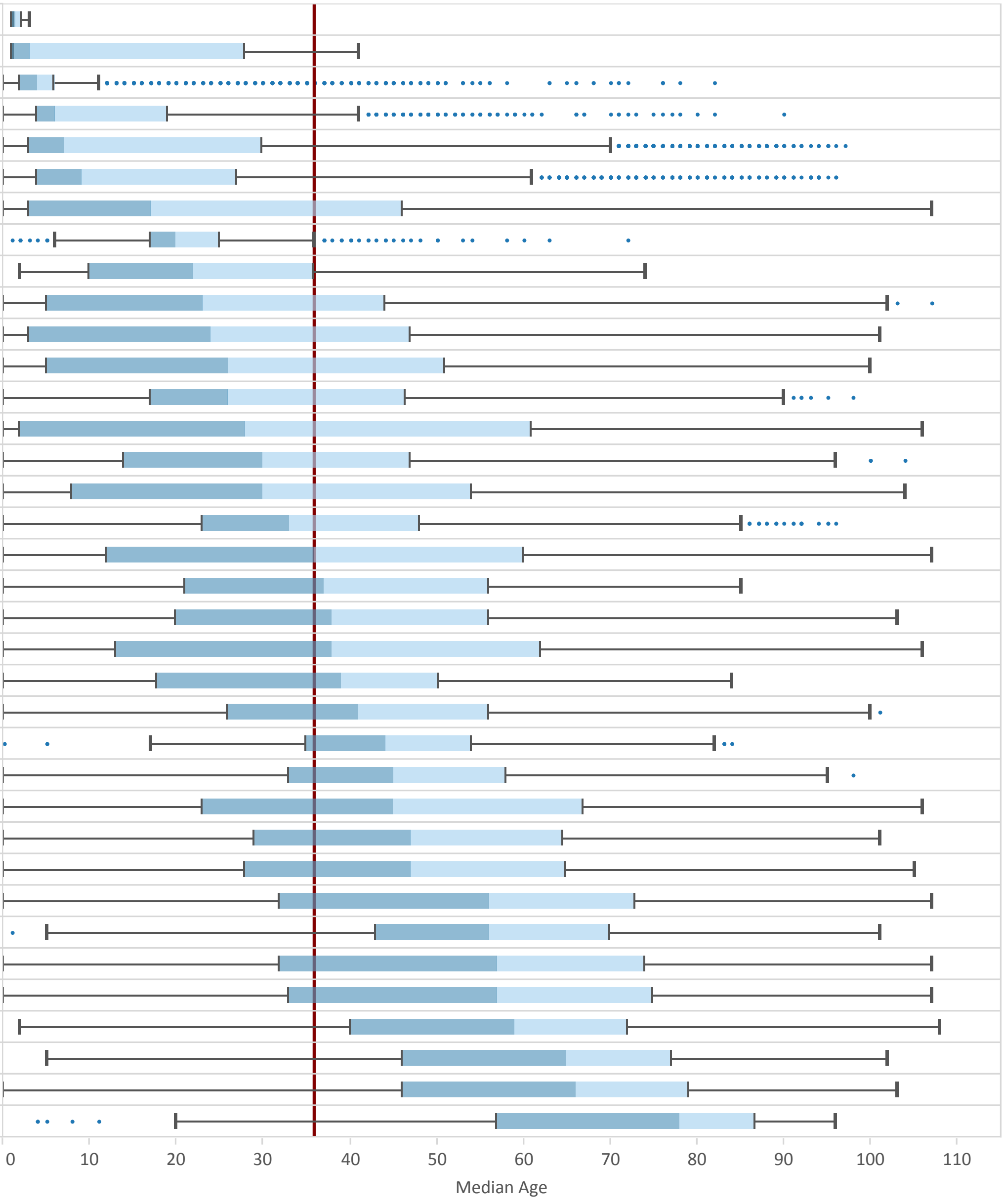

Median Age for each Sample Size broken down by Disease Name. Color shows details about Disease Name. The view is filtered on Disease Name, which excludes RSC.

Gender

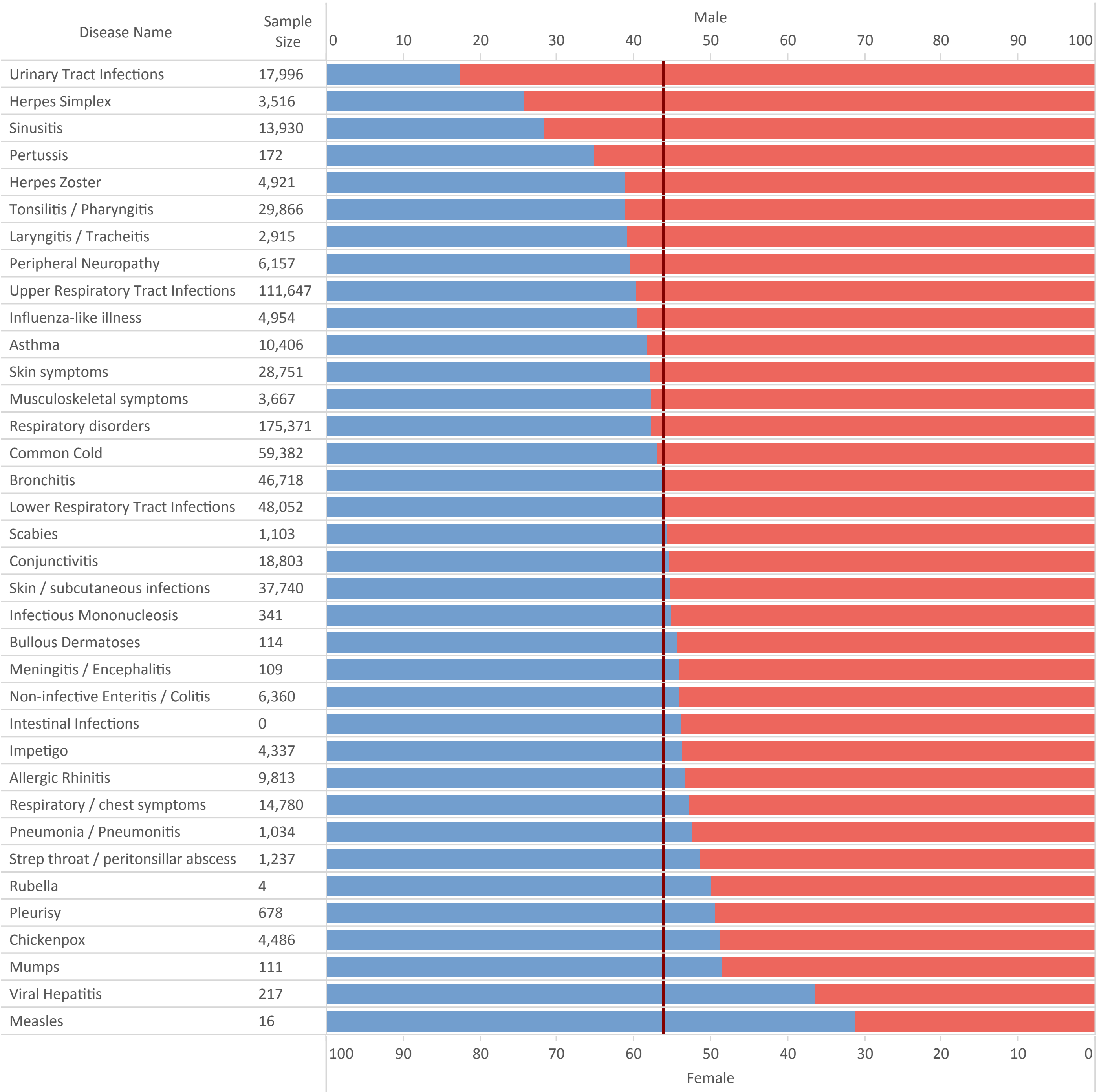

Female and Male for each sum of Sample Size broken down by Disease Name. Color shows details about Female and Male. The view is filtered on Disease Name, which keeps 36 of 38 members.

Measure Names

- Female
- Male

Ethnicity

Disease Name	Sample Size
Viral Hepatitis	173
Allergic Rhinitis	7,662
Musculoskeletal symptoms	2,908
Chickenpox	3,016
Intestinal Infections	5,182
Common Cold	42,442
Influenza-like illness	3,874
Non-infective Enteritis / Colitis	4,752
Upper Respiratory Tract Infections	81,048
Scabies	830
Skin symptoms	21,543
Conjunctivitis	13,230
Tonsilitis / Pharyngitis	21,694
Respiratory disorders	131,717
Asthma	8,281
Respiratory / chest symptoms	11,214
Acute Otitis Media	10,844
Strep throat / peritonsillar abscess	902
Herpes Simplex	2,660
Mumps	70
Skin / subcutaneous infections	28,493
Meningitis / Encephalitis	78
Peripheral Neuropathy	4,866
Sinusitis	11,012
Pneumonia / Pneumonitis	793
Bronchitis	35,788
Lower Respiratory Tract Infections	36,800
Laryngitis / Tracheitis	2,099
Urinary Tract Infections	14,188
Impetigo	3,015
Measles	11
Pertussis	134
Pleurisy	519
Herpes Zoster	3,797
Infectious Mononucleosis	227
Bullous Dermatoses	91
Rubella	3

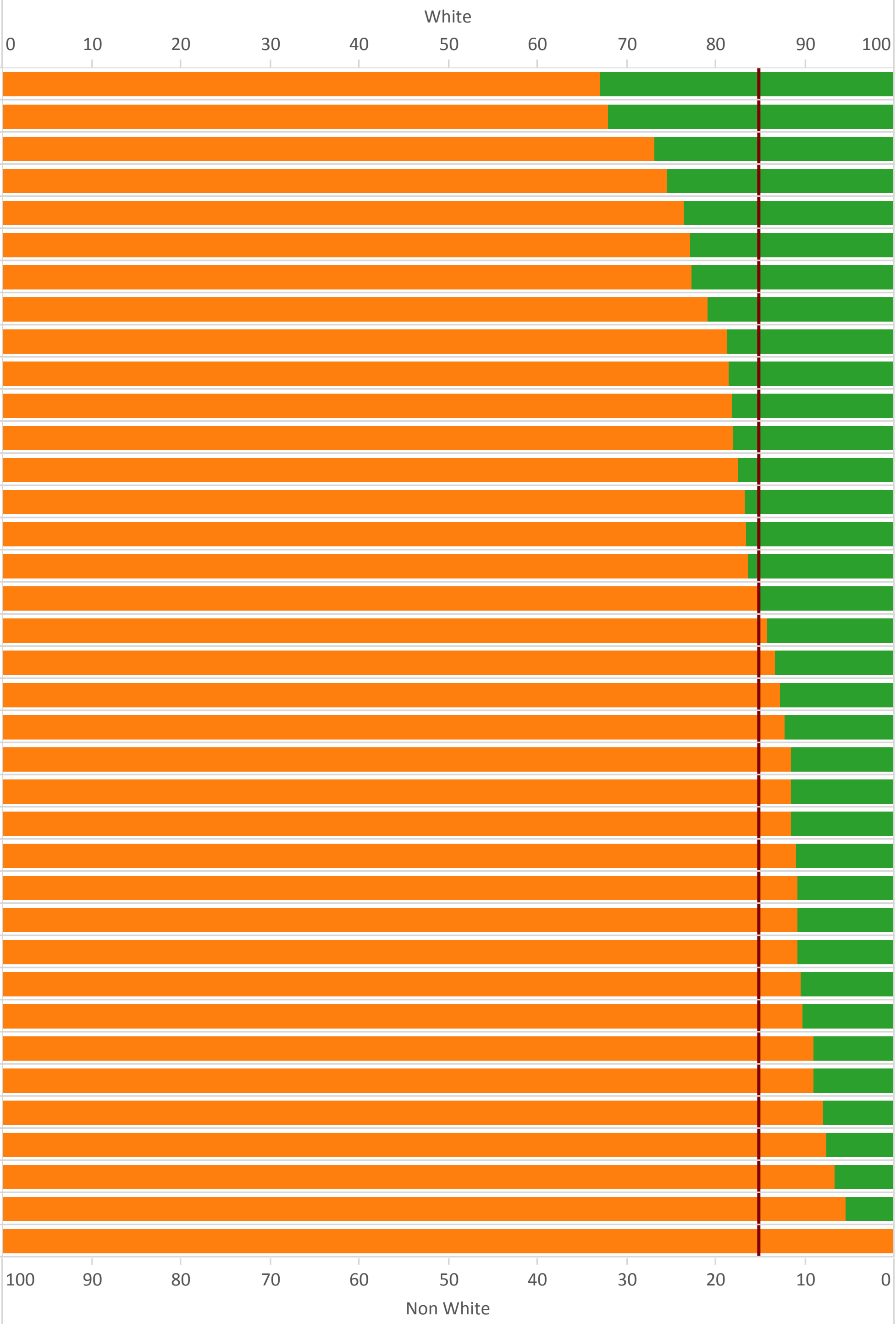

Non White and White for each sum of Sample Size broken down by Disease Name. Color shows details about Non White and White. The view is filtered on Disease Name, which keeps 37 of 38 members.

Measure Names

- Non White
- White

Median IMD

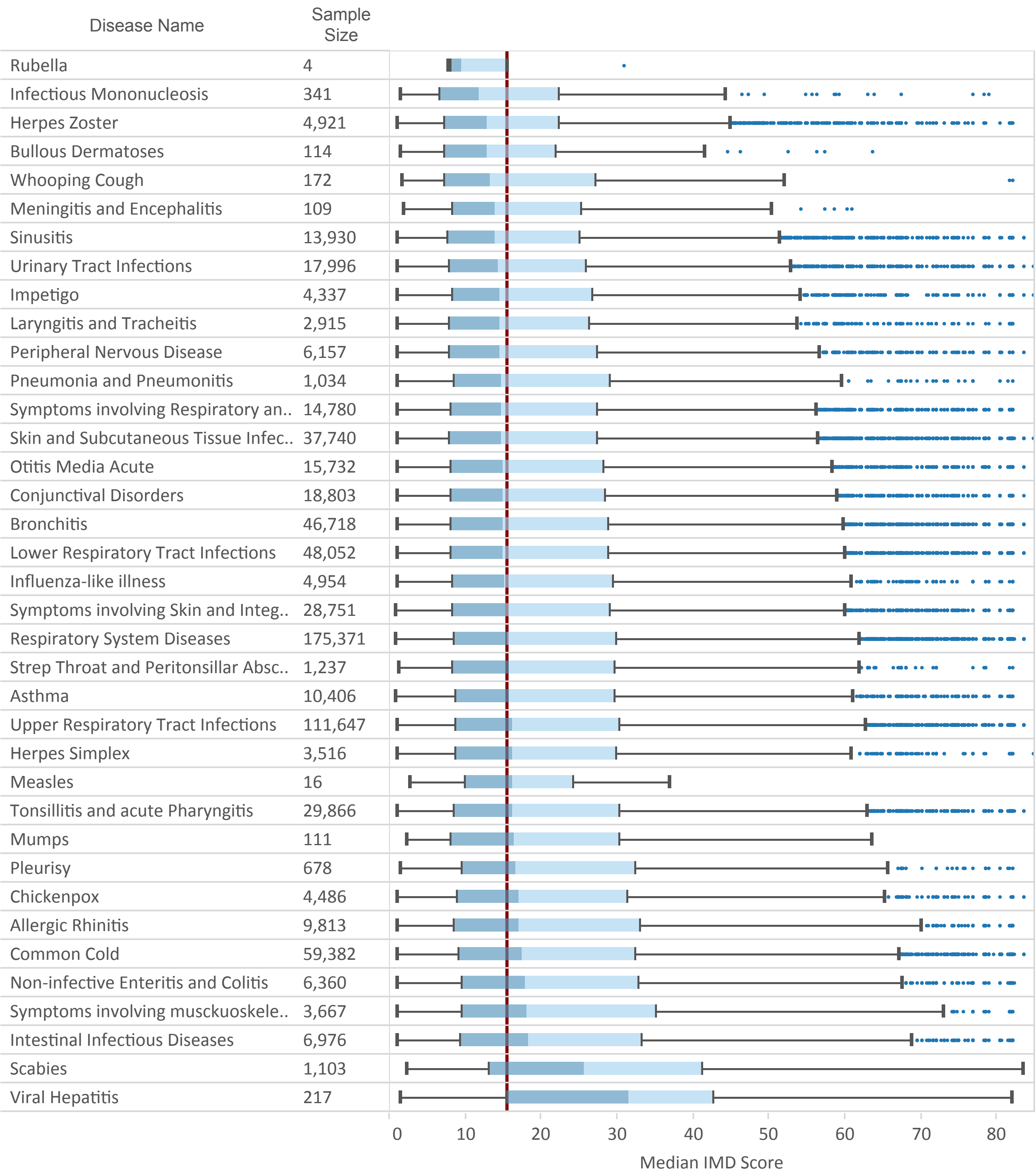

Median IMD Score for each Sample Size broken down by Disease Name. Details are shown for Disease Name. The view is filtered on Disease Name, which excludes RSC. A lower score represents a less deprived area.

Living environment

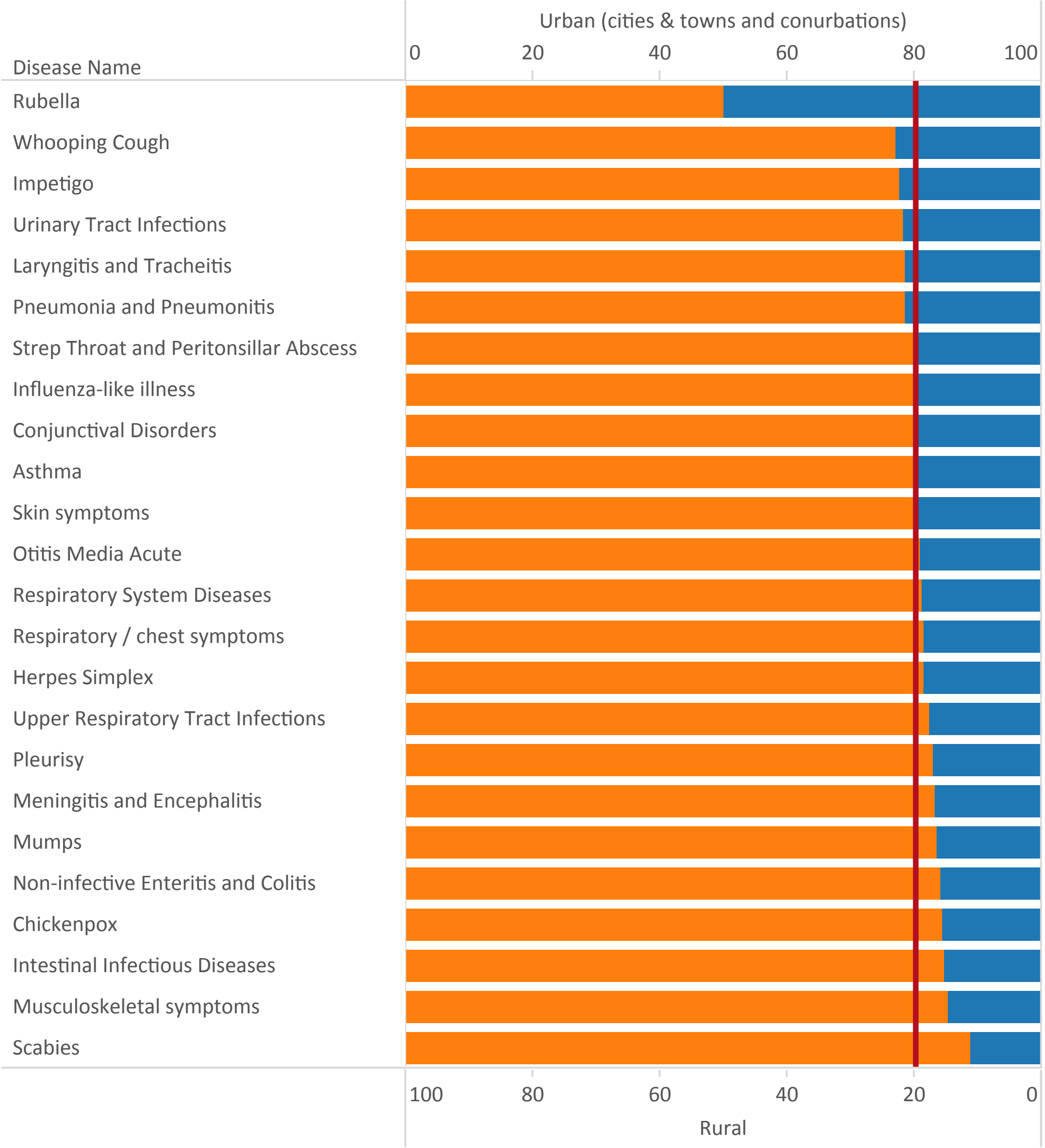

Rural and Urban (cities & towns and conurbations) for each Disease Name. Color shows details about Rural and Urban. The view is filtered on sum of Urban, which keeps all values.

7. Disease incidence

We monitor the incidence of 37 conditions in our weekly disease surveillance reports, both nationally and for the four English NHS regions, in comparison with the 10-year average. Graphs showing weekly incidence of all monitored diseases are included in Appendix A.

These graphs are presented in the same format as in the RCGP RSC weekly surveillance report[23]. The data presented is an amalgamation of the data from the weekly surveillance reports. The five year average presented in the graphs is calculated using data from the winter reports.

Like the weekly surveillance report, the data are presented in this report by disease chapter. The key points for each chapter are:

- **Water and food-borne disorders.** National rates of intestinal infectious diseases for all ages were below seasonal averages, especially in weeks 48-18. However, rates in London and the North were higher than the 5 year average. For children aged 0-4, national rates of intestinal infections were above average in weeks 38-48. National rates of non-infective enteritis/colitis were below seasonal average. Viral hepatitis was mainly at seasonal average apart from peaks at weeks 2-4, 8 and 17. Rates were above average for both non-infective enteritis/colitis and viral hepatitis in London.
- **Environmentally-sensitive disorders.** Rates of asthma were above the national average, especially in weeks 40-6. There were large peaks of asthma presentations in weeks 48 and 1-7 in the Midlands and the East. Conjunctivitis and Respiratory/Chest symptoms were at below seasonal levels. Allergic rhinitis showed the usual peak in rates around May, however the peak was 2 weeks earlier this season at week 23, compared to the 5-year average of 25-26.
- **Respiratory infections.** Rates of influenza-like illness was at seasonal average up until weeks 2-5 when there was an above average increase, followed by weeks 6-18 where rates were below average. Pertussis rates were at seasonal averages with peaks at weeks 38-41 and 2-3. Particularly high spikes were shown in the Midlands and the East. A similar pattern was found with infectious mononucleosis, with rates mainly at national average with a few peaks in weeks 30-48. Again, particularly high spikes were shown in the Midlands and the East. Rates of all other respiratory infections were at or below seasonal averages.
- **Vaccine-sensitive disorders.** Measles rates were similar to seasonal average. The rates were above average in Weeks 33-36, with higher rates shown in the Midlands and East and London. Mumps rates were mainly at seasonal levels. There were peaks in weeks 46, 52 8-10. These peaks were found in the South and Midlands and the East. Rubella rates were below season average apart from peaks in weeks 26, 28, 1 and 12.
- **Skin contagions.** Rates of chickenpox were below national average. Skin symptoms mainly followed seasonal averages with a peak at weeks 23-24 and 28-30. All other skin contagions were at or below seasonal averages.
- **Disorders affecting the nervous system.** Peripheral neuropathy and musculoskeletal symptoms were below seasonal averages. Meningitis and encephalitis rates were at seasonal averages.

[23] RCGP RSC Weekly Report. URL: http://www.rcgp.org.uk/clinical-and-research/our-programmes/research-and-surveillance-centre.aspx

- **Genitourinary system disorders.** Presentations of urinary tract infections were at seasonal average.
- **Scarlatina.** Scarlett fever is usually grouped with strep throat in the weekly report, however recently we have seen some increase in scarlatina rates. Rates were above seasonal average at different points across the season, with the largest peaks shown in weeks 23-24, 27-29, 46-52, 4-5, 7-9 and 14-15.

As noted above, considerable regional variation can be seen for some diseases. For environmentally-sensitive disorders such as allergic rhinitis, this variation may reflect differing weather patterns across the country. We have produced an accompanying paper exploring disease incidence in conurbation, urban and rural areas[24]. The following conditions were explored in this paper:

- **Allergic rhinitis:** Across all age groups, 1.08% of those living in conurbations presented with allergic rhinitis compared to 0.81% of those living in urban areas, and 0.71% of those living in rural areas.
- **Acute gastroenteritis:** Acute gastroenteritis was most common in conurbations (0.63%) followed by urban (0.55%) and rural areas (0.48%).
- **Asthma:** Patients living in rural areas were most likely to present with asthma (3.22% of all patients living in rural areas). This was followed by 3.13% of patients who lived in towns and cities. Patients who lived in conurbations were the least likely to present with asthma (2.5% of this population).
- **Lower respiratory tract infection (LRTI):** Across all age groups, 3.23% of people living in conurbations presented with LRTI. This is in comparison to 3.72% of those living in urban areas and 3.65% of people living in rural areas.
- **Upper respiratory tract infection (URTI):** Those living in conurbations were most likely to present with URTI (9.61%). This was followed by those living in urban (8.9%) and rural areas (7.99%).
- **Urinary Tract Infection (UTI):** Patients living in rural areas were more likely to present with a UTI (1.64%) compared to those living in urban areas (1.51%) and conurbations (1.42%).

[24] de Lusignan S, McGee C, Webb R, Pebody R, Yonova I, Smith G, Byford R, Pathirannehelage S, Elliot A J, Hriskova M, Ferreira F, Rafi I, Joy M. Rural, urban and city living are determinants of allergic and infectious disease: Royal College of General Practitioners (RCGP) Research and Surveillance Centre (RSC) Annual Report 2016-2017**.** In prep.

8. Episode typing – key part of data quality

Recording episode type is the only way that we can differentiate incident (first and new) from prevalent cases (ongoing care/reviews). RCGP RSC practices get constant feedback and reminders about the importance of recording whether a clinical consultation is the first time a patient presents the condition or whether it is a follow up appointment. Different conditions will have varying ratios of incident (first or new) to prevalent cases (reviews). We expect:

- Only a small proportion of people with influenza or influenza-like illness will be seen more than once by their general practitioner. We therefore expect there to be very few follow-ups.
- A higher proportion of people with acute bronchitis and/or bronchiolitis will be followed up.
- Most people with asthma (a long term condition) will be seen for follow-up/review. In asthma we anticipate there will be many more reviews than new cases.

The chart below shows the mean rate of First and New episodes against all episodes for the seven key conditions for which we provide monthly feedback to practices:

- Allergic rhinitis
- Asthma
- Bronchitis
- Infectious intestinal diseases
- Influenza-like illness
- Urinary tract infections

Episode typing by disease

Disease	First/New episode (n)	Ongoing (n)	Total (n)
**Allergic rhinitis**	11252	2504	13756
**Asthma**	12490	58576	71066
**Bronchitis**	55841	16700	72541
**Influenza-like illness**	5374	1136	6510
**Intestinal infectious diseases**	8064	1502	9566
**Urinary tract infections**	24541	10133	34674
**Grand Total**	**117562**	**90551**	**208113**

Patients mainly go to their general practitioner once for allergic rhinitis, bronchitis, influenza-like illness, intestinal infectious diseases and urinary tract infections. On the other hand, most appointments for asthma are follow-ups. The percentages are very similar to last season.

Graph showing percentage of appointment for first/new disease or ongoing

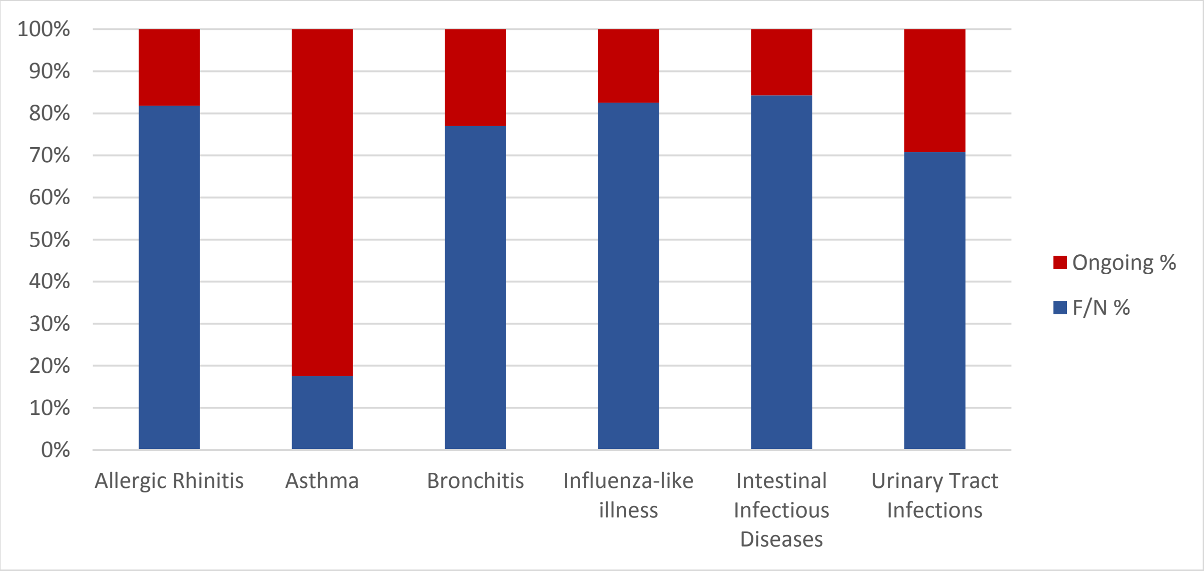

9. Current Projects

The RSC has been involved in the following projects during 2016/17.

European Projects

- **I-MOVE+:** RCGP RSC works in collaboration with leading European Public Health Institutes and Universities. It aims at measuring and comparing the effectiveness (VE) and impact (VI) of influenza and Pneumococcal vaccines and vaccination strategies in the elderly population in Europe. The goal is to develop a sustainable platform of primary care practices, hospitals and laboratory networks that share validated methods to evaluate post marketing vaccine performances.
- **ADVANCE:** The RSC is involved in The Accelerated Development of Vaccine benefit-risk Collaboration in Europe (ADVANCE) project funded by the Innovative Medicines Initiative (IMI). The project is focused on more rapidly generating best evidence on vaccine benefits and risks. It aims to establish a blueprint for a validated and tested best practice framework that could rapidly provide robust data to support accelerated decision making.

Other Projects

- **The live-attenuated influenza vaccination (LAIV) project:** The RSC is working with Public Health England and the University of Surrey on the pilot of a universal childhood (LAIV) programme. The UK initiated a universal childhood immunisation programme with a newly licensed intransally-administered trivalent LAIV in the 2013/14 influenza season.
- **Flu virology scheme:** Just over 70 of the RSC practices provided virology specimens this season. These practices provide nasal and throat specimens during the flu season (between the International Standards Organisation (ISO) week 40 and ISO week 20) each year from patients clinically suspected of having flu. This helps to establish which strain of the flu is dominant in the current season and is likely to be dominant the following flu season and how the flu vaccine can be improved in order to target these specific strains.
- **Diabetes Real World Evidence Centre:** The RSC is providing data, subject to ethical approval for studies looking at adherence to different types of diabetes medicines and the thresholds at which different clinicians implement injectable therapy in Type 2 diabetes.

10. RCGP Publications 2016/17

From oldest to newest:

Pebody R, Warburton F, Ellis J, Andrews N, Potts A, Cottrell S, Johnston J, Reynolds A, Gunson R, Thompson C, Galiano M, Robertson C, Mullett D, Gallagher N, Sinnathamby M, Yonova I, Moore C, McMenamin J, de Lusignan S, Zambon M. Effectiveness of seasonal influenza vaccine in preventing laboratory-confirmed influenza in primary care in the United Kingdom: 2015/16 mid-season results. Euro Surveill. 2016;21(13). doi: 10.2807/1560-7917.ES.2016.21.13.30179.

Correa A, Hinton W, McGovern A, van Vlymen J, Yonova I, Jones S, de Lusignan S. Royal College of General Practitioners Research and Surveillance Centre (RCGP RSC) sentinel network: a cohort profile. BMJ Open. 2016 Apr 20;6(4):e011092. doi: 10.1136/bmjopen-2016-011092.

Pebody R, Warburton F, Ellis J, Andrews N, Potts A, Cottrell S, Johnston J, Reynolds A, Gunson R, Thompson C, Galiano M, Robertson C, Byford R, Gallagher N, Sinnathamby M, Yonova I, Pathirannehelage S, Donati M, Moore C, de Lusignan S, McMenamin J, Zambon M. Effectiveness of seasonal influenza vaccine for adults and children in preventing laboratory-confirmed influenza in primary care in the United Kingdom: 2015/16 end-of-season results. Euro Surveill. 2016 Sep 22;21(38). doi: 10.2807/1560-7917.ES.2016.21.38.30348.

de Lusignan S, Correa A, Pathirannehelage S, Byford R, Yonova I, Elliot AJ, Lamagni T, Amirthalingam G, Pebody R, Smith G, Jones S, Rafi I. RCGP Research and Surveillance Centre Annual Report 2014-2015: disparities in presentations to primary care. Br J Gen Pract. 2017 Jan;67(654):e29-e40. doi: 10.3399/bjgp16X688573

11. Participating practices

This list includes all practices who were members of the RSC network at any point during the period covered by this report. For technical reasons, we were unable to extract data from all of the practices below for use in this report; therefore, the report is based on 174 of these practices. However, we would like to thank all practices for their participation in the work of the RSC.

Practice Name	County
**Aberfeldy Practice**	Greater London
**Adderlane Surgery**	Northumberland
**Adelaide Medical Centre**	Greater London
**Albert Road And Britannia Village Surgery**	Greater London
**Alcester Health Centre**	Warwickshire
**Alconbury & Brampton Surgeries**	Cambridgeshire
**Aldershot Health Centre**	Hampshire
**Ampthill Square Medical Centre**	Greater London
**Ash Tree House Surgery**	Lancashire
**Aspartia Medical Group**	Cumbria
**Avisford Medical Group**	West Sussex
**Axbridge & Wedmore M.P.**	Somerset
**Aylestone Surgery (Sahdev)**	Leicestershire
**Bangor Street Health Centre**	Lancashire
**Barlow Medical Centre**	Manchester
**Barnoldswick Medical Centre**	Lancashire
**Barrington Medical Centre**	Manchester
**Block Lane Surgery**	Manchester
**Bloxwich Medical Practice**	West Midlands
**Bridge End Surgery**	Durham
**Brigstock Medical Centre**	Greater London
**Brownlow Health (Brownlow Group Practice)**	Merseyside
**Burn Brae Medical Group**	Northumberland
**Cator Medical Centre**	Greater London
**Cheddar Medical Centre**	Somerset
**Cherry Hinton Surgery**	Cambridgeshire
**City Road Medical Centre**	Greater London
**Clevedon Riverside Group**	Somerset
**Cleveleys Group Practice**	Lancashire
**Corner Place Practice**	Devon
**Cotterils Lane Surgery**	West Midlands
**Creffield Medical Centre**	Essex
**Debenham Group Practice**	Suffolk
**Devon Square Surgery**	Devon
**Dr Bm Thomas Practice**	Greater London
**Dr C E Noren & Partners**	West Sussex
**Dr D.J Lawrence And Partners**	Kent

**Dr Gj Penrice**	Tyne and Wear
**Dr Ij Moodies Practice**	Lancashire
**Dr Maclennan & Partners**	Oxfordshire
**Dr Parker & Partners**	Cambridgeshire
**Dr Scriven, Lee, Hopkins & Sissons (Leek Health Centre)**	Staffordshire
**Dr Slade & Partners**	Somerset
**Dr Steiner & Partners**	Essex
**Dr. Lone & Partners**	North Yorkshire
**Dronfield Medical Centre**	Derbyshire
**Dr'S Mackinnon Chande & Chappell**	Manchester
**East Park Medical Centre**	West Yorkshire
**Eastham Group Practice**	Merseyside
**Eaton Socon Health Centre**	Cambridgeshire
**Edith Cavell Surgery**	Greater London
**Ellenbrook Medical Centre**	Manchester
**Elm House Surgery**	Greater London
**Farnborough Bank House Surgery**	Greater London
**Fieldway Medical Centre**	Greater London
**Frankley Health Centre**	West Midlands
**Garswood Surgery**	Merseyside
**Glenpark Surgery**	Tyne and Wear
**Greenway Community Practice**	Bristol
**Guildowns Group Practice**	Surrey
**Harbour View Healthcare**	West Sussex
**Haslemere Health Centre**	Surrey
**Haven Health Centre**	Suffolk
**Haydock Medical Centre**	Merseyside
**Headley Drive Surgery**	Greater London
**Heatherside Surgery**	Surrey
**Husbands Bosworth Surgery**	Leicestershire
**Immeary Street Surgery**	Tyne and Wear
**James Street Group Practice**	Cumbria
**Killick Street Health Centre**	Greater London
**Kiltearn Medical Centre**	Cheshire
**Kings Cross Road Surgery**	Greater London
**Knockin Medical Centre**	Shropshire
**Lache Health Centre**	Cheshire
**Laurel Bank Surgery**	West Yorkshire
**Laurel Bank Surgery**	Cheshire
**Lawrence House Surgery**	Greater London
**M84037**	West Midlands
**Market Harbororugh Med. Centre**	Leicestershire
**Mendip Vale Medical Practice**	Somerset
**Merepark Medical Park**	Cheshire

**Mill Hill Surgery**	Greater London
**Millway Medical Practice**	Greater London
**Mitchinson Road Surgery**	Greater London
**Much Wenlock Practice**	Shropshire
**Myhealth**	North Yorkshire
**New Hayesbank Surgery**	Kent
**New Inn Surgery**	Surrey
**New Road Surgery Bromsgrove**	Worcestershire
**Newton Place Surgery**	Kent
**Nightingale Valley Pract**	Bristol
**North Road West Medical Centre**	Devon
**Northbourne Medical Centre**	West Sussex
**Oak Lodge Medical Centre**	Greater London
**Oak Vale Medical Centre**	Merseyside
**Oaklands Health Centre**	Kent
**Palacci & Partners**	Greater London
**Papworth Surgery**	Cambridgeshire
**Parkside Group Practice**	Greater London
**Parkway Health Centre**	Greater London
**Pendle View Medical Centre**	Lancashire
**Phoenix Surgery**	Gloucestershire
**Pickering Medical Practice**	North Yorkshire
**Pinfold Medical Centre**	Leicestershire
**Pontesbury Medical Practice**	Shropshire
**Portishead Medical Group**	Somerset
**Portslade Health Centre**	East Sussex
**Priory Medical Centre**	Merseyside
**Queens Road Surgery**	West Yorkshire
**Regent House Surgery**	Lancashire
**Richmond Hill Practice**	Lancashire
**Riverside Surgery**	Lincolnshire
**Rowner**	Hampshire
**Saddleworth Medical Practice**	Manchester
**Sandy Lane Surgery**	Lancashire
**Sheepmarket Surgery**	Lincolnshire
**Silverlock Medical Centre**	Greater London
**South Chadderton Health Centre**	Manchester
**Spring Street**	Surrey
**St Fillans Medical Centre**	Lancashire
**St Gabriels Medical Centre**	Manchester
**St James Health Centre**	Merseyside
**St Paul'S Medical Centre**	Lancashire
**Station House Surgery**	Cumbria
**Steven Shackman Practice**	Greater London

**Stoneleigh Surgery**	Cumbria
**Stoneleigh Surgery**	Surrey
**Streatham High Practice**	Greater London
**Streatham Place Surgery**	Greater London
**Summertown Health Centre**	Oxfordshire
**Swan Lane Medical Centre**	Manchester
**Thamesmead Health Centre**	Greater London
**The Bridge Practice**	Surrey
**The Calverton Practice**	Nottinghamshire
**The Carnewater Practice**	Cornwall
**The Church Lane Practice**	Greater London
**The Fairfields Practice**	Nottinghamshire
**The Friarsgate Practice**	Hampshire
**The Grange Medical Centre**	Warwickshire
**The Grange Practice**	Kent
**The Hall Practice**	Buckinghamshire
**The Hambleden Clinic**	Greater London
**The Health Centre**	Cumbria
**The Manor Health Centre- Dr Curran & Partners**	Greater London
**The Manor Health Centre- Dr Sheila Santamaria**	Greater London
**The Marshside Surgery**	Merseyside
**The Schoolhouse Surgery**	Cheshire
**The Stokes Medical Centre**	Gloucestershire
**The Valley Surgery**	Nottinghamshire
**The Whipton Surgery**	Devon
**Thornton Road Surgery**	Greater London
**Trowbridge Surgery**	Greater London
**Tyntesfield Medical Group(Nailsea)**	Somerset
**Vauxhall Primary Health Centre**	Merseyside
**Victoria Park Practice**	Merseyside
**Villa Med Ctr**	Merseyside
**Village Surgery**	South Yorkshire
**Vine Medical Group**	Hampshire
**Warlingham Green Med Practice**	Surrey
**Wellside Surgery**	Cambridgeshire
**West Common Lane Medical Ctr**	Lincolnshire
**West Timperley Medical Centre**	Manchester
**Westongrove Partners**	Buckinghamshire
**Whalley Medical Centre**	Lancashire
**White Rose Surgery**	West Yorkshire
**Whitechapel Health Centre**	Greater London
**Windermere And Bowness Med Practice**	Cumbria
**Wolstanton Medical Centre**	Staffordshire
**Wonford Green Surgery**	Devon

**Woodbridge Hill Surgery**	Surrey
**Worden Medical Centre**	Lancashire

12. Contributors

Contributors

Simon de Lusignan

Chris McGee

Rebecca Webb

Rachel Byford

Sameera Pathirannehelage

Ivelina Yonova

Mariya Hrishkova

Filipa Ferreira

Imran Rafi

Contact details

RCGP Research & Surveillance Centre
University of Surrey
Clinical Medicine & Ageing
GUILDFOFD
GU2 7PX
Tel: +44 (0)1483 684802

Medical Director: Professor Simon de Lusignan
MedicalDirectorRSC@rcgp.org.uk

Practice Liaison Officer: Ivelina Yonova
i.yonova@surrey.ac.uk
Tel: +44 (0)1483 682758

13. Appendices

APPENDIX A : Weekly Disease Incidence Graphs

1. Water and Food Borne Disorders:

National | North | South | London | Midlands And East | 5yr Avg

Intestinal Infections (All ages) (ICD10 : A00-A09)

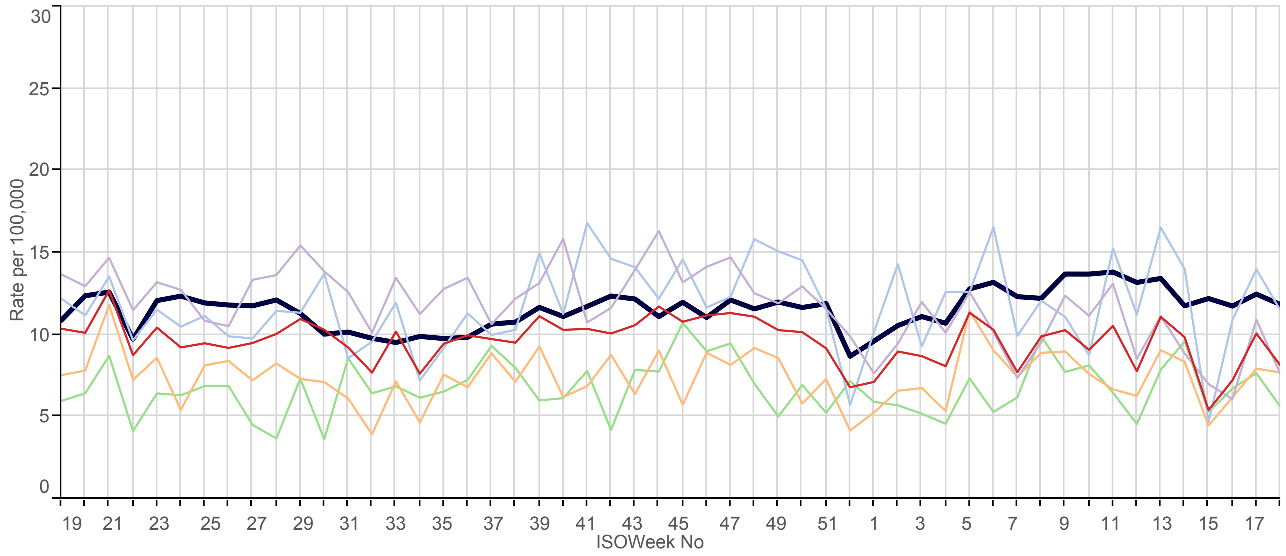

Intestinal Infections (0-4 years) (ICD10 : A00-A09)

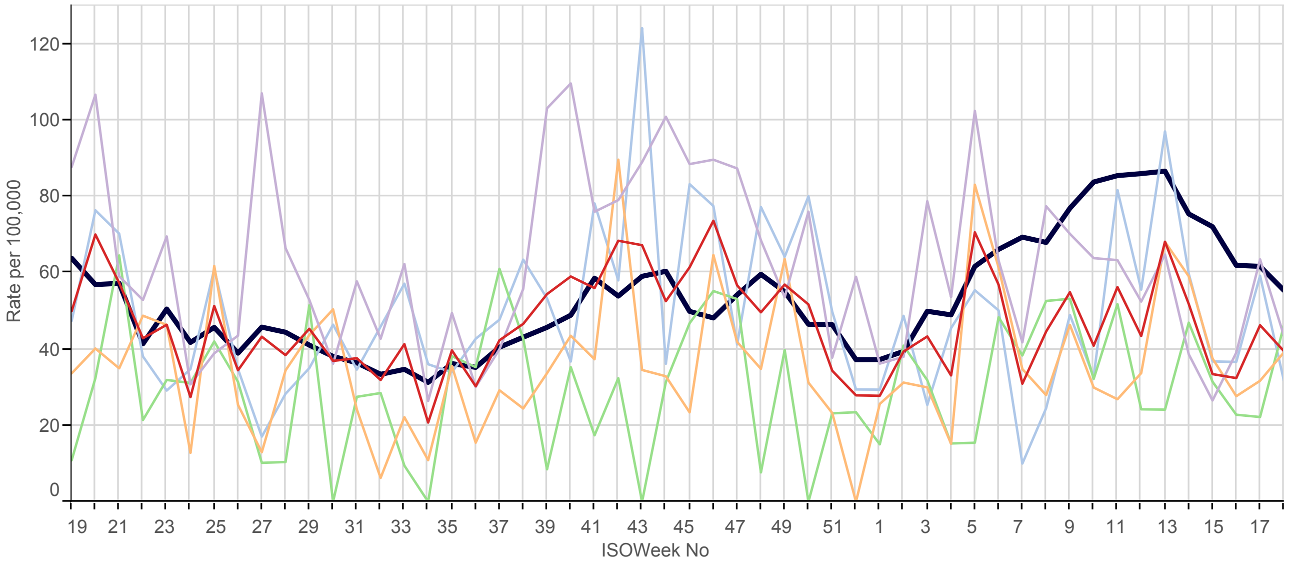

Non-infective Enteritis / Colitis (ICD10 : K50-K52)

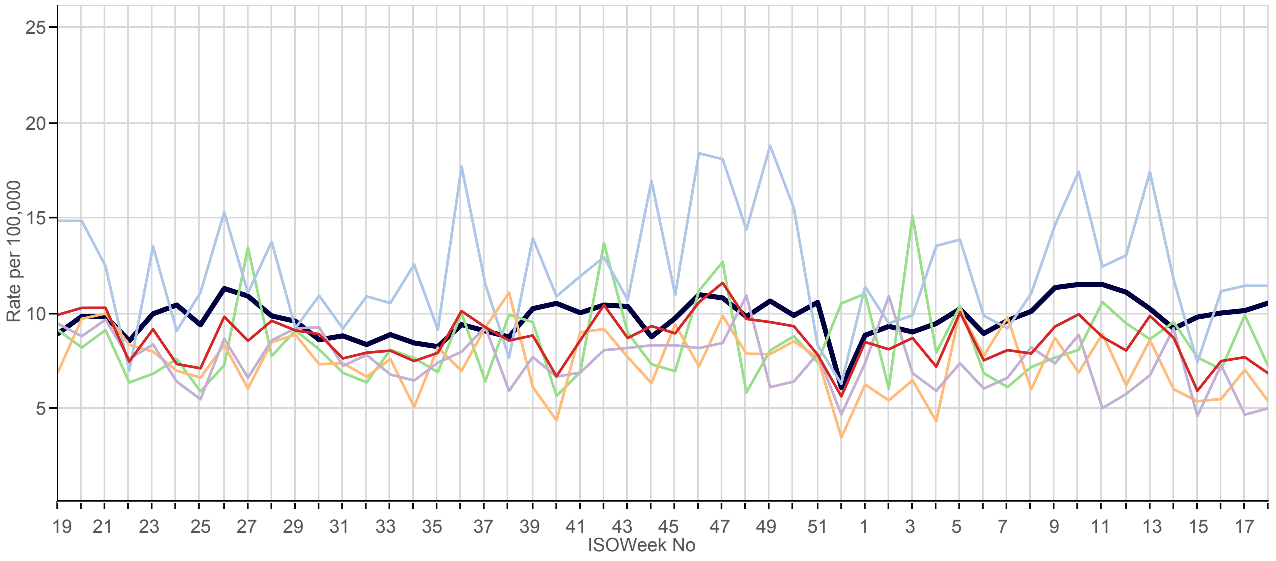

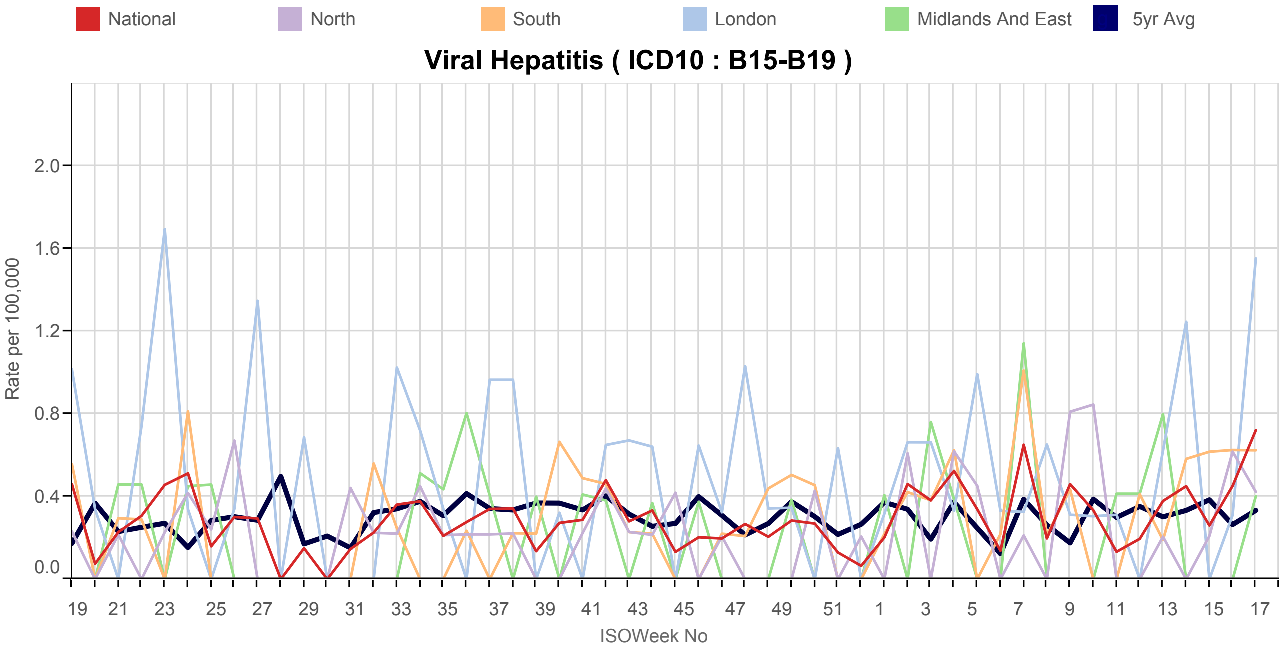
National
North
South
London
Midlands And East
5yr Avg
Viral Hepatitis (ICD10 : B15-B19)
Rate per 100,000
2.0
1.6
1.2
0.8
0.4
0.0
19
21
23
25
27
29
31
33
35
37
39
41
43
45
47
49
51
1
3
5
7
9
11
13
15
17
ISOWeek No

2. Environmentally Sensitive Disorders:

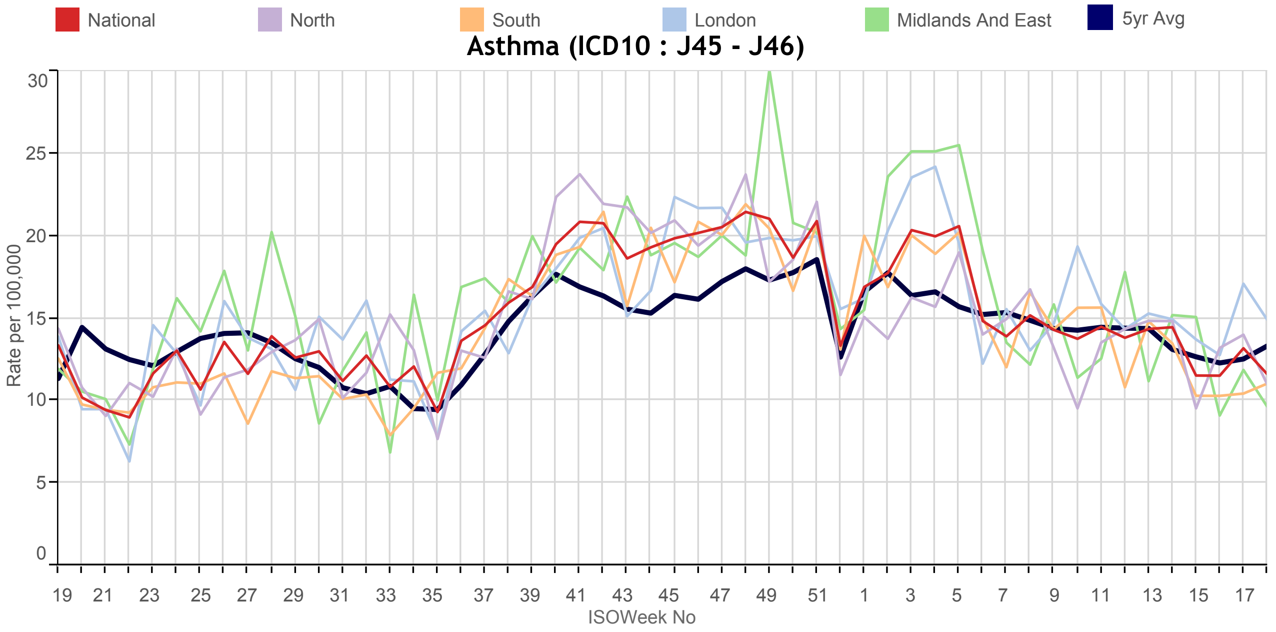

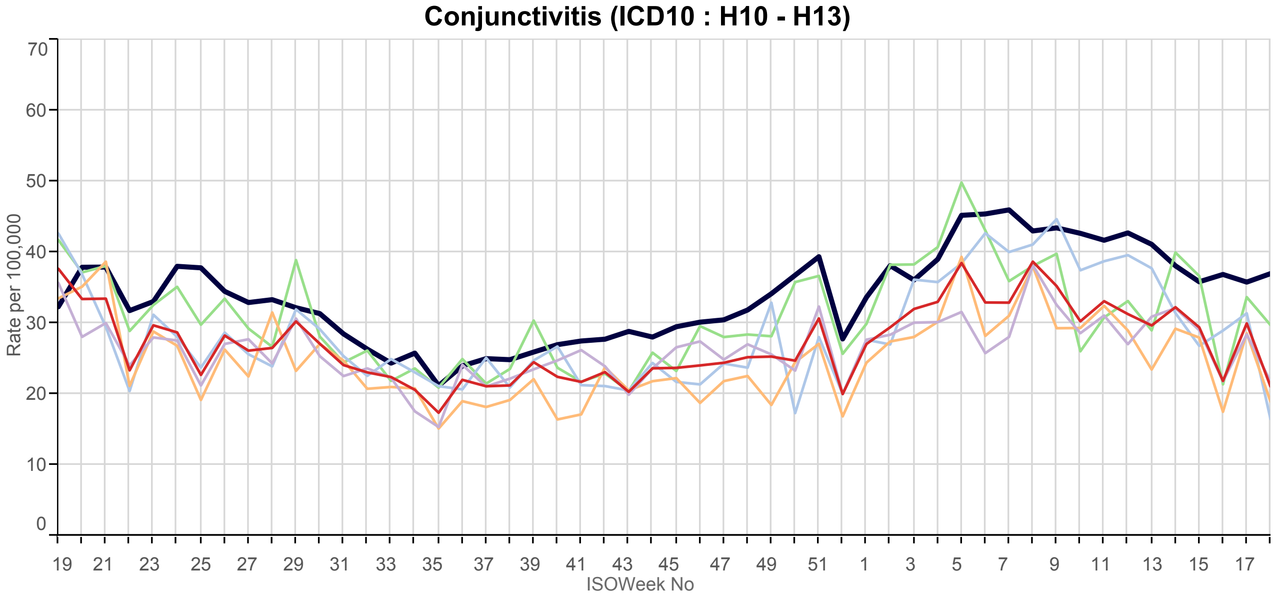

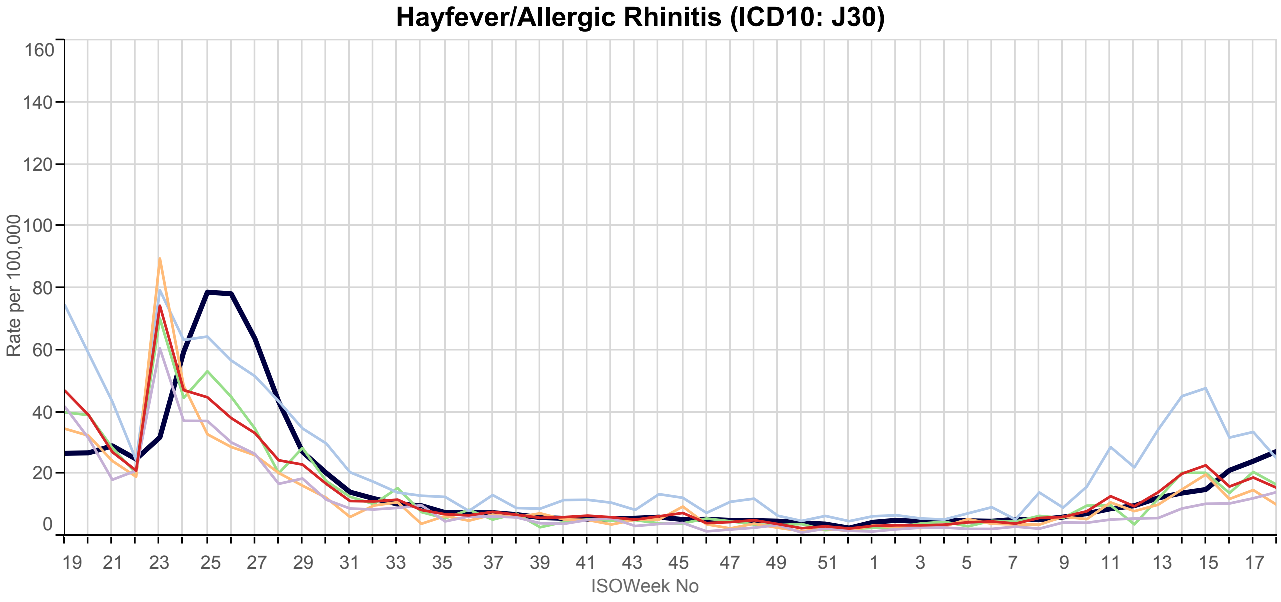

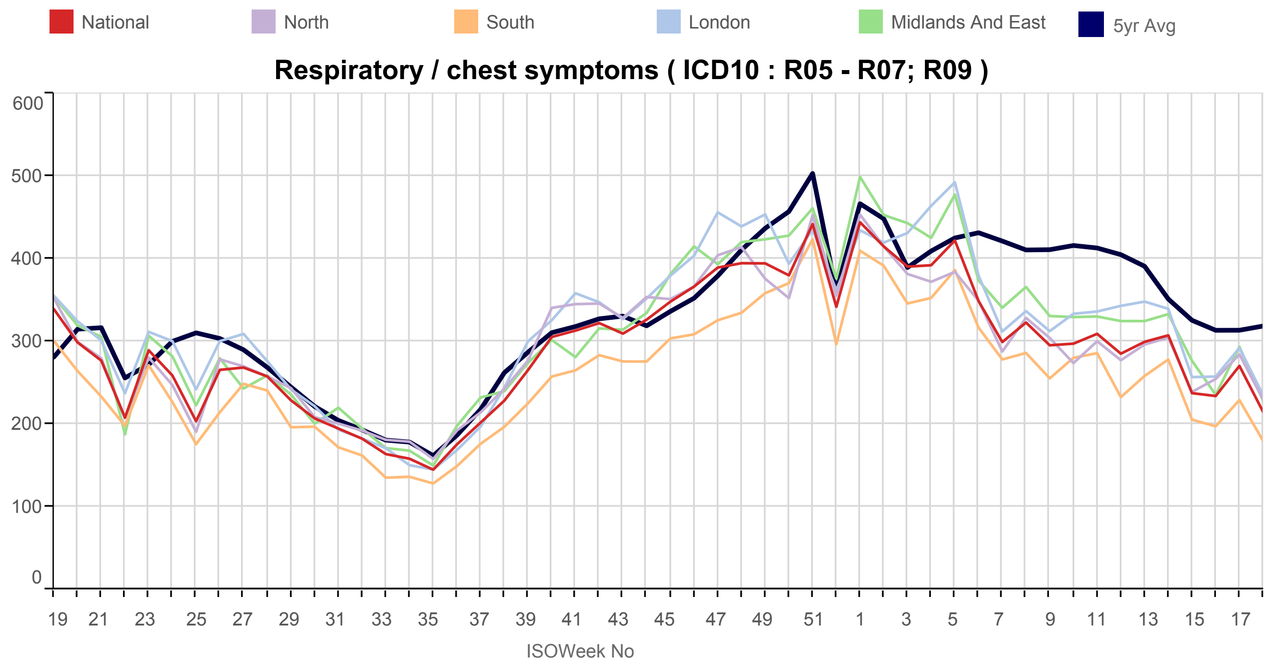
National
North
South
London
Midlands And East
5yr Avg
Respiratory / chest symptoms (ICD10 : R05 - R07; R09)
600
500
400
300
200
100
0
19
21
23
25
27
29
31
33
35
37
39
41
43
45
47
49
51
1
3
5
7
9
11
13
15
17
ISOWeek No

3. Respiratory Infections:

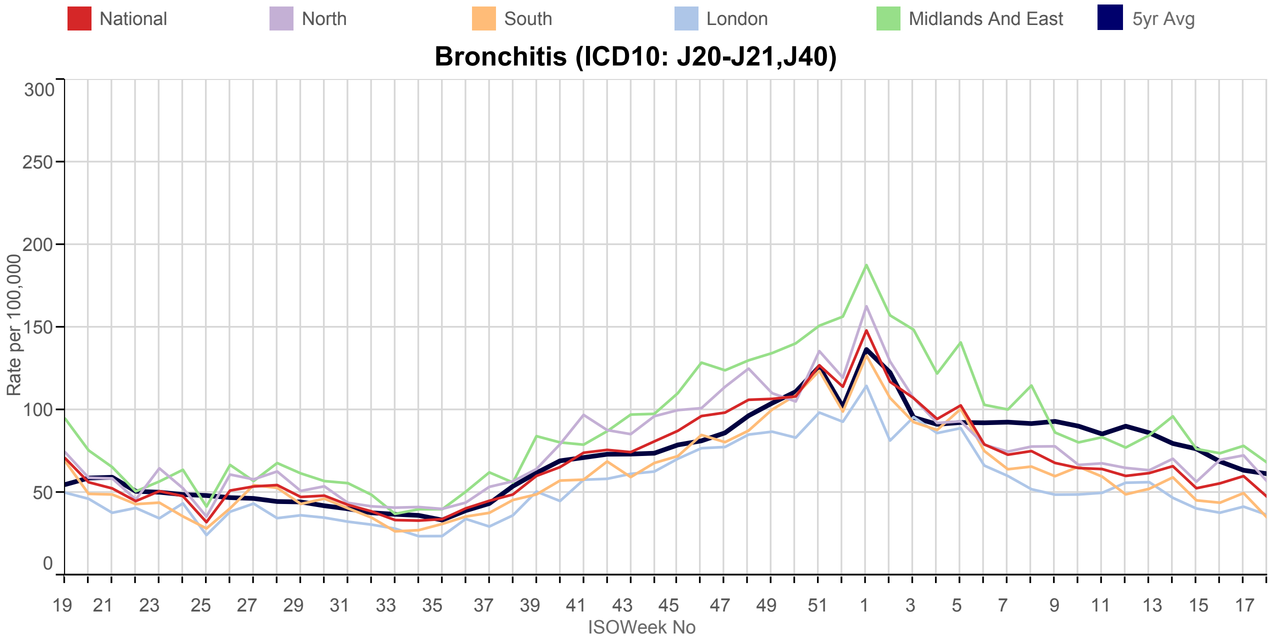

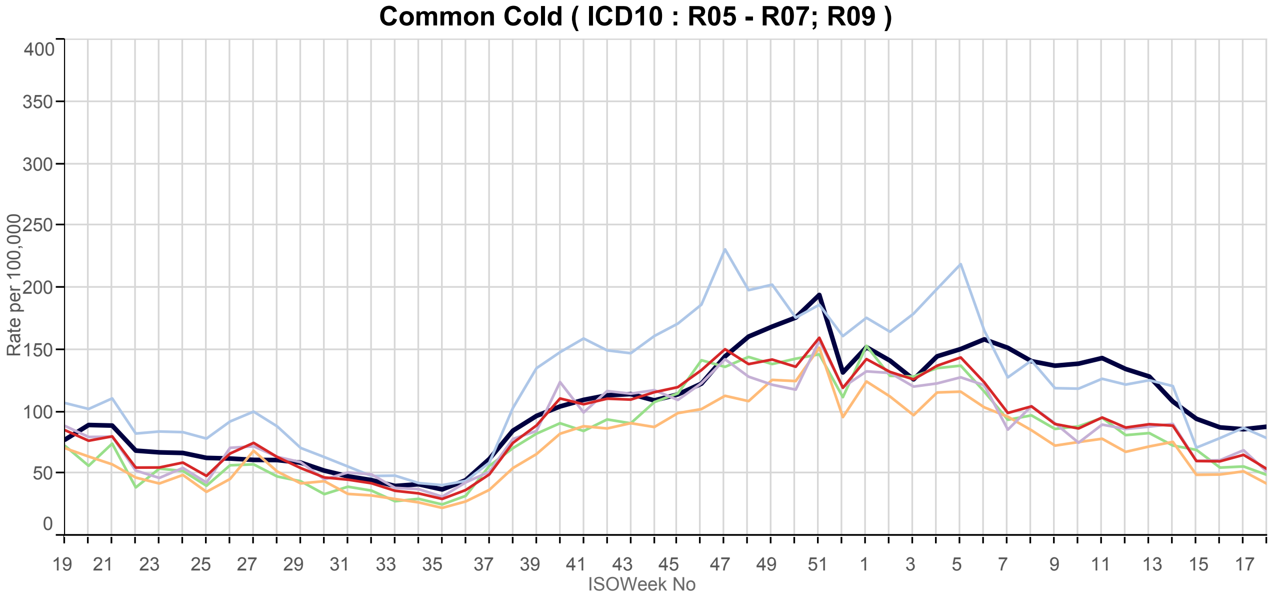

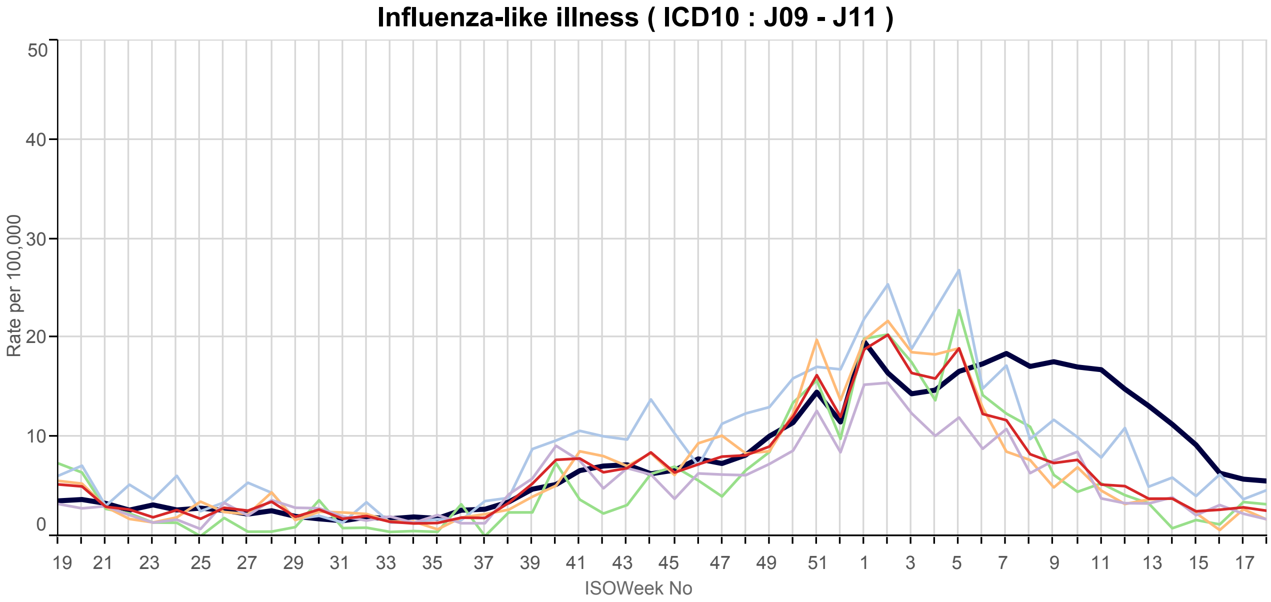

Laryngitis / Tracheitis (ICD10 : J04)

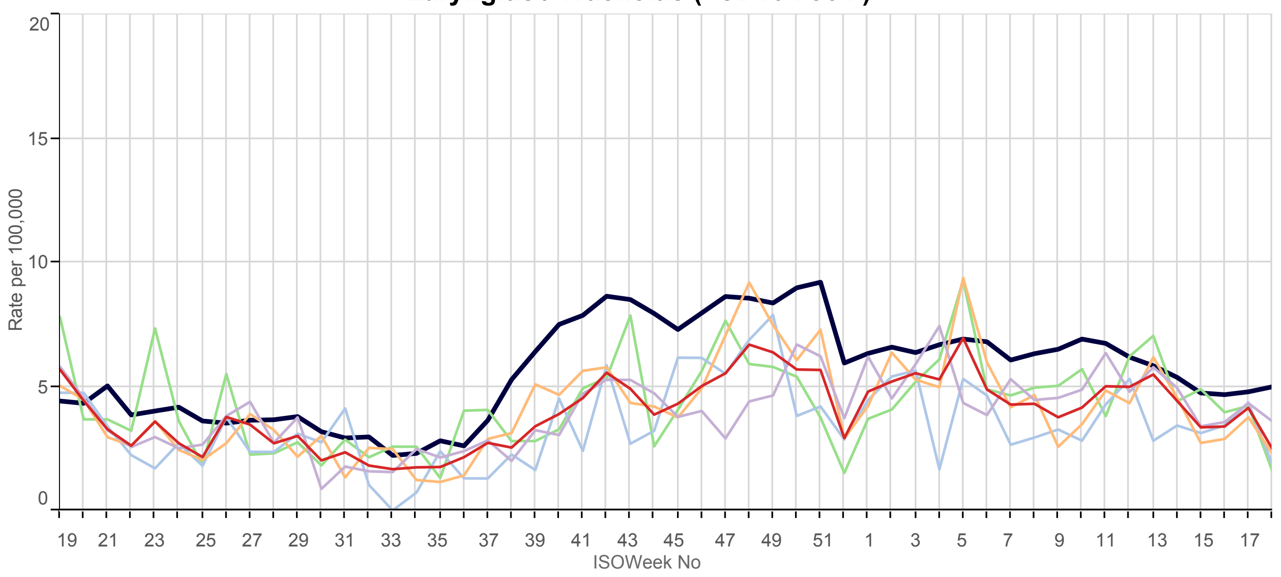

Pleurisy (ICD10 : R091)

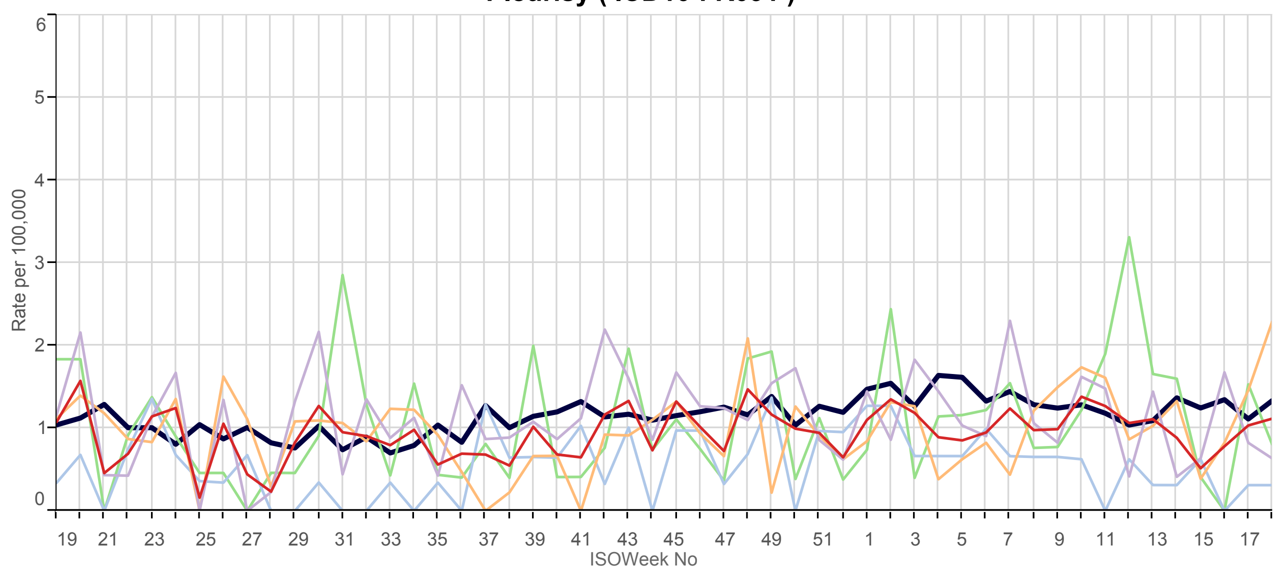

Pneumonia / Pneumonitis (ICD10 : J12 - J18)

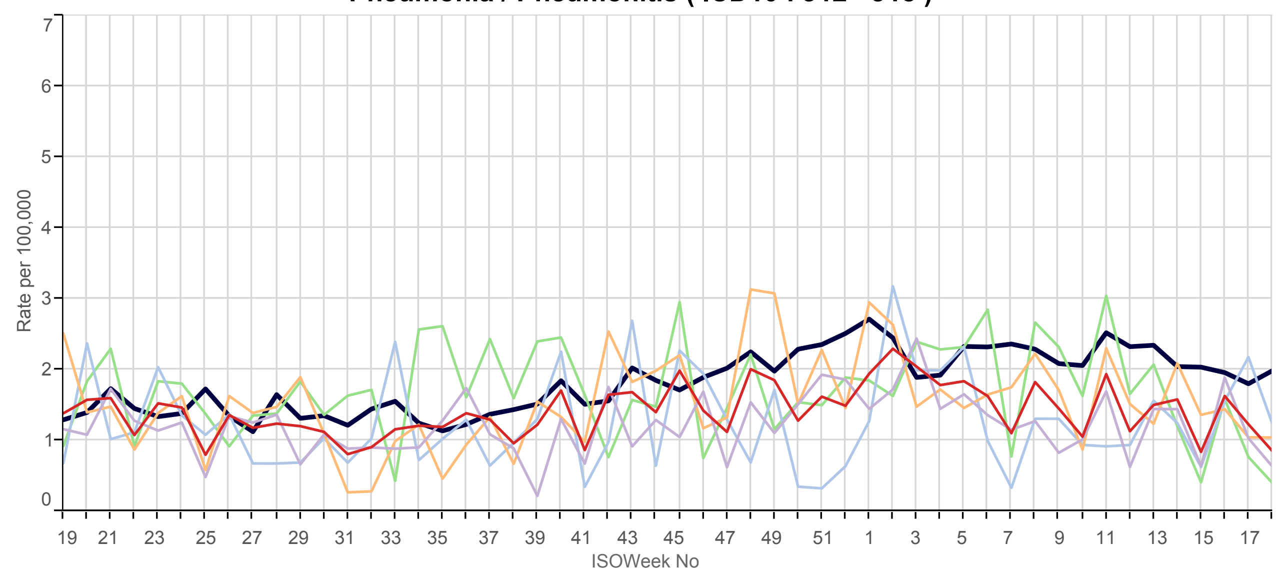

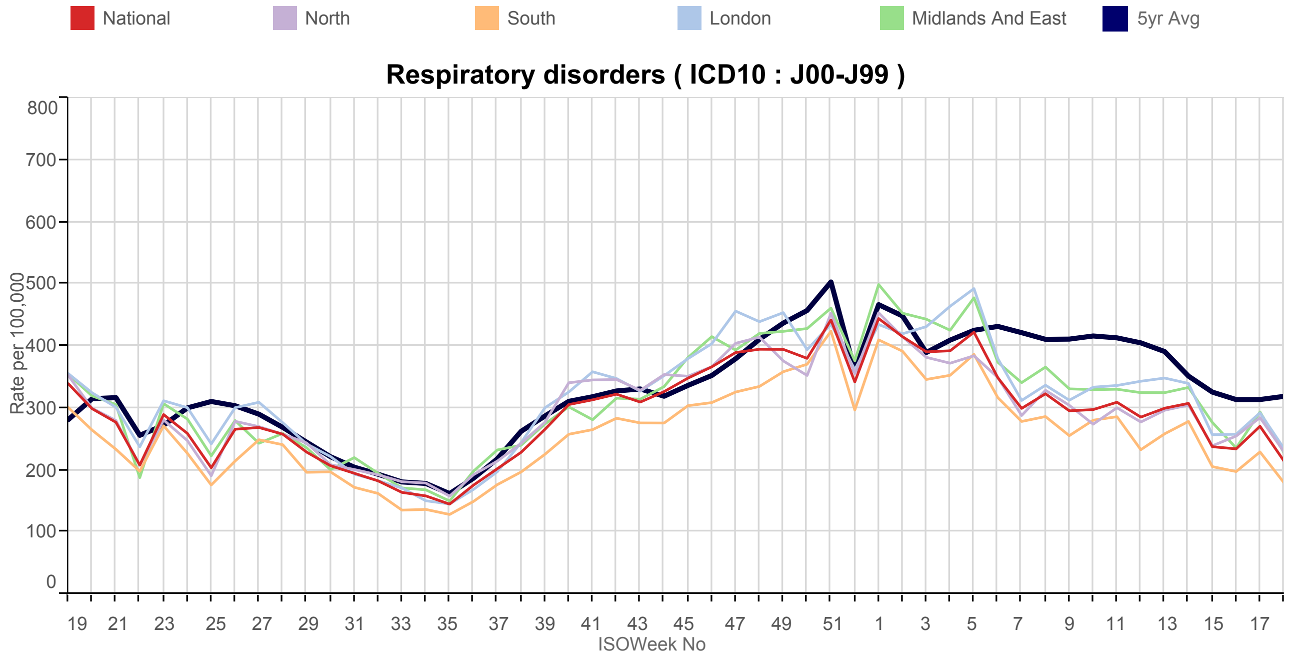

National
North
South
London
Midlands And East
5yr Avg
Respiratory disorders (ICD10 : J00-J99)
Rate per 100,000
ISOWeek No

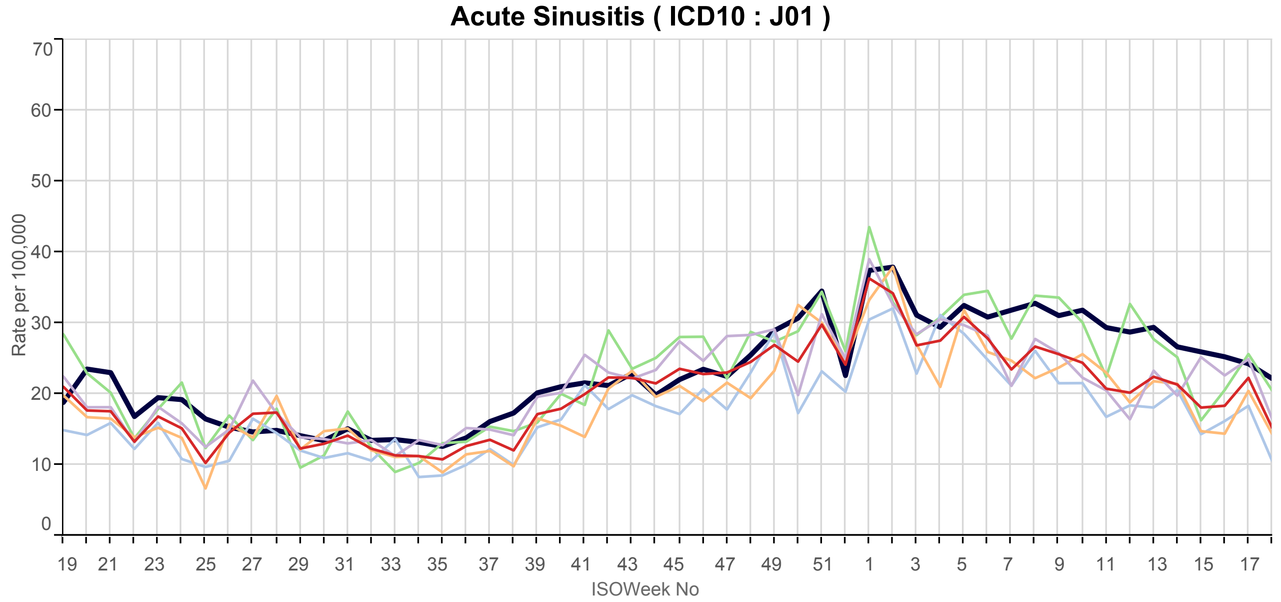

Acute Sinusitis (ICD10 : J01)
Rate per 100,000
ISOWeek No

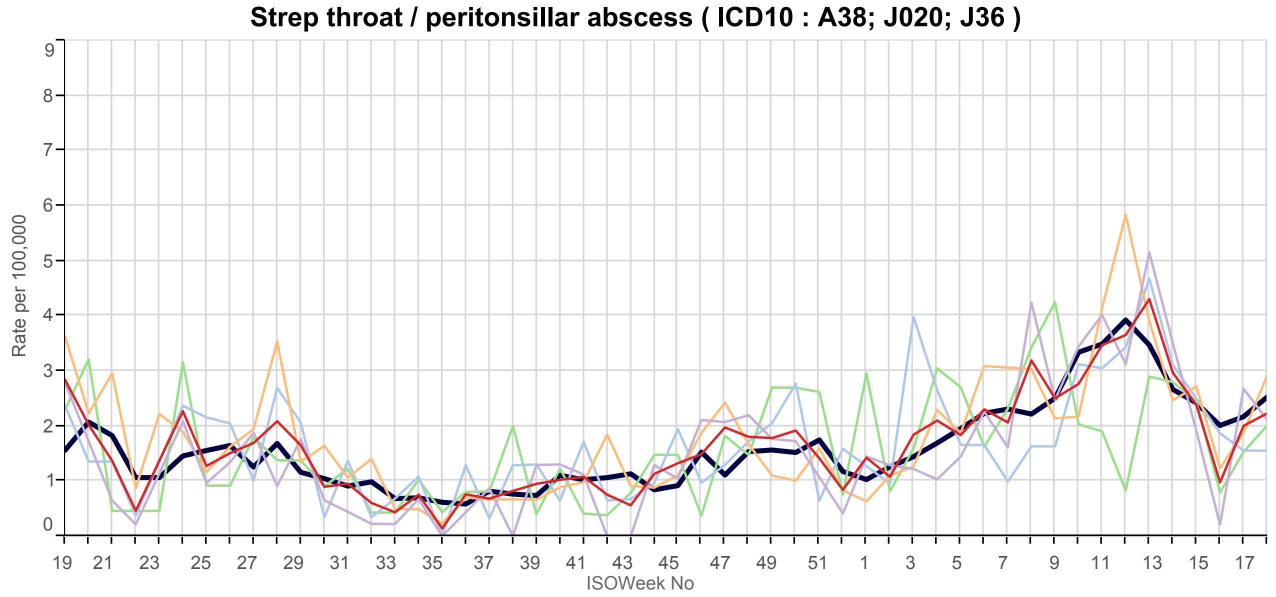

Strep throat / peritonsillar abscess (ICD10 : A38; J020; J36)
Rate per 100,000
ISOWeek No

National North South London Midlands And East 5yr Avg

Tonsilitis / Pharyngitis (ICD10 : J02 - J03)

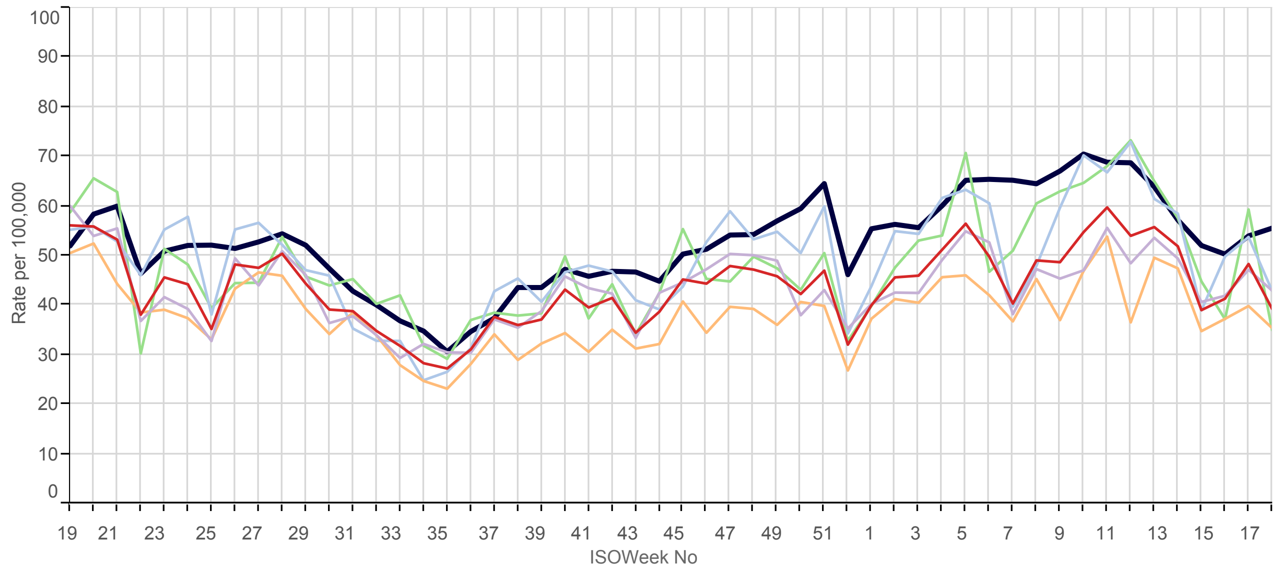

Upper Respiratory Tract Infections (ICD10 : J00 - J06)

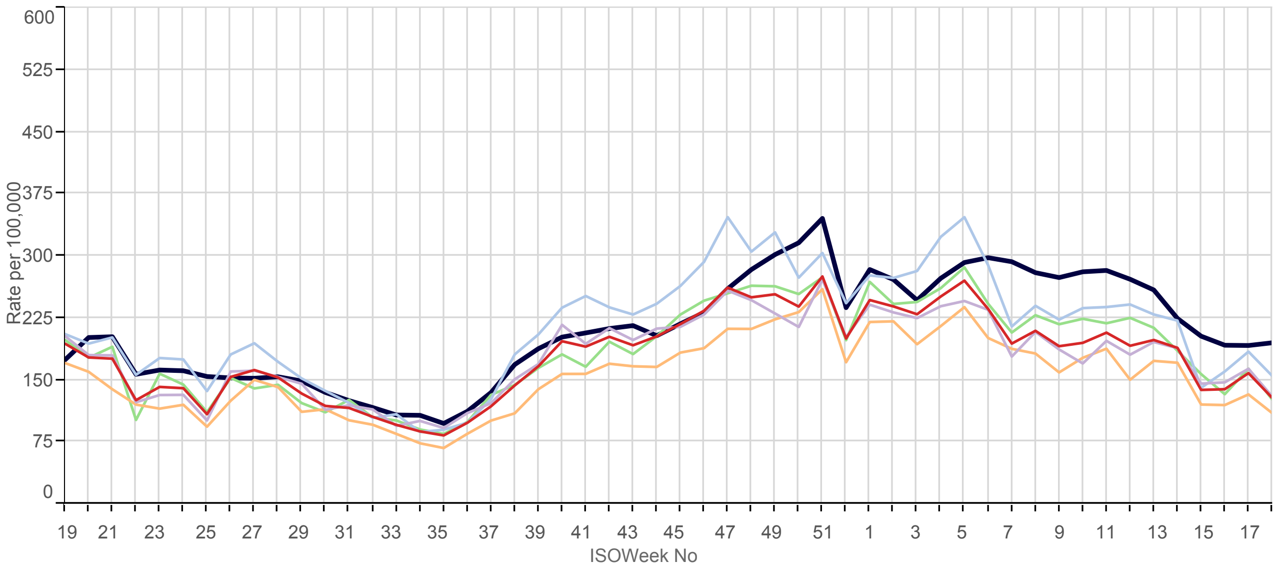

Pertussis (ICD10 : A37)

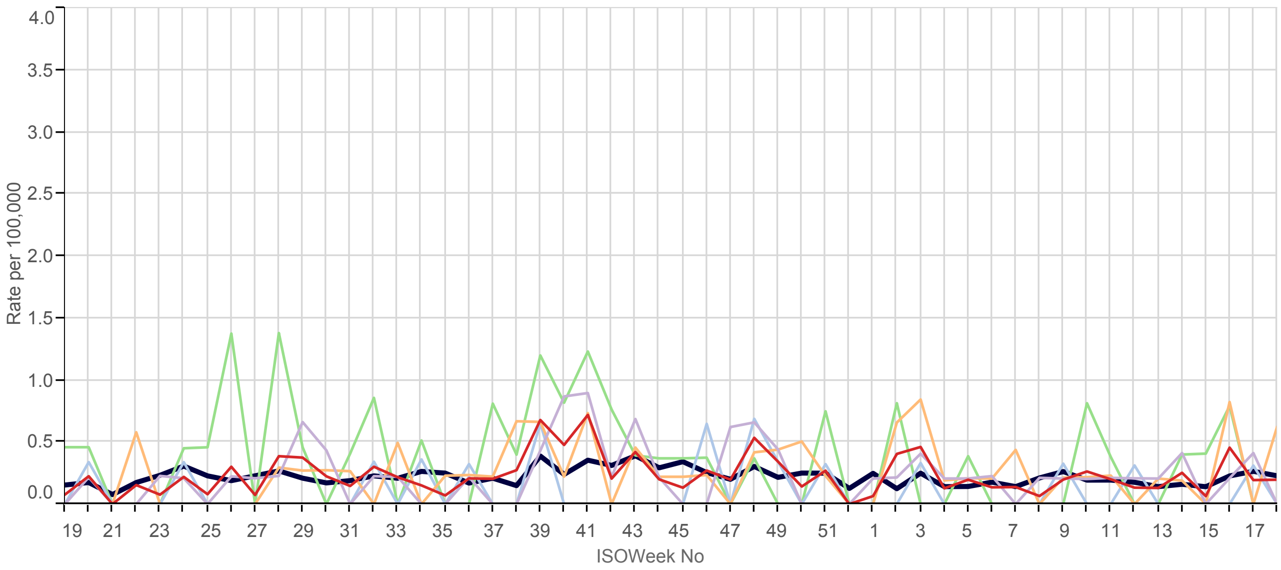

National | North | South | London | Midlands And East | 5yr Avg

Infectious Mononucleosis (ICD10 : B27)

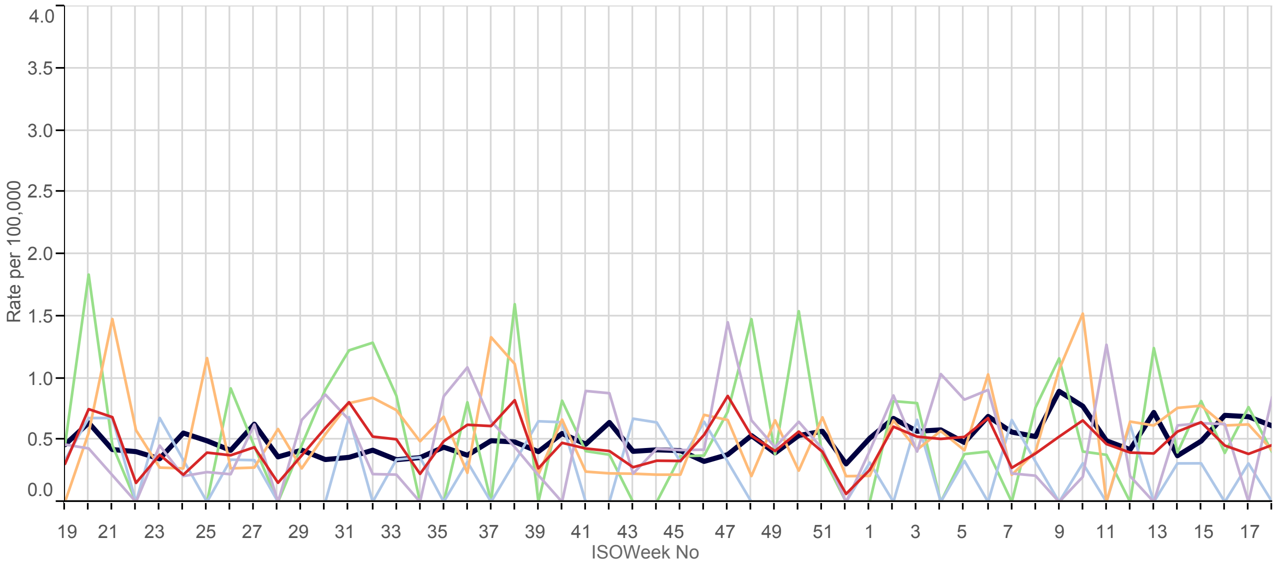

Lower Respiratory Tract Infections (ICD10 : J20-J22)

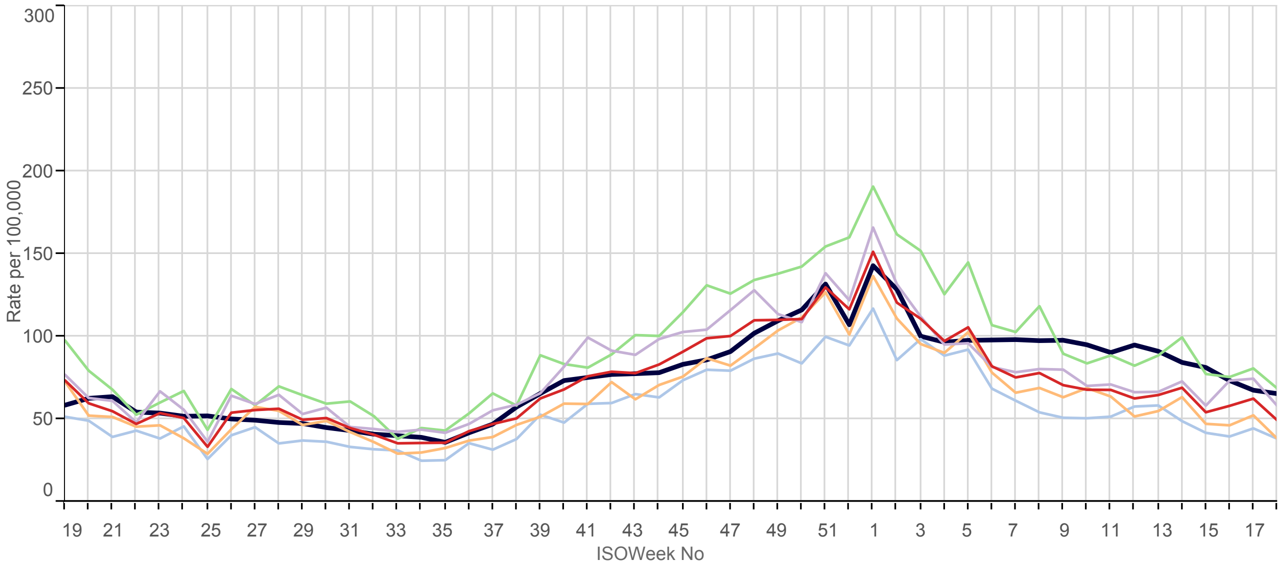

Acute Otitis Media (ICD10 : H650 - H651; H660; H669)

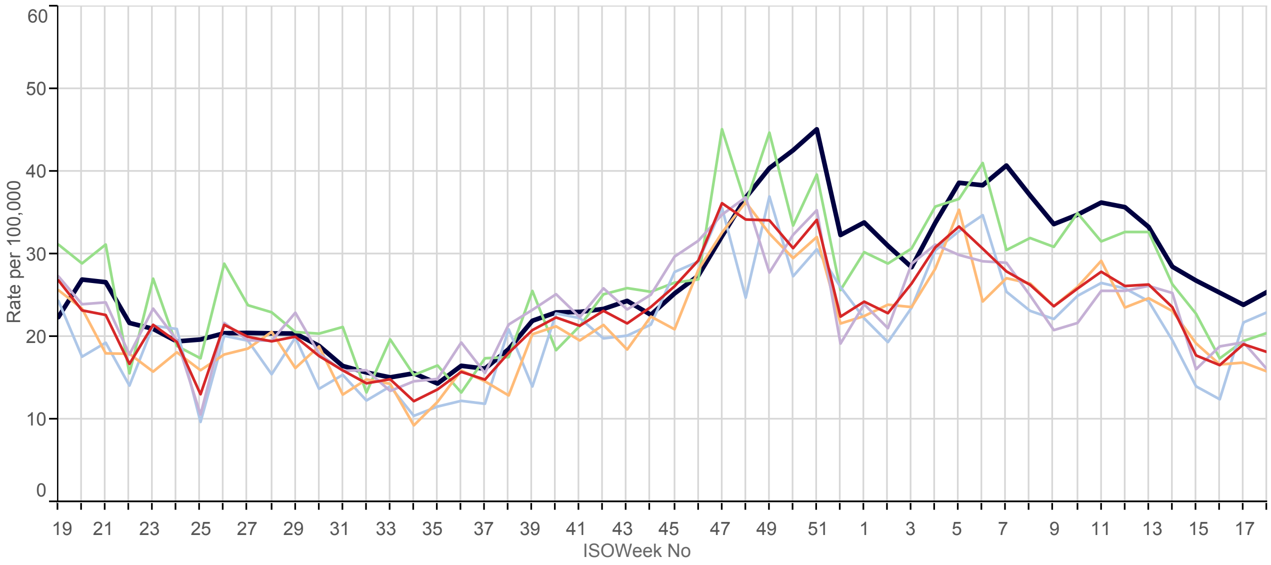

4. Vaccine Sensitive Disorders:

Measles (ICD10 : B05)

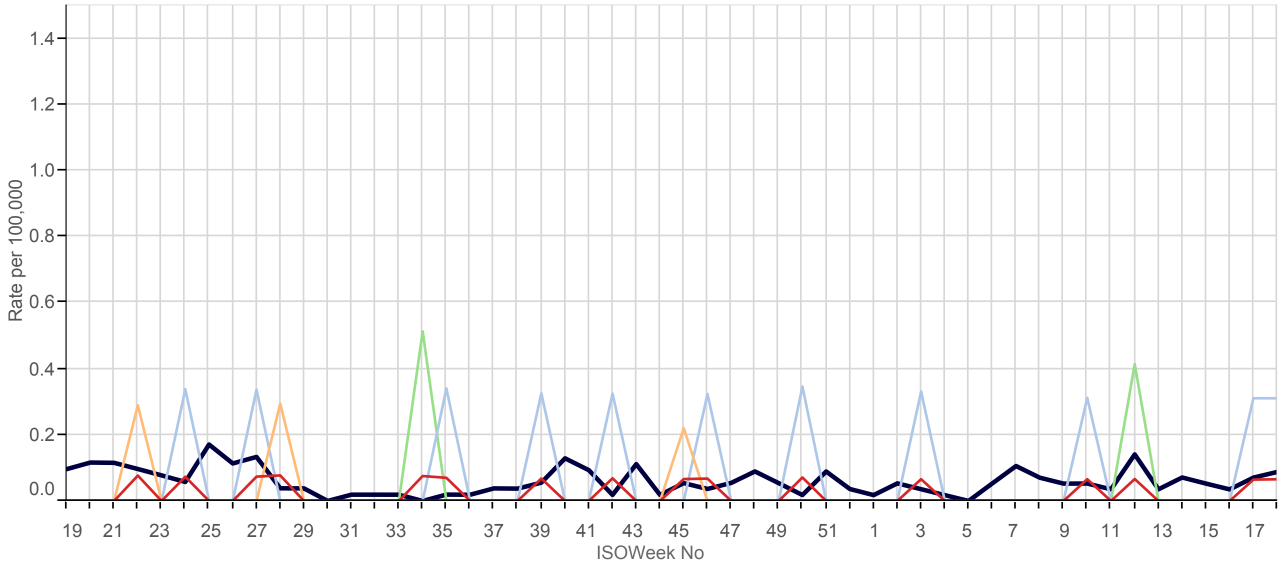

Mumps (ICD10 : B26)

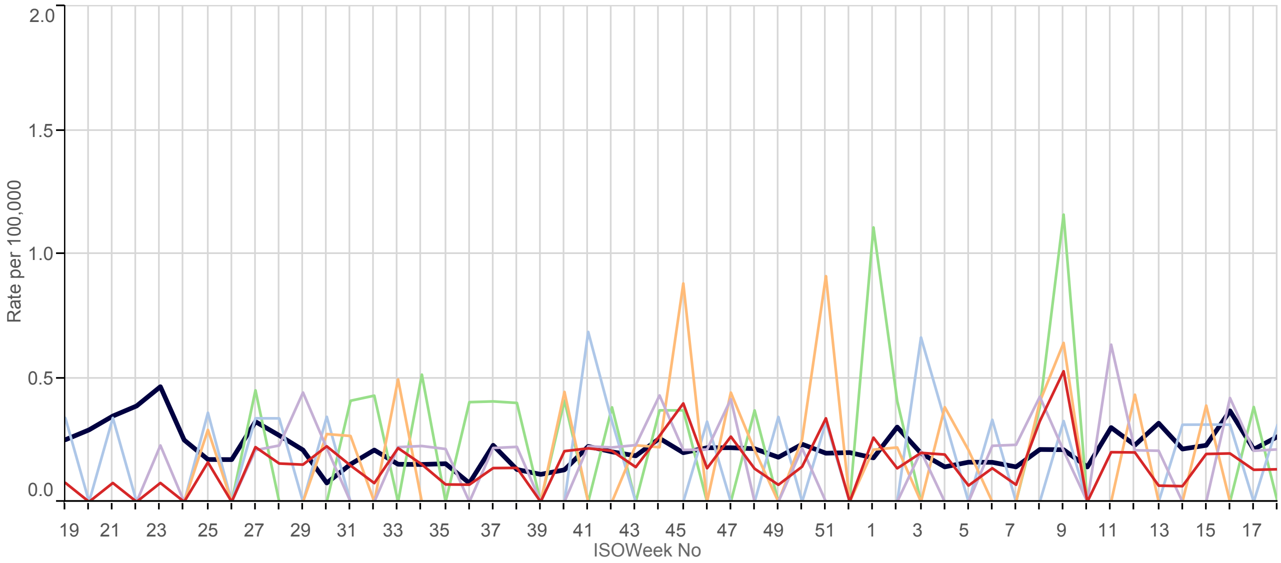

Rubella (ICD10 : B06)

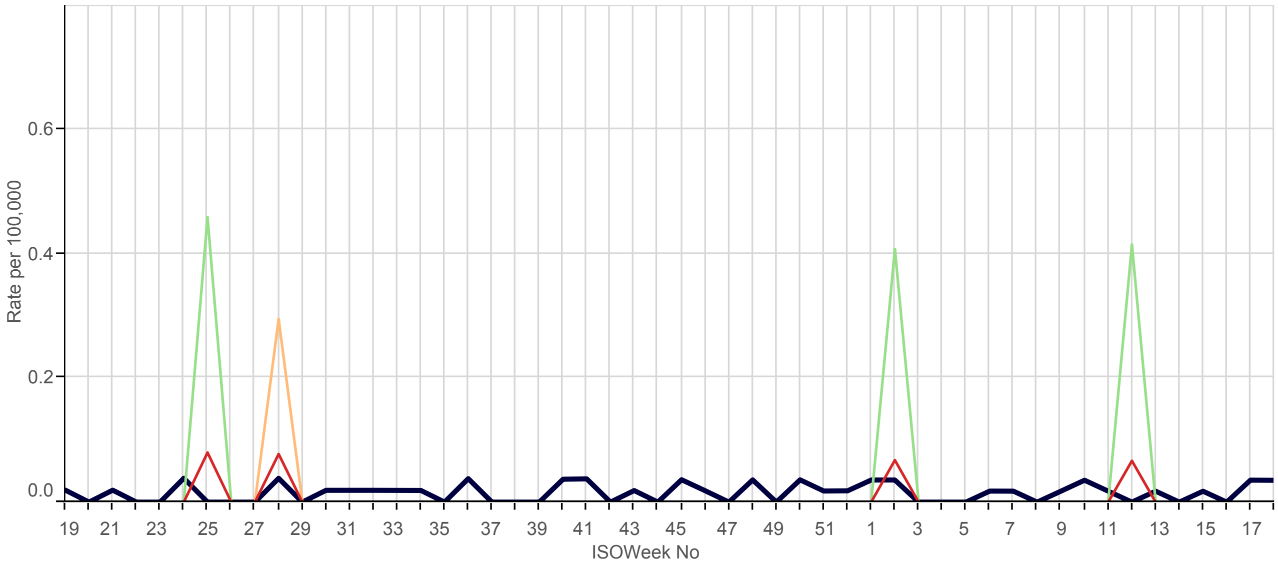

5. Skin Contagions:

National North South London Midlands And East 5yr Avg

Bullous Dermatoses (ICD10 : L10 - L14)

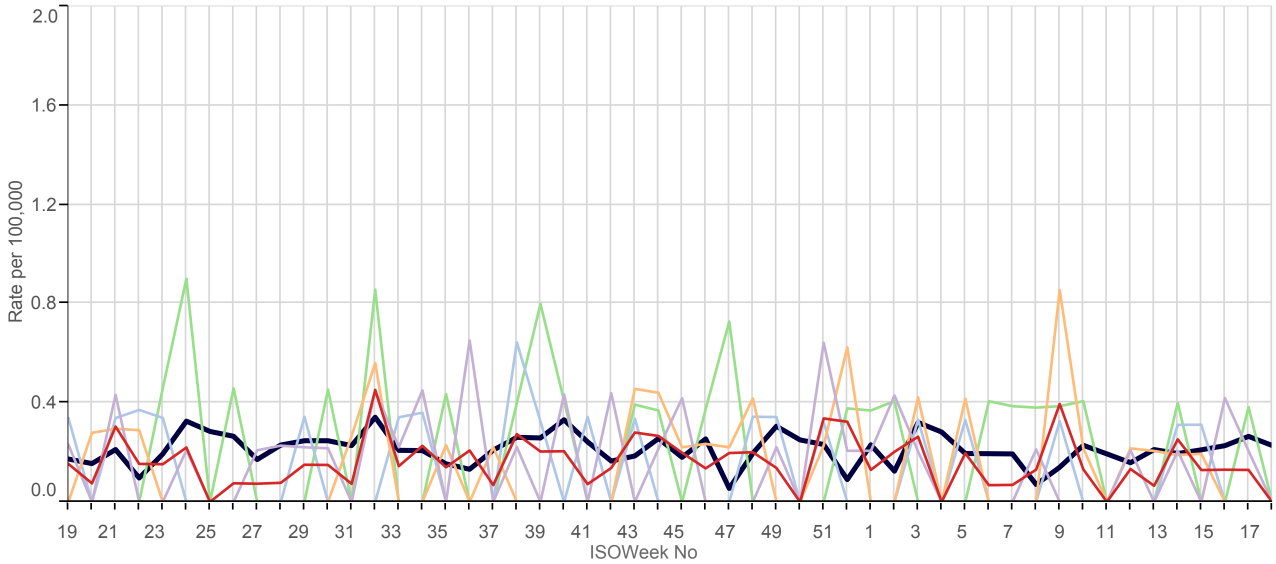

Chickenpox (ICD10 : B01)

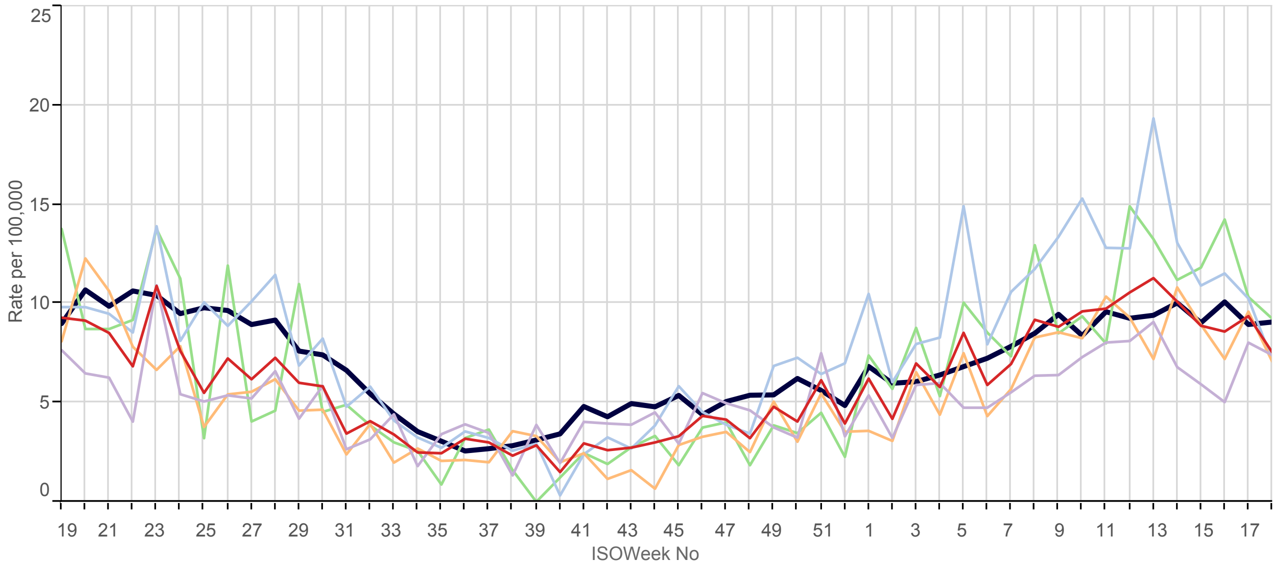

Herpes Simplex (ICD10 : B00)

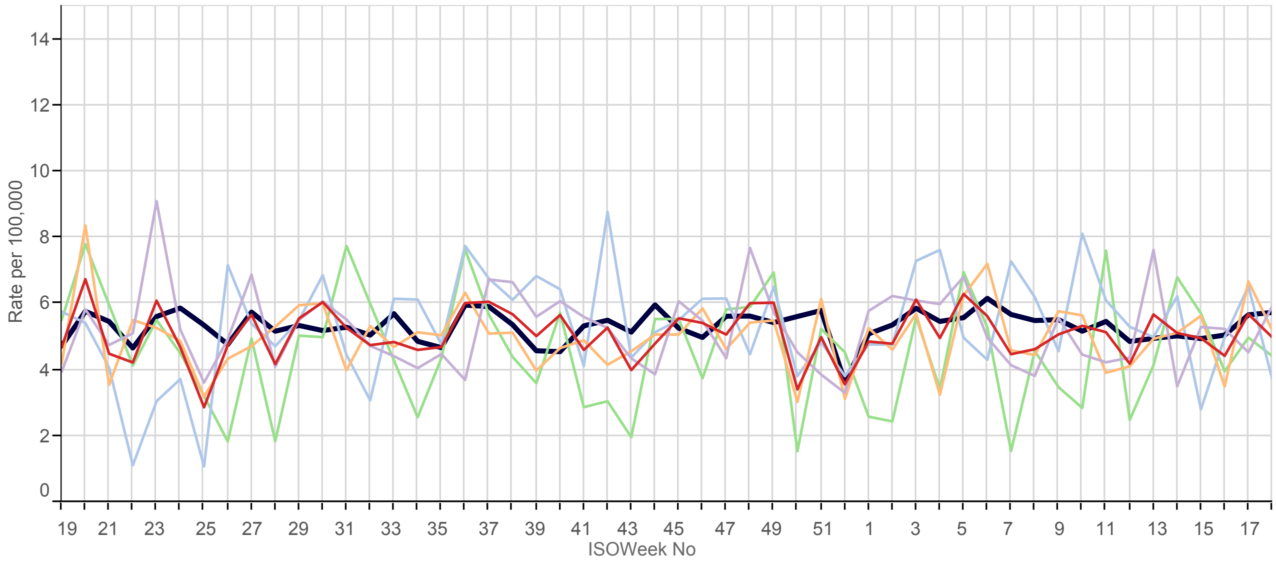

Herpes Zoster (ICD10 : B02)

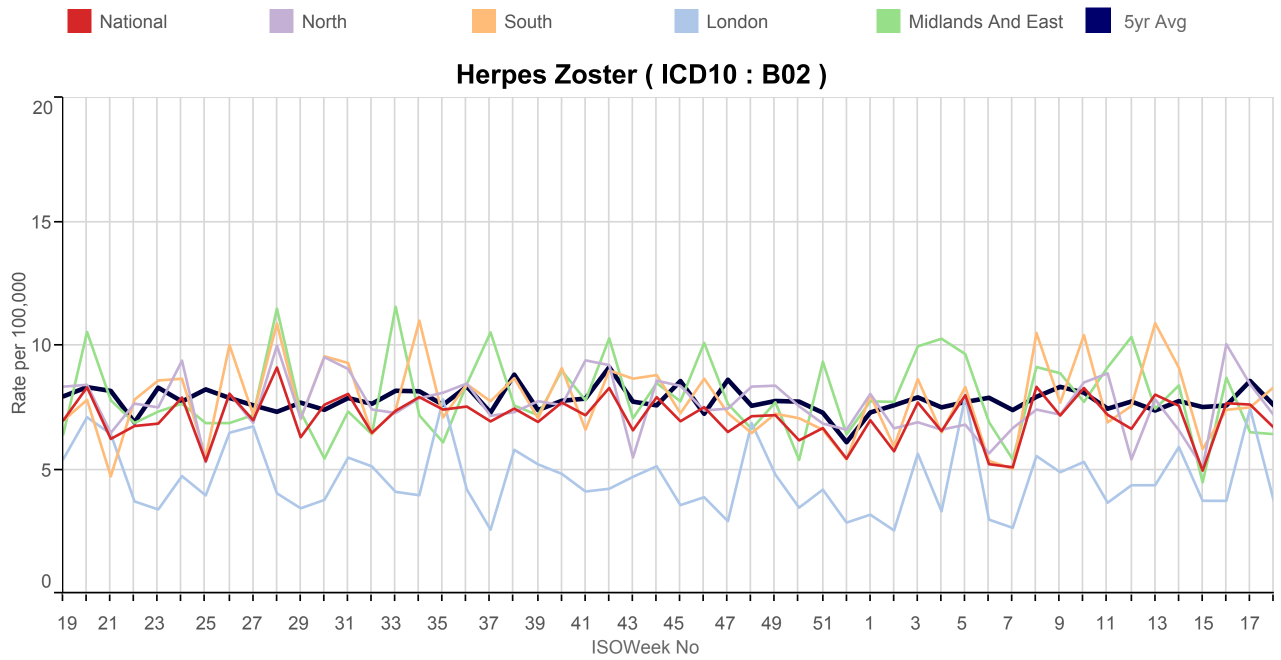

Skin / subcutaneous infections (ICD10 : L00 - L08)

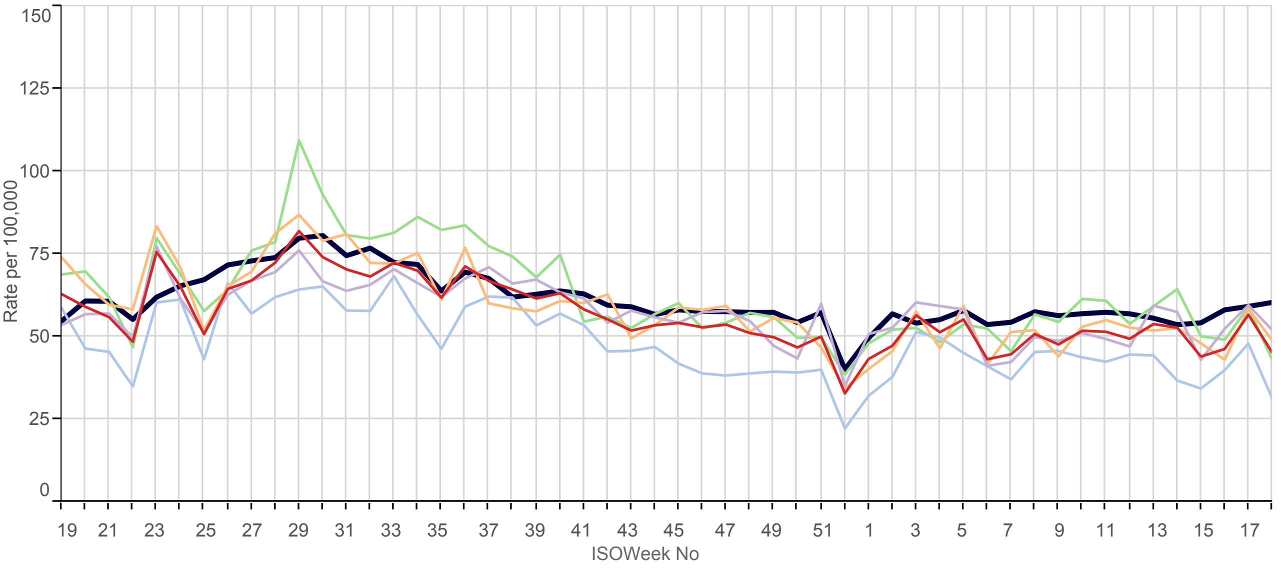

Scabies (ICD10 : B86)

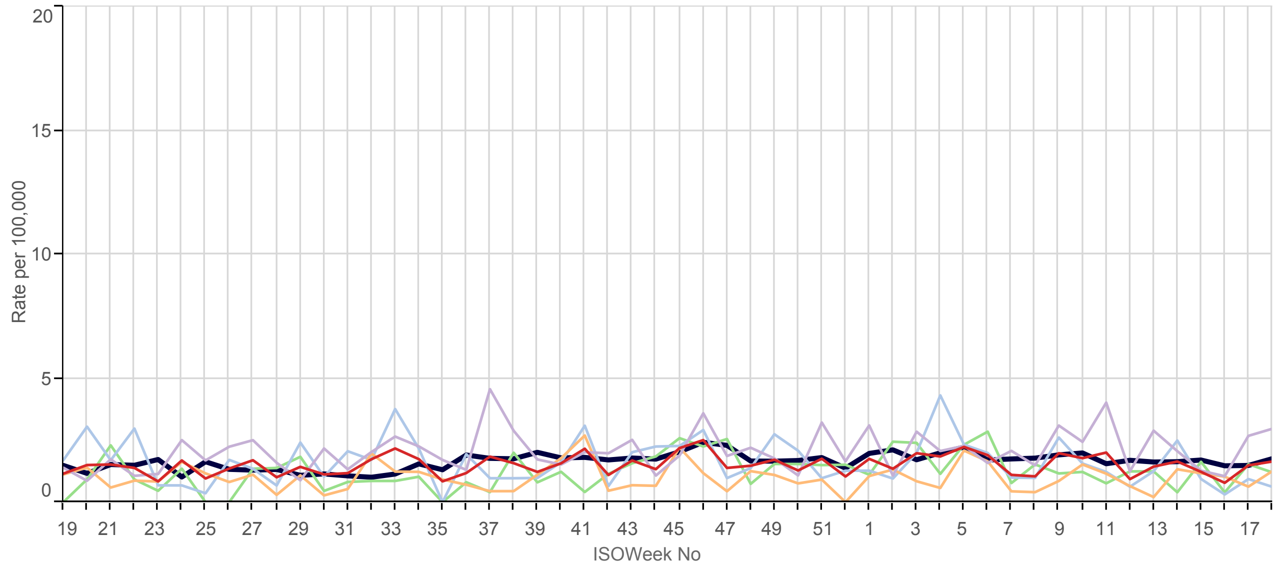

National North South London Midlands And East 5yr Avg

Skin symptoms (ICD10 : R20 - R23)

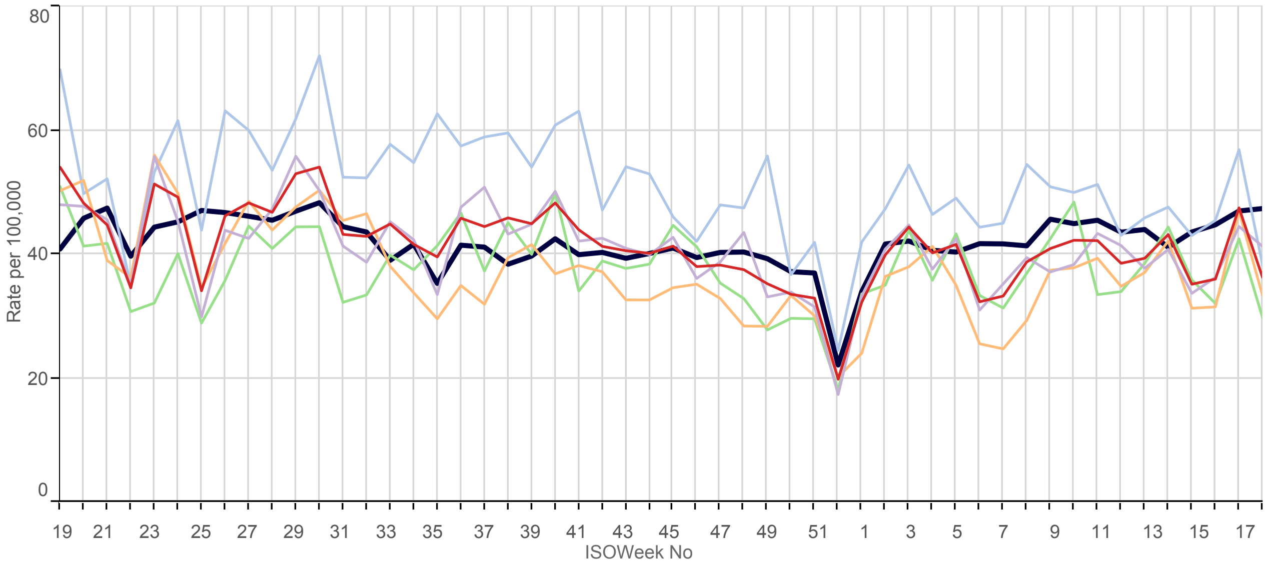

Impetigo (ICD10 - L01)

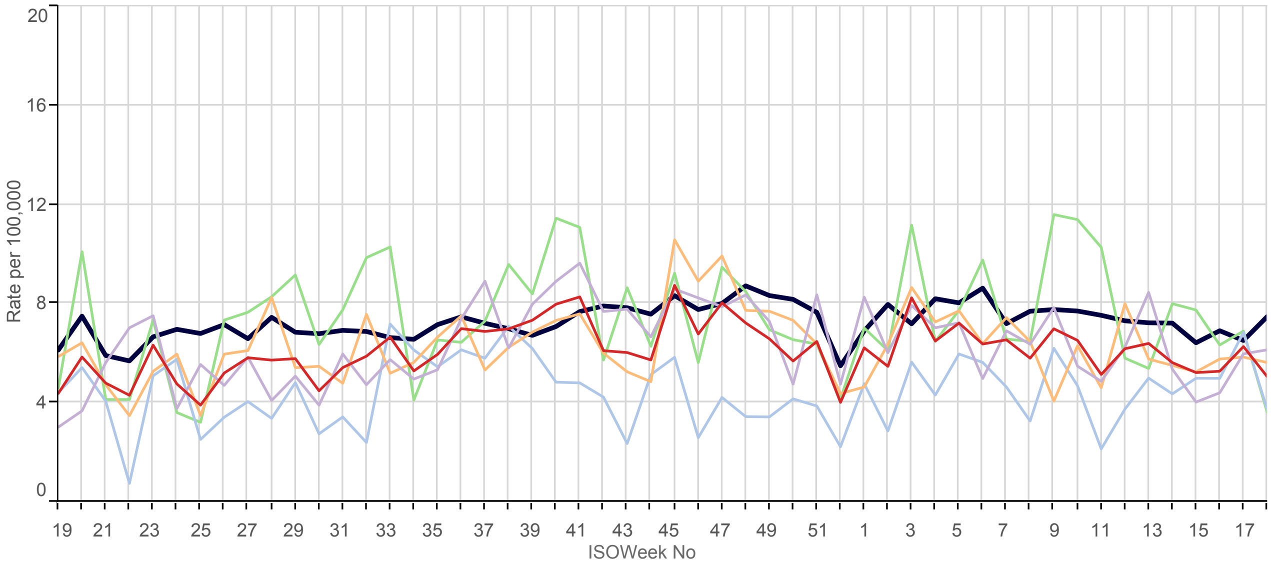

6. Disorders Affecting the Nervous System:

National North South London Midlands And East 5yr Avg

Peripheral Neuropathy (ICD10 : G50 - G64; G70 - G72)

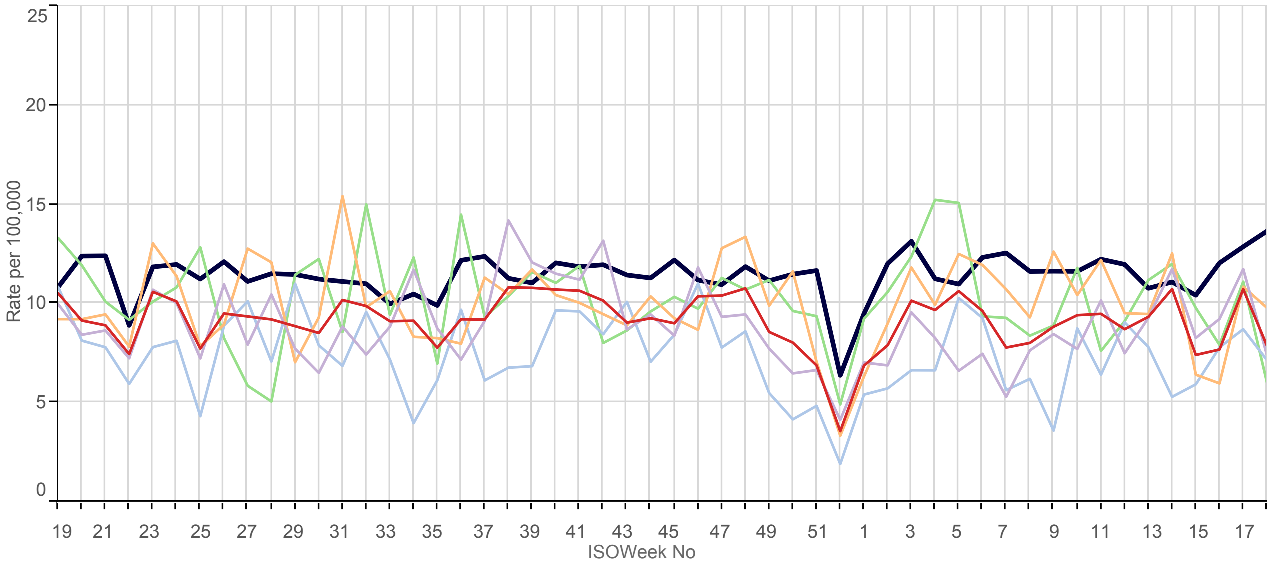

Meningitis and Encephalitis (ICD10 : A170 - A171; A 390; A83 - A85; A87; G00 - G05)

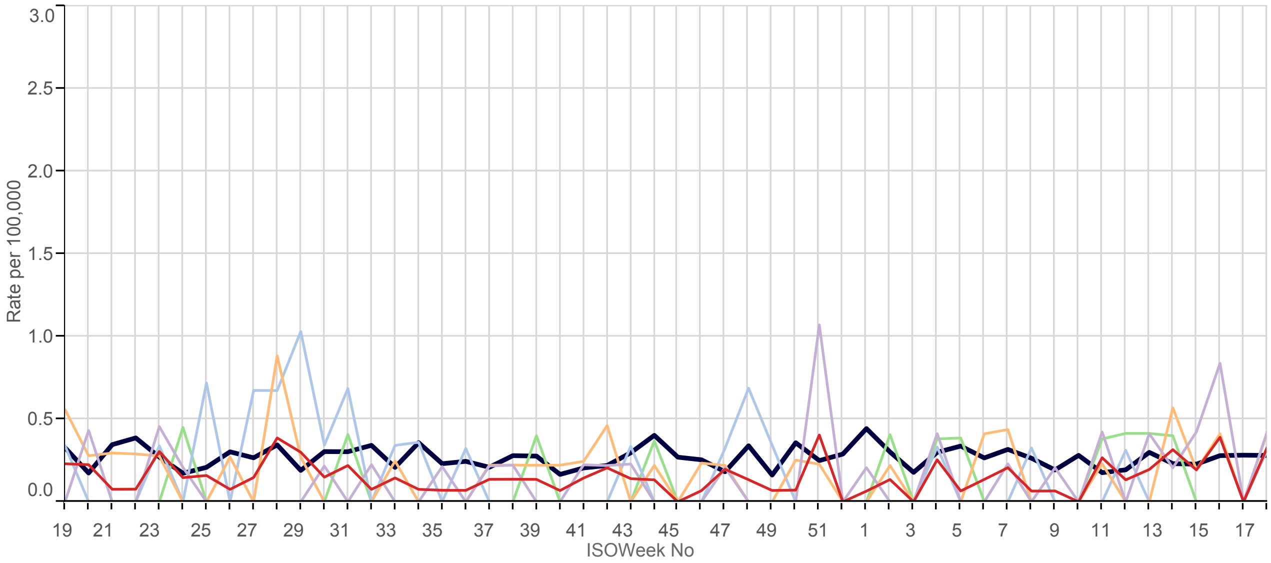

Musculoskeletal symptoms (ICD10 : R25 - R29)

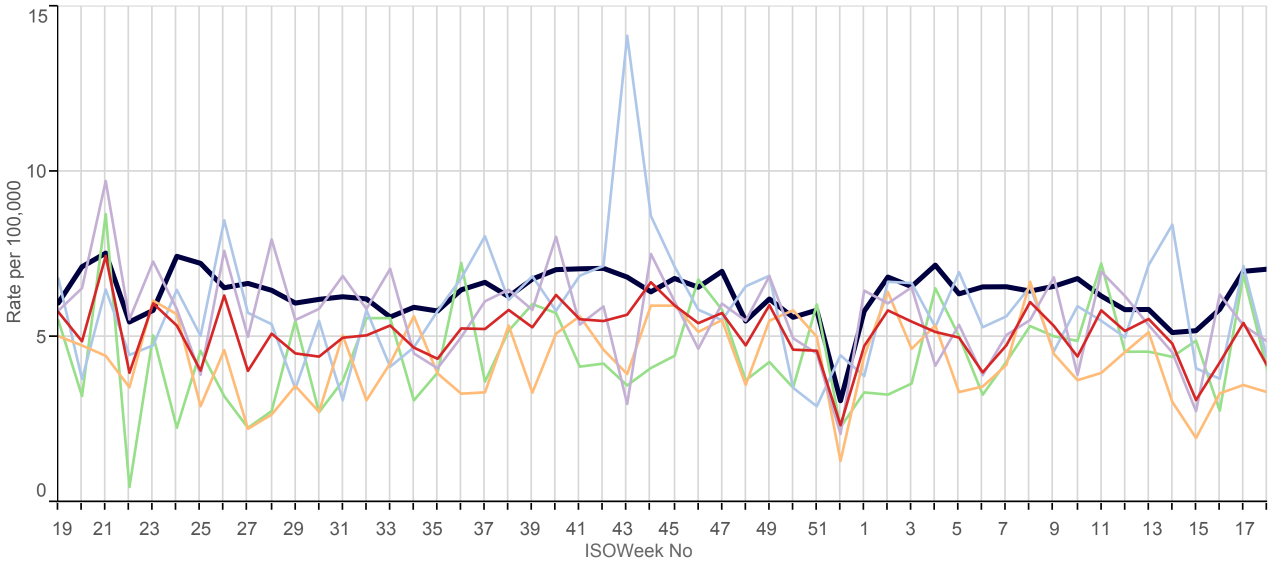

7. Genitourinary System Disorders:

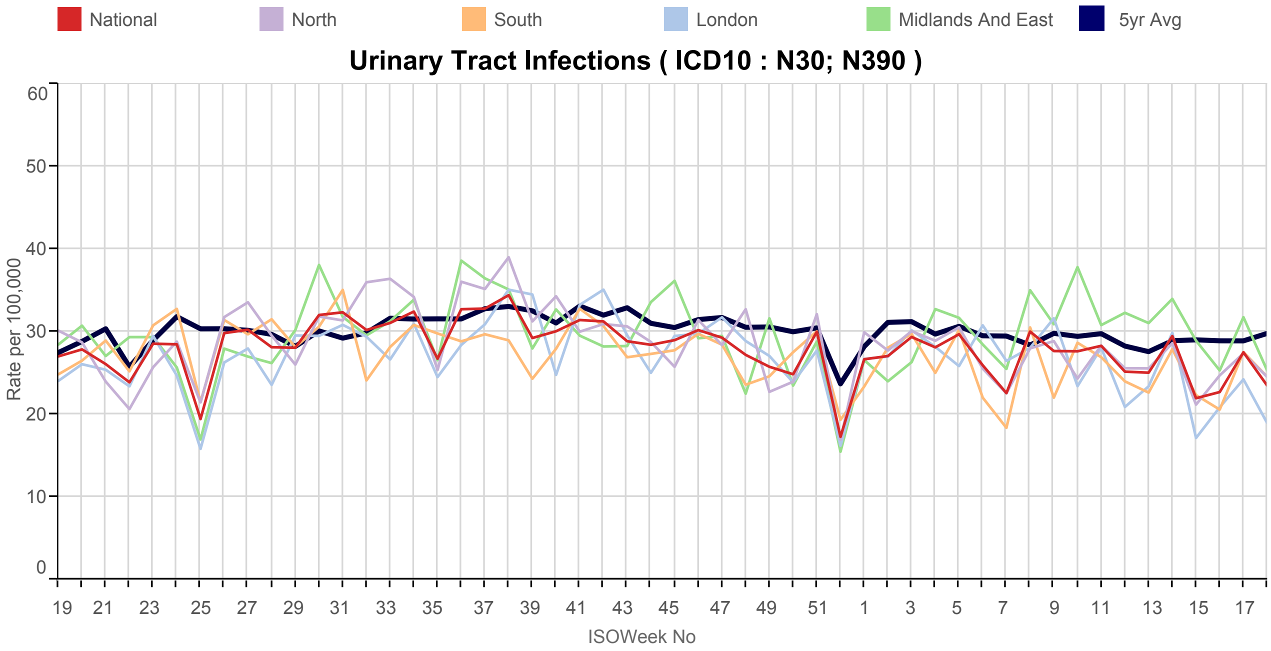

APPENDIX B: Demographic distribution by condition

1. Water and Food Borne Disorders:

Intestinal Infections (ICD10 : A00-A09)

Age-sex profile

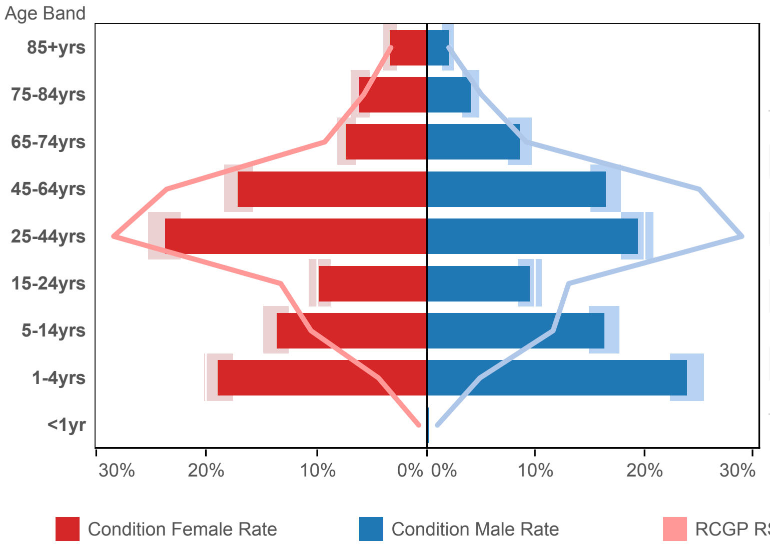

Age Band	Condition Female Rate	Condition Male Rate	Condition Female Population	Condition Male Population
**<1yr**	0.00	0.00	0.0	0.0
**1-4yrs**	18.93	23.92	610.0	652.0
**5-14yrs**	13.69	16.29	441.0	444.0
**15-24yrs**	9.78	9.50	315.0	259.0
**25-44yrs**	23.81	19.37	767.0	528.0
**45-64yrs**	17.10	16.43	551.0	448.0
**65-74yrs**	7.29	8.51	235.0	232.0
**75-84yrs**	6.05	4.04	195.0	110.0
**85+yrs**	3.35	1.94	108.0	53.0

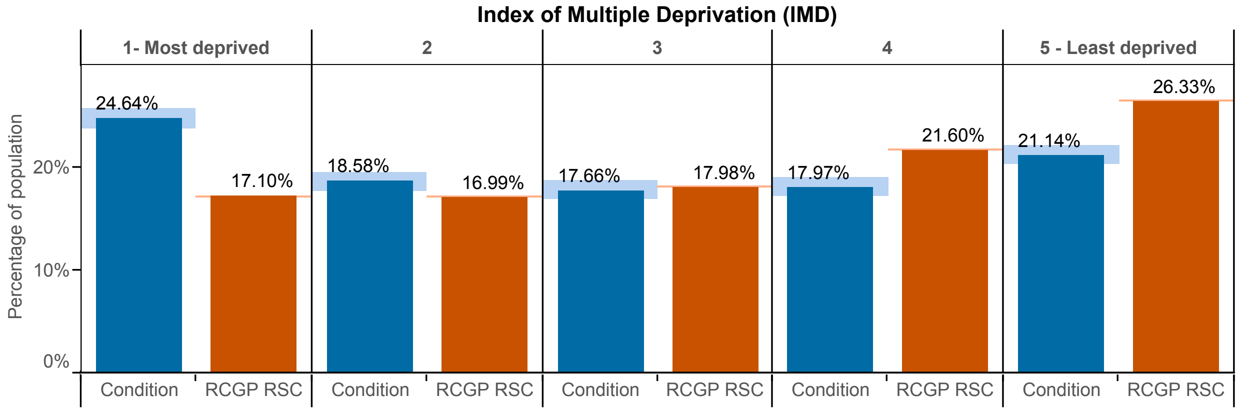

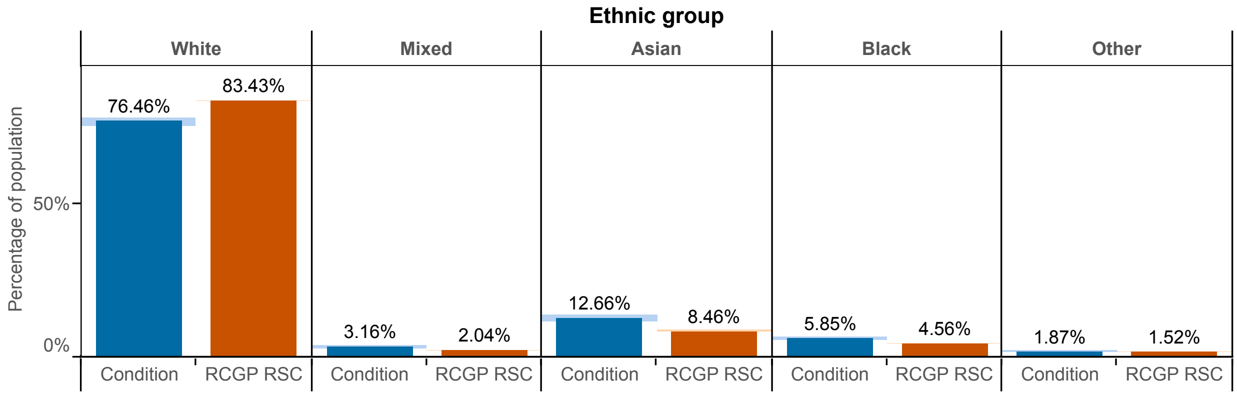

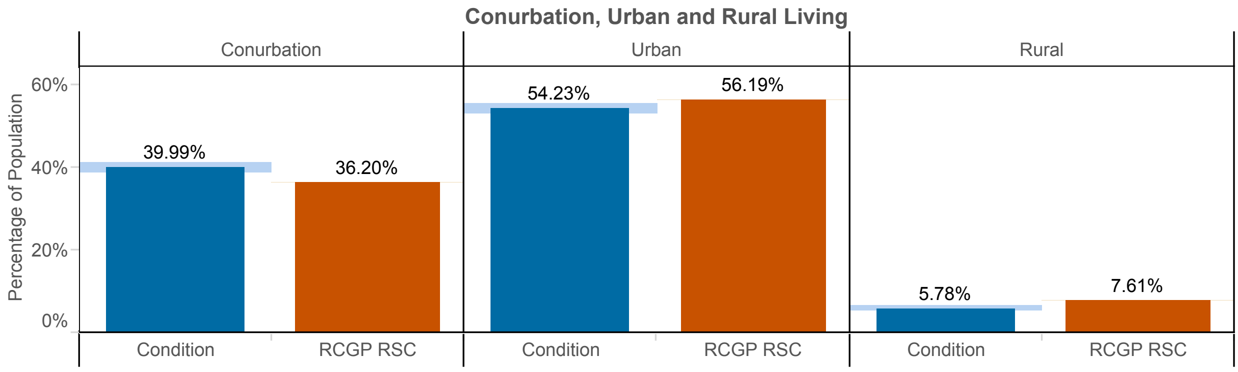

Non-infective Enteritis / Colitis (ICD10 : K50-K52)

Age-sex profile

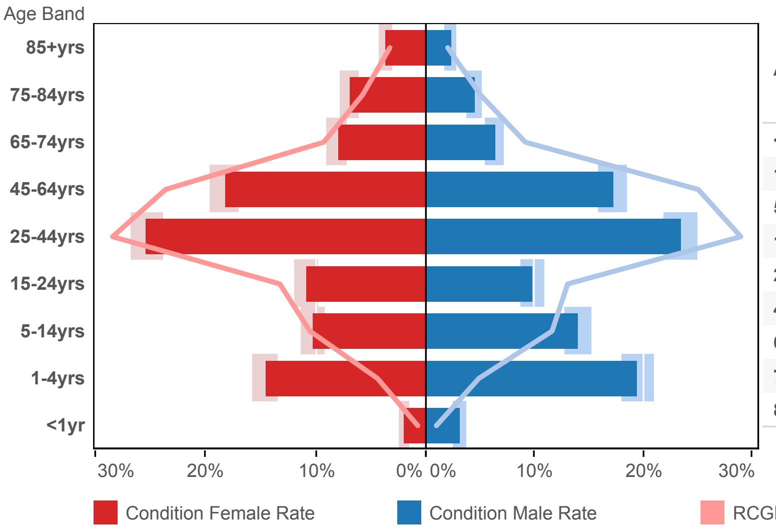

Age Band	Condition Female Rate	Condition Male Rate	Condition Female Population	Condition Male Population
<1yr	1.95	3.07	67.0	90.0
1-4yrs	14.60	19.47	501.0	570.0
5-14yrs	10.26	14.00	352.0	410.0
15-24yrs	10.90	9.73	374.0	285.0
25-44yrs	25.38	23.50	871.0	688.0
45-64yrs	18.30	17.18	628.0	503.0
65-74yrs	8.04	6.32	276.0	185.0
75-84yrs	6.91	4.44	237.0	130.0
85+yrs	3.67	2.29	126.0	67.0

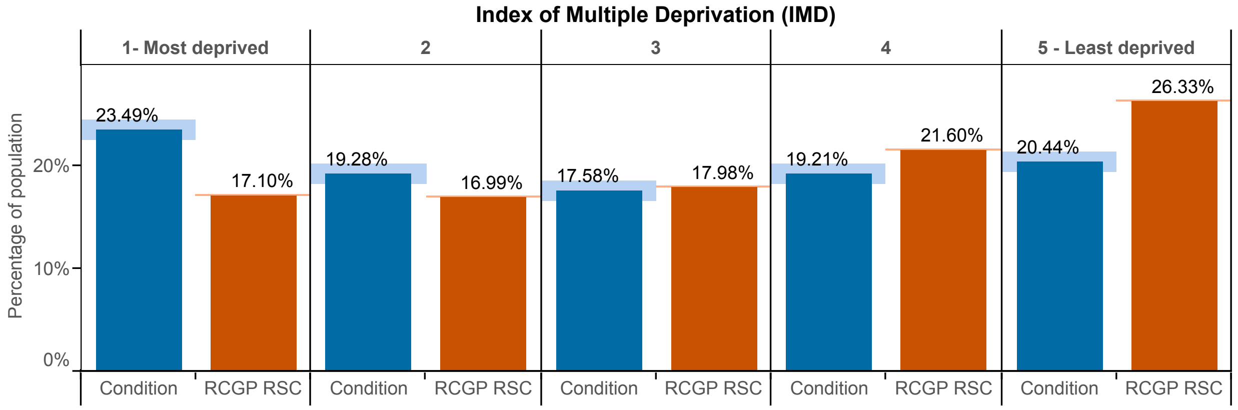

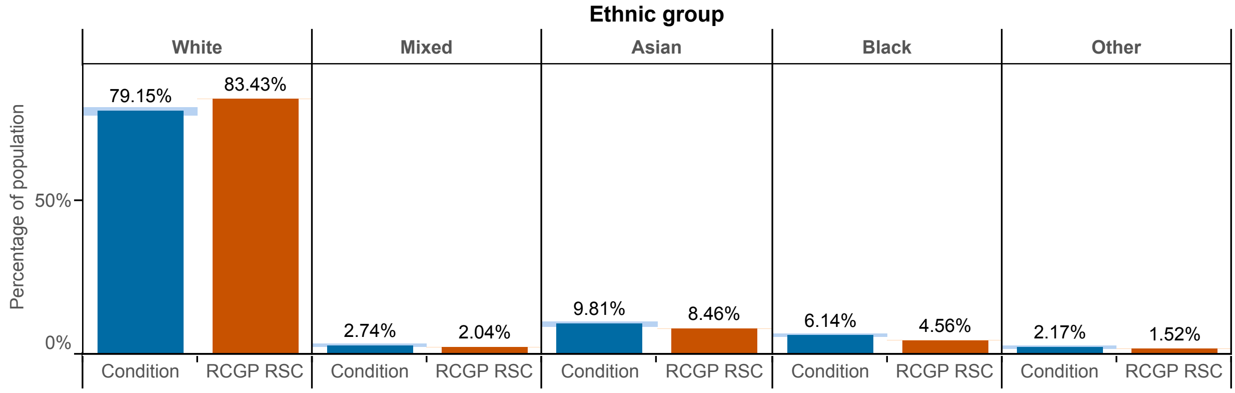

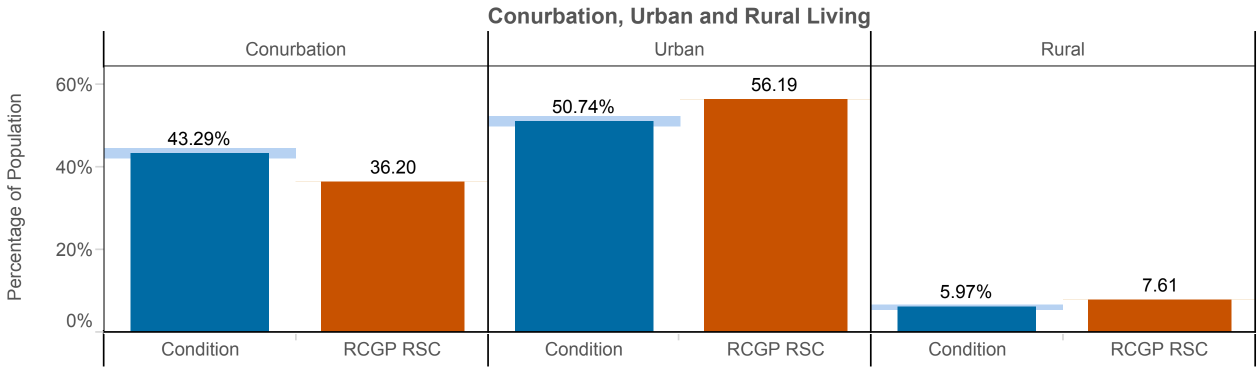

Viral Hepatitis (ICD10 : B15-B19)

Age-sex profile

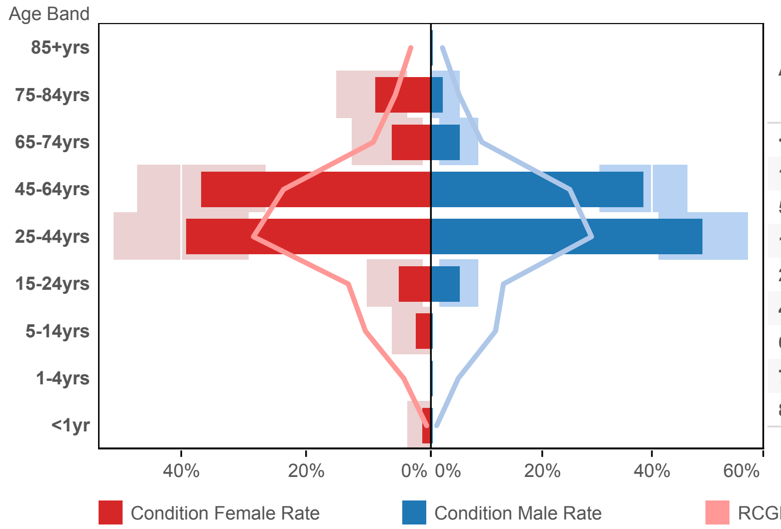

Age Band	Condition Female Rate	Condition Male Rate	Condition Female Population	Condition Male Population
<1yr	1.27	0.00	1.00	0.00
1-4yrs	0.00	0.00	0.00	0.00
5-14yrs	2.53	0.00	2.00	0.00
15-24yrs	5.06	5.07	4.00	7.00
25-44yrs	39.24	49.28	31.00	68.00
45-64yrs	36.71	38.41	29.00	53.00
65-74yrs	6.33	5.07	5.00	7.00
75-84yrs	8.86	2.17	7.00	3.00
85+yrs	0.00	0.00	0.00	0.00

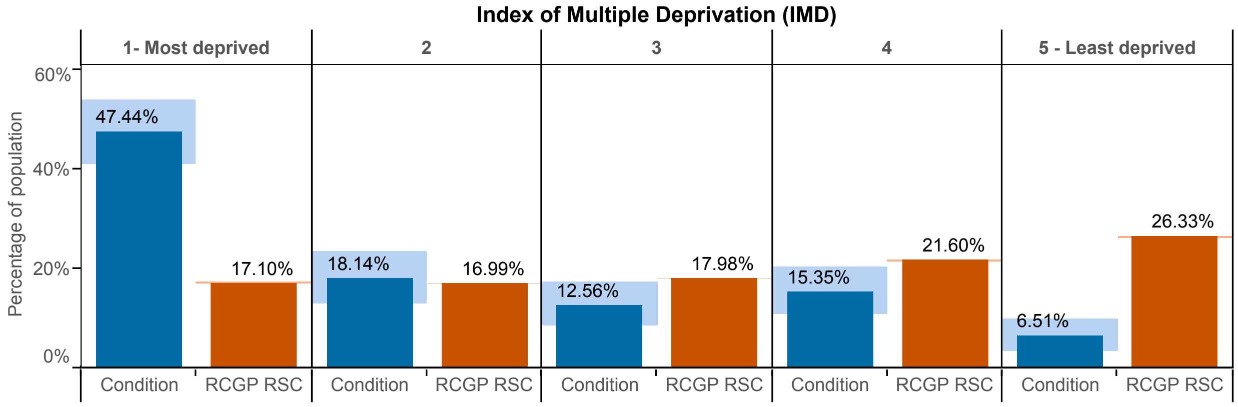

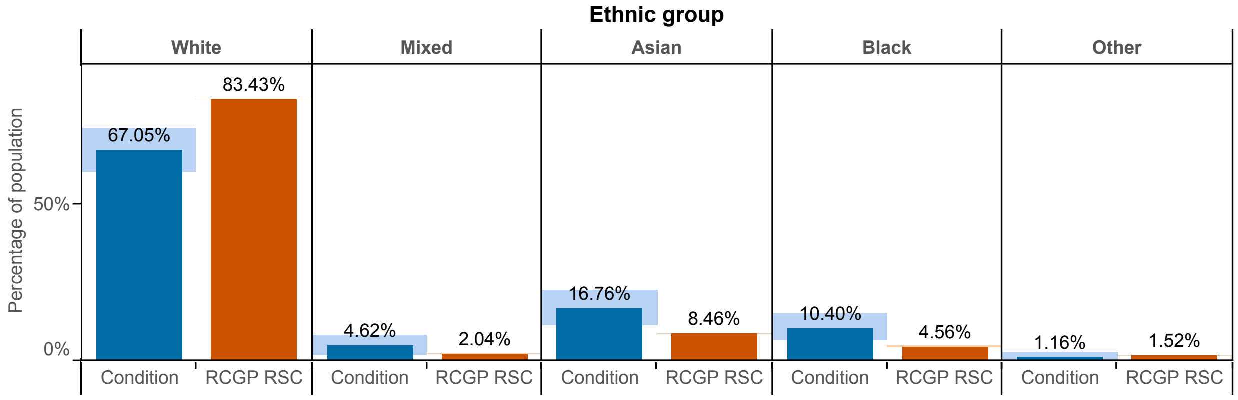

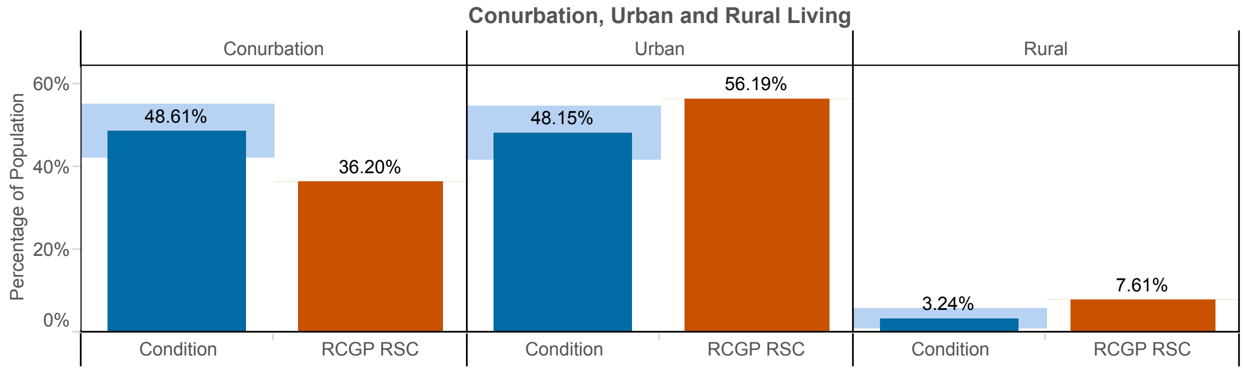

2. Environmentally Sensitive Disorders:

Asthma (ICD10 : J45 - J46)

Age-sex profile

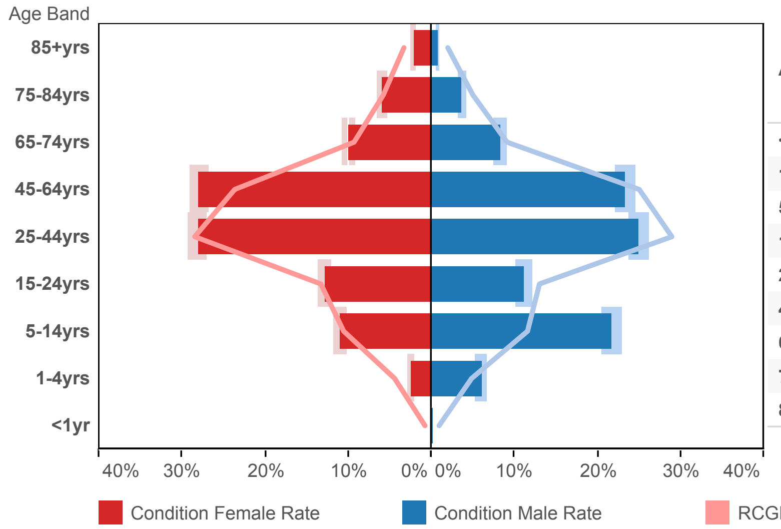

Age Band	Condition Female Rate	Condition Male Rate	Condition Female Population	Condition Male Population
<1yr	0.00	0.02	0	1
1-4yrs	2.41	6.05	146	263
5-14yrs	10.96	21.65	664	941
15-24yrs	12.77	11.16	774	485
25-44yrs	28.07	24.97	1,701	1,085
45-64yrs	27.94	23.38	1,693	1,016
65-74yrs	9.90	8.24	600	358
75-84yrs	5.86	3.73	355	162
85+yrs	2.10	0.81	127	35

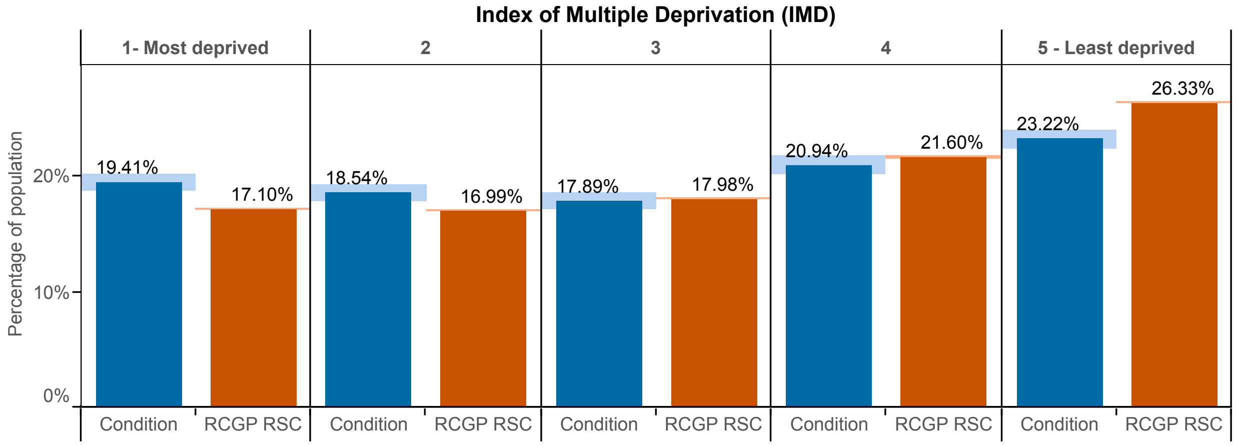

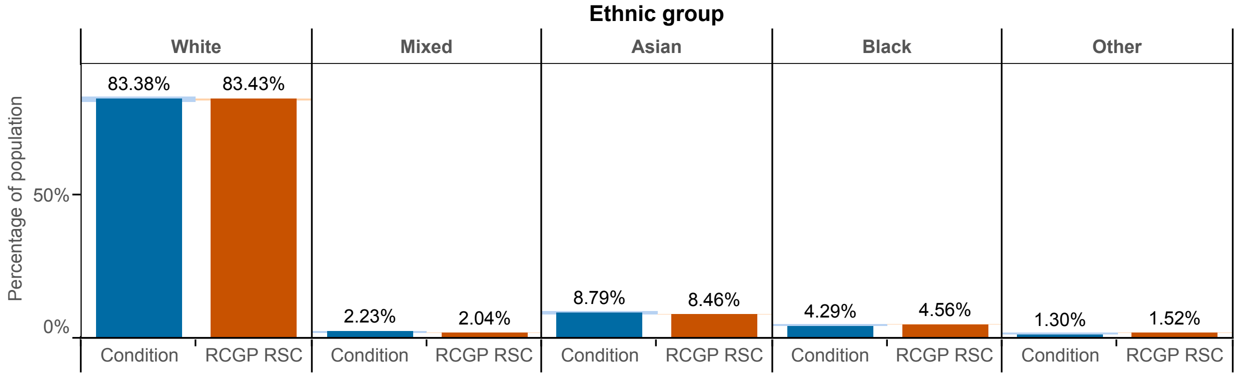

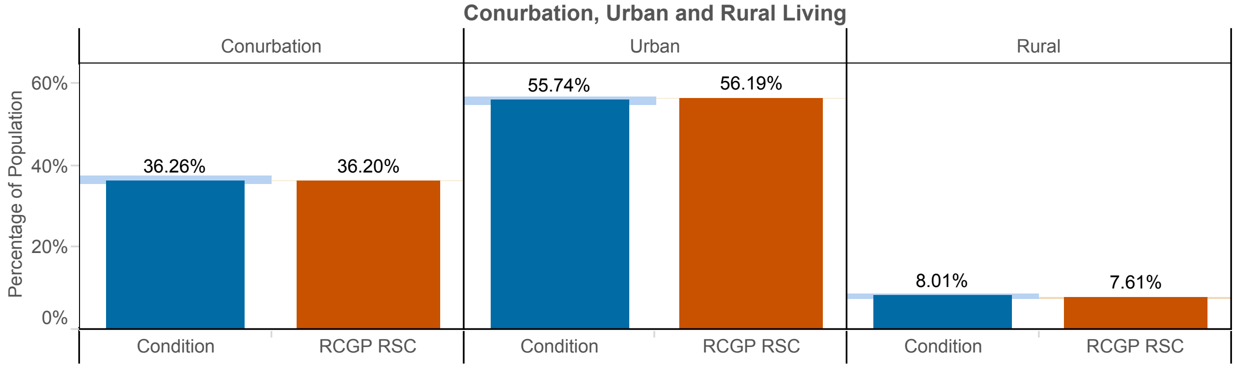

Conjunctivitis (ICD10 : H10 - H13)

Age-sex profile

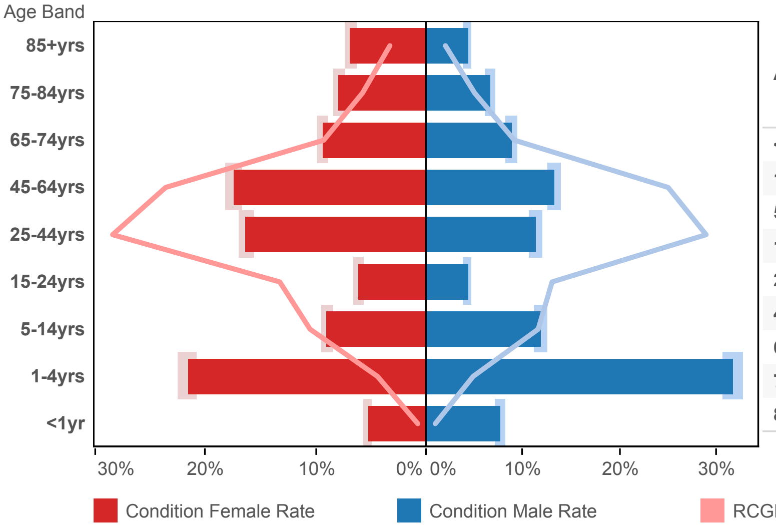

Age Band	Condition Female Rate	Condition Male Rate	Condition Female Population	Condition Male Population
**<1yr**	5.26	7.68	549	642
**1-4yrs**	21.66	31.74	2,262	2,653
**5-14yrs**	9.00	11.80	940	986
**15-24yrs**	6.13	4.35	640	364
**25-44yrs**	16.35	11.35	1,708	949
**45-64yrs**	17.47	13.28	1,825	1,110
**65-74yrs**	9.37	8.88	979	742
**75-84yrs**	7.92	6.57	827	549
**85+yrs**	6.84	4.35	714	364

Index of Multiple Deprivation (IMD)

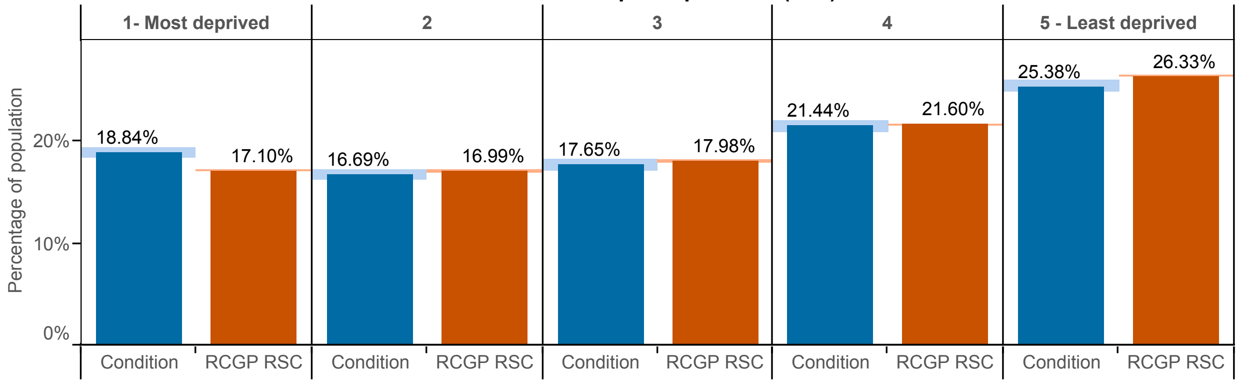

Ethnic group

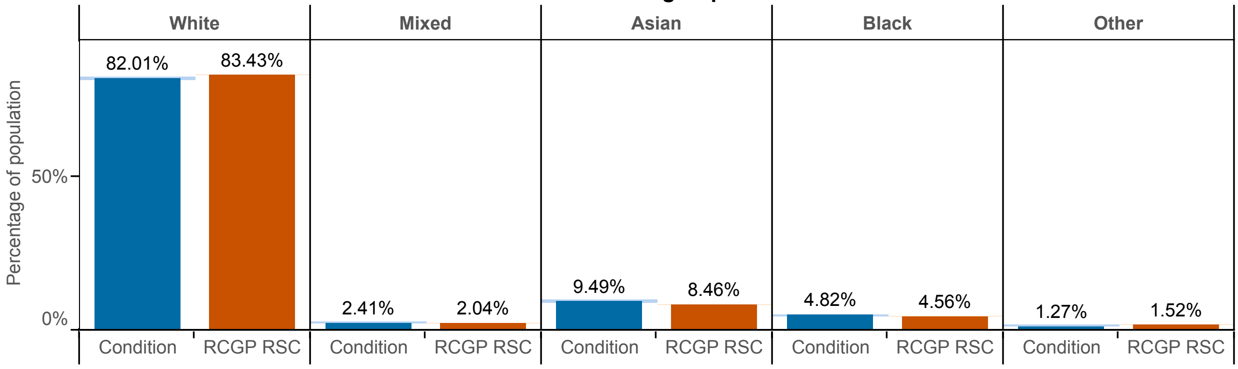

Conurbation, Urban and Rural Living

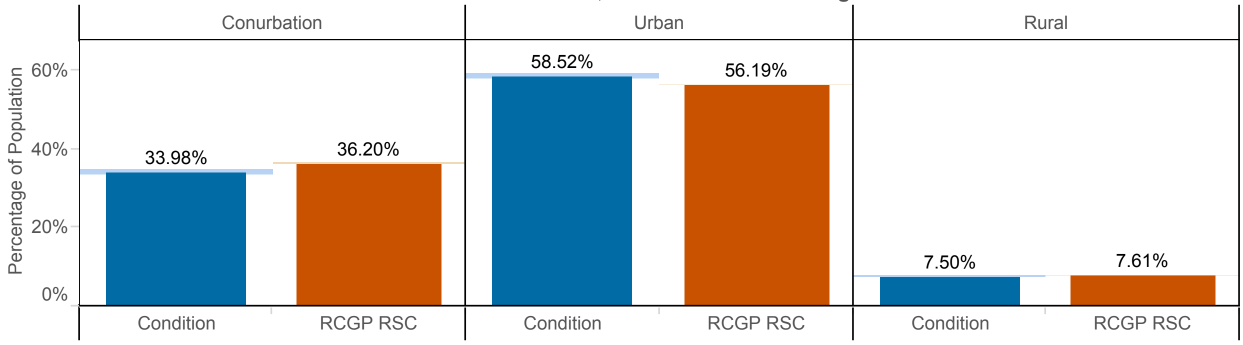

Hayfever/Allergic Rhinitis (ICD10: J30)

Age-sex profile

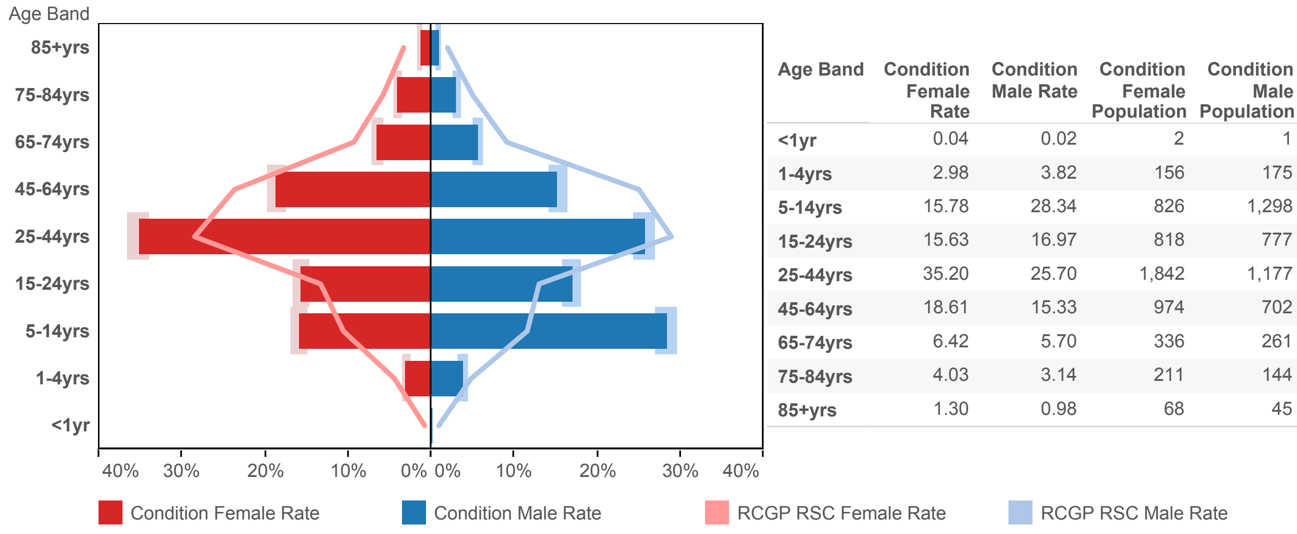

Age Band	Condition Female Rate	Condition Male Rate	Condition Female Population	Condition Male Population
<1yr	0.04	0.02	2	1
1-4yrs	2.98	3.82	156	175
5-14yrs	15.78	28.34	826	1,298
15-24yrs	15.63	16.97	818	777
25-44yrs	35.20	25.70	1,842	1,177
45-64yrs	18.61	15.33	974	702
65-74yrs	6.42	5.70	336	261
75-84yrs	4.03	3.14	211	144
85+yrs	1.30	0.98	68	45

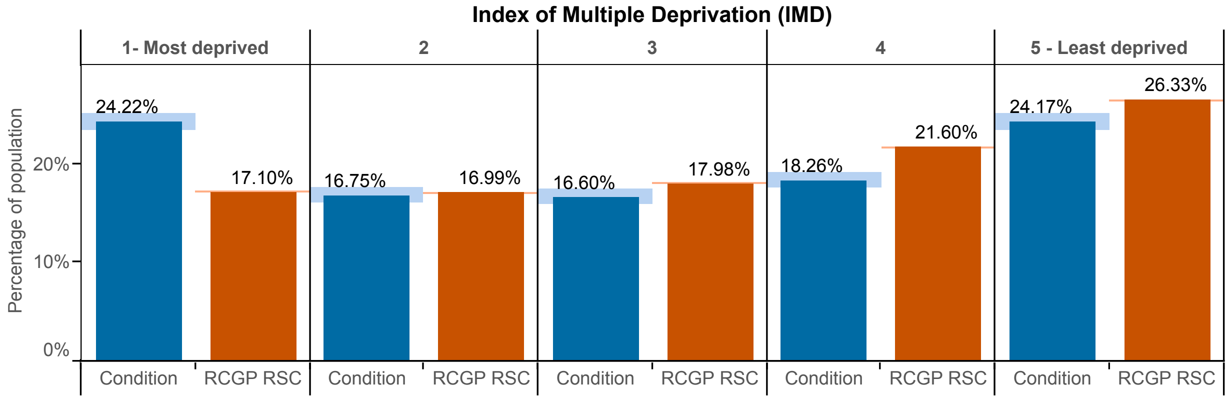

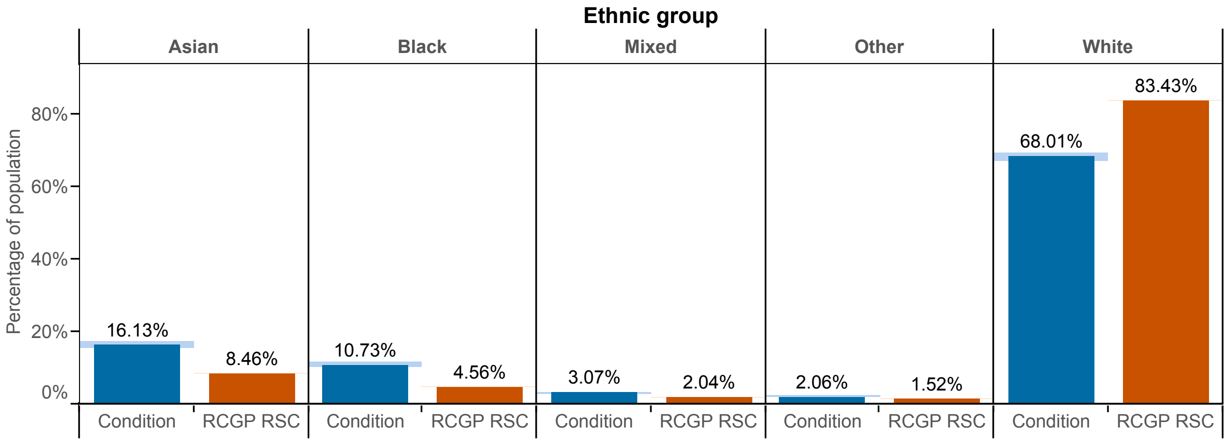

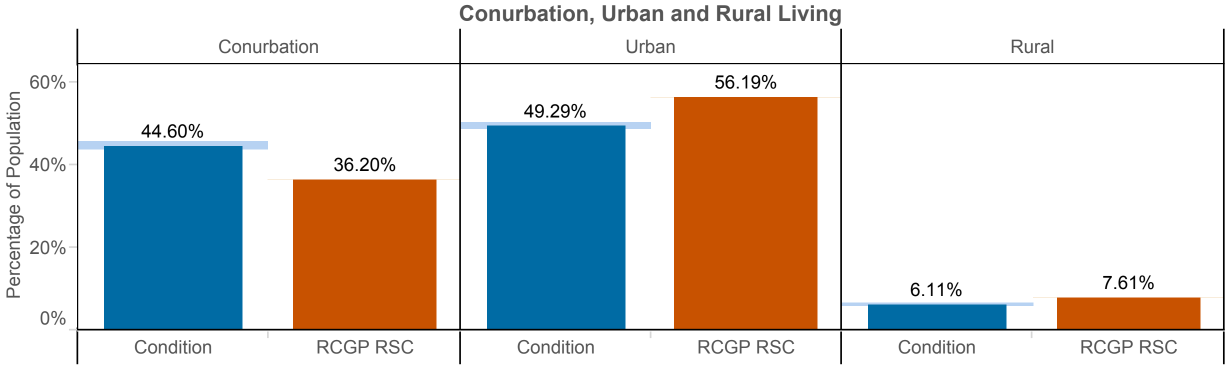

Respiratory / chest symptoms (ICD10 : R05 - R07; R09)

Age-sex profile

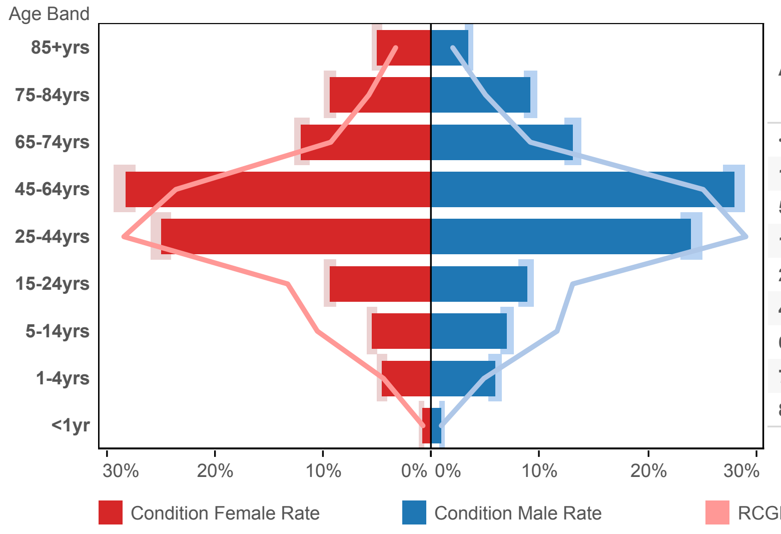

Age Band	Condition Female Rate	Condition Male Rate	Condition Female P opulation	Condition Male Pop ulation
**<1yr**	0.86	0.96	67	67
**1-4yrs**	4.59	5.90	358	411
**5-14yrs**	5.44	6.96	425	485
**15-24yrs**	9.39	8.78	733	612
**25-44yrs**	24.97	23.91	1,950	1,667
**45-64yrs**	28.33	27.87	2,212	1,943
**65-74yrs**	11.96	13.02	934	908
**75-84yrs**	9.41	9.15	735	638
**85+yrs**	5.05	3.46	394	241

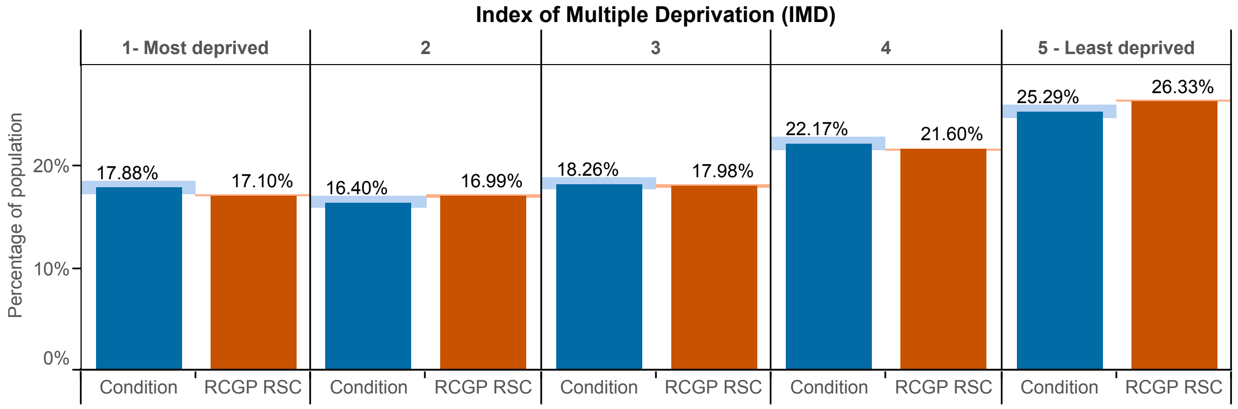

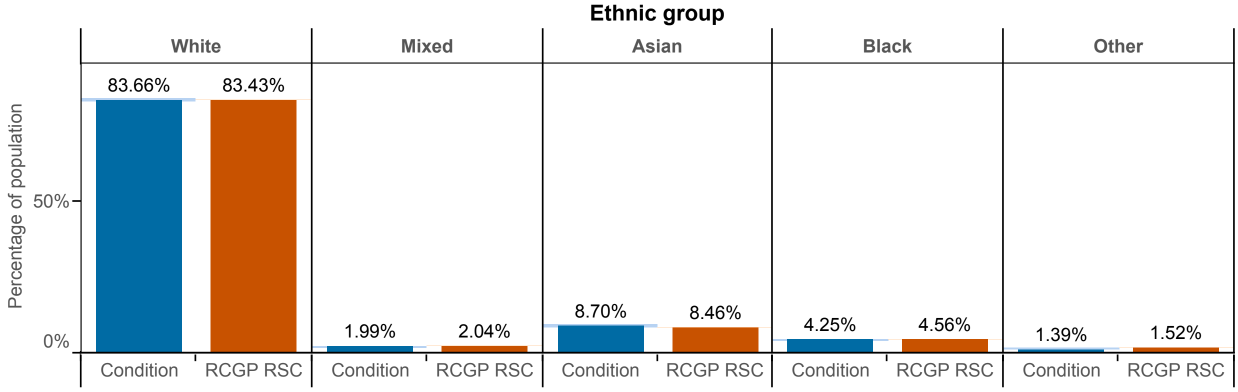

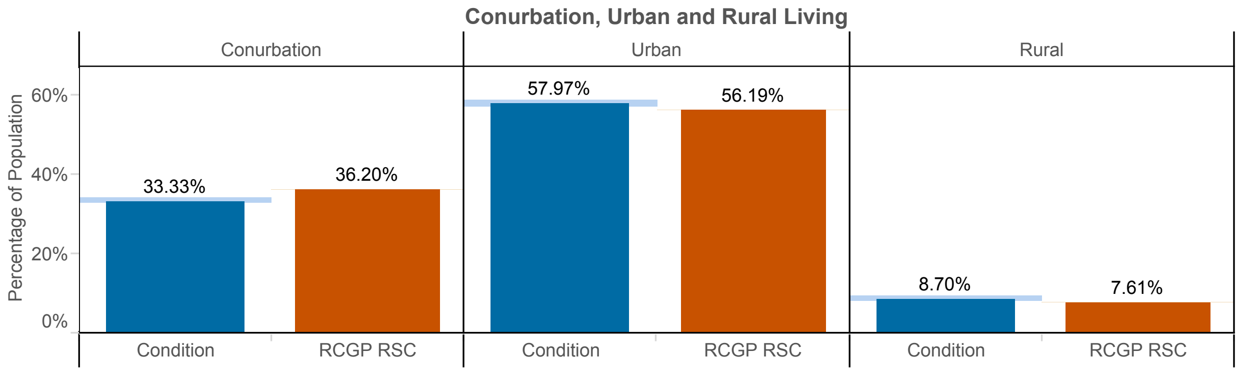

3. Respiratory Infections:

Bronchitis (ICD10: J20-J21,J40)

Age-sex profile

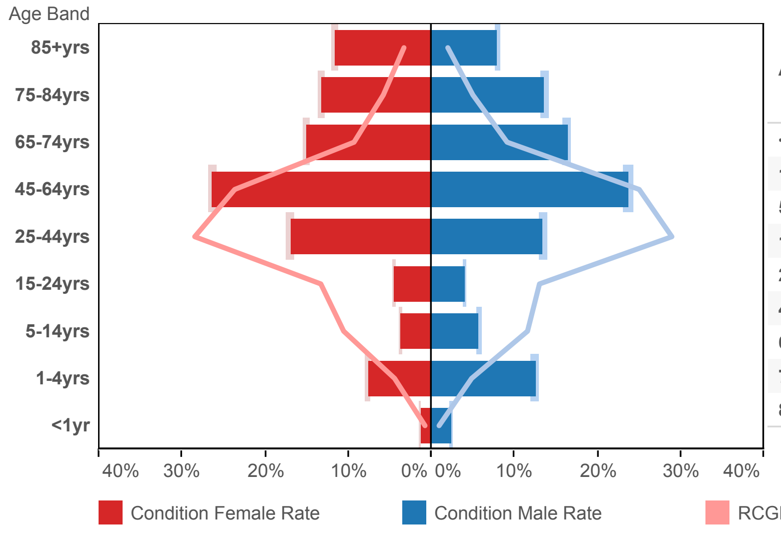

Age Band	Condition Female Rate	Condition Male Rate	Condition Female Population	Condition Male Population
<1yr	1.28	2.43	338	496
1-4yrs	7.53	12.50	1,983	2,549
5-14yrs	3.66	5.73	963	1,168
15-24yrs	4.48	4.05	1,180	827
25-44yrs	16.92	13.49	4,453	2,751
45-64yrs	26.37	23.69	6,941	4,832
65-74yrs	15.03	16.43	3,955	3,352
75-84yrs	13.22	13.66	3,480	2,786
85+yrs	11.51	8.02	3,029	1,635

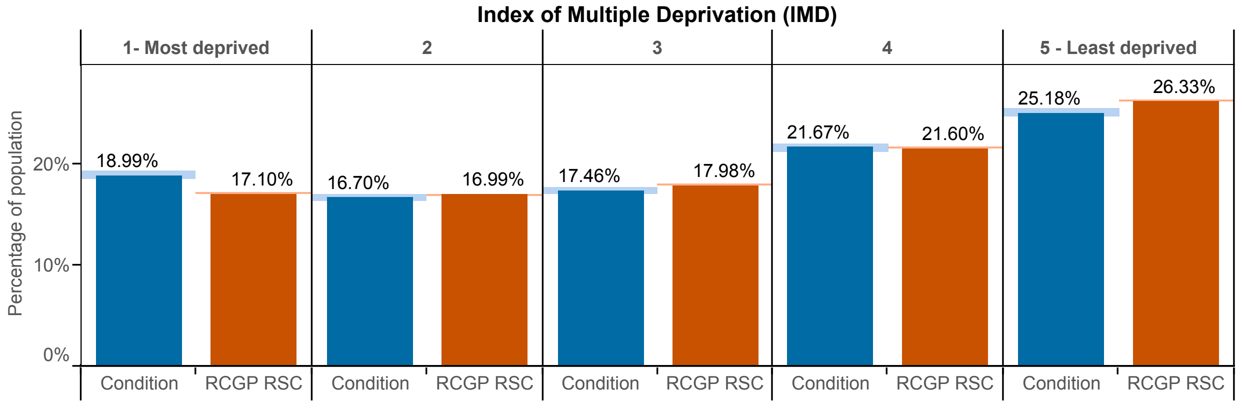

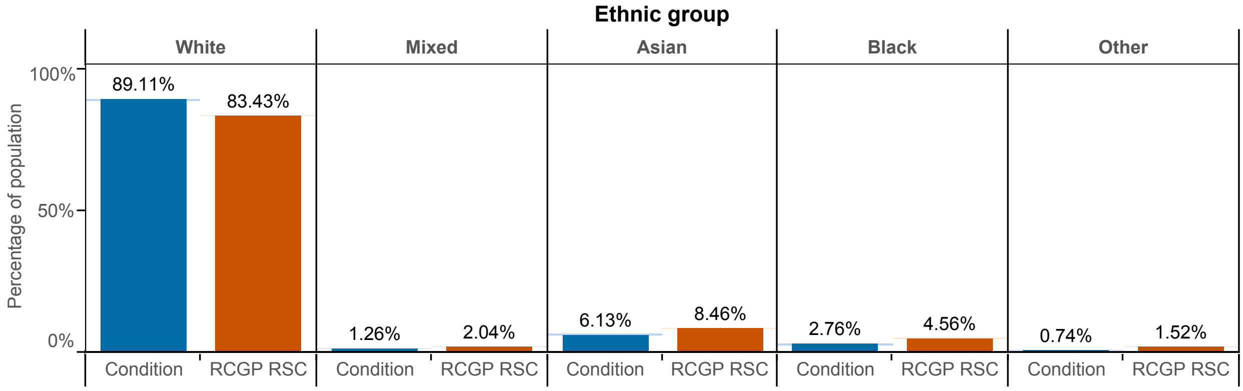

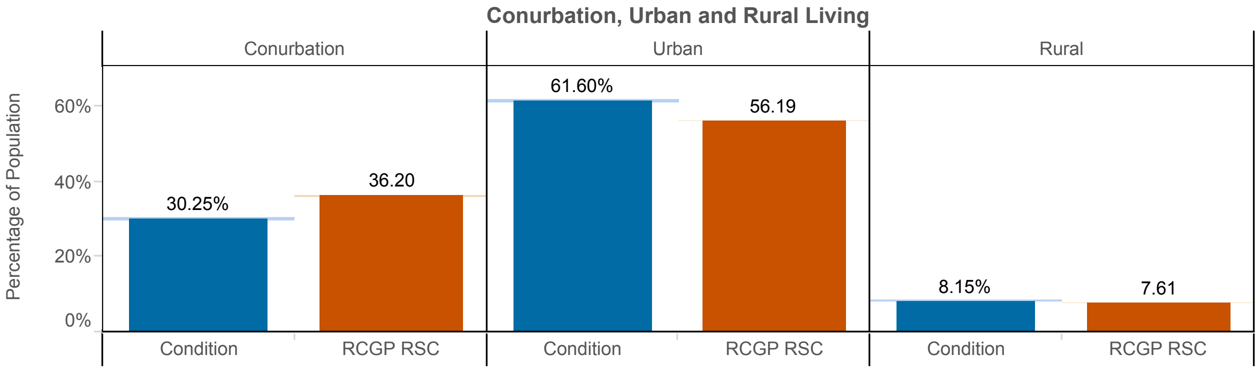

Common Cold (ICD10 : R05 - R07; R09)

Age-sex profile

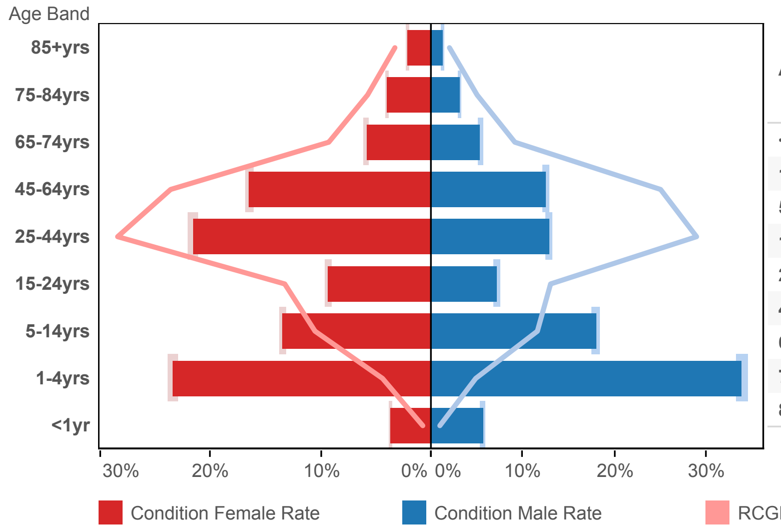

Age Band	Condition Female Rate	Condition Male Rate	Condition Female Population	Condition Male Population
<1yr	3.68	5.67	1,249	1,444
1-4yrs	23.46	33.96	7,958	8,646
5-14yrs	13.47	17.96	4,570	4,573
15-24yrs	9.28	7.16	3,148	1,823
25-44yrs	21.56	12.89	7,312	3,281
45-64yrs	16.50	12.48	5,596	3,178
65-74yrs	5.87	5.41	1,992	1,378
75-84yrs	3.99	3.19	1,355	811
85+yrs	2.19	1.28	742	326

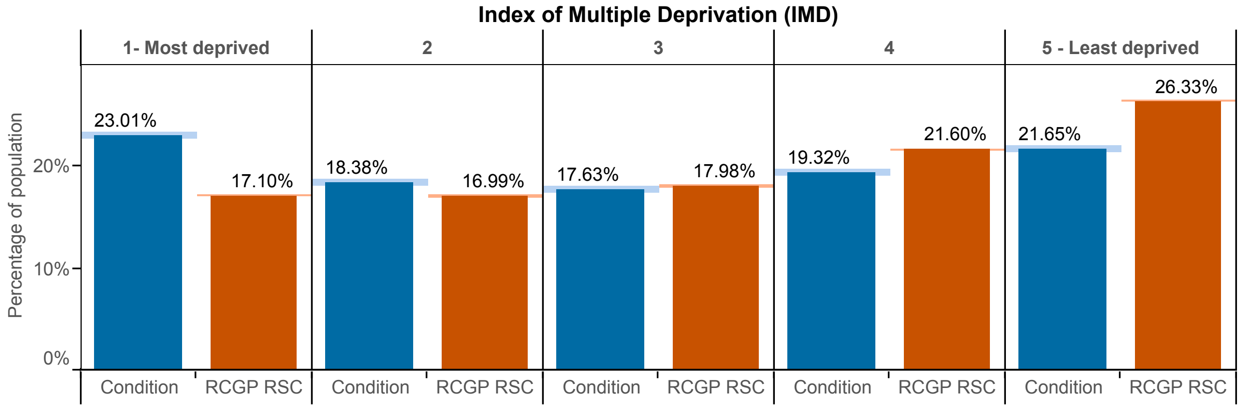

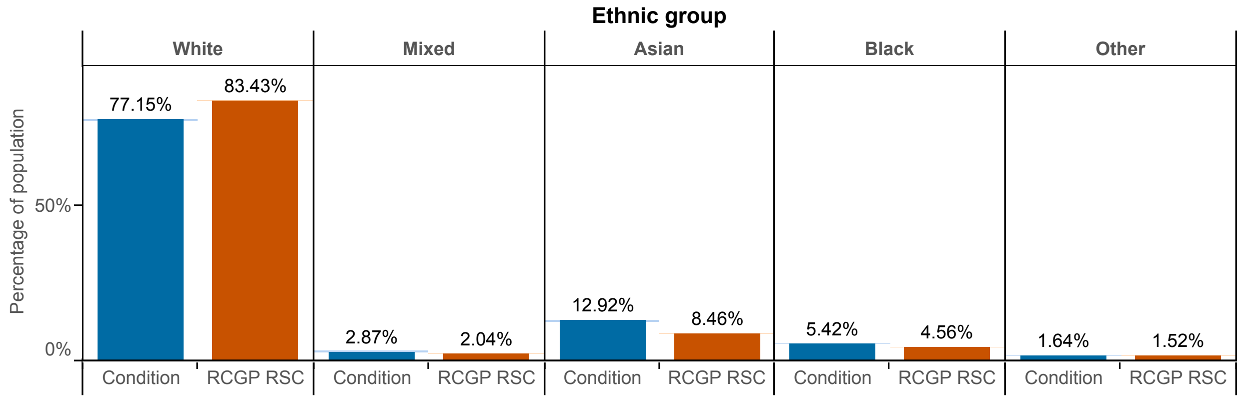

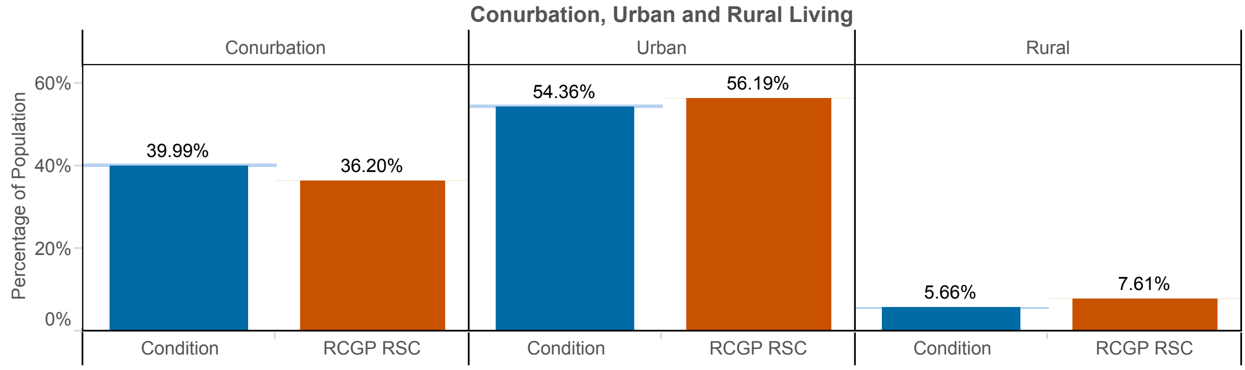

Influenza-like illness (ICD10 : J09 - J11)

Age-sex profile

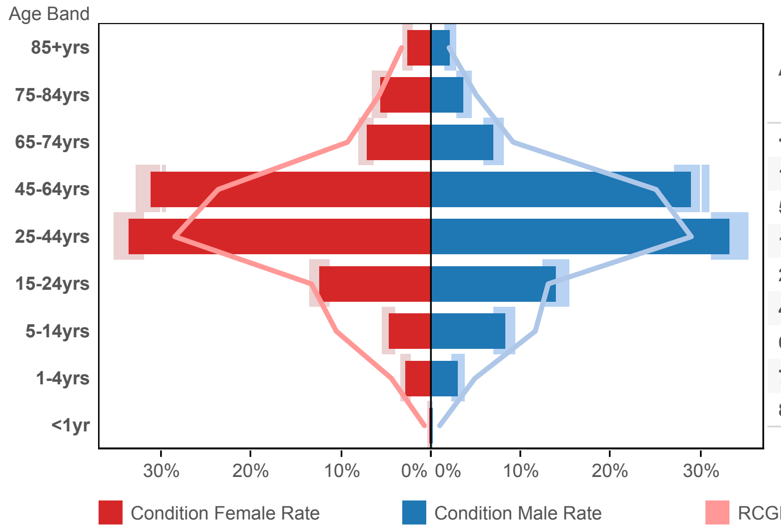

Age Band	Condition Female Rate	Condition Male Rate	Condition Female Population	Condition Male Population
<1yr	0.17	0.10	5.0	2.0
1-4yrs	2.85	2.99	84.0	60.0
5-14yrs	4.65	8.23	137.0	165.0
15-24yrs	12.38	13.86	365.0	278.0
25-44yrs	33.45	33.20	986.0	666.0
45-64yrs	31.11	29.01	917.0	582.0
65-74yrs	7.19	6.88	212.0	138.0
75-84yrs	5.63	3.64	166.0	73.0
85+yrs	2.58	2.09	76.0	42.0

Index of Multiple Deprivation (IMD)

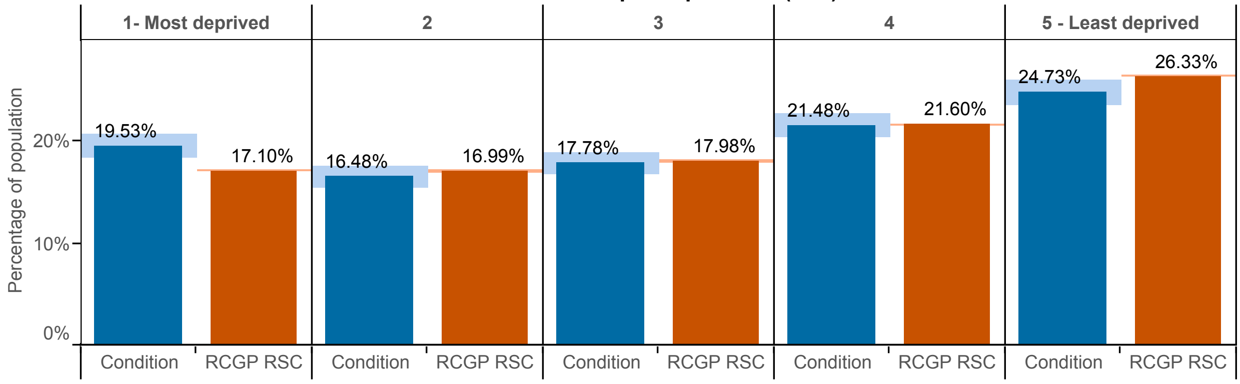

Ethnic group

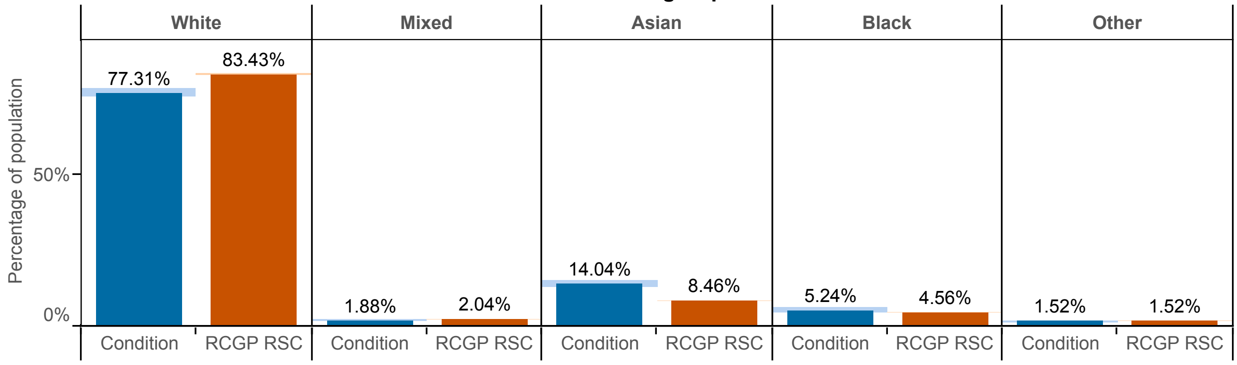

Conurbation, Urban and Rural Living

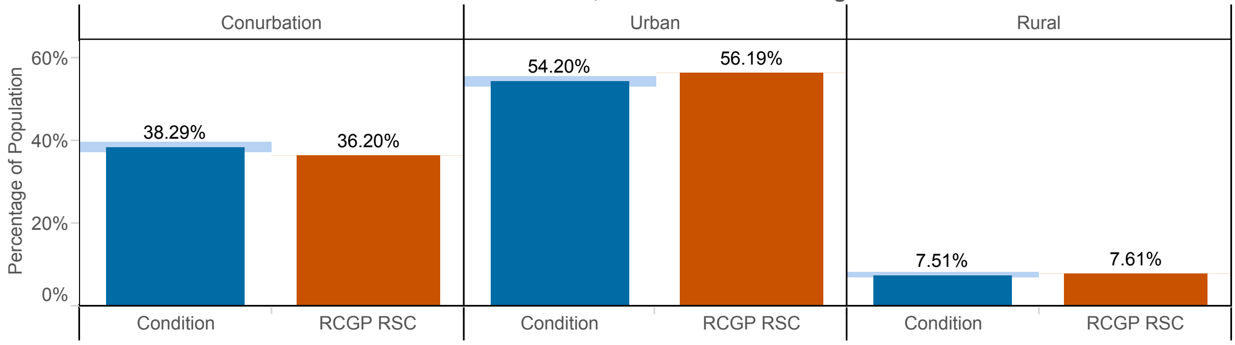

Laryngitis / Tracheitis (ICD10 : J04)

Age-sex profile

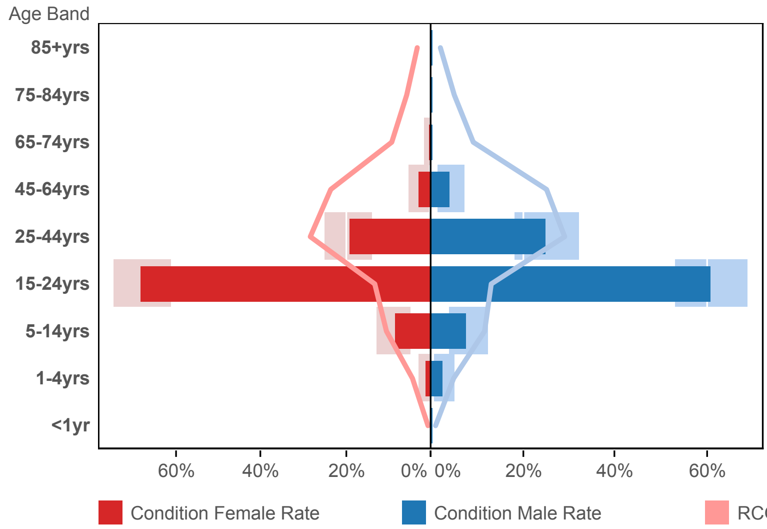

Age Band	Condition Female Rate	Condition Male Rate	Condition Female Population	Condition Male Population
<1yr	0.00	0.00	0.0	0.0
1-4yrs	1.06	2.61	2.0	4.0
5-14yrs	8.51	7.84	16.0	12.0
15-24yrs	68.09	60.78	128.0	93.0
25-44yrs	19.15	24.84	36.0	38.0
45-64yrs	2.66	3.92	5.0	6.0
65-74yrs	0.53	0.00	1.0	0.0
75-84yrs	0.00	0.00	0.0	0.0
85+yrs	0.00	0.00	0.0	0.0

Index of Multiple Deprivation (IMD)

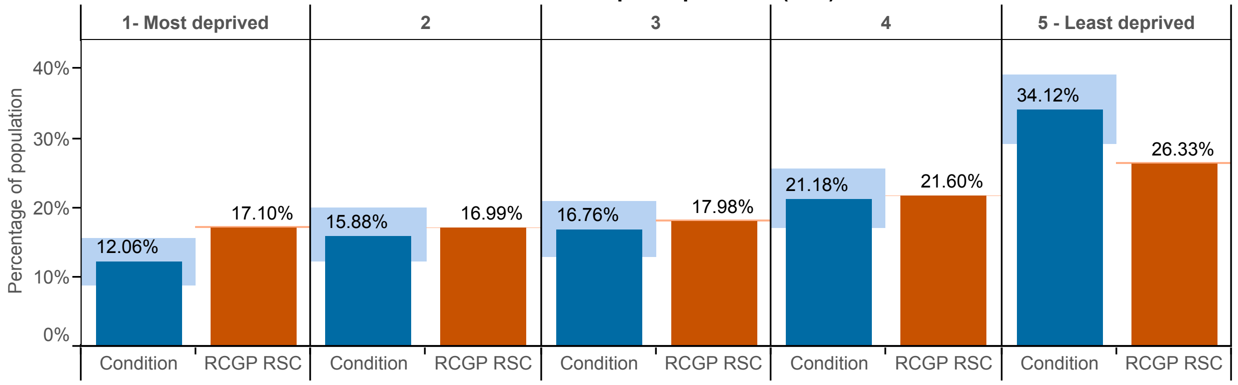

Ethnic group

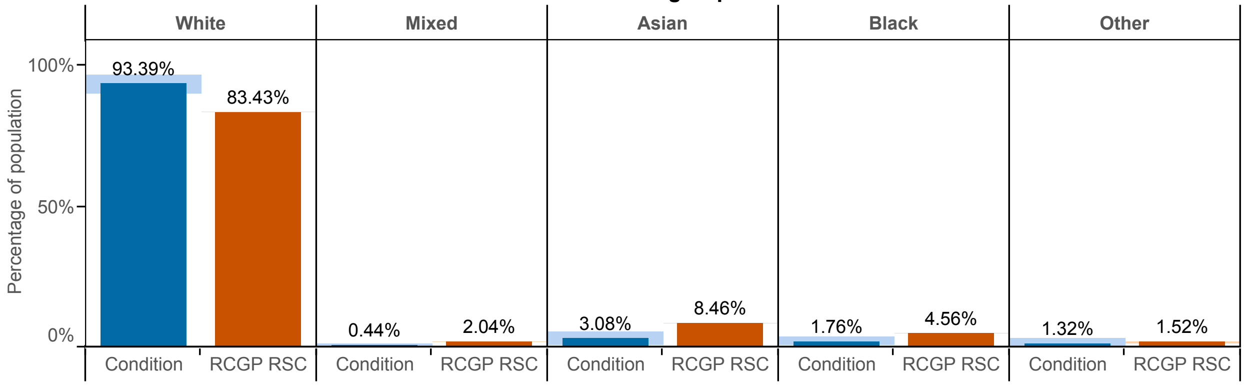

Conurbation, Urban and Rural Living

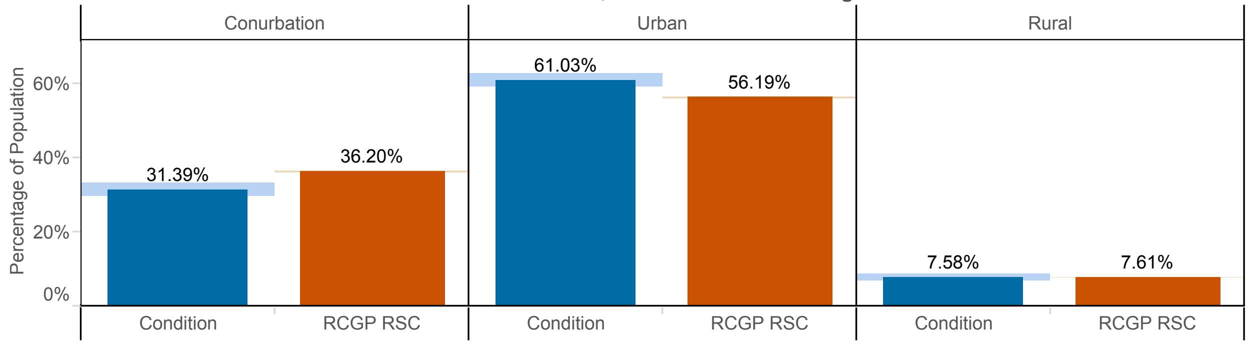

Pleurisy (ICD10 : R091)

Age-sex profile

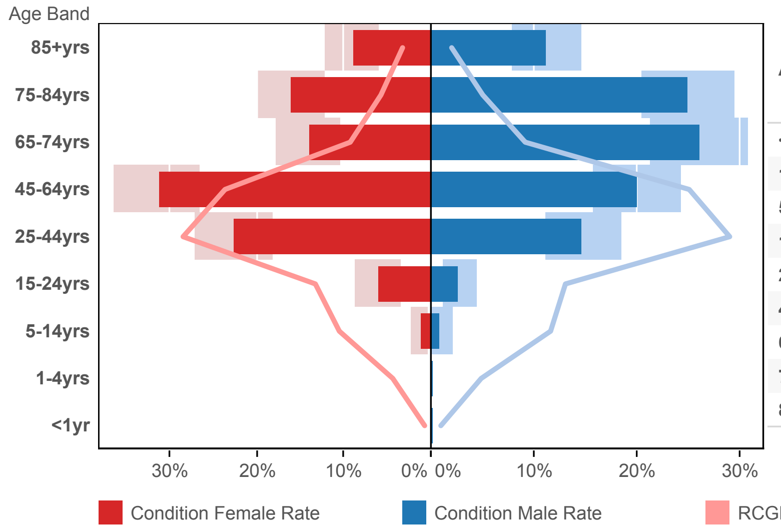

Age Band	Condition Female Rate	Condition Male Rate	Condition Female Population	Condition Male Population
<1yr	0.00	0.00	0.0	0.0
1-4yrs	0.00	0.00	0.0	0.0
5-14yrs	1.19	0.88	4.0	3.0
15-24yrs	5.95	2.63	20.0	9.0
25-44yrs	22.62	14.62	76.0	50.0
45-64yrs	31.25	19.88	105.0	68.0
65-74yrs	13.99	26.02	47.0	89.0
75-84yrs	16.07	24.85	54.0	85.0
85+yrs	8.93	11.11	30.0	38.0

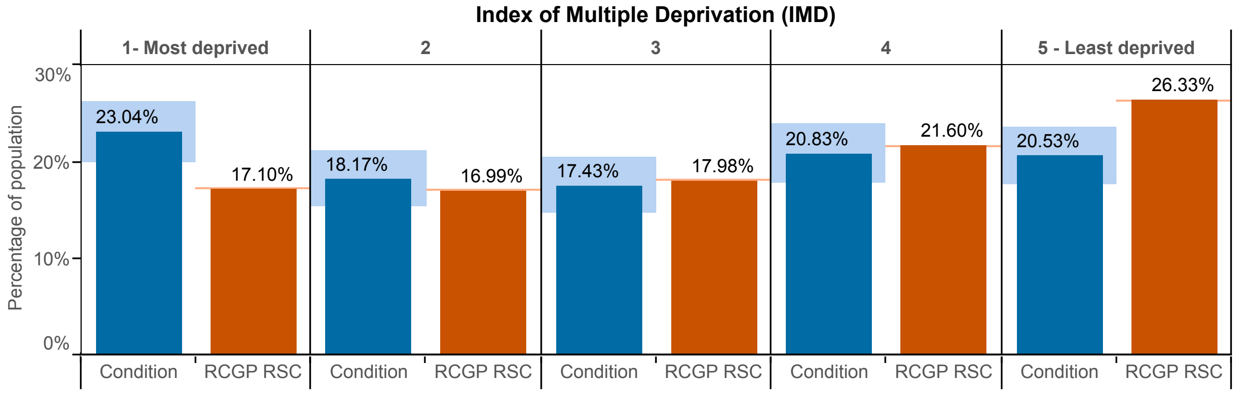

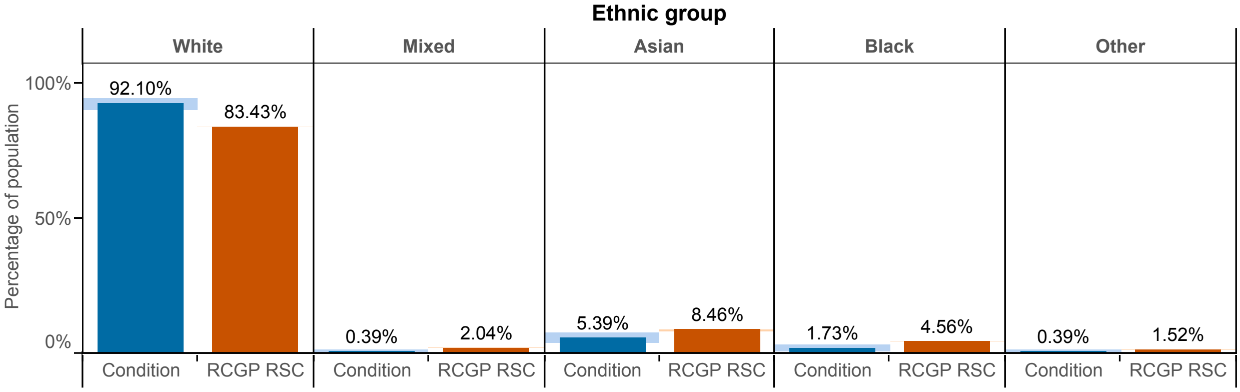

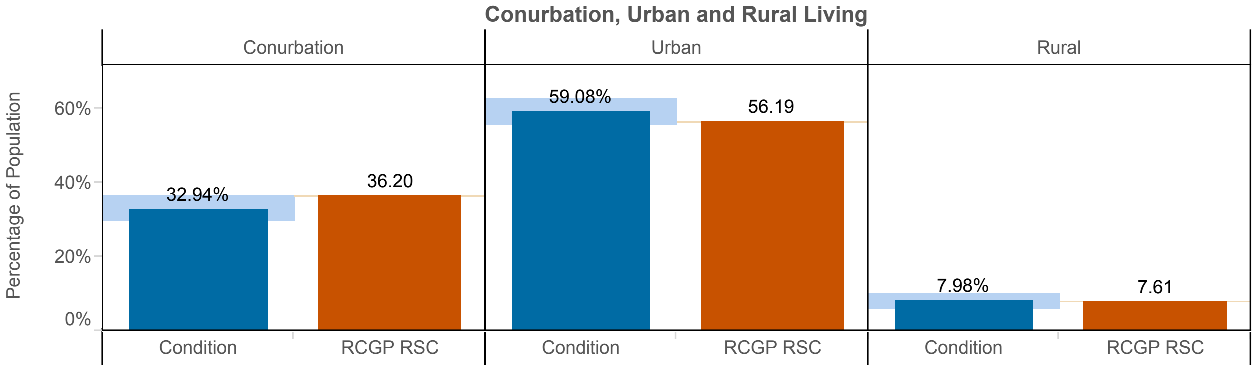

Pneumonia / Pneumonitis (ICD10 : J12 - J18)

Age-sex profile

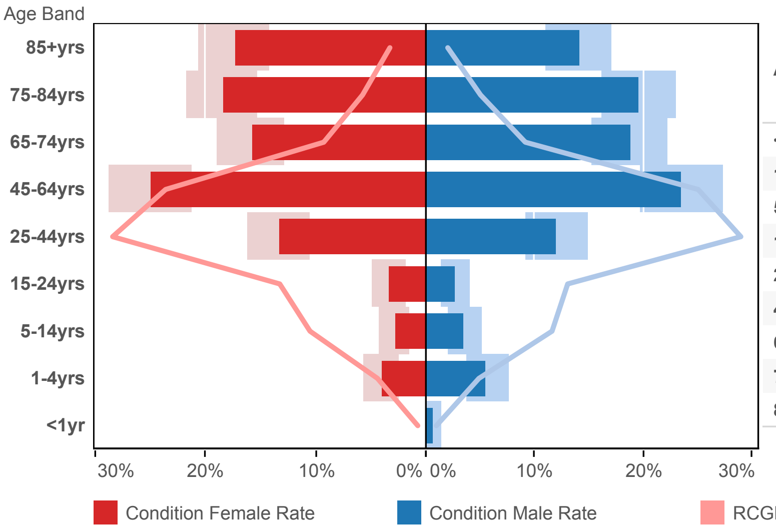

Age Band	Condition Female Rate	Condition Male Rate	Condition Female Population	Condition Male Population
<1yr	0.00	0.61	0.0	3.0
1-4yrs	4.05	5.50	22.0	27.0
5-14yrs	2.76	3.46	15.0	17.0
15-24yrs	3.31	2.65	18.0	13.0
25-44yrs	13.26	12.02	72.0	59.0
45-64yrs	25.05	23.42	136.0	115.0
65-74yrs	15.84	18.74	86.0	92.0
75-84yrs	18.42	19.55	100.0	96.0
85+yrs	17.31	14.05	94.0	69.0

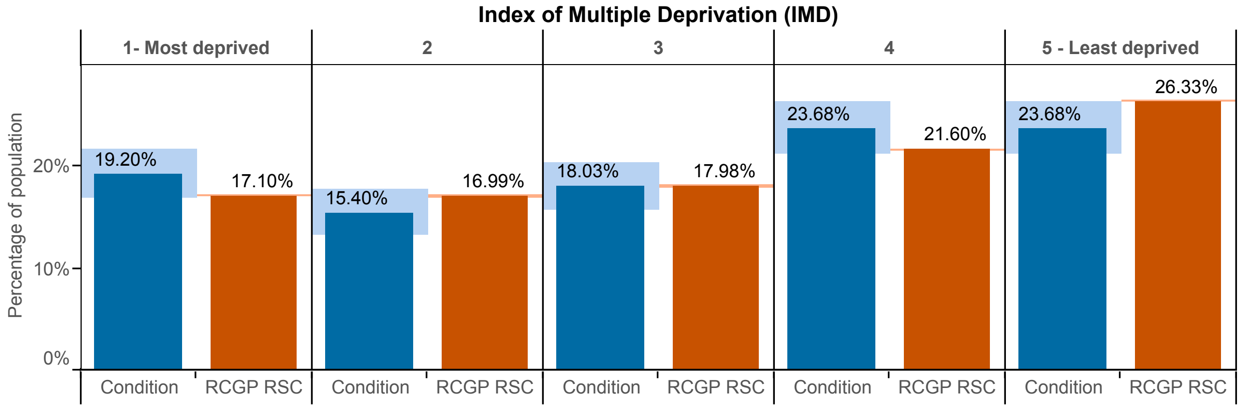

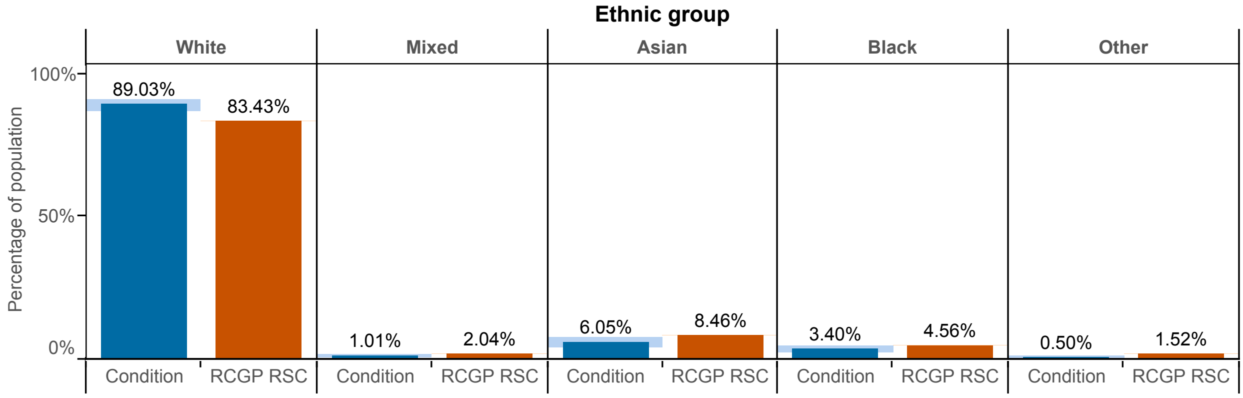

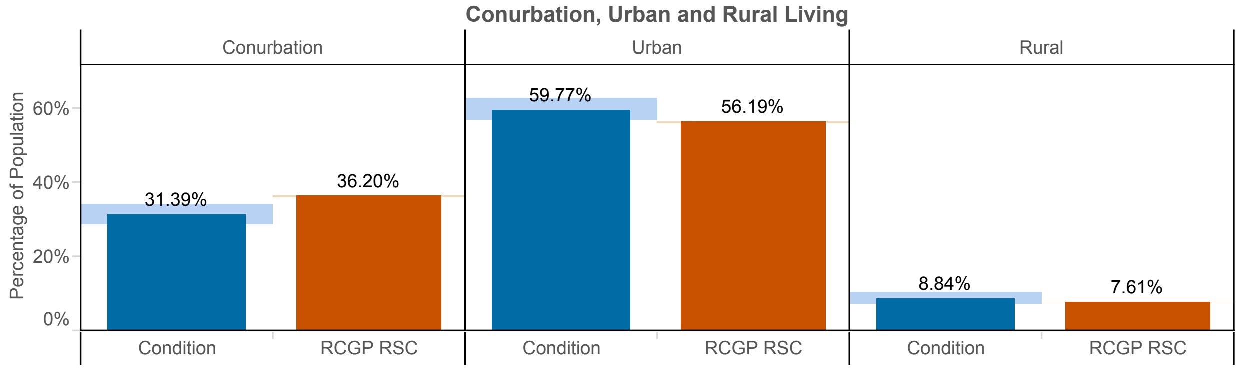

Respiratory disorders (ICD10 : J00-J99)

Age-sex profile

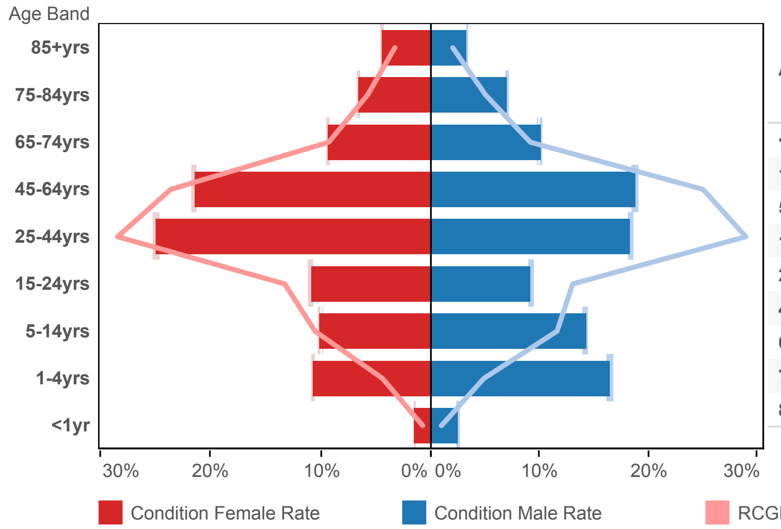

Age Band	Condition Female Rate	Condition Male Rate	Condition Female Population	Condition Male Population
<1yr	1.52	2.51	1,541	1,860
1-4yrs	10.71	16.47	10,843	12,210
5-14yrs	10.05	14.25	10,180	10,558
15-24yrs	10.92	9.21	11,053	6,829
25-44yrs	24.94	18.38	25,251	13,622
45-64yrs	21.52	18.78	21,795	13,922
65-74yrs	9.30	10.06	9,414	7,455
75-84yrs	6.61	7.02	6,693	5,203
85+yrs	4.43	3.31	4,488	2,454

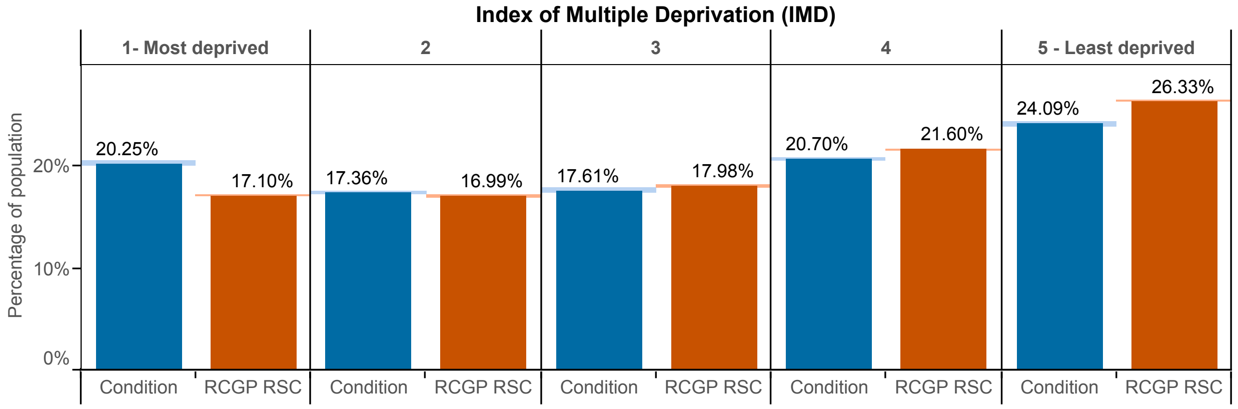

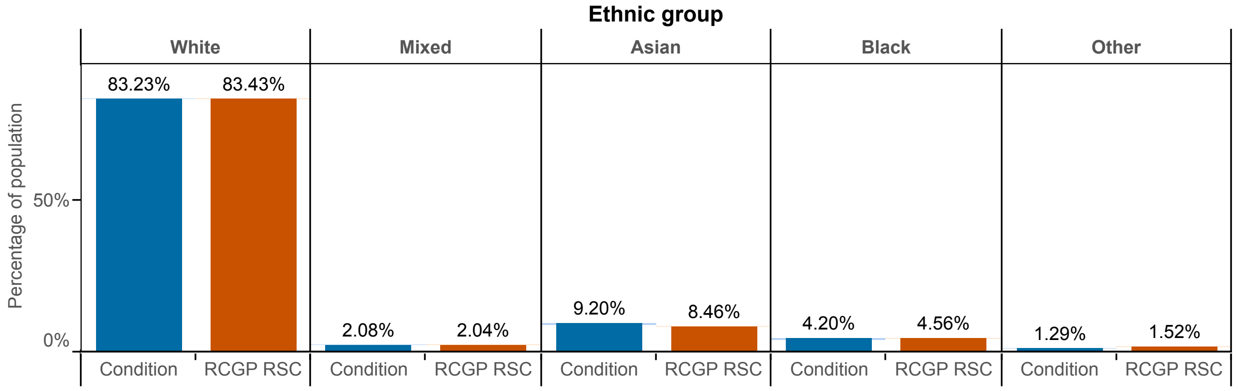

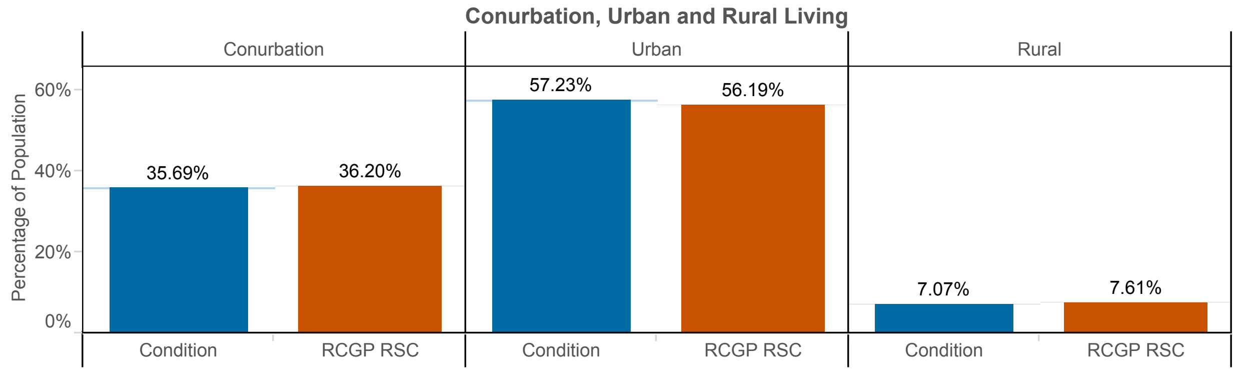

Acute Sinusitis (ICD10 : J01)

Age-sex profile

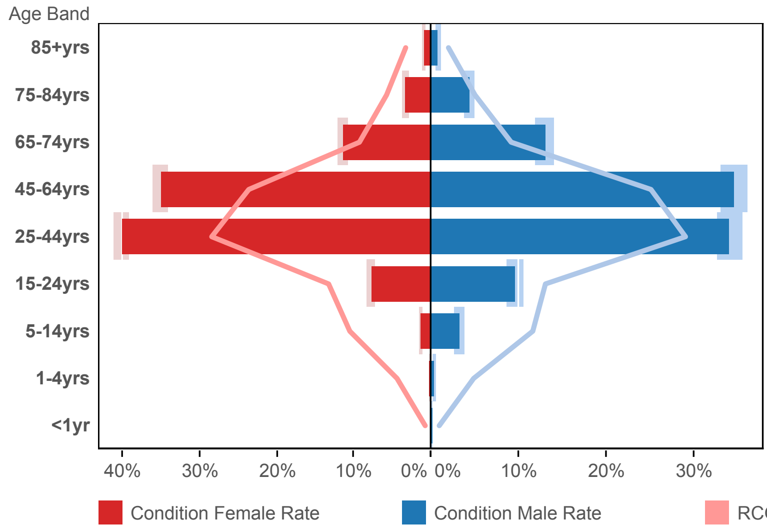

Age Band	Condition Female Rate	Condition Male Rate	Condition Female Population	Condition Male Population
**<1yr**	0.02	0.08	2	3
**1-4yrs**	0.17	0.41	17	16
**5-14yrs**	1.31	3.22	131	127
**15-24yrs**	7.73	9.54	772	376
**25-44yrs**	40.10	34.01	4,006	1,340
**45-64yrs**	35.12	34.49	3,508	1,359
**65-74yrs**	11.40	12.99	1,139	512
**75-84yrs**	3.28	4.39	328	173
**85+yrs**	0.87	0.86	87	34

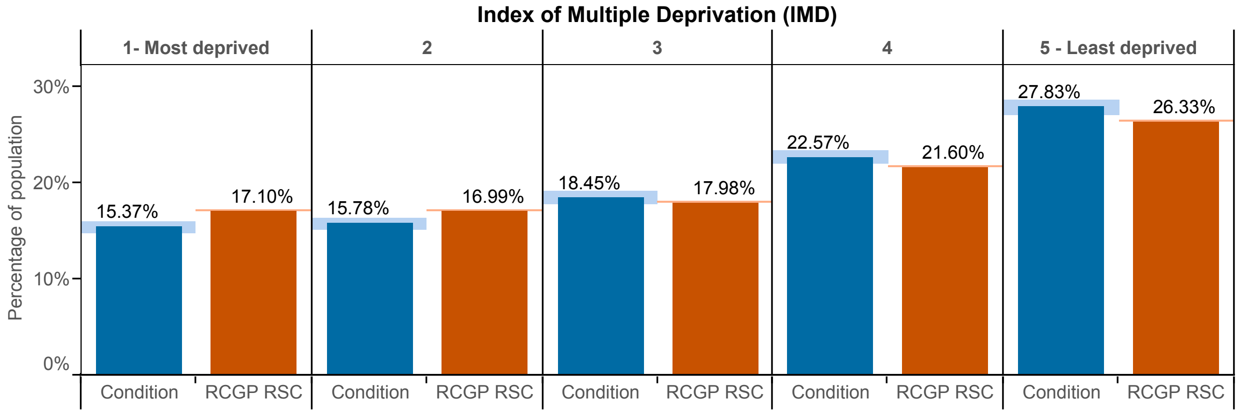

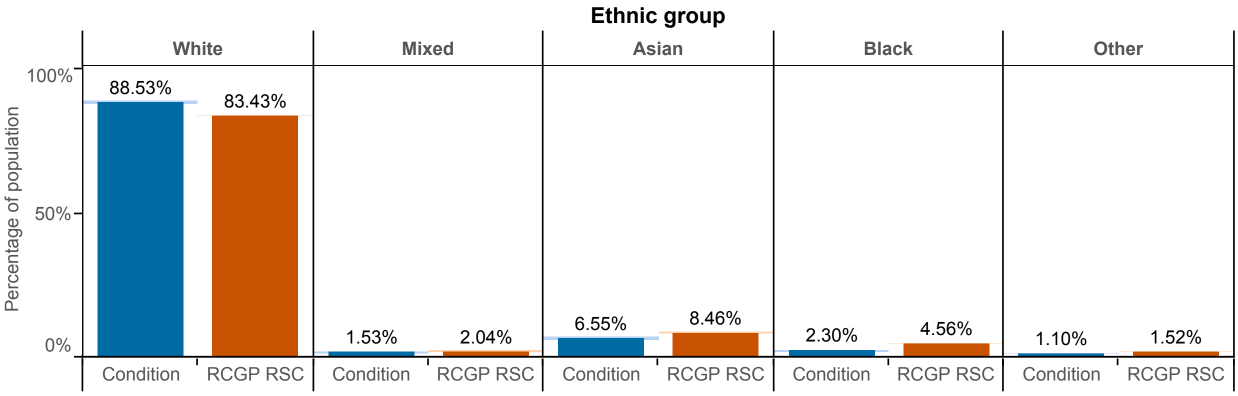

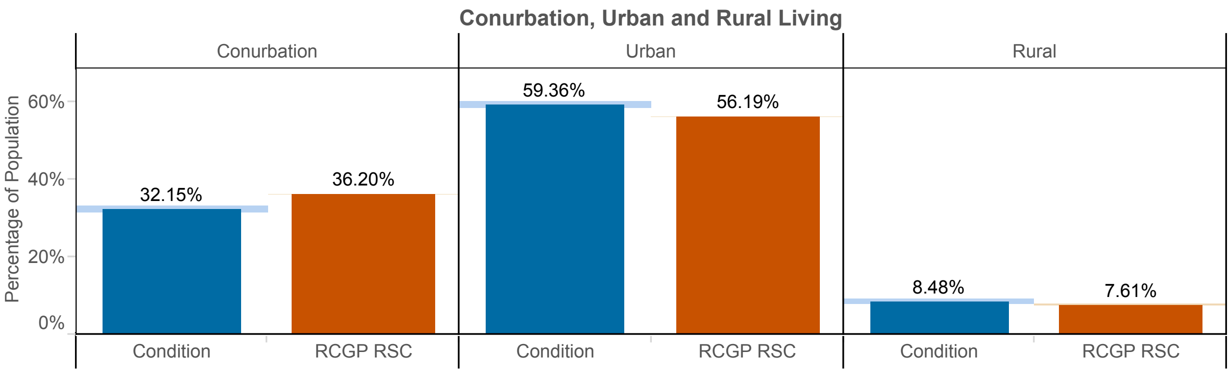

Strep throat / peritonsillar abscess (ICD10 : A38; J020; J36)

Age-sex profile

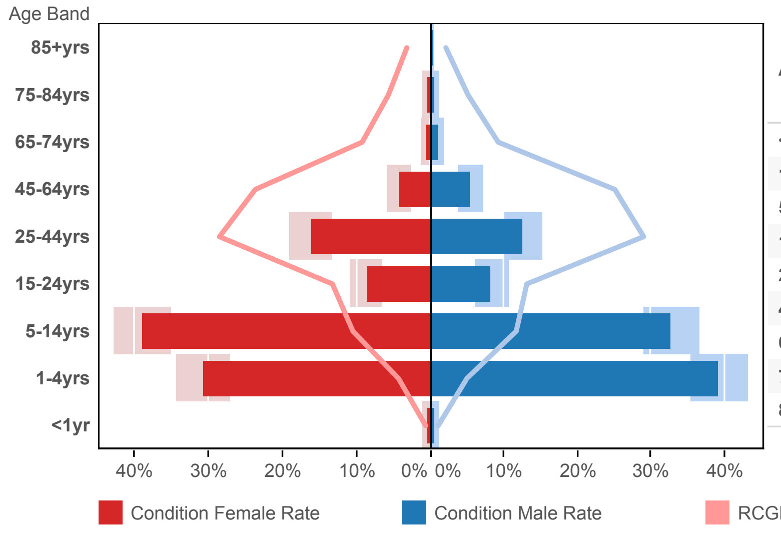

Age Band	Condition Female Rate	Condition Male Rate	Condition Female Population	Condition Male Population
<1yr	0.47	0.50	3.0	3.0
1-4yrs	30.61	39.17	195.0	235.0
5-14yrs	38.78	32.67	247.0	196.0
15-24yrs	8.63	8.17	55.0	49.0
25-44yrs	16.17	12.50	103.0	75.0
45-64yrs	4.24	5.33	27.0	32.0
65-74yrs	0.63	1.00	4.0	6.0
75-84yrs	0.47	0.50	3.0	3.0
85+yrs	0.00	0.17	0.0	1.0

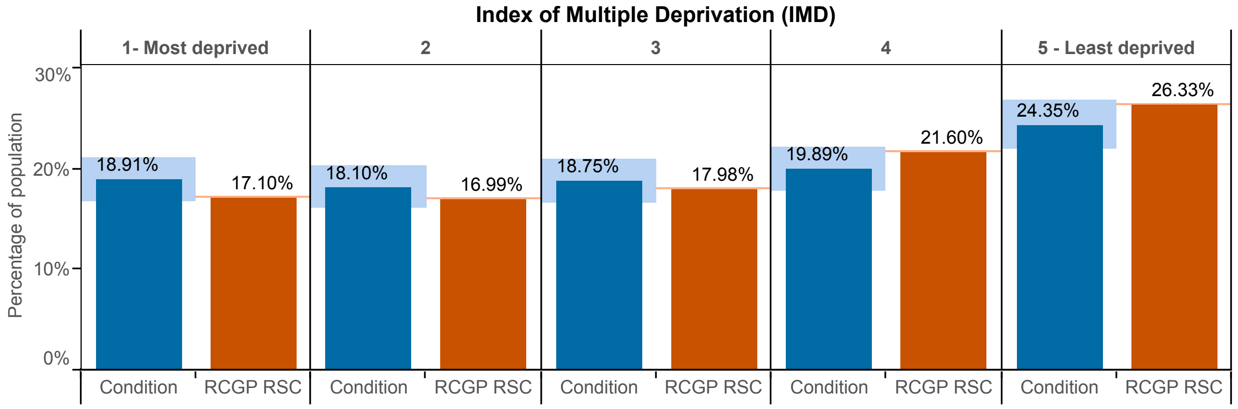

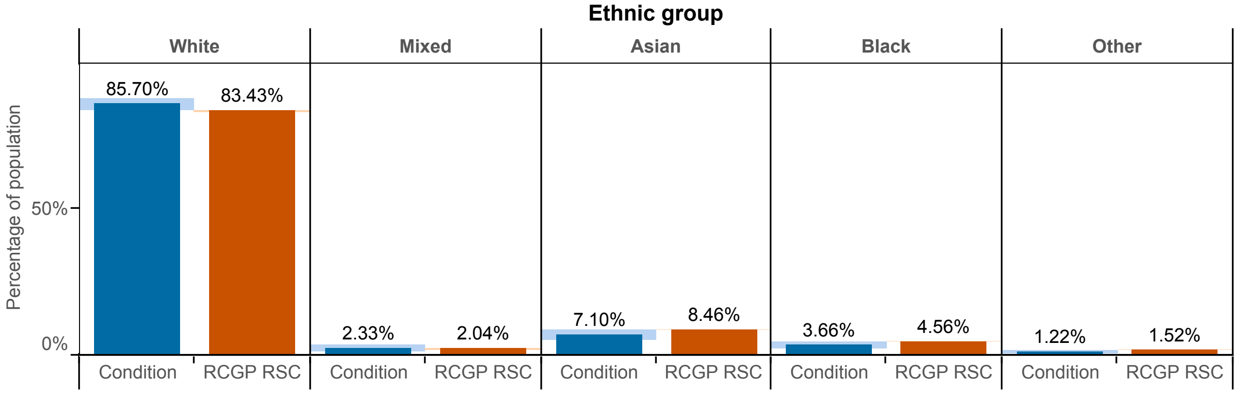

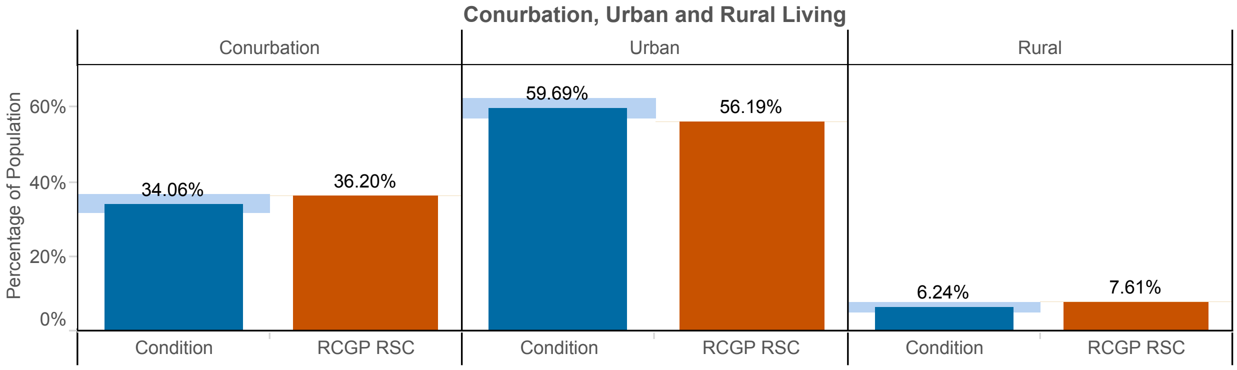

Tonsilitis / Pharyngitis (ICD10 : J02 - J03)

Age-sex profile

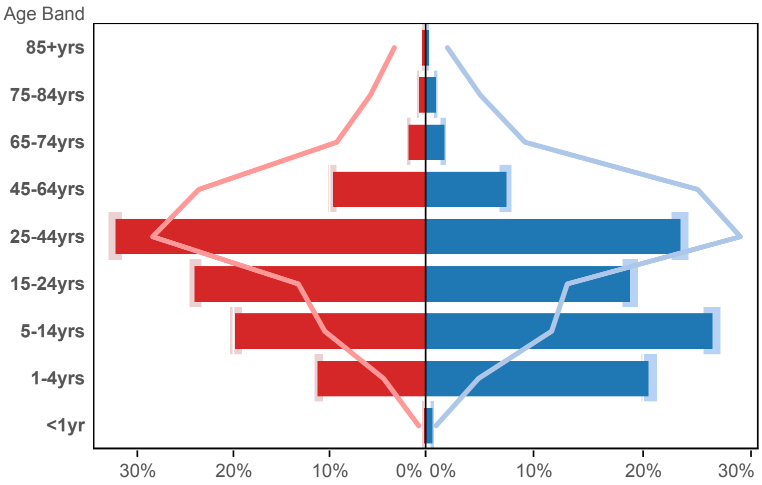

Age Band	Condition Female Rate	Condition Male Rate	Condition Female Population	Condition Male Population
**<1yr**	0.23	0.58	42	67
**1-4yrs**	11.19	20.55	2,041	2,390
**5-14yrs**	19.78	26.41	3,608	3,071
**15-24yrs**	24.00	18.83	4,377	2,189
**25-44yrs**	32.27	23.43	5,886	2,725
**45-64yrs**	9.71	7.38	1,770	858
**65-74yrs**	1.76	1.65	321	192
**75-84yrs**	0.78	0.90	143	105
**85+yrs**	0.27	0.27	50	31

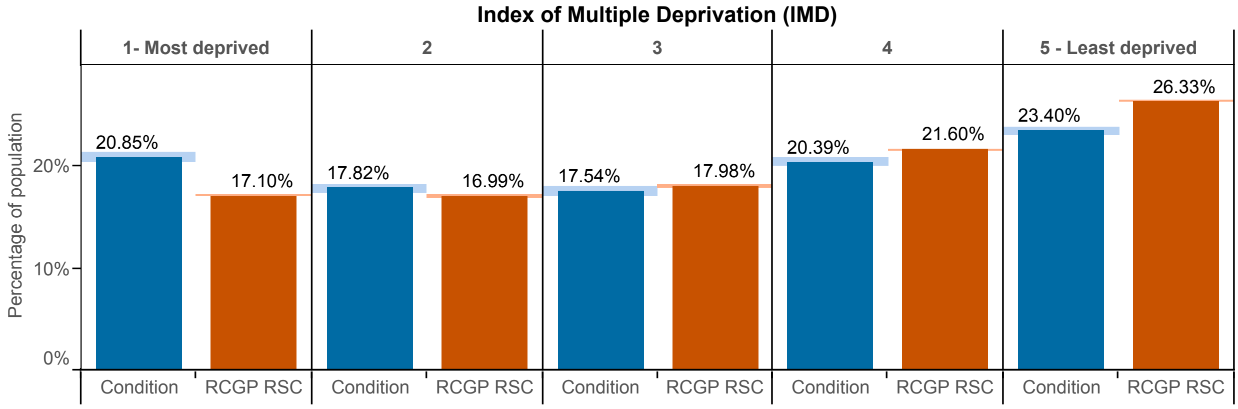

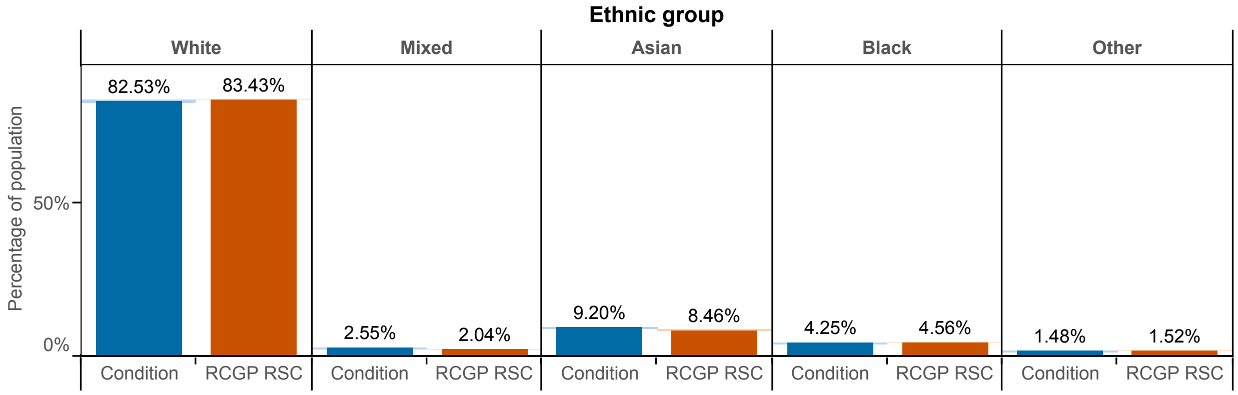

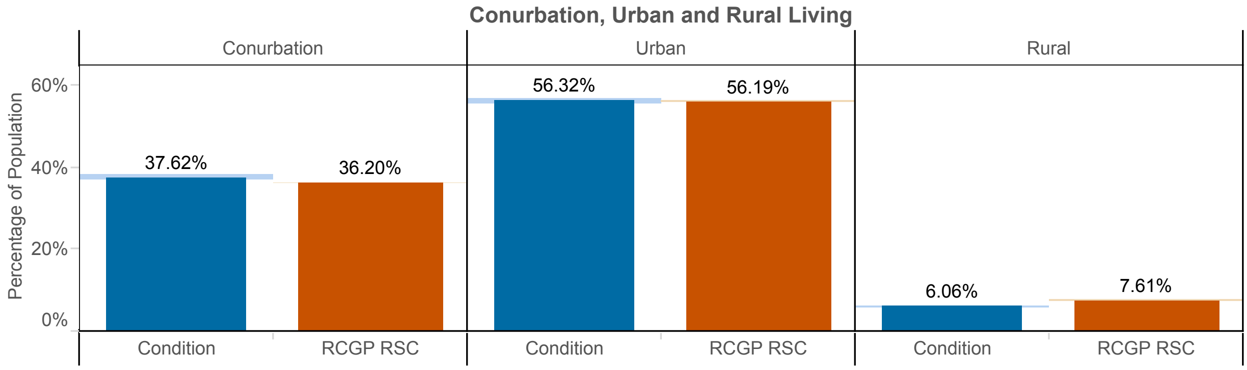

Upper Respiratory Tract Infections (ICD10 : J00 - J06)

Age-sex profile

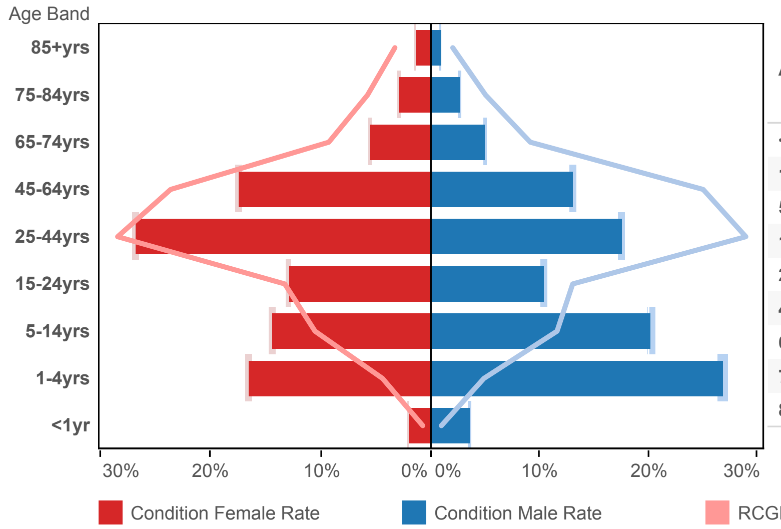

Age Band	Condition Female Rate	Condition Male Rate	Condition Female Population	Condition Male Population
<1yr	2.03	3.51	1,348	1,583
1-4yrs	16.51	26.85	10,986	12,112
5-14yrs	14.38	20.24	9,566	9,130
15-24yrs	12.92	10.35	8,600	4,671
25-44yrs	26.81	17.52	17,839	7,905
45-64yrs	17.48	13.06	11,634	5,892
65-74yrs	5.55	4.97	3,691	2,243
75-84yrs	2.92	2.58	1,940	1,163
85+yrs	1.40	0.91	934	410

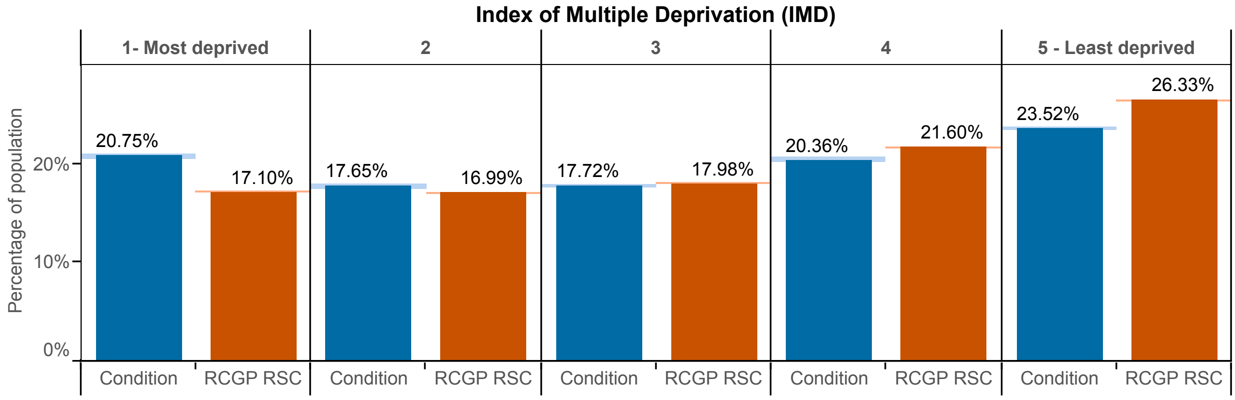

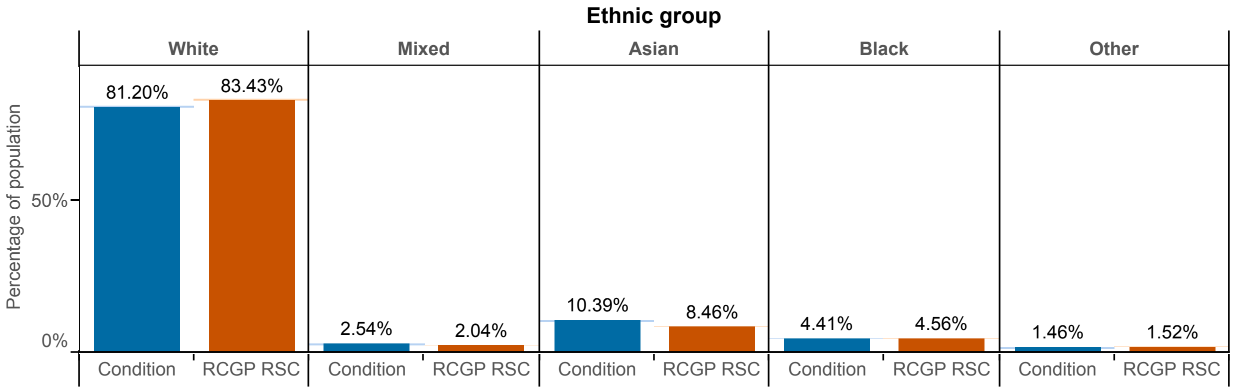

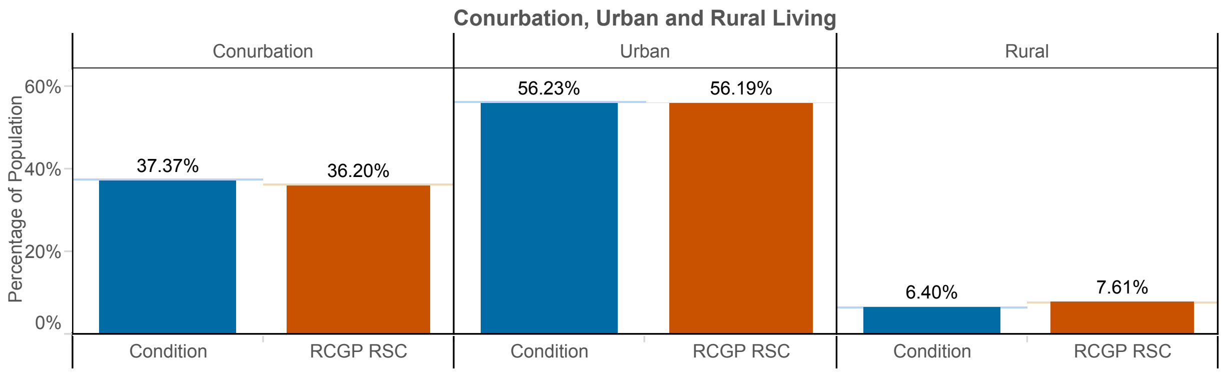

Pertussis (ICD10 : A37)

Age-sex profile

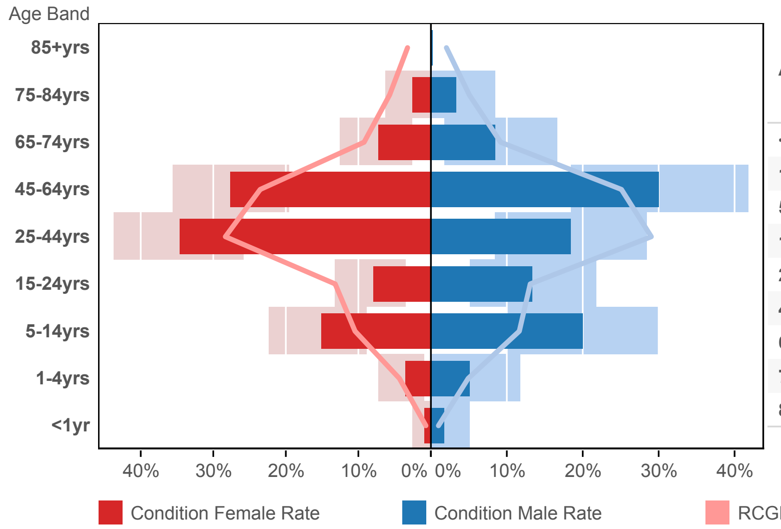

Age Band	Condition Female Rate	Condition Male Rate	Condition Female Population	Condition Male Population
<1yr	0.89	1.67	1.00	1.00
1-4yrs	3.57	5.00	4.00	3.00
5-14yrs	15.18	20.00	17.00	12.00
15-24yrs	8.04	13.33	9.00	8.00
25-44yrs	34.82	18.33	39.00	11.00
45-64yrs	27.68	30.00	31.00	18.00
65-74yrs	7.14	8.33	8.00	5.00
75-84yrs	2.68	3.33	3.00	2.00
85+yrs	0.00	0.00	0.00	0.00

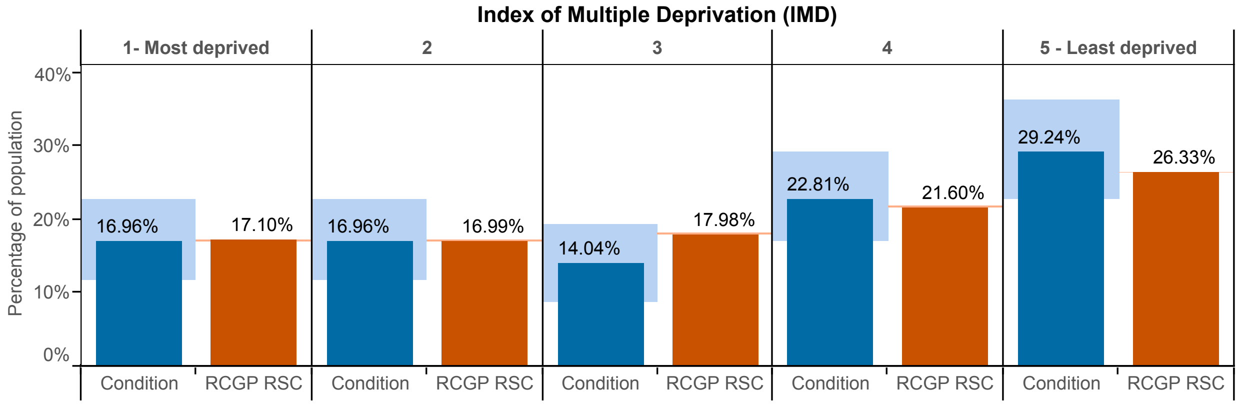

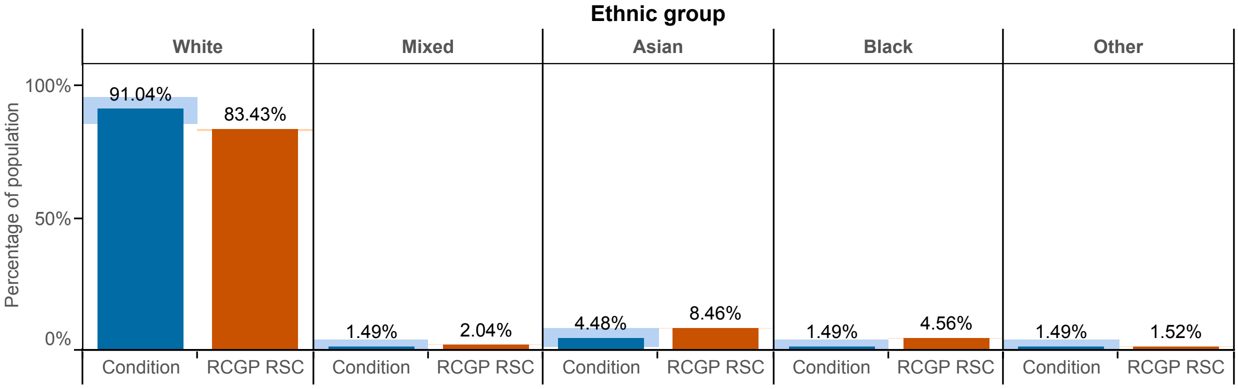

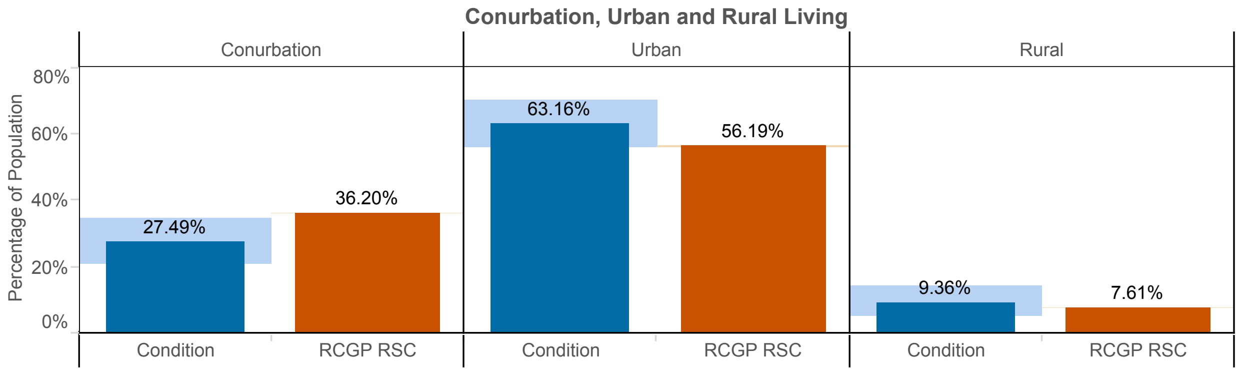

Infectious Mononucleosis (ICD10 : B27)

Age-sex profile

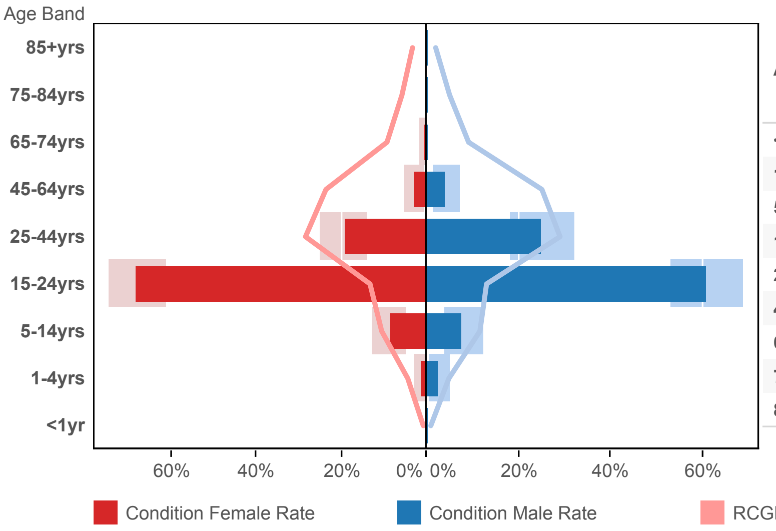

Age Band	Condition Female Rate	Condition Male Rate	Condition Female Population	Condition Male Population
<1yr	0.00	0.00	0.0	0.0
1-4yrs	1.06	2.61	2.0	4.0
5-14yrs	8.51	7.84	16.0	12.0
15-24yrs	68.09	60.78	128.0	93.0
25-44yrs	19.15	24.84	36.0	38.0
45-64yrs	2.66	3.92	5.0	6.0
65-74yrs	0.53	0.00	1.0	0.0
75-84yrs	0.00	0.00	0.0	0.0
85+yrs	0.00	0.00	0.0	0.0

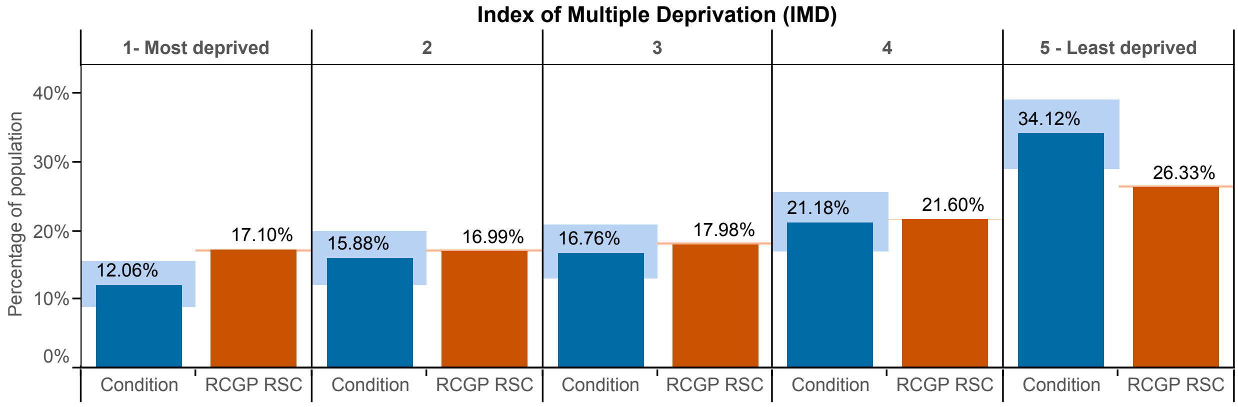

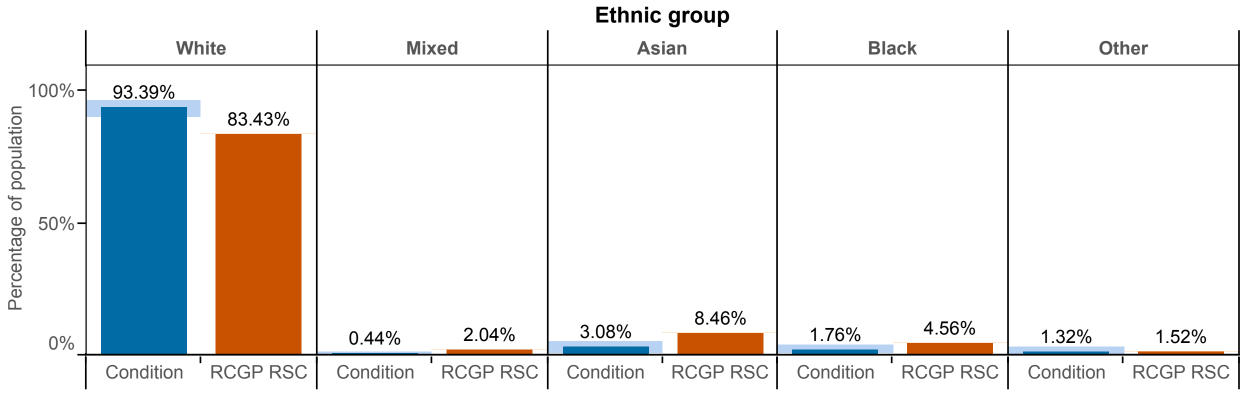

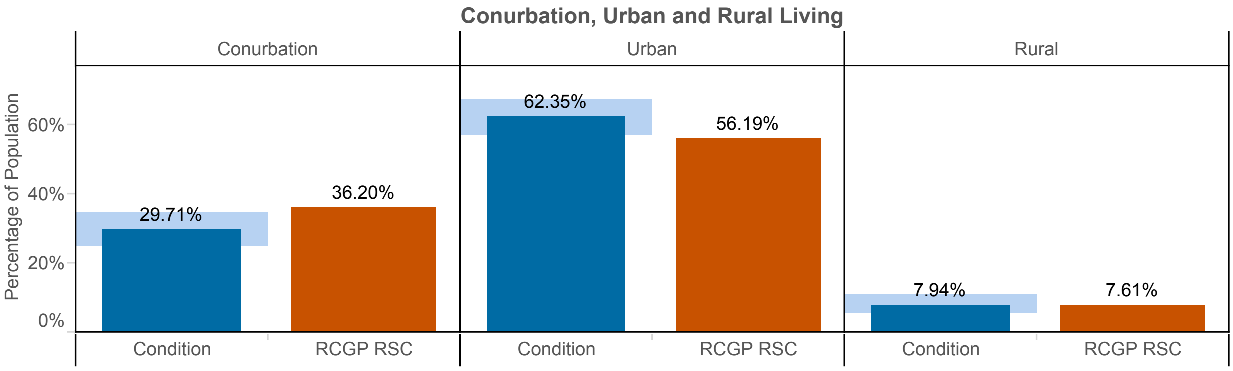

Lower Respiratory Tract Infections (ICD10 : J20-J22)

Age-sex profile

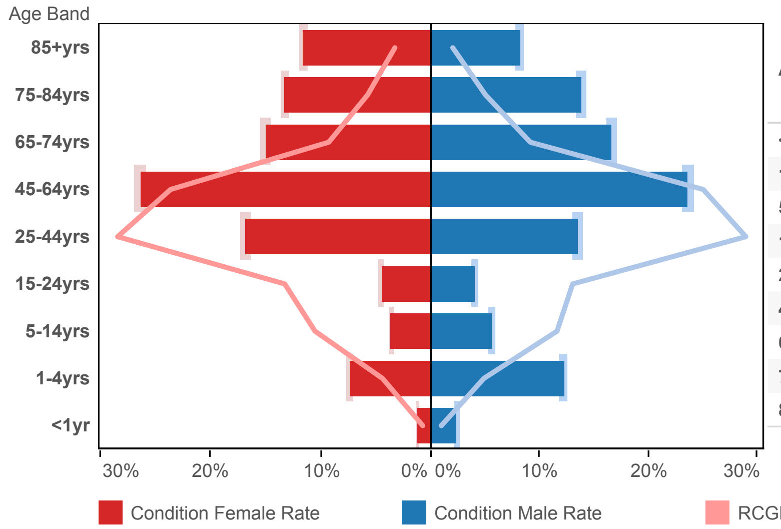

Age Band	Condition Female Rate	Condition Male Rate	Condition Female Population	Condition Male Population
<1yr	1.25	2.37	338	499
1-4yrs	7.42	12.20	2,003	2,569
5-14yrs	3.62	5.61	978	1,181
15-24yrs	4.49	4.03	1,213	848
25-44yrs	16.89	13.50	4,560	2,842
45-64yrs	26.40	23.65	7,126	4,981
65-74yrs	15.04	16.58	4,061	3,492
75-84yrs	13.29	13.87	3,588	2,921
85+yrs	11.58	8.20	3,126	1,726

Index of Multiple Deprivation (IMD)

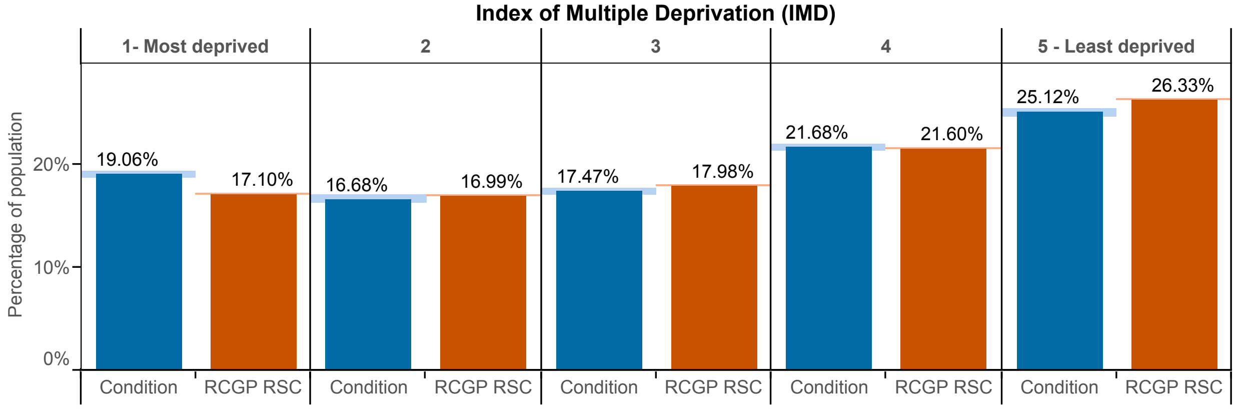

Ethnic group

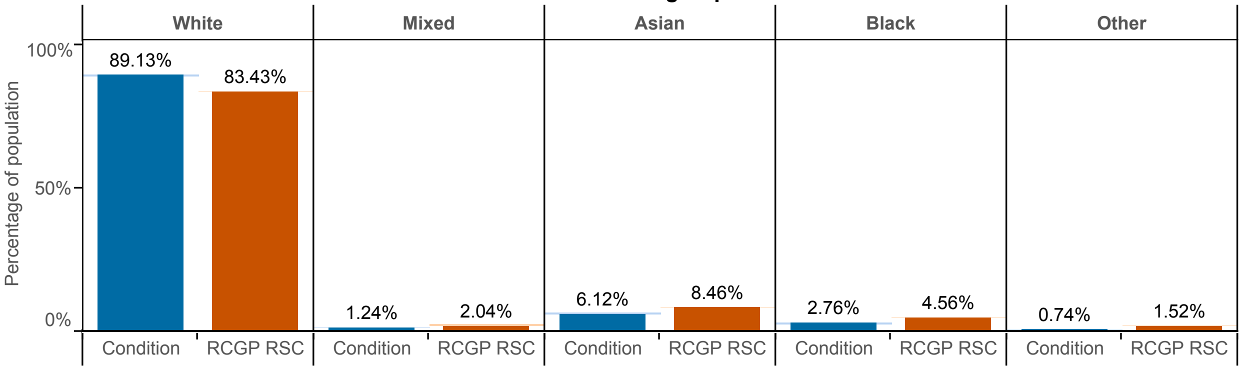

Conurbation, Urban and Rural Living

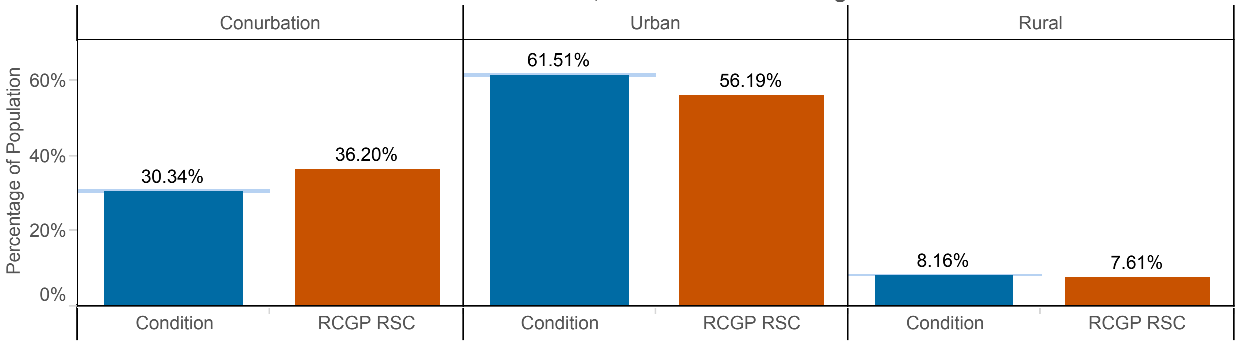

Acute Otitis Media (ICD10 : H650 - H651; H660; H669)

Age-sex profile

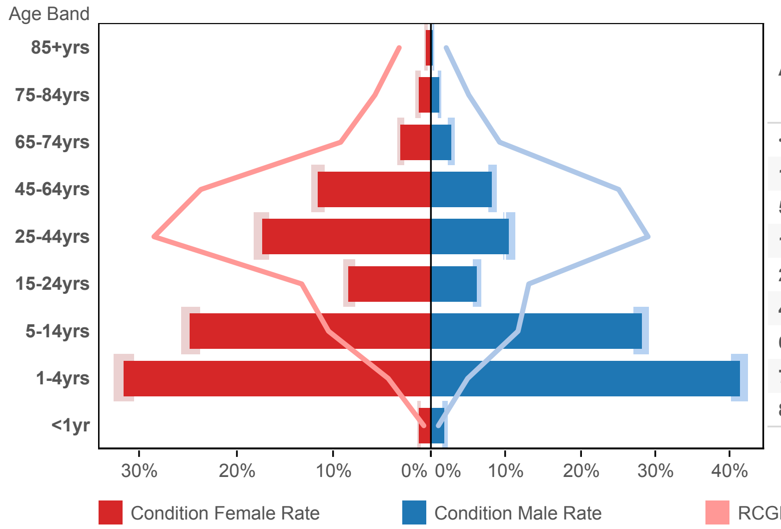

Age Band	Condition Female Rate	Condition Male Rate	Condition Female Population	Condition Male Population
<1yr	1.23	1.90	103	139
1-4yrs	31.51	41.15	2,647	3,017
5-14yrs	24.72	28.13	2,077	2,062
15-24yrs	8.44	6.10	709	447
25-44yrs	17.40	10.48	1,462	768
45-64yrs	11.67	8.21	980	602
65-74yrs	3.15	2.69	265	197
75-84yrs	1.30	1.04	109	76
85+yrs	0.58	0.31	49	23

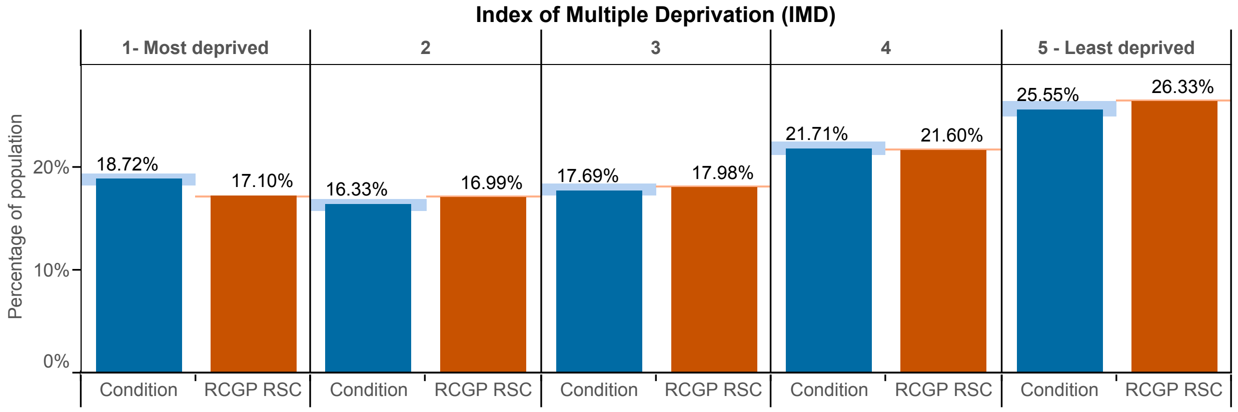

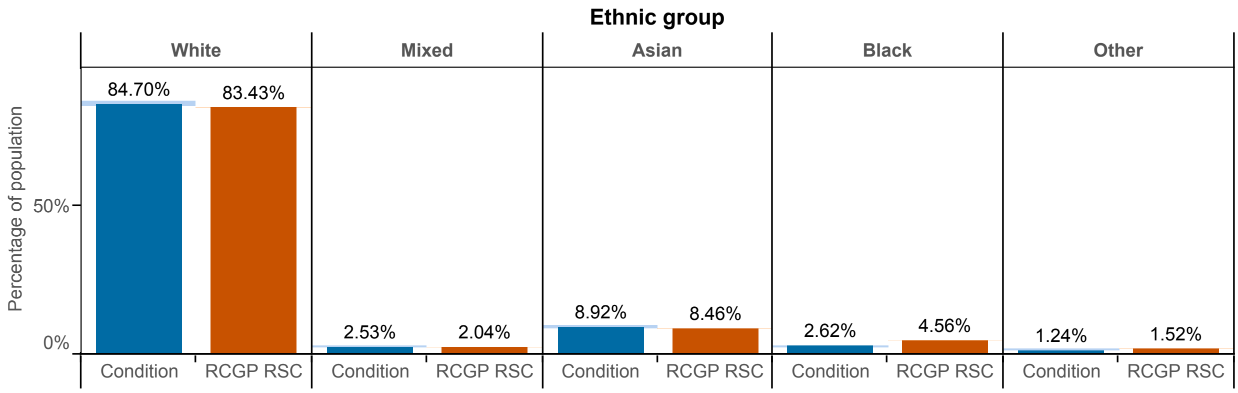

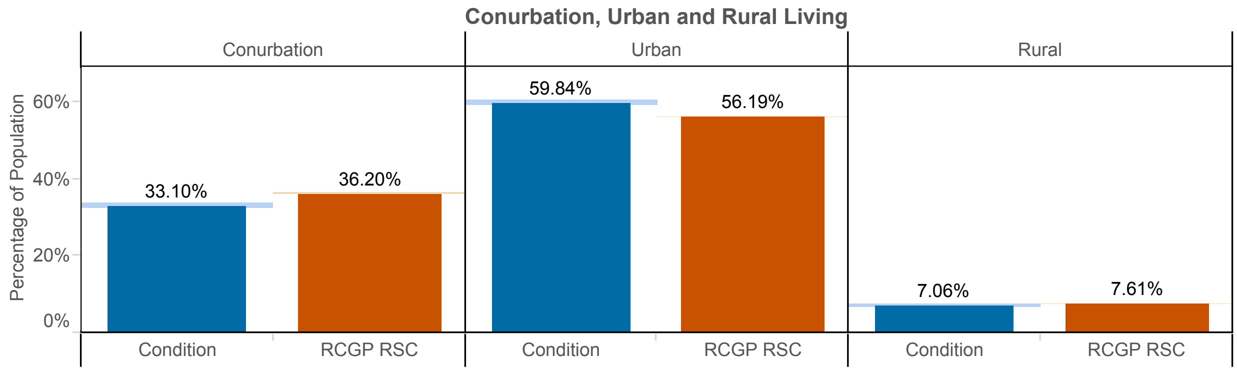

4. Vaccine Sensitive Disorders:

Measles (ICD10 : B05)

Age-sex profile

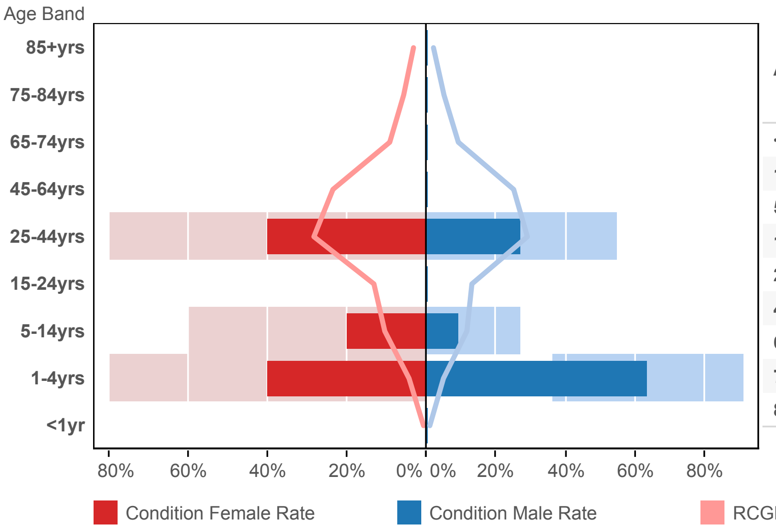

Age Band	Condition Female Rate	Condition Male Rate	Condition Female Population	Condition Male Population
<1yr	0.00	0.00	0.00	0.00
1-4yrs	40.00	63.64	2.00	7.00
5-14yrs	20.00	9.09	1.00	1.00
15-24yrs	0.00	0.00	0.00	0.00
25-44yrs	40.00	27.27	2.00	3.00
45-64yrs	0.00	0.00	0.00	0.00
65-74yrs	0.00	0.00	0.00	0.00
75-84yrs	0.00	0.00	0.00	0.00
85+yrs	0.00	0.00	0.00	0.00

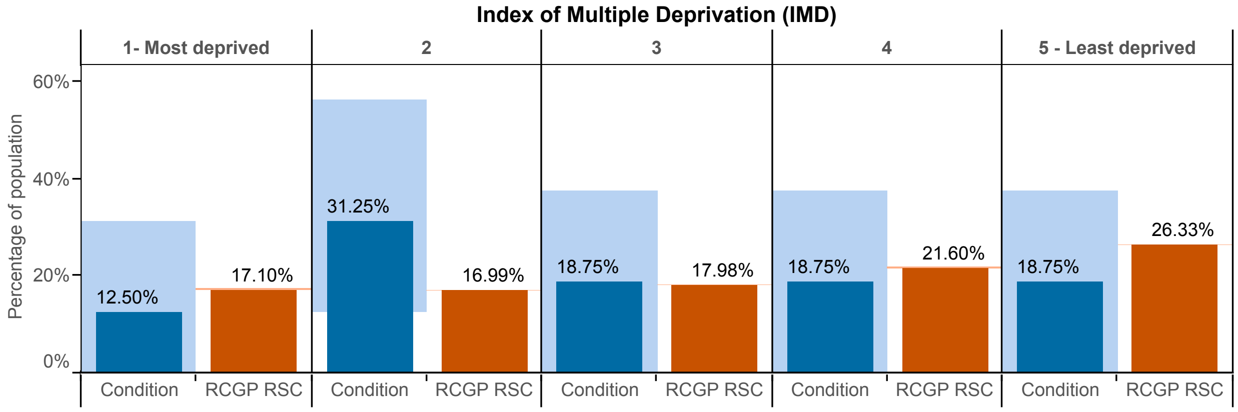

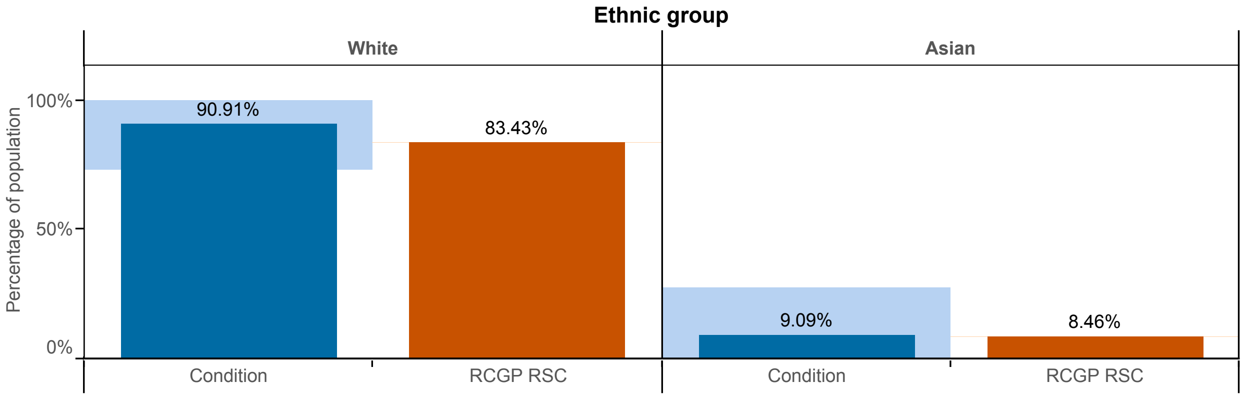

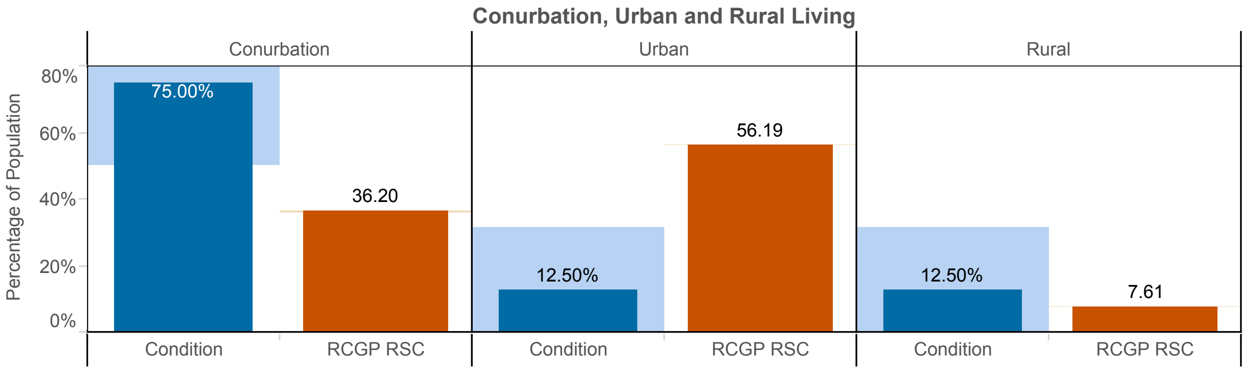

Mumps (ICD10 : B26)

Age-sex profile

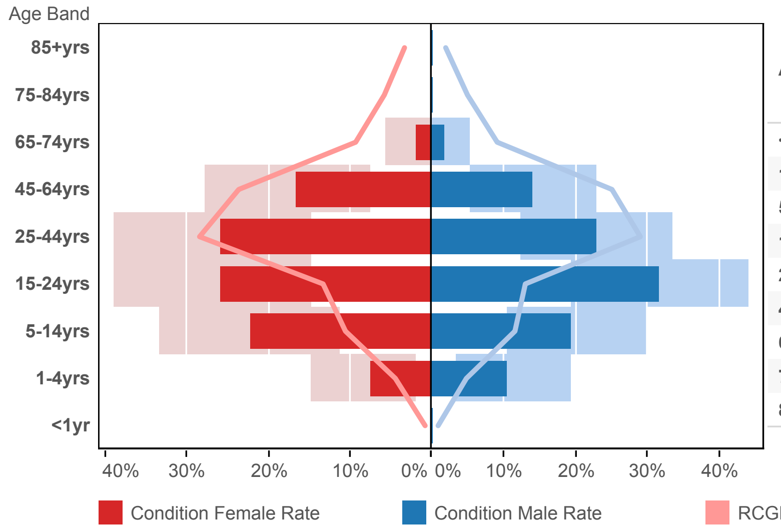

Age Band	Condition Female Rate	Condition Male Rate	Condition Female Population	Condition Male Population
<1yr	0.00	0.00	0.00	0.00
1-4yrs	7.41	10.53	4.00	6.00
5-14yrs	22.22	19.30	12.00	11.00
15-24yrs	25.93	31.58	14.00	18.00
25-44yrs	25.93	22.81	14.00	13.00
45-64yrs	16.67	14.04	9.00	8.00
65-74yrs	1.85	1.75	1.00	1.00
75-84yrs	0.00	0.00	0.00	0.00
85+yrs	0.00	0.00	0.00	0.00

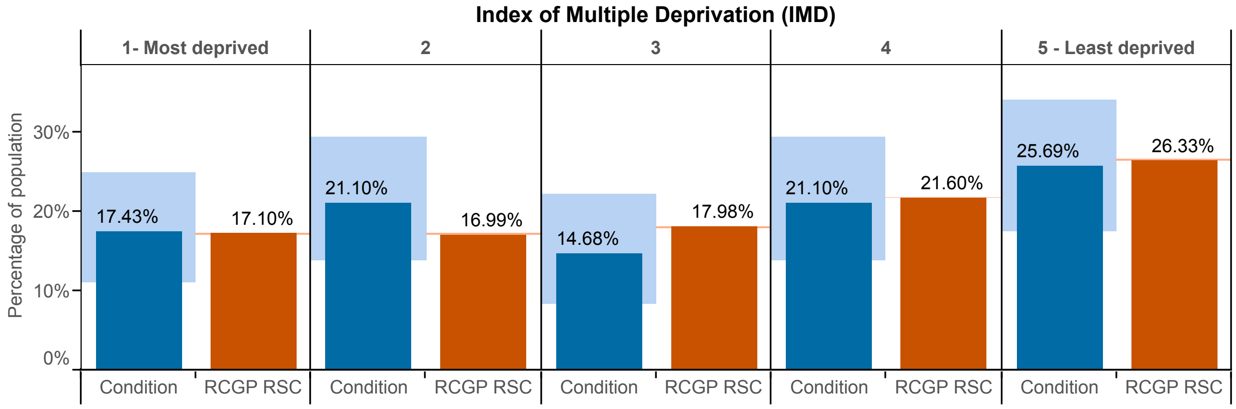

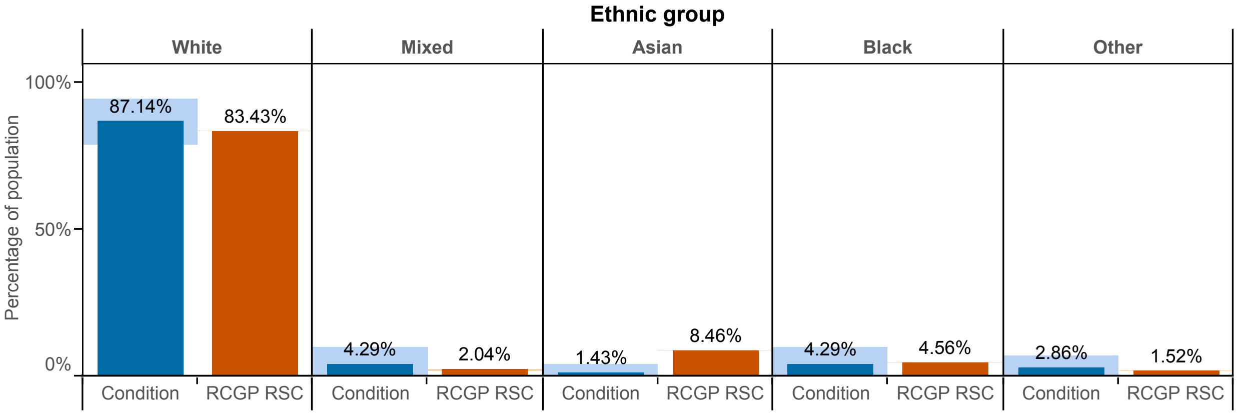

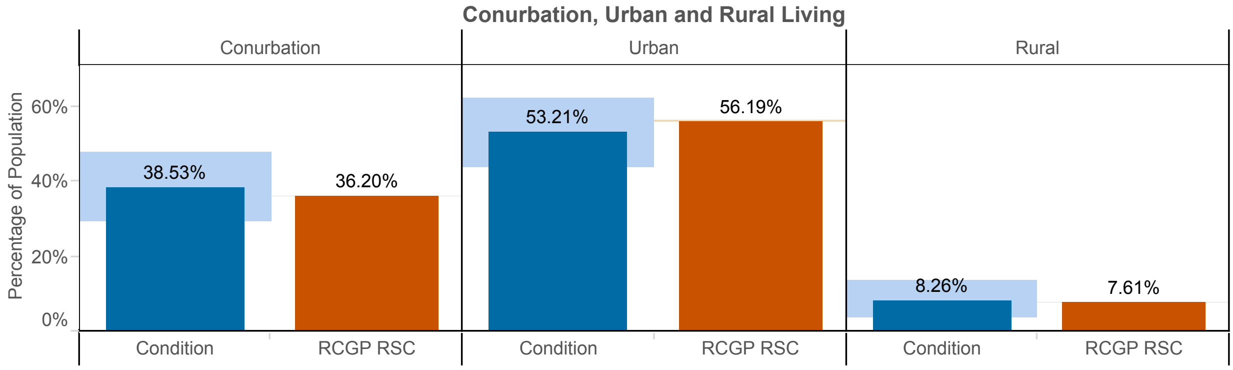

Rubella (ICD10 : B06)

Age-sex profile

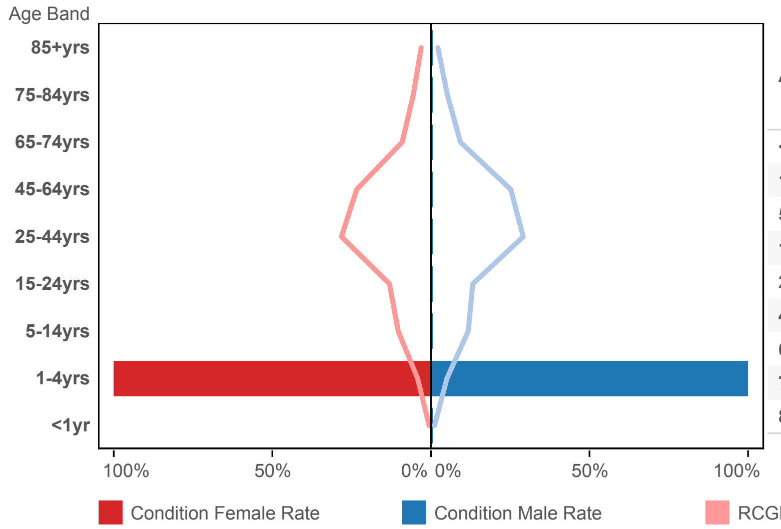

Age Band	Condition Female Rate	Condition Male Rate	Condition Female Population	Condition Male Population
<1yr	0.00	0.00	0.0	0.0
1-4yrs	100.00	100.00	2.0	2.0
5-14yrs	0.00	0.00	0.0	0.0
15-24yrs	0.00	0.00	0.0	0.0
25-44yrs	0.00	0.00	0.0	0.0
45-64yrs	0.00	0.00	0.0	0.0
65-74yrs	0.00	0.00	0.0	0.0
75-84yrs	0.00	0.00	0.0	0.0
85+yrs	0.00	0.00	0.0	0.0

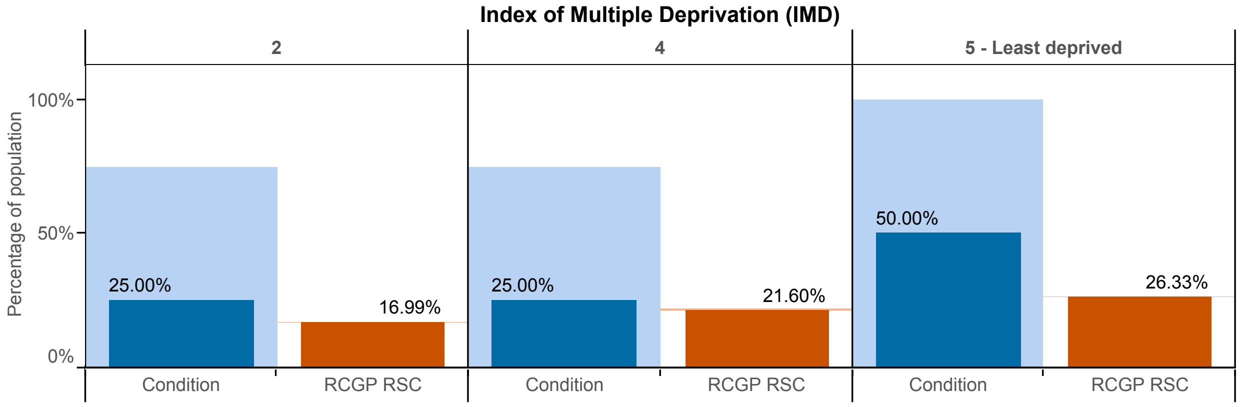

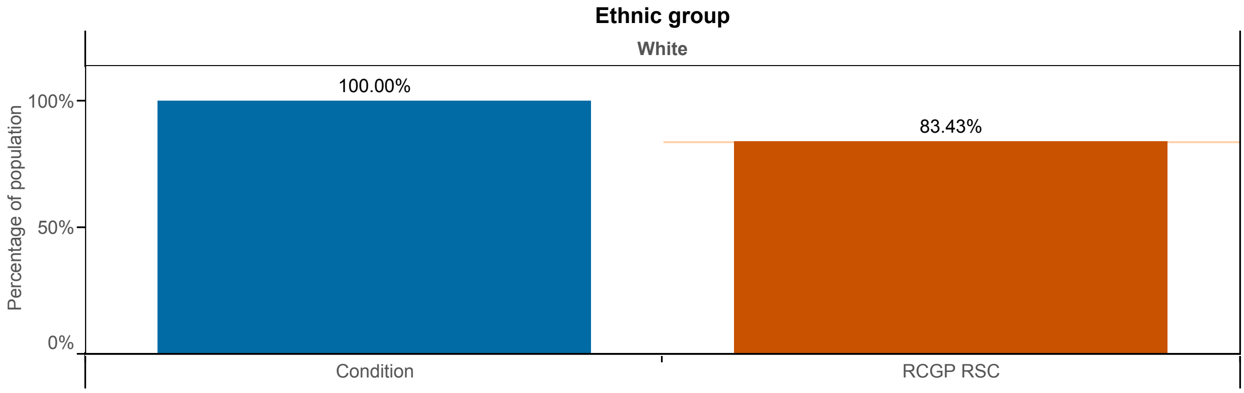

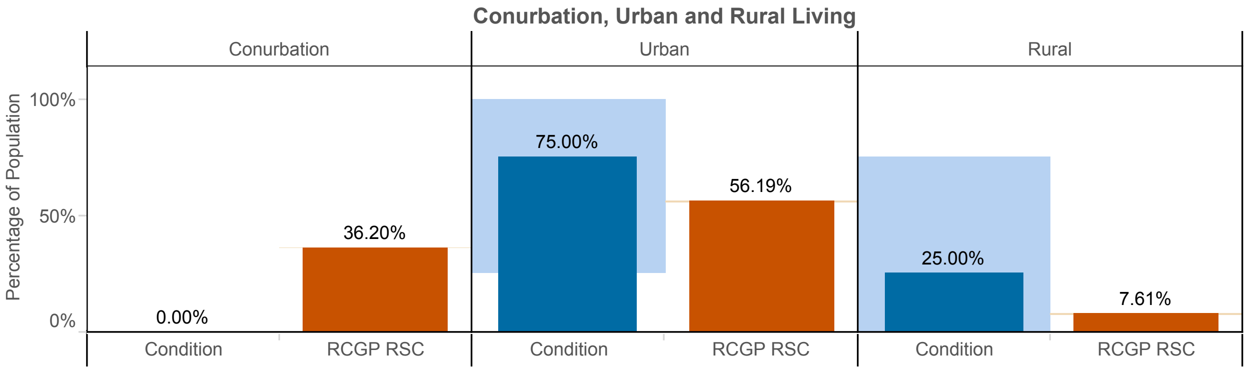

5. Skin Contagions:

Bullous Dermatoses (ICD10 : L10 - L14)

Age-sex profile

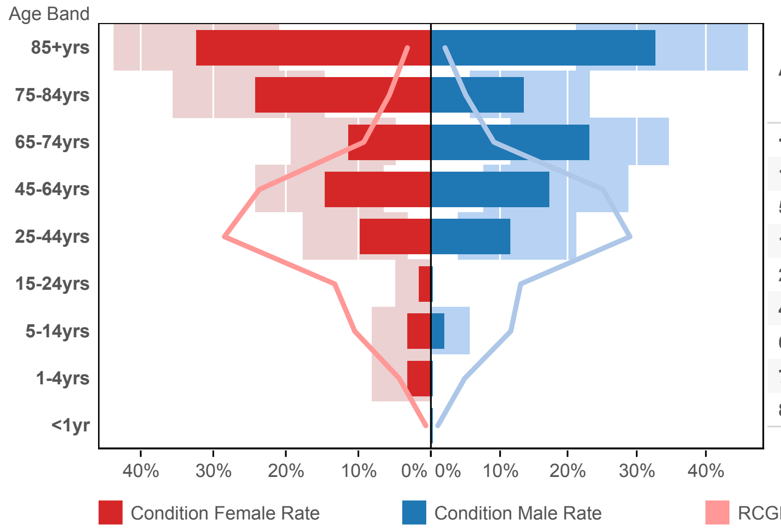

Age Band	Condition Female Rate	Condition Male Rate	Condition Female Population	Condition Male Population
**<1yr**	0.00	0.00	0.00	0.00
**1-4yrs**	3.23	0.00	2.00	0.00
**5-14yrs**	3.23	1.92	2.00	1.00
**15-24yrs**	1.61	0.00	1.00	0.00
**25-44yrs**	9.68	11.54	6.00	6.00
**45-64yrs**	14.52	17.31	9.00	9.00
**65-74yrs**	11.29	23.08	7.00	12.00
**75-84yrs**	24.19	13.46	15.00	7.00
**85+yrs**	32.26	32.69	20.00	17.00

Index of Multiple Deprivation (IMD)

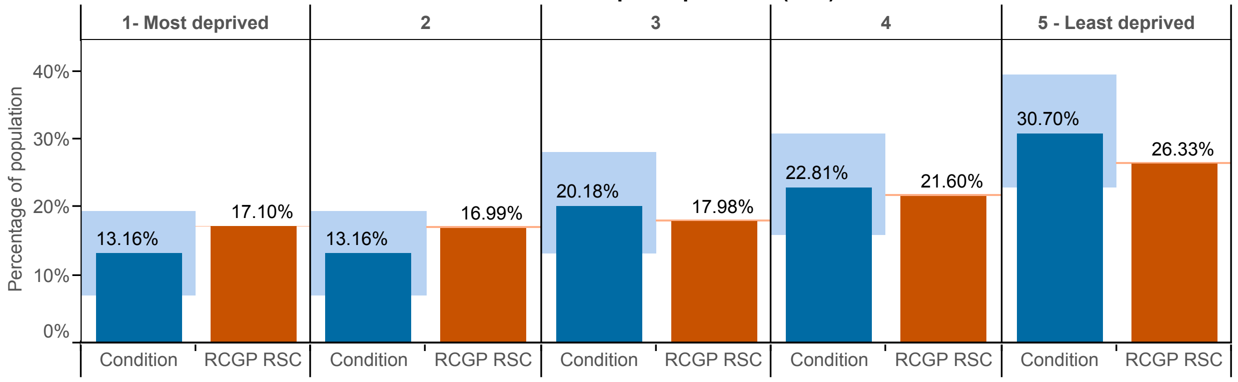

Ethnic group

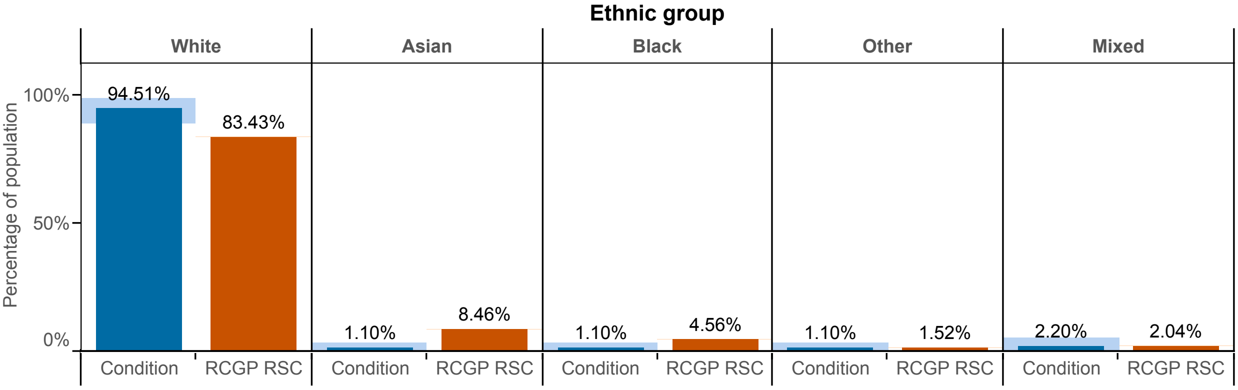

Conurbation, Urban and Rural Living

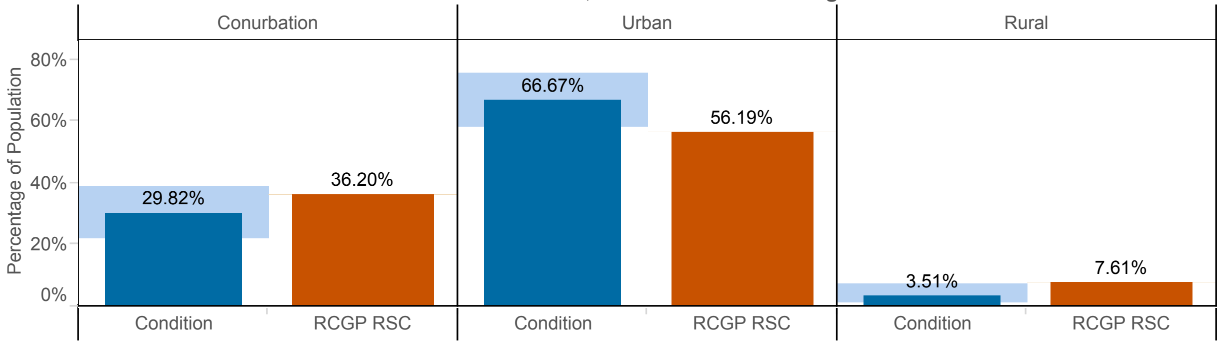

Chickenpox (ICD10 : B01)

Age-sex profile

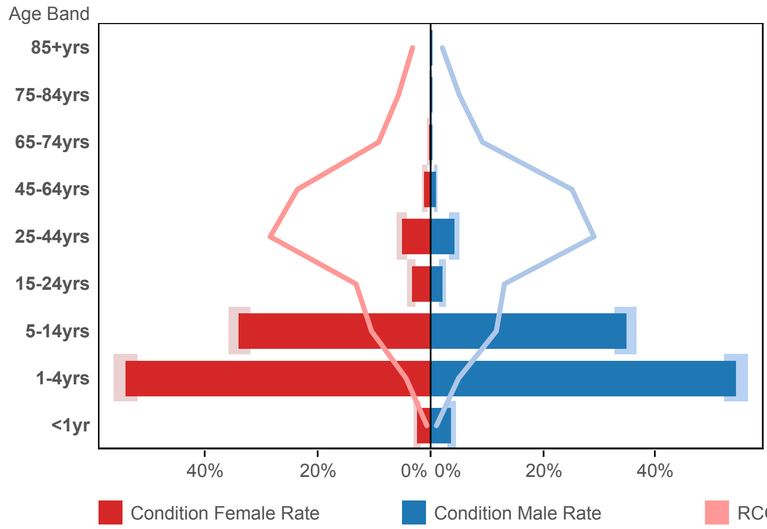

Age Band	Condition Female Rate	Condition Male Rate	Condition Female Population	Condition Male Population
<1yr	2.38	3.70	52	85
1-4yrs	53.84	54.17	1,177	1,246
5-14yrs	33.90	34.74	741	799
15-24yrs	3.29	2.04	72	47
25-44yrs	5.08	4.22	111	97
45-64yrs	1.14	0.87	25	20
65-74yrs	0.23	0.17	5	4
75-84yrs	0.14	0.09	3	2
85+yrs	0.00	0.00	0	0

Index of Multiple Deprivation (IMD)

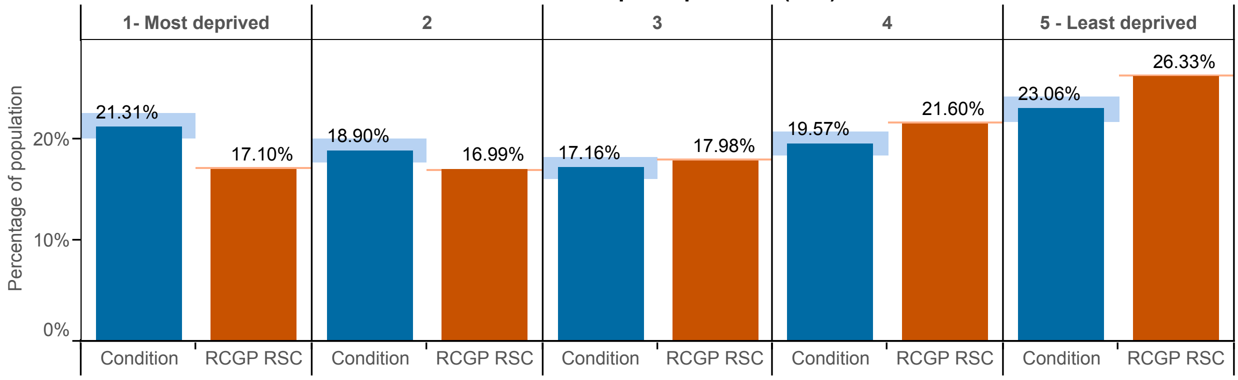

Ethnic group

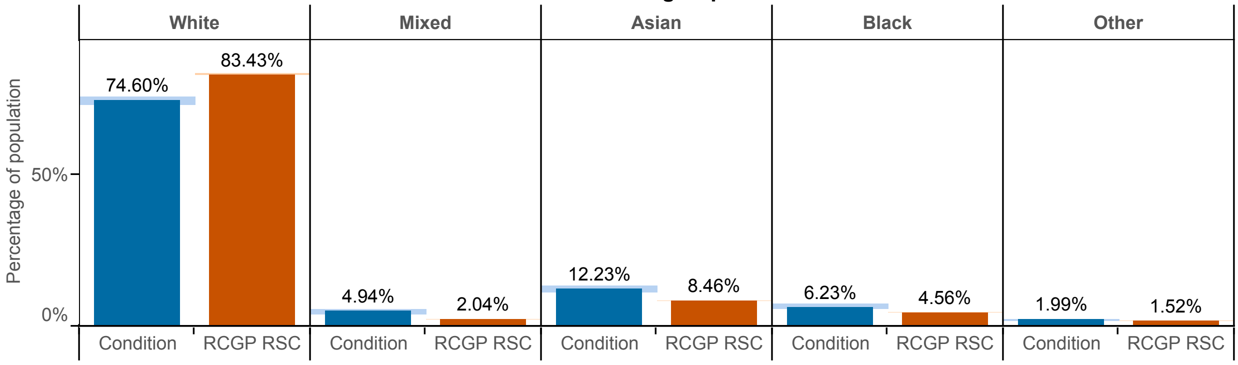

Conurbation, Urban and Rural Living

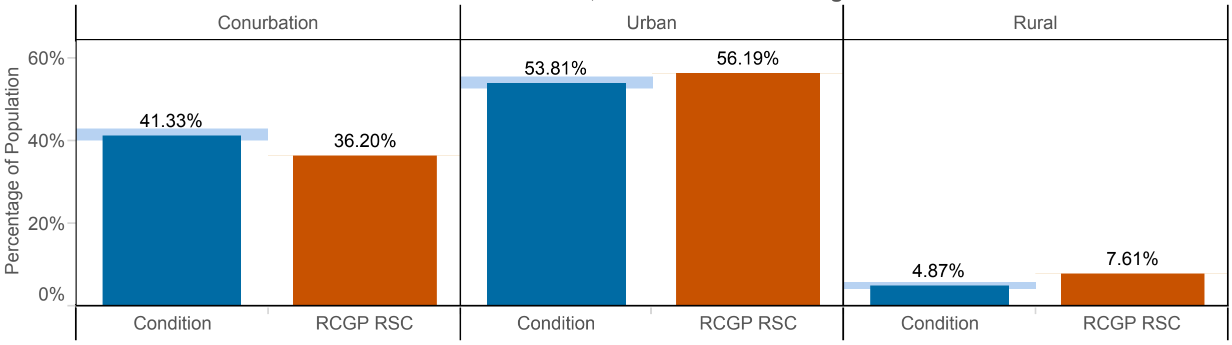

Herpes Simplex (ICD10 : B00)

Age-sex profile

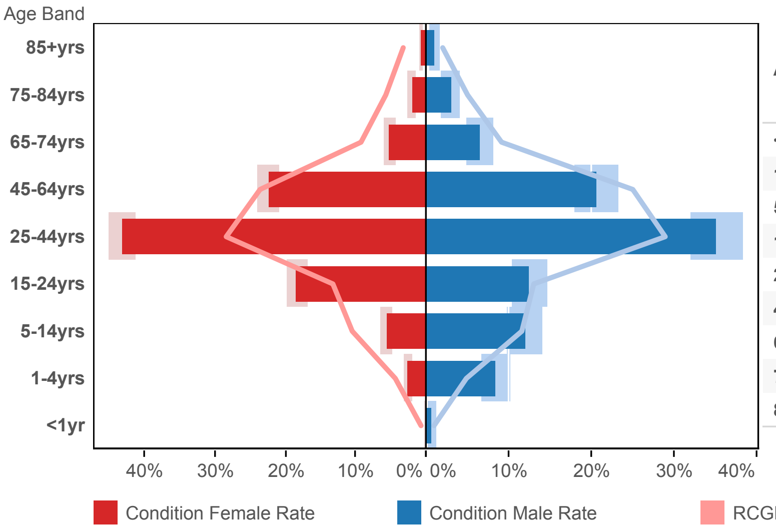

Age Band	Condition Female Rate	Condition Male Rate	Condition Female Population	Condition Male Population
<1yr	0.11	0.67	3	6
1-4yrs	2.52	8.43	66	76
5-14yrs	5.62	11.97	147	108
15-24yrs	18.36	12.53	480	113
25-44yrs	43.11	35.25	1,127	318
45-64yrs	22.38	20.62	585	186
65-74yrs	5.16	6.54	135	59
75-84yrs	2.03	2.99	53	27
85+yrs	0.69	1.00	18	9

Index of Multiple Deprivation (IMD)

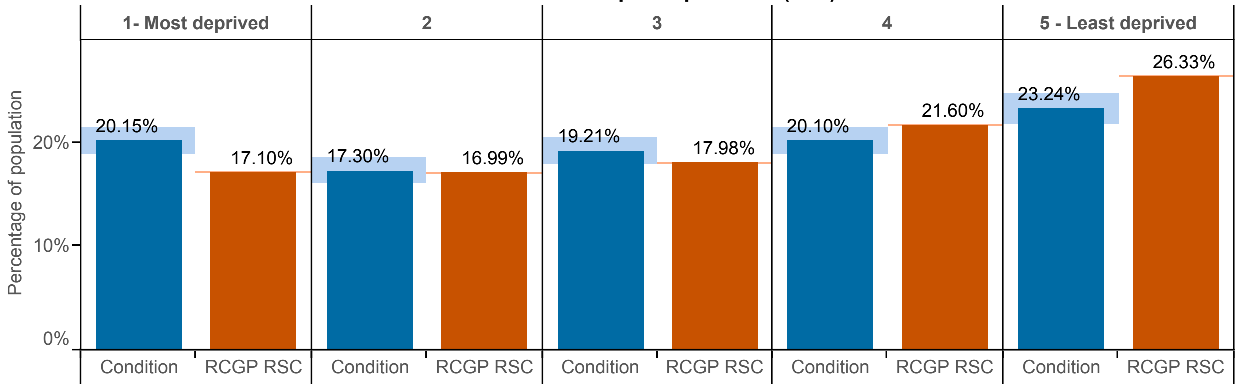

Ethnic group

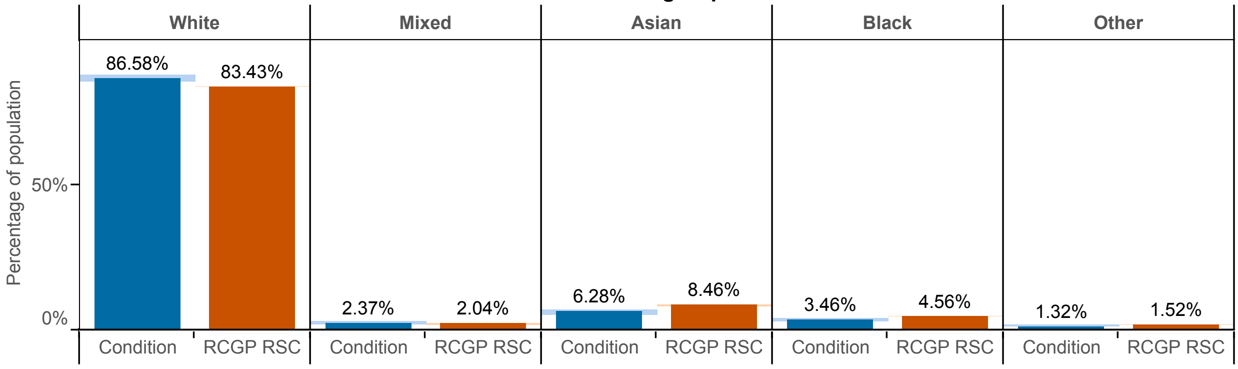

Conurbation, Urban and Rural Living

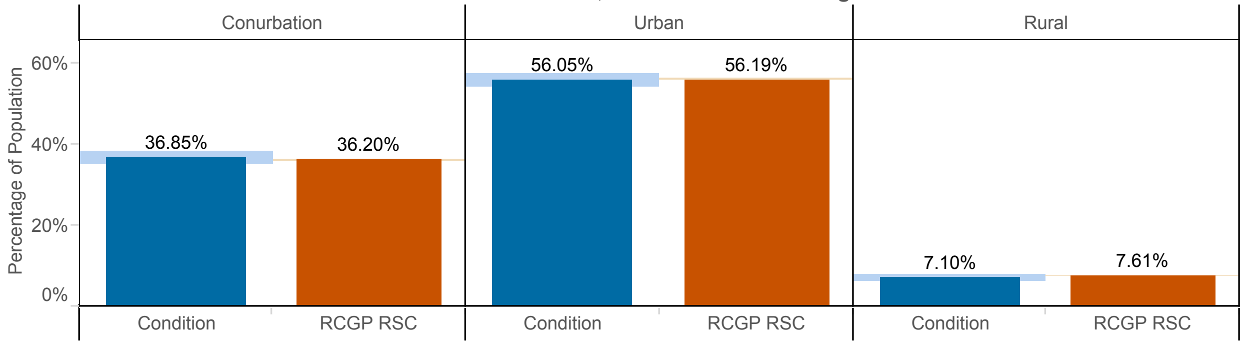

Herpes Zoster (ICD10 : B02)

Age-sex profile

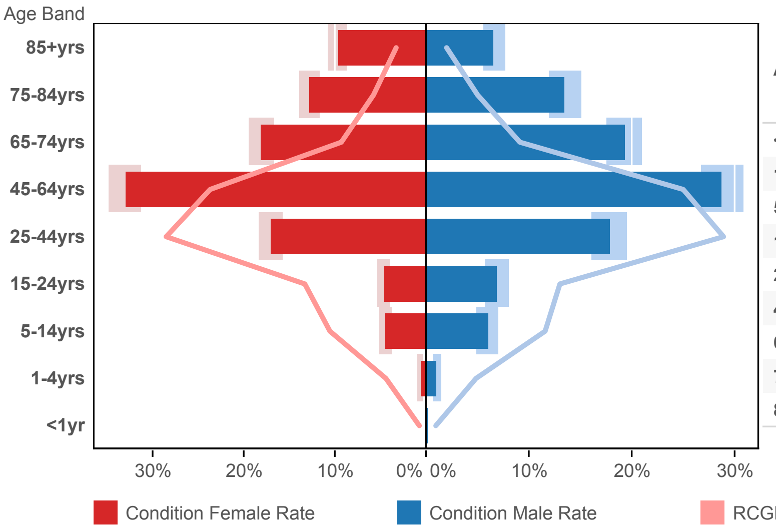

Age Band	Condition Female Rate	Condition Male Rate	Condition Female Population	Condition Male Population
**<1yr**	0.00	0.00	0.0	0.0
**1-4yrs**	0.63	0.99	19.0	19.0
**5-14yrs**	4.35	6.02	131.0	115.0
**15-24yrs**	4.65	6.91	140.0	132.0
**25-44yrs**	16.98	17.84	511.0	341.0
**45-64yrs**	32.96	28.78	992.0	550.0
**65-74yrs**	18.04	19.31	543.0	369.0
**75-84yrs**	12.72	13.50	383.0	258.0
**85+yrs**	9.67	6.65	291.0	127.0

Index of Multiple Deprivation (IMD)

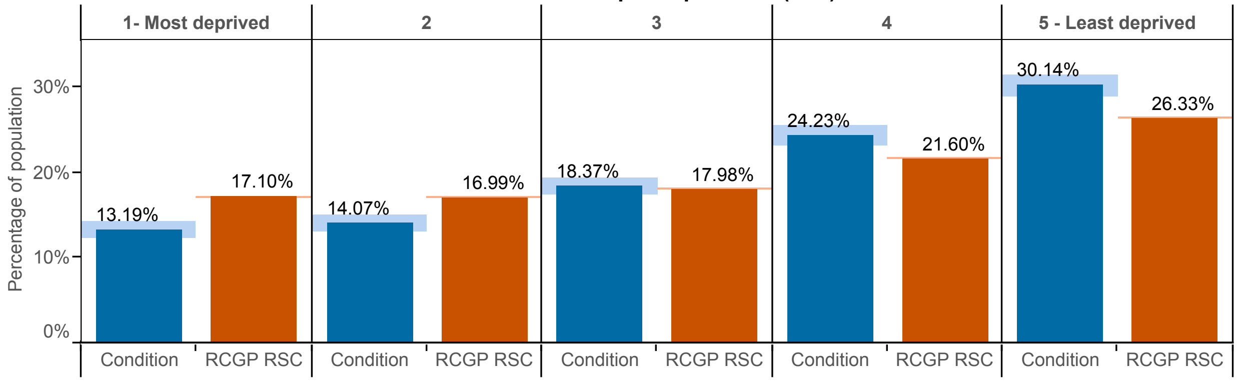

Ethnic group

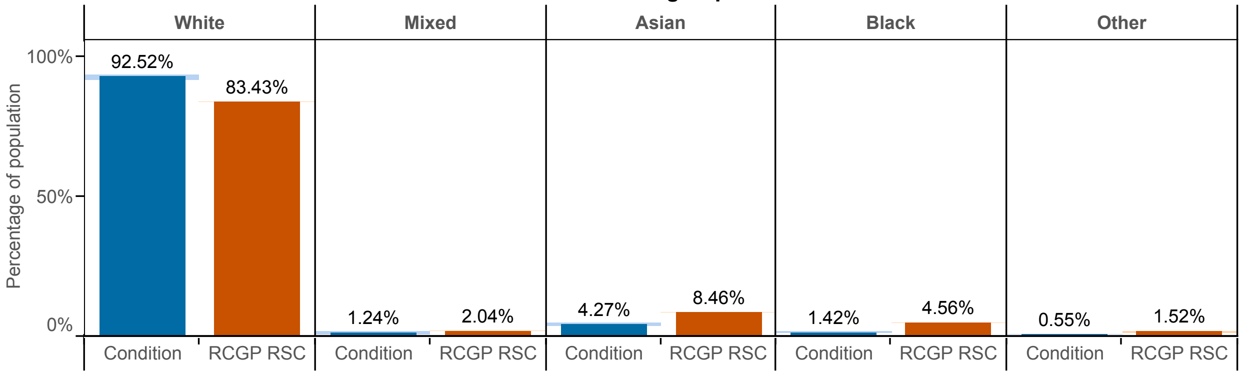

Conurbation, Urban and Rural Living

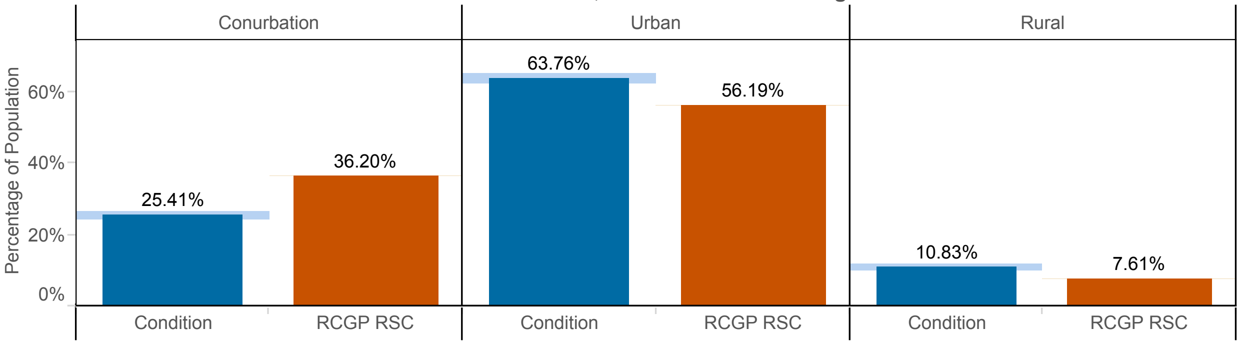

Skin / subcutaneous infections (ICD10 : L00 - L08)

Age-sex profile

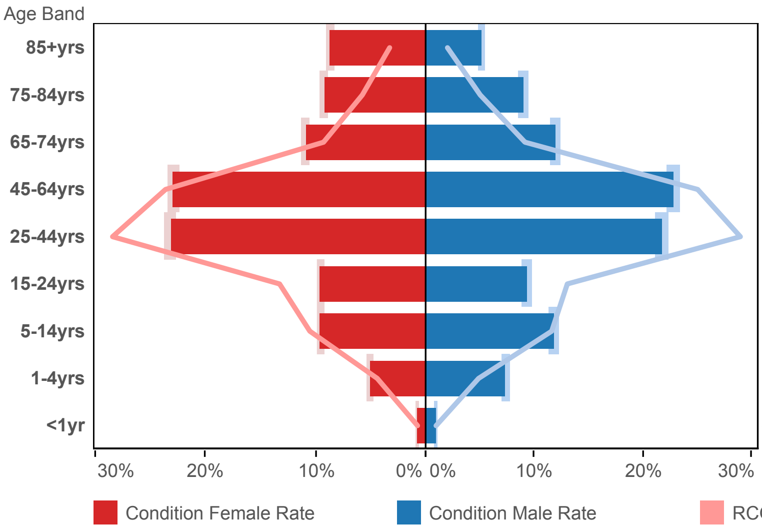

Age Band	Condition Female Rate	Condition Male Rate	Condition Female Population	Condition Male Population
**<1yr**	0.78	0.92	163	155
**1-4yrs**	5.04	7.35	1,051	1,240
**5-14yrs**	9.60	11.82	2,004	1,995
**15-24yrs**	9.60	9.33	2,003	1,574
**25-44yrs**	23.17	21.69	4,835	3,660
**45-64yrs**	22.94	22.84	4,787	3,853
**65-74yrs**	10.93	11.95	2,280	2,016
**75-84yrs**	9.26	8.98	1,933	1,515
**85+yrs**	8.68	5.13	1,811	865

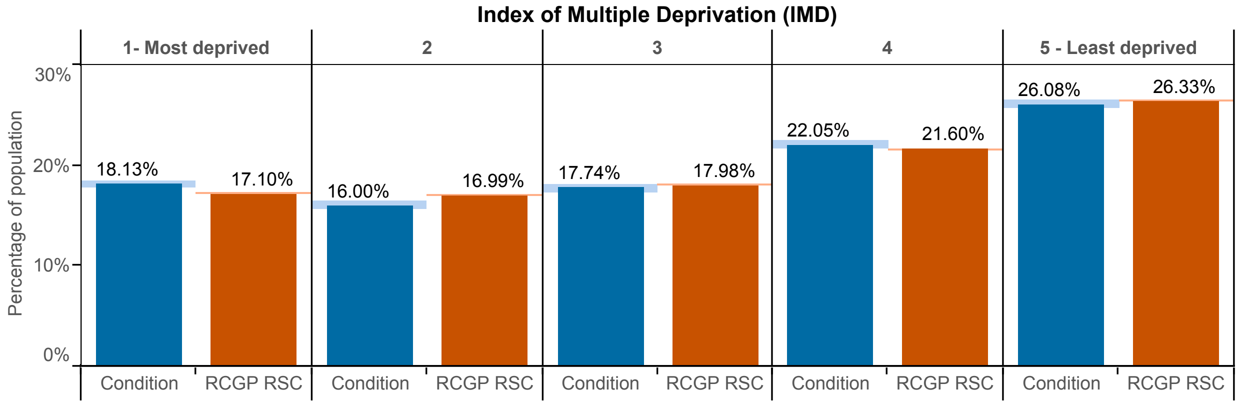

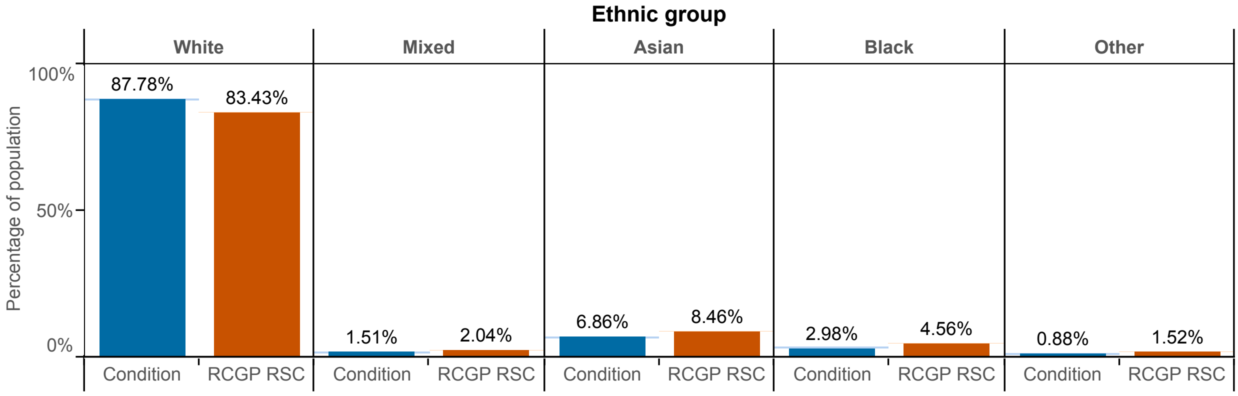

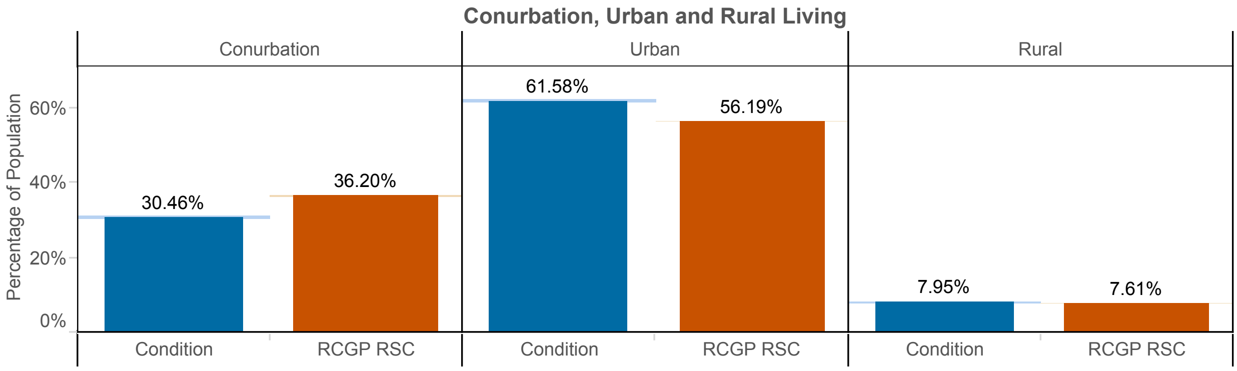

Scabies (ICD10 : B86)

Age-sex profile

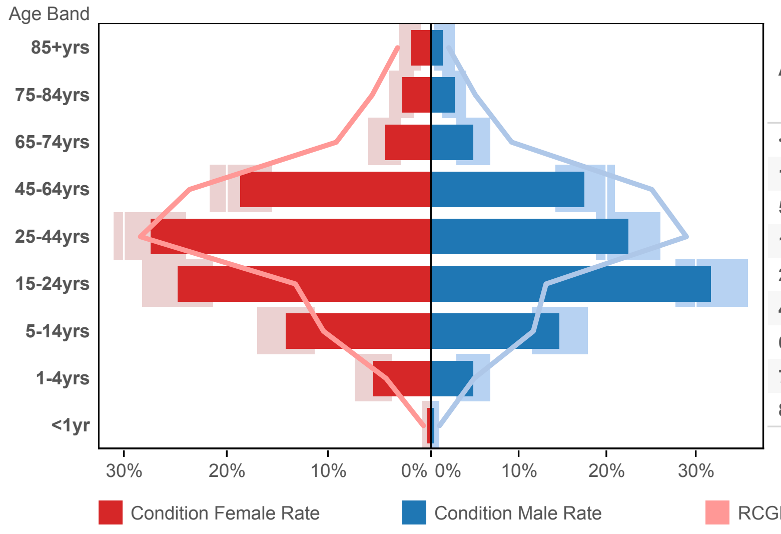

Age Band	Condition Female Rate	Condition Male Rate	Condition Female Population	Condition Male Population
<1yr	0.33	0.41	2.0	2.0
1-4yrs	5.55	4.69	34.0	23.0
5-14yrs	14.19	14.49	87.0	71.0
15-24yrs	24.80	31.84	152.0	156.0
25-44yrs	27.41	22.45	168.0	110.0
45-64yrs	18.60	17.35	114.0	85.0
65-74yrs	4.40	4.69	27.0	23.0
75-84yrs	2.77	2.65	17.0	13.0
85+yrs	1.96	1.43	12.0	7.0

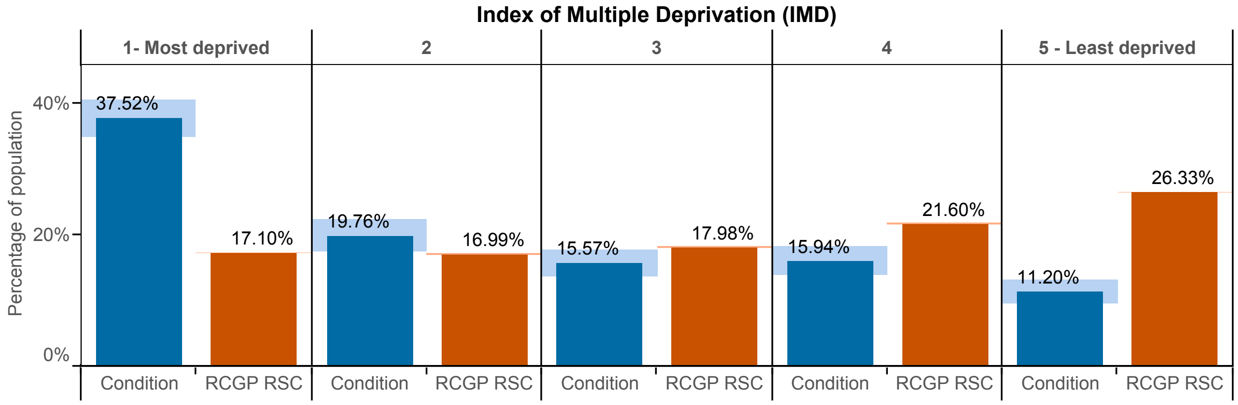

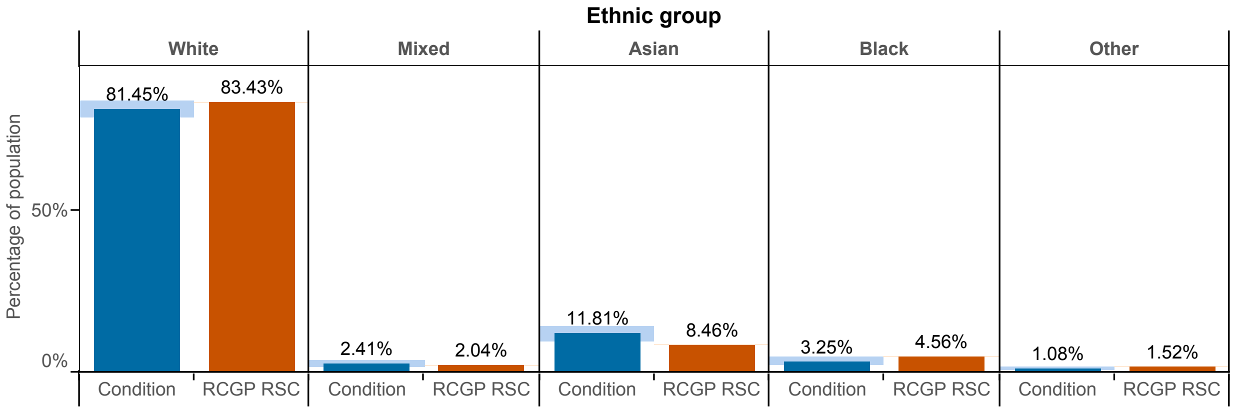

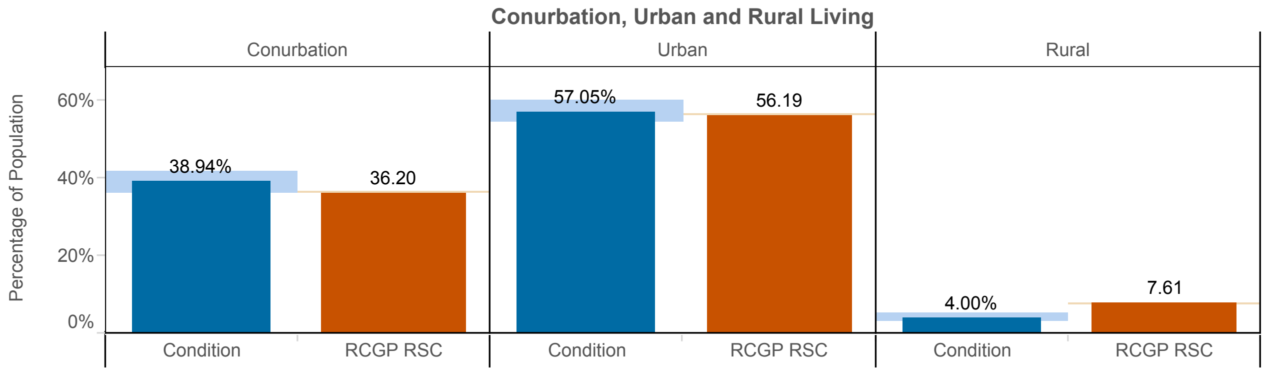

Skin symptoms (ICD10 : R20 - R23)

Age-sex profile

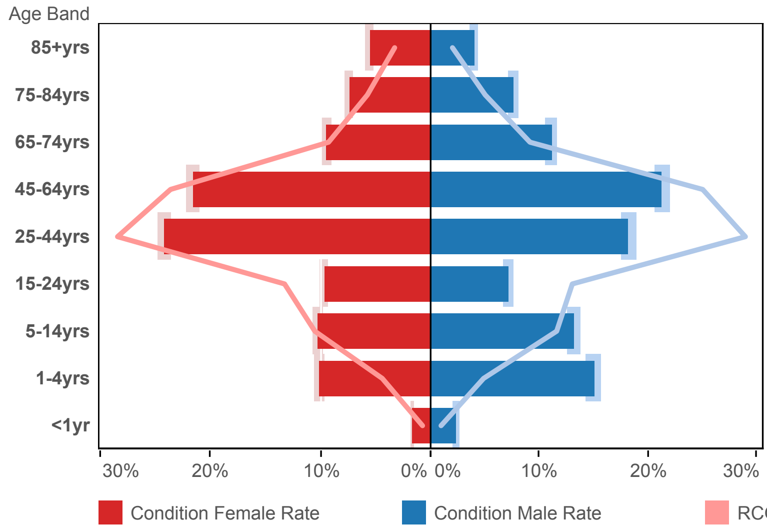

Age Band	Condition Female Rate	Condition Male Rate	Condition Female Population	Condition Male Population
<1yr	1.71	2.36	285	285
1-4yrs	10.16	14.99	1,693	1,812
5-14yrs	10.21	13.22	1,701	1,598
15-24yrs	9.72	7.16	1,620	865
25-44yrs	24.20	18.19	4,033	2,199
45-64yrs	21.62	21.32	3,603	2,577
65-74yrs	9.44	11.15	1,574	1,348
75-84yrs	7.38	7.63	1,230	922
85+yrs	5.56	3.97	926	480

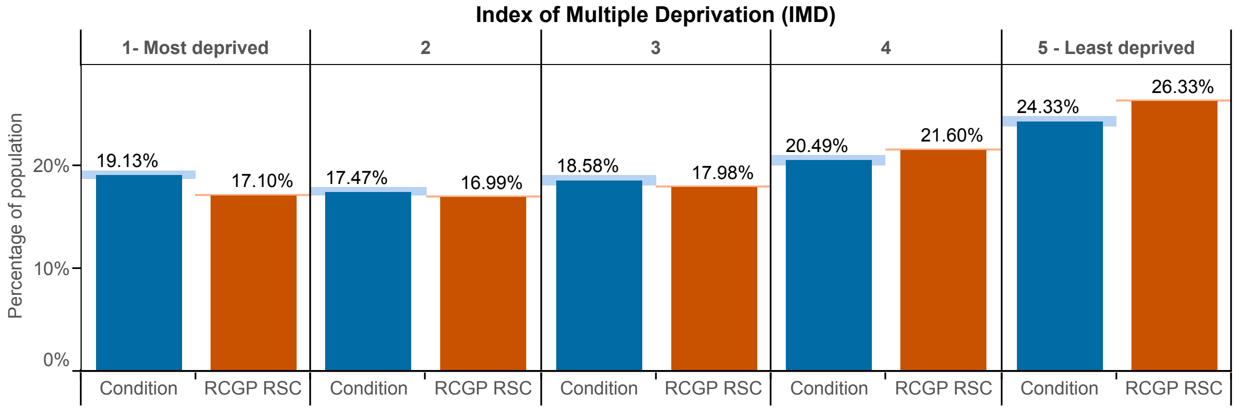

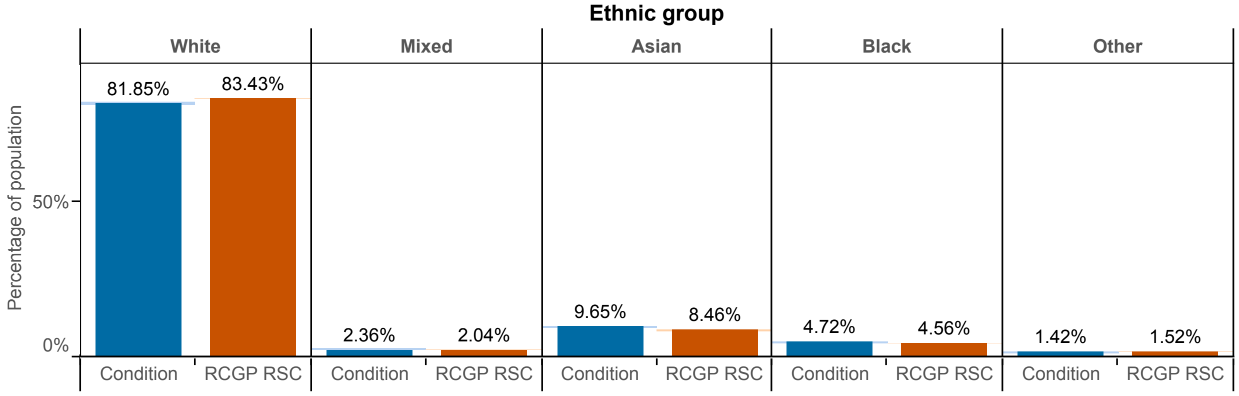

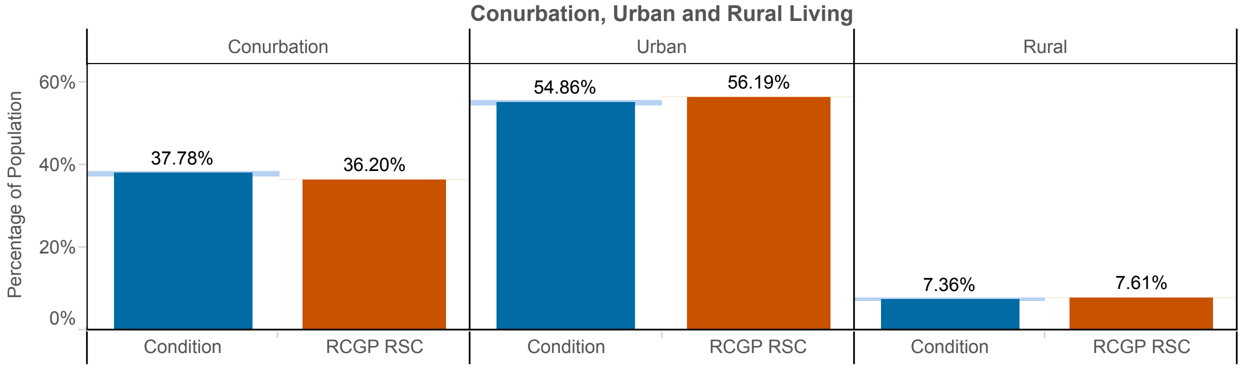

Impetigo (ICD10 - L01)

Age-sex profile

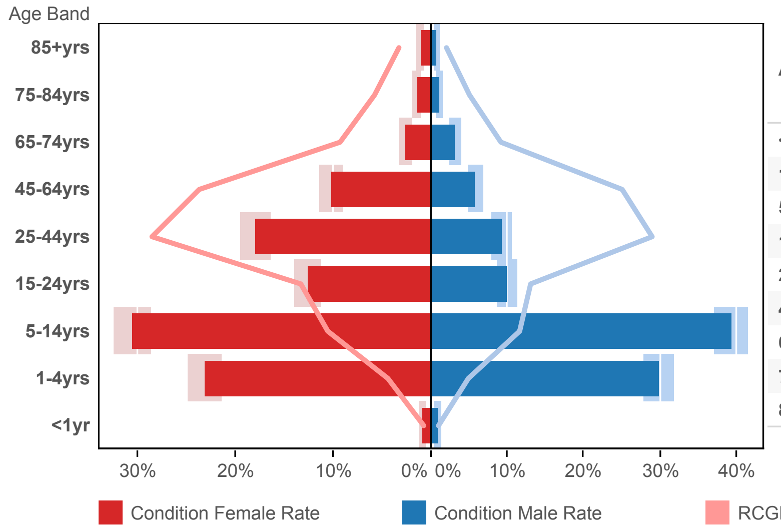

Age Band	Condition Female Rate	Condition Male Rate	Condition Female Population	Condition Male Population
<1yr	0.90	0.85	21.0	17.0
1-4yrs	23.09	29.90	538.0	600.0
5-14yrs	30.39	39.36	708.0	790.0
15-24yrs	12.49	9.87	291.0	198.0
25-44yrs	17.81	9.32	415.0	187.0
45-64yrs	10.13	5.78	236.0	116.0
65-74yrs	2.62	3.09	61.0	62.0
75-84yrs	1.46	1.15	34.0	23.0
85+yrs	1.12	0.70	26.0	14.0

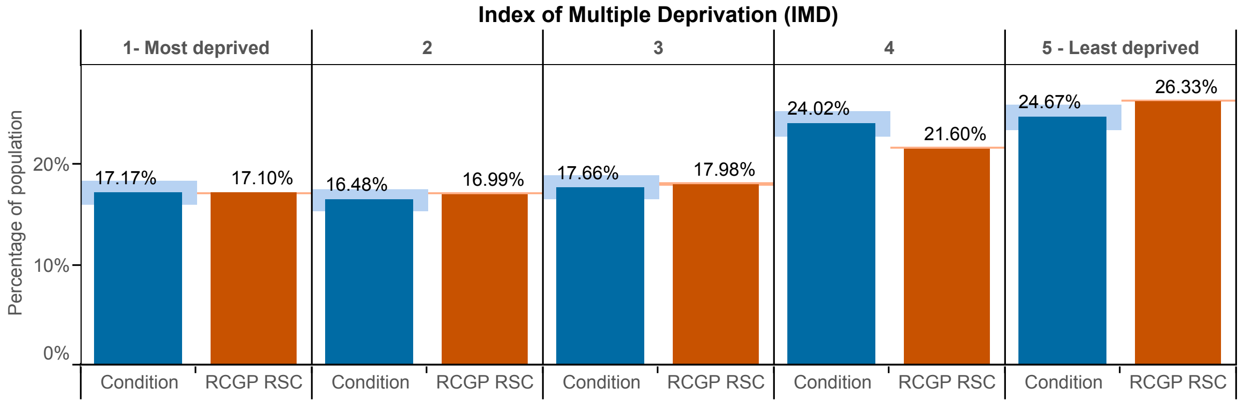

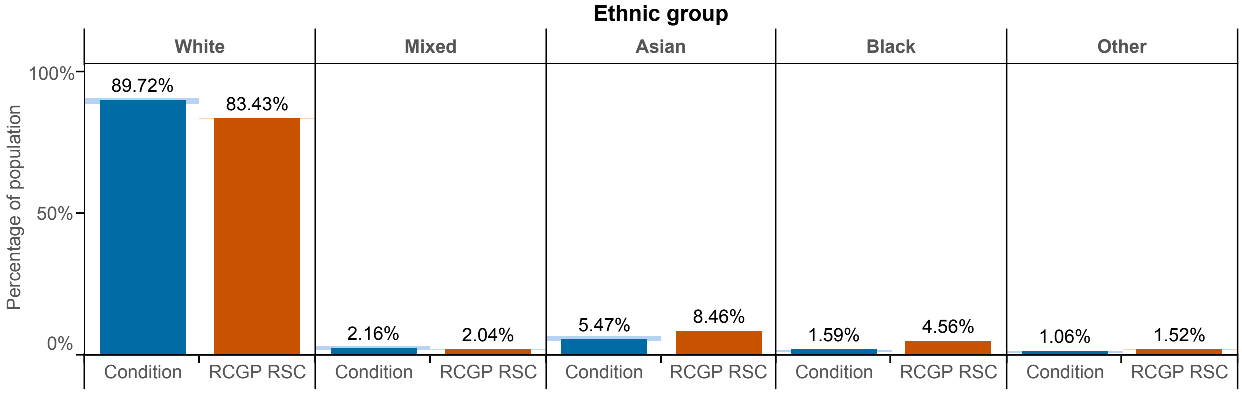

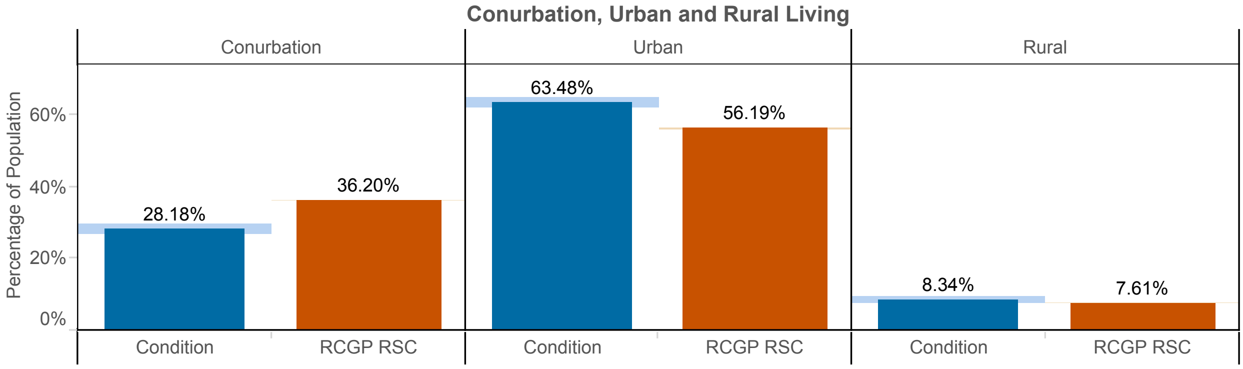

6. Disorders Affective the Nervous System:

Peripheral Neuropathy (ICD10 : G50 - G64; G70 - G72)

Age-sex profile

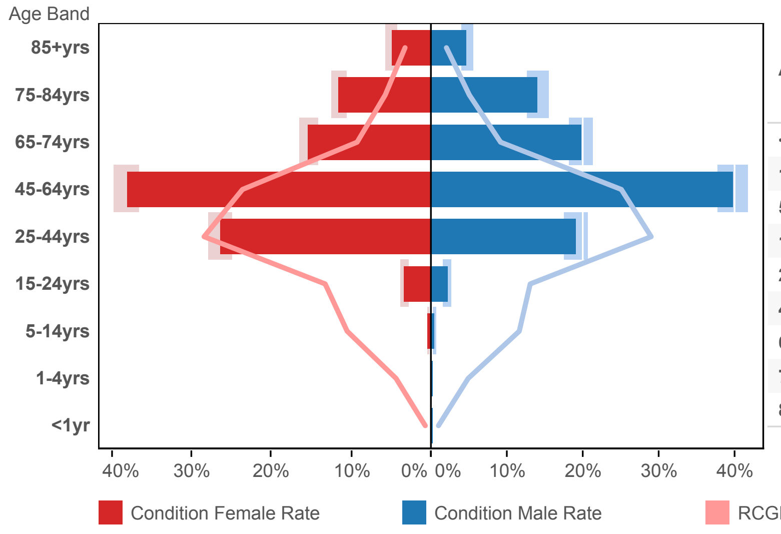

Age Band	Condition Female Rate	Condition Male Rate	Condition Female Population	Condition Male Population
**<1yr**	0.00	0.00	0	0
**1-4yrs**	0.03	0.00	1	0
**5-14yrs**	0.32	0.41	12	10
**15-24yrs**	3.30	2.14	123	52
**25-44yrs**	26.45	19.13	987	464
**45-64yrs**	38.10	39.75	1,422	964
**65-74yrs**	15.35	19.75	573	479
**75-84yrs**	11.52	14.06	430	341
**85+yrs**	4.93	4.74	184	115

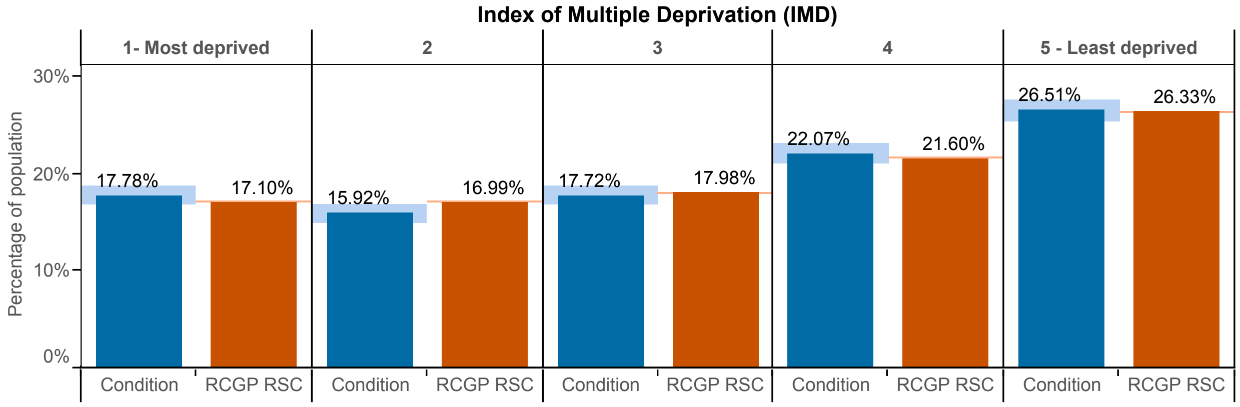

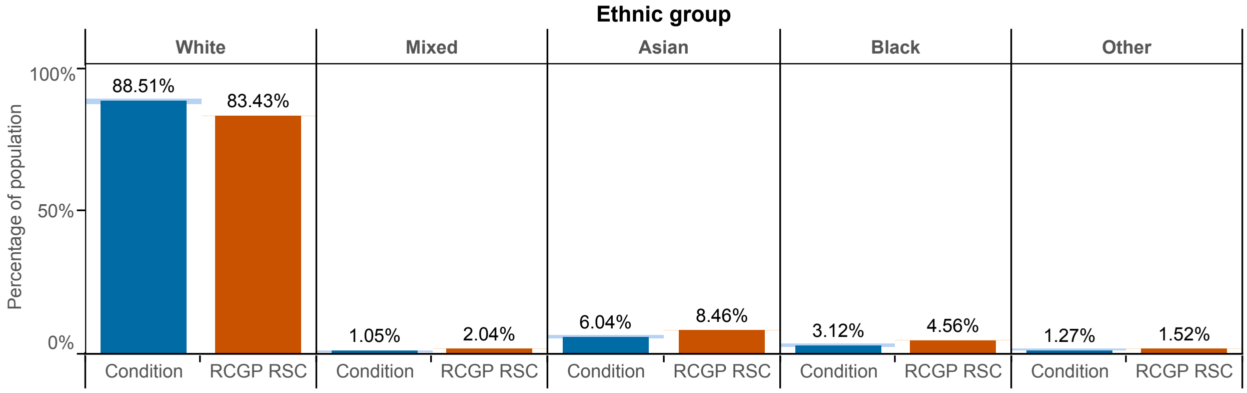

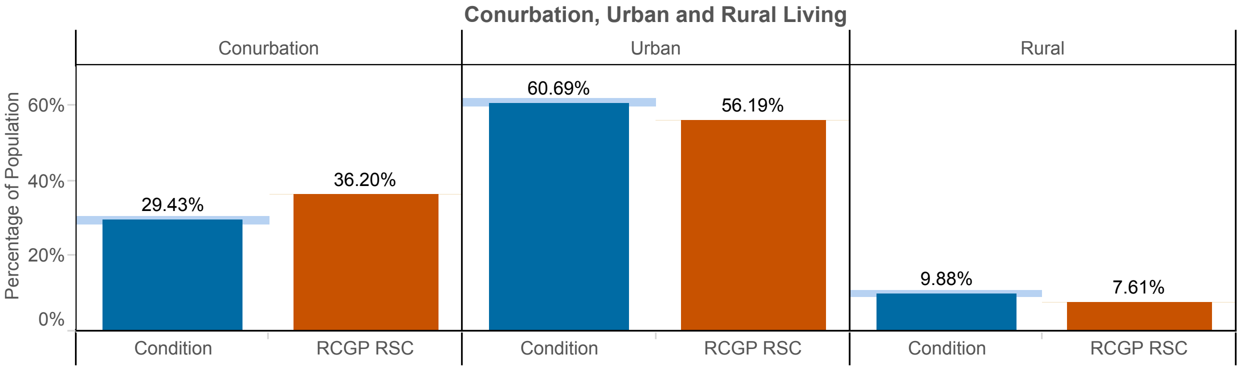

Meningitis and Encephalitis (ICD10 : A170 - A171; A 390; A83 - A85; A87; G00 - G05)

Age-sex profile

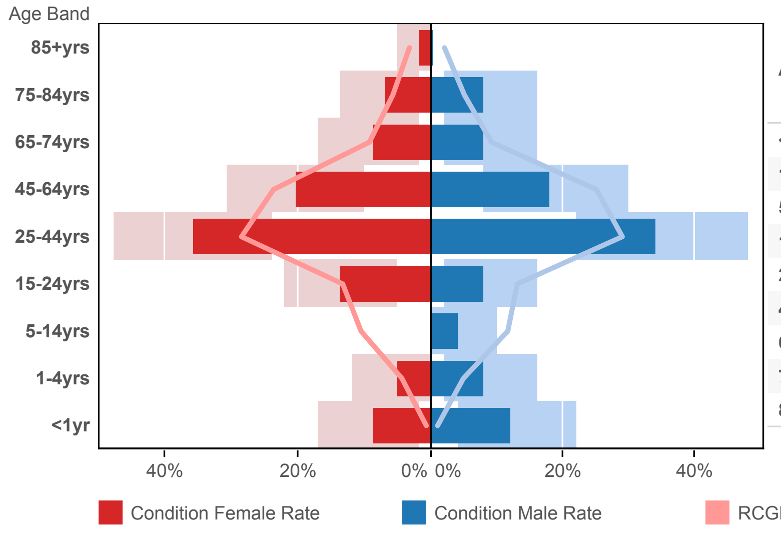

Age Band	Condition Female Rate	Condition Male Rate	Condition Female Population	Condition Male Population
<1yr	8.47	12.00	5.00	6.00
1-4yrs	5.08	8.00	3.00	4.00
5-14yrs	0.00	4.00	0.00	2.00
15-24yrs	13.56	8.00	8.00	4.00
25-44yrs	35.59	34.00	21.00	17.00
45-64yrs	20.34	18.00	12.00	9.00
65-74yrs	8.47	8.00	5.00	4.00
75-84yrs	6.78	8.00	4.00	4.00
85+yrs	1.69	0.00	1.00	0.00

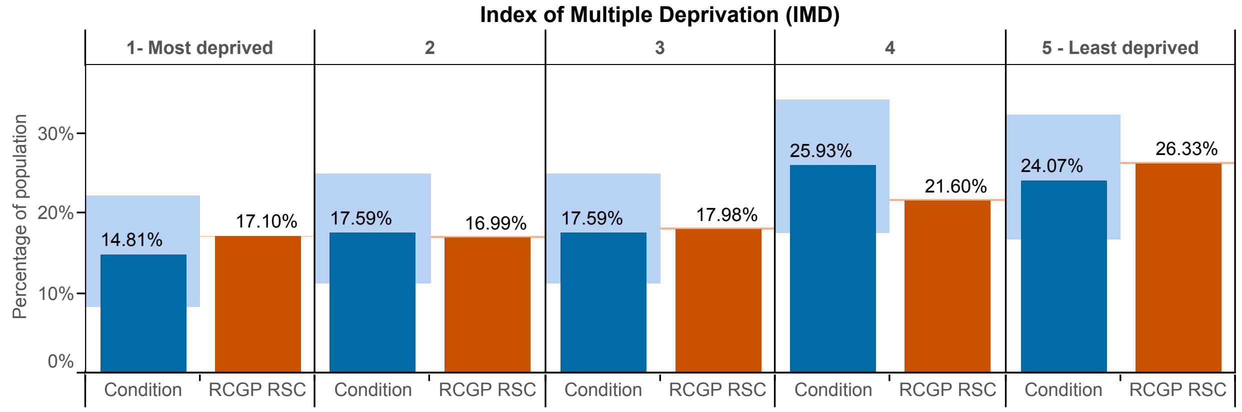

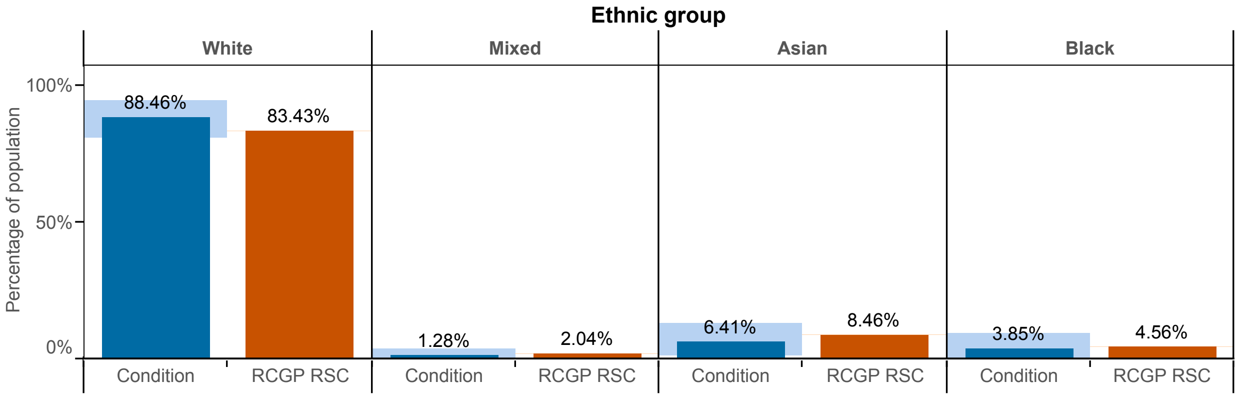

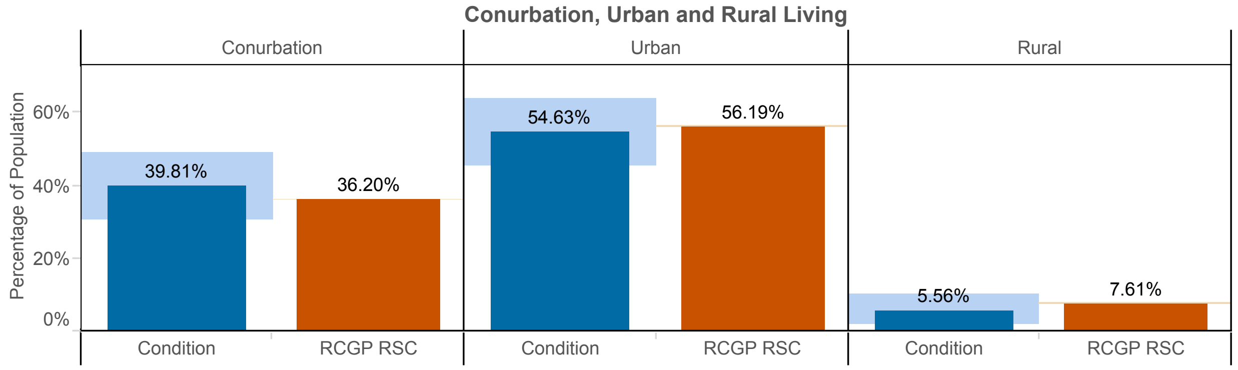

Musculoskeletal symptoms (ICD10 : R25 - R29)

Age-sex profile

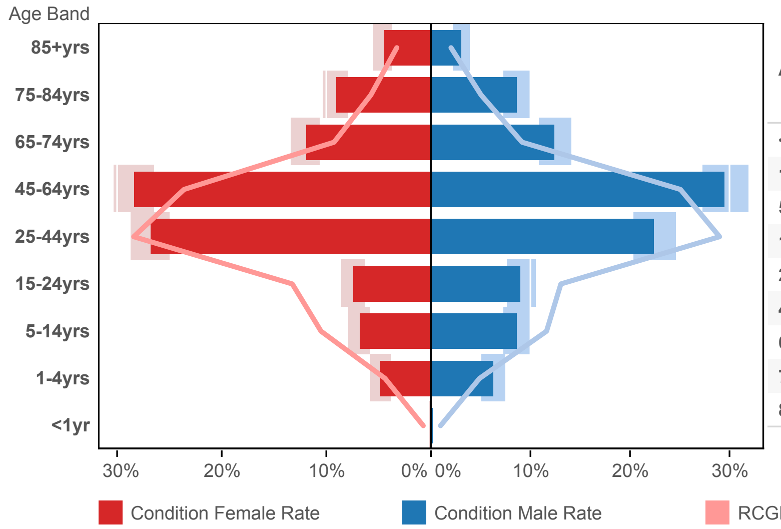

Age Band	Condition Female Rate	Condition Male Rate	Condition Female Population	Condition Male Population
<1yr	0.05	0.00	1.0	0.0
1-4yrs	4.81	6.27	102.0	97.0
5-14yrs	6.80	8.66	144.0	134.0
15-24yrs	7.46	9.04	158.0	140.0
25-44yrs	26.85	22.42	569.0	347.0
45-64yrs	28.36	29.52	601.0	457.0
65-74yrs	11.99	12.40	254.0	192.0
75-84yrs	9.11	8.66	193.0	134.0
85+yrs	4.58	3.04	97.0	47.0

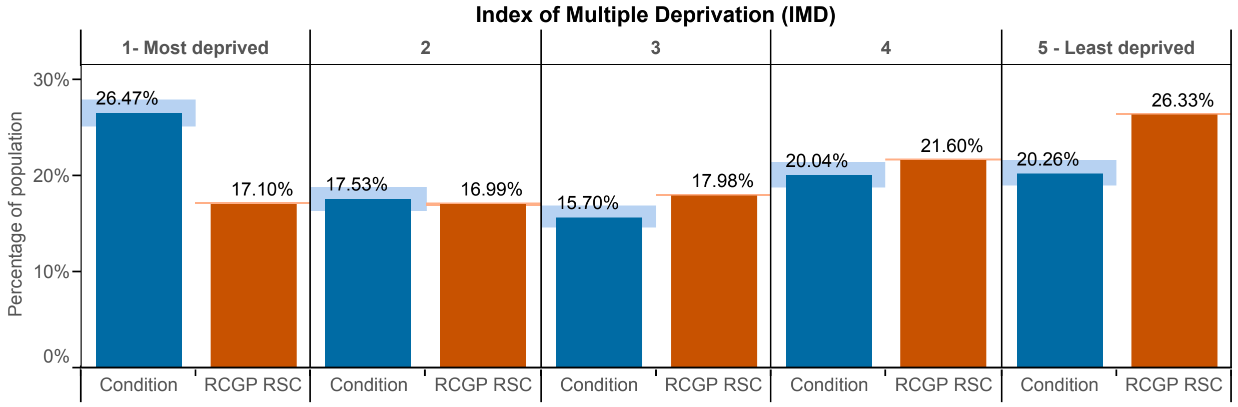

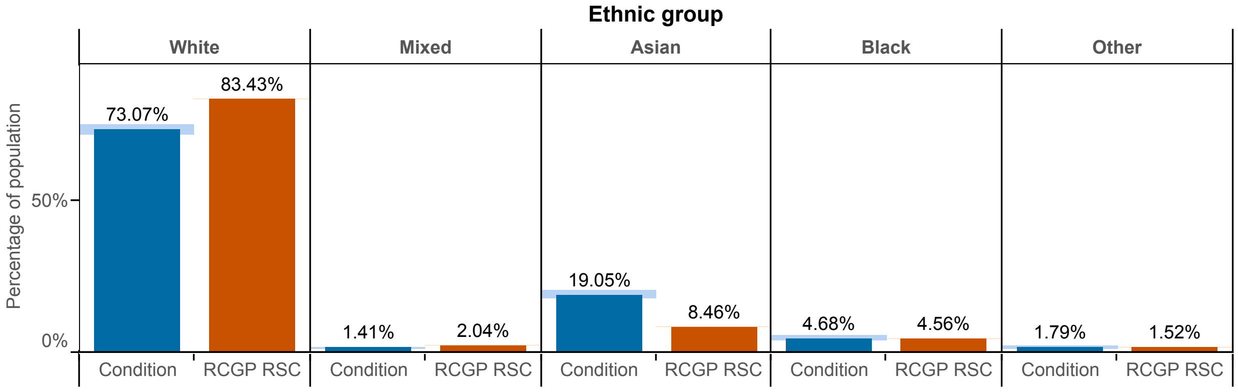

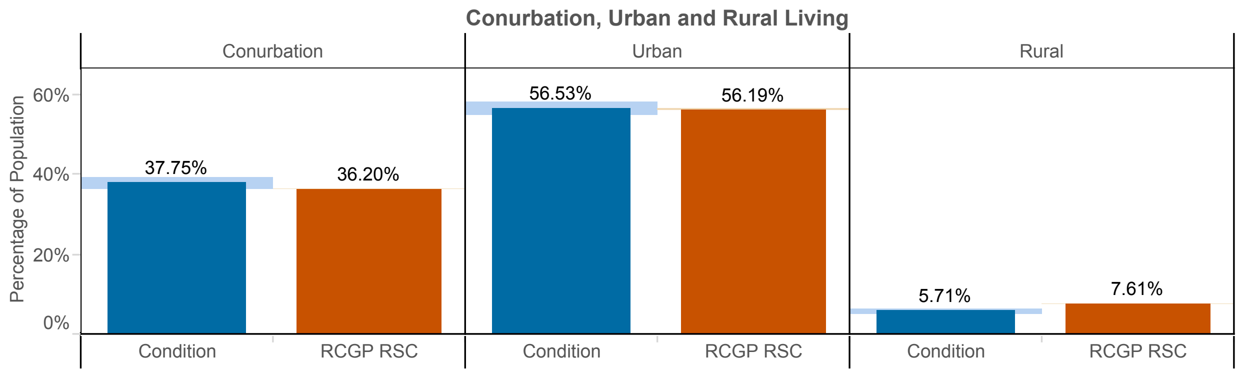

7. Genitourinary System Disorders

Urinary Tract Infections (ICD10 : N30; N390)

Age-sex profile

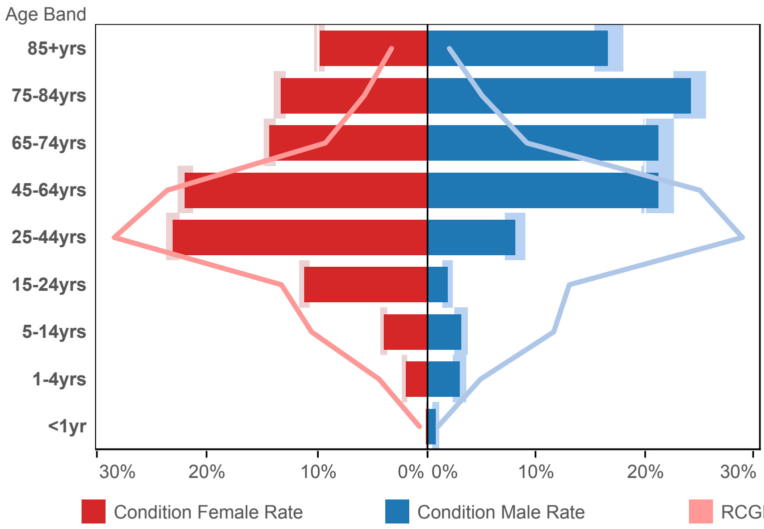

Age Band	Condition Female Rate	Condition Male Rate	Condition Female Population	Condition Male Population
<1yr	0.10	0.73	15	23
1-4yrs	2.05	2.90	305	91
5-14yrs	3.95	3.06	587	96
15-24yrs	11.19	1.85	1,662	58
25-44yrs	23.14	8.12	3,438	255
45-64yrs	21.99	21.20	3,266	666
65-74yrs	14.37	21.30	2,135	669
75-84yrs	13.40	24.16	1,990	759
85+yrs	9.81	16.68	1,457	524

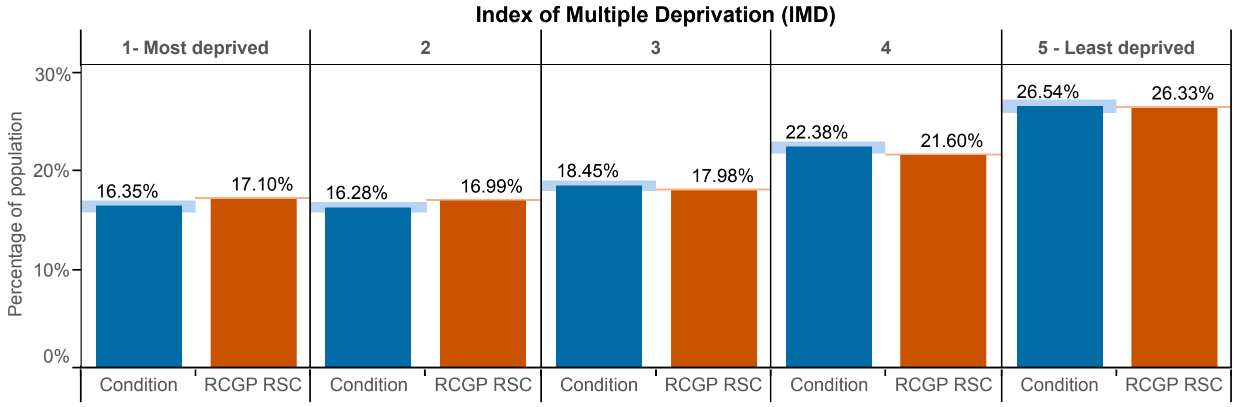

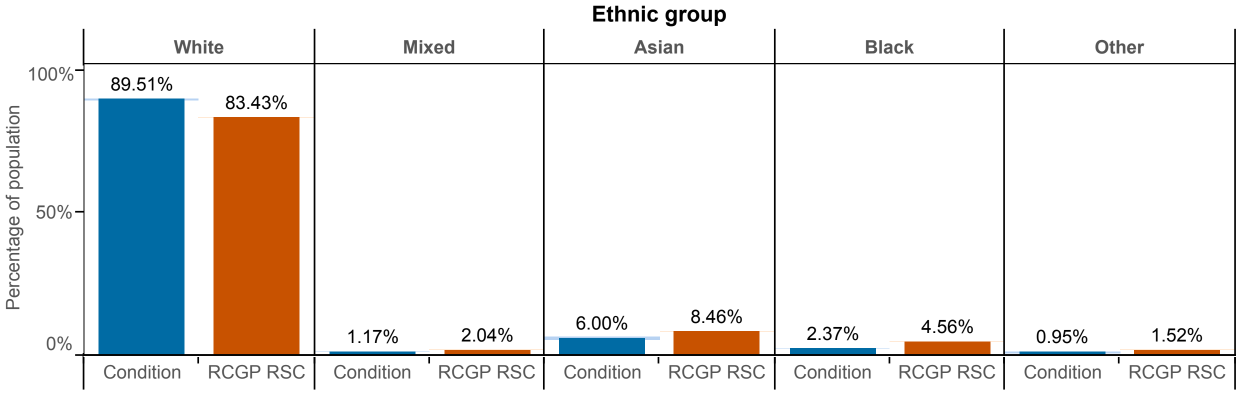

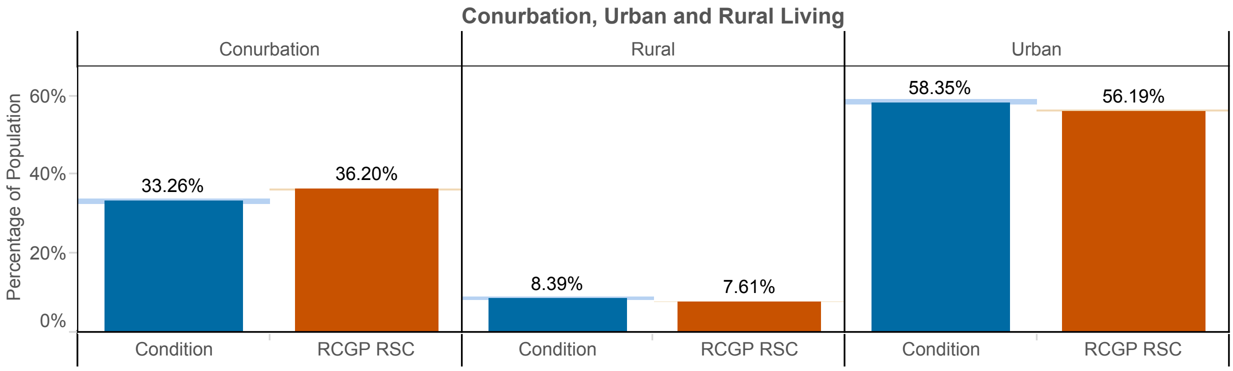

APPENDIX C: Data tables

These data tables show the incidence of monitored diseases by age, gender and region, by 4-week period, quarter and year. We are also including disparities for each individual condition.

1. Water and Food Borne Disorders:

Mean weekly incidence rate per 100,000 Persons.

Intestinal Infectious Diseases (ICD10: A00-A09)

		M										F									
		<1yr	1-4yrs	5-14yrs	15-24yrs	25-44yrs	45-64yrs	65-74yrs	75-84yrs	85+yrs	All Ages	<1yr	1-4yrs	5-14yrs	15-24yrs	25-44yrs	45-64yrs	65-74yrs	75-84yrs	85+yrs	All Ages
**4 weekly**	1	0.00	45.57	11.45	5.45	4.08	4.18	4.77	5.73	4.89	7.33	0.00	36.89	13.99	8.20	5.87	5.56	9.18	4.98	7.22	8.55
	2	0.00	38.18	8.13	7.34	7.01	5.60	7.72	4.89	14.96	8.30	0.00	40.21	12.42	7.62	8.82	8.30	4.42	10.13	8.30	10.10
	3	0.00	35.77	9.15	10.30	7.89	6.01	9.19	8.64	7.11	9.15	36.55	40.09	8.21	6.77	9.19	7.89	6.60	11.77	6.78	9.79
	4	0.00	30.48	7.82	7.00	4.81	5.78	7.28	8.09	4.43	7.41	0.00	33.05	7.45	6.50	6.89	8.33	8.39	15.97	13.04	9.38
	5	10.81	42.06	8.92	4.41	5.22	6.92	7.94	7.59	9.41	8.16	0.00	41.94	11.04	8.42	10.59	6.76	8.84	12.82	9.02	10.72
	6	47.12	61.24	14.44	6.79	5.31	6.94	11.16	8.16	8.69	10.20	11.08	53.63	13.06	9.76	7.88	5.62	9.12	7.75	18.59	10.32
	7	57.61	62.35	15.59	5.18	7.36	5.41	6.01	6.70	18.57	10.50	48.90	61.63	9.78	9.02	10.27	7.22	7.20	9.31	10.97	11.43
	8	68.00	55.50	18.80	7.52	6.13	4.04	7.89	8.28	8.81	10.28	38.93	46.21	13.68	8.05	8.62	7.11	9.89	14.63	8.88	10.96
	9	35.90	33.28	9.26	5.86	5.08	4.41	4.39	4.54	9.14	7.15	27.00	30.57	12.00	8.46	5.98	4.94	9.21	12.36	8.05	8.80
	10	73.67	52.61	14.88	6.52	5.71	4.87	9.39	6.02	2.68	9.49	36.06	41.97	11.80	10.11	7.56	5.36	4.35	8.07	14.74	9.37
	11	42.70	38.90	12.40	6.69	6.19	5.12	7.04	5.40	8.29	8.55	29.69	48.81	16.58	6.23	7.45	5.72	5.14	5.80	14.80	9.83
	12	54.69	50.94	13.91	6.53	5.20	4.25	8.52	7.51	5.70	9.20	51.95	55.05	13.93	6.49	7.73	7.24	6.14	11.44	3.95	10.47
	13	44.35	31.12	6.44	6.38	4.74	5.06	6.65	7.07	4.65	7.18	63.23	37.43	10.99	6.06	7.31	4.56	3.40	7.23	4.16	8.33
**Quarter**	1	0.00	40.28	9.72	7.53	6.16	5.18	7.04	6.37	8.67	8.19	11.25	38.89	11.73	7.58	7.80	7.12	6.92	8.65	7.42	9.41
	2	27.44	45.67	10.55	6.18	5.63	6.41	8.60	7.86	9.08	8.84	13.45	44.97	10.84	8.22	8.39	7.03	8.79	12.32	13.63	10.35
	3	53.02	48.84	15.04	6.07	5.68	4.40	6.93	6.15	9.21	9.06	31.23	44.44	11.94	8.73	8.09	5.79	7.64	11.49	10.18	9.98
	4	51.23	43.05	11.27	6.63	5.41	4.94	7.27	6.72	5.74	8.53	48.70	45.68	13.41	6.75	7.57	5.97	4.95	7.66	7.82	9.52
**Year**	16/17	81.00	821.00	521.00	319.00	631.00	515.00	259.00	133.00	62.00	3342.00	63.00	775.00	504.00	385.00	906.00	628.00	264.00	230.00	126.00	3881.00
**Episodes**	F/N	32.82	44.48	11.63	6.59	5.72	5.25	7.48	6.80	8.19	8.66	25.92	43.52	11.96	7.83	7.97	6.49	7.11	10.08	9.84	9.82

Mean weekly incidence rate per 100,000 Persons.

Intestinal Infectious Diseases (ICD10: A00-A09)

			M										F									
			<1yr	1-4yrs	5-14yrs	15-24yrs	25-44yrs	45-64yrs	65-74yrs	75-84yrs	85+yrs	All Ages	<1yr	1-4yrs	5-14yrs	15-24yrs	25-44yrs	45-64yrs	65-74yrs	75-84yrs	85+yrs	All Ages
**North**	Quarter	1	0.00	56.75	13.31	8.96	9.95	7.43	11.69	6.63	12.24	11.51	0.00	59.62	15.55	9.46	10.31	9.34	9.02	13.13	12.52	12.54
		2	88.98	69.23	15.24	6.74	7.89	8.39	11.21	8.73	16.83	12.09	30.58	61.12	11.91	9.37	11.10	10.48	10.51	16.24	18.51	13.36
		3	112.68	61.57	17.98	6.63	6.29	5.82	9.74	5.40	12.68	10.79	60.65	66.33	14.91	7.91	9.18	8.16	10.71	17.56	14.89	12.68
		4	65.64	52.67	10.59	4.96	4.96	5.22	8.31	8.50	14.98	8.79	62.12	53.28	15.64	4.87	9.33	6.09	6.64	5.99	14.54	10.25
	Year	16/17	41.00	312.00	188.00	117.00	235.00	217.00	128.00	49.00	32.00	1,319.00	25.00	293.00	183.00	142.00	311.00	269.00	122.00	107.00	62.00	1,514.00
	Episodes	F/N	67.24	60.23	14.30	6.82	7.28	6.74	10.26	7.34	14.23	10.82	38.19	60.11	14.45	7.93	10.00	8.56	9.25	13.29	15.18	12.23
**South**	Quarter	1	0.00	32.62	5.67	9.01	5.33	3.37	4.94	8.45	1.98	6.59	0.00	36.36	5.21	7.99	8.19	7.15	4.83	5.94	2.16	7.99
		2	0.00	29.93	6.00	6.62	4.86	4.45	5.73	6.92	9.15	6.52	23.22	35.13	3.69	6.28	7.54	5.26	5.94	8.91	9.44	7.58
		3	34.85	39.32	7.36	4.70	5.27	3.91	5.06	7.35	8.23	6.92	33.59	32.76	5.14	7.70	7.33	5.33	3.42	5.48	10.10	7.41
		4	38.04	39.19	9.83	6.36	3.85	4.09	3.91	8.11	4.90	7.08	58.35	38.76	10.62	6.54	5.63	4.36	6.93	7.74	6.76	8.07
	Year	16/17	14.00	179.00	98.00	99.00	139.00	118.00	58.00	48.00	15.00	768.00	20.00	175.00	78.00	107.00	204.00	160.00	65.00	54.00	32.00	895.00
	Episodes	F/N	17.88	35.16	7.19	6.67	4.83	3.96	4.92	7.69	6.12	6.77	28.68	35.74	6.12	7.11	7.18	5.52	5.29	7.05	7.16	7.76
**London**	Quarter	1	0.00	45.88	16.97	7.10	4.51	6.91	6.60	5.87	11.43	9.55	44.98	33.26	19.95	8.08	8.84	8.73	6.88	10.67	2.94	11.33
		2	20.77	57.67	15.47	7.18	4.40	6.74	12.99	8.77	0.00	10.29	0.00	54.11	18.42	11.51	8.61	6.73	11.43	15.16	22.08	12.82
		3	51.50	57.79	23.34	7.07	6.23	4.78	7.77	6.01	5.21	11.55	14.79	55.55	22.02	9.48	9.53	5.96	12.55	12.28	6.34	13.07
		4	34.87	44.83	19.61	9.01	6.76	6.11	10.76	3.93	0.00	11.05	49.51	53.03	20.44	11.45	8.61	8.66	3.71	11.80	3.20	12.99
	Year	16/17	17.00	240.00	185.00	65.00	175.00	104.00	36.00	13.00	3.00	838.00	14.00	225.00	191.00	95.00	300.00	119.00	37.00	34.00	12.00	1,027.00
	Episodes	F/N	26.67	51.66	18.78	7.58	5.45	6.15	9.60	6.19	4.08	10.60	26.81	49.08	20.17	10.15	8.89	7.50	8.69	12.53	8.89	12.56
**Midlands And East**	Quarter	1	0.00	25.87	2.93	5.03	4.83	3.01	4.92	4.52	9.04	5.12	0.00	26.33	6.21	4.80	3.86	3.26	6.97	4.87	12.04	5.77
		2	0.00	25.83	5.51	4.16	5.36	6.08	4.49	7.04	10.36	6.47	0.00	29.52	9.35	5.74	6.33	5.67	7.26	8.97	4.48	7.63
		3	13.06	36.68	11.48	5.86	4.92	3.09	5.16	5.83	10.70	6.97	15.89	23.15	5.68	9.82	6.33	3.73	3.87	10.65	9.39	6.76
		4	66.37	35.51	5.08	6.17	6.10	4.33	6.08	6.36	3.06	7.22	24.81	37.63	6.94	4.16	6.69	4.78	2.52	5.12	6.80	6.79
	Year	16/17	9.00	90.00	50.00	38.00	82.00	76.00	37.00	23.00	12.00	417.00	4.00	82.00	52.00	41.00	91.00	80.00	40.00	35.00	20.00	445.00
	Episodes	F/N	19.48	30.88	6.23	5.28	5.30	4.16	5.15	5.96	8.33	6.45	9.98	29.16	7.09	6.12	5.81	4.38	5.19	7.43	8.11	6.75

Mean weekly incidence rate per 100,000 Persons.

Non-infective Enteritis and Colitis (ICD10: K50-K52)

		M										F									
		<1yr	1-4yrs	5-14yrs	15-24yrs	25-44yrs	45-64yrs	65-74yrs	75-84yrs	85+yrs	All Ages	<1yr	1-4yrs	5-14yrs	15-24yrs	25-44yrs	45-64yrs	65-74yrs	75-84yrs	85+yrs	All Ages
**4 weekly**	1	0.00	27.51	8.18	8.76	5.28	3.86	5.97	4.29	5.81	6.78	0.00	26.09	9.46	7.75	7.50	6.09	10.82	8.33	6.68	8.54
	2	0.00	28.27	10.27	7.06	7.40	4.53	7.74	8.97	15.30	8.29	0.00	26.68	8.19	6.60	8.11	7.35	11.86	4.77	11.94	8.75
	3	65.79	30.53	7.20	9.07	6.72	8.00	9.73	9.34	2.88	8.76	104.17	35.24	6.81	5.95	8.94	8.41	10.41	12.86	11.39	9.81
	4	81.44	29.40	3.77	4.94	8.72	5.01	5.86	10.94	5.55	7.49	0.00	24.25	2.72	10.77	8.18	8.08	7.79	8.47	8.58	8.51
	5	36.87	35.97	8.56	7.88	9.56	6.47	5.03	4.55	7.33	9.21	16.76	28.77	5.37	8.39	8.66	7.43	8.30	10.47	6.87	9.08
	6	111.87	39.79	9.32	5.95	6.29	7.23	7.10	5.77	12.53	8.94	26.63	30.06	7.57	8.60	7.87	7.78	6.39	9.94	8.64	9.11
	7	68.28	43.32	11.53	4.53	7.39	4.79	7.80	7.30	8.00	9.18	41.73	37.92	13.15	12.34	8.92	5.76	6.08	9.41	6.00	10.30
	8	57.09	41.29	13.14	7.76	6.54	6.74	4.53	6.49	5.84	9.33	62.74	41.66	14.72	10.24	9.08	6.70	11.49	17.92	12.43	11.77
	9	26.34	26.92	7.60	5.51	5.14	5.91	3.51	8.87	13.57	7.15	23.23	25.73	10.28	5.97	5.94	7.00	6.47	14.27	11.19	8.47
	10	62.11	38.78	9.91	6.47	5.75	5.63	3.38	5.61	20.35	8.41	40.84	24.26	9.17	7.85	8.58	7.19	8.13	13.16	13.78	9.49
	11	43.29	35.48	11.74	4.08	6.94	5.64	6.35	9.97	13.99	8.53	62.80	39.72	10.51	6.31	6.00	7.32	6.10	17.98	12.93	9.54
	12	60.82	33.12	12.80	4.30	5.46	5.14	4.06	6.34	12.93	8.04	58.56	38.62	11.37	8.69	8.64	7.66	7.56	10.54	15.87	10.72
	13	72.54	21.22	11.17	5.53	5.03	4.36	5.85	5.12	9.54	7.25	44.28	15.79	6.96	7.38	7.32	5.68	6.23	7.88	9.66	7.63
**Quarter**	1	20.24	28.67	8.52	8.33	6.38	5.34	7.67	7.28	7.83	7.85	32.05	29.09	8.25	6.84	8.13	7.19	11.01	8.63	9.75	9.00
	2	77.44	37.12	8.17	6.27	7.98	6.00	5.94	7.45	8.93	8.71	19.97	29.71	5.85	9.16	8.11	7.38	7.39	8.80	8.18	8.92
	3	51.79	35.88	10.40	5.93	6.15	5.97	4.83	7.22	12.13	8.43	39.81	32.05	12.61	8.96	8.30	7.16	8.59	15.04	10.35	10.30
	4	55.79	30.38	11.43	4.85	5.82	5.08	5.22	6.60	11.98	7.88	54.85	30.35	9.35	7.81	7.34	6.62	6.39	12.42	13.45	9.21
**Year**	16/17	94.00	584.00	416.00	291.00	707.00	522.00	192.00	135.00	67.00	3008.00	67.00	515.00	359.00	380.00	888.00	644.00	286.00	241.00	129.00	3509.00
**Episodes**	F/N	51.81	33.09	9.60	6.34	6.61	5.61	5.92	7.14	10.19	8.23	36.36	30.29	8.95	8.21	7.97	7.09	8.33	11.18	10.39	9.35

Mean weekly incidence rate per 100,000 Persons.

Non-infective Enteritis and Colitis (ICD10: K50-K52)

			M										F									
			<1yr	1-4yrs	5-14yrs	15-24yrs	25-44yrs	45-64yrs	65-74yrs	75-84yrs	85+yrs	All Ages	<1yr	1-4yrs	5-14yrs	15-24yrs	25-44yrs	45-64yrs	65-74yrs	75-84yrs	85+yrs	All Ages
**North**	Quarter	1	0.00	23.46	9.10	6.31	6.85	5.62	6.58	7.84	8.61	7.46	0.00	28.85	5.89	3.74	8.09	6.32	8.45	6.64	12.56	7.73
		2	110.88	24.50	8.21	7.36	5.89	5.05	7.44	6.37	10.31	7.45	9.16	26.12	4.28	6.44	8.15	5.67	6.15	11.55	8.40	7.62
		3	52.64	27.12	10.07	6.58	3.70	5.85	5.16	9.14	9.12	7.13	39.50	34.89	6.36	7.93	6.99	6.14	6.14	10.98	7.93	8.27
		4	51.74	19.25	6.64	3.63	6.22	3.33	2.55	7.22	7.54	5.75	47.61	23.06	8.75	5.02	6.88	5.04	6.28	9.45	11.50	7.49
	Year	16/17	29.00	122.00	112.00	103.00	183.00	160.00	68.00	51.00	20.00	848.00	16.00	137.00	80.00	106.00	233.00	183.00	89.00	78.00	41.00	963.00
	Episodes	F/N	54.89	23.60	8.50	6.00	5.67	4.96	5.47	7.62	8.92	6.96	23.79	28.19	6.28	5.79	7.54	5.79	6.74	9.69	10.06	7.78
**South**	Quarter	1	0.00	28.08	5.96	7.75	4.77	5.28	5.81	6.90	9.76	6.79	128.21	19.74	5.13	7.63	8.02	6.67	9.99	6.47	9.73	8.01
		2	32.24	32.68	6.88	4.08	7.00	4.73	5.52	4.12	10.49	6.98	10.55	26.70	3.01	7.28	7.57	7.23	9.71	8.71	8.60	8.11
		3	53.47	28.44	4.22	5.16	5.06	4.66	3.98	7.86	6.19	6.24	19.08	19.63	5.76	8.08	8.22	5.89	7.50	13.31	15.00	8.40
		4	36.13	29.53	6.86	4.71	5.84	3.93	5.33	6.99	7.87	6.66	55.49	33.67	3.83	7.53	5.58	5.98	5.61	8.91	7.84	7.62
	Year	16/17	19.00	150.00	80.00	79.00	163.00	137.00	60.00	41.00	21.00	750.00	17.00	123.00	55.00	114.00	208.00	189.00	99.00	72.00	45.00	922.00
	Episodes	F/N	30.49	29.74	6.00	5.40	5.69	4.65	5.17	6.42	8.61	6.67	52.52	24.97	4.40	7.62	7.35	6.46	8.23	9.34	10.26	8.04
**London**	Quarter	1	0.00	37.51	14.90	8.56	7.28	5.70	10.92	9.84	9.92	10.11	0.00	34.77	17.67	8.44	9.31	8.01	19.82	13.63	2.96	11.97
		2	38.71	54.92	11.48	9.36	9.69	7.15	8.26	9.27	9.53	11.97	42.55	38.36	11.16	12.32	10.72	9.49	7.02	5.43	8.43	11.94
		3	81.61	47.33	19.10	5.66	8.06	6.07	5.49	4.02	16.36	11.43	63.75	38.40	27.20	12.44	10.43	8.39	15.69	23.16	0.00	14.62
		4	72.85	46.62	22.64	4.92	6.34	8.65	9.69	6.03	5.59	11.89	61.87	33.59	14.09	12.06	9.76	9.18	7.50	16.68	22.45	12.56
	Year	16/17	29.00	218.00	168.00	61.00	251.00	117.00	32.00	15.00	8.00	899.00	24.00	166.00	165.00	107.00	340.00	139.00	52.00	39.00	11.00	1,043.00
	Episodes	F/N	48.11	46.75	16.92	7.17	7.88	6.90	8.58	7.33	10.34	11.36	42.05	36.32	17.41	11.34	10.07	8.78	12.40	14.55	8.46	12.76
**Midlands And East**	Quarter	1	80.97	25.65	4.12	10.71	6.61	4.75	7.38	4.55	3.02	7.03	0.00	32.98	4.33	7.55	7.10	7.77	5.79	7.76	13.74	8.26
		2	127.94	36.38	6.10	4.27	9.36	7.06	2.55	10.03	5.39	8.45	17.64	27.67	4.94	10.60	5.99	7.13	6.69	9.51	7.28	8.00
		3	19.43	40.64	8.23	6.31	7.78	7.32	4.68	7.85	16.88	8.95	36.92	35.28	11.13	7.37	7.57	8.20	5.02	12.69	18.46	9.92
		4	62.44	26.13	9.57	6.12	4.87	4.42	3.33	6.15	26.90	7.23	54.44	31.07	10.72	6.63	7.13	6.27	6.16	14.66	12.01	9.17
	Year	16/17	17.00	94.00	56.00	48.00	110.00	108.00	32.00	28.00	18.00	511.00	10.00	89.00	59.00	53.00	107.00	133.00	46.00	52.00	32.00	581.00
	Episodes	F/N	73.74	32.28	6.99	6.81	7.20	5.91	4.45	7.20	12.90	7.92	27.07	31.67	7.72	8.09	6.93	7.34	5.93	11.13	12.77	8.82

Mean weekly incidence rate per 100,000 Persons.

Viral Hepatitis (ICD10: B15-B19)

		M										F									
		<1yr	1-4yrs	5-14yrs	15-24yrs	25-44yrs	45-64yrs	65-74yrs	75-84yrs	85+yrs	All Ages	<1yr	1-4yrs	5-14yrs	15-24yrs	25-44yrs	45-64yrs	65-74yrs	75-84yrs	85+yrs	All Ages
**4 weekly**	1	0.00	0.00	0.00	0.00	0.46	0.45	0.30	0.50	0.00	0.30	0.00	0.00	0.00	0.00	0.00	0.25	0.88	0.97	0.00	0.16
	2	0.00	0.00	0.00	0.20	0.35	0.96	0.00	0.00	0.00	0.35	0.00	0.00	0.25	0.38	0.45	0.64	0.00	0.00	0.00	0.35
	3	0.00	0.00	0.00	0.00	0.32	0.40	0.00	0.00	0.00	0.22	0.00	0.00	0.00	0.00	0.10	0.00	0.00	0.00	0.00	0.04
	4	0.00	0.00	0.00	0.20	0.55	0.91	0.28	0.00	0.00	0.46	0.00	0.00	0.00	0.00	0.00	0.43	0.00	0.00	0.00	0.10
	5	0.00	0.00	0.00	0.00	0.95	0.49	0.27	0.00	0.00	0.42	0.00	0.00	0.44	0.00	0.19	0.29	0.00	0.41	0.00	0.23
	6	0.00	0.00	0.00	0.00	0.88	0.30	0.43	0.00	0.00	0.34	0.00	0.00	0.00	0.00	0.75	0.00	0.76	0.00	0.00	0.24
	7	0.00	0.00	0.00	0.18	0.51	0.47	0.28	1.58	0.00	0.41	0.00	0.00	0.00	0.00	0.30	0.11	0.00	0.00	0.00	0.10
	8	0.00	0.00	0.00	0.00	0.32	0.60	0.00	0.00	0.00	0.27	0.00	0.00	0.00	0.54	0.20	0.00	0.00	1.30	0.00	0.24
	9	0.00	0.00	0.00	0.00	0.21	0.47	0.00	0.00	0.00	0.17	5.17	0.00	0.00	0.34	0.21	0.11	0.00	0.00	0.00	0.16
	10	0.00	0.00	0.00	0.00	1.00	0.81	0.25	1.63	0.00	0.53	0.00	0.00	0.00	0.00	0.59	0.40	0.80	0.44	0.00	0.34
	11	0.00	0.00	0.00	0.21	0.91	0.57	0.25	0.00	0.00	0.50	0.00	0.00	0.00	0.19	0.10	0.58	0.26	0.00	0.00	0.21
	12	0.00	0.00	0.00	0.19	0.61	0.47	0.25	0.00	0.00	0.36	0.00	0.00	0.00	0.00	0.30	0.47	0.00	0.00	0.00	0.21
	13	0.00	0.00	0.00	0.37	0.90	0.79	0.00	0.00	0.00	0.57	0.00	0.00	0.00	0.00	0.51	0.70	0.00	1.21	0.00	0.33
**Quarter**	1	0.00	0.00	0.00	0.06	0.38	0.59	0.11	0.19	0.00	0.29	0.00	0.00	0.08	0.12	0.17	0.29	0.34	0.37	0.00	0.18
	2	0.00	0.00	0.00	0.06	0.77	0.57	0.36	0.45	0.00	0.42	0.00	0.00	0.13	0.00	0.32	0.24	0.22	0.12	0.00	0.18
	3	0.00	0.00	0.00	0.06	0.53	0.54	0.08	0.00	0.00	0.31	1.59	0.00	0.00	0.27	0.34	0.16	0.00	0.40	0.00	0.21
	4	0.00	0.00	0.00	0.23	0.74	0.66	0.15	0.50	0.00	0.47	0.00	0.00	0.00	0.06	0.28	0.54	0.32	0.51	0.00	0.25
**Year**	16/17	0.00	0.00	0.00	7.00	68.00	53.00	8.00	3.00	0.00	139.00	1.00	0.00	2.00	5.00	31.00	29.00	5.00	7.00	0.00	80.00
**Episodes**	F/N	0.00	0.00	0.00	0.10	0.61	0.59	0.18	0.29	0.00	0.38	0.39	0.00	0.05	0.11	0.28	0.30	0.22	0.35	0.00	0.21

Mean weekly incidence rate per 100,000 Persons.

Viral Hepatitis (ICD10: B15-B19)

			M										F									
			<1yr	1-4yrs	5-14yrs	15-24yrs	25-44yrs	45-64yrs	65-74yrs	75-84yrs	85+yrs	All Ages	<1yr	1-4yrs	5-14yrs	15-24yrs	25-44yrs	45-64yrs	65-74yrs	75-84yrs	85+yrs	All Ages
**North**	Quarter	1	0.00	0.00	0.00	0.25	0.26	0.12	0.00	0.00	0.00	0.14	0.00	0.00	0.31	0.00	0.14	0.40	0.00	0.00	0.00	0.17
		2	0.00	0.00	0.00	0.23	0.83	0.12	0.31	0.00	0.00	0.32	0.00	0.00	0.00	0.00	0.12	0.37	0.00	0.47	0.00	0.16
		3	0.00	0.00	0.00	0.23	0.60	0.13	0.00	0.00	0.00	0.22	0.00	0.00	0.00	0.21	0.25	0.25	0.00	0.00	0.00	0.16
		4	0.00	0.00	0.00	0.45	0.36	1.12	0.00	0.00	0.00	0.45	0.00	0.00	0.00	0.00	0.13	0.25	0.00	0.54	0.00	0.13
	Year	16/17	0.00	0.00	0.00	5.00	17.00	12.00	1.00	0.00	0.00	35.00	0.00	0.00	1.00	1.00	5.00	10.00	0.00	2.00	0.00	19.00
	Episodes	F/N	0.00	0.00	0.00	0.29	0.52	0.37	0.08	0.00	0.00	0.28	0.00	0.00	0.08	0.05	0.16	0.32	0.00	0.26	0.00	0.15
**South**	Quarter	1	0.00	0.00	0.00	0.00	0.00	0.49	0.45	0.77	0.00	0.22	0.00	0.00	0.00	0.00	0.16	0.00	0.40	0.00	0.00	0.08
		2	0.00	0.00	0.00	0.00	0.67	0.55	0.63	0.00	0.00	0.38	0.00	0.00	0.00	0.00	0.38	0.13	0.00	0.00	0.00	0.13
		3	0.00	0.00	0.00	0.00	0.39	0.65	0.31	0.00	0.00	0.30	6.36	0.00	0.00	0.00	0.25	0.11	0.00	1.60	0.00	0.23
		4	0.00	0.00	0.00	0.49	0.64	0.60	0.62	0.00	0.00	0.45	0.00	0.00	0.00	0.23	0.25	0.73	0.32	0.00	0.00	0.31
	Year	16/17	0.00	0.00	0.00	2.00	13.00	17.00	6.00	1.00	0.00	39.00	1.00	0.00	0.00	1.00	8.00	8.00	2.00	3.00	0.00	23.00
	Episodes	F/N	0.00	0.00	0.00	0.12	0.43	0.57	0.51	0.19	0.00	0.34	1.56	0.00	0.00	0.06	0.26	0.24	0.18	0.39	0.00	0.19
**London**	Quarter	1	0.00	0.00	0.00	0.00	0.67	1.50	0.00	0.00	0.00	0.60	0.00	0.00	0.00	0.47	0.37	0.52	0.96	1.50	0.00	0.41
		2	0.00	0.00	0.00	0.00	0.83	1.37	0.00	1.81	0.00	0.68	0.00	0.00	0.00	0.00	0.33	0.25	0.87	0.00	0.00	0.23
		3	0.00	0.00	0.00	0.00	0.65	0.72	0.00	0.00	0.00	0.42	0.00	0.00	0.00	0.87	0.61	0.26	0.00	0.00	0.00	0.40
		4	0.00	0.00	0.00	0.00	1.46	0.47	0.00	2.01	0.00	0.74	0.00	0.00	0.00	0.00	0.23	0.74	0.98	1.49	0.00	0.33
	Year	16/17	0.00	0.00	0.00	0.00	29.00	17.00	0.00	2.00	0.00	48.00	0.00	0.00	0.00	3.00	13.00	7.00	3.00	2.00	0.00	28.00
	Episodes	F/N	0.00	0.00	0.00	0.00	0.90	1.02	0.00	0.97	0.00	0.61	0.00	0.00	0.00	0.33	0.38	0.44	0.71	0.73	0.00	0.34
**Midlands And East**	Quarter	1	0.00	0.00	0.00	0.00	0.61	0.25	0.00	0.00	0.00	0.21	0.00	0.00	0.00	0.00	0.00	0.25	0.00	0.00	0.00	0.07
		2	0.00	0.00	0.00	0.00	0.75	0.26	0.49	0.00	0.00	0.31	0.00	0.00	0.50	0.00	0.47	0.21	0.00	0.00	0.00	0.22
		3	0.00	0.00	0.00	0.00	0.49	0.64	0.00	0.00	0.00	0.30	0.00	0.00	0.00	0.00	0.24	0.00	0.00	0.00	0.00	0.06
		4	0.00	0.00	0.00	0.00	0.51	0.44	0.00	0.00	0.00	0.25	0.00	0.00	0.00	0.00	0.52	0.43	0.00	0.00	0.00	0.24
	Year	16/17	0.00	0.00	0.00	0.00	9.00	7.00	1.00	0.00	0.00	17.00	0.00	0.00	1.00	0.00	5.00	4.00	0.00	0.00	0.00	10.00
	Episodes	F/N	0.00	0.00	0.00	0.00	0.59	0.39	0.13	0.00	0.00	0.27	0.00	0.00	0.13	0.00	0.31	0.22	0.00	0.00	0.00	0.15

2. Environmentally Sensitive Disorders:

Mean weekly incidence rate per 100,000 Persons.

Asthma (ICD10: J45-J46)

		M										F									
		<1yr	1-4yrs	5-14yrs	15-24yrs	25-44yrs	45-64yrs	65-74yrs	75-84yrs	85+yrs	All Ages	<1yr	1-4yrs	5-14yrs	15-24yrs	25-44yrs	45-64yrs	65-74yrs	75-84yrs	85+yrs	All Ages
**4 weekly**	1	0.00	10.02	15.71	5.18	5.53	5.77	6.43	7.36	3.69	7.22	0.00	6.63	14.33	8.69	9.22	9.87	5.64	9.10	4.78	9.31
	2	0.00	11.68	23.84	9.59	9.21	9.38	7.94	5.47	5.60	10.53	0.00	12.08	14.68	14.69	17.64	14.39	14.68	16.69	6.83	14.67
	3	0.00	13.04	19.48	10.14	9.38	8.06	8.37	15.07	4.71	10.45	0.00	7.77	15.72	15.37	15.48	17.80	16.71	16.80	10.94	15.29
	4	0.00	10.94	18.81	9.20	12.36	7.48	10.60	4.38	8.73	10.45	0.00	2.94	7.29	12.38	14.91	16.71	12.80	16.61	9.20	13.21
	5	0.00	11.02	20.66	9.99	8.88	11.22	7.59	9.12	9.55	10.88	0.00	5.01	16.54	17.61	15.70	19.75	15.70	13.28	8.79	16.07
	6	0.00	22.97	30.92	20.71	12.41	14.67	11.40	6.40	9.85	16.19	0.00	14.05	24.54	31.32	23.28	21.21	18.78	24.45	16.18	22.39
	7	0.00	19.38	28.70	13.05	13.77	14.80	10.88	11.27	15.53	15.46	0.00	16.62	23.02	30.49	23.82	27.65	17.03	16.43	16.70	23.44
	8	0.00	21.30	37.04	10.76	12.46	13.59	16.47	13.13	7.27	16.16	0.00	16.36	25.68	21.53	23.70	31.63	26.82	24.73	21.17	24.95
	9	0.00	13.38	17.41	12.86	10.89	17.26	16.28	14.68	4.35	14.00	0.00	4.40	15.66	17.67	22.55	27.95	25.52	19.22	15.92	20.78
	10	8.49	26.94	28.66	11.74	16.04	16.04	14.68	12.22	13.32	17.01	0.00	9.49	18.68	20.31	21.39	30.53	20.56	22.94	10.01	22.12
	11	0.00	12.18	21.77	9.57	11.02	11.64	10.52	7.58	7.29	12.02	0.00	6.55	15.76	14.43	14.88	20.32	22.23	16.72	19.46	16.50
	12	0.00	14.62	25.27	7.19	10.28	10.95	10.81	7.06	4.00	11.72	0.00	12.80	16.41	8.87	17.92	19.82	24.37	15.26	16.04	16.90
	13	0.00	20.11	17.28	8.87	10.68	9.92	9.11	5.84	1.27	11.17	0.00	8.27	8.07	10.70	13.83	15.10	15.52	23.44	2.39	13.05
**Quarter**	1	0.00	11.46	19.37	8.06	7.84	7.59	7.49	9.15	4.59	9.23	0.00	8.66	14.86	12.59	13.74	13.70	11.83	13.81	7.30	12.80
	2	0.00	15.32	24.19	13.18	11.40	11.50	10.43	7.62	10.46	12.86	0.00	8.91	16.63	21.72	18.75	20.38	15.87	18.10	11.52	18.00
	3	2.61	20.42	28.52	12.43	13.43	16.12	15.18	13.13	8.71	16.02	0.00	11.05	21.26	21.45	23.71	30.12	23.27	21.65	17.53	23.22
	4	0.00	16.26	21.28	8.42	10.84	10.75	10.01	6.88	5.00	11.68	0.00	9.04	13.65	12.18	15.17	18.90	20.96	18.26	11.87	15.67
**Year**	16/17	1.00	280.00	1024.00	518.00	1170.00	1114.00	385.00	171.00	42.00	4705.00	0.00	154.00	700.00	835.00	1905.00	1939.00	666.00	394.00	140.00	6733.00
**Episodes**	F/N	0.64	15.85	23.36	10.58	10.89	11.49	10.77	9.16	7.25	12.46	0.00	9.41	16.60	17.07	17.86	20.77	17.94	17.96	12.05	17.43

Mean weekly incidence rate per 100,000 Persons.

Asthma (ICD10: J45 - J46)

			M										F									
			<1yr	1-4yrs	5-14yrs	15-24yrs	25-44yrs	45-64yrs	65-74yrs	75-84yrs	85+yrs	All Ages	<1yr	1-4yrs	5-14yrs	15-24yrs	25-44yrs	45-64yrs	65-74yrs	75-84yrs	85+yrs	All Ages
**North**	Quarter	1	0.00	8.85	18.18	8.24	7.89	8.94	7.97	6.10	1.66	9.14	0.00	6.80	12.44	14.02	13.77	13.00	12.38	14.16	5.77	12.79
		2	0.00	25.88	27.94	14.88	12.50	10.22	12.22	9.91	3.49	14.12	0.00	6.24	15.28	24.04	17.64	20.23	16.52	19.22	6.51	18.17
		3	0.00	23.10	23.15	13.25	13.71	12.15	16.11	12.11	1.79	14.53	0.00	18.18	22.95	19.76	19.90	25.23	21.92	21.52	9.97	21.38
		4	0.00	14.63	18.11	10.47	11.13	9.78	13.43	9.15	3.73	11.54	0.00	7.38	17.03	10.59	13.55	17.33	21.31	18.48	11.34	15.16
	Year	16/17	0.00	95.00	289.00	203.00	368.00	331.00	155.00	62.00	6.00	1,509.00	0.00	47.00	215.00	312.00	504.00	597.00	238.00	148.00	34.00	2,095.00
	Episodes	F/N	0.00	18.26	21.96	11.77	11.33	10.27	12.43	9.33	2.68	12.36	0.00	9.59	16.89	17.23	16.24	18.97	18.01	18.36	8.36	16.90
**South**	Quarter	1	0.00	12.84	17.89	4.57	6.50	6.93	6.30	8.53	11.70	8.16	0.00	5.09	12.28	9.55	13.95	12.91	10.62	8.36	8.81	11.63
		2	0.00	15.83	20.34	13.73	8.92	12.07	9.21	4.11	4.64	11.70	0.00	9.36	17.02	19.84	20.58	17.05	15.91	16.88	11.97	17.60
		3	0.00	20.22	28.59	11.67	12.11	16.45	13.86	14.10	8.10	15.65	0.00	5.90	19.49	19.78	23.83	27.36	23.25	20.03	16.19	22.26
		4	0.00	9.27	18.90	8.02	10.04	11.11	10.37	7.03	7.72	10.83	0.00	6.54	13.56	13.04	16.54	17.82	17.83	13.06	13.12	15.33
	Year	16/17	0.00	74.00	290.00	149.00	275.00	355.00	118.00	53.00	19.00	1,333.00	0.00	34.00	198.00	240.00	546.00	565.00	214.00	114.00	55.00	1,966.00
	Episodes	F/N	0.00	14.56	21.41	9.58	9.38	11.65	9.92	8.36	7.98	11.59	0.00	6.77	15.62	15.63	18.76	18.75	16.88	14.63	12.51	16.72
**London**	Quarter	1	0.00	6.41	19.19	13.14	7.93	7.95	7.69	11.75	4.98	9.88	0.00	4.55	13.69	11.73	10.66	16.63	15.60	18.19	5.95	12.39
		2	0.00	8.96	24.14	11.63	9.46	10.70	10.05	8.91	33.69	11.98	0.00	5.76	18.00	20.25	16.28	21.37	19.33	27.30	14.22	17.78
		3	0.00	18.37	34.48	10.75	12.63	17.04	17.53	17.53	10.56	16.67	0.00	10.78	22.20	22.11	20.09	36.59	26.20	29.30	28.84	23.87
		4	0.00	17.81	27.81	11.24	11.96	11.16	9.65	1.93	5.49	13.46	0.00	9.40	14.49	13.03	15.49	21.24	23.60	24.05	9.72	16.30
	Year	16/17	0.00	60.00	260.00	100.00	336.00	199.00	42.00	21.00	11.00	1,029.00	0.00	35.00	163.00	158.00	529.00	382.00	90.00	67.00	19.00	1,443.00
	Episodes	F/N	0.00	12.81	26.36	11.69	10.48	11.69	11.21	10.01	14.06	12.98	0.00	7.59	17.11	16.85	15.64	23.91	21.15	24.76	14.67	17.59
**Midlands And East**	Quarter	1	0.00	17.74	22.24	6.30	9.06	6.53	8.00	10.24	0.00	9.75	0.00	18.19	21.03	15.06	16.57	12.26	8.70	14.51	8.69	14.38
		2	0.00	10.61	24.36	12.49	14.73	13.01	10.23	7.54	0.00	13.65	0.00	14.30	16.23	22.75	20.50	22.89	11.72	9.01	13.39	18.46
		3	10.45	20.00	27.85	14.07	15.27	18.84	13.22	8.76	14.39	17.24	0.00	9.34	20.38	24.14	31.01	31.28	21.73	15.77	15.10	25.36
		4	0.00	23.32	20.28	3.96	10.23	10.96	6.57	9.40	3.06	10.88	0.00	12.85	9.53	12.09	15.09	19.23	21.11	17.45	13.29	15.90
	Year	16/17	1.00	51.00	185.00	66.00	191.00	229.00	70.00	35.00	6.00	834.00	0.00	38.00	124.00	125.00	326.00	395.00	124.00	65.00	32.00	1,229.00
	Episodes	F/N	2.56	17.78	23.69	9.27	12.37	12.35	9.52	8.96	4.28	12.89	0.00	13.68	16.78	18.59	20.79	21.44	15.74	14.09	12.63	18.52

Mean weekly incidence rate per 100,000 Persons.

Disorders of Conjunctiva (ICD10: H10-H13)

		M										F									
		<1yr	1-4yrs	5-14yrs	15-24yrs	25-44yrs	45-64yrs	65-74yrs	75-84yrs	85+yrs	All Ages	<1yr	1-4yrs	5-14yrs	15-24yrs	25-44yrs	45-64yrs	65-74yrs	75-84yrs	85+yrs	All Ages
**4 weekly**	1	0.00	200.77	30.27	7.65	9.33	11.12	20.82	23.59	49.92	23.45	0.00	155.05	33.50	11.46	18.39	18.11	33.74	32.69	52.87	28.66
	2	3748.92	159.06	39.79	11.94	10.29	14.22	25.02	34.96	87.12	25.83	781.25	124.43	32.04	18.25	20.55	23.49	27.99	37.28	66.00	30.07
	3	799.65	151.58	33.67	6.88	12.24	15.11	33.49	43.74	51.76	25.75	1036.68	116.53	24.71	11.13	19.97	24.24	36.20	49.35	59.64	29.95
	4	535.34	91.43	20.50	10.23	8.16	10.25	26.32	44.07	83.07	20.10	502.53	72.11	18.25	11.64	12.91	26.01	30.35	43.28	69.68	25.50
	5	516.78	67.42	24.21	8.85	8.65	11.28	31.72	28.19	51.07	19.07	438.49	56.25	14.29	9.46	13.84	17.73	33.56	41.97	59.82	22.50
	6	489.21	83.92	20.56	7.90	9.63	11.91	23.23	48.02	69.02	20.57	497.34	91.60	20.02	12.69	15.42	21.24	23.26	36.30	68.50	25.66
	7	363.22	119.94	22.97	6.18	8.91	12.72	16.38	30.32	42.24	20.02	364.32	115.09	16.82	17.85	15.13	17.86	24.12	38.25	80.26	25.91
	8	203.78	189.41	17.88	9.21	6.81	11.00	19.15	31.10	40.48	21.74	384.87	174.23	24.74	12.37	14.55	18.25	29.89	29.34	66.45	29.01
	9	388.90	203.82	17.67	8.11	7.73	14.66	18.71	26.31	56.27	25.01	343.67	169.87	21.12	12.40	14.80	21.06	23.06	43.43	66.68	29.61
	10	475.89	298.16	20.48	7.29	8.85	12.89	28.55	38.10	72.79	32.03	456.71	251.96	33.09	15.67	17.22	27.01	27.47	42.26	82.30	38.94
	11	458.17	283.76	22.42	7.97	10.97	12.78	23.32	19.57	90.04	32.02	417.50	243.61	20.78	19.61	19.02	24.27	27.29	51.86	80.25	37.69
	12	560.01	198.88	28.58	10.04	9.56	12.12	18.82	34.49	66.78	28.70	401.57	208.52	32.53	12.53	18.91	22.15	26.54	44.90	61.99	35.68
	13	446.82	127.03	20.13	7.82	10.15	15.34	22.99	36.11	49.44	24.89	351.68	115.85	20.20	12.81	17.43	17.54	25.91	32.52	62.12	27.46
**Quarter**	1	1399.56	172.80	34.24	8.73	10.52	13.30	26.01	33.29	61.93	24.89	559.36	133.78	30.34	13.45	19.54	21.65	32.73	39.23	58.99	29.49
	2	491.20	83.06	22.16	8.17	8.91	11.26	25.30	38.27	61.94	19.70	479.14	79.48	17.36	12.28	14.06	21.12	27.88	39.04	70.24	24.73
	3	344.74	214.76	18.41	8.42	7.92	12.72	21.75	32.70	53.76	25.19	369.71	180.36	23.50	13.92	15.68	21.35	27.33	38.19	70.94	31.04
	4	491.85	207.72	24.00	8.51	9.99	13.60	21.52	29.55	70.67	28.72	393.88	195.30	26.07	15.02	18.18	21.48	26.17	44.11	68.20	34.01
**Year**	16/17	717.00	2945.00	1050.00	382.00	998.00	1187.00	808.00	597.00	408.00	9092.00	621.00	2508.00	980.00	668.00	1803.00	1953.00	1051.00	905.00	836.00	11325.00
**Episodes**	F/N	678.24	167.95	24.66	8.45	9.33	12.69	23.68	33.54	62.07	24.53	451.07	145.95	24.19	13.64	16.81	21.39	28.51	40.12	67.15	29.72

Mean weekly incidence rate per 100,000 Persons.

Disorders of Conjunctiva (ICD10: H10-H13)

			M										F									
			<1yr	1-4yrs	5-14yrs	15-24yrs	25-44yrs	45-64yrs	65-74yrs	75-84yrs	85+yrs	All Ages	<1yr	1-4yrs	5-14yrs	15-24yrs	25-44yrs	45-64yrs	65-74yrs	75-84yrs	85+yrs	All Ages
**North**	Quarter	1	1,243.44	180.58	28.48	7.66	8.86	12.06	21.51	30.47	50.91	22.60	789.22	156.44	24.89	15.17	20.81	16.67	28.57	36.81	55.28	28.08
		2	603.47	79.03	24.43	6.78	9.22	10.44	25.57	29.38	58.99	18.97	657.67	99.51	18.29	13.70	14.19	19.56	26.52	39.22	64.41	25.35
		3	410.60	223.24	19.70	8.66	7.20	13.25	21.10	25.43	52.79	24.79	375.47	196.46	25.62	16.27	14.30	17.31	24.94	38.34	71.26	29.79
		4	576.20	218.80	19.36	7.51	8.75	12.59	23.27	31.60	49.24	27.16	442.80	201.16	15.10	10.72	17.34	16.70	28.09	42.82	74.41	30.56
	Year	16/17	250.00	900.00	303.00	130.00	277.00	388.00	287.00	195.00	119.00	2,849.00	202.00	791.00	265.00	250.00	517.00	554.00	357.00	318.00	270.00	3,524.00
	Episodes	F/N	706.45	173.60	23.02	7.64	8.52	12.05	22.91	29.22	53.10	23.30	568.01	162.19	20.92	13.96	16.61	17.60	27.02	39.30	66.31	28.39
**South**	Quarter	1	1,842.97	168.34	27.91	6.73	10.05	10.91	23.22	33.03	50.49	22.60	757.82	110.70	32.59	14.56	17.88	20.35	32.36	41.60	67.22	28.71
		2	322.16	78.46	15.25	6.82	9.05	8.76	25.77	32.91	61.44	17.46	420.17	63.28	12.94	15.08	11.49	19.23	24.97	36.31	71.57	22.55
		3	284.39	182.04	10.76	6.49	8.25	9.53	19.17	25.33	48.02	20.86	288.12	157.49	18.53	11.78	16.59	19.88	26.68	32.81	71.99	28.53
		4	432.07	186.43	21.69	5.40	9.61	10.88	18.88	24.72	59.32	24.66	419.46	174.95	17.95	12.90	18.08	20.13	25.49	39.90	56.12	31.10
	Year	16/17	167.00	775.00	245.00	96.00	265.00	297.00	253.00	181.00	135.00	2,414.00	167.00	627.00	246.00	203.00	458.00	583.00	333.00	285.00	290.00	3,192.00
	Episodes	F/N	712.88	152.40	18.83	6.37	9.24	10.00	21.84	29.07	54.94	21.32	470.43	125.41	20.36	13.61	15.93	19.88	27.33	37.63	66.82	27.63
**London**	Quarter	1	499.31	141.09	41.80	11.06	9.93	16.19	36.51	41.31	76.62	26.41	227.60	132.38	33.52	10.34	15.80	22.28	36.34	40.79	43.17	27.69
		2	453.91	91.60	28.33	13.10	8.95	14.67	25.17	41.41	57.45	21.39	555.50	84.39	20.28	13.22	14.76	23.17	30.39	41.02	50.43	24.62
		3	292.22	169.51	22.74	9.89	7.66	16.40	23.89	45.62	48.00	25.51	347.44	151.52	26.27	11.10	14.58	22.52	23.50	44.91	51.56	28.95
		4	471.65	205.57	30.72	9.38	11.38	15.14	21.44	36.78	83.16	32.37	371.78	206.80	35.70	16.23	19.12	26.17	25.51	48.62	61.77	37.86
	Year	16/17	179.00	703.00	303.00	93.00	303.00	264.00	100.00	86.00	51.00	2,082.00	160.00	655.00	273.00	120.00	543.00	375.00	122.00	119.00	67.00	2,434.00
	Episodes	F/N	429.74	150.80	30.85	10.90	9.47	15.58	26.72	41.28	66.14	26.32	378.97	142.65	28.78	12.73	16.04	23.53	28.96	43.78	51.71	29.68
**Midlands And East**	Quarter	1	2,012.52	201.19	38.79	9.46	13.25	14.05	22.79	28.35	69.72	27.95	462.81	135.59	30.37	13.71	23.67	27.30	33.64	37.73	70.29	33.48
		2	585.26	83.14	20.64	5.97	8.43	11.17	24.72	49.37	69.87	20.97	283.23	70.75	17.92	7.11	15.80	22.51	29.63	39.59	94.53	26.39
		3	391.75	284.24	20.44	8.63	8.58	11.70	22.83	34.43	66.25	29.59	467.82	215.98	23.56	16.53	17.25	25.70	34.20	36.69	88.97	36.89
		4	487.49	220.09	24.22	11.76	10.22	15.78	22.48	25.11	90.96	30.70	341.50	198.30	35.53	20.22	18.16	22.92	25.62	45.12	80.48	36.51
	Year	16/17	121.00	567.00	199.00	63.00	153.00	238.00	168.00	135.00	103.00	1,747.00	92.00	435.00	196.00	95.00	285.00	441.00	239.00	183.00	209.00	2,175.00
	Episodes	F/N	863.90	195.01	25.92	8.90	10.09	13.14	23.23	34.60	74.12	27.19	386.85	153.56	26.68	14.25	18.66	24.57	30.75	39.78	83.78	33.19

Mean weekly incidence rate per 100,000 Persons.

Hayfever/Allergic Rhinitis (ICD10: J30)

		M										F									
		<1yr	1-4yrs	5-14yrs	15-24yrs	25-44yrs	45-64yrs	65-74yrs	75-84yrs	85+yrs	All Ages	<1yr	1-4yrs	5-14yrs	15-24yrs	25-44yrs	45-64yrs	65-74yrs	75-84yrs	85+yrs	All Ages
**4 weekly**	1	0.00	30.42	80.83	38.78	16.65	14.31	11.81	9.87	10.20	26.12	0.00	25.51	43.19	37.10	30.54	25.82	22.94	18.66	10.93	29.00
	2	0.00	42.54	134.11	86.23	43.49	26.19	24.52	17.71	7.21	50.74	0.00	38.42	87.49	79.83	74.86	32.16	27.72	28.96	18.09	54.32
	3	0.00	27.44	52.12	31.31	20.74	12.22	19.53	12.74	18.77	22.96	0.00	18.75	36.10	35.39	33.96	22.55	19.09	30.79	14.97	28.09
	4	0.00	3.59	22.71	12.99	8.25	5.58	12.37	12.34	13.11	10.14	0.00	7.08	16.41	13.67	11.84	12.13	10.29	12.79	13.32	12.08
	5	0.00	2.03	15.37	5.35	6.45	5.57	7.83	9.72	8.72	7.15	0.00	3.99	11.76	10.87	7.13	5.10	7.53	8.31	5.17	7.46
	6	0.00	3.47	10.41	8.36	6.62	4.26	4.50	0.51	12.04	6.17	0.00	2.15	4.89	6.27	8.84	6.08	4.22	6.97	4.05	6.41
	7	0.00	1.39	7.98	6.54	5.35	4.37	6.76	6.75	7.59	5.66	0.00	2.57	7.51	10.17	7.46	4.37	5.49	4.67	9.88	6.45
	8	0.00	5.00	6.33	4.40	3.36	2.99	3.73	6.66	1.54	3.93	0.00	0.00	6.00	6.10	4.89	5.25	3.60	7.01	2.66	4.81
	9	0.00	0.64	5.66	2.31	3.26	2.61	3.80	6.34	1.36	3.23	0.00	0.00	3.30	4.75	3.61	3.71	2.26	3.24	1.46	3.22
	10	0.00	1.66	5.71	7.44	4.32	3.49	1.97	3.92	3.60	4.37	0.00	0.00	4.20	5.85	5.60	4.48	4.99	0.44	0.00	4.24
	11	0.00	4.74	9.75	8.80	5.75	4.41	4.60	8.39	17.81	6.31	0.00	0.63	8.69	7.50	7.85	6.07	7.57	9.59	6.77	6.68
	12	0.00	8.47	29.16	18.37	11.78	8.72	8.76	10.33	8.90	13.22	3.87	12.28	22.11	14.86	20.89	17.70	13.55	9.95	6.82	17.43
	13	3.45	12.99	49.62	19.45	15.86	15.03	14.60	16.44	5.35	19.47	3.62	13.44	24.36	27.13	23.39	14.55	18.93	19.96	6.42	19.65
**Quarter**	1	0.00	33.23	88.39	51.08	26.17	17.32	18.10	13.16	11.92	32.72	0.00	27.40	54.64	49.72	45.23	26.76	23.23	25.56	14.38	36.51
	2	0.00	2.79	15.45	8.43	6.87	5.02	8.02	7.57	11.45	7.59	0.00	4.31	10.98	9.84	8.91	7.10	7.10	8.26	7.50	8.27
	3	0.00	2.46	5.85	4.58	3.89	3.31	3.81	5.66	1.66	3.99	0.00	0.22	4.44	6.50	5.02	4.62	3.60	4.34	3.16	4.41
	4	1.06	8.06	27.58	15.27	10.56	8.84	8.76	11.23	10.62	12.35	2.31	8.11	17.35	15.88	16.61	12.19	12.89	12.29	6.16	13.89
**Year**	16/17	1.00	185.00	1394.00	824.00	1262.00	741.00	280.00	151.00	50.00	4888.00	2.00	160.00	862.00	863.00	1980.00	1034.00	356.00	231.00	76.00	5564.00
**Episodes**	F/N	0.26	11.47	33.96	19.63	11.78	8.55	9.64	9.37	8.96	14.04	0.57	9.90	21.65	20.28	18.75	12.56	11.62	12.53	7.79	15.63

Mean weekly incidence rate per 100,000 Persons.

Hayfever/Allergic Rhinitis (ICD10: J30)

			M										F									
			<1yr	1-4yrs	5-14yrs	15-24yrs	25-44yrs	45-64yrs	65-74yrs	75-84yrs	85+yrs	All Ages	<1yr	1-4yrs	5-14yrs	15-24yrs	25-44yrs	45-64yrs	65-74yrs	75-84yrs	85+yrs	All Ages
**North**	Quarter	1	0.00	26.37	69.42	37.28	23.33	14.86	11.50	13.25	3.63	25.89	0.00	29.71	49.01	36.90	36.54	18.17	16.53	18.08	5.85	28.09
		2	0.00	1.53	11.78	6.09	7.56	3.60	4.41	8.65	8.49	6.25	0.00	2.36	11.30	6.47	7.53	3.57	3.82	5.23	6.40	5.97
		3	0.00	0.00	3.02	1.84	1.59	2.11	1.57	1.20	1.90	1.82	0.00	0.00	4.43	3.22	3.24	2.30	3.38	2.02	0.00	2.81
		4	0.00	7.71	12.33	7.51	5.52	2.71	6.05	2.41	3.77	5.71	4.46	4.12	10.97	7.54	11.35	6.59	4.52	7.01	3.11	7.95
	Year	16/17	0.00	44.00	313.00	212.00	302.00	184.00	73.00	43.00	10.00	1,181.00	1.00	43.00	236.00	220.00	444.00	238.00	92.00	65.00	16.00	1,355.00
	Episodes	F/N	0.00	8.76	23.90	13.05	9.46	5.78	5.85	6.42	4.52	9.85	1.09	8.92	18.78	13.40	14.53	7.58	7.00	8.03	3.89	11.11
**South**	Quarter	1	0.00	32.31	74.26	46.32	24.09	12.50	15.76	11.68	13.75	28.42	0.00	13.62	46.85	45.47	40.38	19.84	20.19	18.14	16.30	30.76
		2	0.00	1.44	11.48	7.78	6.66	4.42	5.30	5.41	2.96	6.23	0.00	3.27	6.49	8.00	7.48	5.09	5.01	7.05	3.52	6.18
		3	0.00	2.26	6.12	5.72	3.89	2.45	1.99	4.79	4.76	3.80	0.00	0.00	2.73	5.44	3.84	3.12	1.81	1.49	1.79	3.12
		4	0.00	4.76	23.00	12.38	6.42	6.31	6.45	9.94	1.58	9.13	0.00	9.41	10.91	15.58	12.01	7.93	7.02	3.26	6.97	9.84
	Year	16/17	0.00	44.00	347.00	248.00	273.00	180.00	81.00	49.00	13.00	1,235.00	0.00	30.00	187.00	256.00	417.00	247.00	97.00	52.00	29.00	1,315.00
	Episodes	F/N	0.00	10.03	28.39	17.86	10.20	6.38	7.34	7.91	5.71	11.79	0.00	6.51	16.55	18.42	15.77	8.92	8.44	7.48	7.08	12.36
**London**	Quarter	1	0.00	25.97	116.46	60.21	32.55	27.12	30.94	22.05	15.12	43.95	0.00	26.59	74.38	55.52	53.66	48.53	37.06	40.86	26.68	51.97
		2	0.00	8.20	23.20	11.88	8.45	8.26	11.03	9.27	18.64	10.81	0.00	7.60	20.09	16.15	12.74	14.18	8.77	12.35	17.13	13.78
		3	0.00	3.66	9.45	7.62	6.89	5.29	7.52	13.78	0.00	6.85	0.00	0.88	9.11	11.61	6.85	9.72	6.78	10.53	9.37	8.00
		4	4.25	14.28	55.77	26.65	18.98	19.15	14.95	23.24	22.09	23.91	4.77	16.07	33.73	26.04	25.60	23.71	29.20	28.35	12.93	25.56
	Year	16/17	1.00	60.00	496.00	221.00	529.00	251.00	60.00	35.00	11.00	1,664.00	1.00	58.00	320.00	249.00	824.00	377.00	86.00	62.00	22.00	1,999.00
	Episodes	F/N	1.04	12.94	50.69	26.31	16.56	14.83	16.02	16.94	14.05	21.18	1.17	12.69	34.06	27.12	24.49	23.85	20.23	22.82	16.54	24.62
**Midlands And East**	Quarter	1	0.00	48.27	93.42	60.53	24.69	14.80	14.18	5.68	15.18	32.64	0.00	39.69	48.33	61.00	50.34	20.52	19.13	25.17	8.67	35.22
		2	0.00	0.00	15.34	7.99	4.80	3.79	11.36	6.93	15.72	7.05	0.00	4.00	6.04	8.74	7.90	5.55	10.81	8.38	2.96	7.14
		3	0.00	3.92	4.78	3.14	3.19	3.39	4.14	2.87	0.00	3.47	0.00	0.00	1.49	5.74	6.14	3.33	2.42	3.31	1.49	3.70
		4	0.00	5.49	19.21	14.52	11.33	7.19	7.59	9.33	15.05	10.66	0.00	2.82	13.78	14.35	17.47	10.51	10.80	10.52	1.62	12.23
	Year	16/17	0.00	37.00	238.00	143.00	158.00	126.00	66.00	24.00	16.00	808.00	0.00	29.00	119.00	138.00	295.00	172.00	81.00	52.00	9.00	895.00
	Episodes	F/N	0.00	14.15	32.85	21.29	10.89	7.22	9.36	6.22	11.57	13.34	0.00	11.48	17.20	22.20	20.23	9.90	10.79	11.78	3.67	14.43

Mean weekly incidence rate per 100,000 Persons.

Symptoms involving Respiratory & Chest (ICD10: R05-R07,R09)

		M										F									
		<1yr	1-4yrs	5-14yrs	15-24yrs	25-44yrs	45-64yrs	65-74yrs	75-84yrs	85+yrs	All Ages	<1yr	1-4yrs	5-14yrs	15-24yrs	25-44yrs	45-64yrs	65-74yrs	75-84yrs	85+yrs	All Ages
**4 weekly**	1	0.00	22.72	8.21	9.25	14.30	15.26	16.65	25.06	36.83	14.80	0.00	17.94	9.71	13.77	13.87	20.98	27.46	30.50	27.70	17.65
	2	0.00	23.13	13.08	8.45	14.21	18.34	31.45	30.94	30.47	17.33	0.00	19.77	10.84	9.71	20.05	19.92	26.20	27.33	25.06	18.40
	3	90.58	29.33	9.34	8.87	16.40	20.68	26.92	36.48	44.54	18.85	48.45	21.44	10.09	13.79	14.34	21.97	25.43	32.18	27.71	18.09
	4	29.48	18.29	8.90	12.92	13.65	22.00	30.11	28.69	36.44	17.97	44.88	20.20	6.47	15.72	16.09	23.59	24.49	32.20	30.08	18.81
	5	25.33	21.17	10.66	14.25	18.20	19.70	31.98	42.75	38.93	20.06	12.76	19.25	13.34	17.80	18.71	23.67	23.28	30.90	43.63	21.04
	6	43.50	31.91	14.92	18.51	18.13	23.71	28.01	33.81	61.57	22.06	31.21	17.98	9.03	22.44	22.47	27.52	31.55	41.16	33.99	24.14
	7	47.33	46.81	12.35	16.23	18.58	22.72	33.11	42.49	39.93	22.23	78.56	40.87	14.21	16.76	20.34	25.42	32.30	48.56	38.91	24.41
	8	53.21	32.85	16.70	15.22	15.58	24.58	30.30	43.16	37.99	21.73	74.29	37.25	13.10	19.91	23.31	28.75	29.14	44.37	39.86	25.96
	9	62.49	28.02	7.92	15.49	17.86	26.95	35.56	47.48	45.45	22.66	55.55	27.02	9.85	12.94	22.52	34.58	32.73	39.34	51.58	25.55
	10	42.99	15.99	15.00	14.68	18.18	28.78	33.38	55.71	44.72	23.46	23.31	30.20	18.25	19.43	23.36	33.58	37.23	45.44	43.61	27.86
	11	54.75	25.91	13.34	12.40	18.85	21.14	25.80	38.27	43.34	20.49	48.82	35.02	7.57	10.80	19.92	28.13	32.56	54.15	33.90	23.63
	12	60.97	27.86	16.86	14.26	16.99	21.75	33.60	43.48	38.13	21.95	51.06	17.97	13.55	19.60	20.41	27.08	32.28	33.87	51.08	23.49
	13	51.36	16.19	9.33	12.23	14.15	19.06	35.83	35.86	32.37	18.31	46.40	20.85	6.12	14.76	17.34	20.44	25.55	36.68	22.28	18.28
**Quarter**	1	27.87	24.88	10.05	8.88	14.92	17.88	24.37	30.38	37.24	16.82	14.91	19.58	10.17	12.53	15.92	20.96	26.45	30.04	26.89	18.02
	2	34.20	25.84	11.57	15.58	16.68	21.59	30.78	36.50	45.07	20.17	39.28	21.95	10.32	18.17	19.75	24.80	26.87	35.99	36.56	21.75
	3	51.55	31.58	13.23	14.87	18.05	26.29	33.22	46.61	43.29	22.90	56.33	33.88	13.49	17.56	21.97	31.50	33.23	44.08	46.38	26.20
	4	56.66	22.13	13.07	13.33	16.35	21.35	31.37	40.76	37.06	20.28	45.01	24.51	9.69	15.24	19.68	25.71	30.48	42.28	33.92	22.12
**Year**	16/17	74.00	444.00	504.00	627.00	1729.00	2069.00	974.00	690.00	270.00	7381.00	74.00	401.00	438.00	755.00	2032.00	2347.00	1003.00	785.00	427.00	8262.00
**Episodes**	F/N	42.42	26.10	11.97	13.21	16.50	21.77	29.95	38.52	40.75	20.04	38.89	24.92	10.91	15.91	19.34	25.73	29.21	38.06	35.95	22.02

Mean weekly incidence rate per 100,000 Persons.

Symptoms involving Respiratory and Chest (ICD10: R05 - R07; R09)

			M										F									
			<1yr	1-4yrs	5-14yrs	15-24yrs	25-44yrs	45-64yrs	65-74yrs	75-84yrs	85+yrs	All Ages	<1yr	1-4yrs	5-14yrs	15-24yrs	25-44yrs	45-64yrs	65-74yrs	75-84yrs	85+yrs	All Ages
**North**	Quarter	1	0.00	22.49	11.73	8.94	15.37	15.82	25.22	34.62	34.56	17.06	0.00	16.22	8.83	11.85	16.32	19.80	21.79	23.86	40.70	17.82
		2	33.70	25.90	7.94	14.52	15.65	22.99	23.44	35.92	40.61	19.49	72.25	12.70	8.19	18.48	17.05	24.80	21.24	27.22	34.77	19.98
		3	52.84	25.35	9.37	15.97	15.68	25.81	28.63	28.96	36.47	20.70	87.01	27.19	12.85	16.31	20.44	28.62	26.38	33.36	44.17	23.89
		4	26.67	20.96	10.52	13.37	15.50	21.54	31.73	29.52	60.15	19.76	41.34	15.60	5.99	15.34	19.44	26.36	27.57	40.85	25.74	21.68
	Year	16/17	18.00	123.00	130.00	229.00	507.00	696.00	340.00	216.00	96.00	2,355.00	26.00	87.00	114.00	284.00	568.00	784.00	320.00	253.00	149.00	2,585.00
	Episodes	F/N	28.41	23.71	9.85	13.23	15.55	21.57	27.18	32.32	42.90	19.26	50.57	17.83	8.95	15.55	18.29	24.89	24.19	31.25	36.32	20.82
**South**	Quarter	1	0.00	20.89	7.74	9.42	16.89	17.46	21.58	26.82	36.45	16.63	0.00	14.66	7.45	12.84	16.61	18.07	19.61	21.90	30.29	16.63
		2	47.67	15.12	7.51	13.52	14.00	17.99	21.85	27.21	27.36	16.18	0.00	11.12	5.74	12.34	15.42	15.43	21.11	22.92	31.86	15.50
		3	28.13	19.63	8.88	12.05	16.89	19.93	24.44	30.95	33.23	18.17	41.15	19.59	8.26	18.15	22.26	22.59	29.47	30.83	33.02	22.01
		4	43.17	15.23	7.28	11.36	14.08	16.61	18.78	30.91	22.19	15.41	52.53	11.16	10.93	10.11	16.01	17.55	20.27	25.42	28.12	16.65
	Year	16/17	15.00	88.00	105.00	177.00	444.00	534.00	251.00	183.00	72.00	1,869.00	18.00	68.00	102.00	200.00	507.00	541.00	280.00	194.00	134.00	2,044.00
	Episodes	F/N	30.08	17.67	7.85	11.62	15.44	18.00	21.66	28.94	29.76	16.59	22.98	14.08	8.05	13.34	17.53	18.35	22.59	25.22	30.84	17.66
**London**	Quarter	1	111.48	28.67	11.91	9.60	14.79	19.69	32.19	31.78	35.41	17.33	59.63	21.03	10.19	11.72	12.41	25.16	40.03	48.32	17.68	17.89
		2	14.97	25.93	15.12	12.60	12.81	22.38	41.72	35.45	57.08	18.37	69.38	27.59	14.45	17.42	18.28	29.64	34.46	51.22	39.03	22.97
		3	45.42	34.34	17.80	12.24	15.21	29.29	44.61	55.27	53.87	22.34	34.51	41.77	15.51	14.63	19.89	36.40	42.59	63.16	53.76	26.40
		4	51.24	23.80	16.28	15.22	13.78	23.73	35.38	41.00	11.15	19.01	43.72	31.98	7.93	13.32	16.91	26.60	45.34	60.41	51.57	21.76
	Year	16/17	19.00	131.00	151.00	107.00	452.00	403.00	145.00	85.00	31.00	1,524.00	19.00	140.00	114.00	134.00	572.00	469.00	172.00	151.00	53.00	1,824.00
	Episodes	F/N	55.01	28.14	15.27	12.42	14.12	23.75	38.54	40.77	39.71	19.24	52.14	30.53	12.06	14.33	16.90	29.45	40.49	55.69	40.48	22.27
**Midlands And East**	Quarter	1	0.00	27.47	8.84	7.57	12.63	18.52	18.47	28.31	42.56	16.28	0.00	26.42	14.23	13.70	18.33	20.80	24.35	26.07	18.90	19.72
		2	40.47	36.42	15.73	21.68	24.25	23.00	36.14	47.42	55.24	26.64	15.49	36.38	12.90	24.43	28.26	29.33	30.68	42.61	40.57	28.54
		3	79.82	46.99	16.86	19.21	24.40	30.15	35.19	71.26	49.58	30.38	62.65	46.97	17.37	21.13	25.28	38.40	34.48	48.97	54.56	32.51
		4	105.57	28.54	18.20	13.37	22.04	23.51	39.61	61.61	54.74	26.94	42.45	39.31	13.92	22.17	26.35	32.34	28.75	42.45	30.24	28.39
	Year	16/17	22.00	102.00	118.00	114.00	326.00	436.00	238.00	206.00	71.00	1,633.00	11.00	106.00	108.00	137.00	385.00	553.00	231.00	187.00	91.00	1,809.00
	Episodes	F/N	56.16	34.89	14.92	15.58	20.89	23.78	32.42	52.06	50.62	25.09	29.87	37.26	14.57	20.43	24.63	30.20	29.58	40.08	36.15	27.31

Mean weekly incidence rate per 100,000 Persons.

Acute Bronchitis (ICD10: J20-J21,J40)

3.

		M										F									
		<1yr	1-4yrs	5-14yrs	15-24yrs	25-44yrs	45-64yrs	65-74yrs	75-84yrs	85+yrs	All Ages	<1yr	1-4yrs	5-14yrs	15-24yrs	25-44yrs	45-64yrs	65-74yrs	75-84yrs	85+yrs	All Ages
**4 weekly**	1	0.00	111.53	22.35	12.21	20.61	33.68	85.01	122.32	183.47	41.35	0.00	87.92	18.33	17.63	29.60	47.10	86.33	116.33	201.33	49.74
	2	0.00	91.13	23.88	13.96	19.27	34.82	69.45	117.22	234.19	40.41	0.00	83.16	19.47	20.06	29.14	53.14	86.51	113.27	198.14	51.23
	3	65.79	111.16	27.14	19.68	23.28	34.23	82.01	106.54	194.77	43.90	28.67	86.99	26.18	19.95	39.20	62.07	91.89	125.83	186.52	58.11
	4	0.00	67.67	13.70	9.29	19.33	32.66	64.26	112.94	164.01	34.66	0.00	51.60	13.37	15.29	27.43	37.86	57.75	103.01	168.38	39.72
	5	67.91	90.21	17.29	16.15	20.97	35.22	61.10	102.80	179.75	37.47	26.79	58.83	12.03	21.89	31.86	55.81	66.45	97.62	177.34	47.28
	6	247.51	218.40	32.93	23.72	31.51	46.55	107.32	148.01	224.05	60.22	155.99	149.30	23.82	41.99	53.91	78.17	104.64	166.08	299.31	77.87
	7	531.80	344.90	37.80	20.61	32.57	61.82	123.30	182.11	306.16	76.29	460.05	283.24	31.39	32.40	57.39	89.60	137.44	206.72	356.47	95.32
	8	884.84	404.48	58.73	24.68	33.46	68.95	139.38	228.82	366.06	90.83	717.44	353.11	47.64	35.06	63.10	128.61	171.32	240.44	403.82	120.82
	9	564.65	202.53	40.11	33.73	43.74	110.84	219.96	337.48	592.34	110.74	369.37	168.08	34.83	38.30	73.37	166.27	259.25	378.15	642.98	145.48
	10	342.17	199.40	34.75	26.90	39.50	91.64	147.39	288.30	500.20	89.90	249.75	139.78	36.30	33.31	61.22	117.26	169.34	264.56	469.15	107.64
	11	292.09	147.83	24.27	17.48	30.93	60.93	118.18	183.48	275.59	63.54	181.35	97.71	22.94	22.61	48.45	90.53	141.63	187.87	294.07	79.65
	12	287.10	111.26	29.44	18.22	27.06	57.84	109.09	175.58	289.76	59.28	203.03	113.72	25.54	22.05	45.24	69.47	111.69	177.15	261.10	70.59
	13	188.60	90.47	15.98	14.00	23.14	47.48	90.05	160.86	260.06	48.81	113.58	48.17	14.96	25.18	37.57	69.61	111.99	148.42	246.65	61.65
**Quarter**	1	20.24	105.14	24.29	15.05	21.02	34.20	79.30	115.89	202.56	41.85	8.82	86.17	21.10	19.09	32.41	53.57	88.09	118.31	195.79	52.77
	2	158.42	147.12	22.95	17.19	25.01	41.02	81.31	129.84	200.71	47.60	99.72	106.15	18.30	27.60	40.16	61.28	85.51	132.50	231.30	59.76
	3	616.20	297.07	45.36	27.76	38.86	87.35	169.87	275.49	471.31	96.96	485.18	245.77	39.47	35.23	66.58	134.04	194.74	285.44	499.71	123.96
	4	261.40	122.08	23.78	16.74	27.19	57.38	105.65	175.57	284.22	58.46	169.69	90.23	21.69	23.69	43.88	77.87	122.87	178.04	272.51	72.00
**Year**	16/17	568.00	2938.00	1236.00	875.00	2925.00	5347.00	3825.00	3243.00	1997.00	22954.00	383.00	2230.00	1026.00	1254.00	4846.00	7753.00	4541.00	4045.00	3699.00	29777.00
**Episodes**	F/N	262.07	167.46	28.98	19.15	27.96	54.72	108.51	173.36	288.02	60.96	189.13	131.59	25.01	26.42	45.65	81.30	122.10	177.70	298.54	76.80

Mean weekly incidence rate per 100,000 Persons.

Acute Bronchitis (ICD10: J20-J21,J40)

			M										F									
			<1yr	1-4yrs	5-14yrs	15-24yrs	25-44yrs	45-64yrs	65-74yrs	75-84yrs	85+yrs	All Ages	<1yr	1-4yrs	5-14yrs	15-24yrs	25-44yrs	45-64yrs	65-74yrs	75-84yrs	85+yrs	All Ages
**North**	Quarter	1	0.00	115.03	21.30	14.02	21.96	41.20	81.10	119.81	231.77	45.70	35.29	78.61	18.74	20.89	40.95	63.01	89.90	116.24	189.86	58.68
		2	269.54	196.05	25.95	16.10	27.94	49.21	91.30	116.79	225.97	54.60	180.67	145.45	19.17	23.78	45.98	73.30	96.37	150.85	250.31	70.19
		3	644.56	302.76	35.34	24.99	40.05	93.93	175.42	276.82	464.95	99.71	571.51	246.45	38.55	32.18	72.41	141.05	200.41	287.96	501.69	130.41
		4	270.63	111.43	19.32	14.33	28.01	61.52	102.41	176.34	305.81	59.59	146.57	97.24	20.21	19.15	49.27	83.54	131.15	193.72	297.66	78.87
	Year	16/17	182.00	941.00	335.00	300.00	961.00	1,980.00	1,405.00	1,146.00	684.00	7,934.00	126.00	691.00	306.00	434.00	1,619.00	2,838.00	1,706.00	1,509.00	1,254.00	10,483.00
	Episodes	F/N	295.68	181.59	25.49	17.34	29.46	61.23	112.16	171.39	305.59	64.70	232.51	142.00	24.07	24.00	52.04	89.91	128.83	186.51	308.76	84.27
**South**	Quarter	1	0.00	108.81	18.21	10.70	19.31	26.89	64.31	99.69	197.21	37.07	0.00	95.86	20.64	16.45	29.08	46.37	63.28	95.23	201.57	48.55
		2	136.68	131.00	17.01	15.77	18.14	33.00	61.52	108.41	187.54	40.35	96.43	88.08	12.04	27.66	35.25	47.70	65.62	99.13	199.09	51.13
		3	640.58	263.88	31.91	18.46	33.89	68.50	143.88	238.08	449.30	85.43	466.05	237.73	27.36	31.12	61.39	107.30	166.35	234.92	436.32	111.22
		4	272.35	90.58	19.83	11.69	24.09	45.43	87.72	141.51	220.41	49.45	151.79	70.71	16.70	18.83	39.67	59.26	91.36	145.88	261.79	62.16
	Year	16/17	162.00	754.00	294.00	219.00	696.00	1,321.00	1,056.00	940.00	650.00	6,092.00	103.00	603.00	238.00	361.00	1,210.00	1,955.00	1,215.00	1,120.00	1,212.00	8,017.00
	Episodes	F/N	260.03	148.24	21.65	14.19	23.75	43.26	88.83	146.20	262.18	52.84	177.02	122.43	19.05	23.59	41.23	64.83	96.07	142.95	273.27	67.94
**London**	Quarter	1	0.00	90.75	24.74	17.84	15.36	32.40	72.15	105.56	160.64	31.69	0.00	69.34	21.56	15.03	23.49	48.47	104.11	128.17	154.69	39.87
		2	91.32	94.79	20.38	20.43	20.78	38.76	79.79	132.85	157.80	36.55	90.80	73.54	19.37	25.54	26.78	62.77	83.21	141.36	206.78	45.60
		3	499.59	259.24	55.90	28.77	29.79	85.09	156.73	267.02	478.85	78.01	426.44	209.77	45.12	31.17	52.06	132.45	189.20	304.31	459.89	97.09
		4	209.74	103.81	27.16	16.51	20.12	52.41	87.07	193.34	182.77	43.24	135.86	79.56	21.61	27.45	32.59	78.34	133.95	181.84	197.48	55.53
	Year	16/17	123.00	635.00	314.00	179.00	688.00	884.00	370.00	360.00	185.00	3,738.00	87.00	490.00	254.00	235.00	1,137.00	1,282.00	539.00	509.00	331.00	4,864.00
	Episodes	F/N	198.11	136.35	31.83	20.88	21.50	51.91	98.58	173.90	243.37	47.17	161.91	107.40	26.77	24.81	33.60	80.17	126.78	188.02	253.81	59.26
**Midlands And East**	Quarter	1	80.97	105.96	32.92	17.63	27.45	36.31	99.64	138.51	220.60	52.92	0.00	100.86	23.46	23.98	36.13	56.41	95.08	133.62	237.05	63.99
		2	136.15	166.63	28.45	16.47	33.19	43.11	92.63	161.30	231.54	58.91	30.99	117.52	22.62	33.41	52.64	61.36	96.85	138.65	269.02	72.10
		3	680.06	362.40	58.28	38.80	51.72	101.87	203.44	320.05	492.14	124.70	476.72	289.12	46.85	46.45	80.46	155.34	222.98	314.57	600.94	157.13
		4	292.89	182.50	28.79	24.40	36.55	70.17	145.40	191.07	427.89	81.58	244.53	113.44	28.24	29.33	53.98	90.33	135.01	190.71	333.13	91.44
	Year	16/17	101.00	608.00	293.00	177.00	580.00	1,162.00	994.00	797.00	478.00	5,190.00	67.00	446.00	228.00	224.00	880.00	1,678.00	1,081.00	907.00	902.00	6,413.00
	Episodes	F/N	294.47	203.66	36.95	24.18	37.15	62.49	134.47	201.95	340.94	79.14	185.10	154.52	30.15	33.29	55.74	90.30	136.71	193.33	358.32	95.71

Mean weekly incidence rate per 100,000 Persons.

Common Cold (ICD10: J00,J06)

		M										F									
		<1yr	1-4yrs	5-14yrs	15-24yrs	25-44yrs	45-64yrs	65-74yrs	75-84yrs	85+yrs	All Ages	<1yr	1-4yrs	5-14yrs	15-24yrs	25-44yrs	45-64yrs	65-74yrs	75-84yrs	85+yrs	All Ages
**4 weekly**	1	0.00	531.39	86.20	28.13	16.38	21.19	30.95	20.80	30.64	53.16	0.00	476.60	89.81	44.67	49.63	32.76	36.86	31.04	32.23	65.14
	2	0.00	477.18	85.59	27.39	21.60	21.35	21.38	34.67	28.81	52.86	178.57	430.59	81.43	39.66	45.52	37.75	35.76	40.51	40.76	63.17
	3	437.93	455.98	90.83	24.11	22.04	20.41	26.06	15.00	27.49	51.30	233.11	442.04	109.25	46.82	49.78	37.55	29.11	34.36	39.95	67.57
	4	291.81	299.89	40.85	19.30	17.63	18.44	18.17	18.02	43.11	35.60	290.96	273.84	40.01	36.51	31.05	27.20	25.85	31.45	44.84	42.85
	5	477.20	398.06	58.07	31.07	20.47	18.85	19.94	26.40	16.00	45.27	433.93	348.63	65.48	45.35	35.35	30.62	20.48	29.82	24.17	51.83
	6	1048.51	897.35	137.83	60.92	33.97	36.12	49.81	46.81	34.51	97.03	824.99	807.60	126.40	102.15	74.65	61.41	61.93	59.79	78.04	114.09
	7	1441.47	1051.38	131.30	48.67	41.15	40.87	52.01	47.81	61.89	109.09	1286.44	1013.29	143.13	94.28	85.54	80.06	72.56	86.50	87.40	136.18
	8	1466.16	1087.43	204.14	56.36	44.85	51.09	60.68	80.13	88.11	128.10	1556.04	1121.32	233.81	95.77	106.29	96.48	89.12	99.68	110.68	165.38
	9	1199.59	710.83	150.59	62.96	54.92	79.39	93.03	127.44	154.83	119.80	1204.80	707.07	146.42	107.59	124.43	134.43	134.76	158.24	158.53	161.29
	10	1257.66	811.44	217.37	72.10	47.21	56.95	57.32	71.43	77.97	120.59	1098.89	792.92	206.27	115.21	113.84	104.46	92.05	106.58	111.86	154.58
	11	1030.34	645.30	110.77	43.55	36.01	38.31	49.05	48.89	54.00	85.33	959.58	591.30	129.47	70.12	79.45	74.72	70.46	71.75	69.74	109.00
	12	1290.64	616.37	130.39	29.93	28.22	30.22	36.59	37.67	24.23	80.83	1209.99	590.84	133.55	58.88	66.67	65.59	53.06	56.21	46.60	103.15
	13	1006.48	347.11	61.16	28.17	22.38	25.38	28.61	29.33	48.86	54.50	683.60	316.69	74.16	44.34	51.93	43.04	53.18	40.39	43.93	67.67
**Quarter**	1	134.75	491.51	87.44	26.67	19.73	21.00	26.50	23.28	29.11	52.49	126.67	451.81	93.21	43.79	48.41	35.77	34.14	34.98	37.23	65.28
	2	699.05	581.76	81.77	38.75	27.23	26.94	33.52	32.72	36.45	64.86	625.75	535.65	84.47	65.68	53.15	45.83	42.53	44.87	56.16	78.32
	3	1353.19	929.59	187.41	62.39	47.22	60.06	68.58	88.46	101.53	123.40	1287.96	919.75	192.00	105.20	110.14	107.83	100.19	120.92	121.66	158.85
	4	1127.79	556.25	106.82	35.27	30.17	32.66	38.47	40.59	44.91	76.33	976.38	522.93	116.64	61.06	69.30	63.73	61.22	58.58	56.56	97.12
**Year**	16/17	1812.00	11456.00	5080.00	1908.00	3401.00	3310.00	1431.00	846.00	350.00	29594.00	1526.00	10512.00	5073.00	3308.00	7699.00	5891.00	2093.00	1411.00	786.00	38299.00
**Episodes**	F/N	826.25	638.68	115.22	40.73	31.01	35.01	41.61	46.01	52.69	79.00	751.77	606.18	120.88	68.87	69.93	62.96	59.20	64.46	67.68	99.48

Mean weekly incidence rate per 100,000 Persons.

Common Cold (ICD10: J00,J06)

			M										F									
			<1yr	1-4yrs	5-14yrs	15-24yrs	25-44yrs	45-64yrs	65-74yrs	75-84yrs	85+yrs	All Ages	<1yr	1-4yrs	5-14yrs	15-24yrs	25-44yrs	45-64yrs	65-74yrs	75-84yrs	85+yrs	All Ages
**North**	Quarter	1	290.96	550.03	95.24	26.51	18.92	20.50	27.57	30.52	27.55	52.90	63.05	479.58	98.55	37.99	49.47	36.52	33.86	34.71	37.04	63.39
		2	764.29	638.88	93.49	37.01	29.90	27.96	38.13	35.61	42.99	66.64	712.94	610.22	93.68	66.82	55.15	49.14	45.76	46.97	56.28	81.49
		3	1,181.60	869.06	170.79	53.08	44.33	54.21	71.10	91.18	99.96	108.68	1,266.28	918.26	170.35	81.42	110.53	105.25	96.56	106.10	101.42	145.58
		4	1,229.37	602.11	119.59	32.13	29.38	30.05	41.72	37.34	35.83	74.37	1,053.86	528.74	118.26	52.60	68.53	60.91	54.47	48.10	48.86	90.58
	Year	16/17	526.00	3,441.00	1,577.00	642.00	998.00	1,068.00	557.00	323.00	115.00	9,247.00	455.00	3,086.00	1,523.00	1,093.00	2,201.00	1,980.00	760.00	476.00	247.00	11,821.00
	Episodes	F/N	864.63	664.52	119.28	37.18	30.62	33.08	44.51	48.42	51.42	75.48	772.88	633.75	119.71	59.84	70.62	62.69	57.44	58.74	60.81	95.00
**South**	Quarter	1	136.55	410.70	63.85	22.68	20.96	16.36	19.33	13.13	42.89	41.81	315.93	356.62	72.83	38.68	40.54	33.28	29.47	30.24	42.55	53.17
		2	638.14	444.71	56.74	29.72	21.56	18.99	26.78	28.35	33.94	47.95	491.59	425.51	62.48	59.67	44.03	36.53	29.01	42.70	39.15	61.32
		3	1,156.04	798.98	134.33	51.26	39.65	47.16	57.76	66.54	85.02	98.42	1,081.60	743.01	143.10	94.16	94.58	86.78	77.32	107.45	116.98	129.78
		4	905.17	437.14	82.27	32.51	26.56	26.81	36.06	33.44	37.65	60.55	819.20	432.79	85.50	58.98	63.91	52.94	48.75	50.08	42.29	80.56
	Year	16/17	431.00	2,662.00	1,143.00	525.00	793.00	829.00	419.00	229.00	122.00	7,153.00	370.00	2,427.00	1,150.00	972.00	1,789.00	1,577.00	581.00	453.00	265.00	9,584.00
	Episodes	F/N	707.64	521.41	83.78	33.96	27.08	27.17	34.83	35.23	49.58	61.92	673.58	488.28	90.44	62.81	60.45	52.09	45.81	57.33	59.84	80.83
**London**	Quarter	1	111.48	593.73	117.84	30.92	22.76	28.10	38.77	32.47	15.87	71.90	44.98	607.02	135.21	50.46	51.28	46.97	44.81	52.70	45.38	91.32
		2	713.94	706.11	104.50	53.95	31.16	35.72	40.54	35.37	43.05	87.01	700.47	656.73	116.34	80.86	59.95	58.19	60.67	57.64	68.08	104.41
		3	1,632.67	1,143.62	288.88	83.02	51.60	80.79	88.12	108.97	141.13	170.33	1,349.95	1,068.64	278.94	113.43	115.61	150.30	141.70	177.91	150.37	205.94
		4	1,285.03	649.61	143.36	46.43	32.73	41.12	41.99	46.89	61.37	100.18	1,062.02	633.67	154.68	68.30	75.39	85.04	89.35	87.43	81.20	127.40
	Year	16/17	567.00	3,593.00	1,601.00	462.00	1,105.00	787.00	195.00	115.00	49.00	8,474.00	452.00	3,385.00	1,616.00	737.00	2,549.00	1,355.00	356.00	252.00	111.00	10,813.00
	Episodes	F/N	931.60	772.00	162.53	53.59	34.50	46.23	52.13	55.54	64.94	106.97	787.68	739.91	170.26	78.31	75.26	84.62	83.69	93.24	85.91	131.74
**Midlands And East**	Quarter	1	0.00	411.58	72.81	26.56	16.26	19.03	20.34	17.01	30.12	43.37	82.71	364.02	66.25	48.03	52.36	26.32	28.41	22.26	23.95	53.24
		2	679.81	537.32	72.35	34.33	26.28	25.07	28.62	31.56	25.83	57.83	597.99	450.16	65.37	55.36	53.46	39.48	34.68	32.17	61.15	66.07
		3	1,442.46	906.71	155.64	62.21	53.28	58.11	57.34	87.15	80.00	116.18	1,454.03	949.09	175.59	131.79	119.83	88.98	85.19	92.23	117.87	154.08
		4	1,091.58	536.16	82.08	29.99	32.01	32.64	34.09	44.67	44.79	70.21	970.42	496.52	108.13	64.36	69.35	56.04	52.31	48.69	53.88	89.93
	Year	16/17	288.00	1,760.00	759.00	279.00	505.00	626.00	260.00	179.00	64.00	4,720.00	249.00	1,614.00	784.00	506.00	1,160.00	979.00	396.00	230.00	163.00	6,081.00
	Episodes	F/N	801.13	596.80	95.28	38.20	31.85	33.55	34.98	44.84	44.82	71.63	772.92	562.78	103.11	74.52	73.37	52.45	49.86	48.53	64.15	90.36

Mean weekly incidence rate per 100,000 Persons.

Influenza-like illness (ICD10 : J09 - J11)

		M										F									
		<1yr	1-4yrs	5-14yrs	15-24yrs	25-44yrs	45-64yrs	65-74yrs	75-84yrs	85+yrs	All Ages	<1yr	1-4yrs	5-14yrs	15-24yrs	25-44yrs	45-64yrs	65-74yrs	75-84yrs	85+yrs	All Ages
**4 weekly**	1	0.00	0.00	2.24	4.30	3.66	3.23	2.43	0.42	2.46	2.98	0.00	0.60	0.89	3.53	5.24	4.48	2.41	3.14	0.00	3.72
	2	0.00	0.76	1.60	2.83	2.77	2.21	1.86	0.00	3.91	2.17	0.00	1.47	1.43	2.22	3.34	2.26	2.50	2.95	0.00	2.41
	3	0.00	1.57	2.62	2.19	3.70	3.01	1.35	1.99	2.86	2.85	0.00	0.66	0.67	2.31	2.41	3.40	1.36	2.17	0.00	2.29
	4	0.00	1.52	0.26	1.65	1.63	1.61	2.67	0.55	3.04	1.44	0.00	0.00	0.58	1.91	3.06	1.31	0.82	0.42	0.82	1.64
	5	0.00	1.81	0.25	1.96	1.57	3.27	0.28	1.32	0.00	1.76	0.00	0.68	0.00	1.62	3.85	3.16	1.29	1.92	0.83	2.38
	6	0.00	4.44	3.53	5.50	5.09	4.46	2.72	6.50	2.96	4.62	0.00	5.40	3.53	10.41	12.14	8.98	5.53	8.09	5.39	8.82
	7	0.00	3.38	2.69	3.92	6.92	7.16	4.10	2.57	1.38	5.36	0.00	8.13	3.26	9.16	10.58	12.01	8.76	7.22	5.65	9.25
	8	0.00	6.66	4.88	7.78	8.36	9.41	7.09	4.14	1.54	7.68	0.00	17.78	6.39	11.06	12.40	14.77	9.84	10.34	2.57	11.40
	9	5.02	3.97	7.47	14.72	14.43	15.89	12.14	18.08	27.46	13.58	16.34	10.61	5.81	16.47	21.54	27.44	20.68	27.72	29.64	20.58
	10	5.00	8.42	14.21	18.61	14.76	14.79	10.25	9.18	9.56	14.13	0.00	11.10	12.56	19.33	19.59	25.09	12.44	15.34	21.25	18.80
	11	0.00	6.56	7.68	9.44	9.23	7.14	4.54	4.27	7.04	7.64	4.77	7.89	4.63	10.97	11.94	12.66	6.09	10.24	4.77	10.35
	12	0.00	2.59	2.47	3.39	4.33	4.89	1.71	2.26	4.49	3.83	3.82	2.69	1.49	6.46	6.10	7.59	3.57	2.61	3.45	5.28
	13	0.00	1.08	0.86	2.54	3.32	2.50	1.95	2.55	1.55	2.45	0.00	0.00	0.99	2.65	3.59	5.05	2.34	5.22	0.71	3.13
**Quarter**	1	0.00	0.72	2.16	3.20	3.40	2.85	1.92	0.77	3.03	2.69	0.00	0.88	0.99	2.75	3.78	3.46	2.12	2.78	0.00	2.88
	2	0.00	2.42	1.43	3.27	3.52	3.63	1.86	2.54	1.71	3.00	0.00	2.94	1.62	5.49	6.89	6.03	3.55	4.08	2.24	5.09
	3	3.09	6.03	8.04	11.62	11.57	12.62	8.81	9.98	11.53	10.78	5.03	12.50	6.87	14.57	17.21	20.64	14.15	16.74	17.30	15.92
	4	0.00	3.81	4.04	6.25	6.07	5.36	3.79	3.10	4.78	5.22	2.64	4.11	3.45	7.21	7.62	9.19	3.98	6.29	3.38	6.79
**Year**	16/17	2.00	60.00	166.00	282.00	672.00	590.00	139.00	75.00	42.00	2028.00	5.00	85.00	137.00	367.00	1000.00	925.00	213.00	166.00	77.00	2975.00
**Episodes**	F/N	0.76	3.23	3.87	6.03	6.09	6.07	4.05	4.07	5.20	5.38	1.88	5.07	3.20	7.47	8.84	9.76	5.90	7.41	5.67	7.62

Mean weekly incidence rate per 100,000 Persons.

Influenza-like illness (ICD10: J09 - J11)

			M										F									
			<1yr	1-4yrs	5-14yrs	15-24yrs	25-44yrs	45-64yrs	65-74yrs	75-84yrs	85+yrs	All Ages	<1yr	1-4yrs	5-14yrs	15-24yrs	25-44yrs	45-64yrs	65-74yrs	75-84yrs	85+yrs	All Ages
**North**	Quarter	1	0.00	0.00	1.56	2.25	2.59	1.92	1.98	3.10	7.01	2.19	0.00	1.73	0.32	0.71	3.97	2.85	3.15	1.50	0.00	2.38
		2	0.00	2.20	1.48	2.23	3.91	4.34	1.23	1.73	3.43	3.04	0.00	3.17	1.83	3.94	6.54	7.06	3.29	2.41	4.67	4.99
		3	6.18	3.02	3.88	8.68	7.85	8.84	7.06	5.97	10.98	7.47	0.00	3.33	6.28	9.87	12.90	13.97	10.33	13.40	22.89	11.69
		4	0.00	1.51	3.62	4.22	5.94	4.22	5.18	4.29	7.44	4.63	0.00	1.69	2.80	3.74	7.39	6.66	4.82	5.48	5.06	5.47
	Year	16/17	1.00	9.00	35.00	75.00	164.00	156.00	48.00	25.00	16.00	529.00	0.00	12.00	36.00	85.00	239.00	241.00	71.00	46.00	33.00	763.00
	Episodes	F/N	1.52	1.69	2.61	4.30	5.05	4.82	3.81	3.73	7.14	4.31	0.00	2.49	2.79	4.55	7.68	7.62	5.36	5.64	8.09	6.11
**South**	Quarter	1	0.00	1.05	1.49	2.60	4.41	2.49	0.00	0.00	2.07	2.41	0.00	0.00	0.38	4.95	4.07	3.69	3.19	0.66	0.00	3.05
		2	0.00	0.90	1.40	4.58	3.36	3.55	1.61	3.05	3.42	3.03	0.00	3.12	0.96	8.01	7.07	4.96	3.35	4.43	0.00	4.98
		3	0.00	9.01	5.27	12.55	10.94	14.39	8.97	8.29	21.10	11.10	12.75	4.46	8.01	17.26	16.89	25.42	12.13	18.54	22.61	17.41
		4	0.00	0.69	1.64	4.47	4.78	6.69	2.80	2.99	6.15	4.38	0.00	1.49	2.08	6.18	6.04	8.29	2.69	4.40	3.55	5.41
	Year	16/17	0.00	15.00	34.00	95.00	171.00	209.00	41.00	24.00	20.00	609.00	2.00	12.00	37.00	142.00	254.00	326.00	68.00	56.00	29.00	926.00
	Episodes	F/N	0.00	2.88	2.43	6.02	5.82	6.72	3.31	3.57	8.10	5.19	3.13	2.28	2.83	9.08	8.49	10.48	5.30	6.96	6.42	7.66
**London**	Quarter	1	0.00	1.81	2.10	3.54	4.50	3.73	4.47	0.00	0.00	3.60	0.00	1.80	2.63	3.26	5.60	4.79	0.96	3.14	0.00	4.20
		2	0.00	4.03	1.93	5.74	4.63	3.77	3.05	3.49	0.00	4.02	0.00	4.14	3.19	7.90	8.87	9.32	5.25	5.55	2.79	7.49
		3	6.16	9.56	15.19	15.52	13.45	15.82	9.77	15.72	5.35	13.94	7.37	21.63	8.12	20.55	19.83	26.93	21.31	16.67	12.72	19.81
		4	0.00	10.39	8.41	9.10	7.76	6.38	2.22	1.94	5.53	7.35	10.57	10.50	4.66	12.32	10.20	12.69	4.76	7.54	3.22	9.85
	Year	16/17	1.00	30.00	68.00	73.00	241.00	125.00	18.00	11.00	2.00	569.00	3.00	43.00	44.00	104.00	376.00	215.00	34.00	22.00	6.00	847.00
	Episodes	F/N	1.51	6.40	6.81	8.42	7.53	7.36	4.84	5.25	2.67	7.17	4.40	9.42	4.62	10.95	11.08	13.35	8.02	8.18	4.65	10.28
**Midlands And East**	Quarter	1	0.00	0.00	3.50	4.42	2.10	3.26	1.23	0.00	3.03	2.56	0.00	0.00	0.62	2.07	1.49	2.51	1.16	5.82	0.00	1.88
		2	0.00	2.55	0.93	0.52	2.19	2.87	1.53	1.88	0.00	1.91	0.00	1.36	0.50	2.13	5.07	2.79	2.32	3.93	1.51	2.91
		3	0.00	2.54	7.83	9.73	14.02	11.42	9.46	9.96	8.70	10.60	0.00	20.57	5.08	10.60	19.21	16.26	12.84	18.35	10.99	14.77
		4	0.00	2.63	2.50	7.21	5.80	4.14	4.95	3.19	0.00	4.52	0.00	2.75	4.27	6.62	6.84	9.11	3.65	7.74	1.70	6.43
	Year	16/17	0.00	6.00	29.00	39.00	96.00	100.00	32.00	15.00	4.00	321.00	0.00	18.00	20.00	36.00	131.00	143.00	40.00	42.00	9.00	439.00
	Episodes	F/N	0.00	1.94	3.64	5.38	5.96	5.38	4.24	3.72	2.88	4.84	0.00	6.08	2.58	5.29	8.09	7.58	4.94	8.87	3.51	6.43

Mean weekly incidence rate per 100,000 Persons.

Acute Laryngitis/Tracheitis (ICD10: J04)

		M										F									
		<1yr	1-4yrs	5-14yrs	15-24yrs	25-44yrs	45-64yrs	65-74yrs	75-84yrs	85+yrs	All Ages	<1yr	1-4yrs	5-14yrs	15-24yrs	25-44yrs	45-64yrs	65-74yrs	75-84yrs	85+yrs	All Ages
**4 weekly**	1	0.00	31.30	4.13	1.11	1.09	0.97	0.49	2.01	0.00	2.77	0.00	10.34	2.48	2.91	4.29	3.89	2.92	3.09	0.00	3.69
	2	0.00	24.40	2.25	2.10	1.07	0.50	2.13	1.39	2.96	2.39	0.00	13.83	2.65	4.66	4.36	3.29	4.32	1.01	2.26	3.95
	3	0.00	17.35	2.42	0.58	1.05	0.71	0.27	0.60	1.45	1.74	0.00	8.00	2.06	2.86	4.52	3.38	5.55	0.84	3.17	3.70
	4	0.00	15.83	1.00	1.95	0.37	1.03	1.31	2.70	0.00	1.74	0.00	6.87	0.28	3.15	2.89	1.92	1.52	0.42	0.79	2.12
	5	19.47	17.31	4.38	0.41	0.73	0.97	0.00	0.00	2.29	1.91	0.00	11.86	2.44	4.08	1.57	3.31	2.00	0.00	1.37	2.74
	6	25.79	49.15	3.73	0.58	1.37	0.67	0.57	1.04	1.43	3.58	9.98	26.79	3.32	4.59	5.03	3.99	3.11	0.84	0.74	4.91
	7	14.98	51.45	5.96	1.67	0.94	0.63	0.41	0.00	0.00	3.91	7.48	26.37	2.11	3.81	4.87	6.08	3.64	2.08	2.48	5.23
	8	24.75	50.74	6.79	1.56	1.07	1.90	0.97	1.32	0.00	4.52	36.37	37.90	8.41	4.74	6.71	6.33	5.37	2.51	1.89	7.59
	9	20.16	32.36	4.46	1.18	1.33	2.82	1.71	1.46	0.00	3.69	0.00	18.35	1.96	4.56	3.62	6.30	7.01	3.99	10.01	5.21
	10	37.44	52.76	7.69	0.78	1.45	1.82	2.65	1.86	0.00	5.01	49.39	30.31	6.09	4.39	3.95	7.21	2.31	3.69	2.82	6.19
	11	29.70	36.17	4.67	1.21	1.82	1.21	0.26	0.50	0.00	3.50	16.89	19.46	3.38	3.53	4.62	5.43	2.68	1.89	0.81	4.73
	12	59.64	36.76	6.17	1.97	0.60	1.45	1.64	0.48	0.00	3.83	25.35	26.49	3.87	2.55	7.43	4.81	4.13	3.12	3.46	6.02
	13	15.26	20.47	1.93	1.01	1.32	0.97	1.69	4.28	1.53	2.40	10.17	13.41	2.31	2.05	4.73	4.70	4.41	3.09	3.93	4.32
**Quarter**	1	0.00	24.89	3.02	1.25	1.07	0.75	0.92	1.38	1.35	2.33	0.00	10.70	2.40	3.43	4.38	3.55	4.16	1.76	1.67	3.77
	2	15.31	30.86	3.37	0.96	0.86	0.82	0.65	1.07	1.06	2.59	2.85	17.53	1.97	3.97	3.26	3.50	2.38	0.71	0.83	3.51
	3	27.39	46.29	6.51	1.35	1.18	2.00	1.27	1.28	0.00	4.38	21.51	28.35	4.85	4.45	4.98	6.75	4.83	3.39	5.29	6.26
	4	32.19	32.17	4.25	1.41	1.28	1.25	1.47	1.77	0.47	3.32	23.30	19.80	3.54	2.79	5.48	4.89	3.74	2.49	2.52	5.05
**Year**	16/17	42.00	599.00	192.00	59.00	116.00	119.00	41.00	28.00	6.00	1202.00	27.00	330.00	132.00	183.00	486.00	446.00	130.00	50.00	27.00	1811.00
**Episodes**	F/N	18.66	33.50	4.27	1.24	1.09	1.20	1.07	1.37	0.73	3.15	11.75	19.06	3.17	3.67	4.50	4.65	3.75	2.06	2.55	4.63

Mean weekly incidence rate per 100,000 Persons.

Acute Laryngitis/Tracheitis (ICD10: J04)

			M										F									
			<1yr	1-4yrs	5-14yrs	15-24yrs	25-44yrs	45-64yrs	65-74yrs	75-84yrs	85+yrs	All Ages	<1yr	1-4yrs	5-14yrs	15-24yrs	25-44yrs	45-64yrs	65-74yrs	75-84yrs	85+yrs	All Ages
**North**	Quarter	1	0.00	28.49	4.07	1.47	0.67	0.76	1.63	0.59	3.56	2.50	0.00	6.94	2.30	4.41	3.88	4.56	0.92	3.11	0.90	3.61
		2	9.24	31.93	4.12	0.46	0.83	0.48	0.94	2.35	0.00	2.47	11.41	18.32	2.13	4.51	3.59	3.33	1.47	1.89	0.00	3.65
		3	27.61	46.16	7.01	1.14	1.00	1.74	0.62	0.59	0.00	3.82	16.72	28.67	3.49	5.96	5.71	5.48	4.91	3.46	4.00	6.11
		4	36.25	34.82	4.51	0.68	1.83	0.98	1.92	1.82	1.89	3.39	33.05	29.51	4.70	3.21	6.36	4.59	3.66	3.47	4.13	5.78
	Year	16/17	13.00	183.00	65.00	16.00	35.00	32.00	16.00	9.00	3.00	372.00	9.00	102.00	40.00	81.00	151.00	141.00	36.00	24.00	9.00	593.00
	Episodes	F/N	18.10	35.29	4.91	0.93	1.08	0.98	1.27	1.35	1.34	3.03	15.22	20.81	3.13	4.52	4.86	4.47	2.71	2.96	2.22	4.77
**South**	Quarter	1	0.00	26.25	2.98	0.64	0.34	0.99	0.83	0.74	1.86	2.08	0.00	10.34	1.59	2.35	5.64	3.66	3.57	1.24	1.05	3.77
		2	9.50	43.58	3.10	1.36	1.08	0.64	0.67	0.00	1.64	3.06	0.00	18.58	2.39	2.96	3.07	3.57	2.17	0.96	1.75	3.47
		3	46.54	48.71	6.05	2.25	1.09	2.78	2.27	3.53	0.00	4.89	35.10	34.40	4.56	4.56	6.56	8.44	4.64	2.17	1.71	7.20
		4	21.20	39.66	3.78	2.00	0.76	1.58	0.65	2.30	0.00	3.48	12.94	22.93	2.99	2.96	5.09	3.46	3.60	3.30	0.78	4.56
	Year	16/17	13.00	204.00	54.00	24.00	24.00	45.00	13.00	11.00	2.00	390.00	8.00	110.00	37.00	49.00	146.00	143.00	43.00	15.00	6.00	557.00
	Episodes	F/N	19.12	39.63	3.96	1.56	0.82	1.48	1.10	1.61	0.89	3.37	11.78	21.51	2.87	3.20	5.05	4.76	3.47	1.90	1.33	4.73
**London**	Quarter	1	0.00	12.49	2.10	1.00	1.46	0.99	0.00	1.95	0.00	1.98	0.00	7.31	2.62	4.21	2.99	3.21	5.79	1.71	3.00	3.47
		2	0.00	16.34	3.47	0.90	0.82	0.91	0.00	0.00	0.00	2.02	0.00	10.80	0.39	2.06	2.25	3.79	3.46	0.00	0.00	2.74
		3	6.34	45.83	6.62	0.95	1.40	1.18	0.00	0.00	0.00	4.49	0.00	24.90	4.78	2.16	3.78	6.36	4.82	6.22	12.37	5.63
		4	14.09	18.77	3.58	1.80	1.00	0.69	2.18	1.94	0.00	2.59	19.12	6.86	1.68	2.05	4.87	4.92	5.64	1.45	0.00	4.29
	Year	16/17	4.00	108.00	39.00	10.00	37.00	16.00	2.00	2.00	0.00	218.00	4.00	57.00	22.00	24.00	117.00	73.00	21.00	6.00	5.00	329.00
	Episodes	F/N	5.01	23.22	3.93	1.16	1.16	0.94	0.53	0.95	0.00	2.75	4.69	12.43	2.33	2.61	3.45	4.56	4.90	2.30	3.77	4.01
**Midlands And East**	Quarter	1	0.00	32.30	2.95	1.90	1.81	0.25	1.23	2.25	0.00	2.78	0.00	18.20	3.11	2.76	5.03	2.77	6.37	0.97	1.73	4.24
		2	42.49	31.57	2.80	1.13	0.72	1.25	1.01	1.92	2.62	2.82	0.00	22.41	2.97	6.37	4.14	3.30	2.43	0.00	1.56	4.18
		3	29.08	44.49	6.37	1.05	1.23	2.31	2.20	1.01	0.00	4.32	34.23	25.40	6.59	5.10	3.86	6.73	4.93	1.72	3.09	6.11
		4	57.21	35.43	5.12	1.16	1.54	1.77	1.13	1.01	0.00	3.84	28.09	19.90	4.80	2.95	5.61	6.57	2.06	1.74	5.19	5.57
	Year	16/17	12.00	104.00	34.00	9.00	20.00	26.00	10.00	6.00	1.00	222.00	6.00	61.00	33.00	29.00	72.00	89.00	30.00	5.00	7.00	332.00
	Episodes	F/N	32.39	35.87	4.28	1.31	1.31	1.39	1.39	1.55	0.69	3.43	15.29	21.50	4.34	4.33	4.65	4.81	3.92	1.09	2.87	5.01

Mean weekly incidence rate per 100,000 Persons.

Pleurisy (ICD10: R091)

		M										F									
		<1yr	1-4yrs	5-14yrs	15-24yrs	25-44yrs	45-64yrs	65-74yrs	75-84yrs	85+yrs	All Ages	<1yr	1-4yrs	5-14yrs	15-24yrs	25-44yrs	45-64yrs	65-74yrs	75-84yrs	85+yrs	All Ages
**4 weekly**	1	0.00	0.00	0.00	0.00	0.22	0.63	2.83	2.57	6.85	0.73	0.00	0.00	0.00	0.15	0.53	0.80	2.00	1.73	2.42	0.78
	2	0.00	0.00	0.00	0.66	0.58	1.08	2.10	5.06	1.34	0.94	0.00	0.00	0.00	0.82	0.45	0.89	1.38	2.48	1.40	0.80
	3	0.00	0.00	0.47	0.00	0.32	0.50	1.99	2.96	2.85	0.74	0.00	0.00	0.00	0.00	0.51	0.24	0.25	2.45	4.86	0.53
	4	0.00	0.00	0.00	0.00	0.78	0.85	2.24	4.67	9.60	1.15	0.00	0.00	0.00	0.20	0.53	1.12	0.54	1.55	1.60	0.68
	5	0.00	0.00	0.00	0.22	0.10	0.21	1.69	6.59	1.30	0.58	0.00	0.00	0.00	0.38	0.52	0.72	1.67	1.23	0.00	0.63
	6	0.00	0.00	0.00	0.00	0.62	0.31	3.98	2.64	3.74	0.81	0.00	0.00	0.00	0.18	0.98	0.92	1.71	1.26	7.00	0.91
	7	0.00	0.00	0.00	0.21	0.41	1.21	2.39	2.60	16.74	1.10	0.00	0.00	0.00	1.38	1.16	1.95	0.00	0.82	0.72	1.05
	8	0.00	0.00	0.00	0.00	0.20	1.24	5.22	4.15	4.55	1.14	0.00	0.00	0.26	0.20	0.61	1.68	1.02	2.82	1.22	0.96
	9	0.00	0.00	0.00	0.37	0.72	0.77	2.81	4.81	11.81	1.08	0.00	0.00	0.40	0.34	0.82	1.11	1.02	5.76	1.97	1.00
	10	0.00	0.00	0.00	0.00	0.81	0.86	3.90	6.42	2.95	1.08	0.00	0.00	0.24	0.00	1.01	1.10	0.75	2.88	0.63	0.83
	11	0.00	0.00	0.00	0.44	0.42	0.67	2.19	5.71	6.70	1.03	0.00	0.00	0.00	0.17	0.61	2.09	1.76	3.91	3.86	1.17
	12	0.00	0.00	0.00	0.19	0.84	0.59	2.07	5.50	10.11	1.06	0.00	0.00	0.00	0.58	1.21	1.26	1.50	2.69	3.59	1.22
	13	0.00	0.00	0.44	0.55	0.40	0.82	2.67	3.87	6.56	0.96	0.00	0.00	0.42	0.17	0.32	0.72	1.46	1.60	1.41	0.64
**Quarter**	1	0.00	0.00	0.15	0.20	0.36	0.73	2.35	3.46	3.93	0.80	0.00	0.00	0.00	0.31	0.50	0.66	1.27	2.18	2.86	0.71
	2	0.00	0.00	0.00	0.06	0.49	0.56	2.70	4.12	6.09	0.89	0.00	0.00	0.00	0.35	0.68	1.12	1.12	1.27	2.46	0.77
	3	0.00	0.00	0.00	0.18	0.56	0.98	3.58	4.56	9.04	1.08	0.00	0.00	0.28	0.44	0.95	1.40	0.70	3.52	1.40	0.98
	4	0.00	0.00	0.14	0.36	0.54	0.73	2.49	5.44	7.19	1.04	0.00	0.00	0.13	0.28	0.72	1.29	1.61	2.66	2.72	0.99
**Year**	16/17	0.00	0.00	3.00	9.00	52.00	72.00	93.00	90.00	44.00	363.00	0.00	0.00	4.00	20.00	77.00	109.00	50.00	55.00	30.00	345.00
**Episodes**	F/N	0.00	0.00	0.07	0.20	0.49	0.75	2.78	4.39	6.55	0.95	0.00	0.00	0.10	0.35	0.71	1.12	1.17	2.39	2.36	0.86

Mean weekly incidence rate per 100,000 Persons.

Pleurisy (ICD10: R.09)

			M										F									
			<1yr	1-4yrs	5-14yrs	15-24yrs	25-44yrs	45-64yrs	65-74yrs	75-84yrs	85+yrs	All Ages	<1yr	1-4yrs	5-14yrs	15-24yrs	25-44yrs	45-64yrs	65-74yrs	75-84yrs	85+yrs	All Ages
**North**	Quarter	1	0.00	0.00	0.00	0.00	0.25	0.51	3.02	5.36	6.87	0.96	0.00	0.00	0.00	0.23	0.91	0.78	1.55	3.09	1.91	0.90
		2	0.00	0.00	0.00	0.00	0.95	0.59	2.19	4.09	1.67	0.89	0.00	0.00	0.00	0.87	1.24	1.75	1.77	2.87	0.93	1.28
		3	0.00	0.00	0.00	0.00	0.24	1.11	3.62	6.00	12.65	1.27	0.00	0.00	0.32	0.85	1.67	1.78	1.55	1.46	0.00	1.29
		4	0.00	0.00	0.00	0.23	0.75	0.62	2.55	2.43	7.47	0.92	0.00	0.00	0.00	0.63	1.15	1.42	2.73	2.04	3.11	1.27
	Year	16/17	0.00	0.00	0.00	1.00	18.00	23.00	35.00	30.00	16.00	123.00	0.00	0.00	1.00	12.00	39.00	45.00	25.00	19.00	6.00	147.00
	Episodes	F/N	0.00	0.00	0.00	0.06	0.55	0.71	2.83	4.46	7.06	1.01	0.00	0.00	0.08	0.65	1.24	1.44	1.90	2.37	1.48	1.19
**South**	Quarter	1	0.00	0.00	0.00	0.31	0.67	1.15	2.49	3.10	0.00	0.95	0.00	0.00	0.00	0.32	0.66	0.82	1.99	1.27	4.35	0.89
		2	0.00	0.00	0.00	0.25	0.40	0.50	2.63	3.20	6.60	0.87	0.00	0.00	0.00	0.00	0.56	0.77	0.93	1.41	1.74	0.60
		3	0.00	0.00	0.00	0.25	0.13	0.81	2.67	7.30	1.61	0.99	0.00	0.00	0.30	0.49	0.66	1.04	1.26	2.54	2.56	0.92
		4	0.00	0.00	0.54	0.24	0.78	0.73	2.50	8.92	6.22	1.37	0.00	0.00	0.00	0.50	0.36	1.16	1.17	4.46	4.32	1.02
	Year	16/17	0.00	0.00	2.00	4.00	14.00	23.00	30.00	36.00	9.00	118.00	0.00	0.00	1.00	5.00	16.00	28.00	16.00	19.00	14.00	99.00
	Episodes	F/N	0.00	0.00	0.13	0.26	0.49	0.79	2.57	5.59	3.66	1.04	0.00	0.00	0.07	0.32	0.56	0.95	1.33	2.40	3.21	0.85
**London**	Quarter	1	0.00	0.00	0.00	0.50	0.53	0.49	3.27	1.95	5.85	0.65	0.00	0.00	0.00	0.00	0.13	0.26	0.96	1.50	0.00	0.21
		2	0.00	0.00	0.00	0.00	0.36	0.47	2.90	3.45	0.00	0.48	0.00	0.00	0.00	0.00	0.34	0.72	0.85	0.00	5.64	0.42
		3	0.00	0.00	0.00	0.46	0.37	0.73	6.45	3.98	10.56	0.87	0.00	0.00	0.00	0.41	0.49	1.52	0.00	7.56	0.00	0.79
		4	0.00	0.00	0.00	0.44	0.13	0.94	3.24	1.93	0.00	0.50	0.00	0.00	0.00	0.00	0.35	1.01	0.99	1.48	0.00	0.44
	Year	16/17	0.00	0.00	0.00	3.00	11.00	11.00	15.00	6.00	3.00	49.00	0.00	0.00	0.00	1.00	11.00	14.00	3.00	7.00	2.00	38.00
	Episodes	F/N	0.00	0.00	0.00	0.34	0.35	0.65	3.95	2.84	4.03	0.62	0.00	0.00	0.00	0.10	0.33	0.88	0.70	2.59	1.49	0.46
**Midlands And East**	Quarter	1	0.00	0.00	0.58	0.00	0.00	0.75	0.62	3.42	2.97	0.64	0.00	0.00	0.00	0.69	0.30	0.75	0.58	2.88	5.17	0.83
		2	0.00	0.00	0.00	0.00	0.26	0.67	3.07	5.75	16.10	1.31	0.00	0.00	0.00	0.55	0.56	1.25	0.93	0.81	1.51	0.76
		3	0.00	0.00	0.00	0.00	1.50	1.27	1.57	0.97	11.32	1.20	0.00	0.00	0.50	0.00	0.96	1.26	0.00	2.51	3.03	0.92
		4	0.00	0.00	0.00	0.54	0.51	0.63	1.68	8.48	15.07	1.36	0.00	0.00	0.51	0.00	1.01	1.55	1.55	2.64	3.47	1.22
	Year	16/17	0.00	0.00	1.00	1.00	9.00	15.00	13.00	18.00	16.00	73.00	0.00	0.00	2.00	2.00	11.00	22.00	6.00	10.00	8.00	61.00
	Episodes	F/N	0.00	0.00	0.14	0.13	0.56	0.83	1.76	4.68	11.46	1.13	0.00	0.00	0.25	0.31	0.70	1.20	0.77	2.18	3.26	0.93

Mean weekly incidence rate per 100,000 Persons.

Pneumonia and Pneumonitis (ICD10: J12 - J18)

		M										F									
		<1yr	1-4yrs	5-14yrs	15-24yrs	25-44yrs	45-64yrs	65-74yrs	75-84yrs	85+yrs	All Ages	<1yr	1-4yrs	5-14yrs	15-24yrs	25-44yrs	45-64yrs	65-74yrs	75-84yrs	85+yrs	All Ages
**4 weekly**	1	0.00	1.82	0.24	0.00	0.42	0.66	2.37	4.32	5.41	1.01	0.00	2.41	0.40	0.00	0.52	0.94	2.02	7.01	5.07	1.25
	2	0.00	2.35	0.29	0.65	0.83	0.99	1.41	4.95	1.61	1.11	0.00	0.77	0.38	0.26	0.25	1.57	3.61	8.38	8.42	1.53
	3	0.00	0.00	0.00	0.00	0.82	0.46	3.02	5.43	5.60	1.03	0.00	2.05	0.26	0.00	0.59	1.81	3.04	2.68	5.50	1.37
	4	0.00	1.47	0.00	0.21	0.59	0.88	1.78	5.28	13.24	1.27	0.00	2.20	0.36	0.38	0.80	0.89	0.73	1.93	3.73	0.93
	5	0.00	1.11	0.00	0.49	0.10	1.86	3.12	7.63	13.23	1.51	0.00	0.00	0.74	0.19	0.53	1.44	2.41	2.49	3.52	1.07
	6	9.47	3.09	0.33	0.40	0.73	2.04	0.30	0.52	8.37	1.19	0.00	0.65	0.53	0.00	1.16	1.66	2.35	3.01	11.53	1.61
	7	15.40	4.73	0.59	0.00	0.41	1.46	0.96	7.23	16.46	1.70	0.00	1.75	0.25	0.78	0.82	1.90	1.66	5.25	9.08	1.61
	8	0.00	1.91	0.49	0.19	0.32	1.56	4.49	6.70	6.16	1.56	0.00	1.81	0.62	0.00	0.66	1.38	2.94	5.13	6.54	1.47
	9	0.00	0.00	1.42	0.60	0.63	1.07	1.32	6.81	16.85	1.42	0.00	0.00	0.52	0.69	1.11	2.31	3.44	5.85	18.82	2.14
	10	0.00	0.58	0.97	0.00	0.81	2.03	4.36	3.65	25.37	1.80	0.00	3.70	0.25	0.53	0.61	2.28	1.91	6.91	15.31	1.99
	11	0.00	0.56	0.00	0.46	0.93	0.94	3.07	7.50	12.84	1.39	0.00	0.63	0.42	0.47	0.53	1.07	3.51	5.85	6.03	1.36
	12	0.00	0.64	0.00	0.53	0.10	1.67	3.57	5.71	9.62	1.32	0.00	0.69	0.68	1.18	0.94	2.05	2.79	5.76	4.54	1.80
	13	0.00	1.36	0.57	0.00	0.70	1.30	2.09	8.22	10.66	1.19	0.00	2.43	0.00	0.49	0.41	0.61	2.45	2.99	5.05	1.04
**Quarter**	1	0.00	1.42	0.18	0.20	0.67	0.70	2.28	4.85	4.30	1.05	0.00	1.80	0.35	0.08	0.46	1.40	2.82	6.10	6.23	1.37
	2	5.12	2.03	0.19	0.31	0.43	1.60	1.68	4.66	12.24	1.36	0.00	1.01	0.54	0.38	0.83	1.35	1.64	2.56	6.59	1.24
	3	2.14	1.78	0.96	0.24	0.61	1.42	2.68	6.19	17.12	1.61	0.00	1.84	0.43	0.32	0.76	2.03	2.84	6.29	13.56	1.86
	4	0.00	0.79	0.18	0.31	0.56	1.41	3.22	7.02	10.57	1.34	0.00	1.33	0.34	0.72	0.67	1.31	2.84	4.85	5.23	1.42
**Year**	16/17	3.00	28.00	17.00	13.00	60.00	122.00	93.00	98.00	70.00	504.00	0.00	23.00	17.00	18.00	74.00	140.00	91.00	101.00	97.00	561.00
**Episodes**	F/N	1.88	1.52	0.37	0.27	0.57	1.29	2.45	5.66	11.08	1.34	0.00	1.49	0.42	0.37	0.68	1.52	2.52	4.91	7.88	1.47

Mean weekly incidence rate per 100,000 Persons.

Pneumonia and Pneumonitis (ICD10: J12-J18)

			M										F									
			<1yr	1-4yrs	5-14yrs	15-24yrs	25-44yrs	45-64yrs	65-74yrs	75-84yrs	85+yrs	All Ages	<1yr	1-4yrs	5-14yrs	15-24yrs	25-44yrs	45-64yrs	65-74yrs	75-84yrs	85+yrs	All Ages
**North**	Quarter	1	0.00	0.00	0.00	0.30	0.76	0.75	3.66	5.48	3.39	1.20	0.00	0.00	0.32	0.00	0.54	0.76	1.25	3.51	5.57	0.93
		2	0.00	1.50	0.00	0.45	0.58	1.31	1.85	1.73	8.70	1.08	0.00	0.79	0.30	0.22	1.11	0.63	0.87	2.81	6.49	1.02
		3	0.00	0.76	0.90	0.47	0.38	1.88	2.92	5.45	3.60	1.45	0.00	0.00	0.62	0.43	0.39	1.89	1.80	3.95	14.94	1.63
		4	0.00	0.00	0.31	0.22	0.38	0.62	2.92	6.05	9.31	1.11	0.00	0.00	0.00	0.86	0.78	1.27	1.50	2.54	7.20	1.19
	Year	16/17	0.00	3.00	4.00	6.00	17.00	37.00	35.00	31.00	14.00	147.00	0.00	1.00	4.00	7.00	22.00	36.00	18.00	26.00	35.00	149.00
	Episodes	F/N	0.00	0.58	0.30	0.36	0.53	1.15	2.82	4.62	6.30	1.21	0.00	0.21	0.31	0.37	0.71	1.13	1.35	3.19	8.51	1.19
**South**	Quarter	1	0.00	3.02	0.73	0.00	0.33	0.80	2.50	2.34	7.72	1.08	0.00	1.05	0.00	0.32	0.33	2.51	2.00	4.49	6.59	1.58
		2	10.82	0.75	0.00	0.24	0.55	1.26	1.35	3.55	10.34	1.14	0.00	1.51	0.89	0.47	0.38	1.15	2.13	1.47	6.77	1.19
		3	0.00	3.78	1.17	0.50	0.66	1.53	3.55	6.79	15.72	2.03	0.00	0.67	0.63	0.00	1.32	1.79	4.34	6.04	7.76	2.03
		4	0.00	1.48	0.00	0.00	1.00	0.74	3.99	4.17	9.83	1.36	0.00	2.23	0.32	0.00	1.16	1.36	4.45	4.78	5.27	1.75
	Year	16/17	1.00	11.00	6.00	3.00	19.00	33.00	34.00	27.00	27.00	161.00	0.00	7.00	6.00	3.00	24.00	49.00	41.00	32.00	29.00	191.00
	Episodes	F/N	2.86	2.23	0.47	0.19	0.64	1.09	2.82	4.20	10.89	1.40	0.00	1.37	0.47	0.20	0.79	1.69	3.21	4.14	6.60	1.63
**London**	Quarter	1	0.00	2.68	0.00	0.50	0.40	0.49	1.10	5.91	0.00	0.69	0.00	2.82	0.47	0.00	0.38	1.59	4.00	13.50	5.94	1.46
		2	9.64	3.30	0.77	0.00	0.37	2.01	0.00	5.26	14.09	1.21	0.00	0.00	0.42	0.85	1.02	1.89	2.58	2.75	2.74	1.21
		3	8.58	2.60	0.81	0.00	0.64	1.21	2.25	5.73	32.69	1.39	0.00	2.72	0.45	0.84	0.36	2.52	3.76	7.62	18.87	1.68
		4	0.00	1.68	0.39	0.43	0.37	2.53	4.34	13.66	11.21	1.52	0.00	1.70	0.00	0.79	0.23	1.28	1.91	5.94	0.00	0.81
	Year	16/17	2.00	12.00	5.00	2.00	14.00	27.00	7.00	16.00	11.00	96.00	0.00	8.00	3.00	6.00	17.00	29.00	13.00	20.00	9.00	105.00
	Episodes	F/N	4.65	2.58	0.50	0.23	0.44	1.57	1.89	7.60	14.49	1.20	0.00	1.78	0.34	0.62	0.51	1.82	3.05	7.36	6.81	1.29
**Midlands And East**	Quarter	1	0.00	0.00	0.00	0.00	1.20	0.75	1.85	5.69	6.08	1.21	0.00	3.31	0.62	0.00	0.59	0.75	4.05	2.90	6.83	1.52
		2	0.00	2.55	0.00	0.56	0.24	1.83	3.52	8.09	15.82	2.01	0.00	1.72	0.55	0.00	0.80	1.76	0.98	3.22	10.36	1.55
		3	0.00	0.00	0.96	0.00	0.74	1.05	2.02	6.79	16.45	1.58	0.00	3.98	0.00	0.00	0.98	1.91	1.45	7.54	12.69	2.10
		4	0.00	0.00	0.00	0.57	0.50	1.73	1.63	4.21	11.91	1.35	0.00	1.40	1.03	1.21	0.53	1.32	3.51	6.14	8.46	1.93
	Year	16/17	0.00	2.00	2.00	2.00	10.00	25.00	17.00	24.00	18.00	100.00	0.00	7.00	4.00	2.00	11.00	26.00	19.00	23.00	24.00	116.00
	Episodes	F/N	0.00	0.67	0.24	0.29	0.66	1.35	2.28	6.23	12.63	1.55	0.00	2.59	0.55	0.30	0.73	1.44	2.47	4.92	9.60	1.77

Mean weekly incidence rate per 100,000 Persons.

Respiratory System Diseases (ICD10: J00-J99)

		M										F									
		<1yr	1-4yrs	5-14yrs	15-24yrs	25-44yrs	45-64yrs	65-74yrs	75-84yrs	85+yrs	All Ages	<1yr	1-4yrs	5-14yrs	15-24yrs	25-44yrs	45-64yrs	65-74yrs	75-84yrs	85+yrs	All Ages
**4 weekly**	1	0.00	870.78	306.78	146.37	114.41	122.94	210.30	245.13	302.38	197.27	0.00	734.70	270.19	228.69	230.75	202.80	241.12	272.26	301.68	254.04
	2	0.00	821.26	372.37	205.11	153.33	138.10	190.31	271.40	360.05	226.08	178.57	739.41	329.51	285.49	284.51	216.39	256.04	282.18	346.68	289.41
	3	554.95	801.30	292.80	155.85	138.09	125.83	220.94	243.44	326.33	203.74	328.98	722.65	321.77	246.23	248.75	228.66	254.73	282.34	339.59	276.41
	4	291.81	513.52	153.04	115.70	112.97	114.63	193.44	247.81	327.98	155.27	397.93	437.56	141.86	202.20	178.66	168.12	197.64	249.54	310.77	195.25
	5	605.45	638.37	179.80	127.90	107.41	120.30	177.56	250.68	323.27	165.34	525.18	522.55	180.43	213.90	183.82	190.11	207.36	245.87	280.90	213.06
	6	1436.36	1365.90	301.99	196.42	142.91	163.77	269.85	314.84	403.41	261.18	1036.95	1154.59	290.85	346.30	292.13	266.82	302.87	369.09	514.68	343.33
	7	2047.38	1629.37	296.96	171.49	157.60	190.88	291.88	378.61	518.55	291.77	1785.34	1514.03	302.68	318.57	315.39	321.67	355.59	444.10	558.47	390.87
	8	2419.18	1757.90	423.10	186.29	157.56	205.88	340.59	473.47	595.79	332.14	2372.86	1720.69	458.05	307.97	348.50	392.39	435.72	498.74	651.78	459.30
	9	1861.60	1092.50	311.11	200.83	193.14	294.09	448.44	657.27	907.92	345.04	1632.81	1042.51	299.87	323.78	393.29	492.54	570.38	721.96	969.41	483.03
	10	1704.78	1284.21	428.24	229.58	193.39	257.20	339.09	528.48	730.27	342.35	1433.04	1173.80	432.22	369.45	379.25	408.91	431.55	532.85	712.75	453.24
	11	1430.10	1056.33	283.23	173.11	163.05	189.79	284.54	376.06	483.79	264.18	1250.23	888.85	308.91	272.39	311.57	325.72	363.44	408.22	467.81	354.18
	12	1757.57	1034.51	345.96	151.65	152.31	170.86	257.08	376.79	446.49	260.17	1535.50	949.58	356.33	273.49	310.76	292.77	339.86	367.79	419.52	352.79
	13	1322.37	655.05	224.95	146.49	131.40	154.97	236.46	333.51	393.58	209.92	868.76	539.70	223.63	235.32	253.61	245.23	314.30	336.36	381.92	276.06
**Quarter**	1	170.75	834.16	322.66	167.36	133.67	128.49	207.42	252.69	327.49	208.13	156.17	732.44	304.31	251.56	252.83	214.94	249.90	278.41	327.19	271.81
	2	928.55	916.68	216.62	149.35	126.39	139.95	221.47	285.73	370.85	204.30	797.93	793.98	211.83	262.05	231.88	223.97	253.71	307.82	389.76	267.97
	3	2060.47	1467.42	384.19	201.03	178.75	246.82	374.95	535.56	727.79	339.38	1838.81	1383.39	390.89	331.76	367.95	421.48	465.56	571.64	770.25	460.98
	4	1516.97	938.52	289.72	161.04	150.29	175.49	260.06	368.28	451.62	249.02	1252.22	821.20	302.46	267.06	295.25	292.49	343.88	381.33	430.93	333.85
**Year**	16/17	2559.00	18460.00	13113.00	7890.00	15761.00	16610.00	9283.00	6663.00	3207.00	93546.00	2036.00	15975.00	12541.00	13235.00	30732.00	27120.00	12003.00	8597.00	5849.00	128088.00
**Episodes**	F/N	1164.65	1036.88	301.66	169.31	146.88	172.07	265.14	359.15	467.58	249.34	1007.26	930.13	300.67	277.81	285.94	287.01	326.86	383.35	477.84	332.41

Mean weekly incidence rate per 100,000 Persons.

Respiratory System Diseases (ICD10: J00-J99)

			M										F									
			<1yr	1-4yrs	5-14yrs	15-24yrs	25-44yrs	45-64yrs	65-74yrs	75-84yrs	85+yrs	All Ages	<1yr	1-4yrs	5-14yrs	15-24yrs	25-44yrs	45-64yrs	65-74yrs	75-84yrs	85+yrs	All Ages
**North**	Quarter	1	354.01	884.56	312.76	150.25	135.41	136.22	206.95	268.07	361.49	207.87	98.34	749.56	309.42	224.27	264.18	217.72	244.24	282.42	313.29	270.85
		2	1,062.52	1,049.62	234.49	143.53	138.35	147.55	240.63	284.12	406.78	216.83	964.65	909.98	232.92	260.11	245.78	248.34	273.92	334.81	410.64	289.80
		3	1,968.48	1,413.57	356.59	181.23	171.59	245.66	384.56	523.01	738.20	324.59	1,891.81	1,377.58	385.73	289.47	372.68	420.30	479.96	562.47	765.53	455.65
		4	1,631.09	967.34	271.84	140.41	143.30	169.33	280.63	351.99	497.80	240.62	1,301.12	827.10	306.72	230.80	303.38	291.99	343.37	394.04	467.52	332.40
	Year	16/17	764.00	5,584.00	3,864.00	2,635.00	4,778.00	5,623.00	3,472.00	2,374.00	1,117.00	30,211.00	613.00	4,699.00	3,905.00	4,531.00	9,188.00	9,270.00	4,420.00	3,174.00	1,983.00	41,783.00
	Episodes	F/N	1,250.41	1,078.22	292.80	153.66	147.00	174.18	277.48	355.43	499.29	246.90	1,062.10	965.00	307.27	251.33	295.55	293.71	334.21	392.33	487.77	336.28
**South**	Quarter	1	136.55	757.87	258.86	144.17	125.15	109.07	176.43	217.94	329.25	181.62	315.93	613.11	251.75	239.79	232.08	196.94	207.44	226.52	348.19	243.41
		2	857.53	740.30	161.99	141.09	107.95	122.60	179.59	243.37	323.02	172.83	636.03	641.59	161.15	258.40	208.60	188.78	205.75	254.36	340.82	231.88
		3	1,918.12	1,276.83	300.43	182.36	162.44	210.97	332.49	469.47	657.90	298.09	1,608.48	1,150.41	298.91	311.30	344.02	370.17	401.66	477.81	688.54	409.31
		4	1,288.94	745.63	233.63	145.44	137.77	157.65	232.88	317.26	363.46	215.66	1,047.07	678.41	237.81	249.01	267.92	244.07	282.91	305.78	410.96	288.43
	Year	16/17	645.00	4,459.00	3,175.00	2,330.00	3,857.00	4,532.00	2,721.00	1,993.00	1,029.00	24,741.00	503.00	3,804.00	2,958.00	4,010.00	7,636.00	7,467.00	3,438.00	2,453.00	1,966.00	34,235.00
	Episodes	F/N	1,046.65	877.52	237.28	153.03	132.85	149.55	229.39	310.72	416.61	216.22	896.86	768.44	235.97	264.51	262.13	248.84	273.14	314.96	445.12	292.10
**London**	Quarter	1	111.48	904.61	387.25	187.72	131.26	145.16	232.18	269.37	259.57	228.74	127.70	862.93	370.18	236.57	234.38	242.07	309.68	332.63	310.65	296.33
		2	866.77	985.55	256.54	163.25	122.86	150.87	242.58	302.21	358.17	216.90	867.04	878.04	260.94	253.87	205.62	241.44	281.30	352.39	384.74	276.62
		3	2,192.90	1,678.43	525.35	225.38	169.81	269.65	382.31	595.82	789.27	371.62	1,856.34	1,524.49	506.11	314.07	332.33	473.57	524.72	688.24	795.25	482.49
		4	1,626.75	1,041.92	386.92	184.42	148.09	195.80	242.83	428.80	332.69	272.83	1,329.89	934.30	364.90	262.80	274.85	325.47	399.65	449.53	372.38	353.87
	Year	16/17	734.00	5,354.00	3,809.00	1,626.00	4,565.00	3,223.00	1,029.00	825.00	330.00	21,495.00	583.00	4,789.00	3,542.00	2,500.00	8,820.00	5,100.00	1,603.00	1,228.00	605.00	28,770.00
	Episodes	F/N	1,193.20	1,149.47	386.51	189.68	142.62	189.62	274.37	397.22	433.48	271.47	1,041.88	1,046.70	373.37	266.58	260.73	319.15	377.00	453.75	464.23	350.90
**Midlands And East**	Quarter	1	80.97	789.61	331.77	187.29	142.87	123.54	214.14	255.39	359.66	214.28	82.71	704.16	285.90	305.63	280.68	203.03	238.24	272.08	336.64	276.64
		2	927.37	891.23	213.44	149.52	136.40	138.79	223.06	313.22	395.45	210.64	724.02	746.31	192.33	275.81	267.53	217.31	253.89	289.72	422.86	273.60
		3	2,162.38	1,500.86	354.41	215.14	211.14	261.01	400.43	553.93	725.77	363.21	1,998.60	1,481.10	372.81	412.21	422.76	421.86	455.91	558.03	831.68	496.47
		4	1,521.10	999.18	266.49	173.91	172.00	179.20	283.90	375.07	612.53	266.97	1,330.80	844.98	300.42	325.64	334.86	308.42	349.61	375.98	472.86	360.71
	Year	16/17	416.00	3,063.00	2,265.00	1,299.00	2,561.00	3,232.00	2,061.00	1,471.00	731.00	17,099.00	337.00	2,683.00	2,136.00	2,194.00	5,088.00	5,283.00	2,542.00	1,742.00	1,295.00	23,300.00
	Episodes	F/N	1,168.32	1,042.32	290.06	180.86	165.05	174.94	279.30	373.25	520.94	262.77	1,028.18	940.41	286.06	328.81	325.34	286.33	323.08	372.36	514.25	350.38

Mean weekly incidence rate per 100,000 Persons.

Sinusitis (ICD10: J01)

		M										F									
		<1yr	1-4yrs	5-14yrs	15-24yrs	25-44yrs	45-64yrs	65-74yrs	75-84yrs	85+yrs	All Ages	<1yr	1-4yrs	5-14yrs	15-24yrs	25-44yrs	45-64yrs	65-74yrs	75-84yrs	85+yrs	All Ages
**4 weekly**	1	0.00	1.15	1.93	4.70	9.93	12.40	10.31	10.94	3.20	8.49	0.00	0.59	0.96	10.97	26.14	30.09	26.51	10.56	4.88	19.55
	2	0.00	0.00	1.88	6.65	10.98	9.47	12.43	8.49	2.86	8.19	0.00	2.93	2.43	10.68	33.39	25.42	20.76	14.72	5.90	20.29
	3	0.00	0.64	2.69	7.96	10.36	9.43	11.52	8.76	10.22	8.49	0.00	0.00	1.70	12.18	27.40	31.58	23.88	11.39	11.13	20.58
	4	0.00	0.73	1.75	4.46	6.44	10.60	13.53	6.47	2.79	7.07	0.00	0.71	1.87	9.71	23.10	24.56	27.76	7.77	8.36	17.10
	5	0.00	0.00	0.83	4.46	10.64	10.04	10.67	10.68	2.29	7.63	0.00	0.00	1.79	8.92	21.75	24.34	26.20	12.06	6.49	16.85
	6	0.00	0.70	3.19	7.24	12.75	15.71	14.21	5.90	5.08	10.83	0.00	0.70	6.13	22.72	41.22	35.76	29.04	17.63	13.51	27.67
	7	0.00	0.73	2.56	7.20	14.38	17.16	15.80	15.19	3.55	11.92	7.48	1.39	2.30	19.45	50.44	47.29	39.79	13.64	7.88	33.04
	8	6.71	2.99	3.51	7.84	15.63	14.67	18.40	11.83	5.00	12.40	6.61	1.45	4.05	21.89	56.24	53.57	42.68	18.50	7.46	36.89
	9	0.00	0.69	4.26	12.94	19.65	26.29	22.76	13.10	9.60	17.56	0.00	0.00	3.24	26.02	63.80	68.54	49.33	24.13	7.33	44.27
	10	0.00	2.04	5.06	13.45	19.32	21.57	16.02	5.75	4.05	15.24	5.44	0.66	7.03	26.53	66.14	53.78	48.99	21.00	8.64	41.68
	11	3.86	0.00	3.17	12.49	16.80	19.66	20.29	10.34	4.37	14.22	0.00	1.37	2.86	19.75	54.13	55.26	42.79	22.41	5.28	36.66
	12	3.63	1.26	2.39	9.71	14.28	15.76	13.95	15.32	9.71	11.65	0.00	2.51	4.16	19.14	46.69	41.85	40.98	20.01	9.26	31.50
	13	0.00	0.00	3.93	7.24	12.35	14.38	16.98	9.82	5.41	10.83	0.00	0.69	2.46	14.65	37.23	37.38	34.12	16.74	8.15	25.96
**Quarter**	1	0.00	0.64	2.15	6.30	10.39	10.59	11.33	9.52	5.26	8.40	0.00	1.13	1.64	11.25	28.76	29.11	23.93	12.10	7.12	20.09
	2	0.00	0.62	1.76	5.88	10.43	12.41	13.50	9.47	3.55	8.91	0.00	0.60	2.95	14.73	31.93	30.70	28.24	12.95	9.14	22.22
	3	2.07	1.54	3.92	10.09	17.65	20.72	18.32	10.69	6.13	14.59	6.01	0.87	4.29	23.57	60.03	57.99	46.59	19.18	8.52	39.89
	4	2.30	0.61	3.61	10.35	14.89	16.94	17.18	11.22	6.00	12.54	0.00	1.41	3.58	18.64	47.35	45.05	40.44	20.37	6.98	32.06
**Year**	16/17	3.00	16.00	128.00	383.00	1434.00	1468.00	553.00	194.00	37.00	4216.00	3.00	17.00	134.00	817.00	4430.00	3921.00	1293.00	370.00	98.00	11083.00
**Episodes**	F/N	1.07	0.85	2.84	8.11	13.28	15.11	15.05	10.21	5.20	11.07	1.47	0.99	3.11	17.01	41.83	40.52	34.68	16.09	7.96	28.45

Mean weekly incidence rate per 100,000 Persons.

Sinusitis (ICD10: J01)

			M										F									
			<1yr	1-4yrs	5-14yrs	15-24yrs	25-44yrs	45-64yrs	65-74yrs	75-84yrs	85+yrs	All Ages	<1yr	1-4yrs	5-14yrs	15-24yrs	25-44yrs	45-64yrs	65-74yrs	75-84yrs	85+yrs	All Ages
**North**	Quarter	1	0.00	1.67	2.52	5.51	11.67	12.25	10.89	9.70	3.39	9.18	0.00	0.00	3.92	7.77	33.03	29.38	27.88	14.84	3.83	21.53
		2	0.00	0.00	1.76	6.03	11.65	10.73	14.62	10.45	1.75	9.08	0.00	2.38	3.63	17.62	35.20	36.60	29.85	12.49	8.51	25.44
		3	0.00	0.76	6.34	9.56	19.00	21.89	19.95	15.09	9.22	15.89	0.00	1.67	5.10	20.30	65.57	60.16	44.70	18.44	13.02	41.50
		4	0.00	0.00	4.82	9.25	15.11	15.95	18.64	12.70	7.51	12.74	0.00	1.68	2.85	16.17	49.61	46.05	39.19	17.37	13.40	32.44
	Year	16/17	0.00	3.00	51.00	131.00	465.00	488.00	200.00	80.00	12.00	1,430.00	0.00	7.00	49.00	284.00	1,422.00	1,357.00	467.00	127.00	39.00	3,752.00
	Episodes	F/N	0.00	0.60	3.82	7.56	14.31	15.12	16.00	11.96	5.40	11.67	0.00	1.45	3.87	15.50	45.65	42.93	35.30	15.72	9.67	30.14
**South**	Quarter	1	0.00	0.00	0.37	3.23	9.90	9.09	11.22	7.02	9.57	7.21	0.00	1.04	1.58	11.89	27.95	32.14	19.44	11.49	8.53	20.22
		2	0.00	0.00	0.87	8.07	10.95	10.65	12.16	9.57	4.63	8.71	0.00	0.00	1.53	15.22	28.45	26.68	25.08	13.23	7.81	19.94
		3	0.00	0.00	2.46	10.56	16.11	18.69	22.05	6.09	4.55	13.42	0.00	0.00	4.40	21.59	59.39	59.03	44.19	14.73	5.30	39.01
		4	0.00	0.71	2.41	6.61	17.90	16.84	15.67	12.29	4.62	12.56	0.00	0.00	3.20	16.43	42.59	40.87	37.50	18.54	7.93	28.98
	Year	16/17	0.00	1.00	22.00	111.00	402.00	419.00	179.00	56.00	14.00	1,204.00	0.00	1.00	34.00	249.00	1,159.00	1,180.00	399.00	113.00	32.00	3,167.00
	Episodes	F/N	0.00	0.17	1.51	7.14	13.66	13.76	15.22	8.76	5.82	10.44	0.00	0.25	2.65	16.26	39.39	39.43	31.43	14.48	7.40	26.90
**London**	Quarter	1	0.00	0.89	3.37	7.02	7.93	8.71	7.86	12.26	5.07	7.09	0.00	1.86	0.44	10.94	21.77	25.11	25.20	7.51	9.23	17.09
		2	0.00	2.46	3.51	6.25	8.83	13.88	12.85	14.12	0.00	8.82	0.00	0.00	4.05	12.78	22.16	27.10	33.65	10.92	11.43	18.70
		3	8.26	5.41	4.52	11.67	15.41	19.34	11.95	7.89	5.35	13.39	24.04	1.81	5.61	20.11	42.94	51.34	52.08	22.95	9.60	34.47
		4	9.22	1.71	3.65	12.11	10.31	15.74	10.71	11.54	5.53	10.33	0.00	2.51	2.93	15.43	35.29	40.81	44.38	19.57	3.24	27.60
	Year	16/17	3.00	12.00	37.00	80.00	339.00	245.00	41.00	24.00	3.00	784.00	3.00	7.00	31.00	139.00	1,029.00	574.00	165.00	41.00	11.00	2,000.00
	Episodes	F/N	4.29	2.62	3.75	9.21	10.59	14.41	10.88	11.50	3.91	9.89	5.90	1.51	3.27	14.78	30.38	35.92	38.73	15.16	8.43	24.36
**Midlands And East**	Quarter	1	0.00	0.00	2.35	9.46	12.06	12.29	15.36	9.10	2.99	10.10	0.00	1.62	0.62	14.42	32.28	29.82	23.22	14.55	6.88	21.54
		2	0.00	0.00	0.91	3.17	10.28	14.36	14.37	3.75	7.83	9.02	0.00	0.00	2.58	13.30	41.91	32.42	24.40	15.16	8.81	24.80
		3	0.00	0.00	2.37	8.59	20.07	22.95	19.32	13.70	5.40	15.68	0.00	0.00	2.04	32.30	72.20	61.42	45.38	20.62	6.14	44.59
		4	0.00	0.00	3.58	13.43	16.23	19.24	23.68	8.37	6.33	14.53	0.00	1.43	5.35	26.54	61.91	52.47	40.67	26.01	3.35	39.21
	Year	16/17	0.00	0.00	18.00	61.00	228.00	316.00	133.00	34.00	8.00	798.00	0.00	2.00	20.00	145.00	820.00	810.00	262.00	89.00	16.00	2,164.00
	Episodes	F/N	0.00	0.00	2.28	8.56	14.58	17.16	18.11	8.63	5.68	12.27	0.00	0.75	2.65	21.48	51.88	43.81	33.25	19.01	6.34	32.39

Mean weekly incidence rate per 100,000 Persons.

Strep Sore Throat, Scarlatina and Peritonsillar Abscess (ICD10: A38,J020,J36)

		M										F									
		<1yr	1-4yrs	5-14yrs	15-24yrs	25-44yrs	45-64yrs	65-74yrs	75-84yrs	85+yrs	All Ages	<1yr	1-4yrs	5-14yrs	15-24yrs	25-44yrs	45-64yrs	65-74yrs	75-84yrs	85+yrs	All Ages
**4 weekly**	1	0.00	10.28	4.76	0.00	1.07	0.27	0.00	0.00	0.00	1.39	0.00	10.19	5.24	0.53	0.66	0.22	0.00	0.00	0.00	1.30
	2	0.00	8.71	4.10	1.52	0.93	0.33	0.00	0.00	0.00	1.42	0.00	11.09	6.84	2.41	0.45	0.47	0.00	0.00	0.00	1.81
	3	0.00	11.41	3.42	0.60	0.87	0.85	0.00	0.50	0.00	1.48	0.00	13.42	5.49	0.26	0.96	0.38	0.00	0.00	0.00	1.67
	4	0.00	3.31	0.67	0.71	0.59	0.00	0.33	0.00	0.00	0.54	0.00	4.92	2.26	0.69	1.08	0.13	0.55	0.00	0.00	0.91
	5	0.00	6.28	0.81	0.20	1.19	0.00	0.00	0.99	0.00	0.78	0.00	2.05	1.29	1.43	0.31	0.00	0.00	1.16	0.00	0.53
	6	0.00	9.09	2.78	1.24	0.21	0.61	0.74	0.00	0.00	1.22	0.00	3.40	2.53	0.55	0.41	0.00	0.00	0.00	0.00	0.64
	7	0.00	6.24	2.91	0.37	0.40	0.56	0.00	0.00	0.00	0.94	0.00	8.96	2.95	0.94	1.01	0.58	0.25	0.00	0.00	1.29
	8	0.00	12.22	7.25	1.63	1.24	0.32	0.00	0.00	1.29	2.11	0.00	7.80	6.18	0.79	1.73	0.62	0.00	0.00	0.00	1.71
	9	0.00	4.95	2.44	1.45	0.90	0.43	0.26	0.00	0.00	1.09	0.00	8.62	2.75	1.82	1.53	0.30	0.00	0.00	0.00	1.40
	10	0.00	17.47	6.14	1.61	0.80	0.18	0.24	0.00	0.00	2.06	0.00	11.82	10.76	0.40	0.93	0.31	0.00	0.00	0.00	2.11
	11	10.81	28.51	6.10	0.91	0.41	0.36	0.44	0.00	0.00	2.51	0.00	18.91	10.46	2.03	0.93	0.62	0.00	0.80	0.00	2.69
	12	3.54	32.67	11.39	0.81	1.46	0.48	0.00	0.00	0.00	3.58	3.82	27.57	12.75	2.60	1.26	0.10	0.00	0.00	0.00	3.28
	13	0.00	23.81	3.31	1.28	0.10	0.10	0.00	0.00	0.00	1.73	10.17	16.31	6.31	0.79	1.53	0.00	0.24	0.00	0.00	2.00
**Quarter**	1	0.00	10.14	4.14	0.65	0.96	0.47	0.00	0.15	0.00	1.43	0.00	11.46	5.81	1.03	0.69	0.35	0.00	0.00	0.00	1.57
	2	0.00	6.23	1.53	0.62	0.63	0.25	0.31	0.28	0.00	0.85	0.00	3.27	2.09	0.87	0.60	0.16	0.23	0.33	0.00	0.72
	3	0.00	10.28	5.02	1.37	0.96	0.32	0.15	0.00	0.40	1.63	0.00	10.53	5.55	1.04	1.37	0.43	0.00	0.00	0.00	1.68
	4	4.42	27.49	6.81	1.10	0.61	0.34	0.13	0.00	0.00	2.56	4.30	19.90	10.12	1.73	1.28	0.22	0.07	0.25	0.00	2.63
**Year**	16/17	3.00	239.00	197.00	51.00	76.00	32.00	6.00	3.00	1.00	608.00	3.00	196.00	250.00	57.00	104.00	27.00	4.00	3.00	0.00	644.00
**Episodes**	F/N	1.08	13.40	4.32	0.93	0.79	0.34	0.15	0.11	0.10	1.60	1.06	11.14	5.82	1.16	0.98	0.29	0.08	0.15	0.00	1.64

Mean weekly incidence rate per 100,000 Persons.

Strep Sore Throat, Scarlatina and Peritonsillar Abscess (ICD10: A38,J020,J36)

			M										F									
			<1yr	1-4yrs	5-14yrs	15-24yrs	25-44yrs	45-64yrs	65-74yrs	75-84yrs	85+yrs	All Ages	<1yr	1-4yrs	5-14yrs	15-24yrs	25-44yrs	45-64yrs	65-74yrs	75-84yrs	85+yrs	All Ages
**North**	Quarter	1	0.00	8.17	4.09	1.45	0.64	0.38	0.00	0.61	0.00	1.30	0.00	7.68	4.62	0.95	0.90	0.13	0.00	0.00	0.00	1.18
		2	0.00	3.72	1.17	0.66	0.12	0.12	0.00	0.57	0.00	0.47	0.00	3.94	1.21	1.49	0.37	0.00	0.59	0.00	0.00	0.65
		3	0.00	9.21	4.30	1.14	1.12	0.51	0.33	0.00	0.00	1.48	0.00	9.09	4.73	1.07	1.42	0.25	0.00	0.00	0.00	1.42
		4	9.09	29.30	6.93	1.80	0.12	0.49	0.00	0.00	0.00	2.47	0.00	28.71	11.96	1.05	2.07	0.51	0.00	0.48	0.00	3.18
	Year	16/17	2.00	65.00	54.00	22.00	16.00	12.00	1.00	2.00	0.00	174.00	0.00	60.00	71.00	21.00	37.00	7.00	2.00	1.00	0.00	199.00
	Episodes	F/N	2.23	12.43	4.07	1.25	0.49	0.37	0.08	0.30	0.00	1.41	0.00	12.20	5.55	1.15	1.17	0.22	0.15	0.12	0.00	1.59
**South**	Quarter	1	0.00	12.94	6.02	0.65	1.02	0.50	0.00	0.00	0.00	1.75	0.00	19.81	6.82	1.29	0.52	1.01	0.00	0.00	0.00	2.10
		2	0.00	3.66	2.55	1.31	1.06	0.27	0.71	0.57	0.00	1.09	0.00	4.80	0.30	0.48	0.74	0.39	0.33	0.00	0.00	0.62
		3	0.00	12.50	3.40	1.23	0.40	0.13	0.29	0.00	1.58	1.33	0.00	14.26	5.70	1.00	0.52	0.28	0.00	0.00	0.00	1.55
		4	0.00	26.63	9.60	1.74	1.15	0.00	0.00	0.00	0.00	2.87	4.01	19.67	13.34	1.97	1.57	0.12	0.29	0.50	0.00	3.07
	Year	16/17	0.00	72.00	72.00	19.00	26.00	6.00	3.00	1.00	1.00	200.00	1.00	70.00	82.00	18.00	24.00	12.00	2.00	1.00	0.00	210.00
	Episodes	F/N	0.00	13.74	5.34	1.23	0.91	0.22	0.26	0.15	0.39	1.75	0.98	14.45	6.42	1.17	0.83	0.45	0.16	0.12	0.00	1.81
**London**	Quarter	1	0.00	8.11	2.95	0.50	0.39	0.74	0.00	0.00	0.00	1.23	0.00	11.80	6.22	0.50	0.74	0.00	0.00	0.00	0.00	1.76
		2	0.00	3.36	2.39	0.49	0.60	0.22	0.00	0.00	0.00	0.83	0.00	1.70	4.41	0.40	0.45	0.23	0.00	1.33	0.00	0.93
		3	0.00	11.50	7.11	1.00	0.38	0.24	0.00	0.00	0.00	1.88	0.00	8.00	5.60	0.41	1.08	0.78	0.00	0.00	0.00	1.74
		4	0.00	26.89	5.16	0.88	0.36	0.23	0.00	0.00	0.00	2.49	4.70	12.94	8.27	1.59	0.69	0.26	0.00	0.00	0.00	2.24
	Year	16/17	0.00	58.00	43.00	6.00	14.00	6.00	0.00	0.00	0.00	127.00	1.00	39.00	58.00	7.00	25.00	5.00	0.00	1.00	0.00	136.00
	Episodes	F/N	0.00	12.30	4.36	0.71	0.44	0.35	0.00	0.00	0.00	1.60	1.15	8.48	6.10	0.72	0.74	0.31	0.00	0.35	0.00	1.65
**Midlands And East**	Quarter	1	0.00	11.35	3.52	0.00	1.80	0.25	0.00	0.00	0.00	1.42	0.00	6.55	5.57	1.36	0.60	0.25	0.00	0.00	0.00	1.25
		2	0.00	14.16	0.00	0.00	0.75	0.40	0.51	0.00	0.00	0.99	0.00	2.65	2.43	1.12	0.87	0.00	0.00	0.00	0.00	0.71
		3	0.00	7.92	5.29	2.12	1.95	0.40	0.00	0.00	0.00	1.82	0.00	10.78	6.18	1.66	2.47	0.41	0.00	0.00	0.00	2.03
		4	8.58	27.14	5.55	0.00	0.79	0.66	0.54	0.00	0.00	2.40	8.51	18.28	6.91	2.29	0.78	0.00	0.00	0.00	0.00	2.04
	Year	16/17	1.00	44.00	28.00	4.00	20.00	8.00	2.00	0.00	0.00	107.00	1.00	27.00	39.00	11.00	18.00	3.00	0.00	0.00	0.00	99.00
	Episodes	F/N	2.10	15.12	3.52	0.52	1.31	0.43	0.27	0.00	0.00	1.64	2.09	9.43	5.22	1.60	1.17	0.16	0.00	0.00	0.00	1.49

Mean weekly incidence rate per 100,000 Persons.

Acute Tonsillitis/Pharyngitis (ICD10: J02-J03)

		M										F									
		<1yr	1-4yrs	5-14yrs	15-24yrs	25-44yrs	45-64yrs	65-74yrs	75-84yrs	85+yrs	All Ages	<1yr	1-4yrs	5-14yrs	15-24yrs	25-44yrs	45-64yrs	65-74yrs	75-84yrs	85+yrs	All Ages
**4 weekly**	1	0.00	130.91	84.75	41.00	25.88	7.94	5.42	4.34	5.22	31.42	0.00	111.45	94.36	93.53	62.39	19.97	9.75	10.08	1.82	50.32
	2	0.00	163.41	90.28	43.28	28.50	9.91	3.08	5.66	2.99	35.04	0.00	141.07	111.29	101.76	59.81	17.73	8.42	7.71	5.53	53.00
	3	0.00	162.45	90.42	45.45	31.00	9.14	4.63	5.32	6.82	36.05	0.00	144.80	120.36	100.58	61.32	20.60	10.04	2.18	0.91	55.38
	4	0.00	96.32	43.67	44.88	31.30	11.02	9.13	8.07	9.90	28.08	0.00	83.10	53.45	93.66	48.46	16.24	9.12	7.45	7.75	39.73
	5	15.02	106.90	52.51	42.92	21.15	6.93	6.71	5.90	6.04	25.77	0.00	93.13	59.26	90.83	48.48	17.57	12.95	5.87	2.09	41.12
	6	17.98	165.36	69.08	53.85	21.29	9.73	6.78	5.67	2.90	32.36	0.00	136.44	88.72	110.94	48.67	18.83	10.86	5.87	6.01	49.29
	7	24.24	164.45	68.75	50.11	25.17	8.37	6.39	5.58	2.91	32.68	17.16	160.98	83.13	105.83	53.10	15.48	9.13	8.23	5.16	49.66
	8	23.18	170.76	90.15	59.10	22.46	8.75	8.46	7.13	6.63	36.14	11.02	176.28	117.95	102.35	58.74	20.14	8.42	5.15	11.13	56.82
	9	33.82	126.22	71.13	45.81	27.60	9.11	7.27	7.10	2.76	31.63	26.98	133.63	82.57	94.39	63.93	23.52	8.15	7.77	2.36	51.91
	10	31.77	174.29	104.68	66.30	30.09	12.31	5.86	8.46	7.40	41.99	19.39	178.64	130.84	128.85	64.18	20.51	10.53	9.02	0.70	62.51
	11	27.11	200.54	90.32	58.60	31.74	8.27	3.18	4.66	6.25	39.37	33.96	160.74	115.41	106.69	70.29	27.40	6.71	7.32	2.87	60.46
	12	91.44	233.91	114.75	50.82	35.98	11.18	6.52	7.38	1.53	45.96	62.37	181.21	140.70	127.06	76.25	26.00	13.26	3.51	7.63	68.57
	13	78.33	149.44	63.27	53.15	27.23	10.80	5.93	3.74	3.08	33.24	44.67	126.15	85.91	98.30	62.88	20.51	9.31	8.68	2.12	51.75
**Quarter**	1	0.00	150.62	88.20	43.07	28.26	8.91	4.46	5.05	5.03	33.96	0.00	130.82	107.57	98.23	61.26	19.48	9.43	6.92	2.68	52.70
	2	16.35	126.30	55.06	47.03	24.35	8.93	7.33	6.20	5.38	28.70	2.68	110.78	65.69	98.82	48.94	17.03	10.85	7.29	4.53	43.66
	3	25.88	159.89	87.89	55.65	26.76	9.85	6.84	7.15	6.06	36.36	18.53	163.32	109.91	107.77	60.87	20.73	9.39	6.07	5.95	56.33
	4	62.01	192.76	89.22	55.40	31.31	10.22	5.64	5.77	3.34	39.57	44.91	157.46	114.10	111.62	69.50	24.38	9.22	7.27	3.88	60.29
**Year**	16/17	71.00	2752.00	3457.00	2390.00	2908.00	890.00	200.00	108.00	34.00	12810.00	44.00	2346.00	4122.00	4960.00	6339.00	1855.00	333.00	147.00	50.00	20196.00
**Episodes**	F/N	25.88	156.81	79.62	50.23	27.61	9.47	6.09	6.05	4.96	34.53	16.27	140.04	98.68	104.01	59.93	20.34	9.74	6.89	4.27	53.07

Mean weekly incidence rate per 100,000 Persons.

Acute Tonsillitis/Pharyngitis (ICD10: J02-J03)

			M										F									
			<1yr	1-4yrs	5-14yrs	15-24yrs	25-44yrs	45-64yrs	65-74yrs	75-84yrs	85+yrs	All Ages	<1yr	1-4yrs	5-14yrs	15-24yrs	25-44yrs	45-64yrs	65-74yrs	75-84yrs	85+yrs	All Ages
**North**	Quarter	1	0.00	136.96	95.13	42.21	29.88	7.28	4.60	4.28	12.16	32.70	0.00	136.82	115.34	89.81	64.19	16.97	5.23	7.52	5.59	51.27
		2	20.54	144.37	56.81	47.57	23.35	6.99	4.38	6.94	6.73	28.10	10.71	112.41	77.38	95.74	50.80	16.17	9.47	8.59	5.55	45.09
		3	36.95	144.28	97.05	54.06	24.17	11.33	5.44	3.62	9.08	34.81	13.24	157.72	123.90	101.56	60.20	19.45	7.29	6.97	2.04	55.34
		4	74.03	186.12	79.58	50.74	30.41	8.94	4.52	4.82	7.53	35.42	23.49	145.27	119.82	103.40	74.16	21.00	7.50	5.56	5.09	58.31
	Year	16/17	25.00	791.00	1,078.00	837.00	873.00	278.00	59.00	33.00	20.00	3,994.00	8.00	670.00	1,379.00	1,756.00	1,930.00	579.00	98.00	58.00	19.00	6,497.00
	Episodes	F/N	32.65	152.77	81.66	48.62	26.88	8.61	4.73	4.95	8.84	32.67	11.84	137.57	108.51	97.59	62.12	18.36	7.41	7.19	4.59	52.36
**South**	Quarter	1	0.00	150.88	71.96	43.68	25.34	7.42	6.72	6.21	1.91	30.40	0.00	109.30	88.88	102.09	57.57	15.88	8.36	3.86	2.17	47.11
		2	36.17	94.92	37.48	45.27	22.22	8.23	6.14	6.88	1.64	23.71	0.00	83.80	50.67	102.70	43.56	15.53	7.90	3.59	0.81	38.29
		3	24.57	130.35	71.39	55.92	25.41	7.23	6.63	7.23	1.52	31.48	15.00	120.02	78.54	103.26	56.04	16.00	7.93	3.16	4.43	46.50
		4	50.47	149.22	68.61	51.46	26.71	9.49	6.08	5.35	0.00	32.34	32.10	124.12	94.86	107.10	60.95	18.04	8.95	6.11	3.71	50.91
	Year	16/17	19.00	656.00	824.00	748.00	718.00	241.00	73.00	40.00	3.00	3,322.00	9.00	535.00	973.00	1,561.00	1,564.00	482.00	101.00	32.00	12.00	5,269.00
	Episodes	F/N	27.96	130.65	61.89	49.01	24.87	8.10	6.39	6.43	1.27	29.37	11.55	108.83	77.72	103.77	54.32	16.35	8.28	4.17	2.74	45.56
**London**	Quarter	1	0.00	155.46	94.68	40.37	24.35	12.19	3.43	6.32	0.00	38.37	0.00	128.81	111.62	73.69	53.69	21.74	16.62	12.44	2.96	56.42
		2	8.70	130.53	64.95	36.72	23.08	10.98	13.02	7.11	4.74	32.32	0.00	112.74	73.36	73.58	39.09	18.28	11.35	8.27	5.80	43.78
		3	18.76	187.29	99.52	47.77	23.72	10.12	8.54	11.84	10.99	41.35	20.10	178.83	127.38	87.88	55.04	25.28	16.46	7.45	9.80	63.87
		4	54.69	219.63	110.31	46.30	29.07	14.15	7.52	7.75	0.00	47.44	81.17	163.73	121.18	83.24	57.33	28.79	8.45	10.53	0.00	63.57
	Year	16/17	16.00	807.00	908.00	366.00	801.00	201.00	31.00	17.00	3.00	3,150.00	20.00	667.00	1,024.00	745.00	1,726.00	374.00	56.00	26.00	6.00	4,644.00
	Episodes	F/N	20.32	172.42	91.85	42.67	25.02	11.84	8.22	8.23	3.95	39.73	24.84	145.40	107.73	79.48	51.06	23.43	13.18	9.65	4.66	56.66
**Midlands And East**	Quarter	1	0.00	159.16	91.03	46.02	33.49	8.77	3.08	3.38	6.04	34.35	0.00	148.37	114.43	127.34	69.60	23.31	7.51	3.86	0.00	56.01
		2	0.00	135.39	60.99	58.58	28.77	9.52	5.78	3.87	8.42	30.68	0.00	134.20	61.34	123.27	62.31	18.12	14.68	8.71	5.96	47.49
		3	23.24	177.66	83.59	64.84	33.76	10.70	6.76	5.92	2.65	37.79	25.78	196.71	109.82	138.37	72.20	22.19	5.89	6.68	7.54	59.61
		4	68.86	216.08	98.40	73.12	39.06	8.29	4.43	5.16	5.83	43.07	42.86	196.74	120.51	152.74	85.55	29.68	11.96	6.88	6.72	68.37
	Year	16/17	11.00	498.00	647.00	439.00	516.00	170.00	37.00	18.00	8.00	2,344.00	7.00	474.00	746.00	898.00	1,119.00	420.00	78.00	31.00	13.00	3,786.00
	Episodes	F/N	22.59	171.38	83.08	60.60	33.67	9.32	5.03	4.57	5.79	36.36	16.84	168.35	100.77	135.20	72.23	23.23	10.10	6.57	5.07	57.67

Mean weekly incidence rate per 100,000 Persons.

Upper Respiratory Tract Infections (ICD10: J00-J06)

		M										F									
		<1yr	1-4yrs	5-14yrs	15-24yrs	25-44yrs	45-64yrs	65-74yrs	75-84yrs	85+yrs	All Ages	<1yr	1-4yrs	5-14yrs	15-24yrs	25-44yrs	45-64yrs	65-74yrs	75-84yrs	85+yrs	All Ages
**4 weekly**	1	0.00	842.09	220.01	83.93	59.84	46.88	51.56	39.42	37.12	112.06	0.00	733.28	235.44	165.82	151.79	94.97	82.25	59.34	43.58	156.95
	2	0.00	805.27	223.78	88.22	68.54	47.80	42.59	56.06	37.61	115.52	178.57	739.05	237.27	169.08	157.27	93.94	72.44	70.06	61.82	160.05
	3	437.93	767.91	241.40	84.93	72.16	46.25	47.06	35.45	51.70	115.68	233.11	745.64	285.48	170.76	153.02	101.25	74.79	51.88	57.65	166.11
	4	291.81	501.87	115.56	82.12	61.20	48.28	48.10	40.38	59.61	85.56	290.96	442.24	127.74	156.97	116.52	79.29	72.67	51.95	72.77	117.02
	5	523.92	634.27	150.67	86.73	59.96	42.78	44.36	46.52	32.93	95.39	433.93	532.13	160.65	159.27	119.77	85.76	69.82	54.22	37.29	127.90
	6	1106.55	1309.67	256.34	131.50	76.80	69.26	76.88	67.74	50.45	163.94	856.75	1112.06	272.36	257.60	183.38	130.08	111.48	86.82	99.60	216.81
	7	1500.77	1507.72	260.71	115.11	89.58	72.25	78.92	74.77	80.47	180.40	1357.36	1441.15	284.01	236.70	205.45	159.07	132.14	114.94	106.57	248.90
	8	1569.99	1609.32	397.11	133.27	91.26	82.42	97.30	105.25	106.29	212.08	1679.26	1606.83	464.02	245.33	245.18	189.35	151.69	132.28	134.91	301.16
	9	1274.04	1087.08	283.02	133.99	113.65	124.73	131.61	154.17	170.76	196.25	1286.38	1038.05	291.12	252.53	274.09	247.85	207.59	198.25	182.11	289.32
	10	1437.21	1327.71	397.10	163.18	107.24	100.45	87.67	92.73	95.39	211.33	1234.65	1263.53	414.88	295.53	263.18	199.89	164.10	148.09	127.45	295.40
	11	1236.45	1110.46	258.18	128.67	95.18	74.26	80.81	71.36	69.15	166.56	1081.67	1013.92	301.16	213.82	224.87	172.78	129.53	107.09	79.54	237.12
	12	1587.98	1102.38	309.13	100.21	86.02	65.29	67.60	62.31	39.96	165.34	1379.83	998.58	342.31	225.81	212.34	148.99	119.19	89.48	73.49	235.45
	13	1238.43	654.44	164.24	100.07	70.05	56.08	56.32	51.63	61.32	117.47	855.00	586.14	194.22	168.99	167.89	115.88	111.33	71.22	63.14	167.52
**Quarter**	1	134.75	807.93	227.75	85.55	66.31	46.97	47.42	43.32	41.76	114.24	126.67	738.86	251.40	168.34	153.85	96.59	76.94	60.34	53.52	160.73
	2	744.01	880.37	178.77	101.63	69.71	55.77	60.93	55.42	54.25	121.65	646.30	779.61	194.14	196.58	149.56	107.11	91.49	70.56	75.44	165.70
	3	1460.06	1406.23	353.82	139.83	101.45	99.26	101.93	112.82	119.12	206.15	1387.64	1347.69	381.85	260.64	252.04	206.54	169.04	154.99	145.58	290.87
	4	1359.36	982.57	251.22	112.21	85.29	67.08	69.24	63.46	58.24	153.41	1133.52	900.30	286.76	208.44	206.18	148.56	123.05	93.43	73.76	218.87
**Year**	16/17	2066.00	18149.00	11043.00	5178.00	8641.00	6381.00	2421.00	1256.00	449.00	55584.00	1702.00	16107.00	11631.00	9960.00	20377.00	13095.00	4121.00	2091.00	1011.00	80095.00
**Episodes**	F/N	921.14	1016.66	251.49	109.65	80.49	67.05	69.71	68.50	68.08	148.35	820.19	938.56	276.94	208.28	189.64	139.08	114.69	94.37	86.85	208.22

Mean weekly incidence rate per 100,000 Persons.

Upper Respiratory Tract Infections (ICD10: J00-J06)

			M										F									
			<1yr	1-4yrs	5-14yrs	15-24yrs	25-44yrs	45-64yrs	65-74yrs	75-84yrs	85+yrs	All Ages	<1yr	1-4yrs	5-14yrs	15-24yrs	25-44yrs	45-64yrs	65-74yrs	75-84yrs	85+yrs	All Ages
**North**	Quarter	1	290.96	867.94	242.56	85.31	68.48	47.03	48.32	49.20	46.66	114.12	63.05	772.77	271.77	154.07	164.19	95.77	73.72	66.13	52.25	159.67
		2	794.07	984.15	199.61	100.02	72.05	53.16	67.15	58.81	54.84	124.14	756.75	874.12	224.07	198.59	160.79	115.20	95.12	75.68	76.76	175.83
		3	1,256.90	1,345.07	345.97	130.38	96.49	95.54	101.71	115.35	121.86	188.68	1,369.05	1,366.11	383.23	226.68	259.07	203.67	163.80	139.89	124.58	279.04
		4	1,501.04	1,027.51	258.23	99.26	85.40	61.91	72.54	59.09	52.76	146.67	1,189.15	909.50	297.52	185.91	213.63	142.21	115.11	79.49	76.62	210.33
	Year	16/17	603.00	5,464.00	3,441.00	1,788.00	2,617.00	2,072.00	904.00	470.00	154.00	17,513.00	502.00	4,768.00	3,723.00	3,465.00	6,183.00	4,383.00	1,477.00	729.00	335.00	25,565.00
	Episodes	F/N	957.60	1,054.81	260.42	103.67	80.44	64.20	72.33	70.39	68.76	143.04	842.84	978.62	292.83	191.45	198.69	138.76	111.62	90.02	82.44	205.64
**South**	Quarter	1	136.55	740.29	181.38	77.00	63.95	39.29	41.04	30.09	58.21	97.80	315.93	605.59	214.35	165.25	141.41	91.84	64.89	52.48	59.76	141.42
		2	706.39	731.97	129.57	91.14	62.49	43.57	51.13	50.24	45.07	98.68	514.50	644.18	152.98	194.97	129.66	91.33	67.85	64.99	50.41	139.41
		3	1,285.79	1,252.75	291.69	129.51	89.46	81.16	93.96	85.65	92.76	175.31	1,185.01	1,132.47	297.57	238.30	230.52	180.52	141.65	132.67	130.13	249.24
		4	1,140.76	854.20	205.72	101.45	77.85	58.83	61.35	54.54	42.27	130.32	942.78	767.73	242.87	196.30	183.79	123.94	105.68	80.47	54.72	187.05
	Year	16/17	510.00	4,552.00	2,714.00	1,530.00	2,134.00	1,681.00	732.00	355.00	145.00	14,353.00	415.00	3,904.00	2,845.00	3,022.00	4,989.00	3,640.00	1,194.00	646.00	323.00	20,978.00
	Episodes	F/N	815.28	891.73	200.72	99.61	73.23	55.48	61.67	55.04	59.30	125.02	735.31	784.79	225.55	198.63	170.56	121.33	94.50	82.32	73.31	178.53
**London**	Quarter	1	111.48	869.09	254.48	85.87	60.74	53.96	53.51	58.87	26.00	133.94	44.98	845.08	284.37	146.42	137.82	107.47	100.15	74.36	69.54	184.76
		2	722.64	938.67	208.29	106.12	69.96	68.92	71.35	63.88	61.92	144.53	700.47	860.00	223.27	182.16	132.80	118.80	118.76	82.27	91.16	185.81
		3	1,710.40	1,595.48	450.00	149.93	99.51	117.63	116.36	136.67	168.25	254.41	1,455.32	1,440.02	479.47	239.42	228.99	249.66	222.85	222.01	185.40	337.31
		4	1,448.11	1,032.50	297.78	116.04	78.90	78.96	71.99	77.91	78.00	180.08	1,262.91	945.14	311.30	182.00	185.72	172.42	157.21	123.41	84.44	245.05
	Year	16/17	615.00	5,153.00	2,967.00	984.00	2,470.00	1,355.00	293.00	174.00	63.00	14,074.00	509.00	4,668.00	3,062.00	1,760.00	5,775.00	2,579.00	635.00	337.00	139.00	19,464.00
	Episodes	F/N	992.96	1,105.72	300.86	114.33	77.14	79.66	78.17	83.94	83.14	177.61	862.80	1,019.49	322.69	187.40	170.60	161.27	149.16	124.70	107.32	237.24
**Midlands And East**	Quarter	1	0.00	754.41	232.59	94.03	72.06	47.59	46.79	35.13	36.16	111.09	82.71	731.99	235.11	207.63	171.99	91.27	69.00	48.40	32.55	157.05
		2	752.95	866.69	177.60	109.25	74.36	57.43	54.10	48.74	55.18	119.23	613.48	740.15	176.23	210.62	175.00	103.09	84.26	59.32	83.41	161.75
		3	1,587.15	1,431.64	327.61	149.51	120.35	102.70	95.70	113.59	93.60	206.19	1,541.17	1,452.17	367.12	338.17	289.56	192.29	147.87	125.38	142.19	297.89
		4	1,347.52	1,016.07	243.15	132.07	99.03	68.63	71.07	62.29	59.94	156.58	1,139.21	978.84	295.36	269.53	241.57	155.68	114.21	90.36	79.25	233.05
	Year	16/17	338.00	2,980.00	1,921.00	876.00	1,420.00	1,273.00	492.00	257.00	87.00	9,644.00	276.00	2,767.00	2,001.00	1,713.00	3,430.00	2,493.00	815.00	379.00	214.00	14,088.00
	Episodes	F/N	918.72	1,014.36	243.96	120.99	91.13	68.87	66.67	64.63	61.11	147.72	839.79	971.34	266.72	255.62	218.69	134.97	103.46	80.46	84.33	211.48

Mean weekly incidence rate per 100,000 Persons.

Whooping Cough (ICD10: A37)

		M										F									
		<1yr	1-4yrs	5-14yrs	15-24yrs	25-44yrs	45-64yrs	65-74yrs	75-84yrs	85+yrs	All Ages	<1yr	1-4yrs	5-14yrs	15-24yrs	25-44yrs	45-64yrs	65-74yrs	75-84yrs	85+yrs	All Ages
**4 weekly**	1	0.00	0.00	0.00	0.00	0.00	0.35	0.40	0.00	0.00	0.13	0.00	0.00	0.00	0.00	0.19	0.11	0.00	0.00	0.00	0.07
	2	0.00	0.00	0.24	0.00	0.35	0.20	0.00	0.00	0.00	0.17	0.00	0.00	0.51	0.00	0.58	0.00	0.26	0.00	0.00	0.24
	3	0.00	0.00	0.77	0.00	0.00	0.21	0.00	0.60	0.00	0.18	0.00	0.00	0.26	0.00	0.24	0.57	0.95	0.00	0.00	0.35
	4	0.00	0.00	0.43	0.00	0.00	0.00	0.27	0.00	0.00	0.08	0.00	0.79	1.03	0.00	0.22	0.36	0.00	0.74	0.00	0.38
	5	0.00	0.00	0.00	0.45	0.00	0.32	0.44	0.00	0.00	0.19	0.00	0.00	0.00	0.00	0.31	0.11	0.00	1.15	0.00	0.20
	6	0.00	0.00	0.66	0.68	0.21	0.29	0.00	0.84	0.00	0.35	11.08	0.00	0.87	0.42	1.16	0.86	0.40	0.00	0.00	0.74
	7	0.00	1.04	0.00	0.21	0.22	0.29	0.41	0.00	0.00	0.27	0.00	0.00	0.35	0.40	0.11	0.49	0.00	0.00	0.00	0.25
	8	0.00	0.00	0.00	0.18	0.55	0.11	0.00	0.00	0.00	0.21	0.00	0.70	0.64	0.20	0.53	0.32	0.00	0.00	0.00	0.35
	9	0.00	0.00	0.42	0.00	0.00	0.00	0.25	0.00	0.00	0.08	0.00	0.00	0.45	0.20	0.32	0.55	0.00	0.00	0.00	0.32
	10	5.15	0.59	0.23	0.38	0.00	0.10	0.00	0.00	0.00	0.17	0.00	0.61	0.42	0.17	0.11	0.30	0.24	0.00	0.00	0.23
	11	0.00	0.00	0.42	0.21	0.10	0.18	0.00	0.00	0.00	0.16	0.00	0.00	0.00	0.51	0.11	0.10	0.51	0.00	0.00	0.17
	12	0.00	0.61	0.00	0.00	0.00	0.11	0.00	0.00	0.00	0.05	0.00	0.00	0.25	0.00	0.40	0.37	0.42	0.00	0.00	0.29
	13	0.00	0.00	0.46	0.00	0.00	0.28	0.00	0.00	0.00	0.13	0.00	0.59	0.82	0.00	0.52	0.28	0.00	0.00	0.00	0.31
**Quarter**	1	0.00	0.00	0.31	0.00	0.11	0.26	0.15	0.19	0.00	0.16	0.00	0.00	0.24	0.00	0.33	0.22	0.37	0.00	0.00	0.21
	2	0.00	0.30	0.31	0.38	0.09	0.22	0.20	0.24	0.00	0.22	3.17	0.23	0.54	0.23	0.52	0.44	0.11	0.54	0.00	0.42
	3	1.58	0.18	0.20	0.11	0.20	0.10	0.20	0.00	0.00	0.17	0.00	0.40	0.57	0.18	0.26	0.44	0.07	0.00	0.00	0.30
	4	0.00	0.19	0.27	0.12	0.03	0.18	0.00	0.00	0.00	0.11	0.00	0.18	0.33	0.16	0.35	0.23	0.29	0.00	0.00	0.25
**Year**	16/17	1.00	3.00	12.00	8.00	11.00	18.00	5.00	2.00	0.00	60.00	1.00	4.00	17.00	9.00	39.00	33.00	8.00	3.00	0.00	114.00
**Episodes**	F/N	0.39	0.17	0.27	0.16	0.11	0.19	0.14	0.11	0.00	0.17	0.84	0.20	0.42	0.14	0.37	0.34	0.21	0.14	0.00	0.30

Mean weekly incidence rate per 100,000 Persons.

Whooping Cough (ICD10: A37)

			M										F									
			<1yr	1-4yrs	5-14yrs	15-24yrs	25-44yrs	45-64yrs	65-74yrs	75-84yrs	85+yrs	All Ages	<1yr	1-4yrs	5-14yrs	15-24yrs	25-44yrs	45-64yrs	65-74yrs	75-84yrs	85+yrs	All Ages
**North**	Quarter	1	0.00	0.00	1.25	0.00	0.14	0.13	0.00	0.00	0.00	0.20	0.00	0.00	0.32	0.00	0.13	0.27	0.32	0.00	0.00	0.17
		2	0.00	0.00	0.00	0.00	0.12	0.12	0.31	0.00	0.00	0.09	0.00	0.00	0.63	0.42	1.12	0.25	0.00	0.00	0.00	0.47
		3	0.00	0.00	0.00	0.23	0.00	0.12	0.31	0.00	0.00	0.10	0.00	0.86	0.33	0.21	0.53	0.38	0.30	0.00	0.00	0.36
		4	0.00	0.76	0.30	0.00	0.12	0.12	0.00	0.00	0.00	0.13	0.00	0.00	0.62	0.21	0.27	0.38	0.00	0.00	0.00	0.26
	Year	16/17	0.00	1.00	5.00	1.00	3.00	4.00	2.00	0.00	0.00	16.00	0.00	1.00	6.00	4.00	16.00	10.00	2.00	0.00	0.00	39.00
	Episodes	F/N	0.00	0.19	0.38	0.06	0.09	0.12	0.16	0.00	0.00	0.13	0.00	0.21	0.48	0.21	0.52	0.32	0.15	0.00	0.00	0.32
**South**	Quarter	1	0.00	0.00	0.00	0.00	0.00	0.17	0.00	0.74	0.00	0.09	0.00	0.00	0.00	0.00	0.17	0.35	0.00	0.00	0.00	0.13
		2	0.00	0.00	0.27	0.50	0.26	0.37	0.00	0.00	0.00	0.26	12.66	0.00	0.61	0.51	0.27	0.40	0.00	0.51	0.00	0.37
		3	0.00	0.73	0.28	0.23	0.31	0.27	0.00	0.00	0.00	0.24	0.00	0.75	0.00	0.49	0.26	0.77	0.00	0.00	0.00	0.36
		4	0.00	0.00	0.27	0.50	0.00	0.14	0.00	0.00	0.00	0.13	0.00	0.72	0.29	0.00	0.75	0.12	0.63	0.00	0.00	0.35
	Year	16/17	0.00	1.00	3.00	5.00	4.00	7.00	0.00	1.00	0.00	21.00	1.00	2.00	3.00	4.00	11.00	12.00	2.00	1.00	0.00	36.00
	Episodes	F/N	0.00	0.18	0.21	0.31	0.14	0.24	0.00	0.18	0.00	0.18	3.35	0.36	0.23	0.26	0.36	0.41	0.15	0.13	0.00	0.30
**London**	Quarter	1	0.00	0.00	0.00	0.00	0.00	0.25	0.00	0.00	0.00	0.05	0.00	0.00	0.00	0.00	0.12	0.00	0.00	0.00	0.00	0.05
		2	0.00	0.00	0.00	0.00	0.00	0.00	0.00	0.00	0.00	0.00	0.00	0.90	0.39	0.00	0.23	0.23	0.00	0.00	0.00	0.24
		3	6.34	0.00	0.00	0.00	0.26	0.00	0.00	0.00	0.00	0.16	0.00	0.00	0.87	0.00	0.24	0.00	0.00	0.00	0.00	0.20
		4	0.00	0.00	0.00	0.00	0.00	0.00	0.00	0.00	0.00	0.00	0.00	0.00	0.41	0.42	0.12	0.00	0.00	0.00	0.00	0.14
	Year	16/17	1.00	0.00	0.00	0.00	2.00	1.00	0.00	0.00	0.00	4.00	0.00	1.00	4.00	1.00	6.00	1.00	0.00	0.00	0.00	13.00
	Episodes	F/N	1.55	0.00	0.00	0.00	0.06	0.06	0.00	0.00	0.00	0.05	0.00	0.24	0.42	0.10	0.18	0.06	0.00	0.00	0.00	0.16
**Midlands And East**	Quarter	1	0.00	0.00	0.00	0.00	0.30	0.50	0.62	0.00	0.00	0.28	0.00	0.00	0.62	0.00	0.89	0.25	1.16	0.00	0.00	0.49
		2	0.00	1.19	0.98	1.04	0.00	0.41	0.51	0.96	0.00	0.52	0.00	0.00	0.54	0.00	0.46	0.89	0.46	1.65	0.00	0.59
		3	0.00	0.00	0.52	0.00	0.24	0.00	0.51	0.00	0.00	0.18	0.00	0.00	1.07	0.00	0.00	0.62	0.00	0.00	0.00	0.29
		4	0.00	0.00	0.52	0.00	0.00	0.45	0.00	0.00	0.00	0.19	0.00	0.00	0.00	0.00	0.25	0.43	0.51	0.00	0.00	0.24
	Year	16/17	0.00	1.00	4.00	2.00	2.00	6.00	3.00	1.00	0.00	19.00	0.00	0.00	4.00	0.00	6.00	10.00	4.00	2.00	0.00	26.00
	Episodes	F/N	0.00	0.31	0.51	0.27	0.13	0.34	0.41	0.25	0.00	0.30	0.00	0.00	0.56	0.00	0.40	0.55	0.53	0.44	0.00	0.41

Mean weekly incidence rate per 100,000 Persons.

Infectious Mononucleosis (ICD10: B27)

		M										F									
		<1yr	1-4yrs	5-14yrs	15-24yrs	25-44yrs	45-64yrs	65-74yrs	75-84yrs	85+yrs	All Ages	<1yr	1-4yrs	5-14yrs	15-24yrs	25-44yrs	45-64yrs	65-74yrs	75-84yrs	85+yrs	All Ages
**4 weekly**	1	0.00	0.00	0.00	1.94	0.42	0.00	0.00	0.00	0.00	0.35	0.00	0.00	0.48	2.47	0.48	0.00	0.00	0.00	0.00	0.47
	2	0.00	0.84	0.29	0.94	0.73	0.14	0.00	0.00	0.00	0.42	0.00	0.00	0.26	1.02	0.46	0.00	0.00	0.00	0.00	0.26
	3	0.00	0.00	0.56	1.58	0.00	0.20	0.00	0.00	0.00	0.33	0.00	0.00	0.25	2.48	0.37	0.00	0.00	0.00	0.00	0.42
	4	0.00	0.00	0.00	2.57	1.00	0.13	0.00	0.00	0.00	0.63	0.00	0.00	0.28	3.14	0.23	0.00	0.00	0.00	0.00	0.46
	5	0.00	0.00	0.24	1.87	0.31	0.00	0.00	0.00	0.00	0.35	0.00	0.00	0.34	4.22	0.87	0.18	0.00	0.00	0.00	0.82
	6	0.00	0.00	0.26	1.99	0.00	0.28	0.00	0.00	0.00	0.37	0.00	0.00	0.25	2.64	0.19	0.00	0.00	0.00	0.00	0.41
	7	0.00	0.70	0.34	1.72	0.10	0.00	0.00	0.00	0.00	0.32	0.00	0.66	0.00	3.20	0.00	0.00	0.00	0.00	0.00	0.42
	8	0.00	1.04	0.22	2.03	0.21	0.00	0.00	0.00	0.00	0.40	0.00	0.65	0.94	3.27	0.80	0.11	0.26	0.00	0.00	0.77
	9	0.00	0.00	0.24	0.98	0.32	0.11	0.00	0.00	0.00	0.27	0.00	0.00	0.00	1.73	0.53	0.00	0.00	0.00	0.00	0.35
	10	0.00	0.00	0.83	2.30	0.10	0.00	0.00	0.00	0.00	0.43	0.00	0.00	0.00	3.52	0.20	0.17	0.00	0.00	0.00	0.59
	11	0.00	0.56	0.00	1.76	0.51	0.17	0.00	0.00	0.00	0.44	0.00	0.00	0.25	2.97	0.32	0.11	0.00	0.00	0.00	0.50
	12	0.00	0.00	0.25	1.86	0.62	0.00	0.00	0.00	0.00	0.40	0.00	0.00	0.76	1.76	0.50	0.09	0.00	0.00	0.00	0.48
	13	0.00	0.00	0.22	3.03	0.61	0.00	0.00	0.00	0.00	0.57	0.00	0.00	0.65	2.15	0.10	0.00	0.00	0.00	0.00	0.38
**Quarter**	1	0.00	0.26	0.26	1.52	0.39	0.10	0.00	0.00	0.00	0.36	0.00	0.00	0.34	2.03	0.44	0.00	0.00	0.00	0.00	0.39
	2	0.00	0.20	0.24	1.94	0.37	0.12	0.00	0.00	0.00	0.42	0.00	0.00	0.25	3.23	0.37	0.05	0.00	0.00	0.00	0.53
	3	0.00	0.32	0.27	1.87	0.19	0.03	0.00	0.00	0.00	0.35	0.00	0.40	0.29	2.97	0.44	0.09	0.08	0.00	0.00	0.56
	4	0.00	0.17	0.27	2.23	0.57	0.05	0.00	0.00	0.00	0.48	0.00	0.00	0.51	2.34	0.32	0.06	0.00	0.00	0.00	0.46
**Year**	16/17	0.00	4.00	12.00	93.00	38.00	7.00	0.00	0.00	0.00	154.00	0.00	2.00	16.00	132.00	39.00	5.00	1.00	0.00	0.00	195.00
**Episodes**	F/N	0.00	0.24	0.26	1.89	0.38	0.08	0.00	0.00	0.00	0.40	0.00	0.10	0.35	2.65	0.39	0.05	0.02	0.00	0.00	0.49

Mean weekly incidence rate per 100,000 Persons.

Infectious Mononucleosis (ICD10: B27)

			M										F									
			<1yr	1-4yrs	5-14yrs	15-24yrs	25-44yrs	45-64yrs	65-74yrs	75-84yrs	85+yrs	All Ages	<1yr	1-4yrs	5-14yrs	15-24yrs	25-44yrs	45-64yrs	65-74yrs	75-84yrs	85+yrs	All Ages
**North**	Quarter	1	0.00	0.00	0.32	1.54	0.13	0.00	0.00	0.00	0.00	0.28	0.00	0.00	0.95	1.40	0.41	0.00	0.00	0.00	0.00	0.40
		2	0.00	0.00	0.30	2.69	0.23	0.12	0.00	0.00	0.00	0.50	0.00	0.00	0.00	2.78	0.24	0.00	0.00	0.00	0.00	0.47
		3	0.00	0.00	0.00	2.50	0.12	0.00	0.00	0.00	0.00	0.39	0.00	0.81	0.66	4.27	0.39	0.00	0.00	0.00	0.00	0.84
		4	0.00	0.00	0.31	1.15	0.62	0.00	0.00	0.00	0.00	0.36	0.00	0.00	0.63	3.12	0.00	0.00	0.00	0.00	0.00	0.52
	Year	16/17	0.00	0.00	3.00	34.00	9.00	1.00	0.00	0.00	0.00	47.00	0.00	1.00	7.00	53.00	8.00	0.00	0.00	0.00	0.00	69.00
	Episodes	F/N	0.00	0.00	0.23	1.98	0.27	0.03	0.00	0.00	0.00	0.39	0.00	0.20	0.55	2.89	0.26	0.00	0.00	0.00	0.00	0.55
**South**	Quarter	1	0.00	1.04	0.73	1.00	0.85	0.17	0.00	0.00	0.00	0.53	0.00	0.00	0.42	2.32	0.34	0.00	0.00	0.00	0.00	0.43
		2	0.00	0.00	0.27	2.61	0.27	0.15	0.00	0.00	0.00	0.48	0.00	0.00	0.61	3.36	0.67	0.00	0.00	0.00	0.00	0.66
		3	0.00	0.00	0.56	2.52	0.26	0.14	0.00	0.00	0.00	0.51	0.00	0.79	0.00	1.75	0.27	0.13	0.31	0.00	0.00	0.40
		4	0.00	0.69	0.27	2.44	0.51	0.00	0.00	0.00	0.00	0.52	0.00	0.00	0.90	3.60	0.78	0.24	0.00	0.00	0.00	0.82
	Year	16/17	0.00	2.00	6.00	33.00	13.00	3.00	0.00	0.00	0.00	57.00	0.00	1.00	6.00	42.00	15.00	3.00	1.00	0.00	0.00	68.00
	Episodes	F/N	0.00	0.42	0.46	2.15	0.47	0.11	0.00	0.00	0.00	0.51	0.00	0.19	0.49	2.77	0.52	0.09	0.08	0.00	0.00	0.58
**London**	Quarter	1	0.00	0.00	0.00	1.00	0.26	0.00	0.00	0.00	0.00	0.21	0.00	0.00	0.00	2.33	0.12	0.00	0.00	0.00	0.00	0.31
		2	0.00	0.80	0.39	0.86	0.25	0.00	0.00	0.00	0.00	0.29	0.00	0.00	0.39	1.65	0.34	0.00	0.00	0.00	0.00	0.37
		3	0.00	0.00	0.00	1.41	0.13	0.00	0.00	0.00	0.00	0.20	0.00	0.00	0.00	1.27	0.12	0.00	0.00	0.00	0.00	0.20
		4	0.00	0.00	0.00	0.88	0.37	0.00	0.00	0.00	0.00	0.25	0.00	0.00	0.00	0.83	0.23	0.00	0.00	0.00	0.00	0.19
	Year	16/17	0.00	1.00	1.00	9.00	8.00	0.00	0.00	0.00	0.00	19.00	0.00	0.00	1.00	14.00	7.00	0.00	0.00	0.00	0.00	22.00
	Episodes	F/N	0.00	0.21	0.10	1.03	0.25	0.00	0.00	0.00	0.00	0.24	0.00	0.00	0.10	1.52	0.21	0.00	0.00	0.00	0.00	0.27
**Midlands And East**	Quarter	1	0.00	0.00	0.00	2.54	0.30	0.25	0.00	0.00	0.00	0.43	0.00	0.00	0.00	2.05	0.89	0.00	0.00	0.00	0.00	0.42
		2	0.00	0.00	0.00	1.62	0.75	0.20	0.00	0.00	0.00	0.42	0.00	0.00	0.00	5.14	0.24	0.21	0.00	0.00	0.00	0.64
		3	0.00	1.28	0.51	1.05	0.26	0.00	0.00	0.00	0.00	0.30	0.00	0.00	0.49	4.59	0.98	0.21	0.00	0.00	0.00	0.81
		4	0.00	0.00	0.52	4.44	0.77	0.21	0.00	0.00	0.00	0.80	0.00	0.00	0.51	1.82	0.26	0.00	0.00	0.00	0.00	0.30
	Year	16/17	0.00	1.00	2.00	17.00	8.00	3.00	0.00	0.00	0.00	31.00	0.00	0.00	2.00	23.00	9.00	2.00	0.00	0.00	0.00	36.00
	Episodes	F/N	0.00	0.31	0.25	2.40	0.53	0.16	0.00	0.00	0.00	0.48	0.00	0.00	0.25	3.43	0.58	0.11	0.00	0.00	0.00	0.54

Mean weekly incidence rate per 100,000 Persons.

Lower Respiratory Tract Infections (ICD10: J20-J22)

		M										F									
		<1yr	1-4yrs	5-14yrs	15-24yrs	25-44yrs	45-64yrs	65-74yrs	75-84yrs	85+yrs	All Ages	<1yr	1-4yrs	5-14yrs	15-24yrs	25-44yrs	45-64yrs	65-74yrs	75-84yrs	85+yrs	All Ages
**4 weekly**	1	0.00	115.53	22.79	12.21	21.44	35.07	89.73	127.94	195.74	43.20	0.00	90.98	18.74	17.78	30.77	49.19	90.14	124.66	208.82	51.86
	2	0.00	93.47	24.17	15.68	20.79	36.76	72.70	127.23	237.14	42.47	0.00	83.93	19.86	21.14	29.83	55.70	91.50	124.55	209.34	53.68
	3	65.79	111.16	27.61	19.68	24.42	35.23	87.03	116.51	207.24	45.81	28.67	89.04	26.44	19.95	40.31	64.23	95.81	130.97	196.09	60.08
	4	0.00	69.13	13.70	9.50	20.58	34.81	68.73	121.89	186.85	37.13	0.00	53.80	13.73	16.06	28.89	39.66	60.13	106.49	173.71	41.47
	5	67.91	91.33	17.29	16.86	21.17	37.18	66.79	117.02	194.29	39.63	26.79	58.83	12.78	22.46	32.80	57.68	70.54	102.07	180.86	48.92
	6	256.98	221.49	33.26	24.12	32.66	49.01	111.44	152.00	236.57	62.23	155.99	149.94	24.35	42.66	55.84	80.74	108.69	170.80	320.37	80.51
	7	547.20	347.64	38.39	20.82	33.50	64.27	127.17	192.72	340.95	79.09	460.05	285.72	31.65	34.55	59.67	93.31	140.16	212.39	364.24	98.08
	8	890.83	406.39	59.23	24.87	33.87	71.75	149.09	240.17	376.77	93.56	717.44	354.17	48.52	35.25	64.35	131.56	175.54	248.83	413.71	123.31
	9	564.65	202.53	41.29	34.70	45.28	112.73	224.07	349.10	616.83	113.24	369.37	168.08	35.89	39.61	75.20	170.11	263.46	389.76	661.24	148.67
	10	342.17	199.98	35.72	26.90	40.92	94.51	156.09	299.32	528.59	92.86	249.75	143.47	36.79	33.84	62.85	120.65	172.42	273.95	485.90	110.51
	11	292.09	147.83	24.27	18.38	32.28	62.75	123.69	197.18	295.12	66.04	181.35	99.53	23.36	23.25	49.58	93.48	146.64	198.03	304.71	82.20
	12	287.10	113.02	29.44	18.94	28.00	60.21	114.73	189.26	309.49	61.83	203.03	115.08	26.22	23.80	47.49	72.96	116.49	186.29	269.23	73.81
	13	188.60	91.83	16.74	14.55	24.34	49.60	94.81	172.46	280.36	50.99	113.58	50.59	15.37	25.85	38.09	70.83	115.66	153.42	252.54	63.24
**Quarter**	1	20.24	107.40	24.70	15.58	22.16	35.64	83.66	124.21	212.02	43.78	8.82	88.21	21.45	19.48	33.42	55.82	92.31	126.57	205.07	54.94
	2	163.54	148.94	23.14	17.57	25.87	43.24	86.02	138.79	219.61	49.89	99.72	107.36	18.84	28.53	41.69	63.65	88.59	136.55	240.49	61.83
	3	620.19	298.46	46.25	28.18	39.99	89.82	176.42	286.70	495.69	99.70	485.18	247.38	40.22	36.08	68.25	137.46	198.66	295.26	514.80	126.86
	4	261.40	123.04	24.01	17.40	28.33	59.55	111.44	188.79	303.43	60.92	169.69	92.14	22.16	24.69	45.23	80.45	127.39	186.00	280.52	74.46
**Year**	16/17	572.00	2966.00	1255.00	898.00	3039.00	5544.00	4016.00	3437.00	2118.00	23845.00	383.00	2256.00	1047.00	1294.00	4997.00	8002.00	4690.00	4206.00	3829.00	30704.00
**Episodes**	F/N	264.40	169.07	29.40	19.64	29.03	56.80	113.85	183.76	306.03	63.31	189.13	133.28	25.54	27.22	47.04	83.95	126.02	185.16	308.90	79.19

Mean weekly incidence rate per 100,000 Persons.

Lower Respiratory Tract Infections (ICD10: J20-J22)

			M										F									
			<1yr	1-4yrs	5-14yrs	15-24yrs	25-44yrs	45-64yrs	65-74yrs	75-84yrs	85+yrs	All Ages	<1yr	1-4yrs	5-14yrs	15-24yrs	25-44yrs	45-64yrs	65-74yrs	75-84yrs	85+yrs	All Ages
**North**	Quarter	1	0.00	115.82	21.61	14.32	23.22	42.33	87.78	130.64	242.03	47.95	35.29	78.61	19.06	21.13	42.39	65.08	92.39	123.36	196.38	60.61
		2	269.54	197.55	25.95	16.55	29.59	51.12	95.66	121.46	238.15	56.60	180.67	147.07	19.47	25.09	48.09	75.67	99.01	157.05	256.80	72.51
		3	651.94	303.52	35.93	25.46	40.67	96.81	182.92	290.06	486.59	102.69	571.51	246.45	39.18	33.26	74.34	144.22	204.08	293.94	518.68	133.23
		4	270.63	111.43	19.32	14.78	29.15	62.88	108.18	184.23	328.41	61.72	146.57	98.02	20.21	20.64	51.33	86.22	135.39	199.29	309.00	81.49
	Year	16/17	183.00	945.00	338.00	307.00	999.00	2,039.00	1,480.00	1,207.00	721.00	8,219.00	126.00	694.00	310.00	453.00	1,678.00	2,919.00	1,749.00	1,559.00	1,296.00	10,784.00
	Episodes	F/N	297.49	182.37	25.71	17.76	30.64	63.05	118.20	180.46	322.18	67.04	232.51	142.62	24.39	25.03	53.93	92.48	132.08	192.72	319.02	86.69
**South**	Quarter	1	0.00	111.82	18.94	11.02	20.48	29.03	68.84	105.13	206.82	39.19	0.00	96.91	20.64	17.09	30.25	49.70	68.05	100.34	212.50	51.10
		2	147.50	131.75	17.01	16.26	18.95	34.51	65.50	115.17	207.55	42.33	96.43	89.59	12.93	28.13	36.33	49.62	69.35	101.55	207.60	52.99
		3	640.58	266.08	33.08	19.22	34.55	70.84	149.76	252.18	465.02	88.28	466.05	238.40	28.29	31.61	63.33	110.01	171.68	243.00	446.64	114.07
		4	272.35	91.36	20.37	11.94	25.87	47.16	94.20	156.32	236.46	52.30	151.79	72.17	17.03	19.32	41.18	61.52	97.28	155.97	272.30	64.95
	Year	16/17	163.00	762.00	302.00	226.00	728.00	1,378.00	1,118.00	1,006.00	688.00	6,371.00	103.00	609.00	245.00	369.00	1,252.00	2,029.00	1,276.00	1,170.00	1,256.00	8,309.00
	Episodes	F/N	262.89	149.90	22.25	14.64	24.85	45.18	94.03	156.41	277.61	55.28	177.02	123.61	19.59	24.12	42.65	67.37	100.98	149.30	283.30	70.45
**London**	Quarter	1	0.00	94.34	24.74	19.35	16.29	33.39	76.52	113.43	166.50	33.14	0.00	73.15	22.02	15.03	24.00	50.58	109.07	143.17	160.63	41.64
		2	100.95	97.29	21.15	20.43	21.50	41.72	82.70	141.57	171.89	38.29	90.80	73.54	19.78	26.39	28.14	65.14	86.64	144.10	215.15	47.18
		3	508.16	261.83	56.71	29.23	30.80	87.04	165.44	276.73	511.26	80.16	426.44	211.56	45.57	32.43	52.90	136.24	192.96	319.49	475.64	99.42
		4	209.74	105.49	27.56	17.39	20.73	55.88	94.65	210.86	193.98	45.35	135.86	82.09	21.61	28.24	33.17	80.87	136.84	189.26	197.48	56.87
	Year	16/17	125.00	647.00	319.00	185.00	714.00	924.00	392.00	383.00	197.00	3,886.00	87.00	499.00	257.00	242.00	1,165.00	1,325.00	555.00	536.00	341.00	5,007.00
	Episodes	F/N	202.76	138.94	32.32	21.58	22.31	54.26	104.41	184.81	259.23	49.03	161.91	109.40	27.11	25.54	34.43	82.87	130.53	197.97	261.34	61.01
**Midlands And East**	Quarter	1	80.97	107.60	33.50	17.63	28.65	37.82	101.49	147.62	232.71	54.84	0.00	104.17	24.08	24.67	37.02	57.91	99.72	139.40	250.75	66.42
		2	136.15	169.18	28.45	17.03	33.45	45.61	100.23	176.97	260.86	62.34	30.99	119.24	23.17	34.51	54.22	64.15	99.36	143.48	282.41	74.65
		3	680.06	362.40	59.26	38.80	53.96	104.61	207.57	327.82	519.92	127.66	476.72	293.10	47.84	47.00	82.40	159.35	225.91	324.62	618.23	160.73
		4	292.89	183.89	28.79	25.51	37.56	72.30	148.71	203.76	454.88	84.29	244.53	116.30	29.78	30.54	55.26	93.20	140.06	199.50	343.32	94.53
	Year	16/17	101.00	612.00	296.00	180.00	598.00	1,203.00	1,026.00	841.00	512.00	5,369.00	67.00	454.00	235.00	230.00	902.00	1,729.00	1,110.00	941.00	936.00	6,604.00
	Episodes	F/N	294.47	205.07	37.33	24.60	38.31	64.72	138.76	213.34	365.09	81.91	185.10	157.47	31.06	34.19	57.17	93.10	140.47	200.65	371.95	98.62

Mean weekly incidence rate per 100,000 Persons.

Otitis Media (ICD10: H650-H651,H660,H669)

		M										F									
		<1yr	1-4yrs	5-14yrs	15-24yrs	25-44yrs	45-64yrs	65-74yrs	75-84yrs	85+yrs	All Ages	<1yr	1-4yrs	5-14yrs	15-24yrs	25-44yrs	45-64yrs	65-74yrs	75-84yrs	85+yrs	All Ages
**4 weekly**	1	0.00	155.81	44.47	9.20	6.65	5.06	4.60	2.77	0.00	17.10	0.00	140.31	49.20	13.75	9.97	8.55	6.20	4.98	4.65	18.95
	2	0.00	147.74	44.31	9.31	6.50	6.77	5.52	5.85	0.00	17.67	0.00	156.70	41.36	14.05	14.73	10.74	3.17	6.11	7.38	20.75
	3	0.00	138.38	56.96	6.82	8.01	6.70	4.58	5.76	5.72	18.79	0.00	158.25	54.80	9.42	11.30	8.34	6.21	3.11	2.50	20.06
	4	0.00	94.05	28.29	12.17	5.57	7.66	5.97	5.11	3.80	13.53	0.00	80.55	32.68	13.95	11.23	9.60	9.01	4.86	11.04	15.59
	5	12.23	115.15	36.15	8.74	7.39	6.00	7.05	3.55	6.31	15.33	0.00	83.15	32.01	10.28	12.81	10.13	8.19	6.47	3.18	15.73
	6	14.27	210.45	43.78	9.82	7.86	7.51	5.80	8.31	6.54	21.31	21.78	155.66	49.27	18.50	14.95	10.39	6.54	2.69	1.29	22.28
	7	20.08	259.26	54.55	7.88	8.45	5.62	4.58	6.19	12.12	24.39	46.59	246.51	53.95	15.58	12.74	10.82	7.02	5.14	3.65	26.02
	8	49.19	317.15	93.86	8.61	7.68	6.01	8.79	4.84	6.54	32.18	69.22	288.48	102.87	21.97	17.96	13.49	6.10	6.87	3.75	36.17
	9	35.54	227.89	53.36	11.10	10.54	7.63	6.84	5.07	3.58	24.55	60.60	188.33	59.32	20.55	20.23	15.71	9.38	4.13	3.87	28.22
	10	118.83	296.72	66.09	11.12	9.27	8.01	6.72	5.23	5.98	29.65	61.55	276.73	66.64	22.30	17.10	14.34	10.70	8.49	3.44	32.39
	11	145.45	241.70	51.46	12.83	9.01	6.81	8.29	6.96	4.53	25.12	79.16	250.50	54.02	14.66	17.18	11.06	6.88	3.72	0.84	27.87
	12	170.63	235.89	59.24	8.16	7.26	7.06	9.35	1.46	4.49	25.14	93.06	207.24	64.57	19.37	16.21	11.05	7.76	6.63	6.54	27.71
	13	144.08	145.17	34.20	11.05	6.98	5.67	3.97	4.46	2.44	17.38	120.12	137.18	30.28	10.90	11.83	11.02	10.30	2.32	5.01	18.90
**Quarter**	1	0.00	147.96	48.26	8.50	7.02	6.09	4.88	4.64	1.76	17.79	0.00	150.88	48.51	12.51	11.84	9.16	5.27	4.75	4.83	19.84
	2	13.31	148.64	37.78	9.76	7.23	6.98	6.01	5.95	7.80	17.37	15.02	123.07	39.83	14.24	12.87	10.29	7.66	4.93	4.78	18.79
	3	58.78	284.71	70.19	10.59	8.93	6.93	7.00	5.23	5.40	28.68	57.86	248.10	73.19	21.02	17.52	13.90	8.51	5.76	4.16	31.22
	4	145.44	214.73	49.42	10.12	7.90	6.47	7.25	4.11	3.52	22.95	95.83	208.40	51.80	15.35	15.46	11.21	8.44	4.72	3.81	25.74
**Year**	16/17	150.00	3530.00	2254.00	458.00	814.00	626.00	205.00	81.00	23.00	8141.00	110.00	3063.00	2252.00	741.00	1530.00	1028.00	278.00	117.00	50.00	9169.00
**Episodes**	F/N	53.61	198.06	51.16	9.74	7.76	6.62	6.28	5.00	4.68	21.62	41.67	181.48	53.08	15.75	14.39	11.12	7.47	5.04	4.40	23.80

Mean weekly incidence rate per 100,000 Persons.

Otitis Media (ICD10: H650-H651,H660,H669)

			M										F									
			<1yr	1-4yrs	5-14yrs	15-24yrs	25-44yrs	45-64yrs	65-74yrs	75-84yrs	85+yrs	All Ages	<1yr	1-4yrs	5-14yrs	15-24yrs	25-44yrs	45-64yrs	65-74yrs	75-84yrs	85+yrs	All Ages
**North**	Quarter	1	0.00	161.45	47.18	9.62	7.85	6.37	3.94	4.11	0.00	17.67	0.00	156.23	54.32	14.88	13.90	8.35	5.83	5.95	4.89	20.58
		2	0.00	174.90	44.92	8.95	6.92	7.24	9.41	3.47	3.37	18.53	21.69	142.57	48.16	14.53	16.29	10.32	8.57	5.73	6.42	20.96
		3	16.94	301.10	65.95	12.99	8.47	6.85	4.59	4.86	3.60	26.66	80.19	279.76	82.30	18.08	18.47	13.98	10.63	5.45	4.10	32.19
		4	174.59	216.03	51.54	7.36	8.79	6.23	5.73	2.41	0.00	21.75	83.61	217.53	53.46	11.16	15.36	9.91	10.29	4.99	5.14	24.19
	Year	16/17	43.00	1,103.00	690.00	168.00	260.00	215.00	74.00	25.00	4.00	2,582.00	32.00	966.00	754.00	263.00	497.00	336.00	117.00	45.00	21.00	3,031.00
	Episodes	F/N	46.98	212.65	52.26	9.72	7.99	6.68	5.98	3.71	1.77	21.10	45.91	197.96	59.34	14.66	16.01	10.63	8.82	5.53	5.16	24.42
**South**	Quarter	1	0.00	158.42	43.72	7.10	7.42	5.76	2.95	2.99	1.99	16.86	0.00	135.51	50.64	11.54	10.54	7.23	4.04	6.29	5.46	18.08
		2	22.58	155.41	31.65	7.74	6.80	5.36	5.38	5.43	3.23	15.83	22.91	125.57	36.83	14.69	10.95	9.17	4.35	4.51	0.89	17.12
		3	58.64	295.97	78.68	10.03	7.48	5.57	5.58	2.27	1.68	28.44	62.90	245.70	68.26	15.96	15.27	10.94	8.20	5.15	1.72	28.10
		4	172.90	240.43	49.24	9.35	6.19	4.33	3.21	1.16	0.00	22.35	92.44	195.10	58.65	12.28	12.15	9.48	6.88	2.43	0.00	23.32
	Year	16/17	49.00	1,090.00	682.00	131.00	202.00	155.00	50.00	19.00	4.00	2,382.00	32.00	874.00	669.00	207.00	356.00	273.00	74.00	34.00	8.00	2,527.00
	Episodes	F/N	62.76	211.48	50.46	8.54	6.97	5.26	4.30	3.01	1.75	20.78	44.15	174.53	53.28	13.64	12.20	9.20	5.84	4.59	1.99	21.57
**London**	Quarter	1	0.00	110.97	36.93	6.55	4.37	4.98	5.84	8.07	5.05	15.37	0.00	103.76	37.15	8.52	9.32	11.51	7.73	0.00	8.96	17.86
		2	0.00	91.33	33.03	9.21	6.66	7.88	4.94	7.27	14.13	15.42	0.00	85.52	30.34	14.88	10.37	11.68	9.62	5.45	5.86	17.33
		3	56.71	229.89	55.04	6.51	7.77	6.67	7.75	7.97	10.78	26.75	61.23	171.19	67.05	18.89	13.90	16.90	8.72	7.49	3.26	29.64
		4	89.57	160.41	40.88	9.40	5.91	7.94	11.77	9.79	11.10	21.41	109.44	149.65	35.38	13.80	14.37	14.10	9.38	4.41	0.00	24.40
	Year	16/17	28.00	688.00	407.00	68.00	198.00	117.00	28.00	17.00	8.00	1,559.00	32.00	582.00	399.00	132.00	405.00	215.00	38.00	12.00	6.00	1,821.00
	Episodes	F/N	35.88	147.08	41.31	7.94	6.19	6.89	7.52	8.26	10.34	19.66	41.86	126.74	42.25	14.04	11.96	13.51	8.88	4.36	4.54	22.21
**Midlands And East**	Quarter	1	0.00	161.01	65.21	10.73	8.45	7.25	6.78	3.39	0.00	21.28	0.00	208.01	51.93	15.09	13.61	9.55	3.49	6.77	0.00	22.86
		2	30.65	172.92	41.53	13.13	8.55	7.44	4.32	7.64	10.49	19.71	15.49	138.61	43.98	12.88	13.88	9.99	8.08	4.03	5.94	19.76
		3	102.82	311.89	81.10	12.82	12.02	8.63	10.08	5.82	5.55	32.86	27.13	295.73	75.15	31.16	22.45	13.79	6.48	4.97	7.56	34.93
		4	144.72	242.05	56.02	14.38	10.70	7.36	8.29	3.08	3.00	26.29	97.83	271.30	59.72	24.16	19.94	11.34	7.20	7.04	10.11	31.05
	Year	16/17	30.00	649.00	475.00	91.00	154.00	139.00	53.00	20.00	7.00	1,618.00	14.00	641.00	430.00	139.00	272.00	204.00	49.00	26.00	15.00	1,790.00
	Episodes	F/N	68.81	221.04	60.60	12.77	9.90	7.67	7.31	5.03	4.87	24.93	34.74	226.72	57.44	20.67	17.40	11.15	6.34	5.67	5.90	27.01

4. Vaccine Sensitive Disorders:

Mean weekly incidence rate per 100,000 Persons.

Measles (ICD10: B05)

		M										F									
		<1yr	1-4yrs	5-14yrs	15-24yrs	25-44yrs	45-64yrs	65-74yrs	75-84yrs	85+yrs	All Ages	<1yr	1-4yrs	5-14yrs	15-24yrs	25-44yrs	45-64yrs	65-74yrs	75-84yrs	85+yrs	All Ages
**4 weekly**	1	0.00	0.69	0.00	0.00	0.00	0.00	0.00	0.00	0.00	0.03	0.00	0.00	0.00	0.00	0.00	0.00	0.00	0.00	0.00	0.00
	2	0.00	0.72	0.00	0.00	0.00	0.00	0.00	0.00	0.00	0.04	0.00	0.00	0.00	0.00	0.00	0.00	0.00	0.00	0.00	0.00
	3	0.00	0.00	0.00	0.00	0.00	0.00	0.00	0.00	0.00	0.00	0.00	0.00	0.00	0.00	0.25	0.00	0.00	0.00	0.00	0.08
	4	0.00	1.47	0.00	0.00	0.00	0.00	0.00	0.00	0.00	0.06	0.00	0.00	0.00	0.00	0.00	0.00	0.00	0.00	0.00	0.00
	5	0.00	0.00	0.00	0.00	0.11	0.00	0.00	0.00	0.00	0.04	0.00	0.00	0.00	0.00	0.00	0.00	0.00	0.00	0.00	0.00
	6	0.00	1.41	0.00	0.00	0.00	0.00	0.00	0.00	0.00	0.08	0.00	0.00	0.00	0.00	0.00	0.00	0.00	0.00	0.00	0.00
	7	0.00	0.70	0.00	0.00	0.11	0.00	0.00	0.00	0.00	0.07	0.00	0.00	0.00	0.00	0.00	0.00	0.00	0.00	0.00	0.00
	8	0.00	0.00	0.00	0.00	0.00	0.00	0.00	0.00	0.00	0.00	0.00	0.75	0.00	0.00	0.00	0.00	0.00	0.00	0.00	0.04
	9	0.00	0.00	0.00	0.00	0.00	0.00	0.00	0.00	0.00	0.00	0.00	0.00	0.00	0.00	0.00	0.00	0.00	0.00	0.00	0.00
	10	0.00	0.00	0.00	0.00	0.00	0.00	0.00	0.00	0.00	0.00	0.00	0.00	0.35	0.00	0.00	0.00	0.00	0.00	0.00	0.04
	11	0.00	0.00	0.00	0.00	0.00	0.00	0.00	0.00	0.00	0.00	0.00	0.69	0.00	0.00	0.00	0.00	0.00	0.00	0.00	0.04
	12	0.00	0.00	0.43	0.00	0.00	0.00	0.00	0.00	0.00	0.05	0.00	0.00	0.00	0.00	0.00	0.00	0.00	0.00	0.00	0.00
	13	0.00	0.68	0.00	0.00	0.10	0.00	0.00	0.00	0.00	0.08	0.00	0.00	0.00	0.00	0.00	0.00	0.00	0.00	0.00	0.00
**Quarter**	1	0.00	0.48	0.00	0.00	0.00	0.00	0.00	0.00	0.00	0.02	0.00	0.00	0.00	0.00	0.08	0.00	0.00	0.00	0.00	0.02
	2	0.00	0.82	0.00	0.00	0.03	0.00	0.00	0.00	0.00	0.05	0.00	0.00	0.00	0.00	0.00	0.00	0.00	0.00	0.00	0.00
	3	0.00	0.22	0.00	0.00	0.03	0.00	0.00	0.00	0.00	0.02	0.00	0.23	0.11	0.00	0.00	0.00	0.00	0.00	0.00	0.03
	4	0.00	0.21	0.13	0.00	0.03	0.00	0.00	0.00	0.00	0.04	0.00	0.21	0.00	0.00	0.00	0.00	0.00	0.00	0.00	0.01
**Year**	16/17	0.00	7.00	1.00	0.00	3.00	0.00	0.00	0.00	0.00	11.00	0.00	2.00	1.00	0.00	2.00	0.00	0.00	0.00	0.00	5.00
**Episodes**	F/N	0.00	0.44	0.03	0.00	0.02	0.00	0.00	0.00	0.00	0.04	0.00	0.11	0.03	0.00	0.02	0.00	0.00	0.00	0.00	0.02

Mean weekly incidence rate per 100,000 Persons.

Measles (ICD10: B05)

			M										F									
			<1yr	1-4yrs	5-14yrs	15-24yrs	25-44yrs	45-64yrs	65-74yrs	75-84yrs	85+yrs	All Ages	<1yr	1-4yrs	5-14yrs	15-24yrs	25-44yrs	45-64yrs	65-74yrs	75-84yrs	85+yrs	All Ages
**North**	Quarter	1	0.00	0.00	0.00	0.00	0.00	0.00	0.00	0.00	0.00	0.00	0.00	0.00	0.00	0.00	0.00	0.00	0.00	0.00	0.00	0.00
		2	0.00	0.00	0.00	0.00	0.00	0.00	0.00	0.00	0.00	0.00	0.00	0.00	0.00	0.00	0.00	0.00	0.00	0.00	0.00	0.00
		3	0.00	0.00	0.00	0.00	0.00	0.00	0.00	0.00	0.00	0.00	0.00	0.00	0.00	0.00	0.00	0.00	0.00	0.00	0.00	0.00
		4	0.00	0.00	0.00	0.00	0.00	0.00	0.00	0.00	0.00	0.00	0.00	0.00	0.00	0.00	0.00	0.00	0.00	0.00	0.00	0.00
	Year	16/17	0.00	0.00	0.00	0.00	0.00	0.00	0.00	0.00	0.00	0.00	0.00	0.00	0.00	0.00	0.00	0.00	0.00	0.00	0.00	0.00
	Episodes	F/N	0.00	0.00	0.00	0.00	0.00	0.00	0.00	0.00	0.00	0.00	0.00	0.00	0.00	0.00	0.00	0.00	0.00	0.00	0.00	0.00
**South**	Quarter	1	0.00	1.05	0.00	0.00	0.00	0.00	0.00	0.00	0.00	0.04	0.00	0.00	0.00	0.00	0.18	0.00	0.00	0.00	0.00	0.04
		2	0.00	0.00	0.00	0.00	0.00	0.00	0.00	0.00	0.00	0.00	0.00	0.00	0.00	0.00	0.00	0.00	0.00	0.00	0.00	0.00
		3	0.00	0.00	0.00	0.00	0.13	0.00	0.00	0.00	0.00	0.03	0.00	0.00	0.00	0.00	0.00	0.00	0.00	0.00	0.00	0.00
		4	0.00	0.00	0.00	0.00	0.00	0.00	0.00	0.00	0.00	0.00	0.00	0.00	0.00	0.00	0.00	0.00	0.00	0.00	0.00	0.00
	Year	16/17	0.00	1.00	0.00	0.00	1.00	0.00	0.00	0.00	0.00	2.00	0.00	0.00	0.00	0.00	1.00	0.00	0.00	0.00	0.00	1.00
	Episodes	F/N	0.00	0.26	0.00	0.00	0.03	0.00	0.00	0.00	0.00	0.02	0.00	0.00	0.00	0.00	0.04	0.00	0.00	0.00	0.00	0.01
**London**	Quarter	1	0.00	0.89	0.00	0.00	0.00	0.00	0.00	0.00	0.00	0.05	0.00	0.00	0.00	0.00	0.12	0.00	0.00	0.00	0.00	0.05
		2	0.00	1.61	0.00	0.00	0.12	0.00	0.00	0.00	0.00	0.14	0.00	0.00	0.00	0.00	0.00	0.00	0.00	0.00	0.00	0.00
		3	0.00	0.86	0.00	0.00	0.00	0.00	0.00	0.00	0.00	0.05	0.00	0.93	0.43	0.00	0.00	0.00	0.00	0.00	0.00	0.10
		4	0.00	0.84	0.00	0.00	0.12	0.00	0.00	0.00	0.00	0.10	0.00	0.85	0.00	0.00	0.00	0.00	0.00	0.00	0.00	0.05
	Year	16/17	0.00	5.00	0.00	0.00	2.00	0.00	0.00	0.00	0.00	7.00	0.00	2.00	1.00	0.00	1.00	0.00	0.00	0.00	0.00	4.00
	Episodes	F/N	0.00	1.06	0.00	0.00	0.06	0.00	0.00	0.00	0.00	0.09	0.00	0.44	0.11	0.00	0.03	0.00	0.00	0.00	0.00	0.05
**Midlands And East**	Quarter	1	0.00	0.00	0.00	0.00	0.00	0.00	0.00	0.00	0.00	0.00	0.00	0.00	0.00	0.00	0.00	0.00	0.00	0.00	0.00	0.00
		2	0.00	1.68	0.00	0.00	0.00	0.00	0.00	0.00	0.00	0.07	0.00	0.00	0.00	0.00	0.00	0.00	0.00	0.00	0.00	0.00
		3	0.00	0.00	0.00	0.00	0.00	0.00	0.00	0.00	0.00	0.00	0.00	0.00	0.00	0.00	0.00	0.00	0.00	0.00	0.00	0.00
		4	0.00	0.00	0.53	0.00	0.00	0.00	0.00	0.00	0.00	0.06	0.00	0.00	0.00	0.00	0.00	0.00	0.00	0.00	0.00	0.00
	Year	16/17	0.00	1.00	1.00	0.00	0.00	0.00	0.00	0.00	0.00	2.00	0.00	0.00	0.00	0.00	0.00	0.00	0.00	0.00	0.00	0.00
	Episodes	F/N	0.00	0.44	0.13	0.00	0.00	0.00	0.00	0.00	0.00	0.04	0.00	0.00	0.00	0.00	0.00	0.00	0.00	0.00	0.00	0.00

Mean weekly incidence rate per 100,000 Persons.

Mumps (ICD10: B26)

		M										F									
		<1yr	1-4yrs	5-14yrs	15-24yrs	25-44yrs	45-64yrs	65-74yrs	75-84yrs	85+yrs	All Ages	<1yr	1-4yrs	5-14yrs	15-24yrs	25-44yrs	45-64yrs	65-74yrs	75-84yrs	85+yrs	All Ages
**4 weekly**	1	0.00	0.00	0.27	0.00	0.00	0.00	0.00	0.00	0.00	0.03	0.00	0.00	0.00	0.00	0.00	0.17	0.00	0.00	0.00	0.03
	2	0.00	0.65	0.00	0.43	0.00	0.00	0.00	0.00	0.00	0.07	0.00	0.88	0.00	0.00	0.00	0.00	0.00	0.00	0.00	0.04
	3	0.00	1.93	0.00	0.20	0.21	0.00	0.00	0.00	0.00	0.18	0.00	0.00	0.00	0.00	0.21	0.32	0.31	0.00	0.00	0.17
	4	0.00	0.00	0.26	0.48	0.11	0.23	0.45	0.00	0.00	0.23	0.00	0.00	0.00	0.00	0.13	0.24	0.00	0.00	0.00	0.09
	5	0.00	0.00	0.00	0.20	0.21	0.11	0.00	0.00	0.00	0.11	0.00	0.00	0.44	0.49	0.00	0.11	0.00	0.00	0.00	0.13
	6	0.00	0.00	0.00	0.19	0.00	0.49	0.00	0.00	0.00	0.15	0.00	0.00	0.00	0.21	0.31	0.17	0.00	0.00	0.00	0.18
	7	0.00	0.00	0.88	0.63	0.21	0.00	0.00	0.00	0.00	0.24	0.00	0.00	0.66	0.20	0.11	0.31	0.00	0.00	0.00	0.19
	8	0.00	0.74	0.38	0.24	0.11	0.00	0.00	0.00	0.00	0.15	0.00	0.00	0.00	0.74	0.11	0.00	0.00	0.00	0.00	0.13
	9	0.00	0.00	0.00	0.00	0.39	0.10	0.00	0.00	0.00	0.12	0.00	0.00	0.91	1.48	0.00	0.00	0.00	0.00	0.00	0.28
	10	0.00	0.00	0.34	0.38	0.10	0.00	0.00	0.00	0.00	0.12	0.00	0.00	0.70	0.53	0.11	0.00	0.00	0.00	0.00	0.17
	11	0.00	1.16	0.94	0.38	0.00	0.17	0.00	0.00	0.00	0.27	0.00	0.61	0.94	0.00	0.31	0.00	0.00	0.00	0.00	0.20
	12	0.00	0.00	0.23	0.57	0.20	0.00	0.00	0.00	0.00	0.15	0.00	0.68	0.25	0.00	0.10	0.00	0.00	0.00	0.00	0.08
	13	0.00	0.00	0.00	0.74	0.10	0.19	0.00	0.00	0.00	0.18	0.00	1.11	0.00	0.17	0.20	0.00	0.00	0.00	0.00	0.14
**Quarter**	1	0.00	0.79	0.10	0.20	0.06	0.00	0.00	0.00	0.00	0.09	0.00	0.27	0.00	0.00	0.06	0.16	0.10	0.00	0.00	0.08
	2	0.00	0.00	0.26	0.31	0.12	0.24	0.13	0.00	0.00	0.18	0.00	0.00	0.20	0.20	0.16	0.15	0.00	0.00	0.00	0.13
	3	0.00	0.23	0.30	0.32	0.21	0.03	0.00	0.00	0.00	0.15	0.00	0.00	0.51	0.91	0.03	0.10	0.00	0.00	0.00	0.20
	4	0.00	0.36	0.36	0.52	0.09	0.11	0.00	0.00	0.00	0.18	0.00	0.74	0.48	0.05	0.22	0.00	0.00	0.00	0.00	0.15
**Year**	16/17	0.00	6.00	11.00	19.00	13.00	8.00	1.00	0.00	0.00	58.00	0.00	4.00	12.00	15.00	14.00	9.00	1.00	0.00	0.00	55.00
**Episodes**	F/N	0.00	0.34	0.25	0.33	0.12	0.10	0.03	0.00	0.00	0.15	0.00	0.25	0.29	0.29	0.12	0.10	0.02	0.00	0.00	0.14

Mean weekly incidence rate per 100,000 Persons.

Mumps (ICD10: B26)

			M										F									
			<1yr	1-4yrs	5-14yrs	15-24yrs	25-44yrs	45-64yrs	65-74yrs	75-84yrs	85+yrs	All Ages	<1yr	1-4yrs	5-14yrs	15-24yrs	25-44yrs	45-64yrs	65-74yrs	75-84yrs	85+yrs	All Ages
**North**	Quarter	1	0.00	1.61	0.00	0.25	0.13	0.00	0.00	0.00	0.00	0.14	0.00	0.00	0.00	0.00	0.13	0.12	0.00	0.00	0.00	0.06
		2	0.00	0.00	0.58	0.44	0.24	0.12	0.00	0.00	0.00	0.22	0.00	0.00	0.00	0.00	0.25	0.12	0.00	0.00	0.00	0.09
		3	0.00	0.00	0.30	0.22	0.12	0.00	0.00	0.00	0.00	0.10	0.00	0.00	0.00	0.42	0.13	0.00	0.00	0.00	0.00	0.10
		4	0.00	0.00	0.00	0.67	0.25	0.00	0.00	0.00	0.00	0.16	0.00	0.84	0.95	0.21	0.53	0.00	0.00	0.00	0.00	0.29
	Year	16/17	0.00	2.00	3.00	7.00	6.00	1.00	0.00	0.00	0.00	19.00	0.00	1.00	3.00	3.00	8.00	2.00	0.00	0.00	0.00	17.00
	Episodes	F/N	0.00	0.40	0.23	0.40	0.19	0.03	0.00	0.00	0.00	0.16	0.00	0.21	0.23	0.16	0.26	0.06	0.00	0.00	0.00	0.14
**South**	Quarter	1	0.00	0.00	0.00	0.00	0.00	0.00	0.00	0.00	0.00	0.00	0.00	1.08	0.00	0.00	0.00	0.00	0.38	0.00	0.00	0.09
		2	0.00	0.00	0.00	0.24	0.00	0.12	0.00	0.00	0.00	0.07	0.00	0.00	0.29	0.24	0.15	0.27	0.00	0.00	0.00	0.17
		3	0.00	0.00	0.00	1.05	0.25	0.13	0.00	0.00	0.00	0.24	0.00	0.00	0.63	1.75	0.00	0.13	0.00	0.00	0.00	0.33
		4	0.00	1.43	0.55	0.96	0.00	0.00	0.00	0.00	0.00	0.26	0.00	0.76	0.00	0.00	0.00	0.00	0.00	0.00	0.00	0.03
	Year	16/17	0.00	2.00	2.00	9.00	2.00	2.00	0.00	0.00	0.00	17.00	0.00	2.00	3.00	8.00	1.00	3.00	1.00	0.00	0.00	18.00
	Episodes	F/N	0.00	0.35	0.13	0.56	0.06	0.06	0.00	0.00	0.00	0.14	0.00	0.45	0.23	0.49	0.04	0.10	0.09	0.00	0.00	0.16
**London**	Quarter	1	0.00	0.00	0.42	0.53	0.13	0.00	0.00	0.00	0.00	0.16	0.00	0.00	0.00	0.00	0.12	0.53	0.00	0.00	0.00	0.15
		2	0.00	0.00	0.00	0.00	0.00	0.23	0.00	0.00	0.00	0.05	0.00	0.00	0.00	0.00	0.23	0.00	0.00	0.00	0.00	0.09
		3	0.00	0.90	0.41	0.00	0.00	0.00	0.00	0.00	0.00	0.11	0.00	0.00	0.43	0.85	0.00	0.25	0.00	0.00	0.00	0.20
		4	0.00	0.00	0.41	0.44	0.12	0.23	0.00	0.00	0.00	0.20	0.00	0.00	0.43	0.00	0.11	0.00	0.00	0.00	0.00	0.10
	Year	16/17	0.00	1.00	3.00	2.00	2.00	2.00	0.00	0.00	0.00	10.00	0.00	0.00	2.00	2.00	4.00	3.00	0.00	0.00	0.00	11.00
	Episodes	F/N	0.00	0.22	0.31	0.24	0.06	0.12	0.00	0.00	0.00	0.13	0.00	0.00	0.21	0.21	0.12	0.19	0.00	0.00	0.00	0.13
**Midlands And East**	Quarter	1	0.00	1.56	0.00	0.00	0.00	0.00	0.00	0.00	0.00	0.07	0.00	0.00	0.00	0.00	0.00	0.00	0.00	0.00	0.00	0.00
		2	0.00	0.00	0.44	0.55	0.24	0.47	0.52	0.00	0.00	0.37	0.00	0.00	0.50	0.56	0.00	0.20	0.00	0.00	0.00	0.17
		3	0.00	0.00	0.47	0.00	0.48	0.00	0.00	0.00	0.00	0.17	0.00	0.00	0.99	0.63	0.00	0.00	0.00	0.00	0.00	0.17
		4	0.00	0.00	0.48	0.00	0.00	0.21	0.00	0.00	0.00	0.12	0.00	1.37	0.52	0.00	0.25	0.00	0.00	0.00	0.00	0.18
	Year	16/17	0.00	1.00	3.00	1.00	3.00	3.00	1.00	0.00	0.00	12.00	0.00	1.00	4.00	2.00	1.00	1.00	0.00	0.00	0.00	9.00
	Episodes	F/N	0.00	0.38	0.35	0.15	0.18	0.18	0.14	0.00	0.00	0.19	0.00	0.34	0.50	0.30	0.06	0.05	0.00	0.00	0.00	0.13

Mean weekly incidence rate per 100,000 Persons.

Rubella (ICD10: B06)

		M										F									
		<1yr	1-4yrs	5-14yrs	15-24yrs	25-44yrs	45-64yrs	65-74yrs	75-84yrs	85+yrs	All Ages	<1yr	1-4yrs	5-14yrs	15-24yrs	25-44yrs	45-64yrs	65-74yrs	75-84yrs	85+yrs	All Ages
**4 weekly**	1	0.00	0.00	0.00	0.00	0.00	0.00	0.00	0.00	0.00	0.00	0.00	0.00	0.00	0.00	0.00	0.00	0.00	0.00	0.00	0.00
	2	0.00	1.30	0.00	0.00	0.00	0.00	0.00	0.00	0.00	0.06	0.00	0.00	0.00	0.00	0.00	0.00	0.00	0.00	0.00	0.00
	3	0.00	0.00	0.00	0.00	0.00	0.00	0.00	0.00	0.00	0.00	0.00	0.89	0.00	0.00	0.00	0.00	0.00	0.00	0.00	0.04
	4	0.00	0.00	0.00	0.00	0.00	0.00	0.00	0.00	0.00	0.00	0.00	0.00	0.00	0.00	0.00	0.00	0.00	0.00	0.00	0.00
	5	0.00	0.00	0.00	0.00	0.00	0.00	0.00	0.00	0.00	0.00	0.00	0.00	0.00	0.00	0.00	0.00	0.00	0.00	0.00	0.00
	6	0.00	0.00	0.00	0.00	0.00	0.00	0.00	0.00	0.00	0.00	0.00	0.00	0.00	0.00	0.00	0.00	0.00	0.00	0.00	0.00
	7	0.00	0.00	0.00	0.00	0.00	0.00	0.00	0.00	0.00	0.00	0.00	0.00	0.00	0.00	0.00	0.00	0.00	0.00	0.00	0.00
	8	0.00	0.00	0.00	0.00	0.00	0.00	0.00	0.00	0.00	0.00	0.00	0.00	0.00	0.00	0.00	0.00	0.00	0.00	0.00	0.00
	9	0.00	1.13	0.00	0.00	0.00	0.00	0.00	0.00	0.00	0.05	0.00	0.00	0.00	0.00	0.00	0.00	0.00	0.00	0.00	0.00
	10	0.00	0.00	0.00	0.00	0.00	0.00	0.00	0.00	0.00	0.00	0.00	0.00	0.00	0.00	0.00	0.00	0.00	0.00	0.00	0.00
	11	0.00	0.00	0.00	0.00	0.00	0.00	0.00	0.00	0.00	0.00	0.00	0.00	0.00	0.00	0.00	0.00	0.00	0.00	0.00	0.00
	12	0.00	0.00	0.00	0.00	0.00	0.00	0.00	0.00	0.00	0.00	0.00	1.19	0.00	0.00	0.00	0.00	0.00	0.00	0.00	0.05
	13	0.00	0.00	0.00	0.00	0.00	0.00	0.00	0.00	0.00	0.00	0.00	0.00	0.00	0.00	0.00	0.00	0.00	0.00	0.00	0.00
**Quarter**	1	0.00	0.40	0.00	0.00	0.00	0.00	0.00	0.00	0.00	0.02	0.00	0.27	0.00	0.00	0.00	0.00	0.00	0.00	0.00	0.01
	2	0.00	0.00	0.00	0.00	0.00	0.00	0.00	0.00	0.00	0.00	0.00	0.00	0.00	0.00	0.00	0.00	0.00	0.00	0.00	0.00
	3	0.00	0.35	0.00	0.00	0.00	0.00	0.00	0.00	0.00	0.02	0.00	0.00	0.00	0.00	0.00	0.00	0.00	0.00	0.00	0.00
	4	0.00	0.00	0.00	0.00	0.00	0.00	0.00	0.00	0.00	0.00	0.00	0.37	0.00	0.00	0.00	0.00	0.00	0.00	0.00	0.02
**Year**	16/17	0.00	2.00	0.00	0.00	0.00	0.00	0.00	0.00	0.00	2.00	0.00	2.00	0.00	0.00	0.00	0.00	0.00	0.00	0.00	2.00
**Episodes**	F/N	0.00	0.18	0.00	0.00	0.00	0.00	0.00	0.00	0.00	0.01	0.00	0.16	0.00	0.00	0.00	0.00	0.00	0.00	0.00	0.01

Mean weekly incidence rate per 100,000 Persons.

Rubella (ICD10: B06)

			M										F									
			<1yr	1-4yrs	5-14yrs	15-24yrs	25-44yrs	45-64yrs	65-74yrs	75-84yrs	85+yrs	All Ages	<1yr	1-4yrs	5-14yrs	15-24yrs	25-44yrs	45-64yrs	65-74yrs	75-84yrs	85+yrs	All Ages
**North**	Quarter	1	0.00	0.00	0.00	0.00	0.00	0.00	0.00	0.00	0.00	0.00	0.00	0.00	0.00	0.00	0.00	0.00	0.00	0.00	0.00	0.00
		2	0.00	0.00	0.00	0.00	0.00	0.00	0.00	0.00	0.00	0.00	0.00	0.00	0.00	0.00	0.00	0.00	0.00	0.00	0.00	0.00
		3	0.00	0.00	0.00	0.00	0.00	0.00	0.00	0.00	0.00	0.00	0.00	0.00	0.00	0.00	0.00	0.00	0.00	0.00	0.00	0.00
		4	0.00	0.00	0.00	0.00	0.00	0.00	0.00	0.00	0.00	0.00	0.00	0.00	0.00	0.00	0.00	0.00	0.00	0.00	0.00	0.00
	Year	16/17	0.00	0.00	0.00	0.00	0.00	0.00	0.00	0.00	0.00	0.00	0.00	0.00	0.00	0.00	0.00	0.00	0.00	0.00	0.00	0.00
	Episodes	F/N	0.00	0.00	0.00	0.00	0.00	0.00	0.00	0.00	0.00	0.00	0.00	0.00	0.00	0.00	0.00	0.00	0.00	0.00	0.00	0.00
**South**	Quarter	1	0.00	0.00	0.00	0.00	0.00	0.00	0.00	0.00	0.00	0.00	0.00	1.09	0.00	0.00	0.00	0.00	0.00	0.00	0.00	0.04
		2	0.00	0.00	0.00	0.00	0.00	0.00	0.00	0.00	0.00	0.00	0.00	0.00	0.00	0.00	0.00	0.00	0.00	0.00	0.00	0.00
		3	0.00	0.00	0.00	0.00	0.00	0.00	0.00	0.00	0.00	0.00	0.00	0.00	0.00	0.00	0.00	0.00	0.00	0.00	0.00	0.00
		4	0.00	0.00	0.00	0.00	0.00	0.00	0.00	0.00	0.00	0.00	0.00	0.00	0.00	0.00	0.00	0.00	0.00	0.00	0.00	0.00
	Year	16/17	0.00	0.00	0.00	0.00	0.00	0.00	0.00	0.00	0.00	0.00	0.00	1.00	0.00	0.00	0.00	0.00	0.00	0.00	0.00	1.00
	Episodes	F/N	0.00	0.00	0.00	0.00	0.00	0.00	0.00	0.00	0.00	0.00	0.00	0.27	0.00	0.00	0.00	0.00	0.00	0.00	0.00	0.01
**London**	Quarter	1	0.00	0.00	0.00	0.00	0.00	0.00	0.00	0.00	0.00	0.00	0.00	0.00	0.00	0.00	0.00	0.00	0.00	0.00	0.00	0.00
		2	0.00	0.00	0.00	0.00	0.00	0.00	0.00	0.00	0.00	0.00	0.00	0.00	0.00	0.00	0.00	0.00	0.00	0.00	0.00	0.00
		3	0.00	0.00	0.00	0.00	0.00	0.00	0.00	0.00	0.00	0.00	0.00	0.00	0.00	0.00	0.00	0.00	0.00	0.00	0.00	0.00
		4	0.00	0.00	0.00	0.00	0.00	0.00	0.00	0.00	0.00	0.00	0.00	0.00	0.00	0.00	0.00	0.00	0.00	0.00	0.00	0.00
	Year	16/17	0.00	0.00	0.00	0.00	0.00	0.00	0.00	0.00	0.00	0.00	0.00	0.00	0.00	0.00	0.00	0.00	0.00	0.00	0.00	0.00
	Episodes	F/N	0.00	0.00	0.00	0.00	0.00	0.00	0.00	0.00	0.00	0.00	0.00	0.00	0.00	0.00	0.00	0.00	0.00	0.00	0.00	0.00
**Midlands And East**	Quarter	1	0.00	1.60	0.00	0.00	0.00	0.00	0.00	0.00	0.00	0.07	0.00	0.00	0.00	0.00	0.00	0.00	0.00	0.00	0.00	0.00
		2	0.00	0.00	0.00	0.00	0.00	0.00	0.00	0.00	0.00	0.00	0.00	0.00	0.00	0.00	0.00	0.00	0.00	0.00	0.00	0.00
		3	0.00	1.39	0.00	0.00	0.00	0.00	0.00	0.00	0.00	0.06	0.00	0.00	0.00	0.00	0.00	0.00	0.00	0.00	0.00	0.00
		4	0.00	0.00	0.00	0.00	0.00	0.00	0.00	0.00	0.00	0.00	0.00	1.47	0.00	0.00	0.00	0.00	0.00	0.00	0.00	0.06
	Year	16/17	0.00	2.00	0.00	0.00	0.00	0.00	0.00	0.00	0.00	2.00	0.00	1.00	0.00	0.00	0.00	0.00	0.00	0.00	0.00	1.00
	Episodes	F/N	0.00	0.73	0.00	0.00	0.00	0.00	0.00	0.00	0.00	0.03	0.00	0.36	0.00	0.00	0.00	0.00	0.00	0.00	0.00	0.02

5. Skin Contagions:

Mean weekly incidence rate per 100,000 Persons.

Bullous Disorders (ICD10: L10-L14)

		M										F									
		<1yr	1-4yrs	5-14yrs	15-24yrs	25-44yrs	45-64yrs	65-74yrs	75-84yrs	85+yrs	All Ages	<1yr	1-4yrs	5-14yrs	15-24yrs	25-44yrs	45-64yrs	65-74yrs	75-84yrs	85+yrs	All Ages
**4 weekly**	1	0.00	0.00	0.00	0.00	0.00	0.09	0.92	0.00	2.62	0.14	0.00	0.00	0.00	0.00	0.17	0.19	0.00	0.00	0.00	0.12
	2	0.00	0.00	0.00	0.00	0.10	0.00	0.00	0.92	2.45	0.14	0.00	0.00	0.35	0.00	0.00	0.00	0.00	1.56	0.00	0.15
	3	0.00	0.00	0.00	0.00	0.21	0.00	0.00	0.00	6.66	0.16	0.00	0.00	0.25	0.00	0.00	0.00	0.00	0.42	0.00	0.05
	4	0.00	0.00	0.00	0.00	0.14	0.11	0.00	1.74	7.11	0.21	0.00	0.00	0.00	0.19	0.00	0.11	0.30	0.00	3.57	0.23
	5	0.00	0.00	0.00	0.00	0.10	0.19	0.27	1.51	1.30	0.17	0.00	0.00	0.00	0.00	0.00	0.11	0.42	0.41	2.16	0.19
	6	0.00	0.00	0.00	0.00	0.10	0.49	0.00	0.51	3.78	0.27	0.00	0.00	0.00	0.00	0.21	0.00	0.00	0.00	0.83	0.08
	7	0.00	0.00	0.00	0.00	0.00	0.20	0.00	0.51	3.66	0.15	0.00	0.00	0.00	0.00	0.19	0.00	0.51	1.94	1.47	0.28
	8	0.00	0.00	0.00	0.00	0.00	0.20	0.68	0.00	1.23	0.14	0.00	0.00	0.00	0.00	0.10	0.00	0.26	0.65	0.68	0.14
	9	0.00	0.00	0.00	0.00	0.00	0.28	0.95	1.03	1.52	0.26	0.00	0.00	0.00	0.00	0.00	0.00	0.39	0.40	5.53	0.22
	10	0.00	0.00	0.00	0.00	0.00	0.00	0.97	0.00	0.00	0.10	0.00	0.73	0.00	0.00	0.00	0.21	0.00	0.77	0.70	0.16
	11	0.00	0.00	0.00	0.00	0.00	0.00	0.00	0.83	3.80	0.13	0.00	0.72	0.00	0.00	0.11	0.34	0.00	0.40	2.21	0.27
	12	0.00	0.00	0.22	0.00	0.00	0.00	0.00	0.00	1.33	0.05	0.00	0.00	0.00	0.00	0.00	0.30	0.00	1.11	0.63	0.16
	13	0.00	0.00	0.00	0.00	0.00	0.09	0.51	0.00	0.00	0.08	0.00	0.00	0.00	0.00	0.20	0.00	0.76	0.00	0.85	0.11
**Quarter**	1	0.00	0.00	0.00	0.00	0.09	0.03	0.35	0.28	3.81	0.15	0.00	0.00	0.19	0.00	0.06	0.07	0.00	0.61	0.00	0.11
	2	0.00	0.00	0.00	0.00	0.10	0.28	0.08	1.22	3.90	0.21	0.00	0.00	0.00	0.06	0.11	0.06	0.28	0.44	2.08	0.19
	3	0.00	0.00	0.00	0.00	0.00	0.15	0.66	0.32	1.52	0.16	0.00	0.22	0.00	0.00	0.03	0.06	0.28	0.81	2.36	0.19
	4	0.00	0.00	0.07	0.00	0.00	0.03	0.30	0.26	1.58	0.09	0.00	0.22	0.00	0.00	0.09	0.20	0.23	0.47	1.13	0.17
**Year**	16/17	0.00	0.00	1.00	0.00	6.00	11.00	13.00	8.00	18.00	57.00	0.00	2.00	2.00	1.00	7.00	9.00	7.00	15.00	20.00	63.00
**Episodes**	F/N	0.00	0.00	0.02	0.00	0.05	0.13	0.34	0.53	2.72	0.15	0.00	0.11	0.05	0.01	0.08	0.10	0.20	0.58	1.41	0.17

Mean weekly incidence rate per 100,000 Persons.

Bullous Disorders (ICD10: L10-L14)

			M										F									
			<1yr	1-4yrs	5-14yrs	15-24yrs	25-44yrs	45-64yrs	65-74yrs	75-84yrs	85+yrs	All Ages	<1yr	1-4yrs	5-14yrs	15-24yrs	25-44yrs	45-64yrs	65-74yrs	75-84yrs	85+yrs	All Ages
**North**	Quarter	1	0.00	0.00	0.00	0.00	0.37	0.14	0.33	0.00	0.00	0.17	0.00	0.00	0.31	0.00	0.00	0.13	0.00	0.51	0.00	0.10
		2	0.00	0.00	0.00	0.00	0.12	0.24	0.31	0.59	6.76	0.28	0.00	0.00	0.00	0.22	0.00	0.25	0.00	0.00	1.86	0.16
		3	0.00	0.00	0.00	0.00	0.00	0.00	0.96	0.62	1.88	0.16	0.00	0.00	0.00	0.00	0.00	0.00	0.32	1.48	2.00	0.19
		4	0.00	0.00	0.00	0.00	0.00	0.00	0.63	0.00	0.00	0.06	0.00	0.00	0.00	0.00	0.13	0.12	0.00	0.50	1.04	0.13
	Year	16/17	0.00	0.00	0.00	0.00	4.00	3.00	7.00	2.00	5.00	21.00	0.00	0.00	1.00	1.00	1.00	4.00	1.00	5.00	5.00	18.00
	Episodes	F/N	0.00	0.00	0.00	0.00	0.12	0.10	0.55	0.31	2.25	0.17	0.00	0.00	0.08	0.06	0.03	0.13	0.08	0.61	1.24	0.14
**South**	Quarter	1	0.00	0.00	0.00	0.00	0.00	0.00	0.00	0.00	4.03	0.09	0.00	0.00	0.00	0.00	0.00	0.17	0.00	0.00	0.00	0.04
		2	0.00	0.00	0.00	0.00	0.16	0.00	0.00	0.59	1.48	0.11	0.00	0.00	0.00	0.00	0.00	0.00	0.64	0.95	1.83	0.20
		3	0.00	0.00	0.00	0.00	0.00	0.12	0.65	0.65	1.51	0.17	0.00	0.00	0.00	0.00	0.00	0.00	0.31	0.95	4.35	0.26
		4	0.00	0.00	0.27	0.00	0.00	0.11	0.00	0.00	3.25	0.13	0.00	0.00	0.00	0.00	0.00	0.00	0.00	0.50	3.49	0.16
	Year	16/17	0.00	0.00	1.00	0.00	1.00	2.00	2.00	2.00	6.00	14.00	0.00	0.00	0.00	0.00	0.00	1.00	3.00	5.00	11.00	20.00
	Episodes	F/N	0.00	0.00	0.07	0.00	0.04	0.06	0.16	0.31	2.55	0.12	0.00	0.00	0.00	0.00	0.00	0.04	0.25	0.61	2.41	0.17
**London**	Quarter	1	0.00	0.00	0.00	0.00	0.00	0.00	1.09	0.00	5.05	0.11	0.00	0.00	0.44	0.00	0.26	0.00	0.00	0.00	0.00	0.16
		2	0.00	0.00	0.00	0.00	0.12	0.68	0.00	3.72	4.74	0.34	0.00	0.00	0.00	0.00	0.00	0.00	0.00	0.00	0.00	0.00
		3	0.00	0.00	0.00	0.00	0.00	0.25	0.00	0.00	0.00	0.05	0.00	0.90	0.00	0.00	0.12	0.26	0.00	0.00	3.10	0.20
		4	0.00	0.00	0.00	0.00	0.00	0.00	0.00	0.00	0.00	0.00	0.00	0.89	0.00	0.00	0.00	0.24	0.93	0.00	0.00	0.14
	Year	16/17	0.00	0.00	0.00	0.00	1.00	4.00	1.00	2.00	2.00	10.00	0.00	2.00	1.00	0.00	3.00	2.00	1.00	0.00	1.00	10.00
	Episodes	F/N	0.00	0.00	0.00	0.00	0.03	0.24	0.27	0.98	2.49	0.13	0.00	0.44	0.11	0.00	0.09	0.12	0.23	0.00	0.76	0.12
**Midlands And East**	Quarter	1	0.00	0.00	0.00	0.00	0.00	0.00	0.00	1.14	6.16	0.21	0.00	0.00	0.00	0.00	0.00	0.00	0.00	1.91	0.00	0.14
		2	0.00	0.00	0.00	0.00	0.00	0.21	0.00	0.00	2.62	0.12	0.00	0.00	0.00	0.00	0.46	0.00	0.48	0.81	4.64	0.40
		3	0.00	0.00	0.00	0.00	0.00	0.22	1.01	0.00	2.69	0.24	0.00	0.00	0.00	0.00	0.00	0.00	0.48	0.80	0.00	0.11
		4	0.00	0.00	0.00	0.00	0.00	0.00	0.57	1.02	3.06	0.19	0.00	0.00	0.00	0.00	0.25	0.42	0.00	0.87	0.00	0.24
	Year	16/17	0.00	0.00	0.00	0.00	0.00	2.00	3.00	2.00	5.00	12.00	0.00	0.00	0.00	0.00	3.00	2.00	2.00	5.00	3.00	15.00
	Episodes	F/N	0.00	0.00	0.00	0.00	0.00	0.11	0.39	0.53	3.61	0.19	0.00	0.00	0.00	0.00	0.18	0.10	0.24	1.09	1.22	0.23

Mean weekly incidence rate per 100,000 Persons.

Chickenpox (ICD10: B01)

		M										F									
		<1yr	1-4yrs	5-14yrs	15-24yrs	25-44yrs	45-64yrs	65-74yrs	75-84yrs	85+yrs	All Ages	<1yr	1-4yrs	5-14yrs	15-24yrs	25-44yrs	45-64yrs	65-74yrs	75-84yrs	85+yrs	All Ages
**4 weekly**	1	0.00	71.78	27.77	2.67	0.97	0.11	0.00	0.00	0.00	7.26	0.00	82.46	25.50	2.41	0.90	0.46	0.00	0.00	0.00	6.89
	2	0.00	107.89	24.56	2.00	1.40	0.00	0.25	0.00	0.00	8.71	0.00	79.85	28.45	3.75	1.76	0.34	0.00	0.00	0.00	7.76
	3	0.00	73.51	23.82	0.41	0.69	0.00	0.50	0.00	0.00	6.58	0.00	68.17	24.05	1.09	1.37	0.28	0.00	0.39	0.00	6.38
	4	43.30	29.52	8.93	1.58	0.79	0.12	0.00	0.56	0.00	2.92	0.00	46.03	10.98	0.82	1.54	0.11	0.44	0.00	0.00	3.97
	5	0.00	41.61	5.61	0.20	0.62	0.00	0.00	0.00	0.00	2.84	13.05	35.52	5.85	0.19	0.85	0.00	0.26	0.00	0.00	2.57
	6	8.09	29.65	7.53	0.38	0.10	0.19	0.00	0.00	0.00	2.36	0.00	33.30	5.44	1.09	0.21	0.20	0.26	0.41	0.00	2.29
	7	26.86	44.41	8.55	1.01	0.63	0.00	0.83	1.55	0.00	3.70	0.00	38.51	8.86	0.70	0.60	0.49	0.26	0.00	0.00	3.04
	8	31.21	48.59	15.11	1.19	0.42	0.11	0.00	0.00	0.00	4.54	31.17	46.19	10.61	0.90	0.52	0.00	0.00	1.19	0.00	3.63
	9	25.98	68.28	17.53	0.55	1.01	0.48	0.00	0.00	0.00	5.97	0.00	50.52	15.55	1.64	1.31	0.21	0.00	0.00	0.00	4.65
	10	54.74	82.87	26.99	0.65	0.93	0.18	0.00	0.00	0.00	8.00	22.60	86.21	19.91	1.04	0.41	0.21	0.00	0.00	0.00	6.51
	11	10.99	113.96	25.26	1.28	1.42	0.68	0.00	0.00	0.00	9.22	66.53	111.08	26.46	1.44	1.12	0.00	0.00	0.00	0.00	8.97
	12	94.88	122.15	34.00	0.59	1.00	1.04	0.00	0.00	0.00	11.28	54.92	137.55	28.07	2.03	1.76	0.49	0.49	0.00	0.00	10.61
	13	123.38	104.54	16.64	1.65	1.19	0.36	0.00	0.00	0.00	8.63	72.51	107.73	24.87	3.82	2.41	0.56	0.00	0.00	0.00	9.44
**Quarter**	1	0.00	83.42	25.57	1.77	1.01	0.04	0.23	0.00	0.00	7.49	0.00	77.26	25.97	2.41	1.31	0.37	0.00	0.12	0.00	7.00
	2	18.27	33.26	7.23	0.80	0.53	0.09	0.00	0.16	0.00	2.71	3.73	38.74	7.67	0.80	0.77	0.17	0.27	0.12	0.00	2.97
	3	34.56	63.48	17.89	0.79	0.69	0.18	0.26	0.48	0.00	5.75	15.01	58.13	14.23	1.10	0.84	0.19	0.08	0.37	0.00	4.69
	4	74.82	111.64	25.45	1.14	1.24	0.69	0.00	0.00	0.00	9.63	61.21	113.27	25.70	2.24	1.63	0.32	0.15	0.00	0.00	9.24
**Year**	16/17	85.00	1260.00	800.00	47.00	97.00	21.00	4.00	2.00	0.00	2316.00	52.00	1192.00	747.00	73.00	111.00	25.00	6.00	3.00	0.00	2209.00
**Episodes**	F/N	31.65	72.20	18.81	1.12	0.86	0.25	0.12	0.16	0.00	6.33	19.68	71.22	18.19	1.62	1.13	0.26	0.13	0.15	0.00	5.92

Mean weekly incidence rate per 100,000 Persons.

Chickenpox (ICD10: B01)

			M										F									
			<1yr	1-4yrs	5-14yrs	15-24yrs	25-44yrs	45-64yrs	65-74yrs	75-84yrs	85+yrs	All Ages	<1yr	1-4yrs	5-14yrs	15-24yrs	25-44yrs	45-64yrs	65-74yrs	75-84yrs	85+yrs	All Ages
**North**	Quarter	1	0.00	60.58	20.32	1.06	1.27	0.00	0.31	0.00	0.00	5.29	0.00	76.67	24.13	1.36	0.81	0.00	0.00	0.48	0.00	5.95
		2	36.40	53.22	8.52	0.46	0.46	0.00	0.00	0.00	0.00	3.44	14.91	42.00	9.42	1.06	1.11	0.26	0.29	0.00	0.00	3.18
		3	45.45	71.09	13.59	0.22	0.98	0.26	0.00	0.00	0.00	5.09	0.00	63.98	13.86	0.44	0.90	0.38	0.00	0.00	0.00	4.30
		4	90.36	98.89	15.66	1.36	0.62	0.12	0.00	0.00	0.00	6.95	41.13	91.89	22.21	1.27	1.02	0.26	0.30	0.00	0.00	6.70
	Year	16/17	31.00	367.00	190.00	13.00	27.00	3.00	1.00	0.00	0.00	632.00	10.00	333.00	220.00	18.00	30.00	7.00	2.00	1.00	0.00	621.00
	Episodes	F/N	42.93	70.61	14.41	0.77	0.83	0.09	0.08	0.00	0.00	5.16	14.03	68.13	17.26	1.03	0.96	0.22	0.15	0.12	0.00	5.00
**South**	Quarter	1	0.00	85.45	20.60	1.27	1.01	0.18	0.00	0.00	0.00	6.58	0.00	75.50	21.97	2.30	1.37	0.67	0.00	0.00	0.00	6.28
		2	22.32	27.31	5.83	0.24	0.40	0.14	0.00	0.64	0.00	2.14	0.00	40.49	4.73	0.50	0.28	0.00	0.29	0.47	0.00	2.41
		3	36.57	64.86	11.98	0.51	0.80	0.00	0.00	0.00	0.00	4.80	27.70	57.61	6.90	1.01	0.14	0.13	0.31	0.00	0.00	3.58
		4	75.13	120.05	19.83	0.76	0.89	0.37	0.00	0.00	0.00	8.82	67.27	100.04	18.14	1.72	1.15	0.60	0.30	0.00	0.00	7.44
	Year	16/17	24.00	377.00	190.00	10.00	22.00	5.00	0.00	1.00	0.00	629.00	19.00	333.00	155.00	20.00	20.00	10.00	3.00	1.00	0.00	561.00
	Episodes	F/N	33.29	73.53	14.40	0.69	0.77	0.17	0.00	0.17	0.00	5.52	23.29	67.88	12.78	1.36	0.72	0.34	0.23	0.12	0.00	4.88
**London**	Quarter	1	0.00	63.84	38.41	4.09	1.18	0.00	0.00	0.00	0.00	9.57	0.00	55.78	35.45	3.27	0.99	0.55	0.00	0.00	0.00	8.19
		2	0.00	24.39	11.02	0.90	0.49	0.22	0.00	0.00	0.00	3.15	0.00	32.82	8.58	1.64	0.68	0.23	0.00	0.00	0.00	3.34
		3	56.21	57.54	26.59	1.88	0.76	0.47	0.00	1.91	0.00	7.78	8.14	54.82	24.76	1.74	0.60	0.26	0.00	1.47	0.00	6.54
		4	41.58	85.62	40.71	1.35	2.18	1.40	0.00	0.00	0.00	11.80	41.17	104.11	42.72	4.74	1.51	0.00	0.00	0.00	0.00	12.31
	Year	16/17	17.00	268.00	284.00	17.00	37.00	9.00	0.00	1.00	0.00	633.00	10.00	283.00	262.00	27.00	32.00	4.00	0.00	1.00	0.00	619.00
	Episodes	F/N	23.98	57.22	28.84	2.03	1.14	0.52	0.00	0.47	0.00	7.98	12.10	61.34	27.51	2.83	0.94	0.26	0.00	0.36	0.00	7.52
**Midlands And East**	Quarter	1	0.00	123.82	22.94	0.63	0.60	0.00	0.62	0.00	0.00	8.54	0.00	101.09	22.31	2.72	2.07	0.25	0.00	0.00	0.00	7.58
		2	14.34	28.12	3.54	1.58	0.76	0.00	0.00	0.00	0.00	2.10	0.00	39.65	7.94	0.00	1.02	0.19	0.51	0.00	0.00	2.94
		3	0.00	60.45	19.42	0.56	0.24	0.00	1.02	0.00	0.00	5.32	24.21	56.10	11.37	1.23	1.73	0.00	0.00	0.00	0.00	4.34
		4	92.23	142.00	25.59	1.10	1.26	0.88	0.00	0.00	0.00	10.96	95.28	157.05	19.73	1.23	2.83	0.44	0.00	0.00	0.00	10.52
	Year	16/17	13.00	248.00	136.00	7.00	11.00	4.00	3.00	0.00	0.00	422.00	13.00	243.00	110.00	8.00	29.00	4.00	1.00	0.00	0.00	408.00
	Episodes	F/N	26.41	87.46	17.60	0.98	0.71	0.22	0.40	0.00	0.00	6.64	29.31	87.55	15.20	1.27	1.89	0.22	0.13	0.00	0.00	6.28

Mean weekly incidence rate per 100,000 Persons.

Herpes Simplex (ICD10: B00)

		M										F									
		<1yr	1-4yrs	5-14yrs	15-24yrs	25-44yrs	45-64yrs	65-74yrs	75-84yrs	85+yrs	All Ages	<1yr	1-4yrs	5-14yrs	15-24yrs	25-44yrs	45-64yrs	65-74yrs	75-84yrs	85+yrs	All Ages
**4 weekly**	1	0.00	5.37	1.76	0.63	2.76	1.76	1.66	0.50	3.02	2.07	0.00	3.33	3.15	8.15	8.81	6.46	2.47	2.92	0.73	5.98
	2	0.00	4.41	2.11	1.78	3.01	2.00	3.01	0.98	1.51	2.40	0.00	1.40	1.65	10.33	11.14	5.47	1.50	2.23	1.38	6.38
	3	0.00	3.56	2.12	4.01	2.69	2.63	1.86	0.00	3.85	2.67	0.00	2.80	2.04	12.78	11.34	7.89	7.42	1.63	0.79	7.75
	4	17.22	3.53	2.21	1.44	4.32	1.25	0.72	1.88	0.00	2.39	0.00	4.04	2.75	11.69	10.22	7.25	5.09	4.89	1.60	7.33
	5	0.00	5.86	1.35	1.66	3.84	2.78	4.57	1.82	0.00	2.90	0.00	4.12	3.28	10.05	12.87	9.25	3.45	3.53	0.00	8.36
	6	0.00	3.27	3.41	1.79	3.32	2.45	1.13	1.33	0.00	2.55	10.81	10.93	3.90	10.86	10.42	7.15	4.39	3.30	0.00	7.60
	7	0.00	7.11	1.62	3.29	2.56	1.86	0.55	0.49	1.58	2.24	0.00	2.02	3.78	13.02	11.78	4.94	4.86	6.68	0.81	7.48
	8	0.00	4.04	2.49	3.60	3.47	1.58	2.09	0.00	0.00	2.51	0.00	4.75	3.27	11.02	11.90	5.70	7.96	2.86	2.92	7.68
	9	9.19	0.62	2.27	1.20	3.39	2.76	2.38	0.79	1.23	2.44	0.00	0.00	5.31	7.72	10.44	5.89	3.86	0.83	1.49	6.42
	10	0.00	5.17	4.69	6.35	4.30	2.30	0.79	3.56	1.23	3.69	0.00	6.08	5.76	13.03	11.83	6.50	2.71	0.75	2.62	7.73
	11	11.02	5.13	2.08	1.56	2.69	2.83	1.06	5.14	1.32	2.67	0.00	6.40	5.29	7.86	11.79	3.95	3.28	4.17	3.02	6.91
	12	7.21	7.77	3.37	1.89	2.88	1.43	1.49	0.88	0.00	2.57	7.58	2.69	4.95	12.07	12.27	4.95	5.08	0.69	0.71	7.56
	13	0.00	4.22	2.27	2.75	3.06	1.33	2.35	0.97	0.00	2.32	0.00	3.37	2.78	11.48	11.46	8.21	3.92	1.10	0.85	7.51
**Quarter**	1	0.00	4.51	1.98	2.02	2.82	2.10	2.14	0.49	2.81	2.35	0.00	2.57	2.35	10.24	10.30	6.59	3.69	2.31	0.95	6.65
	2	4.92	4.57	2.06	1.74	3.64	2.29	1.99	1.44	0.45	2.57	3.09	5.85	3.01	10.81	10.95	7.60	4.25	3.80	0.69	7.56
	3	2.83	3.62	3.09	3.49	3.54	2.02	1.38	1.32	0.76	2.71	0.00	2.77	4.92	11.02	11.92	5.80	5.13	2.93	2.16	7.49
	4	5.61	5.84	2.61	2.50	2.95	1.84	1.75	2.32	0.41	2.60	2.33	4.59	4.47	10.81	11.67	5.64	4.02	1.84	1.41	7.32
**Year**	16/17	6.00	83.00	112.00	119.00	353.00	205.00	60.00	28.00	9.00	975.00	3.00	70.00	161.00	536.00	1233.00	619.00	144.00	54.00	18.00	2838.00
**Episodes**	F/N	3.37	4.63	2.43	2.42	3.24	2.07	1.82	1.39	1.09	2.56	1.39	3.98	3.68	10.72	11.20	6.43	4.27	2.74	1.29	7.26

Mean weekly incidence rate per 100,000 Persons.

Herpes Simplex (ICD10: B00)

			M										F									
			<1yr	1-4yrs	5-14yrs	15-24yrs	25-44yrs	45-64yrs	65-74yrs	75-84yrs	85+yrs	All Ages	<1yr	1-4yrs	5-14yrs	15-24yrs	25-44yrs	45-64yrs	65-74yrs	75-84yrs	85+yrs	All Ages
**North**	Quarter	1	0.00	5.60	3.74	3.57	3.37	2.91	2.27	1.20	3.31	3.16	0.00	6.79	3.56	10.57	10.15	6.84	3.10	1.00	0.98	6.81
		2	0.00	11.14	2.93	2.25	2.60	2.40	0.92	1.16	1.81	2.62	0.00	7.87	3.36	10.96	10.85	7.15	3.26	4.34	1.86	7.49
		3	0.00	1.50	4.21	2.29	4.29	3.39	0.65	1.78	0.00	3.04	0.00	5.02	7.84	10.08	13.70	5.86	3.10	2.46	3.07	7.99
		4	0.00	7.59	2.11	2.26	2.52	1.86	1.95	3.06	0.00	2.40	4.56	8.30	4.37	11.49	11.38	5.34	5.18	0.99	2.08	7.37
	Year	16/17	0.00	34.00	43.00	44.00	103.00	85.00	18.00	12.00	3.00	342.00	1.00	34.00	61.00	193.00	358.00	199.00	48.00	18.00	8.00	920.00
	Episodes	F/N	0.00	6.54	3.24	2.59	3.18	2.63	1.44	1.79	1.29	2.80	1.12	7.01	4.75	10.78	11.51	6.32	3.65	2.24	1.99	7.42
**South**	Quarter	1	0.00	4.01	3.00	1.28	3.71	1.49	2.10	0.77	1.86	2.35	0.00	0.00	2.42	12.19	11.82	7.01	2.79	1.29	1.12	7.04
		2	19.68	3.94	2.06	1.24	3.97	2.64	0.63	1.71	0.00	2.49	12.36	1.67	2.11	14.01	10.74	7.16	2.79	2.43	0.90	7.18
		3	0.00	2.99	1.70	3.04	2.75	1.41	0.99	0.54	3.04	2.01	0.00	3.81	2.74	13.09	11.87	7.68	3.79	3.09	0.85	7.73
		4	4.82	1.37	2.98	4.36	3.06	1.36	2.85	1.18	1.62	2.55	0.00	2.96	6.17	11.54	12.37	7.47	2.02	0.85	3.56	7.65
	Year	16/17	2.00	15.00	32.00	39.00	96.00	52.00	19.00	7.00	4.00	266.00	1.00	11.00	43.00	192.00	337.00	216.00	35.00	15.00	7.00	857.00
	Episodes	F/N	6.38	3.09	2.43	2.46	3.38	1.74	1.62	1.06	1.60	2.35	3.26	2.10	3.34	12.73	11.68	7.33	2.84	1.92	1.59	7.40
**London**	Quarter	1	0.00	3.60	0.00	2.01	2.38	1.49	1.11	0.00	0.00	1.77	0.00	1.81	2.17	7.99	8.89	5.25	4.83	5.98	0.00	6.43
		2	0.00	3.19	0.80	1.73	4.19	2.27	4.89	0.00	0.00	2.90	0.00	5.85	4.01	7.03	11.59	7.88	7.96	6.81	0.00	8.55
		3	0.00	6.14	4.56	3.33	2.95	1.18	2.27	2.01	0.00	2.89	0.00	0.88	5.08	7.24	11.35	5.90	7.79	4.53	3.23	7.90
		4	8.87	5.98	4.36	0.00	3.66	2.81	0.00	1.92	0.00	3.11	4.77	4.30	2.09	8.93	11.99	4.71	3.73	4.64	0.00	7.75
	Year	16/17	2.00	22.00	24.00	15.00	106.00	33.00	8.00	2.00	0.00	212.00	1.00	15.00	32.00	73.00	372.00	95.00	26.00	15.00	1.00	630.00
	Episodes	F/N	2.18	4.70	2.40	1.77	3.31	1.94	2.12	0.97	0.00	2.67	1.17	3.26	3.35	7.78	10.97	5.97	6.11	5.52	0.79	7.67
**Midlands And East**	Quarter	1	0.00	4.85	1.18	1.24	1.81	2.51	3.07	0.00	6.07	2.14	0.00	1.68	1.23	10.22	10.35	7.27	4.05	0.97	1.70	6.32
		2	0.00	0.00	2.47	1.74	3.78	1.86	1.52	2.88	0.00	2.27	0.00	8.00	2.58	11.26	10.62	8.22	3.01	1.62	0.00	7.03
		3	11.31	3.84	1.90	5.30	4.18	2.10	1.60	0.97	0.00	2.89	0.00	1.35	4.02	13.67	10.77	3.76	5.84	1.65	1.50	6.35
		4	8.74	8.41	0.99	3.37	2.56	1.32	2.20	3.11	0.00	2.36	0.00	2.82	5.26	11.29	10.92	5.02	5.14	0.85	0.00	6.49
	Year	16/17	2.00	12.00	13.00	21.00	48.00	35.00	15.00	7.00	2.00	155.00	0.00	10.00	25.00	78.00	166.00	109.00	35.00	6.00	2.00	431.00
	Episodes	F/N	4.92	4.19	1.65	2.89	3.10	1.95	2.09	1.76	1.49	2.41	0.00	3.55	3.26	11.60	10.66	6.11	4.48	1.28	0.79	6.55

Mean weekly incidence rate per 100,000 Persons.

Herpes Zoster (ICD10: B02)

		M										F									
		<1yr	1-4yrs	5-14yrs	15-24yrs	25-44yrs	45-64yrs	65-74yrs	75-84yrs	85+yrs	All Ages	<1yr	1-4yrs	5-14yrs	15-24yrs	25-44yrs	45-64yrs	65-74yrs	75-84yrs	85+yrs	All Ages
**4 weekly**	1	0.00	0.00	1.18	1.49	3.17	5.28	7.73	17.95	19.23	4.65	0.00	1.82	0.77	3.82	4.75	8.01	10.96	14.63	19.88	6.58
	2	0.00	1.44	1.50	2.83	2.74	6.43	12.50	12.42	16.92	5.26	0.00	0.00	2.90	1.46	5.57	10.66	19.33	14.18	26.49	8.50
	3	0.00	0.79	2.69	2.85	3.40	6.20	13.30	23.05	31.91	6.43	0.00	2.31	2.99	2.43	5.06	9.77	15.41	19.63	31.88	8.26
	4	0.00	0.66	0.95	2.98	3.40	5.28	12.54	15.13	17.12	4.99	0.00	2.22	3.07	4.05	4.45	12.34	15.00	21.63	46.63	9.59
	5	0.00	2.98	2.52	1.66	5.48	6.37	10.27	13.21	21.23	5.80	0.00	2.98	2.23	2.48	4.91	12.63	13.95	19.61	33.12	8.69
	6	0.00	2.33	1.71	2.81	3.56	7.33	10.61	17.79	20.19	5.93	0.00	0.00	3.33	3.02	4.83	12.49	16.02	17.97	29.70	8.85
	7	0.00	1.32	2.71	3.96	3.99	5.05	12.48	11.27	13.54	5.27	0.00	0.71	2.66	2.30	4.84	12.42	19.93	14.79	32.08	8.94
	8	0.00	0.00	3.24	2.87	4.43	5.76	15.89	14.84	19.89	6.15	0.00	1.43	4.64	4.13	4.70	7.71	11.48	15.19	17.20	7.00
	9	0.00	0.59	4.03	1.37	3.25	5.07	8.50	12.87	17.70	4.55	0.00	1.33	3.60	0.91	4.04	12.29	14.28	11.56	19.77	7.68
	10	0.00	1.72	5.01	2.91	1.83	5.50	14.38	15.44	24.42	5.28	0.00	0.00	3.12	4.66	5.17	11.77	19.22	17.68	19.63	8.62
	11	0.00	1.78	2.16	4.57	2.96	6.30	11.20	9.89	15.47	5.26	0.00	1.29	4.33	4.33	5.63	11.32	12.69	18.88	31.60	8.83
	12	0.00	0.61	3.16	2.46	3.39	5.71	12.11	14.72	22.40	5.44	0.00	0.59	3.64	1.77	5.41	12.45	18.25	23.71	27.22	9.12
	13	0.00	0.00	2.51	2.53	2.33	5.84	12.60	19.64	22.47	5.21	0.00	0.00	3.20	2.07	4.99	10.01	16.67	22.31	27.72	7.84
**Quarter**	1	0.00	0.69	1.74	2.32	3.11	5.92	10.91	17.82	22.42	5.39	0.00	1.41	2.11	2.66	5.10	9.37	14.90	16.03	25.61	7.69
	2	0.00	1.89	1.91	2.88	4.23	6.03	11.47	14.56	18.98	5.57	0.00	1.69	2.86	3.20	4.71	12.44	15.66	18.55	35.75	8.98
	3	0.00	0.57	3.80	2.61	3.33	5.73	13.26	14.34	19.17	5.42	0.00	0.85	3.66	2.83	4.74	10.85	15.97	16.22	20.93	8.05
	4	0.00	1.08	2.75	2.94	2.77	5.68	11.49	14.53	20.21	5.15	0.00	0.58	3.66	2.86	5.25	11.27	15.63	20.21	28.17	8.48
**Year**	16/17	0.00	19.00	116.00	132.00	352.00	577.00	413.00	297.00	141.00	2047.00	0.00	19.00	134.00	141.00	536.00	1073.00	583.00	445.00	355.00	3286.00
**Episodes**	F/N	0.00	1.07	2.54	2.69	3.37	5.85	11.78	15.30	20.17	5.39	0.00	1.14	3.07	2.90	4.94	11.01	15.54	17.77	27.77	8.31

Mean weekly incidence rate per 100,000 Persons.

Herpes Zoster (ICD10: B02)

			M										F									
			<1yr	1-4yrs	5-14yrs	15-24yrs	25-44yrs	45-64yrs	65-74yrs	75-84yrs	85+yrs	All Ages	<1yr	1-4yrs	5-14yrs	15-24yrs	25-44yrs	45-64yrs	65-74yrs	75-84yrs	85+yrs	All Ages
**North**	Quarter	1	0.00	0.77	2.18	2.50	3.59	6.02	11.11	13.21	27.83	5.61	0.00	1.68	2.98	2.11	6.77	9.54	16.26	22.72	22.69	8.92
		2	0.00	2.20	1.17	3.59	4.15	6.34	9.93	17.38	30.67	6.04	0.00	1.59	3.65	2.40	5.04	13.43	16.55	19.59	38.12	9.76
		3	0.00	0.00	4.56	3.20	2.84	5.83	12.92	15.29	25.71	5.83	0.00	1.71	5.93	3.02	5.70	11.35	11.62	23.33	24.01	8.92
		4	0.00	0.76	3.91	2.87	2.20	5.46	14.03	15.71	20.77	5.50	0.00	0.84	3.75	3.39	4.54	12.41	13.94	22.30	31.75	9.09
	Year	16/17	0.00	5.00	39.00	52.00	104.00	191.00	150.00	103.00	59.00	703.00	0.00	7.00	52.00	49.00	171.00	368.00	192.00	178.00	120.00	1,137.00
	Episodes	F/N	0.00	0.96	2.92	3.05	3.21	5.92	11.96	15.43	26.33	5.75	0.00	1.45	4.07	2.72	5.50	11.71	14.63	21.94	29.31	9.18
**South**	Quarter	1	0.00	1.97	0.73	3.86	2.55	6.09	8.29	26.76	11.74	5.60	0.00	2.14	2.73	3.65	4.92	10.97	15.82	18.70	28.14	8.96
		2	0.00	1.47	3.37	2.95	3.24	6.93	15.58	12.54	7.61	6.01	0.00	0.00	2.41	2.64	5.46	14.70	16.27	18.80	44.52	10.43
		3	0.00	2.27	4.70	2.04	3.85	6.02	11.88	11.57	12.11	5.62	0.00	1.68	2.99	3.68	3.93	10.75	17.77	18.63	26.93	8.71
		4	0.00	2.17	2.46	2.94	2.69	6.83	10.32	15.26	24.70	5.68	0.00	1.48	5.00	3.21	6.14	11.23	17.68	27.83	25.09	10.05
	Year	16/17	0.00	10.00	39.00	44.00	90.00	193.00	137.00	101.00	35.00	649.00	0.00	6.00	42.00	49.00	148.00	353.00	209.00	161.00	136.00	1,104.00
	Episodes	F/N	0.00	1.96	2.82	2.95	3.09	6.47	11.59	16.46	13.92	5.73	0.00	1.30	3.27	3.28	5.12	11.97	16.87	20.95	31.42	9.56
**London**	Quarter	1	0.00	0.00	1.70	1.04	2.65	3.81	7.63	11.97	35.06	3.29	0.00	1.83	0.88	2.84	4.85	8.42	13.62	9.13	32.62	5.78
		2	0.00	0.00	0.75	1.33	3.83	4.93	12.02	10.90	19.60	3.92	0.00	0.81	1.61	2.02	3.53	10.22	14.90	15.16	25.55	5.66
		3	0.00	0.00	2.51	0.94	2.21	5.07	18.84	9.75	5.30	3.59	0.00	0.00	2.20	1.77	3.98	7.65	19.34	6.16	6.31	4.88
		4	0.00	0.00	1.60	0.91	2.82	3.02	11.76	13.61	5.53	3.01	0.00	0.00	2.08	2.47	3.97	9.88	16.93	19.33	25.73	5.92
	Year	16/17	0.00	0.00	16.00	9.00	92.00	71.00	47.00	24.00	13.00	272.00	0.00	3.00	16.00	21.00	137.00	145.00	69.00	34.00	30.00	455.00
	Episodes	F/N	0.00	0.00	1.63	1.06	2.89	4.22	12.55	11.54	16.43	3.46	0.00	0.66	1.69	2.27	4.07	9.07	16.17	12.50	22.61	5.56
**Midlands And East**	Quarter	1	0.00	0.00	2.36	1.89	3.63	7.77	16.62	19.33	15.05	7.04	0.00	0.00	1.84	2.06	3.85	8.53	13.92	13.57	18.97	7.09
		2	0.00	3.90	2.36	3.64	5.69	5.92	8.36	17.44	18.04	6.33	0.00	4.35	3.78	5.76	4.80	11.40	14.90	20.65	34.80	10.07
		3	0.00	0.00	3.43	4.28	4.42	6.01	9.39	20.75	33.56	6.67	0.00	0.00	3.54	2.87	5.37	13.64	15.15	16.76	26.48	9.68
		4	0.00	1.39	3.04	5.05	3.36	7.44	9.85	13.54	29.86	6.41	0.00	0.00	3.79	2.35	6.36	11.55	13.94	11.37	30.11	8.87
	Year	16/17	0.00	4.00	22.00	27.00	66.00	122.00	79.00	69.00	34.00	423.00	0.00	3.00	24.00	22.00	80.00	207.00	113.00	72.00	69.00	590.00
	Episodes	F/N	0.00	1.37	2.79	3.71	4.30	6.77	11.00	17.76	24.01	6.61	0.00	1.15	3.25	3.31	5.09	11.28	14.49	15.68	27.73	8.95

Mean weekly incidence rate per 100,000 Persons.

Infections of Skin & Subcutaneous Tissue (ICD10: L00-L08)

		M										F									
		<1yr	1-4yrs	5-14yrs	15-24yrs	25-44yrs	45-64yrs	65-74yrs	75-84yrs	85+yrs	All Ages	<1yr	1-4yrs	5-14yrs	15-24yrs	25-44yrs	45-64yrs	65-74yrs	75-84yrs	85+yrs	All Ages
**4 weekly**	1	0.00	52.25	32.53	29.14	31.97	38.23	53.63	86.64	124.12	40.47	0.00	52.93	40.07	34.08	40.96	48.38	55.02	88.58	160.63	50.16
	2	0.00	74.79	53.89	44.59	43.83	51.55	83.82	113.55	179.52	57.49	0.00	63.56	57.66	47.32	60.06	69.08	82.43	130.06	204.17	70.59
	3	397.69	78.63	62.38	46.14	50.95	63.11	101.89	117.55	173.19	66.23	486.01	71.34	67.94	60.44	68.41	82.84	107.53	127.19	232.39	83.74
	4	162.03	81.55	54.07	43.81	48.81	60.98	86.81	112.31	139.99	62.00	399.24	64.08	56.36	66.96	72.14	78.32	85.24	133.66	197.38	79.76
	5	70.04	72.29	57.54	48.78	47.69	47.84	72.67	106.71	182.17	57.51	296.19	62.31	68.12	57.55	63.82	75.35	85.01	125.40	213.46	76.07
	6	103.53	80.88	53.50	41.24	40.22	46.97	68.83	87.89	167.21	53.43	116.83	70.70	45.73	52.20	57.00	65.60	72.78	109.80	162.91	65.24
	7	103.21	74.93	46.93	33.90	40.65	47.10	55.76	89.06	131.93	48.79	174.54	67.16	54.44	45.51	41.95	51.51	61.04	93.15	172.41	56.18
	8	111.84	86.95	45.18	32.55	36.22	37.28	52.40	92.53	147.41	45.44	91.88	80.33	49.49	44.52	38.31	46.22	62.05	96.62	184.74	54.02
	9	96.67	66.28	43.39	24.43	29.41	33.54	50.62	69.01	110.88	39.14	98.36	59.93	47.31	30.02	38.20	39.80	50.59	74.24	156.36	46.50
	10	101.30	75.88	51.05	35.14	35.16	40.96	57.85	86.76	127.71	46.29	99.75	81.74	50.23	45.01	48.41	48.56	56.36	77.30	193.00	55.88
	11	67.14	94.70	41.37	37.01	34.37	36.82	60.85	80.71	127.28	45.45	50.47	66.44	51.97	40.03	45.72	46.46	57.72	83.55	135.43	52.26
	12	67.51	80.13	43.98	38.55	36.85	42.78	64.90	94.76	140.74	48.29	106.90	61.30	46.54	42.64	45.02	47.10	70.72	100.59	174.90	55.57
	13	102.46	61.86	46.32	31.39	34.12	37.04	50.68	91.27	203.42	44.56	56.85	69.08	44.87	32.92	41.91	41.66	62.31	89.58	164.55	50.33
**Quarter**	1	122.36	67.30	48.29	39.12	41.46	49.99	77.77	104.43	156.26	53.64	149.54	61.87	54.06	46.27	55.28	65.35	79.61	113.22	196.11	66.78
	2	102.55	76.24	53.39	43.18	45.47	51.02	73.89	101.92	156.28	56.42	251.01	64.98	56.52	56.69	61.07	70.09	76.89	119.81	190.18	71.10
	3	118.47	77.07	48.29	31.04	34.52	39.43	54.00	83.81	133.91	44.92	115.13	73.75	50.66	41.49	41.64	45.41	58.58	84.40	176.28	52.92
	4	74.39	79.42	42.94	35.31	34.51	38.33	57.61	86.74	152.78	45.37	73.32	66.45	46.95	38.19	44.13	45.44	62.87	89.31	158.95	52.61
**Year**	16/17	159.00	1330.00	2132.00	1755.00	4094.00	4338.00	2285.00	1760.00	1026.00	18879.00	168.00	1132.00	2164.00	2192.00	5392.00	5361.00	2559.00	2266.00	2182.00	23416.00
**Episodes**	F/N	104.41	75.03	48.32	37.28	39.11	44.81	65.97	94.37	149.93	50.20	149.21	66.73	52.13	45.87	50.73	56.83	69.63	102.03	180.56	61.05

Mean weekly incidence rate per 100,000 Persons.

Infections of Skin & Subcutaneous Tissue (ICD10: L00-L08)

			M										F									
			<1yr	1-4yrs	5-14yrs	15-24yrs	25-44yrs	45-64yrs	65-74yrs	75-84yrs	85+yrs	All Ages	<1yr	1-4yrs	5-14yrs	15-24yrs	25-44yrs	45-64yrs	65-74yrs	75-84yrs	85+yrs	All Ages
**North**	Quarter	1	98.18	69.92	47.90	31.26	40.16	47.37	65.72	84.80	159.55	50.58	122.70	51.87	37.64	38.86	59.55	63.75	76.18	99.59	195.51	64.55
		2	88.03	80.97	55.54	36.43	47.72	52.50	77.79	101.06	156.78	57.83	256.99	68.06	59.30	47.23	64.98	68.81	74.09	94.98	167.06	69.65
		3	106.24	76.82	56.51	31.25	36.13	44.80	55.92	88.55	120.35	48.32	107.08	75.95	60.81	44.82	48.65	46.62	63.50	78.73	183.89	57.92
		4	77.30	71.71	44.33	39.74	34.16	38.01	61.32	82.55	142.96	46.24	76.78	66.57	47.56	34.28	47.86	47.62	58.02	92.16	163.08	54.18
	Year	16/17	44.00	389.00	676.00	595.00	1,288.00	1,473.00	817.00	597.00	327.00	6,206.00	49.00	320.00	656.00	743.00	1,717.00	1,789.00	899.00	739.00	725.00	7,637.00
	Episodes	F/N	92.35	74.97	51.15	34.71	39.70	45.80	65.42	89.46	145.13	50.87	143.08	65.66	51.48	41.41	55.44	56.93	68.06	91.44	177.19	61.73
**South**	Quarter	1	0.00	74.72	49.75	36.24	42.57	57.26	79.36	106.35	166.23	58.02	0.00	67.03	64.56	52.03	58.63	67.97	70.59	116.91	205.29	72.04
		2	173.17	76.35	53.94	45.67	43.42	50.23	73.08	94.26	142.82	56.65	160.92	57.65	47.75	59.47	61.77	71.94	67.27	122.17	188.91	72.04
		3	198.69	84.40	45.07	33.37	34.58	40.42	57.41	94.72	135.63	48.09	95.53	89.59	54.46	48.09	38.17	43.95	57.89	78.27	163.22	54.57
		4	85.08	86.52	46.80	35.39	32.91	36.74	52.83	87.94	157.26	46.38	57.77	65.16	46.40	35.67	44.27	48.11	64.73	83.19	148.81	53.90
	Year	16/17	59.00	408.00	649.00	568.00	1,094.00	1,344.00	751.00	599.00	367.00	5,839.00	35.00	342.00	655.00	725.00	1,442.00	1,680.00	797.00	749.00	761.00	7,186.00
	Episodes	F/N	115.35	80.42	48.98	37.82	38.47	46.24	65.81	95.79	150.34	52.37	80.11	69.63	53.19	49.02	50.92	58.26	65.16	100.55	176.79	63.31
**London**	Quarter	1	310.31	61.82	40.95	42.94	31.32	41.75	92.79	115.16	121.35	44.25	338.11	61.04	50.92	42.31	45.53	60.83	89.91	129.81	197.70	57.74
		2	81.81	71.24	47.58	35.60	39.73	44.89	73.52	98.70	165.76	47.80	268.75	59.92	49.80	46.64	50.85	68.99	83.10	139.78	204.55	62.20
		3	85.86	63.20	40.97	26.88	25.79	34.70	56.18	85.05	141.89	36.42	127.92	52.30	30.94	34.78	31.77	47.26	60.19	94.41	178.37	42.63
		4	81.66	61.26	37.60	32.13	27.54	35.26	53.62	99.63	161.23	37.70	88.16	52.62	37.77	39.41	35.78	44.87	58.61	89.85	161.68	44.38
	Year	16/17	39.00	300.00	411.00	293.00	995.00	662.00	258.00	207.00	114.00	3,279.00	56.00	259.00	401.00	380.00	1,386.00	887.00	310.00	309.00	245.00	4,233.00
	Episodes	F/N	138.81	64.51	41.88	34.41	31.26	39.26	69.11	99.62	147.90	41.66	206.92	56.54	42.50	40.90	41.17	55.74	73.15	113.96	185.93	51.93
**Midlands And East**	Quarter	1	80.97	62.75	54.56	46.05	51.80	53.57	73.20	111.43	177.92	61.70	137.36	67.52	63.11	51.86	57.42	68.87	81.75	106.58	185.93	72.77
		2	67.18	76.41	56.49	55.04	50.99	56.45	71.19	113.65	159.78	63.39	317.41	74.27	69.23	73.41	66.68	70.63	83.08	122.30	200.22	80.50
		3	83.11	83.85	50.60	32.68	41.59	37.82	46.50	66.92	137.79	46.85	129.97	77.17	56.45	38.26	47.98	43.81	52.75	86.19	179.64	56.55
		4	53.52	98.20	43.01	33.97	43.44	43.32	62.66	76.85	149.67	51.16	70.57	81.43	56.07	43.39	48.59	41.15	70.12	92.04	162.21	58.00
	Year	16/17	17.00	233.00	396.00	299.00	717.00	859.00	459.00	357.00	218.00	3,555.00	28.00	211.00	452.00	344.00	847.00	1,005.00	553.00	469.00	451.00	4,360.00
	Episodes	F/N	71.12	80.23	51.27	42.18	47.03	47.95	63.54	92.62	156.35	55.92	166.72	75.08	61.37	52.14	55.38	56.39	72.13	102.17	182.35	67.21

Mean weekly incidence rate per 100,000 Persons.

Scabies (ICD10: B86)

		M										F									
		<1yr	1-4yrs	5-14yrs	15-24yrs	25-44yrs	45-64yrs	65-74yrs	75-84yrs	85+yrs	All Ages	<1yr	1-4yrs	5-14yrs	15-24yrs	25-44yrs	45-64yrs	65-74yrs	75-84yrs	85+yrs	All Ages
**4 weekly**	1	0.00	1.65	1.15	2.33	0.85	1.07	0.89	0.00	0.00	1.10	0.00	0.64	1.87	2.09	1.06	0.90	0.89	0.43	0.00	1.15
	2	0.00	0.72	1.02	2.30	0.98	0.40	0.00	1.25	0.00	0.87	0.00	1.37	1.80	3.09	0.80	1.30	0.24	0.54	2.46	1.28
	3	0.00	3.39	1.31	2.84	0.72	1.56	1.38	0.00	0.00	1.35	0.00	1.38	2.16	0.72	1.90	1.00	0.00	0.00	0.81	1.20
	4	0.00	0.00	1.88	2.75	0.65	1.13	0.57	1.46	5.63	1.31	0.00	3.62	1.68	3.87	2.01	1.51	0.75	2.95	0.88	1.99
	5	11.66	0.63	1.51	1.62	0.83	1.15	1.01	0.50	2.27	1.13	0.00	1.37	3.98	1.77	1.06	1.04	0.69	0.00	0.00	1.37
	6	0.00	1.44	2.44	2.61	0.85	1.09	0.55	0.50	3.56	1.38	0.00	0.76	1.04	2.93	1.41	1.08	0.40	2.22	2.38	1.52
	7	6.97	2.29	2.87	3.33	1.16	1.35	0.68	2.01	0.00	1.75	0.00	4.14	2.70	2.40	2.05	2.41	2.19	0.40	0.00	2.17
	8	0.00	0.65	1.33	4.14	0.61	1.04	0.00	0.57	0.00	1.14	0.00	1.42	3.10	3.42	1.73	1.26	1.38	0.00	0.00	1.85
	9	0.00	2.64	2.59	3.60	1.15	0.37	0.68	0.00	0.00	1.39	0.00	2.00	2.10	1.45	2.12	1.32	0.39	1.20	0.00	1.52
	10	0.00	3.44	1.46	4.98	1.74	0.96	0.70	0.53	0.00	1.85	9.76	3.36	3.66	4.30	1.83	1.90	1.10	0.00	0.63	2.29
	11	0.00	0.00	0.72	3.63	1.55	0.87	1.83	0.00	1.51	1.39	0.00	0.73	1.09	3.34	1.36	1.17	0.91	1.49	3.49	1.50
	12	0.00	0.64	0.95	2.18	0.91	0.97	0.26	1.70	1.51	1.08	4.10	2.72	2.20	3.64	1.70	0.99	0.00	3.52	0.84	1.75
	13	0.00	0.00	1.31	5.16	0.70	0.49	1.32	0.49	0.00	1.30	0.00	1.24	0.83	4.13	0.92	0.31	1.24	1.21	1.50	1.15
**Quarter**	1	0.00	1.90	1.16	2.48	0.85	1.01	0.77	0.38	0.00	1.11	0.00	1.09	1.94	1.98	1.24	1.05	0.42	0.33	1.00	1.21
	2	3.33	0.59	1.85	2.23	0.88	1.15	0.61	1.14	3.27	1.26	0.00	2.02	2.51	2.45	1.49	1.43	0.94	1.48	0.93	1.67
	3	2.14	1.90	2.34	4.57	1.10	0.94	0.64	0.33	0.00	1.61	3.00	2.77	2.44	3.28	2.00	1.51	0.77	0.49	0.19	1.90
	4	0.00	1.07	0.92	3.49	1.07	0.72	1.05	0.84	0.93	1.25	1.26	1.63	1.73	3.70	1.38	0.95	1.00	1.91	1.79	1.57
**Year**	16/17	2.00	23.00	74.00	162.00	110.00	90.00	25.00	13.00	7.00	506.00	3.00	35.00	90.00	156.00	170.00	116.00	28.00	19.00	12.00	629.00
**Episodes**	F/N	1.41	1.35	1.57	3.17	0.97	0.96	0.76	0.68	1.09	1.31	1.05	1.88	2.16	2.84	1.53	1.24	0.79	1.06	0.98	1.59

Mean weekly incidence rate per 100,000 Persons.

Scabies (ICD10: B86)

			M										F									
			<1yr	1-4yrs	5-14yrs	15-24yrs	25-44yrs	45-64yrs	65-74yrs	75-84yrs	85+yrs	All Ages	<1yr	1-4yrs	5-14yrs	15-24yrs	25-44yrs	45-64yrs	65-74yrs	75-84yrs	85+yrs	All Ages
**North**	Quarter	1	0.00	0.77	2.19	2.26	1.17	1.14	0.33	0.00	0.00	1.22	0.00	2.50	2.65	4.45	1.71	1.42	0.30	0.00	0.99	1.82
		2	13.33	0.72	3.85	4.26	1.08	0.84	0.94	0.57	1.70	1.75	0.00	4.75	3.97	4.75	1.98	2.21	0.31	0.47	1.90	2.47
		3	0.00	3.84	3.93	6.13	1.23	1.51	0.64	0.60	0.00	2.28	5.69	5.76	1.90	4.92	2.31	1.01	0.61	0.50	0.00	2.12
		4	0.00	0.79	2.40	6.53	1.54	1.23	0.64	0.66	3.72	2.13	5.04	2.45	3.16	6.04	2.08	1.13	0.00	1.00	3.09	2.31
	Year	16/17	1.00	8.00	41.00	84.00	40.00	38.00	8.00	3.00	3.00	226.00	2.00	19.00	37.00	90.00	63.00	46.00	4.00	4.00	6.00	271.00
	Episodes	F/N	3.52	1.51	3.10	4.79	1.25	1.17	0.64	0.46	1.36	1.84	2.63	3.88	2.94	5.04	2.02	1.46	0.30	0.49	1.50	2.18
**South**	Quarter	1	0.00	0.00	0.00	2.24	0.84	0.16	0.42	1.53	0.00	0.69	0.00	0.00	0.79	2.29	0.50	1.49	0.41	1.33	0.00	1.02
		2	0.00	0.80	2.70	0.74	0.26	0.51	0.99	1.25	1.45	0.86	0.00	0.74	1.54	1.23	1.65	1.05	0.67	1.10	1.82	1.26
		3	0.00	0.78	1.72	3.28	0.53	0.40	0.33	0.71	0.00	0.99	0.00	0.00	0.91	3.00	1.30	0.63	1.04	0.00	0.78	1.11
		4	0.00	0.71	0.27	2.69	0.77	0.72	0.33	0.60	0.00	0.87	0.00	1.45	0.61	2.18	1.02	0.50	1.50	0.50	0.80	1.01
	Year	16/17	0.00	3.00	16.00	34.00	17.00	14.00	6.00	6.00	1.00	97.00	0.00	3.00	12.00	33.00	33.00	26.00	11.00	5.00	4.00	127.00
	Episodes	F/N	0.00	0.58	1.20	2.21	0.59	0.45	0.53	1.03	0.38	0.85	0.00	0.55	0.97	2.16	1.13	0.92	0.90	0.74	0.87	1.10
**London**	Quarter	1	0.00	3.60	1.27	3.54	0.80	2.50	1.10	0.00	0.00	1.67	0.00	1.88	1.86	0.47	1.25	0.78	0.96	0.00	3.02	1.15
		2	0.00	0.85	0.41	1.83	1.45	2.25	0.00	1.74	4.73	1.47	0.00	0.87	3.55	2.51	1.84	1.67	0.89	2.79	0.00	1.97
		3	8.58	1.68	1.25	1.89	1.67	1.24	0.00	0.00	0.00	1.45	6.32	5.32	3.90	3.47	1.95	2.05	0.00	1.48	0.00	2.45
		4	0.00	0.00	0.00	1.37	1.22	0.71	2.12	0.00	0.00	0.89	0.00	2.60	2.06	1.66	1.16	1.76	0.93	4.42	3.28	1.63
	Year	16/17	1.00	7.00	7.00	18.00	41.00	28.00	3.00	1.00	1.00	107.00	1.00	12.00	27.00	19.00	52.00	25.00	3.00	6.00	2.00	147.00
	Episodes	F/N	2.10	1.52	0.73	2.15	1.29	1.69	0.79	0.46	1.25	1.37	1.55	2.63	2.86	2.04	1.56	1.57	0.70	2.18	1.55	1.81
**Midlands And East**	Quarter	1	0.00	3.22	1.17	1.88	0.61	0.25	1.22	0.00	0.00	0.85	0.00	0.00	2.46	0.69	1.49	0.50	0.00	0.00	0.00	0.83
		2	0.00	0.00	0.44	2.09	0.74	1.01	0.51	0.99	5.21	0.99	0.00	1.72	1.00	1.30	0.49	0.78	1.88	1.54	0.00	0.98
		3	0.00	1.30	2.45	6.97	0.99	0.62	1.57	0.00	0.00	1.72	0.00	0.00	3.06	1.73	2.42	2.33	1.45	0.00	0.00	1.91
		4	0.00	2.78	1.00	3.38	0.75	0.21	1.09	2.10	0.00	1.11	0.00	0.00	1.10	4.90	1.27	0.42	1.56	1.74	0.00	1.34
	Year	16/17	0.00	5.00	10.00	26.00	12.00	10.00	8.00	3.00	2.00	76.00	0.00	1.00	14.00	14.00	22.00	19.00	10.00	4.00	0.00	84.00
	Episodes	F/N	0.00	1.79	1.25	3.55	0.77	0.53	1.09	0.78	1.38	1.16	0.00	0.45	1.89	2.14	1.40	1.00	1.24	0.83	0.00	1.26

Mean weekly incidence rate per 100,000 Persons.

Symptoms and signs involving the skin and subcutaneous tissue (ICD10: R20-R23)

		M										F									
		<1yr	1-4yrs	5-14yrs	15-24yrs	25-44yrs	45-64yrs	65-74yrs	75-84yrs	85+yrs	All Ages	<1yr	1-4yrs	5-14yrs	15-24yrs	25-44yrs	45-64yrs	65-74yrs	75-84yrs	85+yrs	All Ages
**4 weekly**	1	0.00	94.17	35.27	16.61	17.66	23.85	38.82	52.97	91.71	29.81	0.00	98.53	42.80	31.83	35.57	38.08	49.34	49.03	67.34	42.51
	2	255.10	135.31	47.67	18.12	23.73	34.02	50.38	54.22	99.61	38.99	109.65	120.47	50.04	33.72	43.79	44.71	43.91	61.50	109.50	50.31
	3	618.72	153.09	50.10	23.24	27.57	36.89	51.05	60.69	95.80	43.25	534.25	122.98	51.55	40.05	45.58	56.07	66.35	68.61	123.87	57.56
	4	320.97	98.23	33.23	16.39	21.85	29.99	45.41	66.56	107.70	34.42	312.36	112.46	37.00	40.67	43.60	46.53	53.44	73.23	103.21	51.80
	5	140.31	128.27	39.44	22.05	25.32	30.17	45.88	56.08	110.66	38.02	243.65	105.80	44.34	42.20	43.07	50.32	53.18	66.42	84.91	51.82
	6	262.70	114.11	37.01	22.36	22.79	32.93	57.24	57.32	60.14	37.26	189.39	128.32	43.85	38.05	47.51	47.50	44.96	76.99	96.43	52.70
	7	167.21	118.68	32.86	21.59	22.03	25.64	49.30	60.69	75.93	34.73	211.79	105.27	43.37	42.33	36.55	41.80	44.86	62.62	83.64	46.60
	8	131.87	91.46	35.74	18.40	16.97	28.13	41.45	48.32	53.46	30.81	170.69	93.24	33.79	31.44	35.01	39.38	46.96	53.93	76.48	42.26
	9	124.33	80.95	31.17	14.16	17.12	25.09	36.27	41.87	86.86	28.17	225.88	73.22	29.51	27.71	27.61	33.61	40.97	38.42	61.40	34.92
	10	185.58	89.79	36.87	15.31	20.94	31.87	41.46	63.11	73.72	33.91	186.70	96.00	46.03	39.09	35.55	42.55	48.60	69.81	74.07	46.69
	11	294.44	117.69	37.90	19.79	20.27	30.63	48.89	49.24	53.21	36.13	142.07	107.98	46.03	37.01	36.26	36.75	36.38	53.37	84.19	43.62
	12	172.26	109.93	44.15	18.60	20.67	30.44	41.77	51.63	88.50	35.79	247.44	106.65	42.38	33.89	38.08	38.11	46.57	59.18	81.68	45.91
	13	143.52	96.18	39.49	20.96	18.93	25.33	46.64	60.95	57.04	32.96	166.99	104.87	42.34	30.87	39.23	37.56	42.75	55.51	81.22	44.70
**Quarter**	1	268.87	124.96	43.65	19.11	22.58	30.99	46.14	55.73	95.40	36.77	198.12	112.80	47.72	34.94	41.18	45.66	52.90	58.89	97.71	49.54
	2	229.16	113.72	35.66	20.93	23.19	30.60	50.03	58.78	92.45	36.36	247.70	112.65	42.37	40.94	43.15	47.64	50.23	69.84	93.09	51.38
	3	153.99	91.71	34.94	16.47	18.48	27.67	40.45	55.33	70.53	31.46	195.72	92.79	36.64	33.82	34.53	38.67	45.90	58.10	73.15	42.46
	4	197.12	107.45	39.85	19.34	20.20	28.68	45.46	52.37	65.98	34.67	182.74	103.99	43.47	33.86	36.68	37.44	41.13	54.21	81.13	44.06
**Year**	16/17	305.00	1963.00	1687.00	913.00	2330.00	2783.00	1493.00	1017.00	532.00	13023.00	308.00	1835.00	1784.00	1724.00	4301.00	3897.00	1704.00	1345.00	1028.00	17926.00
**Episodes**	F/N	212.60	109.54	38.47	19.00	21.15	29.51	45.60	55.61	81.30	34.84	206.86	105.69	42.55	35.99	38.97	42.45	47.59	60.44	86.40	46.95

Mean weekly incidence rate per 100,000 Persons.

Symptoms and signs involving the skin and subcutaneous tissue (ICD10: R20-R23)

			M										F									
			<1yr	1-4yrs	5-14yrs	15-24yrs	25-44yrs	45-64yrs	65-74yrs	75-84yrs	85+yrs	All Ages	<1yr	1-4yrs	5-14yrs	15-24yrs	25-44yrs	45-64yrs	65-74yrs	75-84yrs	85+yrs	All Ages
**North**	Quarter	1	577.72	136.12	45.85	21.82	23.11	28.06	42.52	63.52	84.63	37.18	233.29	121.22	52.83	31.63	39.08	41.50	46.30	59.04	77.46	46.87
		2	124.77	114.27	37.57	21.87	24.89	30.15	42.08	53.69	68.82	35.49	364.01	121.70	42.95	36.40	40.83	47.39	52.11	61.80	101.86	50.58
		3	109.33	101.76	33.87	17.82	17.24	28.50	41.31	42.95	49.23	30.53	196.98	95.45	43.96	32.59	35.86	39.28	43.34	48.26	63.27	42.57
		4	246.22	109.65	33.55	22.74	19.66	24.97	44.06	49.76	88.93	33.73	195.15	108.64	45.12	33.75	34.58	36.51	38.13	53.74	80.85	43.12
	Year	16/17	88.00	596.00	497.00	361.00	689.00	900.00	531.00	351.00	164.00	4,177.00	98.00	544.00	585.00	608.00	1,168.00	1,299.00	594.00	451.00	331.00	5,678.00
	Episodes	F/N	261.87	115.43	37.71	21.08	21.29	27.96	42.48	52.50	72.83	34.26	249.56	111.94	46.16	33.64	37.65	41.29	45.10	55.82	81.25	45.87
**South**	Quarter	1	453.55	125.43	37.76	19.32	20.01	30.86	45.93	61.15	87.13	36.14	256.41	109.10	42.74	30.90	37.28	48.00	53.47	58.27	98.57	48.35
		2	221.28	114.71	30.75	16.85	16.40	23.40	41.57	54.70	76.04	30.94	151.05	94.67	31.49	30.20	36.47	37.92	43.20	64.23	94.60	42.99
		3	153.79	79.53	26.50	12.23	14.76	24.06	32.25	44.12	77.60	26.66	197.33	83.71	27.34	33.56	31.96	29.96	32.51	54.99	76.97	37.43
		4	101.28	89.67	34.93	14.25	19.54	28.01	34.03	44.03	64.75	30.40	150.76	86.69	34.96	30.62	31.01	31.72	40.19	53.81	75.46	38.89
	Year	16/17	64.00	507.00	429.00	234.00	506.00	778.00	442.00	315.00	184.00	3,459.00	73.00	451.00	420.00	472.00	978.00	1,064.00	515.00	440.00	374.00	4,787.00
	Episodes	F/N	232.26	102.57	32.45	15.68	17.65	26.52	38.50	51.07	76.37	31.03	188.17	93.56	34.08	31.30	34.22	36.92	42.36	57.94	86.55	41.94
**London**	Quarter	1	44.21	138.92	58.12	22.75	25.21	40.26	57.30	55.12	128.63	42.63	165.43	148.17	63.16	48.58	51.37	52.03	68.97	67.83	129.05	61.01
		2	381.47	126.50	55.58	30.72	31.82	41.72	74.83	66.23	137.92	47.50	229.09	148.26	59.33	56.29	54.71	63.56	55.87	84.93	98.53	64.75
		3	234.09	101.73	48.13	23.65	24.80	34.10	51.58	66.92	80.97	38.50	300.64	109.58	47.58	32.53	39.52	52.92	61.64	66.73	92.26	50.75
		4	254.25	140.94	54.54	26.84	22.94	36.34	63.60	62.45	49.93	42.58	213.44	112.83	50.39	42.71	42.67	49.91	45.29	64.79	87.52	52.02
	Year	16/17	113.00	592.00	531.00	224.00	839.00	645.00	233.00	131.00	78.00	3,386.00	101.00	594.00	522.00	419.00	1,589.00	872.00	246.00	194.00	134.00	4,671.00
	Episodes	F/N	231.39	127.01	54.12	26.08	26.30	38.17	62.07	62.75	100.09	42.89	227.19	130.06	55.20	45.24	47.21	54.77	57.90	71.33	101.78	57.28
**Midlands And East**	Quarter	1	0.00	99.37	32.86	12.57	21.98	24.78	38.80	43.11	81.22	31.13	137.36	72.73	32.15	28.65	36.99	41.10	42.87	50.44	85.75	41.94
		2	189.13	99.39	18.72	14.28	19.65	27.11	41.64	60.50	87.03	31.53	246.66	85.97	35.70	40.87	40.61	41.68	49.75	68.42	77.38	47.21
		3	118.75	83.82	31.24	12.16	17.12	24.02	36.67	67.34	74.31	30.17	87.94	82.43	27.68	36.61	30.80	32.53	46.10	62.43	60.10	39.07
		4	186.72	89.55	36.38	13.52	18.69	25.39	40.14	53.22	60.31	31.95	171.61	107.81	43.39	28.37	38.44	31.63	40.92	44.51	80.69	42.23
	Year	16/17	40.00	268.00	230.00	94.00	296.00	460.00	287.00	220.00	106.00	2,001.00	36.00	246.00	257.00	225.00	566.00	662.00	349.00	260.00	189.00	2,790.00
	Episodes	F/N	124.89	93.15	29.59	13.16	19.36	25.36	39.36	56.13	75.93	31.20	162.51	87.21	34.75	33.76	36.78	36.83	45.00	56.67	76.01	42.70

Mean weekly incidence rate per 100,000 Persons.

Impetigo (ICD10 : L01)

		M										F									
		<1yr	1-4yrs	5-14yrs	15-24yrs	25-44yrs	45-64yrs	65-74yrs	75-84yrs	85+yrs	All Ages	<1yr	1-4yrs	5-14yrs	15-24yrs	25-44yrs	45-64yrs	65-74yrs	75-84yrs	85+yrs	All Ages
**4 weekly**	1	0.00	11.00	12.08	2.63	1.44	0.63	2.49	0.00	6.12	3.17	0.00	18.13	12.26	4.48	3.17	2.51	0.97	1.38	5.10	4.55
	2	0.00	20.21	17.02	4.17	1.83	1.06	1.11	1.71	0.00	4.54	0.00	23.52	16.16	3.94	3.65	3.19	1.91	2.13	1.55	5.50
	3	0.00	34.89	19.29	4.17	1.70	2.66	3.21	0.50	5.51	6.08	35.51	20.40	17.62	5.72	3.24	2.23	1.74	1.19	1.39	5.25
	4	0.00	37.30	18.08	4.35	1.66	1.80	3.22	0.51	8.28	5.90	46.84	25.75	18.04	7.10	3.93	2.63	1.34	0.50	3.80	6.06
	5	0.00	29.10	22.25	4.95	2.46	0.75	1.12	2.16	1.25	5.65	12.55	36.99	24.89	7.39	4.52	2.80	2.56	2.51	2.09	7.80
	6	0.00	45.95	25.99	5.80	2.80	1.40	2.25	0.83	0.00	7.33	0.00	46.46	21.29	6.11	5.40	2.38	3.10	0.89	1.24	7.50
	7	7.34	47.08	18.43	4.67	2.15	1.80	0.54	0.51	0.00	6.16	25.81	43.38	23.10	5.74	3.98	2.87	0.80	1.99	1.29	7.15
	8	30.26	52.65	20.73	3.58	1.66	1.23	2.48	0.95	2.96	6.57	6.98	50.61	18.06	8.27	2.85	2.15	1.90	0.41	5.41	6.87
	9	18.54	34.55	18.24	3.23	1.81	0.96	2.09	0.50	0.00	5.19	21.10	27.48	16.91	3.44	4.58	2.46	1.25	0.00	2.33	5.61
	10	4.42	39.70	23.30	5.36	1.44	0.78	0.76	0.87	0.00	6.05	13.96	54.30	18.44	8.46	6.00	2.72	2.06	0.39	3.33	8.15
	11	7.83	41.73	17.49	4.57	1.54	1.23	1.83	1.81	1.22	5.72	0.00	46.74	21.26	5.54	5.23	2.77	1.14	1.22	1.43	7.47
	12	6.88	43.74	15.49	5.11	2.69	2.00	2.98	1.80	0.00	6.10	29.27	26.56	14.56	6.39	4.16	2.38	1.13	2.05	0.00	5.60
	13	16.50	28.00	18.69	3.63	1.51	1.02	1.60	0.96	4.11	5.16	3.32	28.90	15.35	5.61	4.08	2.90	0.89	3.53	2.25	5.84
**Quarter**	1	0.00	21.19	15.82	3.58	1.64	1.39	2.29	0.68	4.05	4.49	10.93	20.49	15.11	4.69	3.34	2.63	1.49	1.55	2.86	5.06
	2	0.00	37.20	21.25	4.86	2.27	1.34	1.88	1.14	2.72	6.11	24.34	37.26	21.06	6.52	4.56	2.72	2.00	1.37	2.41	7.06
	3	18.64	43.70	21.12	4.35	1.64	1.19	1.73	0.72	0.91	6.14	12.94	43.42	19.26	6.70	4.35	2.46	1.77	0.47	3.40	6.96
	4	9.60	39.26	17.13	4.33	1.99	1.36	2.05	1.40	1.64	5.69	10.03	35.58	17.11	5.99	4.50	2.63	1.05	2.21	1.13	6.38
**Year**	16/17	17.00	629.00	838.00	212.00	196.00	125.00	70.00	23.00	17.00	2127.00	21.00	579.00	753.00	303.00	439.00	250.00	64.00	37.00	27.00	2473.00
**Episodes**	F/N	6.93	35.37	18.87	4.29	1.89	1.32	1.99	0.99	2.34	5.62	14.74	34.25	18.19	5.98	4.20	2.61	1.59	1.40	2.45	6.38

Mean weekly incidence rate per 100,000 Persons.

Impetigo (ICD10: L01)

			M										F									
			<1yr	1-4yrs	5-14yrs	15-24yrs	25-44yrs	45-64yrs	65-74yrs	75-84yrs	85+yrs	All Ages	<1yr	1-4yrs	5-14yrs	15-24yrs	25-44yrs	45-64yrs	65-74yrs	75-84yrs	85+yrs	All Ages
**North**	Quarter	1	0.00	26.57	15.93	2.95	1.65	0.90	2.28	1.24	5.34	4.34	43.71	20.29	10.12	6.56	4.36	1.96	1.53	2.98	2.82	4.84
		2	0.00	41.55	19.96	3.83	2.70	1.08	2.51	0.58	0.00	5.76	54.68	37.98	26.40	7.74	5.80	3.32	2.37	1.97	1.83	8.20
		3	17.70	39.22	25.21	5.25	1.72	1.99	1.29	0.62	3.65	6.44	0.00	48.69	25.84	7.95	4.87	3.05	2.39	0.00	4.03	8.11
		4	0.00	33.21	16.96	5.42	2.22	1.24	1.27	1.81	1.89	5.20	14.17	36.15	15.68	5.96	5.60	3.16	2.43	2.50	1.04	6.63
	Year	16/17	3.00	183.00	258.00	75.00	68.00	42.00	23.00	7.00	6.00	665.00	8.00	175.00	250.00	126.00	161.00	91.00	29.00	15.00	10.00	865.00
	Episodes	F/N	4.34	35.26	19.52	4.35	2.09	1.30	1.85	1.05	2.67	5.44	28.64	35.82	19.64	7.07	5.17	2.88	2.18	1.86	2.42	6.97
**South**	Quarter	1	0.00	24.83	18.59	5.16	0.99	1.17	3.33	1.48	7.75	5.11	0.00	16.67	20.04	3.97	3.90	2.49	0.00	1.29	1.09	5.10
		2	0.00	38.92	24.31	5.04	2.10	1.05	1.33	1.16	1.43	6.32	33.07	35.09	16.43	5.82	4.01	2.77	2.27	1.09	0.00	6.07
		3	33.22	57.71	20.92	5.02	1.37	1.05	2.32	2.25	0.00	6.88	12.33	53.86	18.33	12.09	4.86	2.42	1.63	1.05	2.77	8.10
		4	21.45	38.43	18.57	5.34	1.52	1.11	1.57	1.78	4.67	5.85	8.69	36.11	15.04	4.47	5.40	1.85	0.27	3.97	1.76	5.99
	Year	16/17	9.00	207.00	275.00	78.00	44.00	32.00	24.00	11.00	8.00	688.00	5.00	180.00	213.00	101.00	132.00	70.00	13.00	14.00	6.00	734.00
	Episodes	F/N	13.41	39.95	20.67	5.14	1.51	1.09	2.12	1.66	3.42	6.05	13.89	35.43	17.44	6.57	4.53	2.39	1.07	1.84	1.38	6.31
**London**	Quarter	1	0.00	12.52	11.11	3.04	2.10	0.99	1.09	0.00	0.00	3.59	0.00	13.72	10.50	2.81	1.85	1.57	0.97	0.00	5.85	3.55
		2	0.00	30.57	19.18	2.66	1.33	0.90	0.96	0.00	9.47	5.34	9.63	28.04	17.27	1.72	1.59	1.89	0.00	0.00	0.00	4.83
		3	0.00	28.22	13.60	2.81	1.01	0.47	1.21	0.00	0.00	4.23	15.95	16.91	9.48	2.13	2.29	2.52	1.06	0.00	6.82	3.97
		4	8.50	30.65	12.80	2.66	1.10	1.16	3.16	0.00	0.00	4.60	8.86	25.25	10.80	4.47	2.11	2.46	0.00	1.49	0.00	4.66
	Year	16/17	2.00	119.00	140.00	24.00	44.00	15.00	6.00	0.00	2.00	352.00	5.00	97.00	115.00	26.00	66.00	34.00	2.00	1.00	4.00	350.00
	Episodes	F/N	2.08	25.59	14.27	2.79	1.38	0.88	1.59	0.00	2.50	4.46	8.63	21.11	12.11	2.76	1.95	2.10	0.50	0.36	3.11	4.26
**Midlands And East**	Quarter	1	0.00	20.82	17.63	3.16	1.81	2.50	2.46	0.00	3.09	4.91	0.00	31.28	19.79	5.44	3.26	4.51	3.48	1.96	1.71	6.74
		2	0.00	37.77	21.54	7.92	2.95	2.31	2.72	2.84	0.00	7.03	0.00	47.91	24.16	10.80	6.84	2.89	3.37	2.41	7.81	9.15
		3	23.63	49.64	24.77	4.33	2.44	1.23	2.10	0.00	0.00	7.01	23.46	54.23	23.38	4.63	5.38	1.86	2.00	0.81	0.00	7.64
		4	8.47	54.73	20.18	3.91	3.12	1.95	2.20	2.03	0.00	7.10	8.40	44.82	26.90	9.05	4.89	3.04	1.51	0.89	1.73	8.24
	Year	16/17	3.00	120.00	165.00	35.00	40.00	36.00	17.00	5.00	1.00	422.00	3.00	127.00	175.00	50.00	80.00	55.00	20.00	7.00	7.00	524.00
	Episodes	F/N	7.87	40.68	21.04	4.89	2.59	2.01	2.38	1.25	0.76	6.52	7.81	44.62	23.57	7.54	5.13	3.07	2.61	1.53	2.91	7.96

Mean weekly incidence rate per 100,000 Persons.

Disorders of The Peripheral Nervous System (ICD10: G50-G64,G70-G72)

6.

		M										F									
		<1yr	1-4yrs	5-14yrs	15-24yrs	25-44yrs	45-64yrs	65-74yrs	75-84yrs	85+yrs	All Ages	<1yr	1-4yrs	5-14yrs	15-24yrs	25-44yrs	45-64yrs	65-74yrs	75-84yrs	85+yrs	All Ages
**4 weekly**	1	0.00	0.00	0.38	0.16	3.60	8.60	13.56	19.74	24.21	6.09	0.00	0.00	0.21	1.96	7.85	14.14	13.87	15.43	14.94	8.59
	2	0.00	0.00	0.00	1.32	5.81	10.46	13.09	16.61	22.54	7.05	0.00	0.00	0.00	2.76	10.60	20.54	18.04	23.95	13.96	11.79
	3	0.00	0.00	0.47	2.00	4.74	12.19	13.71	20.84	3.89	7.29	0.00	0.00	0.63	3.72	7.10	18.67	17.57	23.97	17.12	10.72
	4	0.00	0.00	0.26	1.55	4.74	10.70	19.75	21.39	11.58	7.38	0.00	0.00	0.27	1.44	11.45	20.14	19.22	17.08	17.42	11.80
	5	0.00	0.00	0.49	0.90	4.68	9.41	14.09	23.58	26.97	6.88	0.00	0.00	0.34	3.02	11.37	17.25	17.98	23.37	14.31	11.46
	6	0.00	0.00	0.42	0.59	6.58	13.60	17.93	15.14	15.33	8.24	0.00	0.72	0.35	3.73	9.83	17.76	21.73	30.23	33.98	12.54
	7	0.00	0.00	0.00	0.79	4.14	11.06	11.96	24.01	25.27	6.86	0.00	0.00	0.76	4.02	12.99	17.46	19.14	21.30	9.75	11.87
	8	0.00	0.00	0.00	0.44	5.33	11.19	19.90	22.94	11.11	7.58	0.00	0.00	0.27	3.51	10.83	15.39	19.20	24.48	15.77	11.06
	9	0.00	0.00	0.61	0.98	3.47	7.14	8.27	14.96	14.33	4.91	0.00	0.00	0.24	2.39	6.83	12.97	12.91	11.64	7.37	7.84
	10	0.00	0.00	0.00	2.67	4.82	11.98	15.07	21.63	25.00	7.62	0.00	0.00	0.00	3.31	13.54	19.15	15.52	26.45	21.20	12.68
	11	0.00	0.00	0.66	1.39	4.33	12.18	12.05	15.37	19.73	6.90	0.00	0.00	0.23	1.87	11.24	14.14	13.54	20.78	17.48	9.89
	12	0.00	0.00	0.00	0.77	5.01	11.53	17.66	17.26	36.26	7.47	0.00	0.00	0.24	1.86	10.12	17.65	17.06	26.61	25.51	11.33
	13	0.00	0.00	0.00	0.96	5.91	10.14	12.51	17.67	12.86	6.72	0.00	0.00	0.00	3.31	9.28	16.09	11.69	25.44	19.13	9.99
**Quarter**	1	0.00	0.00	0.29	1.08	4.63	10.27	13.46	19.12	17.44	6.75	0.00	0.00	0.27	2.74	8.46	17.51	16.29	20.68	15.31	10.23
	2	0.00	0.00	0.33	1.04	5.32	11.10	16.29	21.08	18.60	7.43	0.00	0.21	0.38	2.76	11.11	17.80	19.35	24.15	20.91	11.82
	3	0.00	0.00	0.19	1.13	4.33	10.43	14.15	19.40	17.31	6.69	0.00	0.00	0.28	3.45	10.77	16.24	16.41	19.60	13.32	10.73
	4	0.00	0.00	0.20	1.14	5.02	11.13	14.21	17.57	23.71	7.02	0.00	0.00	0.14	2.33	10.35	16.29	14.44	24.35	20.14	10.53
**Year**	16/17	0.00	0.00	10.00	54.00	506.00	1056.00	526.00	380.00	125.00	2657.00	0.00	1.00	12.00	129.00	1112.00	1595.00	634.00	491.00	208.00	4182.00
**Episodes**	F/N	0.00	0.00	0.26	1.10	4.84	10.74	14.56	19.33	19.25	6.98	0.00	0.05	0.27	2.82	10.19	16.97	16.67	22.23	17.49	10.85

Mean weekly incidence rate per 100,000 Persons.

Disorders of The Peripheral Nervous System (ICD10: G50-G64,G70-G72)

			M										F									
			<1yr	1-4yrs	5-14yrs	15-24yrs	25-44yrs	45-64yrs	65-74yrs	75-84yrs	85+yrs	All Ages	<1yr	1-4yrs	5-14yrs	15-24yrs	25-44yrs	45-64yrs	65-74yrs	75-84yrs	85+yrs	All Ages
**North**	Quarter	1	0.00	0.00	0.00	0.49	5.22	10.55	14.08	10.36	6.86	6.44	0.00	0.00	0.66	2.95	9.12	15.97	15.50	12.54	11.39	9.80
		2	0.00	0.00	0.29	1.37	4.98	12.95	16.48	27.95	18.60	8.51	0.00	0.00	0.31	2.11	11.14	20.55	18.01	14.45	16.81	11.76
		3	0.00	0.00	0.00	1.12	3.46	10.98	12.56	15.80	10.94	6.29	0.00	0.00	0.33	1.94	10.27	14.50	14.96	15.92	8.00	9.41
		4	0.00	0.00	0.30	1.85	5.29	10.42	14.63	14.60	26.30	7.18	0.00	0.00	0.00	2.78	9.04	14.82	15.20	21.92	18.55	10.01
	Year	16/17	0.00	0.00	2.00	21.00	153.00	362.00	181.00	115.00	35.00	869.00	0.00	0.00	4.00	44.00	308.00	520.00	210.00	131.00	56.00	1,273.00
	Episodes	F/N	0.00	0.00	0.15	1.21	4.74	11.26	14.48	17.38	15.73	7.13	0.00	0.00	0.33	2.44	9.91	16.54	15.96	16.18	13.75	10.27
**South**	Quarter	1	0.00	0.00	0.00	0.64	5.61	9.86	17.30	25.43	13.38	7.67	0.00	0.00	0.00	1.31	7.79	17.99	13.88	25.81	13.06	10.46
		2	0.00	0.00	0.57	0.77	5.48	10.30	19.71	20.10	24.31	8.00	0.00	0.00	0.00	2.49	12.21	16.11	19.08	27.41	26.48	12.38
		3	0.00	0.00	0.28	0.98	4.39	12.03	18.31	20.10	17.06	7.81	0.00	0.00	0.30	3.29	11.59	16.04	19.73	18.84	18.21	11.45
		4	0.00	0.00	0.00	1.73	6.30	11.99	13.98	29.86	14.44	8.37	0.00	0.00	0.57	0.95	14.02	16.69	19.47	19.93	14.77	11.85
	Year	16/17	0.00	0.00	3.00	16.00	156.00	330.00	199.00	150.00	43.00	897.00	0.00	0.00	3.00	31.00	334.00	488.00	227.00	174.00	81.00	1,338.00
	Episodes	F/N	0.00	0.00	0.22	1.02	5.45	11.03	17.37	23.80	17.43	7.96	0.00	0.00	0.21	2.02	11.42	16.70	18.06	23.08	18.29	11.55
**London**	Quarter	1	0.00	0.00	0.00	2.57	3.17	9.42	8.92	15.67	25.60	4.72	0.00	0.00	0.44	3.31	8.33	20.31	22.45	25.98	17.83	10.24
		2	0.00	0.00	0.00	0.45	3.95	9.86	17.05	16.08	18.57	5.23	0.00	0.82	1.19	3.66	9.02	18.40	21.08	24.58	14.14	10.11
		3	0.00	0.00	0.00	1.90	3.08	7.42	11.02	17.80	16.28	4.19	0.00	0.00	0.00	3.45	10.12	16.81	11.46	13.65	13.01	9.09
		4	0.00	0.00	0.00	0.44	3.30	9.70	15.09	5.90	33.06	4.58	0.00	0.00	0.00	1.97	9.43	17.65	10.28	28.36	32.32	9.42
	Year	16/17	0.00	0.00	0.00	11.00	108.00	155.00	49.00	29.00	18.00	370.00	0.00	1.00	4.00	29.00	312.00	291.00	70.00	63.00	25.00	795.00
	Episodes	F/N	0.00	0.00	0.00	1.32	3.39	9.11	13.09	13.90	23.28	4.69	0.00	0.22	0.42	3.11	9.22	18.29	16.41	23.17	19.23	9.72
**Midlands And East**	Quarter	1	0.00	0.00	1.17	0.63	4.53	11.26	13.55	25.02	23.93	8.18	0.00	0.00	0.00	3.41	8.61	15.76	13.34	18.38	18.96	10.42
		2	0.00	0.00	0.48	1.59	6.87	11.29	11.92	20.21	12.93	7.96	0.00	0.00	0.00	2.79	12.06	16.13	19.23	30.18	26.20	13.02
		3	0.00	0.00	0.47	0.54	6.40	11.28	14.68	23.92	24.96	8.46	0.00	0.00	0.50	5.11	11.09	17.60	19.50	30.00	14.05	12.98
		4	0.00	0.00	0.52	0.54	5.18	12.42	13.13	19.93	21.06	7.96	0.00	0.00	0.00	3.64	8.91	16.01	12.83	27.17	14.91	10.84
	Year	16/17	0.00	0.00	5.00	6.00	89.00	209.00	97.00	86.00	29.00	521.00	0.00	0.00	1.00	25.00	158.00	296.00	127.00	123.00	46.00	776.00
	Episodes	F/N	0.00	0.00	0.66	0.84	5.77	11.56	13.29	22.23	20.57	8.14	0.00	0.00	0.12	3.72	10.20	16.37	16.28	26.50	18.68	11.84

Mean weekly incidence rate per 100,000 Persons.

Meningitis and Encephalitis (ICD10: A170 - A171; A 390; A83 - A85; A87; G00 - G05)

		M										F									
		<1yr	1-4yrs	5-14yrs	15-24yrs	25-44yrs	45-64yrs	65-74yrs	75-84yrs	85+yrs	All Ages	<1yr	1-4yrs	5-14yrs	15-24yrs	25-44yrs	45-64yrs	65-74yrs	75-84yrs	85+yrs	All Ages
**4 weekly**	1	0.00	0.69	0.00	0.00	0.00	0.16	0.00	0.00	0.00	0.06	0.00	0.00	0.00	0.37	0.22	0.11	0.20	0.00	0.00	0.15
	2	0.00	0.00	0.00	0.19	0.11	0.26	0.00	0.00	0.00	0.14	0.00	0.00	0.00	0.00	0.32	0.31	0.00	0.00	0.00	0.20
	3	0.00	0.71	0.00	0.00	0.32	0.00	0.00	0.65	0.00	0.21	67.20	0.89	0.00	0.66	0.34	0.00	0.00	0.41	0.00	0.30
	4	17.51	0.00	0.35	0.00	0.22	0.00	0.00	0.00	0.00	0.14	0.00	0.00	0.00	0.00	0.32	0.11	0.00	0.00	0.00	0.14
	5	0.00	0.00	0.00	0.00	0.00	0.00	0.00	0.00	0.00	0.00	14.24	0.00	0.00	0.00	0.00	0.32	0.25	0.00	0.83	0.18
	6	8.80	0.00	0.00	0.45	0.11	0.00	0.00	0.50	0.00	0.14	0.00	0.00	0.00	0.21	0.22	0.12	0.00	0.00	0.00	0.11
	7	0.00	0.00	0.00	0.00	0.22	0.00	0.00	0.77	0.00	0.11	9.27	0.00	0.00	0.00	0.11	0.00	0.00	0.00	0.00	0.07
	8	6.85	0.00	0.00	0.18	0.22	0.00	0.00	0.00	0.00	0.15	0.00	0.00	0.00	0.00	0.21	0.20	0.00	0.00	0.00	0.11
	9	0.00	0.00	0.00	0.00	0.41	0.21	0.27	0.50	0.00	0.22	0.00	0.00	0.00	0.00	0.21	0.00	0.00	0.00	0.00	0.05
	10	7.84	0.00	0.00	0.00	0.00	0.10	0.43	0.00	0.00	0.12	4.21	0.00	0.00	0.00	0.20	0.00	0.24	0.00	0.00	0.12
	11	0.00	0.00	0.00	0.00	0.11	0.00	0.90	0.00	0.00	0.07	4.37	0.00	0.00	0.00	0.00	0.00	0.00	0.81	0.00	0.08
	12	6.64	0.62	0.00	0.18	0.33	0.38	0.45	0.00	0.00	0.34	0.00	1.10	0.00	0.00	0.00	0.30	0.00	0.40	0.00	0.14
	13	0.00	0.64	0.22	0.00	0.20	0.00	0.00	0.00	0.00	0.11	0.00	0.69	0.00	0.55	0.21	0.10	0.74	0.00	0.00	0.27
**Quarter**	1	0.00	0.48	0.00	0.06	0.13	0.14	0.00	0.20	0.00	0.13	20.68	0.27	0.00	0.34	0.29	0.14	0.08	0.12	0.00	0.21
	2	7.52	0.00	0.10	0.13	0.12	0.00	0.00	0.36	0.00	0.10	6.72	0.00	0.00	0.06	0.19	0.16	0.07	0.00	0.24	0.14
	3	4.52	0.00	0.00	0.06	0.23	0.09	0.22	0.15	0.00	0.16	0.00	0.00	0.00	0.00	0.16	0.06	0.07	0.00	0.00	0.07
	4	2.04	0.38	0.07	0.06	0.20	0.12	0.41	0.00	0.00	0.16	2.64	0.55	0.00	0.17	0.10	0.12	0.23	0.37	0.00	0.17
**Year**	16/17	6.00	4.00	2.00	4.00	19.00	9.00	4.00	4.00	0.00	52.00	5.00	3.00	0.00	8.00	21.00	12.00	6.00	4.00	1.00	60.00
**Episodes**	F/N	3.60	0.21	0.04	0.08	0.17	0.09	0.15	0.18	0.00	0.14	7.49	0.20	0.00	0.14	0.18	0.12	0.11	0.12	0.06	0.15

Mean weekly incidence rate per 100,000 Persons.

Meningitis and Encephalitis (ICD10: A170 - A171; A 390; A83 - A85; A87; G00 - G05)

			M										F									
			<1yr	1-4yrs	5-14yrs	15-24yrs	25-44yrs	45-64yrs	65-74yrs	75-84yrs	85+yrs	All Ages	<1yr	1-4yrs	5-14yrs	15-24yrs	25-44yrs	45-64yrs	65-74yrs	75-84yrs	85+yrs	All Ages
**North**	Quarter	1	0.00	0.00	0.00	0.23	0.00	0.00	0.00	0.00	0.00	0.03	0.00	0.00	0.00	0.23	0.14	0.13	0.31	0.50	0.00	0.17
		2	10.06	0.00	0.00	0.00	0.12	0.00	0.00	0.00	0.00	0.07	0.00	0.00	0.00	0.00	0.12	0.24	0.29	0.00	0.94	0.16
		3	0.00	0.00	0.00	0.23	0.37	0.37	0.00	0.62	0.00	0.26	0.00	0.00	0.00	0.00	0.00	0.00	0.30	0.00	0.00	0.03
		4	4.25	1.54	0.00	0.00	0.12	0.12	0.00	0.00	0.00	0.16	0.00	0.00	0.00	0.21	0.26	0.25	0.61	1.49	0.00	0.32
	Year	16/17	2.00	2.00	0.00	2.00	5.00	4.00	0.00	1.00	0.00	16.00	0.00	0.00	0.00	2.00	4.00	5.00	5.00	4.00	1.00	21.00
	Episodes	F/N	3.70	0.38	0.00	0.11	0.15	0.12	0.00	0.15	0.00	0.13	0.00	0.00	0.00	0.11	0.13	0.16	0.38	0.49	0.25	0.17
**South**	Quarter	1	0.00	1.06	0.00	0.00	0.00	0.32	0.00	0.80	0.00	0.18	0.00	1.09	0.00	0.68	0.50	0.17	0.00	0.00	0.00	0.30
		2	0.00	0.00	0.00	0.00	0.13	0.00	0.00	0.57	0.00	0.06	0.00	0.00	0.00	0.24	0.40	0.38	0.00	0.00	0.00	0.23
		3	8.43	0.00	0.00	0.00	0.28	0.00	0.33	0.00	0.00	0.14	0.00	0.00	0.00	0.00	0.13	0.00	0.00	0.00	0.00	0.03
		4	3.93	0.00	0.27	0.23	0.40	0.11	0.00	0.00	0.00	0.22	10.56	0.00	0.00	0.47	0.13	0.00	0.30	0.00	0.00	0.19
	Year	16/17	2.00	1.00	1.00	1.00	6.00	3.00	1.00	2.00	0.00	17.00	2.00	1.00	0.00	5.00	8.00	4.00	1.00	0.00	0.00	21.00
	Episodes	F/N	3.03	0.26	0.07	0.06	0.20	0.11	0.08	0.35	0.00	0.15	2.59	0.27	0.00	0.35	0.29	0.14	0.07	0.00	0.00	0.19
**London**	Quarter	1	0.00	0.88	0.00	0.00	0.53	0.25	0.00	0.00	0.00	0.32	82.71	0.00	0.00	0.46	0.50	0.00	0.00	0.00	0.00	0.31
		2	20.01	0.00	0.40	0.00	0.00	0.00	0.00	0.00	0.00	0.10	26.87	0.00	0.00	0.00	0.23	0.00	0.00	0.00	0.00	0.19
		3	0.00	0.00	0.00	0.00	0.27	0.00	0.00	0.00	0.00	0.11	0.00	0.00	0.00	0.00	0.25	0.25	0.00	0.00	0.00	0.15
		4	0.00	0.00	0.00	0.00	0.00	0.00	1.11	0.00	0.00	0.05	0.00	0.85	0.00	0.00	0.00	0.24	0.00	0.00	0.00	0.09
	Year	16/17	1.00	1.00	1.00	0.00	6.00	1.00	1.00	0.00	0.00	11.00	3.00	1.00	0.00	1.00	8.00	2.00	0.00	0.00	0.00	15.00
	Episodes	F/N	5.29	0.21	0.11	0.00	0.20	0.06	0.27	0.00	0.00	0.14	27.39	0.21	0.00	0.11	0.24	0.12	0.00	0.00	0.00	0.19
**Midlands And East**	Quarter	1	0.00	0.00	0.00	0.00	0.00	0.00	0.00	0.00	0.00	0.00	0.00	0.00	0.00	0.00	0.00	0.25	0.00	0.00	0.00	0.07
		2	0.00	0.00	0.00	0.52	0.25	0.00	0.00	0.89	0.00	0.17	0.00	0.00	0.00	0.00	0.00	0.00	0.00	0.00	0.00	0.00
		3	9.65	0.00	0.00	0.00	0.00	0.00	0.53	0.00	0.00	0.12	0.00	0.00	0.00	0.00	0.26	0.00	0.00	0.00	0.00	0.06
		4	0.00	0.00	0.00	0.00	0.27	0.23	0.55	0.00	0.00	0.19	0.00	1.35	0.00	0.00	0.00	0.00	0.00	0.00	0.00	0.06
	Year	16/17	1.00	0.00	0.00	1.00	2.00	1.00	2.00	1.00	0.00	8.00	0.00	1.00	0.00	0.00	1.00	1.00	0.00	0.00	0.00	3.00
	Episodes	F/N	2.37	0.00	0.00	0.14	0.13	0.06	0.26	0.23	0.00	0.12	0.00	0.33	0.00	0.00	0.06	0.06	0.00	0.00	0.00	0.05

Mean weekly incidence rate per 100,000 Persons.

Symptoms Involving Nervous and Musculoskeletal (ICD10: R25-R29)

		M										F									
		<1yr	1-4yrs	5-14yrs	15-24yrs	25-44yrs	45-64yrs	65-74yrs	75-84yrs	85+yrs	All Ages	<1yr	1-4yrs	5-14yrs	15-24yrs	25-44yrs	45-64yrs	65-74yrs	75-84yrs	85+yrs	All Ages
**4 weekly**	1	0.00	5.48	3.11	1.71	2.60	4.77	3.27	8.08	11.31	3.73	0.00	7.81	2.78	2.77	3.82	5.85	5.92	6.50	7.64	4.70
	2	0.00	7.29	3.15	3.30	3.01	5.98	5.13	15.74	2.86	4.65	0.00	7.48	4.37	2.70	5.33	5.06	9.85	10.04	9.67	5.80
	3	0.00	5.32	1.61	4.11	3.63	2.42	3.02	6.58	4.00	3.37	0.00	10.10	2.57	2.47	3.00	7.15	5.76	12.83	9.60	5.19
	4	0.00	5.12	2.85	3.59	2.60	4.70	7.34	9.07	8.22	4.31	0.00	11.22	2.27	2.83	4.70	6.57	6.95	9.84	2.95	5.41
	5	0.00	4.04	5.03	6.03	2.82	5.54	4.49	5.62	1.36	4.57	0.00	4.13	3.10	2.97	6.19	6.83	12.29	6.25	7.48	5.93
	6	0.00	5.12	2.56	3.15	2.71	6.63	9.84	7.16	9.45	4.79	0.00	7.90	4.53	5.83	5.72	7.77	8.30	7.26	10.26	6.50
	7	0.00	4.82	4.31	2.66	4.26	6.74	6.37	6.86	9.67	5.09	0.00	5.95	5.65	2.69	6.02	9.93	10.44	12.73	10.80	6.96
	8	0.00	5.68	2.29	2.21	3.11	5.97	4.08	6.29	10.44	4.13	0.00	7.63	3.98	3.83	5.37	8.22	6.09	11.72	10.30	6.25
	9	0.00	2.66	2.00	1.12	2.54	4.77	3.62	6.56	4.35	3.11	0.00	3.02	2.97	5.97	4.09	7.01	5.68	13.34	8.57	5.45
	10	0.00	5.77	3.34	2.55	3.57	4.61	7.67	5.57	6.53	4.26	5.44	4.99	2.86	3.98	6.54	5.32	8.62	10.56	8.80	5.63
	11	0.00	5.85	3.70	4.69	3.52	4.88	6.34	5.19	7.05	4.56	0.00	5.22	3.18	2.34	5.23	6.53	9.43	10.01	11.74	5.72
	12	0.00	6.73	5.21	4.87	2.42	4.35	7.79	12.13	2.38	4.74	0.00	2.02	4.62	2.62	5.85	8.80	8.18	4.11	8.01	6.06
	13	0.00	8.68	2.43	1.93	3.81	3.40	3.49	3.30	9.95	3.53	0.00	3.85	2.76	4.23	5.59	6.23	4.51	9.94	4.36	5.11
**Quarter**	1	0.00	5.99	2.66	2.94	3.04	4.42	3.77	9.98	6.46	3.90	0.00	8.41	3.21	2.65	4.03	6.01	7.08	9.53	8.87	5.19
	2	0.00	4.66	3.41	3.99	3.02	5.80	7.42	6.98	5.81	4.64	0.00	7.22	3.72	3.49	5.70	7.64	9.76	8.67	8.30	6.17
	3	0.00	4.80	2.79	2.13	3.14	5.52	5.14	6.72	8.75	4.04	1.67	5.57	3.80	4.82	5.36	7.21	7.00	11.87	9.27	5.98
	4	0.00	6.94	3.91	3.66	3.26	4.11	5.65	6.61	6.34	4.22	0.00	3.86	3.25	2.89	5.52	7.09	7.26	8.25	7.42	5.53
**Year**	16/17	0.00	101.00	141.00	144.00	359.00	472.00	200.00	142.00	47.00	1606.00	1.00	107.00	148.00	162.00	593.00	640.00	269.00	203.00	100.00	2223.00
**Episodes**	F/N	0.00	5.58	3.20	3.20	3.11	4.98	5.53	7.56	6.82	4.21	0.41	6.29	3.50	3.46	5.16	7.00	7.81	9.57	8.46	5.73

Mean weekly incidence rate per 100,000 Persons.

Symptoms Involving Nervous and Musculoskeletal (ICD10: R25-R29)

			M										F									
			<1yr	1-4yrs	5-14yrs	15-24yrs	25-44yrs	45-64yrs	65-74yrs	75-84yrs	85+yrs	All Ages	<1yr	1-4yrs	5-14yrs	15-24yrs	25-44yrs	45-64yrs	65-74yrs	75-84yrs	85+yrs	All Ages
**North**	Quarter	1	0.00	8.18	5.00	2.27	5.35	5.27	6.85	12.16	5.23	5.53	0.00	9.50	3.19	1.73	6.22	7.29	7.13	9.15	13.17	6.22
		2	0.00	5.24	2.93	4.93	4.02	5.15	8.38	5.78	10.12	5.02	0.00	7.93	3.33	4.31	6.90	6.54	9.95	9.09	8.36	6.61
		3	0.00	4.62	2.73	1.59	3.67	4.88	6.48	10.39	7.27	4.32	0.00	4.84	4.76	3.85	6.91	6.68	5.55	10.01	12.10	6.28
		4	0.00	5.43	2.74	3.04	4.13	5.45	8.91	10.21	7.45	5.06	0.00	6.51	3.13	2.75	6.38	5.48	6.30	6.47	8.23	5.32
	Year	16/17	0.00	30.00	44.00	51.00	138.00	167.00	96.00	64.00	17.00	607.00	0.00	35.00	46.00	58.00	206.00	204.00	96.00	70.00	43.00	758.00
	Episodes	F/N	0.00	5.86	3.34	2.99	4.28	5.19	7.67	9.56	7.57	4.98	0.00	7.21	3.60	3.18	6.61	6.50	7.28	8.69	10.42	6.12
**South**	Quarter	1	0.00	3.91	2.62	2.93	2.55	3.12	2.90	5.39	7.68	3.13	0.00	10.26	4.72	2.32	2.54	5.48	4.68	4.57	4.34	4.26
		2	0.00	6.46	1.76	2.35	2.33	4.60	6.61	7.90	3.05	3.81	0.00	6.29	2.96	2.82	5.87	5.55	5.66	7.55	3.38	5.07
		3	0.00	4.60	3.45	2.05	2.88	5.29	3.18	3.78	11.63	3.80	0.00	6.30	3.99	3.46	5.05	7.15	7.38	7.44	4.95	5.68
		4	0.00	5.05	3.38	1.95	2.57	2.82	4.41	4.07	6.24	3.10	0.00	1.46	2.37	2.73	5.09	5.84	4.17	9.15	8.28	4.77
	Year	16/17	0.00	25.00	37.00	34.00	75.00	118.00	51.00	33.00	17.00	390.00	0.00	28.00	43.00	43.00	137.00	177.00	68.00	56.00	23.00	575.00
	Episodes	F/N	0.00	5.03	2.78	2.32	2.58	3.97	4.32	5.34	7.07	3.47	0.00	6.08	3.50	2.83	4.66	6.00	5.48	7.18	5.20	4.95
**London**	Quarter	1	0.00	5.34	1.26	4.04	3.07	6.26	2.22	14.43	9.91	4.10	0.00	7.32	3.07	3.80	5.31	7.99	7.81	16.67	5.96	6.04
		2	0.00	5.67	5.49	3.55	4.08	7.15	9.08	7.09	4.74	5.27	0.00	9.24	3.59	2.90	6.55	12.95	16.64	12.49	16.96	8.09
		3	0.00	8.70	2.05	3.80	3.34	7.73	7.70	5.84	10.56	4.80	6.69	1.81	3.89	3.83	4.61	10.45	8.65	21.00	9.42	6.26
		4	0.00	9.25	6.49	3.58	3.79	5.34	4.29	3.85	5.59	4.75	0.00	6.10	3.29	2.47	6.03	11.37	10.37	6.06	6.49	6.50
	Year	16/17	0.00	34.00	38.00	32.00	114.00	112.00	22.00	16.00	6.00	374.00	1.00	28.00	33.00	30.00	191.00	172.00	47.00	38.00	13.00	553.00
	Episodes	F/N	0.00	7.21	3.85	3.74	3.58	6.63	5.88	7.79	7.64	4.74	1.64	6.18	3.46	3.24	5.64	10.73	10.98	14.03	9.84	6.75
**Midlands And East**	Quarter	1	0.00	6.52	1.77	2.51	1.20	3.01	3.09	7.93	3.01	2.85	0.00	6.57	1.84	2.76	2.08	3.27	8.69	7.74	12.01	4.24
		2	0.00	1.27	3.47	5.14	1.65	6.30	5.63	7.16	5.33	4.44	0.00	5.43	5.00	3.93	3.47	5.50	6.79	5.56	4.50	4.91
		3	0.00	1.28	2.93	1.08	2.68	4.18	3.18	6.87	5.56	3.24	0.00	9.33	2.56	8.16	4.87	4.57	6.40	9.05	10.62	5.70
		4	0.00	8.01	3.03	6.05	2.54	2.82	4.99	8.32	6.10	3.99	0.00	1.38	4.21	3.60	4.59	5.66	8.20	11.33	6.66	5.54
	Year	16/17	0.00	12.00	22.00	27.00	32.00	75.00	31.00	29.00	7.00	235.00	0.00	16.00	26.00	31.00	59.00	87.00	58.00	39.00	21.00	337.00
	Episodes	F/N	0.00	4.22	2.81	3.72	2.01	4.12	4.25	7.56	5.00	3.64	0.00	5.67	3.43	4.60	3.75	4.76	7.51	8.37	8.37	5.10

7. Genitourinary System Disorders:

Mean weekly incidence rate per 100,000 Persons.

Urinary Tract Infection/Cystitis (ICD10: N39.0,N30)

		M										F									
		<1yr	1-4yrs	5-14yrs	15-24yrs	25-44yrs	45-64yrs	65-74yrs	75-84yrs	85+yrs	All Ages	<1yr	1-4yrs	5-14yrs	15-24yrs	25-44yrs	45-64yrs	65-74yrs	75-84yrs	85+yrs	All Ages
**4 weekly**	1	0.00	6.44	1.26	1.47	2.34	5.72	21.68	33.02	49.20	7.07	0.00	11.43	13.36	27.75	28.02	30.36	50.35	89.25	135.70	34.95
	2	0.00	3.48	1.11	1.52	2.30	7.70	21.79	48.21	71.95	9.21	0.00	13.68	15.77	28.45	33.15	39.66	65.84	117.52	153.98	42.95
	3	0.00	4.13	2.89	0.95	2.18	7.43	22.24	61.03	123.35	10.07	67.20	17.27	10.30	32.91	43.83	42.48	75.65	119.50	183.74	48.62
	4	30.19	8.60	3.45	0.61	2.50	9.21	25.29	50.58	121.44	11.08	0.00	24.07	14.15	36.28	41.41	44.68	86.44	117.82	172.87	51.04
	5	33.86	9.45	2.34	1.91	3.07	7.69	28.05	64.20	96.10	11.34	0.00	21.14	18.09	45.95	38.54	46.74	84.85	127.11	172.19	51.78
	6	8.80	2.68	3.19	1.31	3.63	8.91	24.18	53.29	104.79	10.45	0.00	24.82	20.14	47.70	38.37	41.62	80.28	129.62	156.80	50.12
	7	30.61	1.26	2.00	2.14	2.48	8.57	20.79	48.53	111.24	9.65	7.48	20.48	12.72	40.74	36.68	44.16	83.42	113.94	175.64	48.58
	8	37.78	4.96	2.43	0.19	2.55	7.14	24.64	52.37	112.74	9.70	13.98	19.98	15.78	38.58	31.86	40.64	78.96	97.93	151.48	43.72
	9	5.09	6.53	2.51	1.60	2.61	8.67	22.54	49.39	101.76	9.80	21.83	18.35	17.08	30.84	32.29	39.12	57.50	101.58	117.16	39.84
	10	17.80	6.22	3.88	0.20	3.57	8.49	21.07	44.14	72.94	9.35	13.69	19.85	17.77	42.74	38.17	43.87	74.08	111.29	155.33	47.23
	11	4.08	9.04	1.59	1.26	2.97	8.93	23.25	48.32	90.07	10.14	8.47	27.46	14.31	38.63	35.10	40.30	75.29	110.72	159.47	44.71
	12	11.12	4.01	2.09	1.98	2.49	6.43	21.86	52.29	89.07	8.83	11.41	21.52	21.42	43.86	34.77	39.55	64.98	122.78	157.47	45.74
	13	6.65	8.35	2.12	1.11	2.13	8.96	22.38	44.41	117.82	9.73	3.29	18.38	11.15	34.99	29.26	34.46	60.63	98.23	127.26	38.12
**Quarter**	1	0.00	4.82	1.71	1.33	2.28	6.86	21.89	46.31	79.01	8.65	20.68	13.92	13.16	29.55	34.46	36.95	62.90	107.25	156.11	41.62
	2	29.56	6.28	2.70	1.20	3.04	8.65	25.19	55.23	108.90	10.81	0.00	21.90	16.84	42.67	39.07	44.13	81.71	122.53	172.68	50.43
	3	18.67	4.50	3.08	1.16	2.77	8.06	21.86	49.75	98.50	9.65	17.53	21.00	16.70	37.28	34.34	41.62	75.02	107.14	139.39	44.52
	4	6.72	7.54	1.89	1.34	2.56	8.16	23.02	46.93	97.44	9.50	7.13	21.90	15.19	39.93	33.27	38.58	66.66	108.92	150.24	42.97
**Year**	16/17	25.00	102.00	103.00	59.00	283.00	770.00	798.00	910.00	632.00	3682.00	16.00	335.00	663.00	1836.00	3854.00	3768.00	2568.00	2464.00	1789.00	17293.00
**Episodes**	F/N	14.04	5.79	2.35	1.26	2.67	7.95	23.03	49.66	96.21	9.68	11.12	19.72	15.50	37.46	35.36	40.39	71.76	111.67	154.94	44.99

Mean weekly incidence rate per 100,000 Persons.

Urinary Tract Infection/Cystitis (ICD10: N39.0,N30)

			M										F									
			<1yr	1-4yrs	5-14yrs	15-24yrs	25-44yrs	45-64yrs	65-74yrs	75-84yrs	85+yrs	All Ages	<1yr	1-4yrs	5-14yrs	15-24yrs	25-44yrs	45-64yrs	65-74yrs	75-84yrs	85+yrs	All Ages
**North**	Quarter	1	0.00	6.37	1.62	1.58	2.91	7.91	20.46	49.77	60.30	9.70	0.00	16.13	13.77	31.30	34.67	33.68	61.31	99.87	116.50	41.15
		2	32.82	3.70	2.33	0.67	3.42	9.40	24.05	49.08	124.93	11.45	0.00	26.73	18.89	50.05	39.89	47.01	77.45	125.12	155.40	53.91
		3	25.17	2.39	3.01	1.84	3.19	7.19	24.05	45.99	73.23	9.80	20.21	23.13	16.10	41.67	34.71	40.87	64.83	101.13	132.60	45.51
		4	4.71	5.47	2.09	1.87	3.25	7.97	24.81	43.66	85.02	10.05	13.51	24.60	15.30	38.76	32.36	37.00	52.82	100.25	110.26	41.30
	Year	16/17	8.00	23.00	30.00	25.00	104.00	262.00	292.00	315.00	194.00	1,253.00	6.00	111.00	204.00	735.00	1,102.00	1,253.00	847.00	863.00	527.00	5,648.00
	Episodes	F/N	16.00	4.47	2.26	1.47	3.20	8.14	23.35	47.16	86.61	10.27	8.27	22.73	16.07	40.62	35.49	39.78	64.36	106.94	129.20	45.63
**South**	Quarter	1	0.00	5.06	2.21	0.97	1.68	7.27	20.14	34.60	80.52	8.81	0.00	13.52	15.87	27.07	36.23	35.18	59.24	96.02	174.08	43.51
		2	34.87	8.56	2.02	0.75	2.44	7.23	22.61	55.74	90.85	10.84	0.00	17.45	16.78	33.83	34.14	43.32	70.77	101.44	150.18	46.67
		3	35.25	6.75	2.10	0.76	1.44	7.38	21.09	45.94	107.83	10.15	8.31	18.56	16.86	35.27	30.54	36.38	70.13	93.54	136.68	42.88
		4	13.99	7.77	1.89	0.94	1.94	8.30	21.66	46.66	88.56	10.13	9.40	13.77	15.74	32.41	25.62	30.58	59.42	95.23	141.53	38.46
	Year	16/17	11.00	36.00	27.00	13.00	54.00	224.00	249.00	292.00	225.00	1,131.00	3.00	78.00	205.00	487.00	901.00	1,070.00	804.00	738.00	648.00	4,934.00
	Episodes	F/N	21.29	7.07	2.05	0.85	1.89	7.54	21.40	45.93	91.92	10.00	4.34	15.85	16.32	32.18	31.68	36.50	65.00	96.65	150.61	42.95
**London**	Quarter	1	0.00	4.57	1.27	1.51	2.11	7.21	24.79	62.17	105.53	6.99	82.71	14.49	10.65	28.29	32.92	45.64	71.89	137.12	182.69	39.45
		2	34.51	7.49	3.55	1.79	2.42	10.25	24.98	65.01	133.40	8.62	0.00	21.54	18.93	43.19	46.47	49.48	96.25	136.43	218.37	50.50
		3	14.26	3.53	3.30	0.47	2.80	9.99	23.60	59.50	124.52	7.90	31.24	17.92	18.58	36.51	39.20	50.59	101.59	127.69	175.09	45.69
		4	8.18	6.04	3.57	0.88	1.94	6.57	25.94	50.48	122.48	6.72	5.59	18.12	17.87	40.67	35.20	44.53	80.39	138.31	203.89	42.50
	Year	16/17	5.00	25.00	29.00	10.00	74.00	145.00	93.00	123.00	94.00	598.00	6.00	83.00	157.00	350.00	1,301.00	757.00	373.00	365.00	256.00	3,648.00
	Episodes	F/N	14.62	5.45	2.93	1.17	2.32	8.54	24.83	59.40	121.71	7.58	29.32	18.08	16.55	37.28	38.60	47.60	87.69	134.92	195.45	44.65
**Midlands And East**	Quarter	1	0.00	3.27	1.76	1.25	2.42	5.02	22.15	38.71	69.71	9.10	0.00	11.54	12.35	31.55	34.03	33.29	59.15	96.01	151.16	42.36
		2	16.05	5.38	2.90	1.61	3.90	7.73	29.14	51.11	86.43	12.34	0.00	21.87	12.75	43.63	35.78	36.73	82.36	127.11	166.76	50.66
		3	0.00	5.31	3.91	1.57	3.67	7.69	18.71	47.56	88.42	10.77	10.37	24.38	15.27	35.69	32.90	38.65	63.51	106.18	113.21	44.01
		4	0.00	10.87	0.00	1.66	3.11	9.81	19.66	46.90	93.70	11.08	0.00	31.09	11.86	47.90	39.91	42.23	74.02	101.90	145.27	49.61
	Year	16/17	1.00	18.00	17.00	11.00	51.00	139.00	164.00	180.00	119.00	700.00	1.00	63.00	97.00	264.00	550.00	688.00	544.00	498.00	358.00	3,063.00
	Episodes	F/N	4.24	6.19	2.15	1.52	3.28	7.56	22.54	46.17	84.60	10.85	2.54	22.21	13.05	39.77	35.66	37.71	70.00	108.17	144.53	46.74
