# Supplementary material for: Conurbation, Urban, and Rural Living as Determinants of Allergies and Infectious Diseases: Royal College of General Practitioners Research and Surveillance Centre Annual Report 2016-2017
Source: JMIR Public Health Surveill. 2018 Nov 26;4(4):e11354. doi: 10.2196/11354 (PMC6288591; doi:10.2196/11354)
Supplement: Multimedia Appendix 2 [file publichealth_v4i4e11354_app2.pdf]

Supplementary File B
Detailed results for the interaction between age band/gender and urban/rural/conurbation living on disease rates

Table B.1: *Numerators and denominators with percentages and chi square p value for allergic rhinitis and asthma*

		Allergic Rhinitis Yes n (%)	Allergic Rhinitis No n (%)	Asthma Yes n (%)	Asthma No n (%)	Denominator (N)
Total		14068 (0.88%)	1588298 (99.12%)	47180 (2.94%)	1555186 (97.06%)	1602366
Urban Rural	Conurbation	5613 (1.08%)	514885 (98.92%)	13021 (2.50%)	507477 (97.50%)	520498
	Urban	6255 (0.81%)	766454 (99.19%)	24208 (3.13%)	748501 (96.87%)	772709
	Rural	2200 (0.71%)	306959 (99.29%)	9951 (3.22%)	299208 (96.78%)	309159
	p value	p<0.001		p<0.001		
Gender	M	6540 (0.82%)	787145 (99.18%)	20207 (2.55%)	773478 (97.45%)	793685
	F	7528 (0.93%)	801153 (99.07%)	26973 (3.34%)	781708 (96.66%)	808681
	p value	p<0.001		p<0.001		
Age Band	0-4	613 (0.66%)	92608 (99.34%)	1240 (1.33%)	91981 (98.67%)	93221
	5-17	3891 (0.39%)	232716 (23.48%)	7161 (3.03%)	229446 (96.97%)	236607
	18-64	7971 (0.80%)	991167 (99.20%)	29021 (2.90%)	970117 (97.10%)	999138
	65+	1593 (0.58%)	271807 (99.42%)	9758 (3.57%)	263642 (96.43%)	273400
	p value	p<0.001		p<0.001		
Ethnicity	W	8166 (0.83%)	978002 (99.17%)	35403 (3.59%)	950765 (96.41%)	986168
	A	1569 (1.74%)	88628 (98.26%)	2286 (2.53%)	87911 (97.47%)	90197
	B	1014 (2.01%)	49360 (97.99%)	1077 (2.14%)	49297 (97.86%)	50374
	M	300 (1.39%)	21328 (98.61%)	655 (3.03%)	20973 (96.97%)	21628
	O	197 (1.19%)	16349 (98.81%)	307 (1.86%)	16239 (98.14%)	16546
	U	2822 (0.65%)	434631 (99.35%)	7452 (1.70%)	430001 (98.30%)	437453
	p value	p<0.001		p<0.001		
IMD (IMD 1 = Most Deprived)	1	2976 (1.13%)	260564 (98.87%)	8136 (3.09%)	255404 (96.91%)	263540
	2	2286 (0.85%)	265778 (99.15%)	7577 (2.83%)	260487 (97.17%)	268064
	3	2422 (0.83%)	290099 (99.17%)	9337 (3.19%)	283184 (96.81%)	292521
	4	2828 (0.80%)	349733 (99.20%)	10134 (2.87%)	342427 (97.13%)	352561
	5	3556 (0.84%)	422124 (99.16%)	11996 (2.82%)	413684 (97.18%)	425680
	p value	p<0.001		p<0.001		
Comorbidities	0	9409 (0.89%)	1042048 (99.11%)	24487 (2.33%)	1026970 (97.67%)	1051457
	1-2	4029 (0.87%)	458826 (99.13%)	18799 (4.06%)	444056 (95.94%)	462855
	3+	630 (0.72%)	87424 (99.28%)	3894 (4.42%)	84160 (95.58%)	88054
	p value	p<0.001		p<0.001		
Smoking Status	Active Smoker	1424 (0.58%)	244852 (99.42%)	6973 (2.83%)	239303 (97.17%)	246276
	Non-Smoker	5351 (0.94%)	566427 (99.06%)	17666 (3.09%)	554112 (96.91%)	571778
	Ex-Smoker	3933 (0.84%)	467050 (99.16%)	19651 (4.17%)	451332 (95.83%)	470983
	Unknown	3360 (1.07%)	309969 (98.93%)	2890 (0.92%)	310439 (99.08%)	313329
	p value	p<0.001		p<0.001		

Note. N = denominator; n = numerator; % = n/N

Table B.2: *Numerators and denominators with percentages and chi square p value for LRTI and URTI*

		LRTI Yes n (%)	LRTI No n (%)	URTI Yes n (%)	URTI No n (%)	Denominator (N)
Total		56895 (3.55%)	1545471 (96.45%)	143522 (8.96%)	1458844 (91.04%)	1602366
Urban Rural	Conurbation	16835 (3.23%)	503663 (96.77%)	50026 (9.61%)	470472 (90.39%)	520498
	Urban	28782 (3.72%)	743927 (96.28%)	68791 (8.90%)	703918 (91.10%)	772709
	Rural	11278 (3.65%)	297881 (96.35%)	24705 (7.99%)	284454 (92.01%)	309159
	p value	p<0.001		p<0.001		
Gender	M	24742 (3.12%)	768943 (96.88%)	57932 (7.30%)	735753 (92.70%)	793685
	F	32153 (3.98%)	776528 (96.02%)	85590 (10.58%)	723091 (89.42%)	808681
	p value	p<0.001		p<0.001		
Age Band	0-4	5597 (6.00%)	87624 (94.00%)	31580 (33.88%)	61641 (66.12%)	93221
	5-17	3027 (1.28%)	233580 (98.72%)	27128 (11.47%)	209479 (88.53%)	236607
	18-64	25961 (2.60%)	973177 (97.40%)	68118 (6.82%)	931020 (93.18%)	999138
	65+	22310 (8.16%)	251090 (91.84%)	16696 (6.11%)	256704 (93.89%)	273400
	p value	p<0.001		p<0.001		
Ethnicity	W	40323 (4.09%)	945845 (95.91%)	89459 (9.07%)	896709 (90.93%)	986168
	A	2550 (2.83%)	87647 (97.17%)	9992 (11.08%)	80205 (88.92%)	90197
	B	1148 (2.28%)	49226 (97.72%)	4579 (9.09%)	45795 (90.91%)	50374
	M	505 (2.33%)	21123 (97.67%)	2399 (11.09%)	19229 (88.91%)	21628
	O	314 (1.90%)	16232 (98.10%)	1412 (8.53%)	15134 (91.47%)	16546
	U	12055 (2.76%)	425398 (97.24%)	35681 (8.16%)	401772 (91.84%)	437453
	p value	p<0.001		p<0.001		
IMD (IMD 1 = Most Deprived)	1	10626 (4.03%)	252914 (95.97%)	28362 (10.76%)	235178 (89.24%)	263540
	2	9568 (3.57%)	258496 (96.43%)	24708 (9.22%)	243356 (90.78%)	268064
	3	10082 (3.45%)	282439 (96.55%)	25618 (8.76%)	266903 (91.24%)	292521
	4	12241 (3.47%)	340320 (96.53%)	29527 (8.38%)	323034 (91.62%)	352561
	5	14378 (3.38%)	411302 (96.62%)	35307 (8.29%)	390373 (91.71%)	425680
	p value	p<0.001		p<0.001		
Comorbidities	0	22714 (2.16%)	1028743 (97.84%)	98631 (9.38%)	952826 (90.62%)	1051457
	1-2	23584 (5.10%)	439271 (94.90%)	38115 (8.23%)	424740 (91.77%)	462855
	3+	10597 (12.03%)	77457 (87.97%)	6776 (7.70%)	81278 (92.30%)	88054
	p value	p<0.001		p<0.001		
Smoking Status	Active Smoker	10109 (4.11%)	236128 (95.89%)	16789 (6.82%)	229448 (93.18%)	246276
	Non-Smoker	15128 (2.65%)	556643 (97.35%)	34942 (7.42%)	436107 (92.58%)	571778
	Ex-Smoker	24011 (5.10%)	447038 (94.90%)	41180 (7.20%)	530591 (92.80%)	470983
	Unknown	7647 (2.44%)	305662 (97.56%)	50611 (16.15%)	262698 (83.85%)	313329
	p value	p<0.001		p<0.001		

Note. N = denominator; n = numerator; % = n/N

Table B.3: *Numerators and denominators with percentages and chi square p value for AGE and UTI*

		AGE Yes n (%)	AGE No n (%)	UTI Yes n (%)	UTI No n (%)	Denominator (N)
Total		8981 (0.56%)	1593385 (99.44%)	24105 (1.50%)	1578261 (98.50%)	1602366
Urban Rural	Conurbation	3283 (0.63%)	517215 (99.37%)	7375 (1.42%)	513123 (98.58%)	520498
	Urban	4223 (0.55%)	768486 (99.45%)	11674 (1.51%)	761035 (98.49%)	772709
	Rural	1475 (0.48%)	307684 (99.52%)	5056 (1.64%)	304103 (98.36%)	309159
	p value	p<0.001		p<0.001		
Gender	M	4102 (0.52%)	789583 (99.48%)	4390 (0.55%)	789295 (99.45%)	793685
	F	4879 (0.60%)	803802 (99.40%)	19715 (2.44%)	788966 (97.56%)	808681
	p value	p<0.001		p<0.001		
Age Band	0-4	2245 (2.41%)	90976 (97.59%)	670 (0.72%)	92551 (99.28%)	93221
	5-17	1351 (0.57%)	235256 (99.43%)	1345 (0.57%)	235262 (99.43%)	236607
	18-64	4009 (0.40%)	995129 (99.60%)	11844 (1.19%)	987294 (98.81%)	999138
	65+	1376 (0.50%)	272024 (99.50%)	10246 (3.75%)	263154 (96.25%)	273400
	p value	p<0.001		p<0.001		
Ethnicity	W	5211 (0.53%)	980957 (99.47%)	17468 (1.77%)	968700 (98.23%)	986168
	A	806 (0.89%)	89391 (99.11%)	1015 (1.13%)	89182 (98.87%)	90197
	B	334 (0.66%)	50040 (99.34%)	436 (0.87%)	49938 (99.13%)	50374
	M	171 (0.79%)	21457 (99.21%)	171 (0.79%)	21457 (99.21%)	21628
	O	109 (0.66%)	16437 (99.34%)	171 (1.03%)	16375 (98.97%)	16546
	U	2350 (0.54%)	435103 (99.46%)	4844 (1.11%)	432609 (98.89%)	437453
	p value	p<0.001		p<0.001		
IMD (IMD 1 = Most Deprived)	1	2111 (0.80%)	261429 (99.20%)	3976 (1.51%)	259564 (98.49%)	425680
	2	1599 (0.60%)	266465 (99.40%)	3825 (1.43%)	264239 (98.57%)	352561
	3	1589 (0.54%)	290932 (99.46%)	4467 (1.53%)	288054 (98.47%)	292521
	4	1688 (0.48%)	350873 (99.52%)	5334 (1.51%)	347227 (98.49%)	268064
	5	1994 (0.47%)	423686 (99.53%)	6503 (1.53%)	419177 (98.47%)	263540
	p value	p<0.001		p= 0.009		
Comorbidities	0	5892 (0.56%)	1045565 (99.44%)	8918 (0.85%)	1042539 (99.15%)	1051457
	1-2	2428 (0.52%)	460427 (99.48%)	10375 (2.24%)	452480 (97.76%)	462855
	3+	661 (0.75%)	87393 (99.25%)	4812 (5.46%)	83242 (94.54%)	88054
	p value	p<0.001		p<0.001		
Smoking Status	Active Smoker	1062 (0.43%)	245214 (99.57%)	3184 (1.29%)	243092 (98.71%)	246276
	Non-Smoker	2459 (0.43%)	569319 (99.57%)	8504 (1.49%)	563274 (98.51%)	571778
	Ex-Smoker	2280 (0.48%)	468703 (99.52%)	10640 (2.26%)	460343 (97.74%)	470983
	Unknown	3180 (1.01%)	310149 (98.99%)	1777 (0.57%)	311552 (99.43%)	313329
	p value	p<0.001		p<0.001		

Note. N = denominator; n = numerator; % = n/N

Table B.4: *Numerators and denominators with percentages and chi square p value for interaction terms of AR and asthma*

		Allergic Rhinitis Yes n (%)	Allergic Rhinitis No n (%)	Asthma Yes n (%)	Asthma No n (%)	Denominator (N)
Conurbation						
Total		14068 (0.88%)	1588298 (99.12%)	47180 (2.94%)	1555186 (97.06%)	520498
Gender	M	6540 (0.82%)	787145 (99.18%)	20207 (2.55%)	773478 (97.45%)	258700
	F	7528 (0.93%)	801153 (99.07%)	26973 (3.34%)	781708 (96.66%)	261798
	p value	p<0.001		p<0.001		
Age Band	0-4	613 (0.66%)	92608 (99.34%)	1240 (1.33%)	91981 (98.67%)	33317
	5-17	3891 (0.39%)	232716 (23.48%)	7161 (3.03%)	229446 (96.97%)	74625
	18-64	7971 (0.80%)	991167 (99.20%)	29021 (2.90%)	970117 (97.10%)	353960
	65+	1593 (0.58%)	271807 (99.42%)	9758 (3.57%)	263642 (96.43%)	58596
	p value	p<0.001		p<0.001		
Urban						
Total		7971 (0.80%)	991167 (99.20%)	29021 (2.90%)	970117 (97.10%)	772709
Gender	M	1014 (2.01%)	49360 (97.99%)	1077 (2.14%)	49297 (97.86%)	381730
	F	300 (1.39%)	21328 (98.61%)	655 (3.03%)	20973 (96.97%)	390979
	p value	p<0.001		p<0.001		
Age Band	0-4	197 (1.19%)	16349 (98.81%)	307 (1.86%)	16239 (98.14%)	44806
	5-17	2822 (0.65%)	434631 (99.35%)	7452 (1.70%)	430001 (98.30%)	115267
	18-64	2976 (1.13%)	260564 (98.87%)	8136 (3.09%)	255404 (96.91%)	466210
	65+	2286 (0.85%)	265778 (99.15%)	7577 (2.83%)	260487 (97.17%)	146426
	p value	p<0.001		p<0.001		
Rural						
Total		2976 (1.13%)	260564 (98.87%)	8136 (3.09%)	255404 (96.91%)	309159
Gender	M	3556 (0.84%)	422124 (99.16%)	11996 (2.82%)	413684 (97.18%)	153255
	F	9409 (0.89%)	1042048 (99.11%)	24487 (2.33%)	1026970 (97.67%)	155904
	p value	p= 0.128		p<0.001		
Age Band	0-4	4029 (0.87%)	458826 (99.13%)	18799 (4.06%)	444056 (95.94%)	15098
	5-17	630 (0.72%)	87424 (99.28%)	3894 (4.42%)	84160 (95.58%)	46715
	18-64	1423 (0.58%)	244814 (99.42%)	6978 (2.83%)	239259 (97.17%)	178968
	65+	5350 (0.94%)	566421 (99.06%)	17663 (3.09%)	554108 (96.91%)	68378
	p value	p<0.001		p<0.001		

Note. N = denominator; n = numerator; % = n/N

Table B.5: *Numerators and denominators with percentages and chi square p value for interaction terms of LRTI and URTI*

		LRTI Yes n (%)	LRTI No n (%)	URTI Yes n (%)	URTI No n (%)	Denominator (N)
Conurbation						
Total		56895 (3.55%)	1545471 (96.45%)	143522 (8.96%)	1458844 (91.04%)	520498
Gender	M	24742 (3.12%)	768943 (96.88%)	57932 (7.30%)	735753 (92.70%)	258700
	F	32153 (3.98%)	776528 (96.02%)	85590 (10.58%)	723091 (89.42%)	261798
	p value	p<0.001		p<0.001		
Age Band	0-4	5597 (6.00%)	87624 (94.00%)	31580 (33.88%)	61641 (66.12%)	33317
	5-17	3027 (1.28%)	233580 (98.72%)	27128 (11.47%)	209479 (88.53%)	74625
	18-64	25961 (2.60%)	973177 (97.40%)	68118 (6.82%)	931020 (93.18%)	353960
	65+	22310 (8.16%)	251090 (91.84%)	16696 (6.11%)	256704 (93.89%)	58596
	p value	p<0.001		p<0.001		
Urban						
Total		25961 (2.60%)	973177 (97.40%)	68118 (6.82%)	931020 (93.18%)	772709
Gender	M	1148 (2.28%)	49226 (97.72%)	4579 (9.09%)	45795 (90.91%)	381730
	F	505 (2.33%)	21123 (97.67%)	2399 (11.09%)	19229 (88.91%)	390979
	p value	p<0.001		p<0.001		
Age Band	0-4	314 (1.90%)	16232 (98.10%)	1412 (8.53%)	15134 (91.47%)	44806
	5-17	12055 (2.76%)	425398 (97.24%)	35681 (8.16%)	401772 (91.84%)	115267
	18-64	14378 (3.38%)	411302 (96.62%)	28362 (10.76%)	235178 (89.24%)	466210
	65+	12241 (3.47%)	340320 (96.53%)	24708 (9.22%)	243356 (90.78%)	146426
	p value	p<0.001		p<0.001		
Rural						
Total		14378 (3.38%)	411302 (96.62%)	28362 (10.76%)	235178 (89.24%)	309159
Gender	M	10626 (4.03%)	252914 (95.97%)	35307 (8.29%)	390373 (91.71%)	153255
	F	22714 (2.16%)	1028743 (97.84%)	98631 (9.38%)	952826 (90.62%)	155904
	p value	p<0.001		p<0.001		
Age Band	0-4	23584 (5.10%)	439271 (94.90%)	38115 (8.23%)	424740 (91.77%)	15098
	5-17	10597 (12.03%)	77457 (87.97%)	6776 (7.70%)	81278 (92.30%)	46715
	18-64	10109 (4.11%)	236128 (95.89%)	16789 (6.82%)	229448 (93.18%)	178968
	65+	15128 (2.65%)	556643 (97.35%)	34942 (7.42%)	436107 (92.58%)	68378
	p value	p<0.001		p<0.001		

Note. N = denominator; n = numerator; % = n/N

Table B.6: *Numerators and denominators with percentages and chi square p value for interaction terms of AGE and UTI*

		AGE Yes n (%)	AGE No n (%)	UTI Yes n (%)	UTI No n (%)	Denominator (N)
Conurbation						
Total		8981 (0.56%)	1593385 (99.44%)	24105 (1.50%)	1578261 (98.50%)	520498
Gender	M	4102 (0.52%)	789583 (99.48%)	4390 (0.55%)	789295 (99.45%)	258700
	F	4879 (0.60%)	803802 (99.40%)	19715 (2.44%)	788966 (97.56%)	261798
	p value	p<0.001		p<0.001		
Age Band	0-4	2245 (2.41%)	90976 (97.59%)	670 (0.72%)	92551 (99.28%)	33317
	5-17	1351 (0.57%)	235256 (99.43%)	1345 (0.57%)	235262 (99.43%)	74625
	18-64	4009 (0.40%)	995129 (99.60%)	11844 (1.19%)	987294 (98.81%)	353960
	65+	1376 (0.50%)	272024 (99.50%)	10246 (3.75%)	263154 (96.25%)	58596
	p value	p<0.001		p<0.001		
Urban						
Total		4009 (0.40%)	995129 (99.60%)	11844 (1.19%)	987294 (98.81%)	772709
Gender	M	334 (0.66%)	50040 (99.34%)	436 (0.87%)	49938 (99.13%)	381730
	F	171 (0.79%)	21457 (99.21%)	171 (0.79%)	21457 (99.21%)	390979
	p value	p<0.001		p<0.001		
Age Band	0-4	109 (0.66%)	16437 (99.34%)	171 (1.03%)	16375 (98.97%)	44806
	5-17	2350 (0.54%)	435103 (99.46%)	4844 (1.11%)	432609 (98.89%)	115267
	18-64	2111 (0.80%)	261429 (99.20%)	3976 (1.51%)	259564 (98.49%)	466210
	65+	1599 (0.60%)	266465 (99.40%)	3825 (1.43%)	264239 (98.57%)	146426
	p value	p<0.001		p<0.001		
Rural						
Total		2111 (0.80%)	261429 (99.20%)	3976 (1.51%)	259564 (98.49%)	309159
Gender	M	1994 (0.47%)	423686 (99.53%)	6503 (1.53%)	419177 (98.47%)	153255
	F	5892 (0.56%)	1045565 (99.44%)	8918 (0.85%)	1042539 (99.15%)	155904
	p value	p= 0.052		p<0.001		
Age Band	0-4	2428 (0.52%)	460427 (99.48%)	10375 (2.24%)	452480 (97.76%)	15098
	5-17	661 (0.75%)	87393 (99.25%)	4812 (5.46%)	83242 (94.54%)	46715
	18-64	1060 (0.43%)	245177 (99.57%)	3184 (1.29%)	243053 (98.71%)	178968
	65+	2460 (0.43%)	569311 (99.57%)	8504 (1.49%)	563267 (98.51%)	68378
	p value	p<0.001		p<0.001		

Note. N = denominator; n = numerator; % = n/N

Table B.7: Numerators and denominators with percentages and chi square p value for urban/rural/conurbation living

		Rural Yes n(%)	Urban Yes n(%)	Conurbation Yes n(%)	Denominator (N)
Total		309159 (19.29%)	772709 (48.22%)	520498 (32.48%)	1602366
Gender	M	153255 (19.31%)	381730 (48.10%)	258700 (32.59%)	793685
	F	155904 (19.28%)	390979 (48.35%)	261798 (32.37%)	808681
	p value		p= 0.003		
Age Band	0-4	15098 (16.20%)	44806 (48.06%)	33317 (35.74%)	93221
	5-17	46715 (19.74%)	115267 (48.72%)	74625 (31.54%)	236607
	18-64	178968 (17.91%)	466210 (46.66%)	353960 (35.43%)	999138
	65+	68378 (25.01%)	146426 (53.56%)	58596 (21.43%)	273400
	p value		p<0.001		
Ethnicity	W	204954 (20.78%)	491156 (49.80%)	290058 (29.41%)	986168
	A	2297 (2.55%)	27073 (30.02%)	60827 (67.44%)	90197
	B	528 (1.05%)	5156 (10.24%)	44690 (88.72%)	50374
	M	1265 (5.85%)	6617 (30.59%)	13746 (63.56%)	21628
	O	629 (3.80%)	4764 (28.79%)	11153 (67.41%)	16546
	U	99486 (22.74%)	237943 (54.39%)	100024 (22.87%)	437453
	p value		p<0.001		
IMD (IMD 1 = Most Deprived)	1	5654 (2.15%)	105857 (40.17%)	152029 (57.69%)	263540
	2	29156 (10.88%)	110223 (41.12%)	128685 (48.01%)	268064
	3	70857 (24.22%)	142241 (48.63%)	79423 (27.15%)	292521
	4	99769 (28.30%)	172960 (49.06%)	79832 (22.64%)	352561
	5	103723 (24.37%)	241428 (56.72%)	80529 (18.92%)	425680
	p value		p<0.001		
Comorbidities	0	196594 (18.70%)	487295 (46.34%)	367568 (34.96%)	1051457
	1-2	94283 (20.37%)	237916 (51.40%)	130656 (28.23%)	462855
	3+	18282 (20.76%)	47498 (53.94%)	22274 (25.30%)	88054
	p value		p<0.001		
Smoking Status	Active Smoker	39890 (16.20%)	122961 (49.93%)	83425 (33.87%)	246276
	Non-Smoker	103661 (18.13%)	260715 (45.60%)	207402 (36.27%)	571778
	Ex-Smoker	106842 (22.68%)	231517 (49.16%)	132624 (28.16%)	470983
	Unknown	58766 (18.76%)	157516 (50.27%)	97047 (30.97%)	313329
	p value		p<0.001		

Note. N = denominator; n = numerator; % = n/N
