# Supplementary material for: Conurbation, Urban, and Rural Living as Determinants of Allergies and Infectious Diseases: Royal College of General Practitioners Research and Surveillance Centre Annual Report 2016-2017
Source: JMIR Public Health Surveill. 2018 Nov 26;4(4):e11354. doi: 10.2196/11354 (PMC6288591; doi:10.2196/11354)

Supplementary File C : Figures
Forest plots for age bands and each of the conditions

Figure C.1: Forest plot showing odds ratio for certain conditions based on living area.

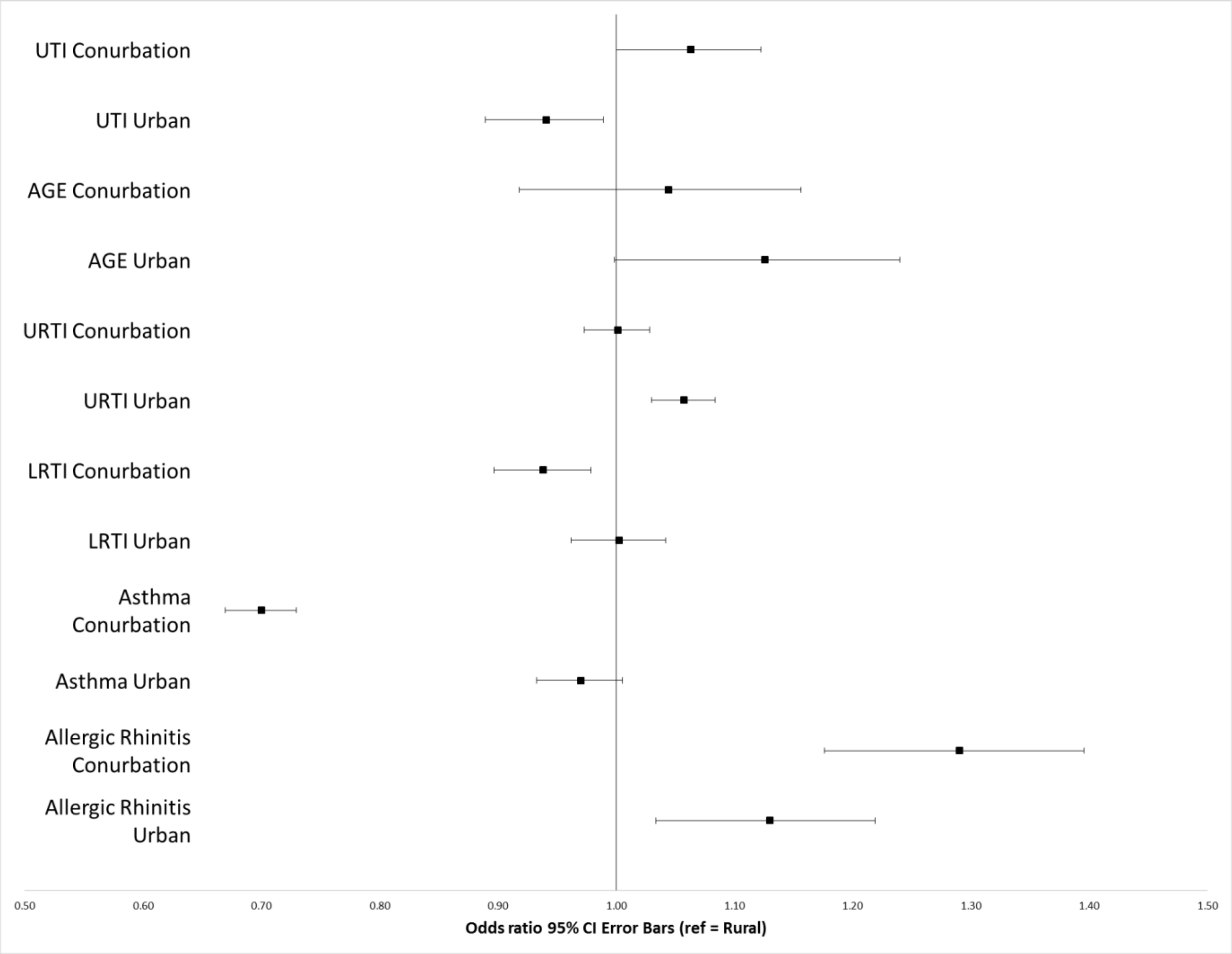

Figure C.2: Forest plot showing odds ratio for certain conditions based on living area and age 0-4 years

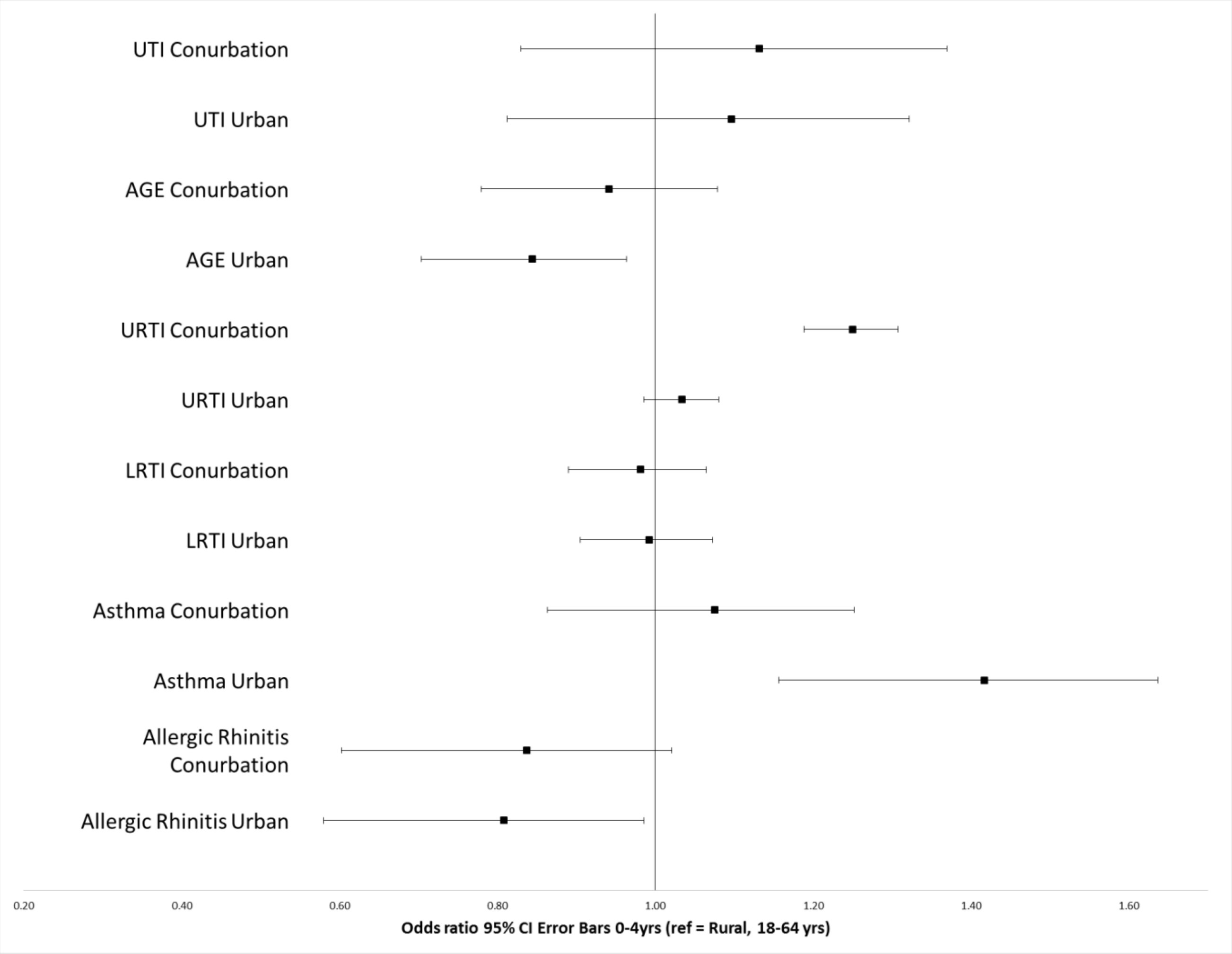

Figure C.3: Forest plot showing odds ratio for certain conditions based on living area and age 5-17 years

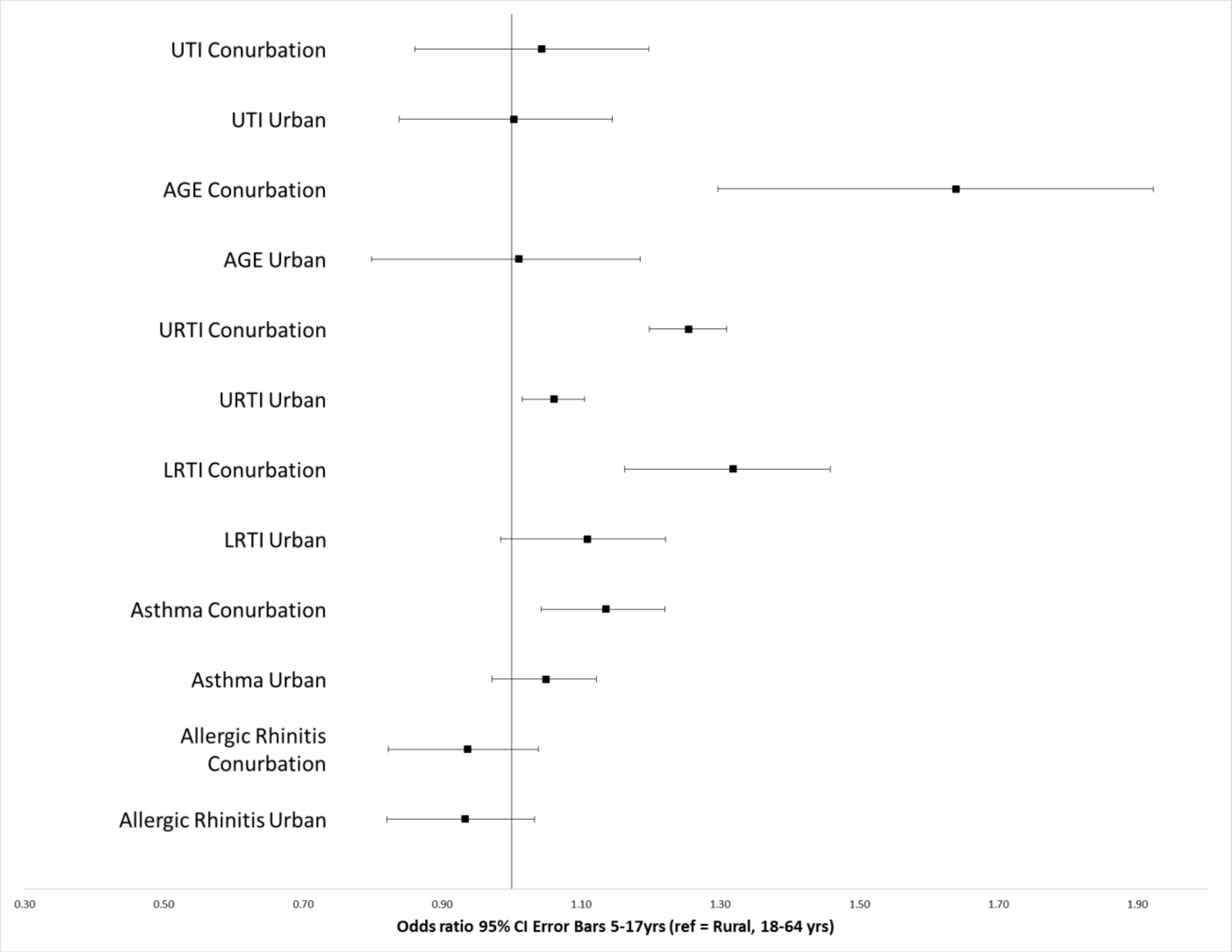

Figure C.4: Forest plot showing odds ratio for certain conditions based on living area and age 65+ years

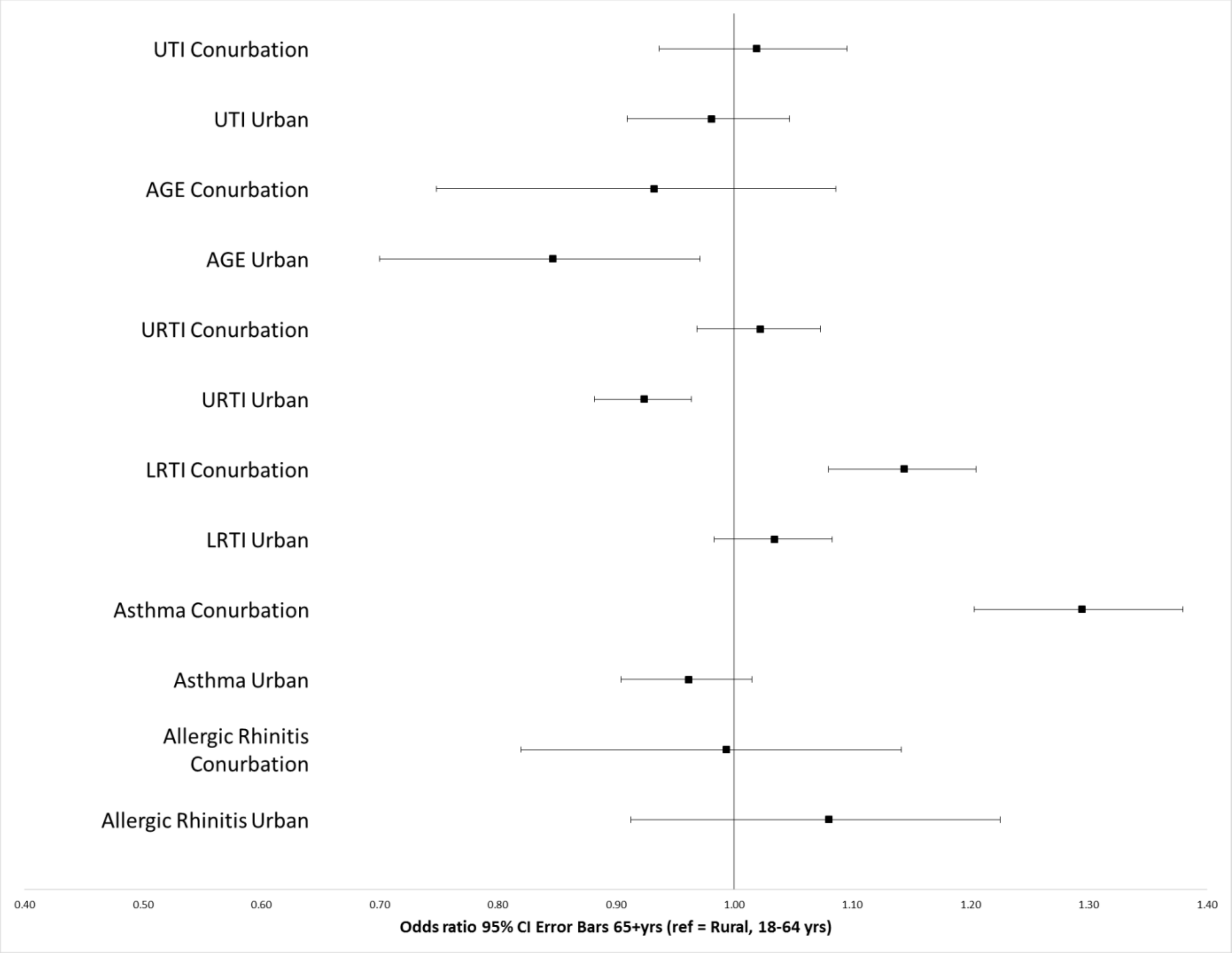

Bar graphs for RSC Population

Figure C.5: RSC Population age bands by living area

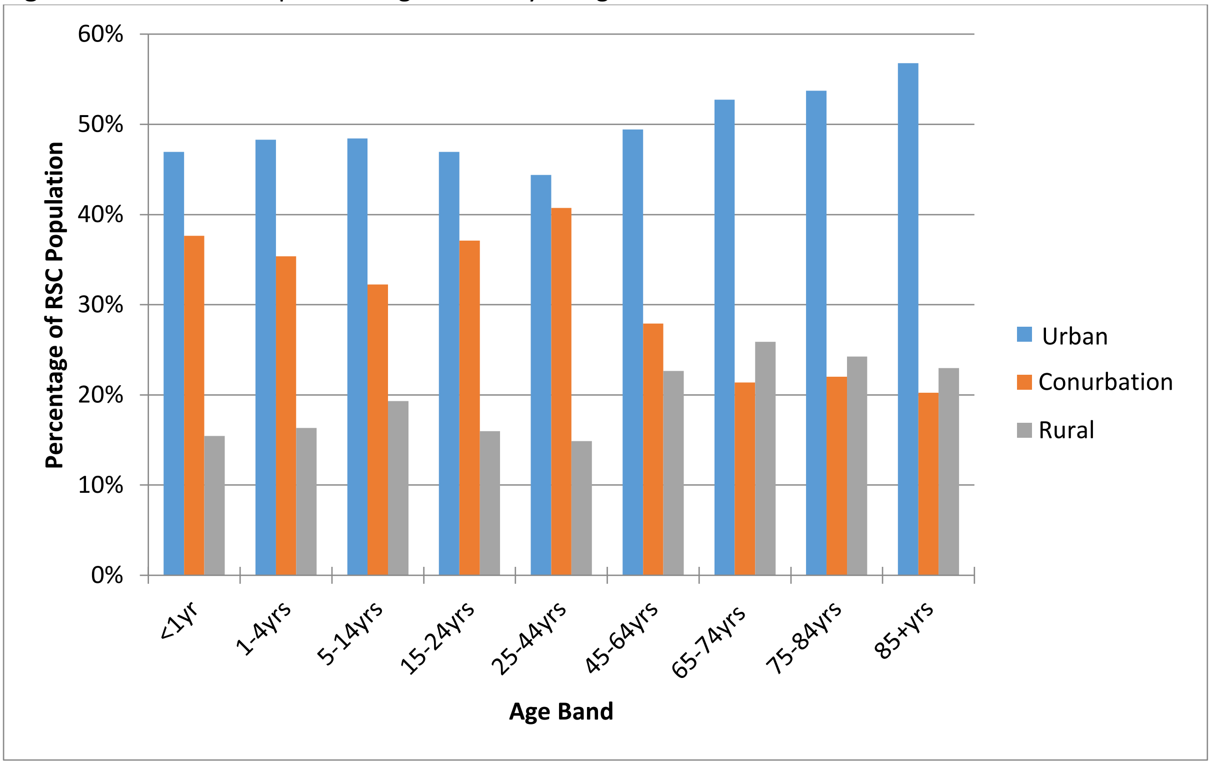

Figure C.6: RSC Population IMD Quintile by living area

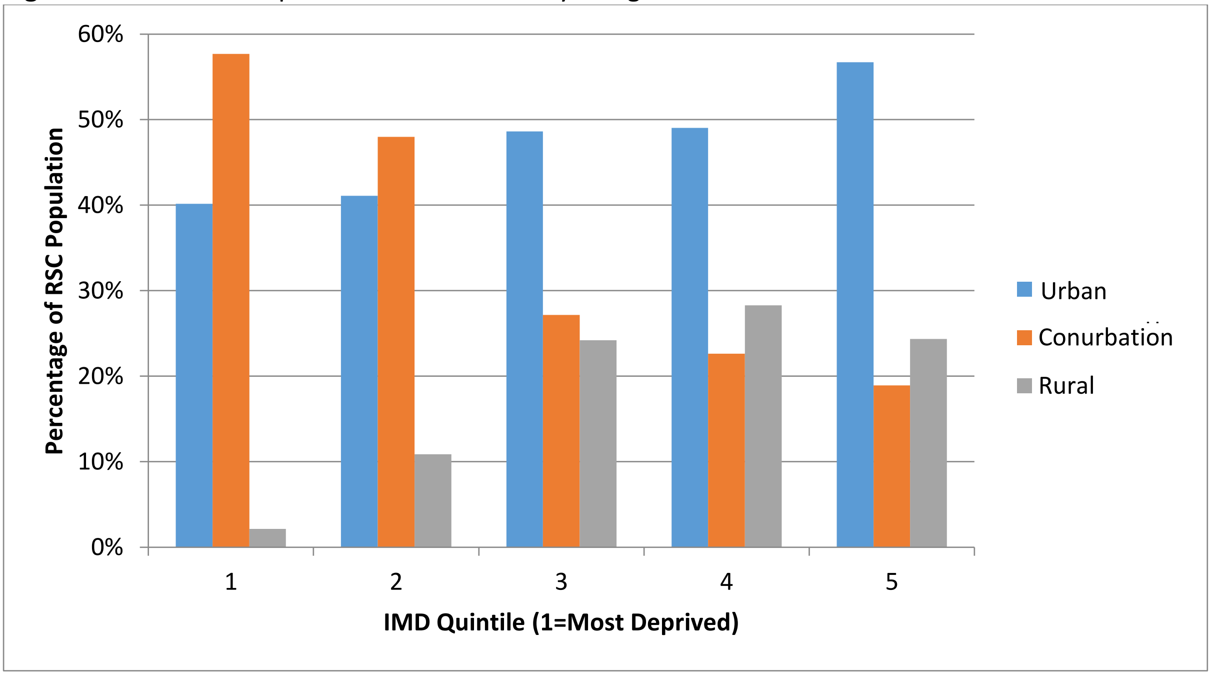

Figure C.7: RSC Population ethnicity by living area

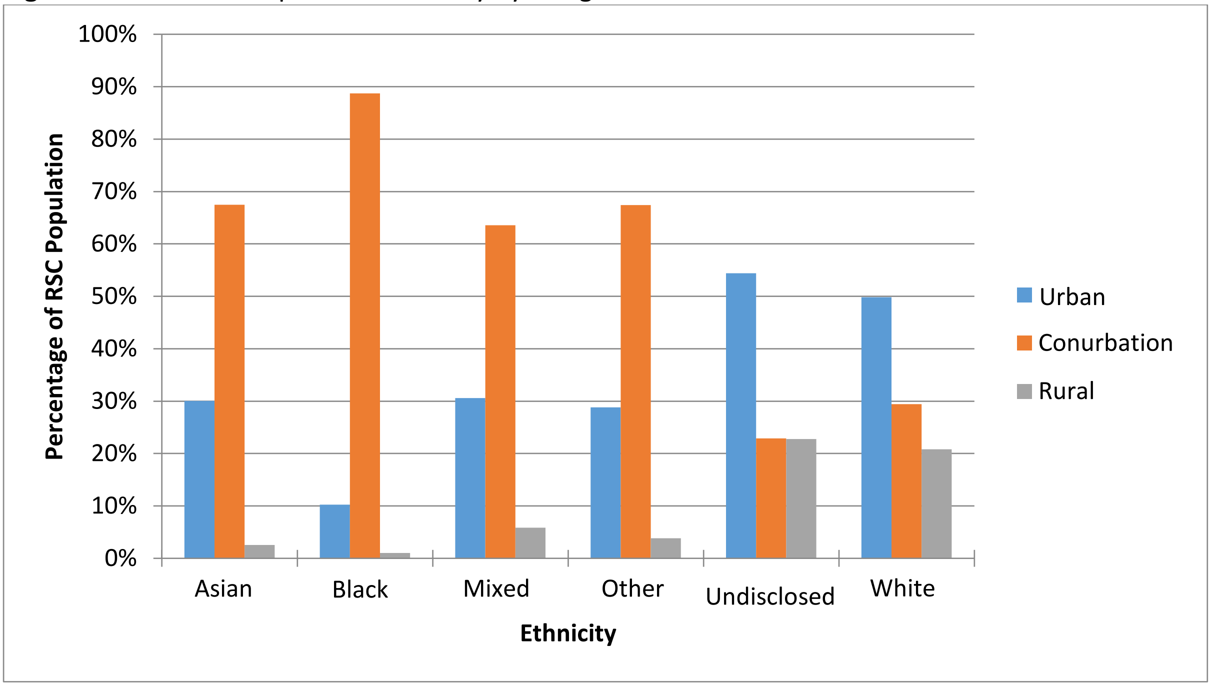

Supplement: Multimedia Appendix 3 [file publichealth_v4i4e11354_app3.pdf]
