# Supplementary material for: Conurbation, Urban, and Rural Living as Determinants of Allergies and Infectious Diseases: Royal College of General Practitioners Research and Surveillance Centre Annual Report 2016-2017
Source: JMIR Public Health Surveill. 2018 Nov 26;4(4):e11354. doi: 10.2196/11354 (PMC6288591; doi:10.2196/11354)
Supplement: Multimedia Appendix 4 [file publichealth_v4i4e11354_app4.pdf]

Supplementary File D
Logistic regression results

Table D.1: Logistic regression output where outcome is presentation of allergic rhinitis

**Allergic Rhinitis**	**Estimate**	**OR**	**LCI**	**UCI**	**p-value**
(Intercept)	-4.97	0.01	0.01	0.01	0.00
Urban	0.12	1.13	1.04	1.23	0.00
Conurbation	0.26	1.29	1.19	1.41	0.00
0-4yrs	0.47	1.60	1.28	2.00	0.00
5-17yrs	1.19	3.28	2.95	3.66	0.00
65+yrs	-0.46	0.63	0.56	0.72	0.00
Male	-0.03	0.97	0.89	1.05	0.43
Asian Ethnicity	0.67	1.95	1.84	2.07	0.00
Black Ethnicity	0.74	2.10	1.96	2.25	0.00
Mixed Ethnicity	0.35	1.42	1.27	1.60	0.00
Other Ethnicity	0.28	1.33	1.15	1.53	0.00
Unknown Ethnicity	-0.27	0.77	0.73	0.80	0.00
IMDQuintile2	-0.20	0.82	0.78	0.87	0.00
IMDQuintile3	-0.10	0.90	0.86	0.96	0.00
IMDQuintile4	-0.09	0.92	0.87	0.97	0.00
IMDQuintile5	-0.06	0.94	0.90	1.00	0.03
1-2 Comorbidities	0.30	1.35	1.29	1.41	0.00
3+ Comorbidities	0.29	1.33	1.22	1.46	0.00
Ex-smoker	-0.34	0.71	0.67	0.75	0.00
Non-smoker	0.11	1.11	1.06	1.16	0.00
Smoking unknown	-0.43	0.65	0.61	0.69	0.00
Urban:0-4yrs	-0.21	0.81	0.63	1.04	0.09
**Conurbation:0-4yrs**	**-0.18**	**0.84**	**0.65**	**1.07**	**0.16**
Urban:5-17yrs	-0.07	0.93	0.83	1.05	0.23
**Conurbation:5-17yrs**	**-0.07**	**0.94**	**0.83**	**1.05**	**0.27**
Urban:65+yrs	0.08	1.08	0.94	1.25	0.29
**Conurbation:65+yrs**	**-0.01**	**0.99**	**0.85**	**1.17**	**0.94**
Urban:Male	-0.03	0.97	0.88	1.07	0.49
**Conurbation:Male**	**-0.11**	**0.89**	**0.81**	**0.99**	**0.03**

Note. Interaction terms are shaded grey. Conurbation vs Rural interaction terms are in bold.

Table D.2: Logistic regression output where outcome is presentation of asthma

Asthma	Estimate	OR	LCI	UCI	p-value
(Intercept)	-3.34	0.04	0.03	0.04	0.00
Urban	-0.03	0.97	0.93	1.01	0.11
Conurbation	-0.36	0.70	0.67	0.73	0.00
0-4yrs	1.13	3.10	2.67	3.61	0.00
5-17yrs	1.47	4.37	4.10	4.65	0.00
65+yrs	-0.20	0.82	0.78	0.86	0.00
Male	-0.21	0.81	0.78	0.84	0.00
Asian Ethnicity	-0.16	0.85	0.81	0.89	0.00
Black Ethnicity	-0.35	0.70	0.66	0.75	0.00
Mixed Ethnicity	0.00	1.00	0.92	1.08	0.92
Other Ethnicity	-0.48	0.62	0.55	0.69	0.00
Unknown Ethnicity	-0.62	0.54	0.53	0.55	0.00
IMDQuintile2	-0.08	0.93	0.90	0.96	0.00
IMDQuintile3	-0.03	0.97	0.94	1.00	0.07
IMDQuintile4	-0.15	0.86	0.84	0.89	0.00
IMDQuintile5	-0.17	0.85	0.82	0.87	0.00
1-2 Commorbidities	0.44	1.56	1.53	1.59	0.00
3+ Commorbidities	0.54	1.71	1.64	1.78	0.00
Ex-smoker	-0.01	0.99	0.97	1.02	0.73
Non-smoker	0.38	1.47	1.43	1.50	0.00
Smoking unknown	-2.17	0.11	0.11	0.12	0.00
Urban:0-4yrs	0.35	1.42	1.20	1.68	0.00
**Conurbation:0-4yrs**	**0.07**	**1.08**	**0.90**	**1.29**	**0.43**
Urban:5-17yrs	0.05	1.05	0.98	1.13	0.18
**Conurbation:5-17yrs**	**0.13**	**1.14**	**1.05**	**1.23**	**0.00**
Urban:65+yrs	-0.04	0.96	0.91	1.02	0.18
**Conurbation:65+yrs**	**0.26**	**1.29**	**1.21**	**1.39**	**0.00**
Urban:Male	0.00	1.00	0.95	1.05	0.97
**Conurbation:Male**	**0.01**	**1.01**	**0.96**	**1.07**	**0.60**

Note. Interaction terms are shaded grey. Conurbation vs Rural interaction terms are in bold.

Table D.3: Logistic regression output where outcome is presentation of LRTI

LRTI	Estimate	OR	LCI	UCI	p-value

(Intercept)	-3.79	0.02	0.02	0.02	0.00
Urban	0.00	1.00	0.96	1.04	0.89
Conurbation	-0.06	0.94	0.90	0.98	0.00
0-4yrs	1.77	5.88	5.40	6.40	0.00
5-17yrs	-0.10	0.91	0.82	1.00	0.05
65+yrs	0.66	1.94	1.86	2.02	0.00
Male	-0.16	0.86	0.82	0.89	0.00
Asian Ethnicity	-0.05	0.95	0.91	0.99	0.02
Black Ethnicity	-0.35	0.70	0.66	0.75	0.00
Mixed Ethnicity	-0.29	0.75	0.69	0.82	0.00
Other Ethnicity	-0.42	0.66	0.59	0.73	0.00
Unknown Ethnicity	-0.23	0.80	0.78	0.81	0.00
IMDQuintile2	-0.12	0.89	0.86	0.92	0.00
IMDQuintile3	-0.22	0.80	0.78	0.82	0.00
IMDQuintile4	-0.23	0.80	0.78	0.82	0.00
IMDQuintile5	-0.23	0.80	0.77	0.82	0.00
1-2 Commorbidities	0.71	2.03	1.98	2.07	0.00
3+ Commorbidities	1.30	3.67	3.56	3.78	0.00
Ex-smoker	0.39	1.47	1.44	1.51	0.00
Non-smoker	0.30	1.35	1.32	1.38	0.00
Smoking unknown	-0.37	0.69	0.66	0.73	0.00
Urban:0-4yrs	-0.01	0.99	0.91	1.08	0.85
**Conurbation:0-4yrs**	**-0.02**	**0.98**	**0.90**	**1.07**	**0.68**
Urban:5-17yrs	0.10	1.11	1.00	1.23	0.06
**Conurbation:5-17yrs**	**0.28**	**1.32**	**1.18**	**1.47**	**0.00**
Urban:65+yrs	0.03	1.03	0.99	1.09	0.17
**Conurbation:65+yrs**	**0.13**	**1.14**	**1.08**	**1.21**	**0.00**
Urban:Male	-0.05	0.95	0.91	1.00	0.04
**Conurbation:Male**	**-0.08**	**0.93**	**0.88**	**0.97**	**0.00**

Note. Interaction terms are shaded grey. Conurbation vs Rural interaction terms are in bold.

Table D.4: Logistic regression output where outcome is presentation of URTIX**URTI**	**Estimate S**	**OR**	**LCI**	**UCI**	**p-value**
(Intercept)	-2.47	0.08	0.08	0.09	0.00
UrbanRuralUrban	0.06	1.06	1.03	1.08	0.00
UrbanRuralConurbation	0.00	1.00	0.97	1.03	0.93
AgeBand0-4yrs	2.27	9.70	9.27	10.15	0.00
AgeBand5-17yrs	0.82	2.27	2.18	2.36	0.00
AgeBand65+yrs	-0.30	0.74	0.71	0.77	0.00
SexM	-0.43	0.65	0.64	0.67	0.00
EthnicityCodeA	0.19	1.20	1.18	1.23	0.00
EthnicityCodeB	-0.18	0.83	0.80	0.86	0.00
EthnicityCodeM	-0.13	0.88	0.84	0.92	0.00
EthnicityCodeO	-0.14	0.87	0.82	0.92	0.00
EthnicityCodeU	-0.26	0.77	0.76	0.79	0.00
IMDQuintile2	-0.11	0.89	0.88	0.91	0.00
IMDQuintile3	-0.13	0.87	0.86	0.89	0.00
IMDQuintile4	-0.16	0.85	0.83	0.87	0.00
IMDQuintile5	-0.17	0.84	0.83	0.86	0.00
Commorbidities1	0.37	1.45	1.43	1.47	0.00
Commorbidities2	0.48	1.61	1.56	1.66	0.00
SmokingStatusEx-smoker	0.03	1.03	1.01	1.05	0.00
SmokingStatusNon-smoker	0.15	1.16	1.14	1.18	0.00
SmokingStatusUnknown	-0.18	0.84	0.82	0.85	0.00
Urban:0-4yrs	0.03	1.03	0.99	1.08	0.16
**Conurbation:0-4yrs**	0.22	1.25	1.19	1.31	0.00
Urban:5-17yrs	0.06	1.06	1.02	1.11	0.01
**Conurbation:5-17yrs**	0.23	1.25	1.20	1.31	0.00
Urban:65+yrs	-0.08	0.92	0.88	0.97	0.00
**Conurbation:65+yrs**	0.02	1.02	0.97	1.08	0.40
Urban:Male	-0.01	0.99	0.96	1.03	0.75
**Conurbation:Male**	0.00	1.00	0.97	1.04	0.90

Note. Interaction terms are shaded grey. Conurbation vs Rural interaction terms are in bold.

Table D.5: Logistic regression output where outcome is presentation of AGE X**IID**	**Estimate**	**OR**	**LCI**	**UCI**	**p-value**
(Intercept)	-5.52	0.00	0.00	0.00	0.00
UrbanRuralUrban	0.12	1.13	1.01	1.25	0.03
UrbanRuralConurbation	0.04	1.04	0.93	1.17	0.46
AgeBand0-4yrs	2.25	9.49	8.12	11.09	0.00
AgeBand5-17yrs	0.46	1.58	1.32	1.89	0.00
AgeBand65+yrs	0.06	1.06	0.92	1.22	0.40
SexM	-0.09	0.91	0.82	1.01	0.08
EthnicityCodeA	0.49	1.63	1.51	1.77	0.00
EthnicityCodeB	0.04	1.04	0.92	1.16	0.54
EthnicityCodeM	0.11	1.12	0.96	1.30	0.16
EthnicityCodeO	0.17	1.19	0.98	1.44	0.08
EthnicityCodeU	-0.07	0.93	0.88	0.98	0.00
IMDQuintile2	-0.23	0.79	0.74	0.85	0.00
IMDQuintile3	-0.28	0.76	0.71	0.81	0.00
IMDQuintile4	-0.39	0.68	0.63	0.73	0.00
IMDQuintile5	-0.40	0.67	0.63	0.71	0.00
Commorbidities1	0.45	1.57	1.48	1.66	0.00
Commorbidities2	0.83	2.29	2.08	2.54	0.00
SmokingStatusEx-smoker	0.04	1.04	0.96	1.12	0.35
SmokingStatusNon-smoker	0.14	1.15	1.08	1.22	0.00
SmokingStatusUnknown	-0.09	0.91	0.84	0.99	0.03
Urban:0-4yrs	-0.17	0.84	0.72	0.98	0.03
**Conurbation:0-4yrs**	-0.06	0.94	0.80	1.10	0.45
Urban:5-17yrs	0.01	1.01	0.84	1.22	0.92
**Conurbation:5-17yrs**	0.49	1.64	1.36	1.98	0.00
Urban:65+yrs	-0.17	0.85	0.72	0.99	0.04
**Conurbation:65+yrs**	-0.07	0.93	0.78	1.12	0.45
Urban:Male	-0.05	0.95	0.84	1.07	0.42
**Conurbation:Male**	-0.10	0.91	0.80	1.03	0.12

Note. Interaction terms are shaded grey. Conurbation vs Rural interaction terms are in bold.

Table D.6: Logistic regression output where outcome is presentation of UTI

**UTI**	**Estimate**	**OR**	**LCI**	**UCI**	**p-value**
(Intercept)	-4.05	0.02	0.02	0.02	0.00
Urban	-0.06	0.94	0.89	0.99	0.02
Conurbation	0.06	1.06	1.00	1.13	0.04
0-4yrs	0.07	1.07	0.87	1.33	0.52
5-17yrs	-0.19	0.82	0.72	0.95	0.01
65+yrs	0.68	1.98	1.86	2.10	0.00
Male	-1.38	0.25	0.23	0.27	0.00
Asian Ethnicity	-0.16	0.85	0.79	0.91	0.00
Black Ethnicity	-0.52	0.60	0.54	0.66	0.00
Mixed Ethnicity	-0.43	0.65	0.56	0.76	0.00
Other Ethnicity	-0.16	0.85	0.73	0.99	0.04
Unknown Ethnicity	-0.21	0.81	0.78	0.83	0.00
IMDQuintile2	-0.08	0.92	0.88	0.97	0.00
IMDQuintile3	-0.08	0.93	0.89	0.97	0.00
IMDQuintile4	-0.11	0.89	0.85	0.93	0.00
IMDQuintile5	-0.09	0.92	0.88	0.96	0.00
1-2 Commorbidities	0.56	1.76	1.70	1.82	0.00
3+ Commorbidities	1.23	3.43	3.28	3.59	0.00
Ex-smoker	-0.11	0.90	0.86	0.94	0.00
Non-smoker	0.09	1.10	1.06	1.13	0.00
Smoking unknown	-0.38	0.68	0.63	0.74	0.00
Urban:0-4yrs	0.09	1.10	0.87	1.38	0.43
**Conurbation:0-4yrs**	0.12	1.13	0.89	1.43	0.31
Urban:5-17yrs	0.00	1.00	0.86	1.17	0.97
**Conurbation:5-17yrs**	0.04	1.04	0.89	1.22	0.61
Urban:65+yrs	-0.02	0.98	0.91	1.05	0.58
**Conurbation:65+yrs**	0.02	1.02	0.94	1.10	0.63
Urban:Male	-0.01	0.99	0.91	1.08	0.90
**Conurbation:Male**	-0.21	0.81	0.74	0.89	0.00

Note. Interaction terms are shaded grey. Conurbation vs Rural interaction terms are in bold.
